# The Biogeography of Fungal Communities Across Different Chinese Wine-Producing Regions Associated With Environmental Factors and Spontaneous Fermentation Performance

**DOI:** 10.3389/fmicb.2021.636639

**Published:** 2022-02-25

**Authors:** Ruilong Li, Siyu Yang, Mengyuan Lin, Sijiang Guo, Xiaoyu Han, Mengmeng Ren, Le Du, Yinghui Song, Yilin You, Jicheng Zhan, Weidong Huang

**Affiliations:** ^1^Beijing Key Laboratory of Viticulture and Enology, College of Food Science and Nutritional Engineering, China Agricultural University, Beijing, China; ^2^Wuhan Donghu Big Data Trading Center Co., Ltd., Wuhan, China; ^3^Penglai Grape and Wine Industry Development Service Center, Yantai, China

**Keywords:** Marselan, fungal community, high-throughput sequencing, spontaneous fermentation, geoclimatic element

## Abstract

Chinese Marselan grapes are believed to possess the potential to become a characteristic regional variety, whose quality is internationally recognized. The fermentation-related mycobiota from six climatically diverse Marselan-producing regions in China were analyzed *via* high-throughput sequencing (HTS), while the influence of environmental factors was evaluated as well. The results implied that the phyla Ascomycota and genus *Aureobasidium* dominated the fungal communities in 166 Marselan must and fermented samples. Significant differences were detected in the fungal microbiota from the regions, as well as the wineries, while these discrepancies decreased as the fermentation progressed. Moreover, the discrepancy in fungal communities between the wineries exceeded the variation involving the regions. Geoclimatic elements (Gc) and physicochemical indexes (Pi) exerted a significant effect on the fungal must consortium, explaining 58.17% of the taxonomic information. Furthermore, a correlation was proposed between the spontaneous fermentation performance and their association with fungal taxonomic composition. In addition to depicting a fundamental landscape of fungal biogeography patterns across Chinese main wine-producing regions, we firstly proposed the correlation between the must polyphenol content and fungal microbiota, which may provide a new strategy for harnessing autochthonous “microbial terroir.”

## Introduction

As a product with both cultural and economic value, wine is appreciated for its regional differences, also known as terroir (Van Leeuwen and Seguin, 2006). Previously, wine terroir was attributed to the soil and the vineyard environment, until the concept of “microbial terroir” was presented (Gilbert et al., 2014). With the development of gene sequencing technology, the microbial regional distribution pattern has been verified in several wine-producing regions worldwide (Bokulich et al., 2014; De Filippis et al., 2017; Knight et al., 2020), and further studies have proved the correlation between regional “microbial terroir” and wine style (Bokulich et al., 2016; Liu et al., 2020). High-throughput sequencing (HTS) has been widely used in microbial diversity research and has been proven an effective method for studying microorganisms during fermentation (Wang et al., 2015; Morgan et al., 2017). Compared with bacteria, fungi have a greater influence on wine style (Liu et al., 2020), and the fermentation process has a more substantial impact on the mycobiota (Pinto et al., 2015), while the fungal must consortium displays higher annual stability (Bokulich et al., 2014).

The distribution of Chinese wine-producing regions is extensive, covering 179 counties, with a range of 24–47°N latitude (LAT) and 76–132°E longitude (LNT). Significant differences in climate are evident between the producing regions, and various microclimates exist in each area. Unlike most of the global wine-producing regions that benefit from the oceanic climate and Mediterranean climate, Chinese wine-producing regions are widely distributed comprising diverse climate types and primarily affected by the continental monsoon climate characterized by hot and rainy summers, and cold and dry winters (Wang et al., 2018). To date, research in China has only focused on partial regions (Gao et al., 2019; Zhang et al., 2019a), and knowledge regarding the fungal microbiome exposed to different climatic conditions nationwide remains minimal (Zhang et al., 2017; Li et al., 2018; Shi et al., 2020).

Since being introduced into China in 2001, due to its excellent adaptability and fermentative characteristics, the planting area of Marselan has gradually expanded. At present, China is one of the countries with the largest Marselan planting area (Ma, 2017). The flavor and quality of wine produced from Marselan are exceedingly popular with consumers and experts both domestically and abroad, and it is expected to become a representative variety of the Chinese wine industry (Jiao and Ouyang, 2019). As an emerging variety, current studies primarily focus on its fermentation and cultivation (Li et al., 2019), and information regarding the mycobiota during the fermentation process of Marselan grape from different regions is scarce (Lyu et al., 2019).

In this study, the fungal communities of the must and fermented samples from six major Marselan regions (fifteen wineries) in China are assessed *via* HTS analysis for internal transcribed spacer II (ITS2) genes. Furthermore, ordination analysis are applied to test the fungal community homeomorphism among regions and wineries. In addition, correlation analysis is used to explore the relationship between the mycobiota and geoclimatic elements (Gc), as well as the influence of fungal composition on the physicochemical indexes (Pi) of spontaneously fermented wine. In conclusion, we conduct a comprehensive study to uncover the biogeographic distribution pattern of fungal communities and its influencing factors in different Marselan producing regions.

## Materials and Methods

### Grape Sampling and Laboratory-Scale Spontaneous Fermentation

The grape samples were collected from six prime Marselan producing regions in China, namely, Ningxia’s Helan Mountain’s Eastern Region (NX), Fangshan District in Beijing (FS), Huailai County in Hebei Province (HL), Xiangning County in Shanxi province (SX), Penglai City in Shandong Province (YT), and Changli County in Hebei Province (CL) in 2017 vintage. Among them, the grapevines in the SX and YT could overwinter without soil burial, but the grapevines in FS, HL, CL, and NX had to be buried with soil for protection against the winter chill (Wang et al., 2018). For each region, three of the most representative wineries (vineyards) were considered for sampling, except for SX and CL, in which only one and two wineries, respectively, had planted Marselan grapevines (Supplementary Table 1).

For all regions, the Marselan grape samples were picked on a sunny morning within 3 days of harvest. Private wine producers authorized the sampling, and the field study did not involve any endangered or protected species. For each winery, three bags of healthy and undamaged grapes, about 1 kg each, were respectively, collected using sterilized garden scissors. To ensure the representativeness of the sampling, multiple bunches of grape samples were randomly picked from different positions in the vineyards, containing different ranks and orientations. These samples were placed into sterile plastic bags and transported to the laboratory chilled on ice within 1 day (Pinto et al., 2015).

Here, 45 bags of grape samples from fifteen wineries (three parallel samples per winery) were aseptically destemmed, and hand-squeezed in a clean bench, after which 350 mL of grape must and pomace from each sample were placed into sterile 500 mL jars, sealed with sterile sealing films. The physical and chemical indexes of the initial must is shown in Supplementary Table 2. Stationary spontaneous fermentation was performed at room temperature (controlled at 25 ± 2°C) (Portillo and Mas, 2016).

According to the pre-experiment of 2016 and the fermentation process of 2017, the Marselan must and krausen samples were collected on days 0 (must), 3, 5, and 8 for microbial diversity analysis. It should be noted that the fermented samples meaned all samples including must and krausen samples. During each stage, 9 mL must, or krausen samples were collected from corresponding spontaneous fermentation samples, placed into sterile 10 mL centrifuge tubes, and stored at −80°C for DNA extraction. In addition, the fermented jars were sealed with sterile plastic and sealing films to provide anaerobic fermentation conditions on day 3, and the fermented mash was transferred from the 500 mL jars to 250 mL sterilized triangular flasks to remove the skin residue on day 5. The fermentation process was monitored by measuring the °Brix every 2 days, and fermentation was considered completed when the °Brix displayed no change three consecutive times, the clarified fermented samples on day 20 was taken as the naturally fermented wine samples. Then, respective 50 mL must, and spontaneously fermented wine samples were stored at −20°C for physical and chemical index analysis. The fermentation process is shown in Supplementary Figure 1.

### DNA Extraction, Polymerase Chain Reaction (PCR) Amplification and ITS2 rDNA Sequencing

The genomic DNA of the fungal communities in the Marselan must and fermented samples were extracted using a modified CTAB method. The purity and concentration of the DNA were quantified with a nano spectrophotometer (Thermo Fisher Scientific, Wilmington, DE, United States) and 1% (w/v) agarose gel. The fungal ITS2 gene PCR was performed *via* the ITS3-2024F (5′-GCATCGATGAAGAACGCAGC −3′) and ITS4-2409R (5′-TCCTCCGCTTATTGATATGC −3′) primers. The primary PCR reactions contained 20 ng DNA template, 2× AmpliTaq TSINGKE^®^ MasterMix (TsingKe Biotech, Beijing, China), 10 pmol of each primer. A secondary PCR to index the amplicons was performed with TaKaRa Taq DNA Polymerase. Amplification was conducted as follows: 94°C for 4 min, followed by 34 cycles of 94°C for 1 min, 57°C for 45 s, 72°C for 1 min, and a final extension at 72°C for 10 min. All the samples were amplified in triplicate, and no-template controls were included in all steps of the process (Zhang et al., 2019b). The Qiagen Gel Extraction Kit and PCR Clean-up (Qiagen, Germany) were employed to refine the PCR products. Furthermore, fluorometric evaluation using the Qubit 2.0 dsDNA HS Assay Kit (Invitrogen, Carlsbad, CA, United States) obtained the purity of the PCR mixture, after which sequencing analysis continued *via* the IonS5TMXL sequencing platform (IonS5TMXL, Thermo Fisher Scientific, United States) from Novogene, Beijing, China.

### Measurement of Physicochemical Parameters

The pH and °Brix of the samples were measured with a HANNA 211 pH meter (HANNA, Padua, Italy) and a refractometer (ATAGO, Tokyo, Japan), respectively.

The glucose, fructose, ethanol, and glycerol content were determined with HPLC using a Waters 2414 RI Detector and a BIO-RAD Aminex HPX-87H resin-based column (300 mm × 7.8 mm) (Sun et al., 2015), and eluted with 5 mM H_2_SO_4_ at 55°C, 0.5 mL/min.

The acetic acid (AA), total acid (TA), polyphenol (PP), and anthocyanins (AC) content were analyzed with corresponding Randox kits on the Randox Monaco Analyzer (Randox, Monaco, United Kingdom).

### Data Analysis

Raw sequencing reads obtained from the IonS5TMXL platform were paired and pre-merged using FLASH software (Version 1.2.7), as well as filtered with the QIIME software (Version 1.7). All quality filtered sequencing reads were then clustered into operational taxonomic units (OTUs) with a minimum identity of 97%, by applying UPARSE software (Version 7.0) (Liu et al., 2018). Additionally, the OTU table underwent a series of filtering steps, including removing low-quality bases and chimeras, removing the OTUs with <3 counts across all samples, removing possible contaminants (mitochondrial and chloroplast sequences). The remaining OTUs samples were rarefied at a value equal to the median amount of all sequences (80160 sequences) to compensate for the uneven sequencing depth between the samples. For each fungal representative sequence (OTUs), taxonomy was assigned based on the UNITE fungal ITS database.

The Shannon index, Simpson index, abundance-based coverage estimator (ACE), chao1, and phylogenetic diversity (PD) whole tree values were calculated to compare the intra-group diversity (α-diversity). Moreover, Wilcoxon tests were used to evaluate the differences in average α-diversity indices between the groups (Blount et al., 2019). Furthermore, unconstrained principal coordinates analysis (PCoA) was conducted based on weighted UniFrac distance to evaluate the differences between the fungal communities of different regions and wineries, using the vegan package (version 2.5.3) in the R software (version 4.0.0). Based on the PCoA results, orthogonal partial least squares discriminant analysis (OPLS-DA) was performed to characterize the similarities and differences between the fungal microbiota of different regions using R software with the DiscriMiner package (version 6.3-73) (Shankar et al., 2013). Moreover, the multivariate analysis of variance (Adonis) and multi-response permutation procedure (MRPP) was conducted to identify the significant differences between the regions and wineries using the vegan R package (Cai et al., 2015).

Subsequently, the linear discriminant analysis (LDA) effect size (LEfSe) algorithm was performed to identify the representative fungal taxa of each region, by utilizing the Huttenhower Lab Galaxy Server (Liu et al., 2018). After the environmental factors (Gc and Pi) were screened *via* the variance inflation factor (VIF) and step selection using the vegan and dplyr (version 0.4.3) R package, detrended correspondence analysis (DCA) result determined to apply redundancy analysis (RDA) to explore the correlation between the environmental factors and fungal communities. Moreover, variation partitioning analysis (VPA) was performed to evaluate the contribution rate of each factor (Li et al., 2017). In addition, Spearman’s correlation analysis was applied to estimate the correlation between the environmental factors, the Pi variations of the spontaneously fermented wine (difference value with must), and the major fungal genus (Huang et al., 2019).

Other data were expressed as mean ± standard deviation, and the statistical significance between the groups was analyzed with one-way analysis of variance (ANOVA) using SPSS 19.0 software (SPSS Inc., Chicago, IL, United States).

## Results

### Fungal Composition of Marselan Must and Fermented Samples

**FIGURE 1:**
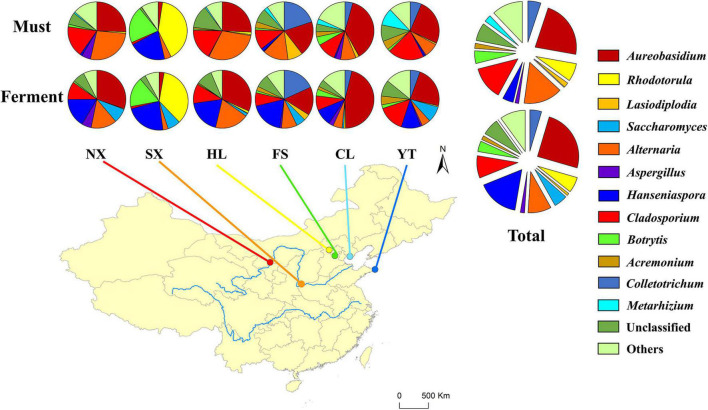
Relative abundance percentages (%) of the Marselan must and fermented sample fungal genera from different regions.

To characterize the fungal communities throughout the Chinese Marselan wine-producing regions, 180 triplicated must, and spontaneously fermented samples were collected from 15 wineries (or vineyard blocks) in six regions. It is worth noting that eight samples were removed according to the abnormal Shannon index and species annotation, while six samples were eliminated since they did not meet the sequencing requirements (Supplementary Table 3). The remaining valid sequences in 166 samples with average ITS2 reads of 80,055 were clustered into 1611 OTUs with 97% similarity (Supplementary Data Sheet 1). Compared with the fungal reference database, taxonomic assignment revealed at least three fungal phyla (Ascomycota, Basidiomycota, and Mucoromycota), 22 classes, 66 orders, 148 families, and 306 genera after removing the samples assigned to bacterial taxa, in addition to some unknown groups, indicating the relative extent of uncharacterized fungi. The Ascomycota dominated 87.68% of all OTUs, followed by Basidiomycota at 4.28% (Supplementary Data Sheet 2). Aureobasidium, Alternaria, and Cladosporium were the dominant fungal genera in the must samples, accounting for 24.40, 17.29, and 14.82%, respectively. The mycobiota of the must also contained Colletotrichum, Rhodotorula, Metarhizium, Botrytis, Papiliotrema, Hanseniaspora, and other trace fungi with a relative content exceeding 2%, while the unclassified genus accounted for 7.97% (Figure 1 and Supplementary Table 4).

Considering the fermentation process, *Aureobasidium*, *Hanseniaspora*, *Alternaria*, *Cladosporium*, and *Rhodotorula* comprised the primary fungal genera, accounting for 24.88, 16.21, 9.16, 8.78, and 6.14%, respectively. Notably, the proportion of *Saccharomyces* throughout the fermentation process increased to 5.32%, while the unclassified genus accounted for 6.57%. The Marselan fermentation mycobiota contained *Colletotrichum*, *Botrytis*, *Metarhizium*, *Aspergillus*, *Acremonium*, and *Papiliotrema* accounting for more than 1% (Figure 1 and Supplementary Table 4). The rarefaction curves and Good’s coverage data of the Marselan must and fermented samples indicated that the ITS2 gene library was generally well-constructed (Supplementary Figure 2 and Supplementary Table 5).

### α-Diversity Analysis of Marselan Fungal Communities

**FIGURE 2:**
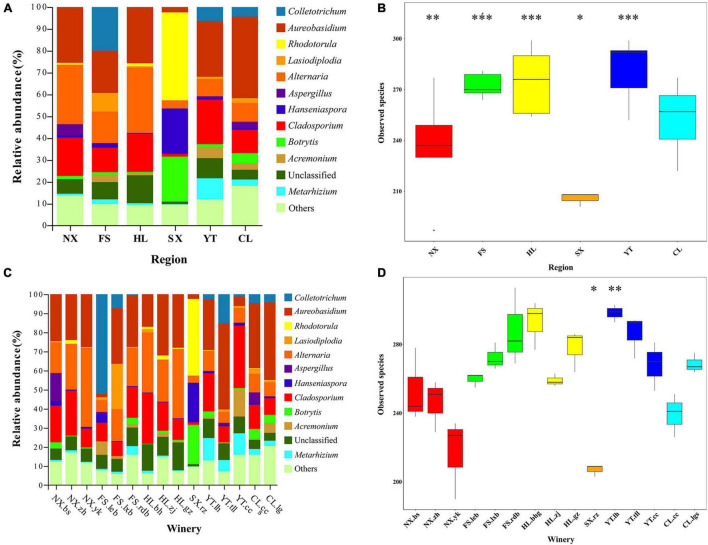
Marselan must fungal communities. **(A)** Relative abundance percentages (%) of the Marselan must fungal genera from different regions. **(B)** Wilcoxon test involving the Marselan must fungal species numbers of different regions. **(C)** Relative abundance percentages (%) of the Marselan must fungal genera of different wineries. **(D)** Wilcoxon test involving the Marselan must fungal species numbers of different wineries. The symbols *, **, and *** represent significant differences.

Moreover, 283 fungal species were detected in the Marselan must samples of YT, which was the highest of all six regions, while SX displayed the least number at only 208 fungal species. The number of observed species in descending order was as follows: YT, HL, FS, CL, NX, and SX (Figure 2B). Although the variations in ACE, Chao1, the Shannon indices, and the Simpson index were generally consistent with the species number trends, FS, the third-highest on the list of fungal species, exhibited the lowest Simpson index value (Supplementary Table 5A). At the winery level, Chateau Rongzi (SX.rz) in SX presented the lowest number of fungal species, while the Winery Baihuagu (HL.bhg) in HL had the highest (Figure 2D and Supplementary Table 6A).

During the fermentation process, the number of fungal species in the six Marselan regions decreased slightly, but YT still had the highest number of species and NX the lowest, while CL, ranking fourth in observed species, demonstrated the minimum Simpson index value (Supplementary Figure 3B and Supplementary Table 5B). At winery level, Winery Yunkou (NX.yk) in NX displayed the least number of species at 208, while HL.bhg in HL exhibited the highest at 299 (Supplementary Figure 3D and Supplementary Table 6B).

### The Biogeographic Distribution Pattern of Fungal Communities in the Main Chinese Marselan Wine-Producing Regions

The relative abundance diagram of the fungal communities in different regions indicated that whether it was must or fermented samples, the fungal microbiota composition of NX and HL regions were relatively similar, while YT and CL were relatively similar. And the FS and SX regions were obviously different from other regions, while FS contained a higher proportion of *Colletotrichum*, SX contains a higher proportion of *Rhodotorula* (Figure 2A and Supplementary Figure 3A). Furthermore, except for individual wineries in FS (FS.rdb) and YT (YT.tll) regions, the fungal structure among different wineries in the same regions were relatively similar for both must and fermented samples (Figure 2C and Supplementary Figure 3C).

**FIGURE 3:**
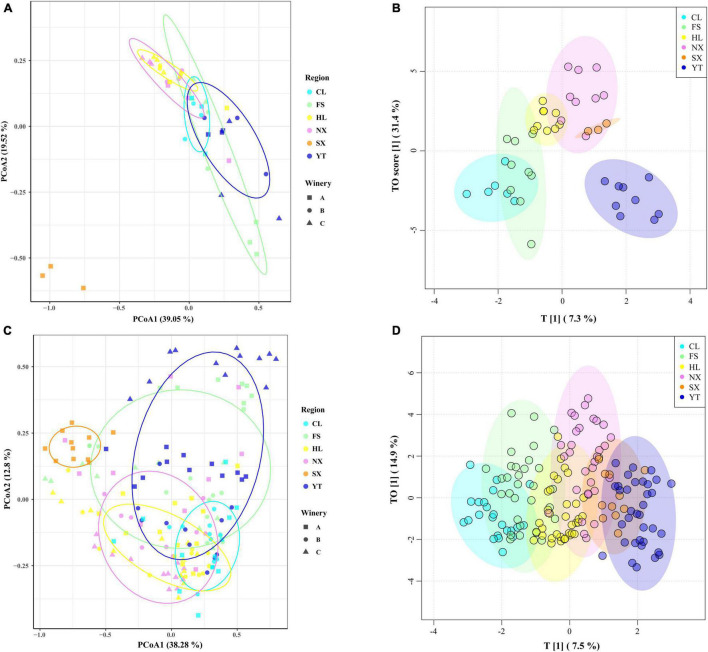
Coordinate and cluster analysis of the Marselan must fungal communities of different regions and wineries. **(A)** Weighted UniFrac PCoA plot of the Marselan must fungal communities. **(B)** OPLS-DA plot of the Marselan must fungal communities. **(C)** Weighted UniFrac PCoA plot of the Marselan fermented sample fungal communities. **(D)** OPLS-DA plot of the Marselan fermented sample fungal communities. Different colors correspond to different regions, and different shapes correspond to different wineries within a region.

As a visual and intuitive display, the PCoA method was conducted to explore the biogeographic distribution patterns of the fungal communities in different regions and wineries. The first two principal coordinates (PC) axis for the must account for 58.57% of the total fungal variance. Although extended distances were evident between the wineries within the FS and YT regions, the fungal communities displayed distinct clustering at the region level. SX was noticeably different from other regions, located at the left bottom of the third phenomenon, indicating the remarkable impact of the high *Rhodotorula* abundance. While the fungal compositions of NX and HL regions were similar displaying closer distances, mainly locating at the first phenomenon, the sample points of FS, YT, and CL regions spanned the second and third quadrants, and some sample points of YT and CL regions displayed a partial overlap (Figure 3A). Although not as clear as the boundaries between different regions in must, the fungal microbiota of the fermented samples also revealed obvious clustering phenomenon at the region level, except for the FS and YT regions (Figure 3C).

Through PCoA analysis, we could preliminarily confirm the regional microbial clustering landscape of fungal communities in the main Chinese Marselan wine-producing regions, supervised OPLS-DA analysis was further applied to verify the regional distribution pattern. The OPLS-DA plots display that the fungal microbiota exhibit the clustering phenomenon at the region level, and the differences among regions are more distinct. For must fungal communities, HL, NX and SX regions are closer, manifesting their relatively approximate fungal structure. FS and CL regions are relatively close, indicating that their fungal composition is relatively similar. And YT is located in the third quadrant alone, indicating its unique fungal structure (Figure 3B). The OPLS-DA plot of the fermented samples displayed the same regional distribution characteristics, but there were some overlapping sample points between different regions, revealing that the diversity of fungal microbiota between different regions decreased (Figure 3D).

Although the regional distribution pattern of fungal communities in the main Chinese Marselan wine-producing regions has been confirmed by the ordination analysis, the similarities and differences varied from different regions and wineries. Consequently, Adonis and MRPP were applied for difference quantitation to further assess the fungal community homeomorphism at different geographic scales. The Adonis and MRPP tests based on weighted UniFrac indicated that substantially significant differences (*P* = 0.001) were observed between the fungal compositions of the must from different regions (*R*^2^_Adonis_ = 0.560, A_MRPP_ = 0.296) and wineries (*R*^2^_Adonis_ = 0.830, A_MRPP_ = 0.518). During the fermentation process, extremely significant discrepancy were still evident in the fungal consortium of both different regions and wineries. However, the differences exhibited a decline (Region: *R*^2^_Adonis_ = 0.308, A_MRPP_ = 0.183, Winery: *R*^2^_Adonis_ = 0.478, A_MRPP_ = 0.287) (Table 1). Differences among the wineries in each region were also investigated, indicating significant discrepancy between the fungal composition of wineries within each region except CL. The Adonis of the wineries in CL displayed no significant differences based on weighted UniFrac distance (*P* = 0.4). During the development of fermentation, the differences between the fungal communities of the wineries in the different regions also decreased (Supplementary Table 7).

**TABLE 1:** Adonis and MRPP tests of the Marselan must and fermented sample fungal communities.

		Weighted UniFrac			
		Adonis		MRPP	
	Group	*R* ^2^	*P*	A	*P*
Must	Region	0.560	0.001	0.296	0.001
	Winery	0.830	0.001	0.518	0.001
Ferment	Region	0.308	0.001	0.183	0.001
	Winery	0.478	0.001	0.287	0.001

*R^2^_Adonis_ and A_MRPP_ are variance contribution factors. Significant (P < 0.05); extremely significant (P < 0.01).*

### The Fungal Biomarkers of the Different Marselan Regions

**FIGURE 4:**
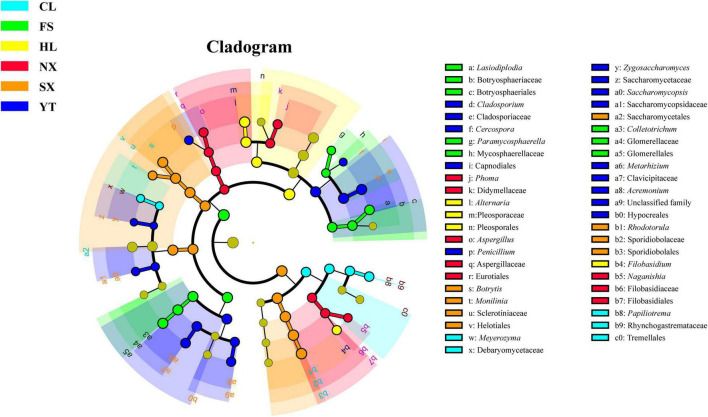
LEfSe results reporting the biomarkers of different regions in the Marselan must fungal communities.

As we have confirmed the regional distribution pattern of fungal communities in the Chinese main Marselan wine-producing regions, LEfSe algorithm with a 4.0 LDA score threshold was applied to identify the discriminative fungal taxa in the Marselan must and fermented samples from the different regions. Among the fungal communities of Marselan must, 47 fungal species were verified as being differentially abundant in six regions. Of these, Filobasidiaceae (family), *Phoma* (genus), and *Aspergillus* (genus) were significantly enriched in NX; Mycosphaerellaceae (family), *Colletotrichum* (genus), and *Lasiodiplodia* (genus) were prominently abundant in FS; genus *Alternaria* and *Filobasidium* were significantly enriched in HL; genus *Rhodotorula*, *Botrytis*, and *Monilinia* were prominently abundant in SX; genus *Cladosporium*, *Metarhizium*, and *Acremonium* were significantly enriched in YT; genus *Meyerozyma* and *Papiliotrema* were prominently abundant in CL (Figure 4).

Considering the Marselan fermented samples, 36 fungal taxa were verified as being differentially abundant in the six regions, which were slightly less than the Marselan must numbers. Among them, genus *Aspergillus* was the only strain exhibiting a significantly higher abundance in NX; genus *Colletotrichum* and *Lasiodiplodia* were significantly enriched in FS; genus *Alternaria* and *Filobasidium* were prominently abundant in HL; genus *Rhodotorula*, *Botrytis*, and *Monilinia* were significantly enriched in SX; genus *Cladosporium*, *Metarhizium*, and *Acremonium* were prominently abundant in YT; genus *Aureobasidium*, *Papiliotrema*, and *Phoma* were significantly enriched in CL (Supplementary Figure 4). These results indicated that of all the sampling regions used in this study, the Marselan grape regions could be effectively distinguished by the demonstrative microorganisms at different biological classification levels, from order to genus.

### Correlation Analysis Between Marselan Must Fungal Communities and Environmental Factors

**FIGURE 5:**
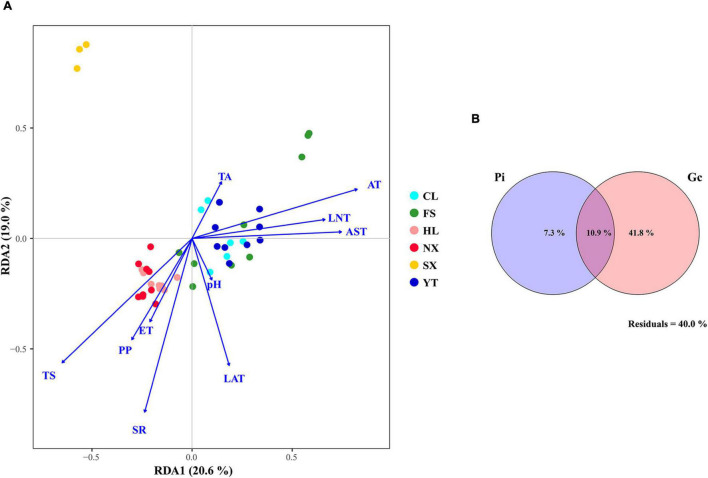
**(A)** RDA and **(B)** VPA of the environmental factors related to the Marselan must fungal communities. For the VPA, the variables presented in the RDA were separated into two groups: Gc (SR, AT, AST, LAT, LNT, and ET), and Pi (TS, PP, TA, and pH).

Redundancy analysis was applied to explore the relationship between the Marselan must fungal communities, and the environmental factors (Gc and Pi). The geoclimatic data of 2017 for the six Marselan regions were supplied by the Wuhan Donghu Big Data Trading Center Co., Ltd. (Table 2)^1^. After removing all the autocorrelation variables, the RDA plot indicated that ten of the 17 tested indexes significantly correlated with the microbial composition. These included six Gc, namely solar radiation (SR), average temperature (AT), average soil temperature (AST), LAT, LNT, and evaporation capacity (ET), as well as four Pi, namely total sugar (TS), PP, TA, and pH. While these environmental factors accounted for 58.17% of the taxonomic information, the first two axis explained 39.60% of the fungal variation (Figure 5A). Further VPA indicated that the Gc was principally responsible for shaping the fungal communities of the Marselan must, accounting for 36.20% of the fungal variation, while the Pi explained 10.20% of the total variability. Furthermore, 41.80% of the community distribution was not constrained by these two groups of determining factors (Figure 5B).

**TABLE 2:** Climatic and geographic data of the six Marselan regions in 2017 vintage.

Region	Meteorological station	Station number	Latitude	Longitude	Average elevation (0.1 m)	Average temperature (0.1°C)	Rainfall (0.1 mm)	Net wind speed (0.1 m/s)
NX	Yinchuan City, Ningxia Province	53614	N38°28	E106°12	11109	110.205	4226	15.991
FS	Fangshan District, Beijing	54596	N39°46	E116°12	489	133.106	13408	21.265
HL	Huailai County, Hebei Province	54405	N40°25	E115°30	5709	107.619	9498	24.41
SX	Ji County, Shanxi Province	53859	N36°06	E110°40	8513	112.695	12858	19.673
YT	Fushan District, Yantai City, Shandong Province	54764	N37°29	E121°14	539	135.435	12448	29.92
CL	Laoting County, Hebei Province	54539	N39°26	E118°53	85	132.58	8962	21.989

**Region**	**Relative humidity (1%)**	**Average pressure (0.1 hPa)**	**Average soil temperature (0.1°C)**	**Solar radiation (0.1 h)**	**Evapotranspiration (0.1 mm)**		**High-temperature weather (days)**	**Low-temperature weather (days)**

NX	47.61	8910.794	141.849	28945	13155		8	129
FS	52.832	10111.271	150.504	25897	13767		14	119
HL	48.671	9502.753	133.498	31245	14624		6	137
SX	61.268	9197.898	133.09	21821	11799		10	129
YT	62.419	10107.712	165.479	25586	11680		10	99
CL	61.679	10160.704	151.213	26842	11017		5	110

**FIGURE 6:**
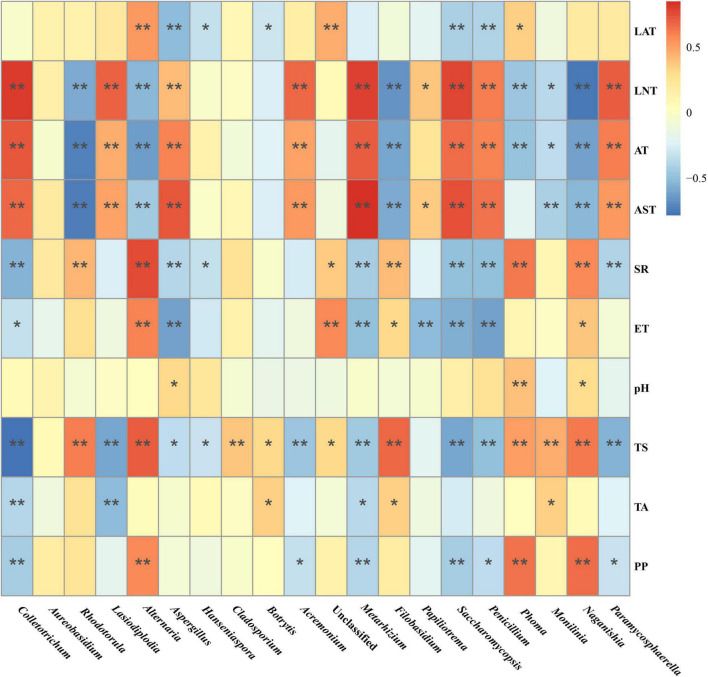
Spearman correlation analysis of the correlation between the major fungi abundance in the Marselan must and various environmental factors. The different color intensities represent the degree of correlation. “*”, significant (*P* < 0.05); “**”, extremely significant (*P* < 0.01). Factors abbreviations: latitude (LAT), longitude (LNT), altitude (AE), average temperature (AT), average soil temperature (AST), solar radiation (SR), evaporation capacity (ET), pH, total sugar (TS), total acid (TA), and polyphenol (PP).

Then, Spearman correlation analysis was used to further investigate the correlation between the variations in the major fungal genus and environmental factors. As such, the relative abundance of genus *Saccharomycopsis*, *Colletotrichum*, *Acremonium*, *Metarhizium*, *Lasiodiplodia*, and *Penicillium* were positively correlated with LNT, AT, and AST, and negatively with TS and PP. Moreover, the relative abundance of genus *Rhodotorula*, *Alternaria*, *Filobasidium*, and *Naganishia* displayed a significant positive correlation with SR, ET, and TS, and a negative correlation with LNT, AT, and AST, while various some other fungal genus were affected by the different Gc and Pi (Figure 6).

### The Correlation Analysis Between Fermented Sample Fungal Communities and the Physicochemical Parameters of Spontaneously Fermented Marselan Wine

Although Marselan must generated more than 8% v/v alcohol *via* spontaneous fermentation (except for FS.leb, FS.rdb, HL.gz, and CL.cc), most of the wineries did not meet the residual sugar standard for dry wine of below 4 g/L, including NX.zh and NX.yk in NX, FS.leb, FS.lxb, and FS.rdb in FS, HL.bhg and HL.gz in HL, as well as CL.cc and CL.lgs in CL. Furthermore, the AA content in most of the wineries exceeded the legal standard of 1.2 g/L, included NX.bs, NX.zh, and NX.yk in NX, FS.leb and FS.rdb in FS, HL.bhg in HL, as well as CL.cc and CL.lgs in CL (Table 3, OIV, 2013).

**TABLE 3:** The physicochemical parameters of spontaneously fermented Marselan wine.

Winery	Residual sugars (g/L)	Ethanol (% v/v)	Acetic acid (g/L)	Total acid (g/L)	Glycerol (g/L)	pH
NX.bs	3.11 ± 1.17^b^	14.6 ± 0.83^a^	1.68 ± 1.33^c^	5.93 ± 0.83^b^	12.26 ± 0.88^a^	3.05 ± 0.1^c^
NX.zh	6.94 ± 4.53^b^	11.46 ± 3.13^ab^	1.2 ± 1.05^c^	5.43 ± 2.15^b^	9.5 ± 1.73^bc^	3.15 ± 0.04^c^
NX.yk	44.75 ± 28.66^ab^	8.2 ± 5.45^bcd^	5.7 ± 5.6^bc^	10.96 ± 3.89^a^	7.86 ± 2.75^d^	3.64 ± 0.29^a^
FS.leb	72.37 ± 23.59^a^	3.76 ± 1.53^de^	7.24 ± 3.24^b^	11.4 ± 3.24^a^	2.11 ± 0.66^e^	3.13 ± 0.07^c^
FS.lxb	44.48 ± 19.28^ab^	8.4 ± 0.54^bcd^	0.16 ± 0.17^c^	5.23 ± 0.83^b^	7.52 ± 0.54^d^	3.57 ± 0.06^ab^
FS.rdb	46.14 ± 5.89^ab^	2.55 ± 1.8^e^	1.59 ± 1.02^c^	5.43 ± 0.85^b^	3.3 ± 1.41^e^	3.45 ± 0.04^bc^
HL.zj	3.31 ± 0.07^b^	12.48 ± 0.2^ab^	1.25 ± 0.26^c^	4.83 ± 0.66^b^	10.85 ± 0.81^abc^	3.53 ± 0.06^ab^
HL.bhg	33.7 ± 22.73^ab^	10.29 ± 1.77^ab^	1.1 ± 0.14^c^	5.73 ± 0.5^b^	6.89 ± 2.85^d^	3.74 ± 0.25^a^
HL.gz	68.48 ± 29.52^a^	7.07 ± 3.51^cd^	1.24 ± 0.23^c^	5.3 ± 0.6^b^	6.66 ± 1.62^d^	3.77 ± 0.11^a^
SX.rz	0.34 ± 0.95^b^	12.86 ± 0.54^ab^	1.07 ± 0.23^c^	6 ± 0.55^b^	11.41 ± 0.65^ab^	3.31 ± 0.15^bc^
YT.lh	0.67 ± 0.34^b^	11.52 ± 0.37^ab^	0.91 ± 0.46^c^	5.6 ± 0.5^b^	9.04 ± 0.08^bc^	3.31 ± 0.03^bc^
YT.tll	1.03 ± 0.26^b^	10.66 ± 0.18^ab^	1.04 ± 0.23^c^	4.66 ± 0.4^b^	8.48 ± 1.35^bcd^	3.12 ± 0.27^bc^
YT.cc	4.87 ± 7.04^b^	10.28 ± 0.6^ab^	1.31 ± 1.75^c^	7.03 ± 2.18^b^	8.12 ± 0.72^bcd^	3.73 ± 0.05^a^
CL.cc	52.97 ± 11.47^a^	2.66 ± 0.94^e^	12.8 ± 5.73^a^	13.76 ± 2.2^a^	3.5 ± 0.73^e^	3.19 ± 0.09^c^
CL.lgs	13.17 ± 10.95^b^	10.76 ± 1.76^ab^	3.69 ± 3.87^bc^	9.1 ± 4.25^a^	8.23 ± 1.64^bcd^	3.23 ± 0.02^bc^

*Values are given as mean ± standard deviation of three biological replicates. Letters indicate the level of significant difference (P < 0.05) as determined with ANOVA analysis.*

**FIGURE 7:**
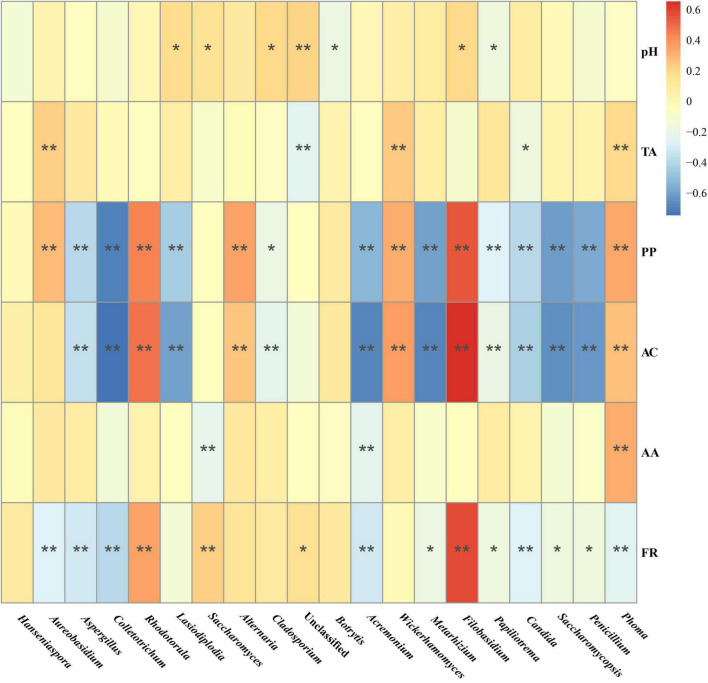
Spearman correlation analysis of the correlation between the fungal composition in the Marselan fermented samples and physicochemical index variations of the spontaneous fermented wine. The different color intensities represent the degree of correlation. “*”, significant (*P* < 0.05); “**”, extremely significant (*P* < 0.01). Factors abbreviations: total acid (TA), polyphenol (PP), anthocyanins (AC), acetic acid (AA), and fermentation rate (FR).

Spearman correlation analysis was performed to investigate the correlation between the fungal composition of the Marselan fermented samples and the Pi variations of the spontaneously fermented wine (difference value with must), the results indicated that AC and PP content correlated positively with genus *Filobasidium* and *Rhodotorula*, and negatively with genus *Colletotrichum* and *Acremonium*. Notably, the fermentation rate (FR) was confirmed to correlate positively with genus *Filobasidium* and *Rhodotorula*, and negatively with genus *Paramycosphaerella*, *Colletotrichum*, and *Acremonium*, which was consistent with AC and PP content (Figure 7).

## Discussion

The wine microbiome has been substantially studied due to its association with wine quality and style, especially after the concept of wine “microbial terroir” was proposed (Gilbert et al., 2014; Bokulich et al., 2016). Compared with the widely cultivated varieties, Cabernet Sauvignon and Chardonnay, the Chinese promising Marselan variety has a relatively short vine age due to the late introduction, a fundamental study to ascertain whether a regional microbial model has been formed nationwide is highly indispensable (Ma, 2017; Lyu et al., 2019). Consequently, based on the systematic evaluation of fungal communities in the six wine-producing regions, we firstly explored the biogeographic distribution patterns of the Marselan fungal microbiota between different regions. The correlation between fungal microbiota and environmental factors such as the Gc and Pi was also examined, further elaborating the influence of geographical location on fungal microbiota. This study also explored the relationship between the fungal communities and the Pi of spontaneously fermented wine, evaluating the possibility of producing wine through spontaneous fermentation to promote regional characteristics.

Besides the frequently encountered genera *Aureobasidium*, *Aspergillus*, *Alternaria*, *Cladosporium*, *Botrytis*, *Hanseniaspora*, *Rhodotorula*, and *Filobasidiella*, which have been simultaneously detected in California, United States (Bokulich et al., 2014) and Spain (Wang et al., 2015), this study has discovered some pathogenic grape strains and biocontrol fungi, such as *Colletotrichum*, *Lasiodiplodia*, *Metarhizium*, and *Filobasidium*, although their proportion is not high, considering their impact on grape fruit quality (Kirchmair et al., 2004; Combrinck et al., 2011), further research is needed to determine their origin and their impact on wine quality, especially when the effect of some strains on wine aroma has been proved (Ma et al., 2021). Meanwhile, corresponds with other studies (Pinto et al., 2015; Morrison-Whittle and Goddard, 2018), this study also detected a considerable proportion of unknown fungi, more researches are requisite to characterize them in order to consummate the microbial wine repository.

Studies have found that the wine-related microbial α-diversity diversity varied with regional origins and vineyard management (Burns et al., 2016; Grangeteau et al., 2017; Liu et al., 2020). The present study supports this, and the order of α-diversity between regions during the fermentation process was basically the same as that of must. While higher fungal diversity may generate richer wine aroma through more abundant metabolic activities, the risk of fermentation stagnation also increases (Capozzi et al., 2015; Garofalo et al., 2015). Fortunately, the alcohol, high osmotic pressure, and high acid condition during the fermentation process reportedly reshape the fungal microbiota (Liu et al., 2017), this is usually conducive to the growth of yeast and the steady fermentation process (Wang et al., 2015).

By systematically analyzing the similarities and differences of the fungal microbiota between regions and wineries, this study determined the regional distribution pattern of fungal communities in the Chinese main Marselan wine-producing regions. This result corresponds with research involving Chardonnay in United States (Bokulich et al., 2014) and Sauvignon Blanc in New Zealand (Morrison-Whittle and Goddard, 2018). The Marselan regional pattern also broadly conforms to the conclusion that the geographical origin is more effective than the variety in impacting the fungal communities (Kioroglou et al., 2019). Furthermore, this study discovered that the differences among the Marselan fungal communities decreased during fermentation, which corresponds with reports that the Cabernet microbial community profiles became less distinct as the microbial signatures among the AVAs and vineyards diminished during fermentation (Bokulich et al., 2016).

However, significant differences in the fungal microbiota of wineries were also detected, even stronger than the difference between regions. Various factors may alter the mycobiota intra-region, such as microclimate, soil conditions, or agricultural management (Vitulo et al., 2018), thus forming the unique mycobiota between different wineries. Furthermore, the similarities in the fungal communities among different wineries in the same regions also reduced the variations in disparate regions. The topography of the sampling wineries in FS and YT is quite different, as the FS.rdb winery is relatively far away from the other two wineries in FS and located on a slope, while the FS.leb and FS.lxb wineries are located on flat ground, and the winery YT.lh is located on a slope, while the wineries YT.tll and YT.cc are located on flat ground. These discrepancies may further alter the fungal compositions of different wineries within the same region, leading to a more substantial difference between the fungal compositions of the wineries in these two regions (Supplementary Table 7). Therefore, further studies are necessary to determine the influencing factors of fungal communities on blocks with different microclimatic, viticultural, and topographical conditions beyond the scope of this study.

Though the demonstrative fungi distinguishing different regions are usually the genera with relatively low abundances, such as *Penicillium*, *Colletotrichum*, *Botrytis* (Kioroglou et al., 2019), the regional biomarkers detected in this study include both dominant genus *Alternaria*, *Aureobasidium*, *Rhodotorula*, and *Cladosporium*, and low abundance genus *Aspergillus*, *Papiliotrema*, and *Phoma*. These fungal biomarkers contained representative phytopathogenic *Colletotrichum*, *Botrytis*, and *Aspergillus*, weakly fermentative yeast *Hanseniaspora* and *Candida*, fermentative yeast *Torulaspora*, as well as *Filobasidium* and *Aureobasidium* whose functions have not been characterized (Barata et al., 2012). Although the function of some fungal biomarkers is unclear, information on fungal microflora discrepancies between regions may guide us to formulate targeted vineyard and fermentation management practices to produce high-quality wines.

Recent investigations have characterized many factors affecting the must and fermentation microbiome, including locally managed (Morgan et al., 2019) and native ecosystems (Kioroglou et al., 2019). The results indicated that SR, AT, AST, LAT, LNT, and ET exerted a significant effect on fungal communities, revealing approximately the same geographic and climatic factors regulated fungal microbiota (Bokulich et al., 2014; Gao et al., 2019). Furthermore, we found that Pi, such as TS, PP, TA, and pH, also modified the fermentation microflora. Although the physicochemical elements of must are closely related to geoclimatic conditions, this correlation somehow guides harvest time selection and fermentation microorganism management. And it is essential to underline the effect of PP on the must mycobiota. Since PP is not only an indispensable constituent of red wine (Soares et al., 2017), but is also a functional component beneficial to health (Greyling et al., 2016), as well as a broad-spectrum antibacterial agent (Kontić et al., 2015), further research is indispensable to clarify the interaction between PP and fermentation microorganisms. As far as is known, no previous reports exist regarding the relationship between PP and fermentation fungal communities. In addition, the factors involved in the study only explained 58.17% of the taxonomic information, with 41.83% unknown influence to be resolved by further research.

Spontaneous fermentation is an effective measure to maximize the role of autochthonous “microbial terroir” (De Filippis et al., 2017). Although most of the wineries in this study are not currently suitable for spontaneous fermentation, the strains associated with fermentation indicators and their influencing factors discovered by this study may provide new approaches for winemaking management, as increasing evidence supporting the role of “microbial terroir” in shaping regional wine phenotypes (Bokulich et al., 2016; Belda et al., 2017).

## Conclusion

“Microbial terroir” is an vital shaping factor of wine terroir, through the elaborated study of the fungal composition in the six representative wine-producing regions, and on the basis of characterizing the similarities and differences of fungal microbiota at the two different geographic levels of region and winery, this study firstly proposed the regional distribution pattern of fungal communities in the Chinese main Marselan wine-producing regions, indicating that the emerging cultivar has already formed a national-level “microbial terroir.” While the fungal biomarkers between different regions further confirmed the validity of the regional distribution patterns, correlation analysis established the primary role of the geoclimatic factors in driving the fungal geographical distribution, and the correlation between the microbiome and spontaneous fermentation performance. In addition to delineating a fundamental landscape of the fungal biogeographical patterns across different Marselan wine-producing regions, these findings provided a new benchmark for harnessing the “microbial terroir” to enhance regional wine expression.

## Data Availability Statement

The datasets presented in this study can be found in online repositories. The names of the repository/repositories and accession number(s) can be found below: https://www.ncbi.nlm.nih.gov/genbank/, PRJNA640504.

## Author Contributions

RL, JZ, and WH: conceptualization. RL, SY, and ML: data curation. RL: formal analysis and writing – original draft. WH: funding acquisition. RL, SY, SG, ML, and XH: investigation. SY, LD, and YS: resources. JZ and WH: supervision. XH, MR, and YY: writing – review and editing. All authors have read and agreed to the published version of the manuscript.

## Conflict of Interest

LD was employed by the company Wuhan Donghu Big Data Trading Center Co., Ltd. The remaining authors declare that the research was conducted in the absence of any commercial or financial relationships that could be construed as a potential conflict of interest.

## Publisher’s Note

All claims expressed in this article are solely those of the authors and do not necessarily represent those of their affiliated organizations, or those of the publisher, the editors and the reviewers. Any product that may be evaluated in this article, or claim that may be made by its manufacturer, is not guaranteed or endorsed by the publisher.

## Footnotes

1
http://www.chinadatatrading.com

## Supplementary Material

Supplementary Data Sheet 1 OTUs detected in the 166 Marselan must and fermented samples.

Supplementary Data Sheet 2 Taxonomic assignment and species relative proportion of the 166 Marselan must and fermented samples.
