# Supplementary material for: The Biogeography of Fungal Communities Across Different Chinese Wine-Producing Regions Associated With Environmental Factors and Spontaneous Fermentation Performance
Source: Front Microbiol. 2022 Feb 25;12:636639. doi: 10.3389/fmicb.2021.636639 (PMC8914289; doi:10.3389/fmicb.2021.636639)
Supplement: Supplementary Data Sheet 1 — OTUs detected in the 166 Marselan must and fermented samples. [file Data_Sheet_1.DOCX]

>OTU_1

GAAATGCGATAAGTAATGTGAATTGCAGAATTCAGTGAATCATCGAATCTTTGAACGCACATTGCGCCCCTTGGTATTCC

GAGGGGCATGCCTGTTCGAGCGTCATTACACCACTCAAGCTATGCTTGGTATTGGGCGTCGTCCTTAGTTGGGCgcgcCT

TAAAGACCTCGGCGAGGCCACTCCGGCTTTAGGCGTAGTAGAATTTATTCGAACGTCTGTCAAAGGAGAGGAACTCTGCC

GACTGAAACCTTTATTtttCTAGGTTGACCTCGGATCAGGTAGGGATACCCGCTGAACTTAA

>OTU_2

GAATTGCGATAAGTAATGTGAATTGCAGATACTCGTGAATCATTGAATTtttGAACGCACATTGCGCCCTTGAGCATTCT

CAGGGGCATGCCTGTTTGAGCGTCATTTCCTTCTCAAAAGATAATTTATTATTttttGGTTGTGGGCGATACTCAGGGTT

AGCTTGAAATTGGAGACTGTTTCAGTCTTttttAATTCAACACTTAGCTTCTTTGGAGACGCTGTTCTCGCTGTGATGTA

TTTATGGATTTATTCGTTTTACTTTACAAGGGAAATGGTAACGTACCTTAGGCAAAGGGTTGCTTTTAATATTCATCAAG

TTTGACCTCAAATCAGGTAGGATTACCCGCTGAACTTAA

>OTU_3

GAAATGCGATAAGTAGTGTGAATTGCAGAATTCAGTGAATCATCGAATCTTTGAACGCACATTGCGCCCTTTGGTATTCC

AAAGGGCATGCCTGTTCGAGCGTCATTTGTACCCTCAAGCTTTGCTTGGTGTTGGGCGTCTTGTCTCTAGCTTTGCTGGA

GACTCGCCTTAAAGTAATTGGCAGCCGGCCTACTGGTTTCGGAGCGCAGCACAAGTCGCACTctctATCAGCAAAGGTCT

AGCATCCATTAAGCCTTtttttCAACTTTTGACCTCGGATCAGGTAGGGATACCCGCTGAACTTAA

>OTU_4

GAAATGCGATAAGTAATGTGAATTGCAGAATTCAGTGAATCATCGAATCTTTGAACGCACATTGCGCCCGCCAGCATTCT

GGCGGGCATGCCTGTTCGAGCGTCATTTCAACCCTCAAGCTCCGCTTGGTGTTGGGGCCCTACAGCTGATGTAGGCCCTC

AAAGGTAGTGGCGGACCCTCCTGGAGCCTCCTTTGCGTAGTAACTTTACGTCTCGCACTGGGATCCGGAGGGACTCTTGC

CGTAAAACCccccAATTTTCCAAAGGTTGACCTCGGATCAGGTAGGAATACCCGCTGAACTTAA

>OTU_5

GAAATGCGATAAGTAATGTGAATTGCAGAATTCAGTGAATCATCGAATCTTTGAACGCACATTGCGCCcccTGGTATTCC

GGggggCATGCCTGTTCGAGCGTCATTTCACCACTCAAGCCTCGCTTGGTATTGGGCAACGCGGTCCGCCGCGTGCCTCA

AATCGACCGGCTGGGTCTTCTGTCCCCTAAGCGTTGTGGAAACTATTCGCTAAAGGGTGTTCGGGAGGCTACGCCGTAAA

ACAACCCCATTTCTAAGGTTGACCTCGGATCAGGTAGGGATACCCGCTGAACTTAA

>OTU_6

GAAATGCGATAAGTAATGTGAATTGCAGAATTCAGTGAATCATCGAATCTTTGAACGCACATTGCGCCCCTTGGTATTCC

GGggggCATGCCTGTTCGAGCGTCATTTCAACCCTCAAGCTTAGCTTGGTATTGAGTCTATGTCAGTAATGGCAGGCTCT

AAAATCAGTGGCGGCGCCGCTGGGTCCTGAACGTAGTAATATCtctcGTTACAGGTTCTCGGTGTGCTTCTGCCAAAACC

CAAATTtttCTATGGTTGACCTCGGATCAGGTAGGGATACCCGCTGAACTTAA

>OTU_7

GAAATGCGATACGTAATGTGAATTGCAGAATTCAGTGAATCATCGAATCTTTGAACGCACCTTGCGCTCCTTGGTATTCC

GAGGAGCATGCCTGTTTGAGTGTCATGAAATCTTCAACCCACCTCTTTCTTAGTGAATCTGGTGGTGCTTGGTTTCTGAG

CGCTGCTCTGCTTCGGCTTAGCTCGTTCGTAATGCATTAGCATCCGCAACCGAACTTCGGATTGACTTGGCGTAATAGAC

TATTCGCTGAGGATTCTAGTTTACTAGAGCCGAGTTGGGTTAAAGGAAGCTCCTAATCCTAAAGTCTATTttttGATTAG

ATCTCAAATCAGGTAGGACTACCCGCTGAACTTAA

>OTU_8

AAAATGCGATAAGTAATGTGAATTGCAGAATTCAGTGAATCATCGAATCTTTGAACGCACATTGCGCCCGCCAGTATTCT

GGCGGGCATGCCTGTCTGAGCGTCATTTCAACCCTCAGGACCCGTTCGCGGGACCTGGCGTTGGGGATCAGCCTGCCCCT

GGCGGCGGCTGGCCCTGAAATCCAGTGGCGGTTCCCTCGCGAACTCCTCCGTGCAGTAATTAAACCTCTCGCGGCAGGAT

AGCGGTTGAACCACGCCGTTAAACCccccACTTCTCAAGGTTGACCTCAGATCAGGTAGGAATACCCGCTGAACTTAA

>OTU_9

GAAATGCGATACGTAATGTGAATTGCAGAATTCCGTGAATCATCGAATCTTTGAACGCACATTGCGCCCCTTGGTATTCC

AGGgggCATGCCTGTTTGAGCGTCATTTCCTTCTCAAACATTCTGTTTGGTAGTGAGTGATACTCTTTGGAGTTAACTTG

AAATTGCTGGCCTTTTCATTGGATGTTttttttCCAAAGagagGTTTCTCTGCGTGCTTGAGGTATAATGCAAGTACGGT

CGTTTTAGGTTTTACCAACTGCGGCTAATCTTtttttATACTGAGCGTATTGGAACGTTATCGATAAGAAGagagCGTCT

AGGCGAACAATGTTCTTAAAGTTTGACCTCAAATCAGGTAGGAGTACCCGCTGAACTTAA

>OTU_10

GAAATGCGATAAGTAATGTGAATTGCAGAATTCAGTGAATCATCGAATCTTTGAACGCACATTGCGCCCTTTGGTATTCC

GAAGGGCATGCCTGTTCGAGCGTCATTACAACCCTCAAGCTCTGCTTGGTATTGGGCACCGTCCTTTGCGGGCgcgcCTC

AAAGACCTCGGCGGTGGCGTCTTGCCTCAAGCGTAGTAGAACATACATCTCGCTTCGGAGCGCAGGGCGTCGCCCGCCGG

ACGAACCTTCTGAACTTTTCTCAAGGTTGACCTCGGATCAGGTAGGGATACCCGCTGAACTTAA

>OTU_11

GAATTGCGATAAGTAATGTGAATTGCAGATTCTCGTGAATCATTGAATTtttGAACGCACATTGCGCCCTCTGGTATTCC

AGAGGGCATGCCTGTTTGAGCGTCATTTCCTTCTCAAaaaCCCAGTTtttGGTTGTGAGTGATACTCTGTTACAGGGTTA

ACTTGAAAATGCTATGCCCATTTGGCTGCCCCTTCTCTGAGGGGACTGCGCGTCTGTGCAGGATGTAACCAATGTATTTA

GGTATTCATACCAACTTTCATTGTGCGCGTCTTATGCAGTTGCAGTCCACCCAACCTCGGACacacTGGGCTGGCTGGGC

CAACAGTATTCATAAAGTTTGACCTCAAATCAGGTAGGAGTACCCGCTGAACTTAA

>OTU_12

GAATTGCGATAAGTAATATGAATTGCAGATTTTCGTGAATCATCGAATCTTTGAACGCACATTGCGCCCTTTGGTATTCC

AAAGGGCATGCCTGTTTGAGCGTCATTTCtctctcAAATCTTCGGATTTGGTTTTGAGTGATACTCTTAGTCGAACTAGG

CGTTTGCTTGAAAAGTATTGGCAAGAGTGGTACTTTAGGTGCTAAACTGTTTCAATGTATTAGGTTTATCCAACTCGTTG

AATCTGGTTAGTTACTTTAGGGTGCTTAGGCTCGGCCTTACAACAACAAACAAAGTTTGACCTCAAATCAGGTAGGATTA

CCCGCTGAACTTAA

>OTU_13

GAAATGCGATAAGTAATGTGAATTGCAGAATTCAGTGAATCATCGAATCTTTGAACGCACCTTGCGCCTTTTGGTATTCC

GAAAGGCATGCCTGTTTCAGTGTCATGAAATCTCAATCTAATATGTTTTCTGAACATGTTAGGCTTGGACTTGGGTGTCT

GCCAGTGATGGCTCACCTCAAATGACTTAGTGGAACATCCCACATCAGTGTTAGACGTAATAAGTTTCGTCtctcCTTGT

GGTGATGACTGCTCAGAACCTGCCATCGCGCATCTTTTGACTTTGACCTGAAATCAGGTAGGGCTACCCGCTGAACTTAA

>OTU_14

GAAATGCGATAACTAATGTGAATTGCAGAATTCAGTGAATCATCGAGTCTTTGAACGCACATTGCGCCcccTGGTATTCC

GGggggCATGCCTGTCCGAGCGTCATTGCTGCCCTCAAGCCCGGCTTGtgtgtTGGGTCGCCGTCCcccTCTCCGGgggg

ACGGGCCCGAAAGGCAGCGGCGGCACCGCGTCCGATCCTCGAGCGTATGGGGCTTTGTCACATGCTCTGTAGGATTGGCC

GGCGCCTGCCGACGTTTTCCAACCATTttttCCAGGTTGACCTCGGATCAGGTAGGGATACCCGCTGAACTTAA

>OTU_15

GAAATGCGATAAGTAGTGTGAATTGCAGAATTCAGTGAATCATCGAATCTTTGAACGCACATTGCGCCCCTTGGTATTCC

ATGGGGCATGCCTGTTCGAGCGTCATTTGTACCTTCAAGCTCTGCTTGGTGTTGGGTGTTTGTCTCGCCTCTGCGTGTAG

ACTCGCCTCAAAACAATTGGCAGCCGGCGTATTGATTTCGGAGCGCAGTACATCTCGCGCTTTGCACTCATAACGACGAC

GTCCAAAAGTACATTtttACACTCTTGACCTCGGATCAGGTAGGGATACCCGCTGAACTTAA

>OTU_16

GAAATGCGATACGTAATGTGAATTGCAGAATTCAGTGAATCATCGAGTCTTTGAACGCACATTGCGCCCTCTGGTATTCC

GGAGGGCATGCCTGTCCGAGCGTCATTGCTGCCCTCAAGCACGGCTTGtgtgtTGGGCTCCGTCCTCCTTCCGGgggACG

GGCCCGAAAGGCAGCGGCGGCACCGCGTCCGGTCCTCAAGCGTATGGGGCTTTGTCACCCGCTTTGTAGGACTGGCCGGC

GCCTGCCGATCAACCAAACTTttttCCAGGTTGACCTCGGATCAGGTAGGGATACCCGCTGAACTTAA

>OTU_17

GAAATGCGATAAGTAATGTGAATTGCAGAATTCAGTGAATCATCGAATCTTTGAACGCACATTGCGCCCCGTGGTATTCC

GCGGGGCATGCCTGTTCGAGCGTCATTTCACCACTCGAGTCTGACTCGGTATTGGGCGCCGCGTTTCGATGCgcgcCTTA

AAGTTTCCGGCTGGACCGTCCGTCTCCGAGCGTTGTGGCATCTGTCTCGCTAGGGAGTCGCGGAGGGCGTGGGCCGTTAA

ACACCCCATCAAAGGTTGACCTCGGATCAGGTAGGGATACCCGCTGAACTTAA

>OTU_18

GAAATGCGATAAGTAATGTGAATTGCAGAATTCAGTGAATCATCGAATCTTTGAACGCACCTTGCGCTCCTTGGTATTCC

GAGGAGCATGCCTGTTTGAGTGTCATGAAACCCTCAAACCCAAGTTTTGGATTTCGATCCATGCTTGAGTTTGGATTTGG

ATGTTTGCCGGTGATGAACCGACTCATCTTAAAAGTATTAGCTTGGATCTGTCTATATGACTGGTTTGACTTGGCATAAT

AAGTATTTTGCTGAGGACATCTTCGGATGGCCAGGACCTAGACTACTGTCTGCTAACTAAACCATCACTTTAAGTGCATC

TTTGGATGTTACTCATTGTGTAACTTTGACATCTGGCCTCAAATCAAGTAGGACTACCCGCTGAACTTAA

>OTU_19

AAAATGCGATAAGTAATGTGAATTGCAGAATTCAGTGAATCATCGAATCTTTGAACGCACATTGCGCCCGCCAGTATTCT

GGCGGGCATGCCTGTTCGAGCGTCATTTCAACCCTCAAGCTCAGCTTGGTGTTGGGACTCGCGGTAACCCGCGTTCCCCA

AATCGATTGGCGGTCACGTCGAGCTTCCATAGCGTAGTAATCATACACCTCGTTACTGGTAATCGTCGCGGCCACGCCGT

AAAACCCCAACTTCTGAATGTTGACCTCGGATCAGGTAGGAATACCCGCTGAACTTAA

>OTU_20

GAAATGCGATAAGTAATGTGAATTGCAGAATTCAGTGAATCATCGAATCTTTGAACGCACCTTGCGCTCGTTGGTATTCC

GACGAGCATGCCTGTTTGAGTGTCATTAAATTCTCAACCTCATCGCTTTTGTGATGAAGGCTTGGATGTGGAGGTTTTCT

GCAGGCCGAAAGGTCTGCTCCTCTCAAATGAATTAGTGGGTGCCCCGCGCAAACCTATCTATTGGTGTGATAATTATCTA

CGCCGTGGATTTAGGATTGCTGTAAaaaGGTGTTTGCCCTGCTTCTAACCGTCTTCGGACAACTTGAACCATTTGACCTC

AAATCAGGTAGGACTACCCGCTGAACTTAA

>OTU_21

GAAATGCGATACGTATTGTGAATTGCAGATTTTCGTGAATCATCGAATCTTTGAACGCACATTGCACCCTCTGGTATTCC

AGAGGGTATGCCTGTTTGAGCGTCATTTCtctctcAAACCTTCGGGTTTGGTATTGAGTGATACTCTGTCAAGGGTTAAC

TTGAAATATTGACTTAGCAAGAGTGTACTAATAAGCAGTCTTTCTGAAATAATGTATTAGGTTCTTCCAACTCGTTATAT

CAGCTAGGCAGGTTTAGAAGTATTTTAGGCTCGGCTTAACAACAATAAACTAAAAGTTTGACCTCAAATCAGGTAGGACT

ACCCGCTGAACTTAA

>OTU_22

GAAATGCGATAAGTAATGTGAATTGCAGAATTCAGTGAATCATCGAATCTTTGAACGCACCTTGCGCCCTTTGGTATTCC

GAAGGGCATGCCTGTTTGAGTGTCATGAAACCTCACCCCACTTGGGTTtttGCCTGAGCGGTGGTGTATTGGGTGTTGCC

TTGCTAAAGGCTCGCCTTAAAGACATAAGCACCTTGGATGTAATACGTTTCATCCTTCTGGGTGGCTGATAACCCCACAT

ATTCATGATCTGGCCTCAAATCAGGTAGGGCTACCCGCTGAACTTAA

>OTU_23

GAAATGCGATAAGTAATGTGAATTGCAGAATTCAGTGAATCATCGAATCTTTGAACGCACATTGCGCCCGCTGGAATTCC

AGCGGGCATGCCTGTTCGAGCGTCATTTCACCCCTCAAGCCTTGCTTGGTGTTGGAGCACTACCGTTTGTATAAAGCGGT

AGGCTCTGAAATTCAGTGGCGGGCTCGCTAAGACTCTGAGCGTAGTAGTTTATCACCTCGCTTTGGAAGAATTAGCGGTG

CTCTTGCCGTAAAACCccccAAATTTCTGAATTTTGACCTCGGATCAGGTAGGAATACCCGCTGAACTTAA

>OTU_24

GAAATGCGATAAGTAGTGTGAATTGCAGAATTCAGTGAATCATCGAATCTTTGAACGCACATTGCGCCCCTCGGTATTCC

GTGGGGCATGCCTGTTCGAGCGTCATTTACCCCTCAAGCTCTGCTTGGTGTTGGGCGTTGTCCCGCTTCGcgcgAGGACT

CGCCCCAAAGGTATTGGCAGCGGTCGTGCCACCCCTCGCGCAGCACATTGCGCTTCTTGAGGCGGTCGATCAGCGTCCAC

AAAGCTCACATCACATCTTGACCTCGGATCAGGTAGGGATACCCGCTGAACTTAA

>OTU_25

GAAATGCGATAAGTAATGTGAATTGCAGAATTCAGTGAATCATCGAGTCTTTGAACGCACATTGCGCCcccTGGTATTCC

GGggggCATGCCTGTCCGAGCGTCATTGCACCCCTCAAGCCCGGCTTGTCCTTGGGTTATGTCCCTCCGTTCCGGAGGGA

CAGGCCTCAAATGCAATGGCGGCACCGCGTCCGGTCCTCGAGCGTATGGGGCTTTGTCACCCGCTTTGTAGGCCGGCCGG

TCGCTTGCCCTTCAAGCACAACTTCTTATGTTGACCTCGGATCAGGTAGGGATACCCGCTGAACTTAA

>OTU_26

GAAATGCGATAAGTAATATGAATTGCAGATTTTCGTGAATCATCGAATCTTTGAACGCACATTGCGCCCTCTGGTATTCC

AGAGGGCATGCCTGTTTGAGCGTCATTTCtctctcAAACCcccGGGTTTGGTATTGAGTGATACTCTTAGTCGAACTAGG

CGTTTGCTTGAAAAGTATTGGCATGGGTAGTACTGGATAGTGCTGTCGACCTCTCAATGTATTAGGTTTATCCAACTCGT

TGAATGGTGTGGCGGGATATTTCTGGTATTGTTGGCCCGGCCTTACAACAACCAAACAAGTTTGACCTCAAATCAGGTAG

GAATACCCGCTGAACTTAA

>OTU_27

GAAACGCGATAGGTATTGTGAATTGCAGAATCAGGGAATCATCGAATTtttGAACGCACCTTGCGCTCCCTGGTATTCCT

AGGAGCATGCCTGTTTGAGTGTTGATAGCCTCTCCAAACCTTGGTTttttATTAAATCGAGTGCTTTGGGTCCCTGGGCC

TGTAGCGGCGACGTTACTTGCCTTAAAAGGATCAAAGAAGACCCAATCGGATGTTAAGCATGATATCCTTTGGGGTCTTT

GAACGACTATAAACTATACATACAACCTCAAATCAGGTAGGACTACCCGCTGAACTTAA

>OTU_28

GAAATGCGATAAGTAATGTGAATTGCAGAATTCAGTGAATCATCGAATCTTTGAACGCACATTGCGCCCACCAGTACTCT

GGTGGGCATGCCTGTCCGAGCGTCATTTCAACCCTCAGGGCCCGTCCGCGGGACCTGGTGTTGGGGATCGGCCCCTACCG

GCCGGCCCCGAAATACAGTGGCGGCACACCCGCGACCTCCTCTGCGTAGTAGCAATGCCTCGCAGCTGGATAGCGGATGC

GCCTCGCCGTAAAACCccccACTTACCAAAGGTTGACCTCGGATCAGGTAGGAATACCCGCTGAACTTAA

>OTU_29

GAAATGTGATAAGTAATGTGAATTGCAGAATTCAGTGAATCATCGAATCTTTGAACGCATCTTGCACTctctGGTATTCC

GGAGAGTATGTCTGTTTGAGTGTCATGAATTCTTCAACCCTCCCTTTTCTTAGTGAATTGAGAGGAGTTTGGATTCTGAG

TGTTGCTCCTAACCCGAGCTCATTCGTAATACATTAGCATCCATATTCGATTTCGGATTGACTTGGCGTAATAGACTATT

CGCTGAGGAATCTAACTTCGGTTAGAGCCGTTtttGAACTAGGAAGCTGCTAATCTAGCTTAGTCTACCTTTAGATTAGA

TCTCAAATCAGATAGGATTACCCGCTGAACTTAA

>OTU_30

GAAATGCGATAAGTAATGTGAATTGCAGAATTCAGTGAATCATCGAATCTTTGAACGCACATTGCGCCCATTAGTATTCT

AGTGGGCATGCCTGTTCGAGCGTCATTTCAAACCCTTAAGCCCTGTTGCTTAGCGTTGGGAGCCTGCTGTAAAAGGCAGC

TCCTCAAAGTTATTGGCGGAGTTTTAGCAATCTCTAAGCGTAGTAATTTATTCTCGTTTCTGTAGTTGCTCTAGCCCCTG

CCGTAAAACCCCAACTTTTCAGGTTTGACCTCGGATCAGGTAGGAATACCCGCTGAACTTAA

>OTU_31

GAAATGCGATACGTAATGTGAATTGCAGAATTCAGTGAATCATCGAATCTTTGAACGCATCTTGCGCTctctGGTATTCC

GGAGAGCATGTCTGTTTGAGTGTCATGAATTCTTCAACCCAATCTTTTCTTGTAATCGATTGGTGTTTGGATTCTGAGCG

TTGCTGGCGTTTGCCTAGCTCGTTCGTAATACATTAGCATCCCTAATACAAGTTTGGATTGACTTGGCGTAATAGACTAT

TCGCTAAGGATTCGGTGGAAACATCGAGCCAACTTCATTAAGGAAGCTCCTAATTTAAAAGTCTACCTTTTGATTAGATC

TCAAATCAGGCAGGATTACCCGCTGAACTTAA

>OTU_32

GAAATGCGATAAGTAATGTGAATTGCAGAATTCAGTGAATCATCGAATCTTTGAACGCACATTGCGCCcccTGGTATTCC

GGggggCATGCCTGTTCGAGCGTCATTACAACCAATCCAGCCCGGCTGGGTGTTGGGCGTCGCGGCCTGCCGcgcgcCTC

AAAGTCCTCGGCGGAAGCCGCCCGTTCCTCTGCGTGATGATCCATCGCCGCTTGGGAGTCGGgggAGAGCGCCTGCACGC

GTCGACGGAGACGTCGACTTCAAGGTTGACCTCGGATCAGGTAGGGATACCCGCTGAACTTAA

>OTU_33

GAAATGCGATAAGTAATGTGAATTGCAGAATTCAGTGAATCATCGAATCTTTGAACGCACATTGCGCCCATTAGTATTCT

AGTGGGCATGCCTGTTCGAGCGTCATTTCAACCCTTAAGCCTAGCTTAGTGTTGGGAATCTGCTGTATTGCAGTTCCTCA

AATACAACGGCGGATCTGTAACATCCTCTGAGCGTAGTAAATTtttATCTCGCTTTTGTTAGGTGTTGCAGCTCTCAGCC

GCTAAACCccccAATTtttGTGGTTGACCTCGGATCAGGTAGGAATACCCGCTGAACTTAA

>OTU_34

GAAATGCGATAAGTAATGTGAATTGCAGAATTCAGTGAATCATCGAATCTTTGAACGCACATTGCGCCCCTTGGTATTCC

GAGGGGCATGCCTGTTCGAGCGTCATTTCACCACTCAAGCCTCGCTTGGTATTGGGCGCCGCGGTGTTCCGcgcgcCTCA

AAGTCTCCGGCTGAGCTGTCCGTCTCTAAGCGTTGTGATTTCATTAATCGCTTCGGAGCGCGGGCGGTCGCGGCCGTTAA

ATCTTTCACAAGGTTGACCTCGGATCAGGTAGGGATACCCGCTGAACTTAA

>OTU_35

GAAATGTGATAAGTAATGTGAATTGCAGAATTCAGTGAATCATCGAATCTTTGAACGCACCTTGCGCTctctGGTATTCC

GGAGAGCATGTCTGTTTGAGTGTCATGAACTCTTCAACCTACCGGTTTCTAGTAAATCGGATCAGTGTTTGGATTCTGAA

CGCCTGCTGGCCTTTATTGGCGAAGCTCGTTCGTAAATGATTAGCATCTCATATTCGAAAATCGGATTGACTCAGTGTAA

TAGACTATTCGCTGAGGACGCTCTTTTGGGAGTGGCCGAGTTTTGTAAAGTAGAAGCTTCTAATTCTATTAGTCAACTTT

AGATTAGACCTCAGATCAGGCAGGATTACCCGCTGAACTTAA

>OTU_36

GAAATGCGATAAGTAATGTGAATTGCAGAATTCAGTGAATCATCGAATCTTTGAACGCACATTGCGCCCTCTGGTATTCC

GGAGGGCATGCCTGTTCGAGCGTCATTTCAACCCTCAAGCCTGGCTTGGTGATGGGGCACTGCTTCTTACCCAAGAAGCA

GGCCCTGAAATTCAGTGGCGAGCTCGCCAGGACCCCGAGCGCAGTAGTTAAACCCTCGCTCTGGAAGGCCCTGGCGGTGC

CCTGCCGTTAAACCcccAACTTCTGAAAATTTGACCTCGGATCAGGTAGGAATACCCGCTGAACTTAA

>OTU_37

GAAATGCGATAAGTAATGTGAATTGCAGAATTCCGTGAATCATCGAATCTTTGAACGCACATTGCGCCCCTCGGTATTCC

GGggggCATGCCTGTTCGAGCGTCATTACACCAATCAAGCCCCGGCTTGGTCTTGGGCGTCGCGGTCTCCCGCGTGCCTC

AATGTCGCCGGCTGGTGCGACCGTCTCTAAGCGTTGTGAATCAACAGTCGCTCCGGAGGTCGGTCGGGCTGCGCCGTCAA

GCCTTtttttACAGGTTGACCTCGGATCAGGTAGGGATACCCGCTGAACTTAA

>OTU_38

GAAATGCGATAAGTAGTGTGAATTGCAGAATTCAGTGAATCATCGAATCTTTGAACGCACATTGCGCCCCTTGGTATTCC

ATGGGGCATGCCTGTTCGAGCGTCATTTGTACCCTCAAGCTCTGCTTGGTGTTGGGTGTTTGTCTCCTCTGGGAGACTCG

CCTCAAAACAATTGGCAGCCGGCATATTGGTATCGGAGCGCAGCACAAGTCGCGCTTCTGTCCATGTTTGTTGGCATCCA

GCAAGACCATTtttCACTCTTGACCTCGGATCAGGTAGGGATACCCGCTGAACTTAA

>OTU_39

GAAATGTGATAAGTAATGTGAATTGCAGAATTCAGTGAATCATCGAATCTTTGAACGCATCTTGCACTCCTTGGTATTCC

GAGGAGTATGTCTGTTTGAGTGTCATGAATTCTTCAACCCTctctTTTCTTAGTGATTAGagagGAGTTTGGATTCTGAG

TGTTGCTCCTAACCCGAGCTCATTCGTAATGTATTAGCATCCATATTCGAATTTCGGATTGACTTGGCGTAATAGACTAT

TCGCTGAGGAATCTAACTTCGGTTAGAGCCGTGTTTGAACTAGGAAGCTACTAATCTAGCTTAGTCTACTTTTAGATTAG

ACCTCAAATCAGATAGGATTACCCGCTGAACTTAA

>OTU_40

GAAATGCGATAAGTAATGTGAATTGCAGAATTCAGTGAATCATCGAATCTTTGAACGCAACTTGCGCCTTTTGGTATTCC

GAAAGGCATGCCTGTTTGAGTGTCATATAAACCTCACCTCTACTGTTtttCATTAGACATGTGGACTGGTGCTTTGGGCG

TCTGCCGTTTACTCTGGCTCGCCTCAAATGTCTAAGTGGAATGCGTAATAAGTCTTCGCAACCCTCCACTCTAATACAAA

CTTTTATATTCTGACCTCAAATCAGGTAGGACTACCCGCTGAACTTAA

>OTU_41

GAAATGCGATAAGTAGTGTGAATTGCAGAATTCAGTGAATCATCGAATCTTTGAACGCACATTGCGCCCTTTGGTATTCC

TTAGGGCATGCCTGTTCGAGCGTCATCTAAACCTTCAAGCACTGCTTGGTGTTGGGTGCCTGTCCCGCCcccGcgcgTGG

ACTCACCTCAAATCCATTGGCGGCCCTCACGTCGGCTACGAGCGCAGCAGAAACGCGAACTTGTGGACCCGGCGGAGTGG

CTCCCAGAAGCTACATTCACCATTTTGACCTCGGATCAGGTAGGGATACCCGCTGAACTTAA

>OTU_42

GAAATGCGATAAGTAATGTGAATTGCAGAATTCAGTGAATCATCGAATCTTTGAACGCACATTGCGCCcccTGGTATTCC

GGggggCATGCCTGTTCGAGCGTCATTTCACCACTCAAGCCTCGCTTGGTATTGGGCGACGCGGTCCGCCGcgcgcCTCA

AATCGACCGGCTGGGTCTTCTGTCCCCTCAGCGTTGTGGAAACTATTCGCTAAAGGGTGCCACGGGAGGCCACGCCGAAa

aaCAAACCCATTTCTAAGGTTGACCTCGGATCAGGTAGGGATACCCGCTGAACTTAA

>OTU_43

AAAATGCGATAAGTAATGTGAATTGCAGAATTGTGAATCATCGAATCTTTGAACGCACATTGCGCCTTTTGGTATTCCAA

AAGGCATGCCTGTTTGAGCGTGATTACATCTTCTTAAATCAACTTTATTGTTTGAATTTTAAGGTATTGGAAATTTTGTA

CTAATTttttAGTACAATTTTCTGAAATACATTGGTGATCTTGTGAAATTCCAATTTATAAaaaaaCGTATTAGGTTTTA

TCCACTCGTTtttAATTATATTATTGACTTTTCGCAAATTTATAGATCTGTCCAAAATTTGTTTTATCACTTTCCACCTC

AAATCAAGTAGGACTACCCGCTGAACTTAA

>OTU_44

GAAATGCGATAAGTAATGTGAATTGCAGAATTTAGTGAATCATCGAATCTTTGAACGCACATTGCGCCCCTTGGCATTCC

GAGGGGCATGCCTGTTCGAGCGTCATAACACCccccTCAAGCTGCCCTTGTGGTGGCTTCGGTGTTGGGGCTCGTCGCAG

TTTTGCGGTGGCCCTTAAAGACAGTGGCGGTCCCCGCGTGGGCTCTACGCGTAGTAACTTGTTCCTCGCGACAGAGTGAC

GCTCGTGATCAGCCAGAACCACCCACCTTTGTCCGGTCATCCGGATTGACAGGTTGACCTCGAATCAGGTAGGAATACCC

GCTGAACTTAA

>OTU_45

GAAATGCGATAAGTAATGTGAATTGCAGAATTCAGTGAATCATCGAATCTTTGAACGCACCTTGCGCCTTTTGGTATTCC

GAAAGGCATGCCTGTTTGAGTGTCATGAAATCTCAATCCTCCTAGGTTtttGACCTGGTTGGACTTGGACATGGGCGTCT

GCCAGCAATGGCTCGCCTTAAATGACTTAGTGGAACATCCCACATCAGTGTTAGACGTAATAAGTTTCGTCtctcCTTGT

GGTGATGACTGCTCATAATCTGCCATCGCGCACTTTTGACTTTGACCTCAAATCAGGTAGGGCTACCCGCTGAACTTAA

>OTU_46

GAAATGCGATACGTAATGTGAATTGCAGAATTCCGTGAATCATCGAATCTTTGAACGCACATTGCGCCCCTTGGTATTCC

AGGgggCATGCCTGTTTGAGCGTCATTTCCTTCTCAAACACATGTGTTTGGTTGTGAGTGACACTCTGGTATGGAGTTAG

CTTGAAATTGTTGGCTGTATAAGCTGTCTTTAATTTCTCAAaaaGAATATTCTCAGTGAGAGTTGATTtttGTCGTATTA

GGTTTTACCAACTTCGACTGTGGTCAGTTTGAACTGGgggTGCGAAGTTttttttCTGAAAGGGATGGTGGCTTTTGGCG

AACAATACTCTTTAAGTTTGACCTCAAATCAGGTAGGAATACCCGCTGAACTTAA

>OTU_47

GAAATGCGATAAGTAATGTGAATTGCAGAATTCAGTGAATCATCGAATCTTTGAACGCACATTGCGCCCTCTGGTATTCC

GGggggCATGCCTGTTCGAGCGTCATTATAACCACTCAAGCTCTCGCTTGGTATTGGGGTTCGCGGTTTCGCGGCTCCTA

AAATCAGTGGCGGTGCCTGTCGGCTCTACGCGTAGTAATACTCCTCGCGTCTGGGTCCGGTAGGTCTACTTGCCAGCAAC

CcccAATTtttACAGGTTGACCTCGGATCAGGTAGGGATACCCGCTGAACTTAA

>OTU_48

GAAATGCGATAAGTAATGTGAATTGCAGAATTCAGTGAATCATCGAATCTTTGAACGCACCTTGCGCCTCTTGGTATTCC

GAGAGGTATGCCTGTTTGAGTGTCATGAAACCTCACCcccTCTGGGTTtttGCCTAGTCGGTGGTGGATTGGGCGTCTGC

CGTTACTGGCTCGCCTGAAAAGCATAAGCACCTTGGATGTAATACGTTTCATCCTGTCCGGTGGCTGATAACCCCACATA

TTCATGATCTGGCCTCAAATCAGGTAGGGCTACCCGCTGAACTTAA

>OTU_49

GAAATGCGATAAGTAGTGTGAATTGCAGAATTCAGTGAATCATCGAATCTTTGAACGCACATTGCGCCCCTTGGTATTCC

ATGGGGCATGCCTGTTCGAGCGTCATTTGTACCCTCAAGCTCTGCTTGGTGTTGGGTGTTTGTCCACCATCGTGGACTCG

CCTTAAAGTCATTGGCAGCCAGTGTTTTGGTATTGAAGCGCAGCACATTTTGCGCCTCTAGTCTAGAACACTAGCGTCCA

GTAAGCCTTtttCCACTTTTGACCTCGGATCAGGTAGGGATACCCGCTGAACTTAA

>OTU_50

GAAATGCGATAAGTAGTGTGAATTGCAGAATTCAGTGAATCATCGAATCTTTGAACGCACATTGCGCCCTTTGGTATTCC

TTAGGGCATGCCTGTTCGAGCGTCATTTACACCTTCAAGCTCTGCTTGGTGTTGGGCGTCTGTCCTGCCCCTCGGGGCGT

GGACTCGCCTTAAATCTATTGGCGGCCGGTACCGTGGCTTTGAGCGCAGCACATCAGCGACCTCATCGCCGGAGCGTATC

GACTCCCAGAAGCCCATTTTCTATGTTTTGACCTCGGATCAGGTAGGGATACCCGCTGAACTTAA

>OTU_51

GAAATGCGATAAGTAATGTGAATTGCAGAATTCAGTGAATCATCGAATCTTTGAACGCACCTTGCGCTCCTTGGTATTCC

GAGGAGCATGCCTGTTTGAGTGTCATGAAAACCCTCAACCCTAGATTGGTTAAAACCTTTCTTTGGTTTGGATTTGGACG

TTTGCCGATGATAAGTCGGCTCGTCTTAAAAGTAATAGCTGGATCTGTCTCGCGACATGGTTTGACTTGGCGTAATAAGT

ATTTCGCTAAGGACATCTTCGGATGGCCGCGTTGCAGGACTAAAGACCGCTTTCTAATCCATTGATCTTCGGATTAATAC

TCTTGACATCTGGCCTCAAATCAGGTAGGACTACCCGCTGAACTTAA

>OTU_52

GAAATGCGATAAGTAATGTGAATTGCAGAATTCAGTGAATCATCGAATCTTTGAACGCACATTGCGCCCATTAGTATTCT

AGTGGGCATGCCTGTTCGAGCGTCATTTCAACCCTTAAGCCTAGCTTAGTATTGGGAATCGACTTTACTGTCGTTCCTCA

AATTCAACGGCGGATTTATAGCAATCTCTGAACGTAGTAATTtttttCCTCGTTtttGAAATACTATAAACCTCAGCCGC

TAAACCcccAATTtttttATGGTTGACCTCGGATCAGGTAGGAATACCCGCTGAACTTAA

>OTU_53

GAAATGCGATAAGTAGTGTGAATTGCAGAATTCAGTGAATCATCGAATCTTTGAACGCACATTGCGCCCCTTGGTATTCC

ATGGGGCATGCTTGTTCGAGCGTCATTTGTACCCTCAAGCCTTGCTTGGTGTTGGGTGTTTGTCCTTTCCCTGCGTTAGG

ACTCGCCTTAAATTCATTGGCAGCCGATatatTGGTTTGAAGCGCAGCACAATTTGCGATTCAGGCTGGTTACTATCTGC

GTCCAGACAGTACAATTAACACTTTTGACCTCGGATCAAGTAGGGATACCCGCTGAACTTAA

>OTU_54

GAAATGCGATAAGTAATGTGAATTGCAGAATTCAGTGAATCATCGAATCTTTGAACGCACATTGCGCCCATTAGTATTCT

AGTGGGCATGCCTGTTCGAGCGTCATTTCAAACCCTTAAGCCCTGTTGCTTAGCGTTGGGAGCCTGCCGTAAAAGGCAGC

TCCTCAAAGTCATCGGCGGAGTTTCAGTAATCTCTAAGCGTAGTAATTCGTTCTCGCTTCTGAAGTGCTGTGGCCCCTGC

CGCTAAACACCCAATTtttCAGGTTTGACCTCGGATCAGGTAGGAATACCCGCTGAACTTAA

>OTU_55

AAAATGCGATAAGTAATGTGAATTGCAGAATTCAGTGAATCATCGAATCTTTGAACGCACATTGCGCCCGCTGGTATTCC

GGCGGGCATGCCTGTTCGAGCGTCATTTCAACCCTCAAGCCcccGGGTTTGGTGTTGGGGATCGGCTCTGCCCTTCTGGG

CGGTGCCGCCcccGAAATACATTGGCGGTCTCGCTGCAGCCTCCATTGCGTAGTAGCTAACACCTCGCAACTGGAACGCG

GCGCGGCCATGCCGTAAAACCCCAACTTCTGAATGTTGACCTCGGATCAGGTAGGAATACCCGCTGAACTTAA

>OTU_56

GAAATGCGATACGTAATGTGAATTGCAGAATTCAGTGAATCATCGAATCTTTGAACGCATCTTGCGCTctctGGTATTCC

GGAGAGCATGTCTGTTTGAGTGTCATGAATTCTTCAACCCAACTGTTTCTTGTAAACAGCTGGTGTTTGGATTCTGAGCG

CTGCTGGCTTCGTGCCTAGCTCGTTCGTAATACATCAGCATCCCTAATACAAGTCTGGATTGACTTGGCGTAATAGACTA

TTCGCTAAGGATTCAATGTTCTGCATTGAGCCAACTTAATTAAGGAAGCTTCTAATTGAAAAGTCTACCTTTAGATTAGA

TCTCAAATCAGGCAGGATTACCCGCTGAACTTAA

>OTU_57

GAAATGCGATACGCAATGCGAATTGCAGAACCGTGAGTCATCAGATCTTTGAACGCAAGTGGCGATGGTTTCGGCCATCA

TGTTTGTTTCAGTgtgtTCGGTTAATTACATCATAGAATTTAATGTGACTGAAGCGATTCTTTCACTGAATATGAGGAAG

TGTCAGCCAGATGACATTAAGCGAACTAGTCACTAATTTGGTGATAATCGTTTAAGTGTTCCTGGTTGTAAATCACTGCC

TAGTAATCAACTATCCAATCATAACACCTGAAATAAGCAAGAATACCCGCTGAACTTAA

>OTU_58

GAAATGCGATAAGTAGTGTGAATTGCAGAATTCAGTGAATCATCGAATCTTTGAACGCACATTGCGCCCCTTGGTATTCC

ATGGGGCATGCCTGTTCGAGCGTCATTTGTACTCTCAAGCTCTGCTTGGTGTTGGGTGCTTGTCCTGTCCCCTGCGGTAG

GACTCGCCTAAAAGTAATTGGCAGCTAGGTATCCGGTAGTAGAGCGCAGCACAAATTGCGATGTACGCCGGCATAATCAG

CATCCATTAAGCATATAAACACGCTTGACCTCGGATCAGGTAGGGATACCCGCTGAACTTAA

>OTU_59

GAATTGCGATAAGTATTGTGAATTGCAGATTTTCGTGAATCATCGAATCTTTGAACGCATATTGCACTCTATAGTATTCT

GTAGAGTATGCTTGTTTGAGCGTCATTTCATTCTTAAACCTCTAGGTTTAGTATTGGAGGATGTAATTCAATTTATTGGA

TAACTCTTCTGAAATGACTTGGCATCTTTACCTTGTGTAAATCTAAGCTTGTATGAACTTATTATTAGGTCTAACCAACT

AATGAGTACTCATCTTGCGTTTGCATCATGGACATATAAAGTGCCTAACAAAATTTATCATTAATTTTGACCTCAAATCA

AGTAAGGATACCCACTGAACTTAA

>OTU_60

GAAATGCGATAAGTAGTGTGAATTGCAGAATTCAGTGAATCATCGAATCTTTGAACGCACATTGCGCCCCTTGGTATTCC

ATGGGGCATGCCTGTTCGAGCGTCATTTGTACCCTCAAGCTCTGCTTGGTGTTGGGTGTTTGTCCACTCCCTGGTGTCTG

GACTCGCCTTAAAGCAATTGGCAGCCAGTGTTTTGGTATTGAAGCGCAGCACATTTTGCGTCTCCAGCCTAGAACGCTTG

CGTCCAGTAAGCCTTtttCCACTTTTGACCTCGGATCAGGTAGGGATACCCGCTGAACTTAA

>OTU_61

GAAATGCGATAACTAATGTGAATTGCAGAATTCAGTGAATCATCGAGTCTTTGAACGCACATTGCGCCccccTGGTATTC

CGGggggCATGCCTGTCCGAGCGTCATTGCTGCCCTCAAGCCCGGCTTGtgtgtTGGGTCGCCGTCCcccTCTCCGGggg

gACGGGCCCGAAAGGCAGCGGCGGCACCGCGTCCGATCCTCGAGCGTATGGGGCTTTGTCACATGCTCTGTAGGATTGGC

CGGCGCCTGCCGACGTTtttCCAACCATTttttCCAGGTGACCTCGGATCAGGTAGGGATACCCGCTGAACTTAA

>OTU_62

GAAATGCGATACGTAATGTGAATTGCAGAACTCAGTGAATCATCGAATTtttGAACGCACCTTGCGCTCTATGGTTATTC

CGTAGAGCATGCTTGTTTGAGTGTCGCGAACTTCTCAACCCCGCCTATTCATTTAGGTAGTTTGGGCTTGGATCATGATT

GTTTGCATTAGCTAGTTCTAGTGCTCGATTGAAATACAACAAGTTGGATAAACGTACAGTTACCGCTTGACCTGATGTTG

TAAAACACTTCGTCGGGGATGGTATATTGATGGTTTAATGAACTTTAACGACCTTATAACCACTTAGTGCTTGTCACTAG

TGCATTATGACCTCAAATCAAGTAGGACTACCCGCTGAACTTAA

>OTU_63

GAAATGCGATAAGTAATGTGAATTGCAGAATTCAGTGAATCATCGAATCTTTGAACGCACCTTGCGCTCCTTGGTATTCC

GAGGAGCATGCCTGTTTGAGTGTCATGAAACTCTCAAACCCAAGTTTTGGATTTCGATCCATGCTTGAGTTTGGATTTGG

ATGTTTGCCGGTGATAAGCCGACTCATCTTAAAAGTATTAGCTAGATCTGTCTCTATGACTGGTTTGACTTGGCATAATA

AGTATTTTGCTGAGGACATCTTCGGATGGCCAGGACCTTGACTATTGTCTGCTAACTAAACCATTACCTTAAGTGCATCT

TTGGATGTTACTCATTGTATAACTTTGACATCTGGCCTCAAATCAGGTAGGACTACCCGCTGAACTTAA

>OTU_64

GAAATGCGATAAGTAATGTGAATTGCAGAATTCAGTGAATCATCGAATCTTTGAACGCACATTGCGCCCGCCAGTATTCT

GGCGGGCATGCCTGTCCGAGCGTCATTTCAACCCTCGGGCCccccccTTTTCCCCTCGCGGgggAGGgggCGGGCCCGGC

GTTGGGGCCCAGGCGTCCTCCAAGGGCGCCTGTCCCCGAAACCCAGTGGCGGCCTCGCCGCTGCCTCCTCCGCGTAGTAG

CACAAACCTCGCGGGCGGAAGGCGGCGCGGCCACGCCGTAAAACCCCAAACTTTTACCAAGGTTGACCTCGGATCAGGTA

GGAATACCCGCTGAACTTAA

>OTU_65

GAAATGCGATAAGTAGTGTGAATTGCAGAATTCAGTGAATCATCGAATCTTTGAACGCACATTGCGCCCATTGGTATTCC

AATGGGCATGCCTGTTCGAGCGTCATTTGTACCCTCAAGCCTTGCTTGGTGTTGGGTGTTTGTCCTCTAGGACTCGCCTT

AAAACAATTGGCAGCCGGCATATTGGTTTCGGAGCGCAGCACAAATTGCGGTCCTTCCATGAATGTCGGCATCCATGAAG

CCcccATTTTCCACTTTTGACCTCGGATCAGGTAGGGATACCCGCTGAACTTAA

>OTU_66

GAAATGCGATACTTGGTGTGAATTGCAGAATCCCGTGAACCATCGAGTCTTTGAACGCAAGTTGCGCCCCAAGCCTTCTG

GCCGAGGGCACGTCTGCCTGGGTGTCACAAATCGTCGTCCccccATCCTCTCGAGGATATGGGACGGAAGCTGATCTCCC

GTgtgtTACCGCACGCGGTTGGCCAAAATCCGAGCTAAGGACGTCAGGAGCGTCTTGACATGCGGTGGTGAATTTAATTC

TCGTCATATAGTCAGACGTTCCGGTCCAAAAGCTCTTGATGACCCAAAGTCCTCAACGCGACCCCAGGTCAGGCGGGATC

ACCCGCTGAGTTTAA

>OTU_67

GAAATGCGATAATTAATGTGAATTGCAGAATTCAGTGAATCATCGAGTCTTTGAACGCACATTGCGCCcccTGGTATTCC

GGggggCATGCCTGTCCGAGCGTCATTGCTGCCCTCAAGCCCGGCTTGTTTGTTGGGTCTTGTTCCTCCGGGAACAGGCC

CGAAAGGCAGTGGCGGCACCGTCCGATCCTCGAGCGTATGGGGCTTTGTCACCCGCTCTGTAGGCCCGGCCGGCGCTTGC

CGACCcccATCAATCTTttttCAGGTTGACCTCGGATCAGGTAGGGATACCCGCTGAACTTAA

>OTU_68

AAAATGCGATAAGTAATGTGAATTGCAGAATTCAGTGAATCATCGAATCTTTGAACGCACATTGCGCCCGCCAGTATTCT

GGCGGGCATGCCTGTTCGAGCGTCATTTCAACCCTCAAGCCCAGCTTGGTGTTGGGAGCTGCAGTCCTGCTGCACTCCCC

AAATACATTGGCGGTCACGTCGAGCTTCCATAGCGTAGTAATTTACACATCGTTACTGGTAATCGTCGCGGCCACGCCGT

TAAACCCCAACTTCTGAATGTTGACCTCGGATCAGGTAGGAATACCCGCTGAACTTAA

>OTU_69

AAAATGCGATAAGTAATGTGAATTGCAGAATTCAGTGAATCATCGAATCTTTGAACGCACATTGCGCCCGCCAGTATTCT

GGCGGGCATGCCTGTTCGAGCGTCATTTCAACCCTCAAGCACAGCTTGGTGTTGGGACTCGCGTTAATTCGCGTTCCTCA

AATTGATTGGCGGTCACGTCGAGCTTCCATAGCGTAGTAGTAAAACCCTCGTTACTGGTAATCGTCGCGGCCACGCCGTT

AAACCCCAACTTCTGAATGTTGACCTCGGATCAGGTAGGAATACCCGCTGAACTTAA

>OTU_70

GAAATGCGATACGTAGTGTGAATTGCAGAATTCAGTGAATCATCGAATCTTTGAACGCACATTGCGCCCTTTGGTATTCC

AAAGGGCATGCCTGTTCGAGCGTCATTTGTACCCTCAAGCTTTGCTTGGTGTTGGGCGTCTTTTGTCTCCAGTTCGCTGG

AGACTCGCCTTAAAGTCATTGGCAGCCGGCCTACTGGTTTCGGAGCGCAGCACAAGTCGCGCTCTTTGCCAGCCAAGGTC

AGCGTCCAGCAAGCCTTtttttCAACCTTTGACCTCGGATCAGGTAGGGATACCCGCTGAACTTAA

>OTU_71

GAAATGCGATACGTAGTGTGAATTGCAGAATTCAGTGAATCATCGAATCTTTGAACGCACATTGCGCCCTTTGGTATTCC

AAAGGGCATGCCTGTTCGAGCGTCATTTGTACCCTCAAGCTTTGCTTGGTGTTGGGCGTCTTTGTCtctcACGAGACTCG

CCTTAAAATGATTGGCAGCCGACCTACTGGTTTCGGAGCGCAGCACAATTCTTGCACTTTGAATCAGCCTTGGTTGAGCA

TCCATCAAGACCACATTtttttCAACTTTTGACCTCGGATCAGGTAGGGATACCCGCTGAACTTAA

>OTU_72

GAAATGCGATAAGTAGTGTGAATTGCAGAATTCAGTGAATCATCGAATCTTTGAACGCACATTGCGCCCCTCGGTATTCC

GTGGGGCATGCCTGTTCGAGCGTCATCTACACCCTCAAGCTCTGCTTGGTGTTGGGCGTCTGTCCCGCCTCCGcgcgTGG

ACTCGCCCCAAATTCATTGGCAGCGGTCCTTGCCTCCTCTCGCGCAGCACATTGCGCTTCTCGAGGGGCTACGGCTCGCG

TCCAACAAGCACATTTACCGTCTTTGACCTCGGATCAGGTAGGGATACCCGCTGAACTTAA

>OTU_73

GAAATGCGATAAGTAATGTGAATTGCAGAATTCAGTGAATCATCGAATCTTTGAACGCACATTGCGCCCTCTGGTATTCC

AGAGGGCATGCCTGTTCGAGCGTCATTTCAACCCTCAAGCCTAGCTTGGTGTTGGGGCACTACCTGACTGTTTTACAGGA

CGGTAGGCCCTGAAATTTAGTGGCGAGCTCGCCAGGACTCCGAGCGCAGTAGTAAAACCCTCGCTTTGGACTGTACTGGC

GCGGCCCTGCCGTAAAACCcccAACTTCTGAAAATTTGACCTCGGATCAGGTAGGAATACCCGCTGAACTTAA

>OTU_74

GAAATGCGATAAGTAATGTGAATTGCAGAATTCAGTGAATCATCGAATCTTTGAACGCAACTTGCGCTctctGGTATTCC

GGAGAGCATGCCTGTTTGAGTGTCATGATCtctcAACCAATAGGGTTTCTTATTGGCTTGGATCTGGGTGTTGCCAGCTT

GTCTGGCTCGCCTTAAAGGAGTTAGCGAGTAAAGCACTGTCGTCTGGCGTAATAAGTTTCGCTGGTAGACTTTGTGAAGG

TCGCTTATAATCGCCTCGGCATTTTGATCTCTGGCCTCAAATCAGGTAGGACTACCCGCTGAACTTAA

>OTU_75

AAAATGCGATAAGTAATGTGAATTGCAGAATTCAGTGAATCATCGAATCTTTGAACGCACATTGCGCCCGCCAGTACTCT

GGCGGGCATGCCTGTCTGAGCGTCATTTCAACCCTCAGGACCCGTTCGCGGGACCTGGCGTTGGGGATCAGCCCTCCGGG

GCTGGCCCTGAAATCTAGTGGCGGTTCtctcGCGACCTCCCCTGTGCAGTAGTAATACCTCGCAGCCGGATAGCATGAGG

GCCACGCCGTAAAACCcccTACTTCTCAAGGTTGACCTCAGATCAGGTAGGAATACCCGCTGAACTTAA

>OTU_76

GAATTGCGATAAGTAATGTGAATTGCAGAATTCAGTGAATCATCGAATCTTTGAACGCACCTTGCGCCCTTTGGTATTCC

GAAGGGCATGCCTGTTTGAGTGTCATGAAACCTCACCCCATTTAGGTTtttGCCTGAGTGGTCGGTGGATTGGGTGTTGC

CGATATACTGGCTCGCCTGAAAAGCATAAGCGCCTTGGATGTAATACGTTTCATCCTTCTGGGTGGCTGATAACCCCACA

TATCTCATGATCTGGCCTCAAATCAGGTAGGGCTACCCGCTGAACTTAA

>OTU_77

GAAATGCGATACCTAGTGTGAATTGCAGCCATCGTGAATCATCGAGTTCTTGAACGCACATTGCGCCcccTGGTATTCCG

GggggCATGCCTGTTCGAGCGTCATTTCACCACTCAAGCCTCGCTTGGTATTGGGCAACGCGGTCCGCCGCGTGCCTCAA

ATCGTCCGGCTGGGTCTTCTGTCCCCTAAGCGTTGTGGAAACTATTCGCTAAAGGGTGTTCGGGAGGCTACGCCGTAAAA

CAACCCCATTTCTAAGGTTGACCTCGGATCAGGTAGGGATACCCGCTGAACTTAA

>OTU_78

GAAATGCGATAAGTAGTGTGAATTGCAGAATTCAGTGAATCATCGAATCTTTGAACGCACATTGCGCCCCTTGGTATTCC

ATGGGGCATGCCTGTTCGAGCGTCATTTGTACCTTCAAGCTTTGCTTGGTGTTGGGTGTTTGTCCCGAGGGACTCGCCTT

AAAGTAATTGGCAGCCAGTGTTTGGTTTTGAAGCGCAGCACAAGTCGCGATTCAAGGCTACACGCCCGCTTCCACAAGCC

TTttttCACTTTTGACCTCGGATCAGGTAGGGATACCCGCTGAACTTAA

>OTU_79

GAAATGCGATACTTGGTGTGAATTGCAGAATCCCGTGAACCATCGAGTCTTTGAACGCAAGTTGCGCCCGAAGCCATTAG

GTTGAGGGCACGTCTGCCTGGGCGTCACATATCGTTGCCCGATGCCAATTTCCTCTTGATAGGAGGTATGTTGTGCAGGG

TGAATGATGGCTTCCCATGAGCACTGCTGCCTCATGGTTGGTTGAAAATTGAGTCCTTGGTAGGGTGTGCCATGATAGAC

GGTGGTTGAGTGTTGCACGAGACCATATCATGCGAGACTCTACCAAACTTGGCCTCTGTGACCCACGTGCGTCTTTGAAC

GCTCATGACGAGACCTCAGGTCAGGCGGGGCTACCCGCTGAGTTTAA

>OTU_80

GAAATGCGATAAGTAGTGTGAATTGCAGAATTCAGTGAATCATCGAATCTTTGAACGCACATTGCGCCCCTTGGTATTCC

ATGGGGCATGCCTGTTCGAGCGTCATCTACACCCTCAAGCTCTGCTTGGTGTTGGGCGTCTGTCCCGCCTCTGCgcgcgG

ACTCGCCCCAAATTCATTGGCAGCGGTCCTTGCCTCCTCTCGCGCAGCACATTGCGCTTCTCGAGGTGCGCGGCCCGCGT

CCACGAAGCAACATTACCGTCTTTGACCTCGGATCAGGTAGGGATACCCGCTGAACTTAA

>OTU_81

GAAATGCGATAAGTAGTGTGAATTGCAGAATTCAGTGAATCATCGAATCTTTGAACGCACATTGCGCCCTTTGGTATTCC

AAAGGGCATGCCTGTTCGAGCGTCATTTGTACCCTCAAGCTTTGCTTGGTGTTGGGCGTCTTGTCTCCAGTTCGCTGGAG

ACTCGCCTTAAAGTAATTGGCAGCCGGCCTACTGGTTTCGGAGCGCAGCACAAGTCGCGCTctctTCCAGCCAAGGTCAG

CATCCACAAAGCCTTttttCAACTTTTGACCTCGGATCAGGTAGGGATACCCGCTGAACTTAA

>OTU_82

GAAATGCGATAAGTAATGTGAATTGCAGAATTCAGTGAATCATCGAATCTTTGAACGCACATTGCGCCCCGTGGTATTCC

GCGGGGCATGCCTGTTCGAGCGTCATTTCACCACTCAAGCCTAGCTTGGTATTGGGCGTCGCGGTTCCGcgcgcCTTAAA

GTCTCCGGCTGAGCAGTTCGTCTCTAAGCGTTGTGGCATatatTTCGCTGAAGAGTTCGGACGGCTTTTGGCCGTTAAAT

CTTTCTCAAGGTTGACCTCGGATCAGGTAGGGATACCCGCTGAACTTAA

>OTU_83

GAAATGCGATAAGTAATGTGAATTGCAGAATTCAGTGAATCATCGAATCTTTGAACGCATCTTGCGCTCCTTGGTATTCC

GAGGAGCATGCCTGTTTGAGTGTCATTATATTCTCAACTCTCCTATACTTTGTTGTAAAGGAGAGCTTGGATTGTGGAGG

CTTGCTGGCCCCTTGCTTGAGGTCAGCTCCTCTGAAATGCATTAGCGGAACTGTTTGCGATCTGCCACAAGTGTGATAAG

TTATCTACACTGGCGCGGGGATTGCTctctGTGTTCAGCTTCTAATCGTCTCAGTGACAATTTCTTGAATGCTTGACCTC

AAATCAGGTAGGACTACCCGCTGAACTTAA

>OTU_84

AAAATGCGATAAGTAATGTGAATTGCAGAATTCAGTGAATCATCGAATCTTTGAACGCACATTGCGCCCGCCAGTATTCT

GGCGGGCATGCCTGTTCGAGCGTCATTTCAACCCTCAAGCCcccGGGTTTGGTGTTGGGGATCGGGCTGTACTCCAGCCC

GGCCCCGAAATCTAGTGGCGGTCTCGCTGCAGCCTCCATTGCGTAGTAGCTAACACCTCGCAACTGGAACGCGGCGCGGC

CAAGCCGTTAAACCcccAACTTCTGAATGTTGACCTCGGATCAGGTAGGAATACCCGCTGAACTTAA

>OTU_85

GAAATGCGATACGTAGTGTGAATTGCAGAATTCAGTGAATCATCGAATCTTTGAACGCACATTGCGCCCTTTGGTATTCC

AAAGGGCATGCCTGTTCGAGCGTCATTTGTACCCTCAAGCTTTGCTTGGTGTTGGGCGTTtttttGTCTTGTTCCAAGAC

TCGCCTTAAAACGATTGGCAGCCGGCCTACTGGTTTCGGAGCGCAGCACATTtttGCGCTTGCAATCAGCAAAAGAGGTC

GGCAATCCATCAAGTCCATTTCTCACTTTTGACCTCGGATCAGGTAGGGATACCCGCTGAACTTAA

>OTU_86

AAAATGCGATAAGTAATGTGAATTGCAGAATTCAGTGAATCATCGAATCTTTGAACGCACATTGCGCCCGCCAGTATTCT

GGCGGGCATGCCTGTTCGAGCGTCATTTCAACCCTCAAGCCcccGGGTTTGGTGTTGGGGATCGGCGAGCCCTTGCGGCA

AGCCGGCCCCGAAATCTAGTGGCGGTCTCGCTGCAGCTTCCATTGCGTAGTAGTAAAACCCTCGCAACTGGTACGCGGCG

CGGCCAAGCCGTTAAACCcccAACTTCTGAATGTTGACCTCGGATCAGGTAGGAATACCCGCTGAACTTAA

>OTU_87

GAAATGCGATAAGTAGTGTGAATTGCAGAATTCAGTGAATCATCGAATCTTTGAACGCACATTGCGCCCCTCGGTATTCC

GTGGGGCATGCCTGTTCGAGCGTCATTTACACCCTCAAGCTCTGCTTGGTGTTGGGCGTCTGTCCCGCCTCCGTGCGCGG

ACTCGCCTCAAAGTCATTGGCAGCGGTCTCGTCGGCTTCTCGCGCAGCACATTTGCGCTTCTCGGAGCCCCGGCGGATCA

GCGTCCAGCAAGCAATTTCATGACTTGACCTCGGATCAGGTAGGGATACCCGCTGAACTTAA

>OTU_88

AAAATGCGATACTTGGTGTGAATTGCAGAATCCCGTGAATCATCGAGTCTTTGAACGCAAGTTGCGCCCGAAGCCATTAG

GCCGAGGGCACGCCTGCCTGGGCGTCACGCACCCGTCGCCccccACCACCTCCCTCCcccTCCGGACGGGAGGCGGAGAG

GgggCGGACATTGGCCTCCCGTGGGCGCCCCAGCCCGCGGTTGGCCGAAAATCGGTCCCGCGGCGACGTACGCCACGACG

AGCGGTGGATTTCGCACGGCTCGGCGTCGcgcgcgTCACGTCGCCTCAGGG

>OTU_89

GAAATGCGATAAGTAATGTGAATTGCAGAATTCAGTGAATCATCGAATCTTTGAACGCACCTTGCGCTCCTTGGTATTCC

GAGGAGCATGCCTGTTTGAGTGTCATGAAAACCCTCAACCTTAGATTGGTTATTTGACCTTTCTTTGGCTTGGATTTGGA

CGTTTGCCGATGTCAAGTCGGCTCGTCTTAAAAGTAATAGCTGGATCTGTCTCGCGACATGGTTTGACTTGGCGTAATAA

GTATTTCGCTAAGGACATCTTCGGATGGCCCTGTTGCAGGACTAAAGACCGCTTTCTAATCCATTGATCTTCGGATTAAT

ATTCATAACATCTGGCCTCAAATCAGGTAGGACTACCCGCTGAACTTAA

>OTU_90

GAAATGCGATAAGTAATGTGAATTGCAGAATTCAGTGAATCATCGAATCTTTGAACGCACATTGCGCCCATTAGTATTCT

AGTGGGCATGCCTGTTCGAGCGTCATTTCAACCCCTAAGCACAGCTTATTGTTGGGACTCTACGGCTTCGTAGTTCCCCA

AAGACATTGGCGGAGTGGCAGCAGTCCTCTGAGCGTAGTAATTCTTTATCTCGCTTTTGTTAGGCGCTGCCcccccGGCC

GTAAAACCcccAATTttttCTGGTTGACCTCGGATCAGGTAGGAATACCCGCTGAACTTAA

>OTU_91

GAAATGCGATAAGTAATGTGAATTGCAGAATTCAGTGAATCATCGAATCTTTGAACGCACATTGCGCCCCTTGGTATTCC

GAGGGGCATGCCTGTTCGAGCGTCATTACACCACTCAAGCACTGCTTGGTATTGGGCATCGTCCGTCGAAAGGCGGGCGT

GCCTCGAAGACCTCGGCGGGGTTTCTCCAACTTCGGGCGTAGTAGAGTTAAATCGAACGTCTTATAAGCTTGGTGGGACT

CCATTGCCGTTAAACCTTTTATTTTCTAGGTTGACCTCGGATCAGGTAGGGATACCCGCTGAACTTAA

>OTU_92

GAAATGCGATAAGTAGTGTGAATTGCAGAATTCAGTGAATCATCGAATCTTTGAACGCACATTGCGCCCCTTGGTATTCC

ATGGGGCATGCCTGTTCGAGCGTCATTACACCCTCAAGCTCTGCTTGGTGTTGGGCGCCTGTCCCGCCTTGTGCGTGGAC

TCGCCCTAAATGTATTGGCAGCGGCTTGCCAGCCCGTAGCGTAGCACTATTGCGCCTGCGAGCTTTGGCGGTTTAGCGTC

CACTAAGTGACCACCCACAGTTTGACCTCGGATCAGGTAGGGATACCCGCTGAACTTAA

>OTU_93

GAAATGCGATAACTAATGTGAATTGCAGAATTCAGTGAATCATCGAGTCTTTGAACGCACATTGCGCCcccTGGTATTCC

GGggggCATGCCTGTCCGAGCGTCATTTCTCCCCTCCAGCCCCGCTGGTTGTTGGGCCGCGCCcccccGGgggCGGGCCT

CGAgagaAACGGCGGCACGTCCGGTCCTCGAGCGTATGGGGCTCTGTCACCCGCTCTATGGGCCCGGCCGGGGCTTGCCT

CGACCcccAATCTTCTCAGATTGACCTCGGATCAGGTAGGGATACCCGCTGAACTTAA

>OTU_94

GAAATGCGATAAGTAATGTGAATTGCAGAATTCAGTGAATCATCGAATCTTTGAACGCACATTGCGCCCGCCAGTATTCT

GGCGGGCATGCCTGTTCGAGCGTCATTTCAACCCTCAGGCCcccAGTGCCTGGTGTTGGGGATCGGCCCAGCCTTCTCGC

AAGGCCGCCGGCCCCGAAATCTAGTGGCGGTCTCGCTGTAGTCCTCCTCTGCGTAGTAGCACAACCTCGCAGTTGGAACG

CGGCGGTGGCCATGCCGTTAAACACCCCACTTCTGAAAGTTGACCTCGGATCAGGTAGGAATACCCGCTGAACTTAA

>OTU_95

GAAATGCGATAAGTAATGTGAATTGCAGAATTCAGTGAATCATCGAATCTTTGAACGCACATTGCGCCCATTAGTATTCT

AGTGGGCATGCCTGTTCGAGCGTCATTTCAACCCTTAAGCCCCAGTTGCTTAGTGTCGGGAGACTGCCGTAAAACGCAGC

TCCTCAGAGTTAGCTGGCAGAGTTAGGTCATACTCTAGGCGTAGTAAATCTTTACATCTCGCTTCTTTGTAGTTGTCCTG

GCCCTCGCCATAAAACACCCCAATTtttCTATGGTTGACCTCGGATCAGGTAGGAATACCCGCTGAACTTAA

>OTU_96

GAAATGCGATAAGTAATGTGAATTGCAGAATTCAGTGAATCATCGAATCTTTGAACGCACCTTGCGCCCTCTGGTATTCC

GGAGGGCATGCCTGTTTGAGTGTCATGAACACCTCACCcccTCGGGTTTCCGATCGGGTGGATTTGGGCGCTGCCAGTCA

CATGGCTCGCCTTAAAAGCATTAGCGGTTGTCGACGTAATAAGTTTCGTCCTGTCGACCGCTCCTAATCTCCTTCGGGAT

GTCtctcGGGACACTTCATGATCTGACCTCAAATCAGGTAGGACTACCCGCTGAACTTAA

>OTU_97

GAAATGCGATAAGTAGTGTGAATTGCAGAATTCAGTGAATCATCGAATCTTTGAACGCACATTGCGCCCTTTGGTATTCC

TTAGGGCATGCCTGTTCGAGCGTCATTTAAACCTTCAAGCTCAGCTTGGTGTTGGGTGACTGTCCGCCGCCTCCGGGCGG

TCGGACTCGCCTCAAATTGATTGGCGGCCGGTACTTTGGCTTCGAGCGCAGCAGAAACGCGAACTCGAGGCCGTCGTCCT

GGCTCCCAGAAGCTATCTTCACAATTTTGACCTCGGATCAGGTAGGGATACCCGCTGAACTTAA

>OTU_98

GAAATGCGATACGTAATGTGAATTGCAGAATTCAGTGAATCATCGAATCTTTGAACGCACCTTGCACTCTTTGGTATTCC

GAAGAGTATGTCTGTTTGAGTGTCATGAAACTCTCAACCcccTCATTTTGTAATGAAATGTAGCGTGGGCTTGGTTTATG

GCTGTCTGTCGGCGTAAATGCCGGCTCAGCTGAAATATACGAGCAACCCAGTTTAAATACTTGACGGCTTGACTCGGCGT

AATAACTATTTCGCTGAGGACGTCTAACCTTTAAATCGTTAGTGGTGCTTCTAATGCATTAATCATTTTAAGCTTTAGAC

CTCAAATCAGTCAGGACTACCCGCTGAACTTAA

>OTU_99

GAAATGCGATAAGTAGTGTGAATTGCAGAATTCAGTGAATCATCGAATCTTTGAACGCACATTGCGCCCCTTGGTATTCC

ATGGGGCATGCCTGTTCGAGCGTCATTTGTACCCTCAAGCTCTGCTTGGTGTTGGGTGTTTGTCTCGCGGTTCTGCGCAG

ACTCGCCTTAAAACAATTGGCAGCCGGCTTGTTAGCCTGGAGCGCAGCACATATTGCGCCTCTTGCTAGCGACTGCCCGG

CACCCATCAAGCCCATTTATTTGCTCTTGACCTCGGATCAGGTAGGGATACCCGCTGAACTTAA

>OTU_100

GAATTGCGATAAGTAATGTGAATTGCAGATACTCGTGAATCATTGAATTtttGAACGCACATTGCGCCCTTGAGCATTCT

CAGGGGCATGCCTGTTTGAGCGTCATTTCCTTCTCAAAAGATAATTTATTATTttttGGTTGTGGGCGATACTCAGGGTT

AGCTTGAAATTGGAGACTGTTTCAGTCTTttttAATTCAACACTTAGCTTCTTTGGAGACGCTGTTCTCGCTGTGATGTA

TTTATGGATTTATTCGTTtttACTTTACAAGGGAAATGGTACGTACCTTAGGCAAAGGGTTGCTTTTAATATTCATCAAG

TTTGACCTCAAATCAGGTAGGATTACCCGCTGAACTTAA

>OTU_101

GAAATGCGATAAGTAATGTGAATTGCAGAATTCAGTGAATCATCGAATCTTTGAACGCACCTTGCGCCCTTTGGTATTCC

GAAGGGCATGCCTGTTTGAGTGTCATGAAACCTCACTCCACTTGGGTTtttGCCTGAGTGGTAGTGTATTGGGTGTTGCC

TTGCCAAAGGCTCGCCTTAAAAGAATAAGCACCTTGGATGTAATACGTTTCATCCTTCTGGGTGGCTAATAACCCCACAT

AACTCATGATCTGGCCTCAAATCAGGTAGGGCTACCCGCTGAACTTAA

>OTU_102

GAAATGCGATACGTAATGTGAATTGCAGAATTCAGTGAATCATCGAGTCTTTGAACGCACATTGCGCCcccTGGTATTCC

GGggggCATGCCTGTCCGAGCGTCATTTCTGCCCTCAAGCCCGGCTTGtgtgtTGGGCCGCGTCCccccGGGGACGGGCC

CGAAAGGCAGCGGCGGCACCGCGTCCGGTCCTCGAGCGTATGGGGCTTTGTCACCCGCTCTTGCAGGCCCGGCCGGCGCC

AGCCGACCcccAACCAATTTTCTCAGGTTGACCTCGGATCAGGTAGGGATACCCGCTGAACTTAA

>OTU_103

GAAATGCGATAAGTAGTGTGAATTGCAGAATTCAGTGAATCATCGAATCTTTGAACGCACATTGCGCCCCTTGGTATTCC

ATGGGGCATGCCTGTTCGAGCGTCATTTGTACCCTCAAGCTCTGCTTGGTGTTGGGTGTTTGTCCACTCCcccGTGTTTG

GACTCGCCTTAAAGCAATTGGCAGCCAGTGTTTTGGTATTGAAGCGCAGCACATTTTGCGTCTCTAGCCTGTAGATACTG

GCGTCCAGTAAGCCTTtttCCACTTTTGACCTCGGATCAGGTAGGGATACCCGCTGAACTTAA

>OTU_104

GAAATGCGATAAGTAATGTGAATTGCAGAATTCAGTGAATCATCGAATCTTTGAACGCACCTTGCGCCTTTTGGTATTCC

GAAAGGCATGCCTGTTTGAGTGTCATGAAATATCAACCCCTCCTGGTTTCTGATCAGTGTTGGGCTTGGACTTGGGTGTC

TGCCAGTTCGCTGGCTCGCCTTAAAAGAGTTAGTGGTTATAACATCCACGGCTAAGACGTAATAAGTTTCGTCTGGTCAA

GGTGTGACGACTGCTCATAATCCGACCGCAAGGTCATTATTTTATGCTCTGACCTCAAATCAGGTAGGACTACCCGCTGA

ACTTAA

>OTU_105

GAAATGCGATACTTGGTGTGAATTGCAGAATCCCGTGAACCATCGAGTTtttGAACGCAAGTTGCGCCCGAAGCCACTAG

GCCGAGGGCACGTCTGCCTGGGCGTCAcacacCGTTGCCcccccTGAACCTCGCCAATCCCTCTACAGGAGAGGCAGCCA

GGAGGGGCGGAGATTGGCCTCCCATGAGCTTTTGTCTCGTGGTTGGCCTAAATTCGAGTCATCGGCTGCGATCGCCGCGA

CATTCGGTGGTTTTCGATTGTATCGGTGCCCCGTCGTGCGCGAATCTGCAGCTGAGTGGACCAATGCGACCCCAATGCAT

TACATTGTAGTGCCTTCAACGCGACCCCAGGTCAGGCGGGATTACCCGCTGAATTTAA

>OTU_106

GAAATGCGATAAGTAATGTGAATTGCAGAATTCAGTGAATCATCGAATCTTTGAACGCATCTTGCGCCCTTTGGTATTCC

GAAGGGCATGCCTGTTTGAGTGTCATATAAACCTCACCTCTACTGTTtttCATTGAACATGTGGATCGGTGCTTTGGGCG

TCTTGCCGTTTACTCTGGCTCGCCTTAAATGTCTAAGTGGAATGCGTAATAAGTCTTCGCAACCCTCCACTCTAATACAA

ACTTTTATATTCTGACCTCAAATCAGGTAGGACTACCCGCTGAACTTAA

>OTU_107

GAAATGCGATAAGTAGTGTGAATTGCAGAATTCAGTGAATCATCGAATCTTTGAACGCACATTGCGCCCCTTGGTATTCC

ATGGGGCATGCCTGTTCGAGCGTCATTTGTACTCTCAAGCTCTGCTTGGTGTTGGGTGTTTGTCCCGCGTTGCGTGTGGA

CTCGCCTTAAAGCAATTGGCAGCCGGCATATTGGCCTTGGAACGCAGCACATTTTGCGATCCTAGTCATACTTGTTGGCA

TCCATCAAGCCTATACATTAACGCTTGACCTCGGATCAGGTAGGGATACCCGCTGAACTTAA

>OTU_108

GAAATGCGAAAAGTAGTGTGAATTGCAGAATTCAGTGAATCATCGAATCTTTGAACGCACATTGCGCCCCTTGGTATTCC

ATGGGGCATGCCTGTTCGAGCGTCATTTGTACCCTCAAGCTCTGCTTGGTGTTGGGTGTTTGTCTCGCCATTGCgcgcAG

ACTCGCCTTAAAGCAATTGGCAGCCGGCAAAAGCCTGGGAGCGCAGCACATGCGTTGCTTTCAGACTGCTTGTCGAGCGT

CCAGCAAGCCTATTATTCTTGCGTTTGACCTCGGATCAGGTAGGGATACCCGCTGAACTTAA

>OTU_109

GAAATGCGATAAGTAATGTGAATTGCAGAATTCAGTGAATCATCGAATCTTTGAACGCACATTGCGCCCCTTGGTATTCC

GAGGGGCATGCCTGTTCGAGCGTCATTACACCACTCAAGCTATGCTTGGTATTGGGCGTCGTCCTTAGTTGGGCgcgcCT

TAAAGACCTCGGCGAGGCCTCACCGGCTTTAGGCGTAGTAGAATTTATTCAATTAACGTCTGTCAATGGAGAGGACTTCT

GCCGACTGAAACCTTTATTttttACAGGTTGACCTCGGATCAGGTAGGGATACCCGCTGAACTTAA

>OTU_110

GAAATGCGATACGTAATGTGAATTGCAGAATTCCGTGAATCATCGAATCTTTGAACGCACATTGCGCCCCTTGGTATTCC

AGGgggCATGCCTGTTTGAGCGTCATTTCCTTCTCAAATACTTGTATTTGGTTGTGAGTGACACTCAGTCTTGCATTGAG

TTAACTTGAAATTGTTGGCCGTAGCGGTTGTTGCAGCTTGTCGTTtttGTGCAATGGTATGTTTTCTTTACTATTAAACA

GGATGCGTGAGCATGTCGTATTAGGTTTTACCAACTCCGGCAGACTCTGTATCTTGGAAGAGCGTACTGGCATTGAGAAG

ATTGAACTGTCGACCGTGGCAAACAGTACTCTTTAAGTTTGACCTCAAATCAGGTAGGAATACCCGCTGAACTTAA

>OTU_111

GAAATGCGATAACTAATGTGAATTGCAGAATTCAGTGAATCATCGAGTCTTTGAACGCACATTGCGCCCTCTGGTATTCC

GGAGGGCATGCCTGTCCGAGCGTCATTGCTGCCCTCAAGCCCGGCTTGtgtgtTGGGTCTCGTCCcccccGGgggACGGG

CCCGAAAGGCAGCGGCGGCACCGTGTCCGGTCCTCGAGCGTATGGGGCTTTGTCACCCGCTCTGTAGGCCCGGCCGGCGC

CTGTCGACCcccAATCTATTtttttCAGGTTGACCTCGGATCAGGTAGGGATACCCGCTGAACTTAA

>OTU_112

GAAATGCGATAAGTAATGTGAATTGCAGAATTCAGTGAATCATCGAATCTTTGAACGCACATTGCGCCCACCAGTACTCT

GGTGGGCATGCCTGTCCGAGCGTCATTTCAACCCTCAGGGCCCGTTCGCGGGACCTGGTGTTGGGGATCGGCCCCACCGG

CCGGCCCCGCAAATACAGTGGCGGCACACCCGCGACCTCCTCTGCGTAGTAGCAATGCCTCGCAGCTGGATAGCGGTTGC

GCCTCGCCGTAAAACCccccACTTCTCAAAGGTTGACCTCGGATCAGGTAGGAATACCCGCTGAACTTAA

>OTU_113

GAAATGCGATAAGTAGTGTGAATTGCAGAATTCAGTGAATCATCGAATCTTTGAACGCACATTGCGCCCTTTGGTATTCC

AAAGGGCATGCTTGTTCGAGCGTCATTTGTACCCTCAAGCTTTGCTTGGTGTTGGGTGTTTGTCCTTTCCCTGCGTCAGG

ACTCGCCTTAAATTCATTGGCAGCCTATGTACTGGTTTGAAGCGCAGCACAATTTGCGATTCAGGCGGTTACCAGGGGCG

TCCAGTAAGCACAGTTAACACTTTTGACCTCGGATCAAGTAGGGATACCCGCTGAACTTAA

>OTU_114

GAAATGCGATAAGTAGTGTGAATTGCAGAATTCAGTGAATCATCGAATCTTTGAACGCACATTGCGCCCTTCGGTATTCC

GTTGGGCATGCCTGTTCGAGCGTCATTTAAACCTTCAAGCTCTGCTTGGTGTTGGGTGTTTGTTCCGCCTCAGCGCGTGG

ACTCGCCTCAAATTCATTGGCAGCCGGTATGTTGGCTTCGTGCGCAGCACATTGCAAGCGACTTCAACAGACCTTCTTCC

ATAAGCTCTTtttttACTTTGACCTCGGATCAGGTAGGGATACCCGCTGAACTTAA

>OTU_115

GAAATGCGATAAGTAGTGTGAATTGCAGAATTCAGTGAATCATCGAATCTTTGAACGCACATTGCGCCCTTTGGTATTCC

TTAGGGCATGCCTGTTCGAGCGTCATTTAACCCCTCAAGCTCAGCTTGGTGTTGGGCGTCCTGTCCcccccGGgggACTC

GCCTCAAATCGATTGGCGGCCGGTACGTTGGCTTCGAGCGCAGCAGAAACGCGAACTCGATGCCCGGCGGGCCGGCTCCC

ACAAGCCACACCTCTTATTTTGACCTCGGATCAGGTAGGGATACCCGCTGAACTTAA

>OTU_116

GAAATGCGATAAGTAATGTGAATTGCAGAATTCAGTGAATCATCGAATCTTTGAACGCACATTGCGCTCGCCAGCATTCT

GGCGAGCATGCCTGTTCGAGCGTCATTTCAACCCTCAAGCACCGCTTGGTTTGGGGCCCACGGCCGACGTGGGCCCTTAA

AGGTAGTGGCGGACCCTCCCGGAGCCTCCTTTGCGTAGTAACTAACGTCTCGCACTGGGATCCGGAGGGACTCTTGCCGT

TAAACCcccAAATTCTTTACAGGTTGACCTCGGATCAGGTAGGAATACCCGCTGAACTTAA

>OTU_117

GAAATGCGATAAGTAATGTGAATTGCAGAATTCAGTGAATCATCGAATCTTTGAACGCACCTTGCGCCTTTTGGTATTCC

GAAAGGCATGCCTGTTTGAGTGTCATGAAATCTCAATCCcccTGGGTTTATGATCTGGGTCGGACTTGGATATGGGCGTC

TGCCGGTCACACGGCTCGCCTCAAATGACTTAGTGGATCtctctGCATCCGTGACAGACGTAATAAGTTTCGTCTTGTCC

CTTGCTTATGAGTCTGCTCATAACCTGCCATCGCGCACTTTAGACTCTGACCTCAAATCAGGTAGGACTACCCGCTGAAC

TTAA

>OTU_118

GAAATGCGATAAGTAATGTGAATTGCAGAATTCAGTGAATCATCGAATCTTTGAACGCACATTGCGCCCTCTGGTATTCC

GGgggCATGCCCGTTCGAGCGTCATTACACCACTCAAGCCTCGCTTGGTCTTGGGCGCCGCGGCCTGCCCGcgcgcCTCA

AAGTCACCGGCCGGGTGACCCGTCTCGGAGCGTCGTAGCCATCGTGTTTCGCTCGCGGGGATCGGGTCGCCGTCGGCGCC

GTTAAACGTCtctcATTAGGTTGACCTCGGATCGGGTAGGGATACCCGCTGAACTTAA

>OTU_119

GAAATGCGATAAGTAATGTGAATTGCAGAATTCAGTGAATCATCGAATCTTTGAACGCACATTGCGCCCCTTGGTATTCC

GAGGGGCATGCCTGTTCGAGCGTCATTACACCACTCAAGCTATGCTTGGTATTGGGTGCCGTCCTTCACTGGGCgcgcCT

TAAAGACCTCGGCGAGGCCTCACCGGCTTTAGGCGTAGTAGAATTTATTCGAACGTCTGTCAAAGGAGAGGACTTCTGCC

GACTGAAACCTTTTATTtttCTAGGTTGACCTCGGATCAGGTAGGGATACCCGCTGAACTTAA

>OTU_120

GAAATGCGATAAGTAATGTGAATTGCAGAATTCCGTGAATCATCGAATCTTTGAACGCACATTGCGCCcccTGGCATTCC

GGggggCATGCCTGTCCGAGCGTCATTTCTGCCCTCAAGCACGGCTTGtgtgtTGGGTGCGGTCCccccGGGGACCTGCC

CGAAAGGCAGCGGCGACGTCCGTCTGGTCCTCGAGCGTATGGGGCTCTGTCACTCGCTCGGGAAGGACCTGCGGgggTTG

GTCACCACCATATTTTACCACGGTTGACCTCGGATCAGGTAGGAGTTACCCGCTGAACTTAA

>OTU_121

GAAATGCGATACCTAGTGTGAATTGCAGCCATCGTGAATCATCGAGTTCTTGAACGCACATTGCGCCCCTCGGCATTCCG

GggggCATGCCTGTTTGAGCGTCGTTTCCATCTTGCGCGTGCGCAGAGTTGGgggAGCGGAGCGGACGACGTGTAAAGAG

CGTCGGAGCTGCGACTCGCCTGAAAGGGAGCGAAGCTGGCCGAGCGAACTAGACTTtttttCAGGGACGCTTGGCGGCCG

AGAGCGAGTGTTGCGAGACAACAAaaaGCTCGACCTCAAATCAGGTAGGAATACCCGCTGAACTTAA

>OTU_122

GAAATGCGATAAGTAATGTGAATTGCAGAATTCAGTGAATCATCGAATCTTTGAACGCACATTGCGCCCGGTGGTATTCC

GCCGGGCATGCCTGTTCGAGCGTCATTTCACCACTCAAGCCTCGCTTGGTATTGGGCGCCGCGAGCCTCTCGcgcgcCTC

AAAGTCGTCCGGCTGAGCCGTCCGTCTCGGAGCGTTGTGACTTTATTGTTTCGCTTCTCGAGCGCGGGCGACCGCGGCCG

TTAAACTTCTTTATTTCAGGTTGACCTCGGATCAGGTAGGGATACCCGCTGAACTTAA

>OTU_123

GAAATGCGATAAGTAGTGTGAATTGCAGAATTCAGTGAATCATCGAATCTTTGAACGCACATTGCGCCCCTTGGTATTCC

ATGGGGCATGCCTGTTCGAGCGTCATTTGTACCCTCAAGCTATGCTTGGTGTTGGGTGATTGTCCAGTCTTTTCTAGTCA

GGACTCACCTTAAAGTAATTGGCAGCCAGTATTTTGGTATAAAGCGCAGCACATTTTGCGATTTAGTCCAGCAATATTAG

CAACCATCAAGTTTATTTATCACTTTTGACCTCGGATCAGGTAGGGATACCCGCTGAACTTAA

>OTU_124

GAAATGCGATACTTGGTGTGAATTGCAGAATCCCGCGAATCATCGAGTCTTTGAACGCAAGTTGCGCCCGAAGCCATTCG

GCCGAGGGCACGTCTGCCTGGGTGTCACGCATCGTTGCCCCAACCCCAAACACTTCTTATGATGtgtgGGGTGCGGGGAA

GACATTGGCCTCCCGTGTGCTTCTGCTCGCGGTTAGCCTAAAAGTGAGTCCTAGGCGACGAGCGCCACGACAATCGGTGG

TTGAGAAACCCTCGTGACCCGTCGTgtgtTGCCCGTCGCTGTGAAGGTGCTCCTCGACCCTATTGCGTCGTTCCTGCGAC

TCTACCATCGCGACCCCAGGTCAGGCGGGATTACCCGCTGAATTTAA

>OTU_125

GCCCGAGGAGCAGTTGCAAATGATAAaaaaaGCTGATATAATGTTATCTCCGTCCGAGCGCCTGAACCGCAAGCTCTGGC

ACGCGATAGCCGGAGCGGCATTtttGAAGCTGGAGCTTCATATCACCGACGAGGATATCATCGGCGCGGTTCGCTGGCAC

ACTACGGGCAAAGCCAACATGACGCAGCTCGAAAAGATAGTCTACCTCGCGGATTTCATAAGCGAGGACAGAAAATATCC

GGATGTCGACAAAGTCCGAGAGCTTTCGGAACAGTCGATAGAATCCGCGATGCTCTACACCCAAAGATACT

>OTU_126

GAAATGCGATACGTAGTGTGAATTGCAGAATTCAGTGAATCATCGAATCTTTGAACGCACATTGCGCCCTTTGGTATTCC

AAAGGGCATGCCTGTTCGAGCGTCATTTGTACCCTCAAGCTTTGCTTGGTGTTGGGCGTTtttGTCTTTGGCACGCCAAA

GACTCGCCTTAAAATGATTGGCAGCCGGCCTACTGGTTTCGCAGCGCAGCACATTtttGCGCTTGCAATCAGCAAAAGAG

GACGGCAATCCATCAAGACTCCTTCTCACGTTTGACCTCGGATCAGGTAGGGATACCCGCTGAACTTAA

>OTU_127

GAAATGCGATAAGTAATGTGAATTGCAGAATTCAGTGAATCATCGAATCTTTGAACGCACCTTGCGCTctctGGTATTCC

GGAGAGCATGCCTGTTTGAGTGTCATGAAATCTTCAACCCTctctTTTCTTAGTGAATCGAGAGGTGCTTGGATCCTGAG

CGCTGCTGGCTTCGGCCTAGCTCGTTCGTAATGCATTAGCATCCGCAATCGAACTTCGGATTGACTTGGCGTAATAGACT

ATTCGCTGAGGATTCTGGTCTCGTACCAGAGCCGGGTTGGGTTAAAGGAAGCTTCTAATCCTAAAAGTCTAACTTTTGAT

TAGATCTCAAATCAGGTAGGACTACCCGCTGAACTTAA

>OTU_128

GAATTGCGATAAGTAATGTGAATTGCAGAATTCAGTGAATCATCGAATCTTTGAACGCACCTTGCACTCTTTGGTATTCC

GAAGAGTATGTCTGTTTGAGTGTCATGAAACTCTCAACCccccTATTTTGTAATGAGATGGGCGTGGGCTTGGATTATGA

CTGCTGTCGGCGTAATTGCCGGCTCAGTTGAAATACACGAGCAACCCATTTGAAATAAACGGCTTGACTCGGCGTAATAA

TTATTTCGCTGAGGACGTTTTCTTCAAAAGTAAGAGGTGCTTCTAATGCGCTTTTATAGCATTTATATTTTAGACCTCAA

ATCAGTCAGGACTACCCGCTGAACTTAA

>OTU_129

GAAATGCGATAAGTAGTGTGAATTGCAGAATTTAGCGAATCATCGAATCTTTGAACGCACATTGCGCCTCTTGGTATTCC

ATGGGGCACGCCTGTTCGAGCGTCATTTCAACCCTCAAGCACTGCTTGGTGTTGGGCGTCTGTCCCGCCTCTGCGCGTGG

ACTCGCCTCAAAGTCATTGGCGGCTGTTCCCCTTCGGCTCGTAAGCGCAGCACAGTCAGCGCCCGGAGCTCTGGTGGATG

GCGTCCAGCAAGCTACATTTCTAGTCTTGACCTCGGATCAGGTGGGGATACCCGCTGAACTTAA

>OTU_130

GAAATGCGATAAGTAATGCGAATTGCAGAATTTCCGTGAGTCATCGAATCTTTGAACGCACATTGCGCCCTTTGGCATTC

CGAAGGGCATGCCTGTTCGAGCGTCATTATCCccccTCAAACCTCGGGTTTGGTGTTGGGCCCGCGTTGGTTGGCGACGA

CCAACTGGCCTCAAAGGCAATGACGGCTCCCGCTGGGACCCTTGGTGCAATGAGCTTCTTTCGAGAGCACGCATCGAGTT

CGGACCCGGTGGGCCGGTCTCCCTTACGACTTTCTAAGGTTGACCTCGGATCAGGTAGGAATACCCGCTGAACTTAA

>OTU_131

GAATTGCGATAAGTATTGTGAATTGCAGATTTTCGTGAATCATCGAATCTTTGAACGCATATTGCACTCTATAGTATTCT

GTAGAGTATGCTTGTTTGAGCGTCATTTCACTCTTAAACCTCTAGGTTTAGTTATGGTGGAAGTGGGAATATTCTTTCCA

CTCCTCTGAAATGACTCGGCAGGTCCTTTAGTGGCTATTTATAGCTTTGGAGAATTTATTACTAGGTTTTACCATTTTAA

TAAATACCTCTAAGCTAGGTTACTTTAGGTTATCGGCCTAATCAACTGTCATCATAAATTTTGACCTCAAATCAAGTAAG

GATACCCACTGAACTTAA

>OTU_132

GAAATGCGATAAGTAGTGTGAATTGCAGAATTCAGTGAATCATCGAATCTTTGAACGCACATTGCGCCCCTTGGTATTCC

ATGGGGCATGCCTGTTCGAGCGTCATTTACACCCTCAAGCTCTGCTTGGTGTTGGGCGTCTGTCCCGCCTCGTGCGCGGA

CTCGCCCCAAAAGCATTGGCAGCGGTTTGCCAGCCCGTAGCGTAGCACTATTGCGCCTGCGAGGCCCGGCGGATCGGCGT

CCATGAAGCACAACCACAGTTTGACCTCGGATCAGGTAGGGATACCCGCTGAACTTAA

>OTU_133

GAAATGCGAAAAGTAGTGTGAATTGCAGAATTCAGTGAATCATCGAATCTTTGAACGCACATTGCGCCCCTTGGTATTCC

ATGGGGCATGCCTGTTCGAGCGTCATTTGTACCTTCAAGCTCTGCTTGGTGTTGGGTGTTTGTCCCGCTTttttGCGCGG

ACTCGCCTCAAAGCAATTGGCAGCCGGCGTACTAGCCTGGGAGCGCAGCACATTTTGCGCGTCTCAACTGGAACGCTTGC

GTCCATGAAGCCTAAATTtttGCTCTTGACCTCGGATCAGGTAGGGATACCCGCTGAACTTAA

>OTU_134

GAAATGCGATAAGTAATGTGAATTGCAGAATCAGTGAATCATCGAGTCTTTGAACGCAACTTGCGCCCTTTGGTATTCCG

AAGGGCATGCCTGTTTGAGTGTCATGAAAACCTCAATCCCTCCGGTTtttACGAACCGGTTGGACTTGGATTTGGGTGCT

GCCGCGGCTCACGCCGTCGGCTCGCCTTAAAAGTGTTAGTGGGACGGTGAACACCCGTCAGCCTGGCGTAATAAGTTTCG

CTGGGCCTCTGGGGTCGTTTGACGGCTTGCTCATAACAAACCTATTTTCTATGACTTCTGACCTCAAATCAGGTAGGGCT

ACCCGCTGAACTTAA

>OTU_135

GAAATGCGATAAGTAGTGTGAATTGCAGAATTCAGTGAATCATCGAATCTTTGAACGCACATTGCGCCCTTTGGTATTCC

TTAGGGCATGCCTGTTCGAGCGTCATTTACAAATTCAAGCTCAGCTTGGTGATGGGTGTCTGTCCCGCCTTTGCGCGTGG

ACTCGCCTCAAATGCAGTTGGCAGCTTGTTCCTCGGCTCTAAACGCAGCAGATTTGCGTCAAGCGTCGGGCGGACGGGCT

CTCCAGTAAGCAAACCCCACAAATTGACCTCGGATCAGGTAGGGATACCCGCTGAACTTAA

>OTU_136

GAAATGCGATACTTGGTGTGAATTGCAGAATCCCGTGAACCATCGAGTCTTTGAACGCAAGTTGCGCCCTAAGCCTTCTG

GCCGAGGGCACGTCTGCCTGGGTGTCACAAATCGTCGTCCccccATCtctcGAGGATAATGGACGGAAGCTGGTCTCCCG

TgtgtTACCGCACGCGGTTGGCCAAAATCCGAGCTAAGGACGCAAGGAGCGTCTCGACATGCGGTGGTGAATTAAAACCT

CGTCATACCGTTGGCCGCTCCTGTCCTGATGCTCTCGATGACCCAAAGTCCTCAACGCGACCCCAGGTCAGGCGGGATCA

CCCGCTGAGTTTAA

>OTU_137

GAAATGCGATAAGTAGTGTGAATTGCAGAATTCCGTGAATCATCGAATCTTTGAACGCACATTGCGCCCTTTGGTATTCC

TTAGGGCATGCCTGTTCGAGCGTCATTCAAACCCTCAAGCTCTGCTTGGTGTTGGGCGCCTGTCCCGCCGCTGGCGCGGA

CTCGCCTCGAAGACATTGGCGGTGGTTGTACCGATCGGAAGCGCAGCAGAGACGCGCCACTCCCGGTTCGGTGCCCTCCT

CCAGGAAGCCATACCCACGTCTTGACCTCGGATCAGGTAGGGATACCCGCTGAACTTAA

>OTU_138

GAAATGCGATAAGTAGTGTGAATTGCAGAATTCAGTGAATCATCGAATCTTTGAACGCACATTGCGCCCCTTGGTATTCC

ATGGGGCATGCCTGTTCGAGCGTCATTTGTACCCTCAAGCTCTGCTTGGTGTTGGGTGTTTGTCTACTCCTTGGTGGTTG

GACTCGCCTTAAAACAATTGGCAGCCAGTGTTTTGGTATTGAAGCGCAGCACATTTTGCGGTTCTGGCCCTGAACACTGG

CATCCAGTAAGCTCTTTTCCACTTTTGACCTCGGATCAGGTAGGGATACCCGCTGAACTTAA

>OTU_139

GAAATGTGATAAGTAATGTGAATTGCAGAATTCAGTGAATCATCGAATCTTTGAACGCATCTTGCACTCCTTGGTATTCC

GAGGAGTATGCCTGTTTGAGTGTCATGAATTCTTCAACCCTctctTTTCTTAGTGATTAGagagGAGCTTGGATTCTGAG

TGTTGCTCCTTACCCGAGCTCATTCGTAATACATTAGCATCCATATTCGAACTTTCGGATTGACTTGGCGTAATAGACTA

TTCGCTGAGGAATCTAACTTCGGTTAGAGCCGGGTTGAACTAGGAAGCTCCTAATCTAGCTTAGTCTACTTTTAGATTAG

ATCTCAAATCAGGTAGGATTACCCGCTGAACTTAA

>OTU_140

GAAATGCGATAAGTAGTGTGAATTGCAGAATTCAGTGAATCATCGAATCTTTGAACGCACATTGCGCCCCATGGTATTCC

GTGGGGCATGCCTGTTCGAGCGTCATTTACCcccTCAAGCTCCGCTTGGTGTTGGGCGTCTGTCCCGCTTCGcgcgcgGA

CTCGCCCCAAAGGTATTGGCAGCGGTCGTGCCAGCTTCTCGCGCAGCACATTGCGCTTCTCGAGGCACCGGTGGGCCCGC

GTCCATCAAGCTCACCcccccAGTTTGACCTCGGATCAGGTAGGGATACCCGCTGAACTTAA

>OTU_141

GAAATGCGATACCTAGTGTGAATTGCAGCCATCGTGAATCATCGAGTTCTTGAACGCACATTGCGCCCGTCGGTATTCCG

GCGGGCATGCCTGTCTGAGCGTCGTTTCCTTCTTGAAGCTTttttttttAAaaaaaGATTCAGAATTGGCCGTGCCACTG

GCCCGGCCGAAAAGAAACGTTGCGGACGAAGCGAACTACATCGGGACGCTTTTGCCGCCGAGCGAAAATatatCATGAGC

TCGACCTCAGATCAGGTAGGAGTACCCGCTGAACTTAA

>OTU_142

GAAATGCGATAAGTAGTGTGAATTGCAGAATTCAGTGAATCATCGAATCTTTGAACGCACATTGCGCCCCTTGGTATTCC

ATGGGGCATGCCTGTTCGAGCGTCATTTGTACCTTCAAGCTCTGCTTGGTGTTGGGTGATTGTCTATCTTTGTAGACTCG

CCTTAAAGTCATTGGCAGCCGGCATATTGGCCTGGGAGCGCAGCACAAGTCGCGCTTCTTGTCATGAATGTTGGCGTCCA

GTAAGTCTACATATTTTGCTCTTGACCTCGGATCAGGTAGGGATACCCGCTGAACTTAA

>OTU_143

GAAATGCGATAAGTAATGTGAATTGCAGAATTCAGTGAATCATCGAATCTTTGAACGCACCTTGCGCCCTCTGGTATTCC

GGAGGGCATGCCTGTTTGAGTGTCATGAACACCTCACCcccTCGGGTTTCCGATCGGGTGGATTTGGGCGTTGCCAGTCA

AATGGCTCGCCTTAAATGCATTAGCGGTTGTTGACGTAATAAGTTTCGTCTGTATGCCGCTGACAATCTCCTTCGGGATG

TCTCTGAGACAATTCATGATCTGACCTCAAATCAGGTAGGACTACCCGCTGAACTTAA

>OTU_144

AAAATGCGATAAGTAATGTGAATTGCAGAATTCAGTGAATCATCGAATCTTTGAACGCACATTGCGCCCGCCAGTATTCT

GGCGGGCATGCCTGTTCGAGCGTCATTTCAACCCTCAAACCcccGGGTTTGGTGTTGGGGATCGGATTGCTAGTCAATCC

GTCTCCGAAATCTAGTGGCGGTCTCGCTGCAGCCTCCATTGCGTAGTAGCTAACACCTCGCAACTGGAACGCGGCGCGGC

CATGCCGTTAAACACCCAACTTCTGAATGTTGACCTCGGATCAGGTAGGAATACCCGCTGAACTTAA

>OTU_145

GAAATGCGATAAGTAATGTGAATTGCAGAATTCAGTGAATCATCGAATCTTTGAACGCACCTTGCGCCCTTTGGTATTCC

GAAGGGCATGCCTGTTTGAGTGTCATGAAACCTCACCCCACTTGGGTTtttGCCTGAGCGGTGGTGTATTGGGTGTTGCC

TTGCCAAAGGCTCGCCTTAAaaaCATAAAGCACCTTGGATGTAATACGTTTCAATCCTTCTTGGGCTGGCTGGATAACCC

CACCATATTCATGATCTGGCCTCAAATCAGGTAGGGCTACCCGCTGAACTTAA

>OTU_146

GCAATGTATCGGTAGAATCAGCCGCACAATTGACTGTATAGGTATAATATGCAGGTGCTACGTTACCCTGAATACATTCG

ATGAATGACAAGGCGAATTGTGCAGGCAAATTGTAGGGGTTAGTAGTGCCCACAAGAGCCTGATatatTTCCGCACCAGT

ATACAGACCCACGATATCACCAACAACAGTACGAGAGCTGCTAACATCACCACCAGTAATAGCCAACCcccAATAATACC

CTAGTGTATTTTCGTCACTACCTAGCGGTTTGAATGGAATGATTGAGG

>OTU_147

GAAATGCGATAAGTAATGTGAATTGCAGAATTCAGTGAATCATCGAATCTTTGAACGCACATTGCGCCCGCCAGTATTCT

GGCGGGCATGCCTGTTCGAGCGTCATTTCAACCATCAAGCCCCGGGCTTGTGTTGGGGACCTGCGGCTGCCCGCAGGCCC

TGAAAAGCAGTGGCGGGCTCGCTGTCACACCGAGCGTAGTAGCATTATCTCGCTCAGGGCGTGCTGCGTGTTCCGGCCGT

TAAACAACGCCTTTTACACCCAAGGTTGACCTCGGATCAGGTAGGAAGACCCGCTGAACTTAA

>OTU_148

GAAATGCGATACGTAGTGTGAATTGCAGAATTCAGTGAATCATCGAATCTTTGAACGCACATTGCACCTCTCGGTATTCC

GGGAGGTATGCCTGTTCGAGCGTCATTTGTACCCTCAAGCTCTGCTTGGTGTTGGGTGTTTGTTCCGCCTCTGtgtgtgA

ACTCGCCTCAAAACAATTGGCAGCCGGCGTACTTGTTTCGGAGCGCAGCACATTTTGCGATCCAGATCAACGTACAGCGG

CATCCAAGAAGCCTTATTTAACGCTCTTGACCTCGGATCAGGTAGGGATACCCGCTGAACTTAA

>OTU_149

GAAATGCGATAAGTAATGTGAATTGCAGAATTCAGTGAATCATCGAATCTTTGAACGCACATTGCGCCCTCTGGTATTCC

GGggggCATGCCTGTTCGAGCGTCATTACAACCACTCAAGCTCACGCTTGGTCTTGGGGTTCGCGATTCCGCGGCCTCTA

AAATCAGTGGCGGTGCCTGTCGGCTCTACGCGTAGTAATACTCCTCGCGTTTGAGTCCGGTAGGTTTACTTGCCAGCAAC

CcccAATTtttACAGGTTGACCTCGGATCAGGTAGGGATACCCGCTGAACTTAA

>OTU_150

GAAATGCGATAAGTAATGTGAATTGCAGAATTCAGTGAATCATCGAATCTTTGAACGCACATTGCGCCcccTGGTATTCC

GGggggCATGCCTGTCCGAGCGTCATTACAACCCTCAAGCTCAGCTTGGTATTGGGCCCCGCCGACCCGGCGGGCCCTAA

AGTCAGTGGCGGTGCCGTCCGGCTCCGAGCGTAGTAATTCTTCTCGCTCTGGAGGTCCGGTCTGTGCTCGCCACGCAACC

cccAATTttttCCATGGTTGACCTCGGATCAGGTAGGGATACCCGCTGAACTTAA

>OTU_151

GAAATGCGATACGTAATATGAATTGCAGATATTCGTGAATCATCGAATCTTTGAACGCACATTGCGCCCTTTGGTATTCC

AAAGGGCATGCCTGTTTGAGCGTCATTTCTCCCTCAAACCcccGGGTTTGGTGTTGAGCAATACGCTAGGTTTGTTTGAA

AGAATTTAACGTGGAAACTTATTTTAAGCGACTTAGGTTTATCCAAAACGCTTATTTTGCTAGTGGCCACCACAATTTAT

TTCATAACTTTGACCTCAAATCAGGTAGGACTACCCGCTGAACTTAA

>OTU_152

GAAATGCGATAAGTAGTGTGAATTGCAGAATTCAGTGAATCATCGAATCTTTGAACGCACATTGCGCCCCTTGGTATTCC

ATGGGGCATGCCTGTTCGAGCGTCATTTGTACCTTCAAGCTTTGCTTGGTGTTGGGTGTTTGTCCTCCCCTTTTGTGTTT

GGACTCGCCTTAAAACAATTGGCAGCCAGTGTTTTGGTATAGAAGCGCAGCACAAGTCGCGATTCTAGCTAAGTAACGCT

GACATCCAGAAGTCTTttttAACTTTTGACCTCGGATCAGGTAGGGATACCCGCTGAACTTAA

>OTU_153

GAAATGCGATAAGTAGTGTGAATTGCAGAATTCAGTGAATCATCGAATCTTTGAACGCACATTGCGCCCTTTGGTATTCC

TTAGGGCATGCCTGTTCGAGCGTCATTTAAACCTTCAAGCTCAGCTTGGTGTTGGGTGACTGTCCGCTTCACCGCGGACT

CGCCTCAAAATTATTGGCGGCCGGTACATTGGCTCTCGAGCGCAGCAGAAACGCGTAACTCGAGGTCCTCGTGCTGGCTC

CCAGAAGCTATCTTCACAATTTTGACCTCGGATCAGGTAGGGATACCCGCTGAACTTAA

>OTU_154

AAAGTGCGATAACTAGTGTGAATTGCATATTCAGTGAATCATCGAGTCTTTGAACGCAGCTTGCACTCTATGGTTtttCT

ATAGAGTACGCCTGCTTCAGTATCATCACAAACCCAcacaTAACATTTGTTTATGTGGTAATGGGTCGCATCGCTGTTTT

ATTACAGTGAGCACCTAAAATGtgtgtgATTTTCTGTCTGGCTTGCTAGGCAGGAATATTACGCTGGTCTCAGGATCTTt

ttCTTTGGTTCGCCCAGGAAGTAAAGTACAAGAGTATAATCCAGCAACTTTCAAACTATGATCTGAAGTCAGGTGGGATT

ACCCGCTGAACTTAA

>OTU_155

GAAATGCGATACGTAATGTGAATTGCAGAATTCAGTGAATCATCGAATCTTTGAACGCATCTTGCGCTctctGGTATTCC

GGAGAGCATGTCTGTTTGAGTGTCATGAATTCTTCAACCCAATTGTTTATTAAAAGCAGTTGGTGTTTGGATTCTGAGCG

CTGCTGGCTTCGGCCTAGCTCGTTCGTAATACATCAGCATCCCTCATACAAGTTTGGATTGACTTGGCGTAATAGACTAT

TCGCTAAGGATTCAATGTTCGCATTGAGCCAACTTAATGAAGGAAGCTACTAATCACAAAGTCTACCTTTAGATTAGATC

TCAAATCAGGCAGGATTACCCGCTGAACTTAA

>OTU_156

GAAATGCGATAAGTAGTGTGAATTGCAGAATTCAGTGAATCATCGAATCTTTGAACGCACATTGCGCCCCTTGGTATTCC

ATGGGGCATGCCTGTTCGAGCGTCATTTGTACTCTCAAGCCTTGCTTGGTGTTGGGTGCTTGTCTTTGCTTTGCGTAGAC

TCTCCCTAAATACATTGGCAGCCGATatatTGGTTTGAAGCGCAGCACAATTGCTATTCAGGCTTGTTGTATCAGCATCC

ACAAAGAATTATAACACTTTTGACCTCGGATCAGGTAGGGATACCCGCTGAACTTAA

>OTU_157

GAACGTGCGAAACGTAATGTGAATTGCAGAATTCAGTGAATCATCGAATCTTTGAACGCATCTTGCGCCCCTTGGTATTC

CGAGGAGCATGCCTGTTTGAGTGTCGTGAACTACTCTCAATTTCAATCTTtttGTTTGAAGTTGGATTTGGGTGTTGTCA

TGGATCACCATGACTCGTCCTAAATGCATTAGCGATCTGGCTTGATCAcacacaATGTGATAAGAAAAGCATTGTTATAA

TTGAGTCAAGACCGCTTCTAATTGTCGCAAGACAAAAGTCATTTTCATGTCATTTGACCTCAAATCAGGTAGGACTACCC

GCTGAACTTAA

>OTU_158

GAAATGCGATAAGTAGTGTGAATTGCAGAATTCAGTGAATCATCGAATCTTTGAACGCACATTGCGCCCCTTGGTATTCC

ATGGGGCATGCCTGTTCGAGCGTCATTTGTACCCTCAAGCTTTGCTTGGTGTTGGGCGTCTTGTCCTCTTTGGAGGACTC

GCCTCAAAACAATTGGCAGCCGGCATATTGGTATCGGAGCGCAGCACAAGTCGCACTTCTTTCCATGAATGTTGGCATCC

ATCAAGACTATTtttCACTCTTGACCTCGGATCAGGTAGGGATACCCGCTGAACTTAA

>OTU_159

GAAATGCGATAAGTAATGTGAATTGCAGAATTCAGTGAATCATCGAGTCTTTGAACGCACATTGCGCCcccTGGTATTCC

GGggggCATGCCTGTCCGAGCGTCATTGCACCCCTCAAGCCCGGCTTGTCCTTGGTTATGTCCTCCGTTCCGGAGGGAGC

AGGCCTCAAATGCAATGGCGGCACCGCGTCCGGTCCTCGAGCGTATGGGGCTTTGTCACCCGCTTTGTAGGCCGGCCGGT

CGCTTGCCCTTCAAGCACAACTTCTTATGTTGACCTCGGATCAGGTAGGGATACCCGCTGAACTTAA

>OTU_160

GAAATGCGATAAGTAATGTGAATTGCAGAATTCAGTGAATCATCGAATCTTTGAACGCACCTTGCGCCCTCTGGTATTCC

GGAGGGCATGCCTGTTTGAGCGTCATGTAGACTCAACCTCCCCGGTTTATGATCGGGACGGGTTGGATGTGGGCGCTGCC

GATTGCCGGCTCGCCTCAAATGTCTTAGCGGGCTCAGAAGCCCCGACCTAGCGTAATAAGTTTCGCTGGCGAGGGCGCGG

ATGACCGCTCACAACCGCCTTTGGGCAACCTTTTGACTCTGGCCTCAAATCAGGTAGGACTACCCGCTGAACTTAA

>OTU_161

GAAATGCGATAAGTAATGTGAATTGCAGAATTCAGTGAATCATCGAATCTTTGAACGCACATTGCGCTCGCCAGTATTCT

GGCGAGCATGCCTGTTCGAGCGTCATTTCAACCATCAAGCTCTGCTTGCGTTGGGGATCCGCGGCTGTCCGCGGTCCCTC

AAAATCAGTGGCGGGCTCGCTAGTCACACCGAGCGTAGTAACTCTACATCGCTATGGTCGTGCGGCGGGTTCTTGCCGTA

AAACCccccATTTCTAAGGTTGACCTCGGATCAGGTAGGAATACCCGCTGAACTTAA

>OTU_162

GAAATGCGATAAGTAGTGTGAATTGCAGAATTCAGTGAATCATCGAATCTTTGAACGCACATTGCGCCCTATAGTATTCT

GTAGGGCATGCATGTTCGAGCGTCATTTGTACCTTCAAGCTTTGCTTGGTGTTGGGTGTTTGTCCTCTCCCTGGTGTTTG

GACTCGCCTCAAAACAATTGGCAGCCAGTGTTTTGGTATTGAAGCGCAGCACAATTTGCGATTCTGGCCTTAAAATACTA

GCGTCCATAAGTACATTCTCACTTTTGACCTCGGATCATGTAGGGATACCCGCTGAACTTAA

>OTU_163

GAAATGCGATAAGTAATGTGAATTGCAGAATTCAGTGAATCATCGAATCTTTGAACGCACATTGCGCCCATTAGTATTCT

AGTGGGCATGCCTGTTCGAGCGTCATTTCAACCCTTAAGCCTAGCTTAGTGTTGGGAATCTACTCCTTTTATTAGTTGTA

GTTCCTGAAATACAACGGCGGATTTGTAGTATCCTCTGAGCGTAGTAATTtttttCTCGCTTTTGTTAGGTGCTATAACT

CCCAGCCGCTAAACCcccAATTttttGTGGTTGACCTCGGATCAGGTAGGAATACCCGCTGAACTTAA

>OTU_164

GAAATGCGATACGTATTGTGAATTGCAGATTTTCGTGAATCATCGAATCTTTGAACGCACATTGCGCCCTTTGGTATTCC

AAAGGGCATGCCTGTTTGAGCGTCATTTCCTTCTCAAACCTTTGGGTTTGGTCGTGAGTGATACTCTTTCCAGGGTTAAC

TTGAAAATGCTGGCCATCTGGCTGCTGCTGGCTGAGGCTCTAGTCCAGTCTGCTGATACTCGACGTATTAGGTTTTACCA

ACTCGTTGTGGCATCGGTCGGGCGTTATTACGACTCTAGCACGAAAGTACAGACAGCCTGGCGAACAGTATTCATAAAGT

TTGACCTCAAATCAGGTAGGATTACCCGCTGAACTTAA

>OTU_165

GAAATGCGATAAGTAATGTGAATTGCAGAATTCAGTGAATCATCGAATCTTTGAACGCACATTGCGCCCGGTGGTATTCC

GCCGGGCATGCCTGTTCGAGCGTCATTTCACCACTCAAGCCTGGCTTGGTATTGGGCGTCGCGGAGTCCCCGcgcgcCTC

AAAGTCGTCCGGCCGAGCTGTCCGTCTCCGAGCGTTGTGACTTTACTGTTTCGCTCTCGAGGTCGGGCgcgcgcCGCTAG

ACTTCTTttttACAGGTTGACCTCGGATCAGGTAGGGATACCCGCTGAACTTAA

>OTU_166

GAAATGCGATAAGTAGTGTGAATTGCAGAATTCAGTGAATCATCGAATCTTTGAACGCACATTGCGCCCCTTGGTATTCC

ATGGGGCATGCCTGTTCGAGCGTCATCTACACCCTCAAGCTCTGCTTGGTGTTGGGCGTCTGTCCCGCCTCGGCgcgcgG

ACTCGCCCCAAAGGCATTGGCAGCGGTCCACGGCCCCTCTCGCGCAGCACATTGCGCTTCTCGAGGCGGCCCGGCCCGCA

TCCACGAAGCCCACATTACCGTCTTTGACCTCGGATCAGGTAGGGATACCCGCTGAACTTAA

>OTU_167

GAAATGCGATAAGTAGTGTGAATTGCAGAATTCAGTGAATCATCGAATCTTTGAACGCACATTGCGCCCCTTGGTATTCC

ATGGGGCATGCCTGTTCGAGCGTCATTTGTACTCTCAAGCCTTGCTTGGTGTTGGGTGCTTGTCTTTGCTTTGCCTAGAC

TCTCCCTAAATACATTGGCAGCCGATatatTGGTTTGAAGCGCAGCACAATTGCAATTCGAACCCGTTTTATTGGCACCC

ACAAAGAATAATAACACTTTTGACCTCGGATCAGGTAGGGATACCCGCTGAACTTAA

>OTU_168

GAAATGCGATAATTAATGTGAATTGCAGAATTCAGTGAATCATCGAGTCTTTGAACGCACATTGCGCCcccTGGTATTCC

GGggggCATGCCTGTCCGAGCGTCATTGCTGCCCTCAAGCCCGGCTTGtgtgtTGGGCCCTGTTCCcccGGGAACAGGCC

CGAAAGGCAGTGGCGGCACCGCGTCCGATCCTCGAGCGTATGGGGCTTTGTCACCCGCTCTGTAGGCCCGGCCGGCGCTT

GCCcccATCAATCTTtttttCAGGTTGACCTCGGATCAGGTAGGGATACCCGCTGAACTTAA

>OTU_169

GAATTGCGATAAGTAATGTGAATTGCAGATTTTCGTGAATCATTGAATTtttGAACGCACATTGCGCCCTTTGGTATTCC

AAAGGGCATGCCTGTTTGAGCGTCATTTCCTTCTCAAAAGAGTTtttttATTCTTTTGGTTGTGAGTGATACTCTTTCCT

TTTACAGGGAAGGGGTTAACTTGAAATTGTTGCCTAGCAAAGAAGAATTTTGATTGAAATTTCTTGTTTATTACTATTAG

GTTTATCCCAACTAGTGATTATTGAGAGTTtttATTACAGAGTCTTTTCACTTGCTATAATACTATTCTATAAGTTTGAC

CTCAAATCAGGTAGGAATACCCGCTGAACTTAA

>OTU_170

GAAATGCGATACTTGGTGTGAATTGCAGAATCCCGTGAACCATCGAGTCTTTGAACGCAAGTTGCGCCCCAAGCCTTCTG

GCCGAGGGCACGTCTGCCTGGGTGTCACAAATCGTCGTCCccccATCCTCTCGAGGATATAGGACGGAAGCTGGTCTCCC

GTgtgtTACCGCACGCGGTTGGCCAAAATCCGAGCTAAGGATGCCAGGAGCGTCTTGACATGCGGTGGTGAATTCAATCT

CCTCGTCATATCGTCGGTCGTTCCGGTCCAAAAGCTCTCGATGACCCAAAGTCCTCAACGCGACCCCAGGTCAGGCGGGA

TCACCCGCTGAGTTTAA

>OTU_171

GAAATGCGATAAGTAGTGTGAATTGCAGAATTCAGTGAATCATCGAATCTTTGAACGCACATTGCGCCCCTTGGTATTCC

ATGGGGCATGCCTGTTCGAGCGTCATTTGTACTCTCAAGCATTGCTTGGTGTTGGGTGTTTGTCCTAACAATGGTTGGGA

CTCGCCTTAAAACAATTGGCAGCCAGTGTATTAGACTTGGAGCGCAGCACAATTTGCGTCTTCTTGCGAATAAACTGGCA

ACCATCAAGCCTATTtttCACGCTTGACCTCGGATCAGGTAGGGATACCCGCTGAACTTAA

>OTU_172

GAAATGCGATAAGTAGTGTGAATTGCAGAATTCAGTGAATCATCGAATCTTTGAACGCACATTGCGCCCCTTGGTATTCC

ATGGGGCATGCCTGTTCGAGCGTCATTTACACCCTCAAGCTCTGCTTGGTGTTGGGCGTCTGTCCCGCTTCGTGCGCGGA

CTCGCCCCAAAGGTATTGGCAGCGGTCTTGCCAGCTTCTCGCGCAGCACATTTGCGTTTCTCGAAGCTATGGCGGATCGG

CGTCCATCAAGCCCACATTACAGTTTGACCTCGGATCAGGTAGGGATACCCGCTGAACTTAA

>OTU_173

GAAATGCGATAAGTAGTGTGAATTGCAGAATTCAGTGAATCATCGAATCTTTGAACGCACATTGCGCCCCTCGGTATTCC

GTGGGGCATGCCTGTTCGAGCGTCATCTACACCCTCAAGCCCTGCTTGGTGTTGGGTGCCTGTCCCGCCCCGcgcgGCGA

CTCACCCCAAATGCATTGGCGGCGGCGCCTTGCCCCTCCCGCGCAGCACATTGCGCTTCTCGAGGCGGCGGGACCGCGTC

CACGAAGCCGACAACCCGTCTTTGACCTCGGATCAGGTAGGGATACCCGCTGAACTTAA

>OTU_174

AAAATGCGATAAGTAGTGTGAATTGCAGAATTCAGTGAATCATCGAATCTTTGAACGCACATTGCGCCCCTTGGTATTCC

ATGGGGCATGCCTGTTCGAGCGTCATTGACACCCTCAAGCTCTGCTTGGTGTTGGGCCGTTTGTCGTTTCCTCGCCcccA

AGCGACACAGCGACACCCCTCAAAGTCATTGGCGGCCTTTCTGCCCGGCTTCCCGCGCAGCAGTTTCGCGCTTCGGAGAC

CACGTGGCAGGCTGGCATCCATCAAGCTGACCCCTTTACGCTTGACCTCGGATCAGGTAGGGATACCCGCTGAACTTAA

>OTU_175

GAAATGCGATAAGTAATGTGAATTGCAGAATTCAGTGAATCATCGAATCTTTGAACGCACATTGCGCCCGCCAGCATTCT

GGCGGGCATGCCTGTTCGAGCGTCATTTCAACCCTCGACTTCCCTTTGGGGAAATCGGCGTTGGGGACCGGCCGTATACC

GCCGGCCCCGAAATGAAGTGGCGGCCCGTCCGCGGCGACCTCTGCGTAGTAATCCAACTCGCACCGGAACCCCGACGTGG

CCACGCCGTAAAACCcccGACTTCTGAACGTTGACCTCGGATCAGGTAGGAATACCCGCTGAACTTAA

>OTU_176

GAATTGCGATAAGTAATGTGAATTGCAGAATTCAGTGAATCATCGAATCTTTGAACGCACCTTGCGCTCCTGGGTATTCC

TAGGAGCATGCCTGTTTGAGTGTCATGAATCCCTCAAATCCCAATGTTTTGTAAAAGAAGCGTTGCTTGGATTTGGTTGT

GGGCCTTTGCAGTCTTTCACGAGTCTGCTGGCCTTAAAGATATTAGCTGGACCTCTTTAATCTCTGAGAAGAGTACTTCC

ATTTGATGTAATAAATTTGCATTGGTGGAAGCTCTACTTAGTACATAAAGCAAGTCTGCTTCTAACCCGGCGCGAGTCTT

TGCACTCAACCCATTATTTATAACTCTGGCCTCAAATCAGGTAGGACTACCCGCTGAACTTAA

>OTU_177

GAAATGCGATAACTAATGTGAATTGCAGAATTCAGTGAATCATCGAGTCTTTGAACGCACATTGCGCCcccTGGTATTCC

GGggggCATGCCTGTCCGAGCGTCATTGCTGCCCTCAAGCCCGGCTTGtgtgtTGGGTCGCGTCCcccTCTCCGGggggA

CGGGGCCCGAAAGGCAGCGGCGGCACCGCGTCCGATCCTCGAGCGTATGGGGCTTTGTCACATGCTCTGGTAGGATTGGC

CGGCGCCTGCCGACGTTTTCCAACCATTCTTTCCAGGTTGACCTCGGATCAGGTAGGGATACCCGCTGAACTTAA

>OTU_178

GAAATGCGATAAGTAATGTGAATTGCAGAATTCAGTGAATCATCGAATCTTTGAACGCACATTGCGCCcccTGGTATTCC

GGgggCATGCCTGTTCGAGCGTCATTTCACCACTCAAGCCTCGCTTGGTATTGGGCATCGCGGTCCGCCGCGTGCCTCAA

ATCGACCGGCTGGGTCTTCTGTCCCCTAAGCGTTTGTGGAAACTATTCGCTAAAGGGTGTTCGGGAGGCTACGCCGTAAA

CAACCCCATTTCTTAAGGTTGACCTCGGATCAGGTAGGGATACCCGCTGAACTTAA

>OTU_179

GAAATGCGATAAGTAGTGTGAATTGCAGAATTCAGTGAATCATCGAATCTTTGAACGCACATTGCGCCCTATGGTATTCC

GTAGGGCATGCCTGTTCGAGCGTCATTCAAACCTTCAAGCTGAGCTTGGTGTTGGGCGTCTGTCCCGCCTCACGGCGCGG

ACTCGCCTCAAATGTATTGGCGGCCGGCACGTTGGCTTCGAGCGCAGCAGAAACGCGAACTCGTTGCCCGACCTGTCGGC

TCCCAGAAGCTAAACCCCATGAATTTGACCTCGGATCAGGTAGGGATACCCGCTGAACTTAA

>OTU_180

AAAGTGCGATAACTAGTGTGAATTGCATATTCAGTGAATCATCGAGTCTTTGAACGCAACTTGCGCTCATTGGTATTCCA

ATGAGCACGCCTGTTTCAGTATCAAAACAAACCCTCTATCCAACATTTTGTTGAATAGGAATACTGAGAGTCTCTTGATC

TATTCTGATCTTGAACCTCTTGAAATGTACAAAGGCCTGATCTTGTTTAAATGCCTGAACTTttttttAATATAAAGAGA

AGCTCTTGCGGTAAACTGTGCTGGGGCCTCCCAAATAATACTTtttttAAATTTGATCTGAAATCAGGCGGGATTACCCG

CTGAACTTAA

>OTU_181

GAAATGCGATAAGTAATGTGAATTGCAGAATTCAGTGAATCATCGAATCTTTGAACGCACATTGCGCCCCTTGGTATTCC

GGAGGGCATGCCTGTTCGAGCGTCATTACACCACTCAAGCTATGCTTGGTATTGGGCGTCGTCCTTAGTTGGGCgcgcCT

TAAAGACCTCGGCGAGGCCAGCTCCGGCTTTAGGCGCTAGTAGAATTTATTCGAACGTCTGTCAAAGGAGAGGAACTCTG

CCGACTAGAAACCTTTATTTTCTAGGTTGACCTCGGATCAGGTAGGGATACCCGCTGAACTTAA

>OTU_182

GAAATGCGATACGTAATGTGAATTGCAGAATTCAGTGAATCATCGAATCTTTGAACGCACCTTGCACTCTTTGGTATTCC

GAAGAGTATGTCTGTTTGAGTGTCATGAAACTCTCAACCcccTCATTTTGTAATGAAGTGAGTCGTGGGCTTGGATTATG

GCTGTCTGTCGGCTTCATTGCCGGCTCAGCTGAAATACACGAGTAACCCAGTTTGAAaaaCTGACGGCTTGACTCGGCGT

AATAATTATTATCGCTGAGGACGTCTAACCTTCAAATCGTTAGTGGTGCTTCTAATGCAAATCATTTTAAGATTTAGACC

TCAAATCAGTCAGGATTACCCGCTGAACTTAA

>OTU_183

GAAATGCGATACGTAGTGTGAATTGCAGAATTCAGTGAATCATCGAATCTTTGAACGCACATTGCGCCCATTGGTATTCC

AATGGGCATGCCTGTTCGAGCGTCATTTGTACCCTCAAGCTTTGCTTGGTGTTGGGCGTTTGTCCTGCGGGACTCGCCTT

AAAACGATTGGCAGCCGGCACACTGGTTTGGAGCGCAGCACAAATTGCGGTCCAGCCATGAATGTCGGCGTCCATGAAGC

CcccATTTCACTTTTGACCTCGGATCAGGTAGGGATACCCGCTGAACTTAA

>OTU_184

GAAATGCGATAAGTAGTGTGAATTGCAGAATTCAGTGAATCATCGAATCTTTGAACGCACATTGCGCCCTTTGGTATTCC

TTAGGGCATGCCTGTTCGAGCGTCATTTAAACCTTCAAGCCCTGCTTGGTGTTGGGTGCCTGTCCCGCCcccGcgcgTGG

ACTCACCTCAAATCCATTGGCGGCCccccGCATGGCCACGAGCGCAGCAGAAACGCAAACTCGTGTGCCGGACCGGGCGG

CTCCCAGAAGCTACACTCACCATTTTGACCTCGGATCAGGTAGGGATACCCGCTGAACTTAA

>OTU_185

GAAATGCGATAAGTAATGTGAATTGCAGAATTCAGTGAATCATCGAATCTTTGAACGCACATTGCGCCCTCTGGTATTCC

GGggggCATGCCCGTTCGAGCGTCATTACACCACTCAAGCCTCGCTTGGTCTTGGGCGCCGCGGCCTGCCGcgcgcCTCA

AAGTCACCGGCCGGGTGACCCGTCTCGGAGCGTCGTAGCCATTGTGTTTCGCTCGCAGGGATCGGGCCGCCGTCGGCGCC

GCTAAACGTCTTTTATCAGGTTGACCTCGGATCGGGTAGGGATACCCGCTGAACTTAA

>OTU_186

GAAACGCGATAGGTAATGTGAATTGCAGAATTCAGTGAATCATCGAATCTTTGAACGCATCTTGCGCTCCTTGGTATTCC

TCGGAGCATGCCTGTTTGAGTGTCGTGAATTTCTCCAAaaaaaTGGTTttttGTTATGAAaaaCTTttttGCGGGTCCTT

GGGCTTGGTGATGGCATAGCCTCTTTGTATCACCTTGCCTTAAATGTATGAGTGGATGAGTGTCAGCCATAGATTtttGT

TAAGGCAAACCCATAAaaaTCTTGTGCTTTTGTCATCTGCTTCCAAACAAACACGCGTAATATACacacTTGtgtgCATA

TTCTTTTCTGGCCTCAAATCAGGTAAGATTACCCGCTGAACTTAA

>OTU_187

GAAATGCGATAAGTAATGTGAATTGCAGAATTCAGTGAATCATCGAATCTTTGAACGCACATTGCGCCCATTAGTATTCT

AGTGGGCATGCCTGTTCGAGCGTCATTTCAACCCTTAAGCCCCAGTTGCTTAGTGTCGGGAGACTGCCGTAAAACGCAGC

TCCTCAGAGTTAGCTGGCAGAGTTGGTTCCAATCTCTGGGCGTAGTAAATCTTTATCTCGTCTCTTGCAGTTGGGCCGGC

GCTCGCCATTAAACACCCCAAATTtttCTATGGTTGACCTCGGATCAGGTAGGAATACCCGCTGAACTTAA

>OTU_188

GAAATGCGATAAGTAGTGTGAATTGCAGAATTCAGTGAATCATCGAATCTTTGAACGCACATTGCGCCCCTTGGTATTCC

ATGGGGCATGCCTGTTCGAGCGTCATTTGTACCCTCAAGCTATGCTTGGTGTTGGGTGTTTGTCCTTTGCTTTATTGCTT

GGACTCGCCTTAAAATAATTGGCAGCCATTGTTTTGGTAGAAAGCGCAGCACATTTTGCAATTTCCTCCAAGAGTATTAG

CGACCAACAAGCCTTtttAACACTTTTGACCTCGGATCAGGTAGGGATACCCGCTGAACTTAA

>OTU_189

GAAATGCGATAAGTAATGTGAATTGCAGAATTCAGTGAATCATCGAATCTTTGAACGCACCTTGCACTCCTTGGTATTCC

GAGGAGTACGCCTGTTCGAGTGTTATGAAGCTCTCAAAACGCAATTGGCTTAAAACCTGATTGTTGTTTTGGATTTGAAC

TTTGCCGTGTTATAACGGCTGGTTTTAAAAGTATTAGCTGGTACTAGAACGAATTGGTTCTACTCAACGTGATAATTATC

TGATCGTTGAGGACATTCCTTTCACGAGGAATGGCCATAGTAAATTCAATGGACTGCTTCTAATCTGGCTTTAATTCTTT

ATTGGATTAACCACTTTTCAAATCTGACCTCGAATCAGGTGGGACTACCCGCTGAACTTAA

>OTU_190

GAATTGCGATAAGTAATGTGAATTGCAGAATTCAGTGAATCATCGAATCTTTGAACGCACCTTGCACTCTTTGGTATTCC

GAAGAGTATGTCTGTTTGAGTGTCATGAAACTCTCAACCcccTTGTTTTGTAATGAAACGAGCGTGGGCTTGGATTATGG

CTGTTTGTCGGCGTCATTGCCGGCTCAGCTGAAATACACGAGCAACCCATTTGAAATAGGCGGTTTGACTCGGCGTAATA

ATTATTTCGCTGAGGACGTCTTCTTTAAAGTTGGTGGTGCTTCTAATGCGCTTTTAATTAAGCATCTTAAGCTTTAGACC

TCAAATCAGTCAGGACTACCCGCTGAACTTAA

>OTU_191

GAAATGCGATAAGTAATGTGAATTGCAGAATTCAGTGAATCATCGAATCTTTGAACGCACATTGCGCCCTCTGGTATTCC

AGAGGGCATGCCTGTTCGAGCGTCATTTCAACCCTCAAGCCCTGCTTGGTGTTGGGGCATTACCTGAGGCCGCCccccGG

GCGGGCCGGGTAAGCCCTGAAATTTAGTGGCGAGCTCGCCAGGACTCCGAGCGCAGTAGTAAAACCCTCGCTTTGGACTG

TACTGGCGCGGCCCTGCCGTAAAACCcccAACTTCTGAAAATTTGACCTCGGATCAGGTAGGAATACCCGCTGAACTTAA

>OTU_192

AAAGTGCGAAAAGTGTTGCGATTTGCACGAATCTGTGAGTCATCTAATTtttGAACGCGAATGGCACTGTTACGTAAGTA

GCAGTATGTCTGTTTGAGAATCGCAAAACAAGATCAACTCTAGTTGAGAGTTGGGATTGACCTTATGTGCCTTTGGGTAC

ATTTGGTTTAAATAAATCGAGTTGATGAGCTTCCCAATACGGCTCAATGtgtgCTAGCGAGACAGTCGTCCCGATCTCAC

ATTTCGGCCGTGTTGCAGAGTTCTGACCACAATTCGCTAAACTTATAATCAAATACGATCTCAAATCAGGCAAGATTACC

CGCTGAACTTAA

>OTU_193

TCATTAACGTTTACTATGGCGGTAACCTTTACCAAGATTTGCCAAGTCAATACCAAGGCGATTTGATCCAGCACTCTCAG

ACTTCCCGAGGCTTTATCCCAATTCACTCTGTGGACATCACGGCCGATAGTTTCACGGGCCAAGTCTTTGGTGCAACTGC

CTTTGTCAATTCTCGCCACCATCAAGCCATCCAGCAAGTCGGAGCCGGTTTGACCGTCACCGCTAAAGCCAAAGATGGCG

TGGTTGAAGCGATTGAAAACTTGGACGATTCGGTCATTGGTGTTCAATGGCACCCTGAAAACTTGTG

>OTU_194

GAAATGCGATAAGTAATGTGAATTGCAGAATTCAGTGAATCATCGAATCTTTGAACGCACATTGCGCCcccTGGTATTCC

GGggggCATGCCTGTTCGAGCGTCATTTCAACCAAACCAGCCCCGCTGGGTGTTGGGCGTCGCGGTCCGCCGcgcgcCTC

AAAGTCTTCGGCGGAAGCCGCCCGTTCCTCTGCGTGATGCATCGTCGTCGCTCGGGACACGGgggTGAGCGCCCGGAAAA

CCGTCGGCGGAGACGCCGACTTCAGGTTGACCTCGGATCAGGTAGGGATACCCGCTGAACTTAA

>OTU_195

GAAATGCGATAAGTAGTGTGAATTGCAGAATTCAGTGAATCATCGAATCTTTGAACGCACATTGCGCCTCCTGGTATTCC

GGGAGGCATGCCTGTTCGAGCGTCATTAAaaaCCACTCAAGCTCTTTTGCTTGGTCATGGAAGATGAGTGAGCTTGTCtc

tctcCCTTTCGAAATTCAAAGGCGGAAAGCCCCATGTGCACCGGCGTAGTAAGTTTTCTTTCGCTTTGGCCATGAGGTGA

TCCTGCCCCAAACCcccAATTtttCTAGGTTGACCTCGGATCAGGTAGGGATACCCGCTGAACTTAA

>OTU_196

GAAATGCGATAAGTAATGCGAATTGCAGAATTTCCGTGAGTCATCGAATCTTTGAACGCACATTGCGCCCACTGGTATTC

CGGTGGGCATGCCTGTTCGAGCGTCATTATCCTCCCTCAAACCCCGGGTTTGGTGTTGGACCGAAGTTGtgtgAACAACT

GGTCTAAaaaaCAATGACGGCGTCCGTGGGACCTCGGTGCAACGAGCTTTTAGGAGCACGCGCCGAGTTGCAAGGACCTT

CCGGGCCGGTCTCCTTTTACTATTTTACAAGGTTGACCTCGGATCAGGTAGGAATACCCGCTGAACTTAA

>OTU_197

GAATTGCGATAAGTAATGTGAATTGCAGATTTTCGTGAATCATCGAATCTTTGAACGCATATTGCGCTCTATAGTATTCT

ATAGAGCATGCCTGTTTGAGCGTCATTTCtctctTAAACCTTTGGGTTTAGTATTGAAGGTTGTGTTAGCTTCTGCTAAC

TCCTTTGAAATGACTTGGCAATTGATTGAGTTTTCCATatatTTGCTTAAGGATTTAATATTAGGTTCTACCAACTTATT

AAATACCCTTTTGCGAAGGACTTACTCGTGTATCAAGGCCTTATAACTTTGTCATTAATTTTGACCTCAAATCAGGTAAG

GATACCCGCTGAACTTAA

>OTU_198

GAAATGCGATAACTAATGTGAATTGCAGAATTCAGTGAATCATCGAGTCTTTGAACGCACATTGCGCCCTCTGGTATTCC

GGAGGGCATGCCTGTCCGAGCGTCATTGCTGCCCTCAAGCACGGCTTGtgtgtTGGGCCcccGCCccccccGCGCTGGgg

gggCGGGCCCGAAAGGCAGCGGCGGCACCGCGTCCGGTCCTCGAGCGTATGGGGCTTCGTCACCCGCTCTTGTAGGCCCG

GCCGGCGCCAGCCGACCccccTCAATCTATTttttCAGGTTGACCTCGGATCAGGTAGGGATACCCGCTGAACTTAA

>OTU_199

GAAATGCGATACTTGGTGTGAATTGCAGAATCCCGTGAACCATCGAGTCTTTGAACGCAAGTTGCGCCCGAAGCCATTAG

GCCGAGGGCACGCCTGCCTGGGTGTCAcacaTCGTTTCCCCAACGCAAACATGTAACAATGTTGCTGCGCGGGGTGTATG

CTGACCTCCCGCGAGCACCCGCCTCGTGGTTGGTTGAAATCTGGGTTCATGGCCGACTTCGCCGTGATAAAATGGTGGAT

GAGCCACGCTCGAGACCAATCACGTGCGAGCCGGTCAGTTCTGGACCCATCGACGACCCTTTGCGTGCACGCACGCTCCC

AACGAGACCTCAGGTCAGGCGGGGCTACCCGCTGAGTTTAA

>OTU_200

GAAATGCGATAAGTAGTGTGAATTGCAGAATTCAGTGAATCATCGAATCTTTGAACGCACATTGCGCCCTATGGTATTCC

GTAGGGCATGCCTGTTCGAGCGTCATTCAACCCTTCAAGCTCTGCTTGGTGTTGGGCGTCTGTCCCCGCCCCTCCCGGCG

GCGGACTCGCCTCAAATCTATTGGCGGCCGGCACGTTGGCTTCGAGCGCAGCAGAAACGCGAACTCGAGGCCCGGCGGAT

CGGCTCCCAGAAGCGAACCcccccATGAATTTGACCTCGGATCAGGTAGGGATACCCGCTGAACTTAA

>OTU_201

GAAATGCGATAAGTAATGTGAATTGCAGAATTCAGTGAATCATCGAATCTTTGAACGCACCTTGCGCTCCTTGGTATTCC

GAGGAGCATGCCTGTTTGAGTGTCATTAAATTCTCAACTTCATCAGTTTTGTTACAAGACTGCGTGAGGCTTGGATATGG

gggCTTGTGCAGAATGTGTCAAAGCGGACTGCTCCCCTTAAATGAATTAGCGAGTTCTAACTGGGCTCCGTCTACTGGTG

TGATAATTATCTACGCCATGGATTGGGCTTAGACTCGCTTCTAATCGTCTGCAAGGACAATCTTTTGACAATTTGACCTC

AAATCAGGTAGGACTACCCGCTGAACTTAA

>OTU_202

GAAATGCGATAAGTAGTGTGAATTGCAGAATTCAGTGAATCATCGAATCTTTGAACGCACATTGCGCCCCTTGGTATTCC

ATGGGGCATGCCTGTTCGAGCGTCATTTGTACCCTCAAGCTTTGCTTGGTGTTGGGCGTTTTGTCTTTTCACAAAGACTC

GCCTCAAAGTAATTGGCAGCCAGTGTTTTGGTAGTAAGCGCAGCACATTTTGCGTCTTAGTCCCTTGACAGCGGCATCCA

CAAAGCCTCTTTCTCACTTTTGACCTCGGATCAGGTAGGGATACCCGCTGAACTTAA

>OTU_203

GAAATGCGATACGTAATATGAATTGCAGATATTCGTGAATCATCGAATCTTTGAACGCACATTGCGCCCTCTGGTATTCC

GGAGGGCATGCCTGTTTGAGCGTCGTTTCTCCCTCAAACCGCTGGGTTTGGTGTTGAGCAATACGACTTGGGTTTGCTTG

AAAGACGGTAGTGGTAAGGCGGGATCGCTTTGACAATGGCTTAGGTCTAACCAAaaaCATTGCTTGCGGCGGTAACGTCC

ACCACGTATATCTTCAAACTTTGACCTCAAATCAGGTAGGACTACCCGCTGAACTTAA

>OTU_204

GAAATGCGATACGTAATGTGAATTGCAGAATTCAGTGAATCATCGAATCTTTGAACGCACCTTGCACTCTTTGGTATTCC

GAAGAGTATGTCTGTTTGAGTGTCATGAAACTCTCAACCcccTTGTTTTGTAACGAAGCAGGCGTGGGCTTGGATTATGG

CTGTCTGCTGGACTCTTTAATTAGAGATCGGCTCGGCTGAAATACACGAGTAACCCAGTTTGAACATTAACGGCTTGACT

CGGCGTAATAGCTTTATTTCGCTGAGGACGTTTCATATCAAGATAGTGGTGCTTCTAATGCGTCGTAGCGTCAGCTGCAC

AACCTTTAAGCTTTAGACCTCAAATCAGTCAGGACTACCCGCTGAACTTAA

>OTU_205

GAAATGCGATAAGTAATGTGAATTGCAGAATTCAGTGAATCATCGAATCTTTGAACGCACATTGCGCCCCGTGGTATTCC

GCGGGGCATGCCTGTTCGAGCGTCATTTCACCACTCGAGTCTGACTCGGTATTGGGCGTCGCGCACTGCCGcgcgTCTCA

AAGTCTCCGGCTGGGCGGACCCGTCCCCGAGCGTTGTGGCATCACAGTTCTCGCTAGGGAGTCGCGGGTCGGCTTGCGGC

CGTTAAATACACCCATCAAAGGTTGACCTCGGATCAGGTAGGGATACCCGCTGAACTTAA

>OTU_206

GAAATGCGATAAGTAGTGTGAATTGCAGAATTCAGTGAATCATCGAATCTTTGAACGCACATTGCGCCCCTTGGTATTCC

ATGGGGCATGCCTGTTCGAGCGTCATTTGTACTCTCAAGCCTTGCTTGGTGTTGGGTGATTGTCTTTGCTATGCCTAGAC

TCGCCTTAAATTTATTGGCAGCCGATATGTTGGTTTGAAGCGCAGCACAATTTGCGGTTTGAGCTTACTATGTTAGCAAC

CATGAAGCCCTTtttAACACTTTTGACCTCGGATCAGGTAGGGATACCCGCTGAACTTAA

>OTU_207

GAAATGCGATAAGTAATGTGAATTGCAGAATTCAGTGAATCATCGAATCTTTGAACGCACCTTGCGCTCCTTGGTATTCC

GAGGAGCATGCCTGTTTGAGTGTCATTAAATTCTCAACTGTACAAGTTtttGAAACCTGTATAGCTTGGATCTTGGGATC

TGCGGGCTTTCACAAAGTCAGCTATCCTTAAATGCATTAGCAGAGCTTTTGCCGCTAACCTCTGGTGTGATAATTATCTA

TGCCATTGAGAGGCGACATAATGAAGCTAAGCTTCCAATCGTCCTTTAGGACAATACTTGACAATCTGACCTCAAATCAG

GTAGGACTACCCGCTGAACTTAA

>OTU_208

GAAATGCGATAAGTAGTGTGAATTGCAGAATTCCGTGAATCATCGAATCTTTGAACGCACATTGCGCCTCCTGGTACTCC

GGGAGGCATGCCTGTCCGAGCGTCATTATAACCcccTCAAGCTTCATTGCTTGGTCTTGGAGCTGGAGCGTCCTCAGCGG

CGGTCCGTTCCGAATTCCAATGGCAGACGACGGTGTCAACGTCAGCGTAGTGAGCATTTTGCATCGCGAAGCGCAGTCAC

CCGTCCCGCCGAACCAAACCCTTCTTTATAATGTTGACCTCGGATCAGGTAGGGATACCCGCTGAACTTAA

>OTU_209

GAAATGCGATAAGTAATGTGAATTGCAGAATTCAGTGAATCATCGAATCTTTGAACGCACATTGCGCCCCGTGGTATTCC

GCGGGGCATGCCTGTTCGAGCGTCATTTCACCACTCAAGCCTGGCTTGGTATTGGGCGCCGCGTGTTCGCACGcgcgcCT

TAAAGTCTTTCCGGCTGAGTCGTCCGTCTCCCAGCGTTGTGATACATTTTCGCTTCGGAGTGCGGATGACCGCGGCCGTT

AAATCTTCATTCAAGGTTGACCTCGGATCAGGTAGGGATACCCGCTGAACTTAA

>OTU_210

GAAATGCGATAAGTAATGTGAATTGCAGAATTCAGTGAATCATCGAATCTTTGAACGCACATTGCGCCCCGTGGTATTCC

GCGGGGCATGCCTGTTCGAGCGTCATTACACCACTCAAGCCTCGCTTGGTATTGGGCGACGCGGTACGCCGcgcgcCTCA

AATCTCCCGGCTGGTTCGAATCGTCCCTCAGCGTTGTGGAAACTATTCGCTAAAGGTGCGCGTTTCGGTCACGCCGTCAA

ACAAACCAATTTATTAAAGGTTGACCTCGGATCAGGTAGGGATACCCGCTGAACTTAA

>OTU_211

GAAATGCGATACGTAATGTGAATTGCAGAATTCCGTGAATCATCGAATCTTTGAACGCACATTGCGCCCTCTGGTATTCC

GGggggCATGCCTGTTTGAGCGTCATTTCCTTCTCAAACACGTTGTGTTTGGTAGTGAGTGATACTCTCGTTtttGAGTT

AACTTGAAATTGTAGGCCATATCAGTATGTGGGACACGAGCGCAAGCTTCTCTATTAATCTGCTGCTCGTTTGCGCGAGC

GGCGGgggTTAATACTGTATTAGGTTTTACCAACTCGGTGTTGATCTAGGGAGGGATAAGTGAGTGTTTTGTGCGTGCTG

GGCAGACAGACGTCTTTAAGTTTGACCTCAAATCAGGTAGGGTTACCCGCTGAACTTAA

>OTU_212

GAAATGCGATAAGTAATGTGAATTGCAGAATTCAGTGAATCATCGAATCTTTGAACGCACATTGCGCCCGCCAGTACTCT

GGCGGGCATGCCTGTTCGAGCGTCATTTCAACCCTCAAGCCCCAGCGGTTTGGTGTTGGGCGTCGGCCGTCCTCTGCGGC

GGCCGTGCCCCAAATACAGTGGCGGACTCGCTGTTGCCTCCTCTGCGTAGTAGTAATATCTCGCACCGGAAAAGCAGCGT

GCCCACGCCGTAAAACAACCCAATTTTCTGAATGTTGACCTCGGATCAGGTAGGAATACCCGCTGAACTTAA

>OTU_213

GAATTGCGATAAGTAATGTGAATTGCAGATACTCGTGAATCATTGAATTtttGAACGCACATTGCGCCCTTGAGCATTCT

CAAGGGCATGCCTGTTTGAGCGTCATTTCCTTCTCAAAAGATAATTtttATTtttGTTTGTGGCGATACTCAGGGTTAGC

TTGAAATTGGAGACTGTTTCAGTCTTttttAATTCAACACTTAGCTTCTTTGGAGACGCTGTTCTCGCTGTGATGTATTT

ATGGATTTATTCGTTTTACTTTACAAGGGAAATGGTAATGTACCTTAGGCAAAGGGTTGCTTTTAATATTCATCAAGTTT

GACCTCAAATCAGGTAGGATTACCCGCTGAACTTAA

>OTU_214

GAAATGCGATAAGTAATGTGAATTGCAGAATTCAGTGAATCATCGAATCTTTGAACGCACATTGCGCCCCGTGGTATTCC

GCGGGGCATGCCTGTTCGAGCGTCATTTCACCACTCAAGCCTCGCTTGGTATTGGGCGCCGCGTGATTTCCACGcgcgcC

TCAAAGTCTTTCCGGCTGAGTCGTCCGTCTCCCAGCGTTGTGATACATTTTCGCTTCGGAGTGCGGGTGCgcgcCGTAAT

CTCCATCAAAGGTTGACCTCGGATCAGGTAGGGATACCCGCTGAACTTAA

>OTU_215

GAAATGCGATAAGTAGTGTGAATTGCAGAATTCAGTGAATCATCGAATCTTTGAACGCACATTGCGCCCCTTGGTATTCC

ATGGGGCATGCCTGTTCGAGCGTCATTTGTACCCTCAAGCTATGCTTGGTGTTGGGTGATTGTCCAGCCTGTATGGCCAG

GACTCGCCTTAAAGGAATTGGCAGCCAGTGTTTTGGTAGAAAGCGCAGCACATTTTGCGATTTACTCCAATAATATTAGC

AACCATAAAGCTCATTTATCACTTTTGACCTCGGATCAGGTAGGGATACCCGCTGAACTTAA

>OTU_216

GAAATGCGATAAGTAGTGTGAATTGCAGAATTCAGTGAATCATCGAATCTTTGAACGCACATTGCGCCCCTTGGTATTCC

ATGGGGCATGCCTGTTCGAGCGTCATTTGTACCCTCAAGCTCTGCTTGGTGTTGGGTGTTTGTCCCGCTTTTCTGCGTGG

ACTCGCCTTAAAGCAATTGGCAGCCGGCATAGTGGCCTGGAGCGCAGCACAATTTGCGCCTCTTGTCATGAATGTTTGGC

ATCCATCAAGCCCAAATTATTTGCTCTTGACCTCGGATCAGGTAGGGATACCCGCTGAACTTAA

>OTU_217

GAAATGCGATAAGTAATGTGAATTGCAGAATTCAGTGAATCATCGAATCTTTGAACGCACATTGCGCCCTCTGGTATTCC

AGAGGGCATGCCTGTTCGAGCGTCATTTCAACCCTCAAGCCCAGCTTGGTGTTGGGGCATTACCTTCCCTCACGGGAGGG

TAAGCCCTGAAATTTAGTGGCGAGCTCGCCAGGACTCCGAGCGCAGTAGTAAAACCCTCGCTCTGGACTGTACTGGCGCG

GCCCTGCCGTAAAACCcccAACTTCTGAAAATTTGACCTCGGATCAGGTAGGAATACCCGCTGAACTTAA

>OTU_218

GAAATGCGATAAGTAATGTGAATTGCAGAATTCAGTGAATCATCGAATCTTTGAACGCACATTGCGCCCATTAGTATTCT

AGTGGGCATGCCTGTTCGAGCGTCATTTCAACCCTTAAGCCTTAGTTGCTTAGTGTTGGGAGCCTACGGCCTGCCGTAGC

TCCTCAAAGTTAGTGGCGGAGTCGGTCTCACACTCTAGACGTAGTAGATTTTATTTCTCGTCTGTAGTTGGGCCGGTCCC

CTGCCGTAAAACACCcccccAATATCAAAGGTTGACCTCGGATCAGGTAGGAATACCCGCTGAACTTAA

>OTU_219

GAAATGCGATACGTAGTGTGAATTGCAGAATTCAGTGAATCATCGAATCTTTGAACGCACATTGCGCCTCCTGGTATTCC

GGGAGGCATGCCTGTTCGAGCGTCATCAAaaaCCTCAGTCTCTGATTTATCATTGATTGGTCTTGCATCGGATGCCAATG

GCGTCCCCTGCGAAATCCAATGGCGAAGAGCCACGCAGCCAAAGCGTAGTATAATAAACCTCGTAATGGATGtgtgGGTG

CTTCTGCCGTAACCcccAAATTCTTAGTTtttGACCTCGGATCAGGTAGGGATACCCGCTGAACTTAA

>OTU_220

GAAATGCGATAAGTAATGTGAATTGCAGAATTCAGTGAATCATCGAATCTTTGAACGCACATTGCGCCCTCTGGTATTCC

GGAGGGCATGCCTGTTCGAGCGTCATTTCAACCCTCAAGCCTGGCTTGGTGTTGGGGCACTGCGTCtctcGCGGGATGCA

GGCCCTGAAATTCATTGGCGAGCTCGCCAGGACCCCGAGCGCAGTAGTTAAACCCTCGCTCTGGAAGGCCCTGGCGTGCC

CTGCCGTTAAACCcccAACTTCTGAAAATTTGACCTCGGATCAGGTAGGAATACCCGCTGAACTTAA

>OTU_221

GAAATGCGATAAGTAATGTGAATTGCAGAATTCAGTGAATCATCGAATCTTTGAACGCACATTGCGCCCATTAGTATTCT

AGTGGGCATGCCTGTTCGAGCGTCATTTCAACCCTTAAGCCTAGCTTAGTGTTGGGAATCTACTGTATTGTAGTTCCTGA

AATACAACGGCGGATCTGTAATATCCTCTGAGCGTAGTAATTttttCTCGCTTTGGTTAGGTGTTGCAGCTCTCAGCCGC

TAAACCccccAATTTTAATGGTTGACCTCGGATCAGGTAGGAATACCCGCTGAACTTAA

>OTU_222

GAAATGCGATAAGTAATGTGAATTGCAGAATTCAGTGAATCATCGAATCTTTGAACGCACCTTGCGCCCCTTGGTATTCC

GAGGGGCATGCCTGTTTGAGTGTCATTAAATTCTCAACCTTACTAGCTTTTGCGAAGTAATGGCTTGGACTTGGgggTCT

TTTGCTGGTTTCGAAAGAGATCTGCTCCCCTTAAATGCATTAGCCGGTGCCCCGCGTGGACCGTCTATTGGTGTGATAAT

TATCTACGCCGTTAGATGTCTGCTATTAAATGGGATGCGCTGCTTCTAATCGTCCTCTAGGACAATTATTGACCATTTGA

CCTCAAATCAGGTAGGACTACCCGCTGAACTTAA

>OTU_223

GAATTGCGATAAGTAATGTGAATTGCAGATACTCGTGAATCATTGAATTtttGAACGCACATTGCGCCCTTGAGCATTCT

CAGGGGCATGCCTGTTTGAGCGTCATTTCCTTCTCAAAAGATAATTTATTATTttttGGTTGTGGGCGATACTCAGGGTT

AGCTTGAAATTGGAGACTGTTTCATGTCTTttttAATTCTAACACTTAGCTTCTTTGGAGACGCTGTTCTCGCTGTGATG

TATTTATGGATTTATTCGTTtttACTTTACAAGGGAAATGGTAACGTACCTTAGCAAAGGGTTGCTTTTAATATTCATCA

AGTTTGACCTCAAATCAGGTAGGATTACCCGCTGAACTTAA

>OTU_224

GAAATGCGATAAGTAATGTGAATTGCAGAATTCAGTGAATCATCGAATCTTTGAACGCACCTTGCGCCTCTTGGTATTCC

GAGAGGCATGCCTGTTTGAGTGTCTTATAAAATCAATCCCCACGGGTTTCTTGACCTGGCGGTGGACTTGGAGTTGGGCG

TCTGCCGGTCACACGGCTCGCCTTAAAGACCTTAGTGGGATCATCGGCCTATGGCTTGACGTAATAAGTTTCGTCTCGTC

CGGCCCCTGGAGCCCGCTCACAACCCGCCCTCGGGCAACTACTTTATGCTAGACCTCAAATCAGGTAGGACTACCCGCTG

AACTTAA

>OTU_225

GAAATGCGATAAGTAGTGTGAATTGCAGATTTCAGTGAATCATCGAATCTTTGAACGCACATTGCGCCCTTTGGTATTCC

GAAGGGCATGCCTGTCTGAGCGTCAATAAATCAATCAAACTCTTGTTTGGTATTGGGAGAAGTGGTCTATACCACTCTTC

TTAAAGCTATTGGCGACACTATTTTCAGCTTGTAAGATGTAGTAAGAAATTCCTCGTCGAAGCAACTGGAGTGGTTTGTC

TGCCAACTGAACCGATTTATTTTACTGACTTGACCTCAGATCAGGTAGGGATACCcccTGAACTTAA

>OTU_226

GAAATGCGATAAGTAGTGTGAATTGCAGAATTCAGTGAATCATCGAATCTTTGAACGCACATTGCGCCCCTTGGTATTCC

ATGGGGCATGCCTGTTCGAGCGTCATTTGTACCCTCAAGCTATGCTTGGTGTTGGGTGATTGTCCACTAGGACTCGCCTT

AAAGACATTGGCAGCCAGTATTTTGGTAGAAAAGCGCAGCACATTTTGCGATTTTACTCCAGTAATATTAGCACCCATTA

AGCTCAATTATCACTTTTGACCTCGGATCAGGTAGGGATACCCGCTGAACTTAA

>OTU_227

GAAATGCGATACGTAGTGTGAATTGCAGAATTCAGTGAATCATCGAATCTTTGAACGCACATTGCGCCCTTTGGTATTCC

AAAGGGCATGCCTGTTCGAGCGTCATTTGTACCCTCAAGCTTTGCTTGGTGTTGGGCGTCTTtttGTCtctcCCCTTGTT

GGgggAGACTCGCCTTAAAACGATTGGCAGCCGACCTACTGGTTTTCGGAGCGCAGCACAAATTTGCGCCTTCCAATCCA

CGGGGCGGCATCCAGCAAGCCTTTGTTTTCTATAACAAATCCACATTTTGACCTCGGATCAGGTAGGGATACCCGCTGAA

CTTAA

>OTU_228

GAAATGCGATAAGTAGTGTGAATTGCAGAATTCAGTGAATCATCGAATCTTTGAACGCACATTGCGCCCCTTGGTATTCC

ATGGGGCATGCCTGTTCGAGCGTCATTTGTACCCTCAAGCTTTGCTTGGTGTTGGGCGTCTTTGTCTCGCGTTGTCGCGT

GGACTCGCCTGAAAGCGATTGGCAGCCGGCATATTTGGCCTTGGAGCGCAGCACATTTTGCGCTTCTGGTCACGAATGTC

AGCGTCCATCAAGCCCTCTTTTACATTTGCGATTGACCTCGGATCAGGTAGGGATACCCGCTGAACTTAA

>OTU_229

GAAATGCGATAAGTAATGTGAATTGCAGAATTCAGTGAATCATCGAATCTTTGAACGCACATTGCGCCCCTTGGTATTCC

GAGGGGCATGCCTGTTCGAGCGTCATTACACCACTCAAGCTATGCTTGGTATTGGGTGTCGTCCTTAGTTGGGCgcgcCT

TAAAGACCTCGGCGAGGCCTCACCGGCTTTAGGCGTAGTAGAATTTATTCGAACGTCTGTCAAAGGAGAGGACTTCTGCC

GATTGAAACCTTTTATTttttCTAGGTGACCTCGGATCAGGTAGGGATACCCGCTGAACTTAA

>OTU_230

GAAATGCGATAAGTAATGTGAATTGCAGAATTCAGTGAATCATCGAATCTTTGAACGCACATTGCGCCCCTTGGTATTCC

GAGGGGCATGCCTGTTCGAGCGTCATTACAACCCTCAAGCCTCGCTTGGTATTGGGCCCTGCCGTCCGCGGCTGGCCTTA

AaaaCAGTGGCGGTGTGCCGGACTCCTCAGCGTAGTAACTTTCTCGCTACAGGACTTCGGCTCCCTGGCCAGAACCCCAT

CTTCAGTTTGACCTCGGATCAGGTAGGGATACCCGCTGAACTTAA

>OTU_231

GAAATGCGATAAGTAATGTGAATTGCAGAATTCAGTGAATCATCGAATCTTTGAACGCACCTTGCGCCTTTTGGTATTCC

GAAAGGCATGCCTGTTTGAGTGTCATGAAATCTCAATCCccccGGGTTTTCTGAACCCGAGGTGGACTTGGACATGGGTG

TCTGCCGTCTCGTACGGCTCGCCTTAAATGACTCAGTGGGATCTTCAGCATCCGTGGCAGACGTAATAAGTTTCGTCTCG

TCCCTTGCTGTGAGGACCGCTCATAACCTGCCATCGCGCACCACTTTTGACTCTGACCTCAAATCAGGTAGGACTACCCG

CTGAACTTAA

>OTU_232

GAAATGCGATAACTAGTGTGAATTGCAGAATTCCGTGAATCATCGAGTCTTTGAACGCACATTGCGCCcccTGGTATTCC

GGggggCATGCCTGTCCGAGCGTCATTGCTGCCCATCAAGCACGGCTTGtgtgtTGGGTCGTCGTCCCCTCTCCGGgggg

gACGGGCCCCAAAGGCAGCGGCGGCACCGCGTCCGATCCTCGAGCGTATGGGGCTTTGTCACCCGCTCTGTAGGCCCGGC

CGGCGCTTGCCGAACGCAAATCAATCTTtttCCAGGTTGACCTCGGATCAGGTAGGGATACCCGCTGAACTTAA

>OTU_233

GAAATGCGAAAAGTAATGTGAATTGCAGAATTCAGTGAATCATCGAATCTTTGAACGCACATTGCGCCCGCCAGTATTCT

GGCGGGCATGCCTGTCTGAGCGTCATTTCAACCCTCATGCCCCTAGGGCGTGGTGTTGGGGATCGGCCAGCGCCCGCGAG

GGACGGCCGGCCCCTAAATCTAGTGGCGGACCCGTCGTGGCCTCCCCTGCGAAGTAGTGATATTCCGCATAGGAGAGCGA

CGAGCCCCTGCCGTTAAACCcccAACTTTCTAAGGTTGACCTCAGATCAGGTAGGAATACCCGCTGAACTTAA

>OTU_234

GAAATGCGATAAGTAATGTGAATTGCAGAATTCAGTGAATCATCGAATCTTTGAACGCACCTTGCGCTCCTTGGTATTCC

GAGGAGCATGCCTGTTTGAGTGTCATGAAATTCTCAACCTCTTCTTGCTTTGCGGCTTGAAGTTGGCTTGGATTTGGGAG

CTTTGCTGGTTTCACAATCGGCTCTCCTTGAATGCATTAGCTGGACTTTCCGATTTAATAGCGGTTCACGGTGTGATAAC

TATCTACGCCTGGTAAATCCGTCTTTAAGTCTGGTCGAGCTTCTAATCAGTTCGTCCATTGCTGGACAAACACTTTTAAG

ATCTGACCTCAAATCAGGTAGGACTACCCGCTGAACTTAA

>OTU_235

GAAATGCGATAAGTAGTGTGAATTGCAGAATTCAGTGAATCATCGAATCTTTGAACGCACATTGCGCCCCTTGGTATTCC

ATGGGGCATGCTTGTTCGAGCGTCATTTGTACTCTCAAGCTTTGCTTGGTGTTGGGTGTTTGTCCTTGCCTTTGTGCTTG

GACTCGCCTTAAATTCATTGGCAGCCAATGTATTGGTTTGAAGCGCAGCACAATTTGCGGTTCTCACTGATCATCATTAG

CGTCCATAAAGCACAATTAACACTTTTGACCTCGGATCAAGTAGGGATACCCGCTGAACTTAA

>OTU_236

GAAATGCGATACTTGGTGTGAATTGCAGAATCCCGTGAACCATCGAGTCTTTGAACGCAAGTTGCGCCCCAAGCCTTCTG

GCCGAGGGCACGTCTGCCTGGGTGTCACAAATCGTCGTCCccccATCCTCTCGAGGATATCGGACGGAAGCTGGTCTCCC

GTgtgtTACCGCACGCGGTTGGCCAAAATCCTAGCTAAGGATGCCAGGAGCGTCTTGACATGCGGTGGTGAATTCAATTC

TCGTCAAATCGTCAGTCGTTTCGGTCCGAAAGCTCTTGATGACCCAAAGTCCTCAACGCGACCCCACGGTCAGGCGGGAT

CACCCGCTGAGTTTAA

>OTU_237

GAAATGCGATAAGTAGTGTGAATTGCAGAATTCAGTGAATCATCGAATCTTTGAACGCACATTGCGCCCCTTGGTATTCC

ATGGGGCATGCCTGTTCGAGCGTCATTTGTACCCTCAAGCTTTGCTTGGTGTTGGGTGTTTGTCCTCTCCTTTGCGTTTG

GACTCGCCTTAAAGCAATTGGCAGCCAGTGTTTTGGTATTGAAGCGCAGCACAATTTGCGATTCTAGCCGACAACACTTG

CGTCCATAAGCCTTttttAACTTTTGACCTCGGATCAGGTAGGGATACCCGCTGAACTTAA

>OTU_238

GAAATGCGATAAGTAATGTGAATTGCAGAATTCAGTGAATCATCGAATCTTTGAACGCACATTGCGCCCTCTGGTATTCC

GGAGGGCATGCCTGTTCGAGCGTCATTTCAACCCTCAAGCCTGGCTTGGTGATGGGGCACTGCCTGTAAAAGGGCAGGCC

CTGAAATTCAGTGGCGAGCTCGCCAGGACCCCGAGCGCAGTAGTTAAACCCTCGCTCTGGAAGGCCCTGGCGGTGCCCTG

CCGTTAAACCcccAACTTCTGAAAATTTGACCTCGGATCAGGTAGGAATACCCGCTGAACTTA

>OTU_239

GAAATGCGATAAGTAATGTGAATTGCAGAATTCAGTGAATCATCGAATCTTTGAACGCACATTGCGCCcccTGGTATTCC

GGggggCATGCCTGTTCGAGCGTCATTTCACCACTCAAGCCTCGCTTGGTATTGGGCATCGCGGTCCGCCGCGTGCCTCA

AATCGACCGGCTGGGTCTTCTGTCCCCTAAGCGTTGTGGAAACTATTCGCTAAAGGGTGTTCGGGAGGACTACCGCCGTT

AAAACAAACCCCATTTCTAAGGTTGACCTCGGATCAGGTAGGGATACCCGCTGAACTTAA

>OTU_240

GAAATGCGATAAGTAATGTGAATTGCAGAATTCAGTGAATCATCGAATCTTTGAACGCACATTGCGCCCGCCAGTACTCT

GGCGGGCATGCCTGTTCGAGCGTCATTACATCCCTCAAGCCCCAGCGGCTTGGTGTTGGGCTTCGGCCGTCCTCAGCGGC

GGCCGTGCCCCAAATACAGTGGCGGTCTCGCCcccGGCTCCTCTGCGTAGTAGTAACATCTCGCACTGGGACGGAGCGAA

GGCCACGCCGTAAAACAACCCAACTTTCTGAATGTTGACCTCGGATCAGGTAGGAATACCCGCTGAACTTAA

>OTU_241

GAAATGCGATACGTAATGTGAATTGCAGAATTCCGTGAATCATCGAATCTTTGAACGCACATTGCGCCCCTTGGTATTCC

AGGgggCATGCCTGTTTGAGCGTCATTTCCTTCTCAAGCACCTGTGCTTGGTTGTGGGTGACACTctctcGAGTTAGCTT

GAAATTGCTGGCCGCACTGCGGTGGAGCAGTTGGCTTGTCTGTCGTGCGCGGTGCCTCGGCGCCGGGCGTGGCTGGCATG

CGATTGTCGTACTAGGTTTTACCAATTCGGCAGGAGCGTGCTGGGCAGagagaCAATACAACCGCCCTCCCTCTGGCTAA

CAGTACTCTTTAAGTTTGACCTCAAATCAGGTAGGAATACCCGCTGAACTTAA

>OTU_242

GAAATGCGATAAGTAGTGTGAATTGCAGAATTCAGTGAATCATCGAATCTTTGAACGCACATTGCGCCCCTTGGTATTCC

ATGGGGCATGCCTGTTCGAGCGTCATTTGTACCTTCAAGCTCTGCTTGGTGTTGGGTGTTTGTCTCGCCTCTGCgcgcAG

ACTCGCCTCAAAACGATTGGCAGCCGGCGTATTGATTTCGGAGCGCAGTACATCTCGCGCTTTGACAACAAAACGACGAC

GTCCAAaaaGTACATTttttACACTCTTGACCTCGGATCAGGTAGGGATACCCGCTGAACTTAA

>OTU_243

GAATTGCGATAAGTAATGTGAATTGCAGATACTCGTGAATCATTGAATTtttGAACGCACATTGCGCCCTTGAGCATTCT

CAGGGGCATGCCTGTTTGAGCGTCATTTCCTTCTTCAAAAGATAATTTATTATTtttGGTTGTGGGCGATACTCAGGGTT

AGCTTGAAATTGGAGACTGTTTCAGTCTTttttAATTCAACACTTAGCTTCTTTGGAGACGCTGTTCTCGCTGTGATGTA

TTTATGGATTTATTCGTTTTACTTTACAAGGGAAATGGTAACGTACCTTAGGCAAAGGGTTGCTTTTAATATTCATCAAG

TTTGACCTCAAATCAGGTAGGATTACCCGCTGAACTTAA

>OTU_244

GAAATGCGATAAGTAGTGTGAATTGCAGAATTCAGTGAATCATCGAATCTTTGAACGCACATTGCGCCCCTTGGTATTCC

ATGGGGCATGCCTGTTCGAGCGTCATTTGTACTCTCAAGCTAAGCTTGGTGTTGGGTGTTTGTCCACTCCCTTGTGTTTG

GACTCGCCTTAAAACAATTGGCAGCCAGTGTTTTGGTATTGAAGCGCAGCACAATTTGCGATTCTAGCCTAGAATACTAG

CAACCATCAAGTCATTtttAACGCTTGACCTCGGATCAGGTAGGGATACCCGCTGAACTTAA

>OTU_245

GAAATGCGATAAGTAATGTGAATTGCAGAATTCAGTGAATCATCGAATCTTTGAACGCACATTGCGCCCTTTGGTATTCC

AAAGGGCATGCCTGTTCGAGCGTCATTTGTACCCTCAAGCTTTGCTTGGTGTTGGGCGTCTTGTCTCTAGCTTTGCTGGA

GACTCGCCTTAAAAGTAATTGGCAGCCGCCTACTGGTTTCGGAGCGCAGCACAAGTCGCACTctctATCAGCAAAGGTCT

AGCATCCATTAAGCCTTtttttCAACTTTTGACCTCGGATCAGGTAGGGATACCCGCTGAACTTAA

>OTU_246

GAAATGCGATACGTAATGTGAATTGCAGAATTCCGTGAATCATCGAATCTTTGAACGCACATTGCGCCCCTTGGTATTCC

AGGgggCATGCCTGTTTGAGCGTCATTTCCTTCTCAAACACTTGTGTTTGGTAGTGAGTGATACTCTGTTAAAACTGGGT

TAGCTTGAAATTGCAAGCCTTTTGGGGATGCGTCTAGAGAAGAGTTTTAGGCGGAAACGTCTGGCTCCTCCTCTATTTTC

CTTTAACCAAATGTCGTATTAGGTTTTACCGACTCGGCAGACAGGTTGCTGGAGACTGAGGTGGGTGATAGAAATATCGA

A

>OTU_247

GAAATGCGATAAGTAATGTGAATTGCAGAATTCAGTGAATCATCGAATCTTTGAACGCACATTGCGCCCGCCGGTATTCT

GGCGGGCATGCCTGTCCGAGCGTCATTTCAACCCCTCGAGCCcccGGGCCCGGTGTTGGGGAACGACCAGCAGCCCTCCG

GGGCGGTCGTCCCTGAAATACAGTGGCGGTCACGTCGCGGACCCTCTGCGTAGTAGCAACACCTCGCACTGGAGAGCGGC

gcgcCCACGCCCCGAAACACTTCTTTCACAGGTTGACCTCGGATCAGGTAGGAATACCCGCTGAACTTAA

>OTU_248

GAAATGCGATAAGTAGTGTGAATTGCAGAATTCAGTGAATCATCGAATCTTTGAACGCACATTGCGCCCTTTGGTATTCC

GAAGGGCATACCTGTTCGAGCGTCATTCTAAACCCTCAAGCCTGGCTTGGTCTTGGGCCTCTTGTCTGCCGGCCCCGGCA

GACTGGCCTCAAAAGCATCGGCGGCGAGTTGGTCGACCCACAAGCACAGTACATTGCTGCTTGCGGGGCGCCGGCTGGCG

CTGTAGAAACGCATTTTCTGACGTTGACCTCGGATCAGGTAGGAATACCCGCTGAACTTAA

>OTU_249

GAAATGCGATAAGTAGTGTGAATTGCAGAATTCAGTGAATCATCGAATCTTTGAACGCACATTGCGCCCCTTGGTATTCC

ATGGGGCATGCCTGTTCGAGCGTCATTTGTACCCTCAAGCTTTGCTTGGTGTTGGGCGTCTTGTCTTGTATTAGACTCGC

CTTAAAGTCATTGGCAGCCAGTGTTtttGGTAGTAAGCGCAGCACATTTTGCGTCTTAGTCCTTGAATCGTGGCATCCAT

GAAGCCTTttttCACTTTTGACCTCGGATCAGGTAGGGATACCCGCTGAACTTAA

>OTU_250

GAAATGCGATAAGTAATGTGAATTGCAGAATTCAGTGAATCATCGAATCTTTGAACGCACATTGCGCCCCTTGGTATTCC

GAGGGGCATGCCTGTTCGAGCGTCATTATAACCAATCTAGCTATGCTAGGTGTTGGGCTCCGCCGTTCGGTGGGCCTCAA

AGCAAGTGGCGGTGCCGTAGGGCTCTAAGCGTAGTAAATCtctcGCTATTGGGTTCCCGCGGTAACTAGCCAGTAACCcc

cATACTTTTCAGGTTGACCTCGGATCAGGTAGGGATACCCGCTGAACTTAA

>OTU_251

GAAATGCGATAAGTAATGTGAATTGCAGAATTCAGTGAATCATCGAATCTTTGAACGCACCTTGCGCTCCTTGGTATTCC

GAGGAGCATGCCTGTTTGAGTGTCATAAACCTCTCAAACCCAAGTTTTGGATTTATCCTTGCTTGAGTTTGGATTTGGGT

GTTTGCCAGTGATGAACTGACTCACCTTAAAAGTATTAGCTAGATCTGTCTTTGACTGGTTTGACTTGGCATAATAAGTA

TTTTGCTAAGGACATCTTCGGATGGCCAGGACTTGACTTTTGTCTGCTTACTAAACCTTACTTTAAGTGCATCTCTGGTG

TTACTTATAGTATTACTTTGACATATGGCCTCAAATCAGGTAGGACTACCCGCTGAACTTAA

>OTU_252

GAAATGCGATAAGTAATGTGAATTGCAGAATTCAGTGAATCATCGAATCTTTGAACGCACATTGCGCCCACCAGTACTCT

GGTGGGCATGCCTGTCCGAGCGTCATTTCAACCCTCAGGGCCCGTTCGCGGGACCTGGTGTTGGgggATCGGCCCCACCG

GCCGGCCCCGAAATACAGTGGCGGCACACCCGCGACCTCCTCTGCGTAGTAGCAATGCCTCGCAGCTGGATAGCGGTTGC

GCCTCGCCGTAAAACCccccACTTCTCAAAGGTTGACCTCGGATCAGGTAGGAATACCCGCTGAACTTAA

>OTU_253

GAAATGCGATAAGTAATGTGAATTGCAGAGCACAGTGAATCATCGAATCTTTGAACGCACATGGCGCCCGCTGGTAATCC

GGCGGGCATGCCTGTCCGAGCGTCATTTCAACCCTCAGAACCCCTctctcGGGAGGGCGTTACTGGTGTTGGGGATCGAG

CGCCccccGCGGCGCCCGTCCCTTAAATCTATTGGCGGCTCCGGAGTCTGTCTCCCCTGCGTAGTATTGCATCTCGCTTT

GGTGACGTCCCCGGGCTGCTGTGGCTCTGCCACGAAACCCCACTAGGTTGACCTCGGATCAGGTAGGGATACCCGCTGAA

CTTAA

>OTU_254

AAAATGCGATACCTGGTGTGAATTGCAGAATCCCGCGAACCATCGAGTTtttGAACGCAAGTTGCGCCCGAGGCCTTCTG

GCCGAGGGCACGCCTGCCTGGGCGTCACGCCAACAGACACTCCCACCCCATCATCGGGTGTAGGATGTGGCGTTTGGCTC

CCCGTGCCTGAAGGTGCGGTGGGCCGAAGTTGGGGCTGCCGGCATACCGTGTCGGGCACAGCACGTGGTGGGCGACTACA

AGTTGTTCTCGGTGCAGCGTCCCGGCACGCAGCTAGCTTGATGGCCCTAAGGACCCATGTACAACCGAAGCGCACTGTCG

CTCGGACCGCGACCCCAGGTCAGGCGGGACTACCCGCTGAGTTTAA

>OTU_255

GAAATGCGATAAGTAATGTGAATTGCAGAATTCAGTGAATCATCGAATCTTTGAACGCACCTTGCGCTCCTTGGTATTCC

GAGGAGCATGCCTGTTTGAGTGTCATTAAATTCTCAACCTCACCAGTTTTGTAATGAAACTGTGTAAGGCTTGGATGTGG

gggTTTATGCAGGCTGCCTCTGGCGGTCTGCTCCCCTGAAATGCATTAGCGAGTTCACTAAGCTCCGTCTATTGGTGTGA

TAATTATCTACGCCGTGGAAAGGGTTTAGACTTGCTTCTAACCGTCTGCAAAGACAACTTTTGACAATTTGACCTCAAAT

CAGGTAGGACTACCCGCTGAACTTAA

>OTU_256

GAAATGCGATAAGTAATGTGAATTGCAGAATTCAGTGAATCATCGAATCTTTGAACGCATCTTGCGCTCCTTGGTATTCC

TTGGAGCATGCCTGTTTGAGTATCATGAGCAAATCTCAAAGTCAATTCCTTTTGTTGATTCAATTGGTTTTGCTTTGGAC

TTGGAGGCCTTGCAGATTTCACAGTCTGCTCCTCTTAAATGCATTAGCTGGATCTCAGTAATTATGCTTGGTTCCACTCG

GCGTGATAAGTATCACTCGCTGAGGACACTGTTAAaaaGGTGGCCAGGAAAATACTGATTGAACCGCTTCTAACGGTCTA

TTAAGTTAGACAATTACCCTTAAGTTTGATCTCAAATCAGGTAGGACTACCCGCTGAACTTAA

>OTU_257

GAAATGCGATAAGTAGTGTGAATTGCAGAATTCAGTGAATCATCGAATCTTTGAACGCACATTGCGCCCCTTGGTATTCC

ATGGGGCATGCCTGTTCGAGCGTCATCTACACCCTCAAGCTCTGCTTGGTGATGGGCGTCTGTCCCGCCTCTGCGCGTGG

ACTCGCCCTAAAGTTATTGGCAGCCGTCCTGTCCGCCTCTCGCGCAGCACAAGTGCGCTTCTCGAGGCGGCGATCCGCGT

CCATTAAGCCCGTATATCACGCATTTTGACCTCGGATCAGGTAGGGATACCCGCTGAACTTAA

>OTU_258

AAAGCGCGATAGGTAATGCGAATTGCACACGTGAGTCATTGAATCTTTGAACGCACATTGCGCCcccTGGTATTCCGGgg

ggCATGCCTGTTCGAGCGTCATTTCACCACTCAAGCCTCGCTTGGTATTGGGCAACGCGGTCCGCCGCGTGCCTCAAATC

GTCCGGCTGGGTCTTCTGTCCCCTAAGCGTTGTGGAAACTATTCGCTAAAGGGTGTTCGGGAGGCTACGCCGTAAAACAA

CCCCATTTCTAAGGTTGACCTCGGATCAGGTAGGGATACCCGCTGAACTTAA

>OTU_259

GAAATGCGATAAGTAATGTGAATTGCAGAATTCAGTGAATCATCGAATCTTTGAACGCACATTGCGCCCTCTGGTATTCC

GGAGGGCATGCCTGTTCGAGCGTCATTACAACCCTCAAGCTCTGCTTGGTATTGGGCGTCACCAGTTTGGTGTGCTCTAA

AATCAGTGGCGGTGCCATCTGGCTCTAAGCGTAGTACATCtctcGCTATGGGTGCCCGGTGGATGCTTGCCAACAACCcc

cAATTttttAAGGTTGACCTCGGATCAGGTAGGGATACCCGCTGAACTTAA

>OTU_260

AAAATGCGATAAGTAATGTGAATTGCAGAATTCAGTGAATCATCGAATCTTTGAACGCACATTGCGCCCGCCAGTATTCT

GGCGGGCATGCCTGTTCGAGCGTCATTTCAACCCTCAAGCACAGCTTGGTGTTGGGATTCGTGAGGTAAACTCACGGTCC

CCAAATTGATTGGCGGTCACGTCGAGCTTCCATAGCGTAGTAATTACACCTCGTTACTGGTAATCGTCGCGGCCACGCCG

TTAAACCCCAACTTCTGAATGTTGACCTCGGATCAGGTAGGAATACCCGCTGAACTTAA

>OTU_261

GAAATGCGATAAGTAATGTGAATTGCAGAATTCAGTGAATCATCGAATCTTTGAACGCACATTGCGCCCGCCAGTATTCT

GGCGGGCATGCCTGTTCGAGCGTCATTACAACCCTCAGGCCcccGGGCCTGGCGTTGGGGATCGGCGGAGCCccccGTGG

GCACACGCCGTCCcccAAATGCAGTGGCGGTCCCGCCGCAGCTTCCATCGCGTAGTAGCTAACACCTCGCGACTGGAGAG

CGGCGCGGCCACGCCGTAAAACACCCAACTCTTCTGAAGTTGACCTCGAATCAGGTAGGAATACCCGCTGAACTTAA

>OTU_262

GAAATGCGATAAGTAATGTGAATTGCAGAATTCAGTGAATCATCGAATCTTTGAACGCACATTGCGCCCTTTGGTATTCC

GAAGGGCATGCCCGTTCGAGCGTCATTTCACCACTCAAGCCTAGCTTGGTATTGGGCGTCGCGTCTGCCGcgcgcCTCGA

AGTCTCCGGCCCCGCGGCCTGTCCCCGAGCGTTGTGGCACTTATGTTCCGCTCCGGGTGGCAGGCGGTGGCTGGCCGTTA

AACTCTTGCAAAGGTTGACCTCGGATCGGGTAGGGATACCCGCTGAACTTAA

>OTU_263

GAAATGCGATACGTAATGTGAATTGCAGAACTCAGTGAATCATCGAATTtttGAACGCACCTTGCGCTctctAGTTATTC

TGGAGAGCATGCTTGTTTGAGTGTCGCGAACCTCTCAACCCCGTTAAAGGCTTAATTGACTTTGATTGCTTGGGCTTGGA

TCATGGTCACTTGTCGTTGTATAACGACTTGACTGAAATACAACAAGTTGAATGAATCCATTTGCAGCTTGACCTGATGT

TGTAAACTACTCATCGGGGATGCATAAGGAAGTCAAGGAACTTTAACAGAACCCCATATAGTACTTAGTACAATTTATGA

CCTCAAATCAAGTAGGACTACCCGCTGAACTTAA

>OTU_264

GAAATGCGATAAGTAATGTGAATTGCAGAATTCAGTGAATCATCGAATCTTTGAACGCACATTGCGCCCATTAGTATTCT

AGTGGGCATGCCTGTTCGAGCGTCATTTCAACCCTTAAGCCTAGCTTAGTATTGGGAGCTGACTTTACTGTCACTCCTCA

AATTCAACGGCGGATTTATAGCAATCTCTGAACGTAGTAATCTTTATCTCGTTtttGAAATACTGTAAACCTCAGCCGCT

AAACCcccAATTtttAATGGTTGACCTCGGATCAGGTAGGAATACCCGCTGAACTTAA

>OTU_265

GAAATGCGATAAGTAATGTGAATTGCAGAATTCAGTGAATCATCGAATCTTTGAACGCATATTGCGCCCTTTGGCATTCC

GAAGGGCATACCTGTTCGAGCGTCATTACACCCCTCAAAGATGACTCTTTGGCGTTGGGCAATGCCTAAAGGCATGCCTC

AAAATCAGCGATGGTGATATTCAACCACAGGCGCAcacacGTCGTCTAGCTGGCGGGATGTTGACAGTCAATTGATGAAT

TTCTAAATGTTGACCTCGGATCAGGTAGGAATACCCGCTGAACTTAA

>OTU_266

GAAATGCGATAAGTAATGTGAATTGCAGAATTCAGTGAATCATCGAATCTTTGAACGCACATTGCGCCCTCTGGTATTCC

GGAGGGCATGCCTGTTCGAGCGTCATTTCAACCCTCAAGCCTGGCTTGGTGATGGGGCACTGCTctctcGCGGGAGCAGG

CCCTGAAATCTAGTGGCGAGCTCGCCAGGACCCCGAGCGTAGTAGTTACATCTCGCTCTGGAAGGCCCTGGCGGTGCCCT

GCCGTTAAACCcccAACTTCTGAAAATTTGACCTCGGATCAGGTAGGAATACCCGCTGAACTTAA

>OTU_267

GAAATGCGATAAGTAGTGTGAATTGCAGAATTCAGTGAATCATCGAATCTTTGAACGCACATTGCGCCCTTTGGTATTCC

TTAGGGCATGCCTGTTCGAGCGTCATTTGAAACCTCAAGCTCAGCTTGGTGTTGGGTGTCTGTCCCCGCCTCCGCGGGGA

CTCGCCTCAAAATCATTGGCGGCCGGTACGTTGGCTTCGAGCGCAGCAGAAACGCGAACTCGAGGCCCGGCGGATCGGCG

ACCAGAAGTCTCTTCTTCACAGTTTGACCTCGGATCAGGTAGGGATACCCGCTGAACTTAA

>OTU_268

GAAATGCGATAAGTAGTGTGAATTGCAGAATTCAGTGAATCATCGAATCTTTGAACGCACATTGCGCCCCTTGGTATTCC

ATAGGGCATGCCTGTTCGAGCGTCGTTTCAAACCTTCAAGCCTAGCTTGGTGTTGGGTGACTGTCCTGCGGAAATCCCGC

GGACTCGCCTCAAATCTATTGGCAGCAGACATTGTAGCTAATTGCGCAGCACATCGCGCCTTAAGCTCGGTGCCGGCTAT

CCACGAGCCTTGTCTCACCAGTTTGACCTCGGATCAGGTAGGGATACCCGCTGAACTTAA

>OTU_269

GAAATGCGATACGTAGTGTGAATTGCAGAATTCAGTGAATCATCGAATCTTTGAACGCACATTGCGCCCTTTGGTATTCC

AAAGGGCATGCCTGTTCGAGCGTCATTTGTACCCTCAAGCTTTGCTTGGTGTTGGGCGTTttttGTCTTGCCTTGCGGCG

GAGACTCGCCTTAAAACGATTGGCAGCCGGCCTACTGGTTTCGGAGCGCAGCACAGCTTTTGCGCTTGCCAACCAGCTAA

AGAGGTCAGCAATCCATCAAGCCCATTTCTCACTTTTGACCTCGGATCAGGTAGGGATACCCGCTGAACTTAA

>OTU_270

GAAATGCGATAAGTAATGTGAATTGCAGATTTTCAGTGAATCATCGAATCTTTGAACGCACCTTGCGCTCCTTGGTATTC

CGAGGAGCATGCCTGTTTGAGTGTCATTAAATCCTCAACCCCGTCCGTCGGTCGGGGCTTGGACTTTGGAGCGTGCTGGC

GAAGGTCGGCTCCTCTTTAAATGCATCAGCGGAGAAaaaCAAACCTTttttCCcccTTCAGCGTGATAACTGCGTTGCGC

TGTGGTCGCGAAAGGGTCCGCTCATAATCGTCCTCGGACAAACACCGAATCTGTTTTGACCTCAGATCAGGTAGGACTAC

CCGCTGAACTTAA

>OTU_271

GAAATGCGATAAGTAATGTGAATTGCAGAATTCAGTGAATCATCGAATCTTTGAACGCACATTGCGCCCGCTAGTACTCT

AGCGGGCATGCCTGTTCGAGCGTCATTTCAACCCTCAAGCCCTGCTTGGTGTTGGGGCCCTACGGCTGCCGTAGGCCCTG

AAAGGAAGTGGCGGGCTCGCTACAACTCCGAGCGTAGTAGAATCCTATCTCGCTAGGGAGGTGCGGCGTGCTCCTGCCGT

TAAAGACCCCATCTTTAACCAAGGTTGACCTCGGATCAGGTAGGAATACCCGCTGAACTTAA

>OTU_272

GAAATGCGATAAGTAGTGTGAATTGCAGAATTCAGCGAATCATCGAATCTTTGAACGCACATTGCGCCCTTCGGTTATTC

CTTAGGGCATGCCTGTTCGAGCGTCATTTCAACCTTCAAGCCCCGCTTGGTGTTGGGCGCTGTCCCGCCTCTGCGCGAGG

ACTCGCCCCAAATGAATTGGCAGTCGCACCcccGATACGCGAGCGCAGCAAAGTCGcgcgAGCCGAACCGGCAGGGACGG

ACGCTCCACGAGACCCACCACAGTCTTGACCTCGGATCAGGTAGGGATACCCGCTGAACTTAA

>OTU_273

GAAATGCGATAAGTAATGTGAATTGCAGAATGGACGTGAATCATCGAATCTTTGAACGCACCTTGCGCTCGCTGGTATTC

CGGCGAGCATGCCTGTTTGAGTATCGCGCATTCTCGCGGGAGGAACGTGTTTTCCTCTTGCGGTGCGTGGGTGGTTGTCG

ACGTCCGCGTCGACTCGCCTGAAATGCATGAGCACGTGTAGATATACGCACGCACCACCGTCTAGGTAAAGCCGAGAGTG

GAGGGGTGAGCGGATCTCGCGGCTTCGAATGGGTGTCTCGCGACACCAACTGATCGAACGCACTGGTCTCAAATCAGGTA

GGACTACCCGCTGAACTTAA

>OTU_274

GAAATGCGATAAGTAATGTGAATTGCAGAATTCAGTGAATCATCGAATCTTTGAACGCACATTGCGCCCCTTGGTATTCC

GAGGGGCATGCCTGTTCGAGCGTCATTACACCACTCAAGCATAGCTTGGTATTGGGCAACCGCCcccGCCAGTCGGGGAC

GCGCCTCAAACACCTCGGCGGAGCCTCACCGGCTTTGGGCGTAGTAGAATTTCTAATAACGTCCTTTAACGGAGATGGTT

CCTTTGCCGATTGAAGCCTTTATTtttCTAGGTTGACCTCGGATCAGGTAGGGATACCCGCTGAACTTAA

>OTU_275

GAAATGCGATAGCTAGTGTGAATTGCAGATTTTAGTGAATCATCGAGTCTTTGAACGCACATTGCGCCCTTTGGTATTCC

TCAGGGCATGCCTATTCGAGCGTCGTTTCGACCTCGAGCGCAGCTTGGTGTTGAGGATTGCATTGCATCCTTCAAAAGCA

TCGGCGGCAGCGCAGCTCCATCGCAGCACAATGCATTGGTACCTGTCAACTGCTTGCCTTTTCTCCTTCTTTACCAGCTC

GACCTCGAATTAGGCAGGGATACCCGCTGAACTTAA

>OTU_276

GAAATGCGATAAGTAGTGTGAATTGCAGAATTCAGTGAATCATCGAATCTTTGAACGCACATTGCGCCCCTTGGTATTCC

ATGGGGCATGCCTGTTCGAGCGTCATTTGTACCCTCAAGCTTTGCTTGGTGTTGGGTGTTTGTTCGCTGCCCTTGGGCAG

GACGGACTCGCCTTAAAACAATTGGCAGCCGGCATATTGGCCTGGAGCGCAGCACATATTGCGCTTCTTGTCAATTGTGT

TGGCATCCATCAAGACCTTCTTTAAGCTCTTGACCTCGGATCAGGTAGGGATACCCGCTGAACTTAA

>OTU_277

GAAATGCGATAAGTAATGTGAATTGCAGAATTCAGTGAATCATCGAATCTTTGAACGCACCTTGCACCTTTTGGTATTCC

GAAAGGTATGCCTGTTTGAGTGTCATGAAATCTCAATCCCCTCGGGTTTTACGACCTGATCGGACTTGGACTTGGACGTC

TGCCACCTGTGGCTCGTCTTAAATGGATTAGTGGGTCTTTGTGCTTACAGCTTGACGTGATAAGTTTCGTCTCGCTTTGT

GCAAGGTCTGCTTACAACCAACCTTCACTTTTATGACTCTGACCTCAAATCAGGTAGGGCTACCCGCTGAACTTAA

>OTU_278

GAAATGCGATAAGTAGTGTGAATTGCAGAATTCAGTGAATCATCGAATCTTTGAACGCACATTGCGCCCCTTGGTATTCC

ATGGGGCATGCCTGTTCGAGCGTCATTACACCCTCAAGCTCTGCTTGGTGTTGGGCGCCTGTCCCGCCTCGTGCGCGGAC

TCGCCCTAAATGTATTGGCAGCGGTTTGCCAGCCTGTAGCGTAGCACTAATGCGCCTGCGGGCTTCGGCGGATTCGCGTC

CACTAAGTATCACCCCAGTTTGACCTCGGATCAGGTAGGGATACCCGCTGAACTTAA

>OTU_279

GAAATGCGATAAGTAGTGTGAATTGCAGACTTTAGTGAATCATCGAATCTTTGAACGCACATTGCGCCCCTTGGTATTCC

ATTGGGCATGCCTGTTCGAGCGTCATTTACACCCTCAAGCCTAGCTTGGTGTTGGGCGTTGTCCGCAAGGACTCGCCTCA

AATTCATTGGCAGCGGCATCGTCAGCTTCTCGCGCAGCACATTGCGCTTCAGAAGGCTTCTGGCGGGCTCGCGTCCACTA

AGATACCACCCAGTTTGACCTCGAATCAGGTAGGGATACCCGCTGAACTTAA

>OTU_280

GAAATGCGATAAGTAATGTGAATTGCAGAATTCAGTGAATCATCGAATCTTTGAACGCACATTGCGCCCTCCGGTATTCC

GGggggCATGCCTGTTCGAGCGTCATTTCACCACTCAAGCAATGCTTGGTATTAGGCACTCGTCCGTCCCTCGCGGCGGG

CGGGCCTCAAAACCTTCGGCGAAGTCACACCGGCTTTGGGCGCAGTAGAATCATCTTAAACGCTCTGTGAGTCCGGTCGA

GTCTTTTGCCGCGAAAGATTCTCACGAATCATCTTtttCAAAGGTTGACCTCGGATCAGGTAGGGATACCCGCTGAACTT

AA

>OTU_281

GAAATGCGATACGTAGTGTGAATTGCAGAATTCAGTGAATCATCGAATCTTTGAACGCACATTGCGCCCTTTGGTATTCC

AAAGGGCATGCCTGTTCGAGCGTCATTTGTACCTTCAAGCTTTGCTTGGTGTTGGGCGTTttttGTCTCCCTCTTGCTGG

GAGACTCGCCTTAAAACGATTGGCAGCCGGCCTACTGGTTTCGGAGCGCAGCACATATTTTGCACTCTGTGTCAGGAGAA

AGGACGGTAATCCATCAAGACTCTACAAATTTTAACTTTTGACCTCGGATCAGGTAGGGATACCCGCTGAACTTAA

>OTU_282

AAAATGCGATAAGTAATGTGAATTGCAGAATTCAGTGAATCATCGAATCTTTGAACGCACATTGCGCCCGCCAGTACTCT

GGCGGGCATGCCTGTCTGAGCGTCATTTCAACCCTCAGGGCCCGTCCGCGGGACCTGGCGTTGGGGATCAGCCCTCCGGG

GCTGGCCCTGAAATCTAGTGGCGGTCCTCTCGCGACCTCCTCTGTGCAGTAGTAATACCTCGCAGCTGGATAGCGTACGG

GCCACGCCGTAAAACCccccACTTCTCAAGGTTGACTCAGATCAGGTAGGAATACCCGCTGAACTTAA

>OTU_283

GAAATGCGATAAGTAATGTGAATTGCAGAATTCAGTGAATCATCGAATCTTTGAACGCACCTTGCGCTCCATGGTATTCC

GTGGAGCATGCCTGTTTGAGTGTCATGAATACTTCAACCCTCCTCTTTCTTAATGATTGAAGAGGTGTTTGGTTTCTGAG

CGCTGCTGGCCTTTACGGTCTAGCTCGTTCGTAATGCATTAGCATCCGCAATCGAACTTCGGATTGACTTGGCGTAATAG

ACTATTCGCTGAGGAATTCTAGTCTTCGGATTAGAGCCGGGTTGGGTTAAAGGAAGCTTCTAATCAGAATGTCTACATTT

TAAGATTAGATCTCAAATCAGGTAGGACTACCCGCTGAACTTAA

>OTU_284

GAAATGCGATAAGTAATGTGAATTGCAGAATTCAGTGAATCATCGAATCTTTGAACGCACCTTGCGCCCGTTGGTATTCC

GACGGGCATGCCTGTTTGAGTGTCGTGAACAACCTCAACCTTGATTGGTTTTACCATCAAGGCTTGGACTTGGGTGTTGC

TGCTGCAAAGTGGCTCGCCTTAAAACTATTAGCTGGACTTGTCTATGACTTGGTTCGACTCGATGTAATAAGTATTCATC

GGGGACAGGGTTAAACCTGGCCAAGGCTTGATGCCGTCTGCTTCTAATCATGTTCTCACTATGAGACGCTACTTTACTGt

gtgGCCTCAAATCAGGTAGGATTACCCGCTGAACTTAA

>OTU_285

GAAATGCGATAAGTAATGTGAATTGCAGAATGGACGTGAATCATCGAATCTTTGAACGCACCTTGCGCTCGCTGGTATTC

CGGCGAGCATGCCTGTTTGAGTATCGCGCATTCTCGCGGGAGGAACGTGTTTTCCTCTTGCGGTGCGTGGGCGGATGTCG

GCGTTTGCGCGTCGACTCGCCTGAAATGCATGAGCACGTGTAGATGCGCACGCACCACCGTCTAGGTAAAGCCGAGAGCG

GAGGGGTGAGCGGATCTGACGGCTTCGAATGGGTGTCTAGCACACCACGTCATCGAACGCACTGGTCTCAAATCAGGTAG

GACTACCCGCTGAACTTAA

>OTU_286

GAAATGCGATAAGTAATGTGAATTGCAGAATTCCGTGAATCATCGAATCTTTGAACGCACATTGCGCCCTCTGGTATTCC

GGggggCATGCCTGTCCGAGCGTCATTGCAACCCTCAAGCGCGGCTTGtgtgtTGGGCCTTCGTCCcccGTGGACGTGCC

CGAAATGCAGTGGCGGCGTCGTGTTCCGGTGCCCGAGCGTATGGGGCTTTGTCACCCGCTCATGGAGGCCCGGCCGGCCC

CGGCCAACCcccAATCGAGGACTCGTTCCTCCACCAAGGTTTGACCTCGGATCAGGTAGGGATACCCGCTGAACTTAA

>OTU_287

GAAATGCGATAAGTAATGTGAATTGCAGAATTCAGTGAATCATCGAATCTTTGAACGCACATTGCGCCCCGTGGTATTCC

GCGGGGCATGCCTGTTCGAGCGTCATTTCAACCCATCAAGCTTCGGCTTGGTCTTGGGGCCTGCGATTCCGCAGCCTCTA

AACCCAGTGGCGGTGCCCTCGAGCTCTGAGCGTAGTAATTCTTCTCGCTATAGGGTCTCGGTGGTGTCTTGCCAGTAACC

cccAATTttttAAAGGTTGACCTCGGATCAGGTAGGGATACCCGCTGAACTTAA

>OTU_288

GAAACGCGAAAGGTAATGCGAATTGCAGAATCAGTGAGTCATCGAATCTTTGAACGCACATTGCGCCCCTCGGTATTCCG

GggggCACGCCTGTTCGAGCGCCATTACACCccccAAGCTCTGCTTGATGTTGGGCGAGGTCTCCGTTCGCGGGGACCCG

CCTGGAACGCGTGGGCGCCccccGCCGGACCACGAGCGTAGCAAGACCTTCAAAACGTCATCGCTTCGTTGGCTCCGGTG

GGCGGTCCGCCGAACCTTTGATCTTCGGATCGACCTTATAGTTTGGCCTCGGATCAGGCGGGGATACCCGCTGAACTTAA

>OTU_289

GAAATGCGATAAGTAATGTGAATTGCAGAATTCAGTGAATCATCGAATCTTTGAACGCACATTGCGCCCGCCAGTATTCT

GGCGGGCATGCCTGTTCGAGCGTCATTTCAACCATCAAGCCCACGGCTTGTGTTGGGGACCTGCGGCTGCCCGCAGGCCC

TGAAAACCAGTGGCGGTCTCGCTGTCACACCGAGCGTAGTAGCATTATCTCGCTCAGGGCGTGCTGCGGGTTCCGGCCGT

TAAaaaGCCTTCGTAACCCAAGGTTGACCTCGGATCAGGTAGGAAGACCCGCTGAACTTAA

>OTU_290

GAAATGTGATAAGTAATGTGAATTGCAGAATTCAGTGAATCATCGAATCTTTGAACGCACCTTGCGCTCCCTGGCATTCC

GGGGAGCATGTCTGTTTGAGTGTCATGAACTCTTCAACCCACCAGTTTCTTGTAAACTGGCTGGTGTTTGGATTTTGAGC

GTTGCTGGATCTTCGGATCGAAGCTCGTTCGTAATatatTAGCATCTCTAATTCGAACTCGGATTGACTCAGTGTAATAG

ACTATTCGCTGAGGACACGTGCTTGCATGTGGCCGAATGAGATCTCAGTAGAGGCTTCCAACCCTTCTAGTCAACTTTAG

ATTAGACCTCAGATCAGGCAGGATTACCCGCTGAACTTAA

>OTU_291

GAAATGCGATACGTAGTGTGAATTGCAGAATTCAGTGAATCATCGAATCTTTGAACGCACATTGCGCCCTTTGGTATTCC

AAAGGGCATGCCTGTTCGAGCGTCATTTGTACCCTCAAGCTTTGCTTGGTGTTGGGCGTTGTTTGTCTTTGGTCCGCCCA

AAGACTCGCCTTAAAACAATTGGCAGCCGGCCTACTGGTTTCGCAGCGCAGCACATTtttGCGCTTGCAATCAGCAAAAG

AGGTTGGTCATCCATCAAGACTACATTTATACGTTTGACCTCGGATCAGGTAGGGATACCCGCTGAACTTAA

>OTU_292

GAAATGCGATAAGTAATGTGAATTGCAGAATTCAGTGAATCATCGAATCTTTGAACGCACCTTGCGCCCCTTGGTTATTC

CTTGGAGCATGCCTGTTTGAGTATCATGAACACCTCAACTCTCATGGTCTTCCATGATGAGCTTGGACTTTGGgggTTTG

CTGGCCCTGCGGCCGGCTCCTCTCAAATGAATCAGCTTGCCAGTGTTTGGTGGCGTCACGGGTGTGATAACTATCTACGC

TCGCGGTCGTCTGCCAGGTAACCCTCAGCAATGAAGGTTCATTGGAGCTCATAAACGTCtctcCTCGGCGAGGACAACTT

TTGAACGTTCGATCTCAAATCAGGTAGGATTACCCGCTGAACTTAA

>OTU_293

GAAATGCGATAAGTAATGTGAATTGCAGAATTCAGTGAATCATCGAATCTTTGAACGCACATTGCGCCCGCCAGTATTCT

GGCGGGCATGCCTGTTCGAGCGTCATTTCAACCATCAAGCCCCGCGCTTGTGTTGGGGACCTGCGGCTGCCGCAGGCCCT

GAAAACCAGTGGCGGGCTCGCTGTCACACCGGGCGTAGTAGATTTTCATCTCGCTCAGGGCGTGCTGCGGGTTCCGGCCG

TAAaaaaGCCTTATTTTACCCAAGGTTGACCTCGGATCAGGTAGGAAGACCCGCTGAACTTAA

>OTU_294

GAAATGCGATAAGTAGTGTGAATTGCAGAATTCAGTGAATCATCGAATCTTTGAACGCACATTGCGCCCCTTGGTATTCC

ATGGGGCATGCCTGTTCGAGCGTCATTACACCCTCAAGCTCTGCTTGGTGTTGGGCGCTGTCCCGCCTCGTGCGTGGACT

CGCCCTAAATGTATTGGCAGCGGTTTGCCGGCCCGTAGCGTAGCACAATTGCGCTGCGAGCTTTGGCAGATTAGCGTCCA

CTAAGTGACCATCCACAGTTTGACCTCGGATCAGGTAGGGATACCCGCTGAACTTAA

>OTU_295

GAAATGCGATAAGTAATGTGAATTGCAGAATTCAGTGAATCATCGAATCTTTGAACGCACATTGCGCCCGCCAGCACTCT

GGCGGGCATGCCTGTCCGAGCGTCATTTCAACCCTCAGGACCCCTTCGGgggACCTGGCGTTGGGGATCGGCCCGCCccc

GTGCGGGCCGGCCCCGAATCACAGTGGCGGCCCCATCGCGAGCTCCCCTGCGCAGTAGCGATACCTCGCAGCCGGATAGC

GGTGCGGCCACGCCGTAAAACACCCCACTTCTTCAAGGTTGACCTCGGATCAGGTAGGAATACCCGCTGAACTTAA

>OTU_296

GAAATGCGATAAGTAATGTGAATTGCAGAATTCAGTGAATCATCGAATCTTTGAACGCACATTGCGCCCGCCAGTACTCT

GGCGGGCATGCCTGTCCGAGCGTCATTTCAACCCTCAGTTCCccccTTCCGGggggTGAGCTGGTGTTGGGGATCGGCCG

TCCCTCGCGGCGCCGGCCCTTAAATTCAGTGGCGGCACCACCGCGAACTCCTCTGCGTAGTAGCTATACCTCGCAGGCGG

ATAGCGGAGGCGACCAGCCGTGAAACCcccATCTTTTACAAGGTTGACCTCGGATCAGGTAGGAATACCCGCTGAACTTA

A

>OTU_297

GAAATGCGATAAGTAGTGTGAATTGCAGAATTCAGTGAATCATCGAATCTTTGAACGCACATTGCGCCCCTTGGTATTCC

ATGGGGCATGCCTGTTCGAGCGTCATTTGTACCCTCAAGCTACCTATTCATTtttATTGAACAGCTTGGTGTTGGGTGTT

TGTCCACTCCCGCGTGTTTGGACTCGCCTCAAAACAATTGGCAGCCAGTGTTTTGGTATTGAAGCGCAGCACAATTTGCG

ATTCTAGCCCATGAATACTAGCAACCATCAAGCCCATTtttAACGCTTGACCTCGGATCAGGTAGGGATACCCGCTGAAC

TTAA

>OTU_298

GAAATGCGATAAGTAGTGTGAATTGCAGAATTCAGTGAATCATCGAATCTTTGAACGCACATTGCGCCCCTTGGTATTCC

ATGGGGCATGCTTGTTCGAGCGTCATTTGTACCCTCAAGCTATGCTTGGTGTTGGGTGTTTGTCCTTTCCGCGTGTTAGG

ACTCGCCTTAAATTCATTGGCAGCCGACGTATTGGTTTGGAGCGCAGCACAATTTGCGAGCCAGGCGAGTATACCAGCGT

CCATTAAGCACAATTAACACTTTTGACCTCGGATCAAGTAGGGATACCCGCTGAACTTAA

>OTU_299

GAAATGCGATACGTAATGTGAATTGCAAATTCAGTGAATCATCGAGTCTTTGAACGCACATTGCGCCcccTGGTATTCCG

GggggCATGCCTGTCCGAGCGTCATTGCTGCCCTCAAGCACGGCTTGtgtgtTGGGCCCCGTCCTCCGATCCCGGgggAC

GGGCCCGAAAGGCAGCGGCGGCACCGCGTCCGGTCCTCGAGCGTATGGGGCTTTGTCACCCGCTCTGTAGGCCCGGCCGG

CGCTTGCCGATCAACCCAAATTtttATCCAGGTTGACCTCGGATCAGGTAGGGATACCCGCTGAACTTAA

>OTU_300

GAAATGCGATAAGTAATGTGAATTGCAGAATTCAGTGAATCATCGAATCTTTGAACGCACATTGCGCCCTTTGGTATTCC

AAAGGGCATGCCTGTTTGAGCGTCAGTTCTCACTTAAAACTTAGGTTtttGTTATGAGATGTAGCTTTATCTTtttttAA

AATAAAGCTTCTCTTGAAATTAATTGGCGATATTTGTAAATAAGTCAACGTTATAGATTTTAACTTTCTTTATTGAAAGT

AAATTtttCGTTGGCCAAAACTTATTTTACGTTTGTGTCGCTTGATCAAaaaaaaaaTTATTATTTGTTTGACCTCAAAT

CAGGTAGGACTACCCGCTGAACTTAA

>OTU_301

GAAATGCGATAAGTAATGTGAATTGCAGAATTCAGTGAATCATCGAATCTTTGAACGCACCTTGCGCCCTCTGGTATTCC

GGAGGGCATGCCTGTTTGAGTGTCATGTAGACTCAATCCCTCGGGTTTCCGAGGAGATTGGACTTGGGTGTTGCCGCTCT

GCCGGCTCGCCTTAAAAGACTTAGCGGGATAGCACCGTAGTCGGCGTAATAAGTTTCGTCGGTGAAGGTTGTGATGACTG

CTTACAATCGCCCTTGGGCAATTtttGACTCTGACCTCAAATCAGGTAGGACTACCCGCTGAACTTAA

>OTU_302

GAAATGCGATAAGTAATGTGAATTGCAGAATTCAGTGAATCATCGAATCTTTGAACGCACCTTGCGCCCTCTGGTATTCC

GGAGGGCATGCCTGTTTGAGTGTCATGAACACCTCACCcccTCGGGTTTCCGATCGGGTGGACTTGGGCGTTGCCAGTTA

CATGGCTCGCCTTAAATGTATTAGCGGTTGTTGACGTAATAAGTTTCGTCTTGTCGACCGCTCCTAATCTCCTTAGGGAT

GTCTTTGGACACTTCATGATCTGACCTCAAATCAGGTAGGACTACCCGCTGAACTTAA

>OTU_303

GAAATGCGATAAGTAATGTGAATTGCAGAATTCAGTGAATCATCGAATTtttGAACGCATATTGCGCCCTTTGGCATTCC

GAAGGGCATGCCTGTTCGAGCGTCATTACACCCCTCAAACCTGTGTTTGATGTTGGGCACCTGCCGTAAGGGCTTGCCTA

AAAGACAGTGACGGTAACCTTAAACCTTAGGCGCACAGATGCTGCCATGAAGGCAAATGGAAGCCGGTCTTAAACCAAAA

TTTCTGAATATTGACCTCGGATCAGGTAGGAATACCCGCTGAACTTAA

>OTU_304

GAAATGCGATAAGTAGTGTGAATTGCAGAATTCAGTGAATCATCGAATCTTTGAACGCACATTGCGCCCCTTGGTATTCC

ATGGGGCATGCCTGTTCGAGCGTCATTTGTACCTTCAAGCTTTGCTTGGTGTTGGGTGTTTGTCTCGCCTCTGCgcgcAG

ACTCGCCTCAAAACGATTGGCAGCCGGCGTATTGATTTCGGAGCGCAGTACATCTCGCGCTTTGTAGTCTCAACGACGAC

GTCCAAaaaGTACTTttttCACTCTTGACCTCGGATCAGGTAGGGATACCCGCTGAACTTAA

>OTU_305

GAAATGCGATAAGTAATGTGAATTGCAGAATTCAGTGAATCATCGAATCTTTGAACGCACATTGCGCCCCTTGGTATTCC

GAGGGGCATGCCTGTTCGAGCGTCATTACACCACTCAAGCATCGCTTGGTATTGGGAACGGTCCGTCGCAAGGCGGGCCT

TCCTCGAAGACCTCGGCGGGGTTCAACCAACTTCGGGCGTAGTAGAGTTAAATCGAACGTCTTATAAGCTTGGTCGGATC

TCATTGCCGTTAAACCTTTAAATTTTCTAGGTTGACCTCGGATCAGGTAGGGATACCCGCTGAACTTAA

>OTU_306

GAAATGCGATAAGTAATGTGAATTGCAGAATTCAGTGAATCATCGAATCTTTGAACGCACATTGCGCCCGGCAGTATTCT

GCCGGGCATGCCTGTTCGAGCGTCATTTCAACCATCAAGCCCCAGCGCTTGTGTTGGgggCCTGCGGCTGCCGCAGCCcc

cTAAAAGCAGTGGCGGGCTCGCTATCACACCGAGTGCAGTAGTTCATCTTCtctcCGGTCTTGTGAGGCGGGTTCCGGCC

GTGAAACACCCATCATACTCAAGGTTGACCTCGGATCAGGTAGGAATACCCGCTGAACTTAA

>OTU_307

GAAATGCGATAAGTAATGTGAATTGCAGAATTCAGTGAATCATCGAATCTTTGAACGCACATTGCGCCCGCCAGCATTCT

GGCGGGCATGCCTGTTCGAGCGTCATTTCAACCCTCAAGCTCCGCTTGGTGTTGGGGCCCTACAGCTGATGTAGGCCCTC

AAAGGTAGTGGCGGACCCTCCCGGAGCCTCCTTTGCGTAGTAACTTTACGTCTCGCACTGGGATCCGGAGGGACTCTTGC

CGTAAACCccccAATTTTCCAAAGGTTGCACCTCGGATCAGGTAGGAATACCCGCTGAACTTAA

>OTU_308

AAAGTGCGATAACTAGTGTGAATTGCATATTCAGTGAATCATCGAGTCTTTGAACGCAACTTGCGCTCATTGGTATTCCA

ATGAGCACGCCTGTTTCAGTATCAAAACAAACCCTCTATCCAACTTTTGTTGAATAGGATGACTGAGAGTCTCTTGATCT

TTGATCTCGAACCTCTTGAAATATACAAAGGCCTGATCTTGTTTAAATGCCTGAACTTtttttAATATAAAGAGAAGCTC

TTGCGATAAACTGTGCTGGGGCCTCCCAAATAACACTTtttAAATTTGATCTGAAATCAGGTGGGATTACCCGCTGAACT

TAA

>OTU_309

GAAATGCGATAAGTAATGTGAATTGCAGAATTCAGTGAATCATCGAATCTTTGAACGCACATTGCGCCcccTGGTATTCC

GGggggCATGCCTGTTCGAGCGTCATTACAACCCTCAAGCTCTGCTTGGTATTGGGCCCCGCCGGTTCCGGCAGGCCTTA

AAATCAGTGGCGGTGCCATTCGGCTTCAAGCGTAGTAATTCTTCTCGCTTCGGAGACCCGGGTGCGTGCTTGCCAGCAAC

CcccAATTttttCAGGTTGACCTCGGATCAGGTAGGGATACCCGCTGAACTTAA

>OTU_310

GAAATGCGATAAGTAATGTGAATTGCAGAATTCAGTGAATCATCGAATCTTTGAACGCACATTGCGCCCCTTGGTATTCC

GGgggCATGCCTGTTCGAGCGTCATTTCAACCCTCAAGCTTAGCTTGGTATTGAGTCTATGTCAGTAATGGCAGGCTCTA

AAATCAGTGGCGGCGCCGCTGGGTCCTGAACGTAGTAATATCtctcGTTACAGGTTCTCGGTGTGCTTCTGCCAAaaaCC

CAAATTttttCTATTGGTTGACCTCGGATCAGGTAGGGATACCCGCTGAACTTAA

>OTU_311

GAAATGCGATAAGTAATGTGAATTGCAGAATTCCGTGAATCATCGAATCTTTGAACGCACATTGCGCCCTCTGGTATTCC

GGAGGGCATGCCTGTCCGAGCGTCATTGCAACCTTCAAGCGCGGCTTGtgtgtTGGGCCTCGTCCcccGTGGACGGGCCT

CAAAGGCAGTGGCGGCGTCCGTTTTGGTGCCCGAGCGTATGGGAATTCTATACCGCTTCAAGGCCCGGCGGCGCTGGTCA

AGACCAATTtttATCGGTTGACCTCGGATCAGGTAGGGATACCCGCTGAACTTAA

>OTU_312

GAAATGCGATAAGTAGTGTGAATTGCAGAATTCAGTGAATCATCGAATCTTTGAACGCACATTGCGCCCATTAGTATTCT

AATGGGCATGCCTGTTCGAGCGTCATTTCAACCCTCAAGCCCTAGTTGCTTGGTGTTGGGAGCTTATCCTGCCGAAAGGT

GGGACAACTCCTTAAAACTATTGGCGGAGTCGCGGTGACCCCAAGCACAGTAATTCCTTTTCTCGCTTTAGGTGTTAACG

CTGGCCTCTAGCCATTAAACCCTTCTATTTTCTCAATGGTTGACCTCGGATCAGGTAGGACTACCCGCTGAACTTAA

>OTU_313

GAAATGCGATAAGTAATGTGAATTGCAGAATTCAGTGAATCATCGAATCTTTGAACGCACCTTGCGCTCCTTGGTATTCC

GAGGAGCATGCCTGTTTGAGTGTCATTAAATTCTCAACCTCACCAGTTTTCTGAACTGACTGAGGCTTGGATGTGGgggT

TTGTGCAGGCTGCCTCACCGGCGGTCTGCTCCCCTGAAATGCATTAGCGAGGTTCATGCTGGACCTCCGTCTATTGGTGT

GATAATTATCTACGCCGTGGACGAGGATACAGACTCGCTTCTAACCGTCCGCGAGGACAATACCTTGACAATTTGACCTC

AAATCAGGTAGGACTACCCGCTGAACTTAA

>OTU_314

GAAATGCGATAAGTAATGTGAATTGCAGAATTCAGTGAATCATCGAATCTTTGAACGCACATTGCGCCCCTTGGTATTCC

GAGGGGCATGCCTGTTCGAGCGTCATTATGACCAATCAAGCCTGGCTTGGTGTTGGGGTTCGCGGTTTCGCGGCTCTTAA

AATCAGTGGCAGTGCCGCCTGGCTCTAAGCGTAGTAATTCtctcGCTATTGGGTCTGGTGGTGACTTGCCAGAAATTAAT

CTCACGGTTGACCTCGGATCAGGTAGGGATACCCGCTGAACTTAA

>OTU_315

GAAATGCGATACGTAATGTGAATTGCAGAATTCAGTGAATCATCGAATCTTTGAACGCATATTGCGCTCTTTGGTATTCC

GAAGAGCATGCTTGTTTGAGTATCAGTAAACACCTCAAAGCTTTTGGATTttttAATCGAAAAGCTTTGGACTTGAGCAA

TCCCAACACCAATCTTtttGAGATCGGTGGCGGGTTGCTTGAAATGCAGGTGCAGCTGGACATTCTCCTGAGCTAAAAGC

ATATTCATTTAGTCCCGTCAAACGGATTATTACTTTTGCTGCAGCTAACATAAAGGGAGTTTGACCGTATTGGCTGACTG

ATGCAGGATTTCACAAGGGTCGGCAACGATTCTTGTTAAACTCGATCTCAAATCAAGTAAGACTACCCGCTGAACTTAA

>OTU_316

GAAATGCGATAAGTAGTGTGAATTGCAGAATTCAGTGAATCATCGAATCTTTGAACGCACATTGCGCCCTTTGGTATTCC

TTAGGGCATGCCTGTTCGAGCGTCATTTGAAAACTCAAGCTCAGCTTGGTGATGGGTGTCTGTCTCCGCCCCGCGGGGAC

TCGCCTCAAAAGCATTGGCGGCCGGTACGTTGGCTTCGAGCGCAGCAGAAACGCGAACTCGGGGCTGGCGGATCGGCGAC

CAGAAGCATCTTCTTTACAGCTTGACCTCGGATCAGGTAGGGATACCCGCTGAACTTAA

>OTU_317

GAAATGCGATAAGTAATGTGAATTGCAGAATTCAGTGAATCATCGAATCTTTGAACGCACATTGCGCCCCTTGGTATTCC

GAGGGGCATGCCTGTTCGAGCGTCATTACACCACTCAAGCTATGCTTGGTGATTGGGCGTCGTCCTTAGTTGGGCgcgcC

TTAAAGACCTCGGCGAGGCCACTCCGGCTTTAGGCGTTAGTAGAATTTATTCGAACGTCTGTCAAAGGAGAGGAACTCTG

CCGAACTGAAACCTTTTATTttttCTTAGGTTGACCTTCGGATCAGGTAGGGATACCCGCTGAACTTAA

>OTU_318

GAAATGCGATAAGTAATGTGAATTGCAGAATTCAGTGAATCATCGAGTCTTTGAACGCACCTTGCGCCCTCTGGTATTCC

GGAGGGCATGCCTGTTTGAGTGTCATGAAAACCTCAATCCCTCGGGTTtttACGAACCCGTTGGACTTGGATTTGGGCTC

TGCCGCGACCTCTCGGGGACGTCGGCTCGCCTTAAAGATGTTAGTGAGAAGGGTGGCTCCCGTCAGCCTGGCGTAATAAG

TTTCGCTGGGCCCCTGGGGATGCCGTTCGGCTCGCTCATAACGACCAACTTTCTTAACACTCTGACCTCAAATCAGGTAG

GACTACCCGCTGAACTTAA

>OTU_319

GAAATGCGATAAGTAGTGTGAATTGCAGAATTCAGTGAATCATCGAATCTTTGAACGCACATTGCGCCCCTTGGTATTCC

ATGGGGCATGCCTGTTCGAGCGTCATTTGTACCCTCAAGCTTTGCTTGGTGTTGGGCGTTTTGTCCCGCGTTGCGCGTGG

ACTCGCCTTAAAGCGATTGGCAGCCGGCATATTTGGCCTTGGAGCGCAGCACATTTTGCGCTTCTCGTCATGAATGCGGG

CGTCCATTAAGCCTATACATTTAACTCTTGACCTCGGATCAGGTAGGGATACCCGCTGAACTTAA

>OTU_320

GAAATGCGATAAGTAATGTGAATTGCAGAATTCAGTGAATCATCGAATCTTTGAACGCACATTGCGCCCCTTGGTATTCC

GGggggCATGCCTGTTCGAGCGTCATTACAACCCTCAAGCCTGGCTTGGTATTGGGCTTCGCCGTTTTGGCGGGCCTTAA

AATCAGTGGCGGTGCCGTCGAGGCCCTGAGCGTAGTAAATATCCTCGCTATAGGGACCCGGTGGTTGCTAGCCATGAACC

cccAACTTTCTAAGTTTGACCTCGGATCAGGTAGGGATACCCGCTGAACTTAA

>OTU_321

GAAATGCGATAAGTAATGTGAATTGCAGAATTCAGTGAATCATCGAATCTTTGAACGCACATTGCACCCTCTGGTATTCC

GGggggTATGCCTGTTCGAGCGTCATTGCAACCCTCAAGCATTGCTTGGTATTGGATGCCACTGTGTTGGTGCATCTCAA

AAGTATTGGTGGTGGCATTCAGCTTCTAGCGTAGTAAATCTTCTCGCCTGGAGTTTGACTGTCTGACTGCCAGAACCCCT

AAATTTATCAAAGGTTGACCTCGGATCAGGTAGGGATACCCGCTGAACTTAA

>OTU_322

GAAATGCGATAAGTAATGTGAATTGCAGAATTCAGTGAATCATCGAATCTTTGAACGCACATTGCGCCCGCCAGTATTCT

GGCGGGCATGCCTGTTCGAGCGTCATTTCAACCATCAAGCCcccGGGCTTGTGTTGGGGACCTGCGGCTGCCGCAGGCCC

TGAAAAGCAGTGGCGGGCTCGCTGTCACACCGAGCGCAGTAGATTTTATCTCGCTCTGGGCGTGCTGCGGGTTCCGGCCG

TTAAACCACCCTTTTAACCCAAGGTTGACCTCGGATCAGGTAGGAAGACCCGCTGAACTTAA

>OTU_323

GAAATGCGATAAGTAGTGTGAATTGCAGAATTCAGTGAATCATCGAATCTTTGAACGCACATTGCGCCCCTTGGTATTCC

ATGGGGCATGCTTGTTCGAGCGTCATTTGTACCCTCAAGCTTTGCTTGGTGTTGGGTGTTTGTCCTTTCCCTGCGTCAGG

ACTCGCCTTAAATTCATTGGCAGCCGATGTATTGGTTTGAAGCGCAGCACAATTTGCGACTCAGGCTGGTGACCATGGGC

GTCCAGTAAGCACATTTAACACTTTTGACCTCGGATCAAGTAGGGATACCCGCTGAACTTAA

>OTU_324

GAAACGCGATAAGTAATGTGAATTGCAGAATCCAGTGAATCATCGAATCTTTGAACGCACATTGCGCCCGCCAGCATTCT

GGCGGGCATGCCTGTTCGAGCGTCATTTCAACCCTCGACCTCCCCTTGGgggggTCGGCGTTGGGGACCGGCAGCACACC

GCCGGCCCTGAAATGGAGTGGCGGCCCGTCCGCGGCGACCTCTGCGCAGTAATACAGCTCGCACCGGAACCCCGACGCGG

CCACGCCGTAAAACACCCAACTTCTGAACGTTGACCTCGAATCAGGTAGGACTACCCGCTGAACTTAA

>OTU_325

GAAATGCGATAAGTAATGTGAATTGCAGAATTCAGTGAATCATCGAATCTTTGAACGCACATTGCGCCcccTGGTATTCC

GGggggCATGCCTGTTCGAGCGTCATTTCACCACTCAAGCCTCGCTTGGTATTGGGCAACGCGGTCCGCCGCGTGCCTCA

AATCGACCGGCTGGGTCTTCTGTCCCCTAAGCGTTGTGGAAACTATTCGCTAAAGGGTGTTCGGGGAGGCTACGCCGTAA

AACAACCCATTCCTAGTTAGTACCTCGGATCAGGTAGGGATACCCGCTGAACTTAA

>OTU_326

GAAATGCGATAAGTAATGTGAATTGCAGAATTCAGTGAATCATCGAATCTTTGAACGCACATTGCGCCCGCCGGCACTCC

GGCGGGCATGCCTGTCCGAGCGTCATTTCAACCCTCAGGCCCACCCTTCCGGgggAGCGGGCCTGGTTCTGGGGATCGGC

GGCCCTCGCGGCCcccGTCCCTCAAATTCAGTGGCGGTCGCGCCGCAGCCTCCCCTGCGTAGTAGCACAACCTCGCACCG

GAGAGCGGAACGACCACGCCGTAAAACACCCAATTttttAAGGTTGACCTCGGATCAGGTAGGAATACCCGCTGAACTTA

A

>OTU_327

AAAATGCGATAACTAATGTGAATTGCAAATTACGTGAATCATCGAGTCTCTGAACGCAAATTGCACCCTGTGGTATCCCG

CAGGGTATACCTGTTTGtgtgCCGTCTCCCTCGGGCCTTTGGAAAAGGGTCTCGAACTAGTGGCCTTTGTGTCCACTGTG

CGGGCTGCAGTCTCGTATCGCAGCCTGTTAGCGAATTCCAGAGCTTTCTCTACCAGATGCTTTTGCATCTTTGTGACCTC

ATCTGGTAAAGGGCAGCAGGAGCAAGTGTAAAGCTTGTGCTTCATGTTTCTCCTTTCCCTTCGGGGTGGAGTTACCTATT

TCTCAAGGCTACAAATCAGGCAGGATTACCCGCTGAACTTAA

>OTU_328

GAAATGCGATAAGTAATGTGAATTGCAGAATTCAGTGAATCATCGAATCTTTGAACGCACATTGCGCCCGCCAGCATTCT

GGCGGGCATGCCTGTTCGAGCGTCATTTCAACCCTCGACGTCCCCTGGgggATGTCGGCGTTGGGGACCGGCAGCACACC

GCCGCCcccGAAATGAAGTGGCGGCCCGTCCGCGGCGACCTCTGCGTAGTACCCCAACTCGCACCGGGAACCCGACTGGC

CACGCCGTAAAACGCCCAACTCTGAACGTTGACCTCGGATCAGGTAGGAATACCCGCTGAACTTAA

>OTU_329

GAAATGCGATAAGTAGTGTGAATTGCAGAATTCAGTGAATCATCGAATCTTTGAACGCACATTGCGCCCTTTGGTATTCC

AAAGGGCATGCCTGTTCGAGCGTCATTTGTACCCTCAAGCTTTGCTTGGTGTTGGGCGTCTTGTCTCTAGCTTTGCTGGA

GACTCGCCTTAAAGTAATTGGCAGCCGGCCTACTGGTTTCGGAGCGCAGCACAAGTCGCACTctctATCAGCAAAGGTCT

AGCATCCATTTAAGCCTTtttttCAACTTTTGACTCGGATCAGGTAGGGATACCCGCTGAACTTAA

>OTU_330

GAAATGCGAAAAGTAGTGTGAATTGCAGAATTCAGTGAATCATCGAATCTTTGAACGCACATTGCGCCCCTTGGTATTCC

ATGGGGCATGCCTGTTCGAGCGTCATTTGTATCTTCAAGCTCTGCTTGGTGTTGGGTGTTTGTCCCGCTTTGTGCGTGGA

CTCGCCTTAAAGCAATTGGCAGCCGGCGTACTAGTCTGGGAGCGCAGCACAATTTGCGCTTCTGAACCTGAACGCTTGCG

TCCATGAAGTCTATACTTTTGCTTTTGACCTCGGATCAGGTAGGGATACCCGCTGAACTTAA

>OTU_331

GAAATGCGATAAGTAGTGTGAATTGCAGAATTCAGTGAATCATCGAATCTTTGAACGCACATTGCGCCCATTGGTATTCC

AATGGGCATGCCTGTTCGAGCGTCATTTGTACCCTCAAGCTTTGCTTGGTGTTGGGCGTTTGTCTTTCGAGACTCGCCTT

AAaaaGATTGGCAGCCGGCATACTGGTTTCGGAGCGCAGCACAAATTGCGGTCTGTTCCTGAATGTTGACGTCCATGAAG

CCcccATTATCACTTTTGACCTCGGATCAGGTAGGGATACCCGCTGAACTTAA

>OTU_332

GAAATGCGATAAGTAATGTGAATTGCAGAATTCAGTGAATCATCGAATCTTTGAACGCACATTGCGCCTAGCGGTATTCC

GCTAGGCATGCCTGTTCGAGCGTCATTTCAACCCTCAAGCCTAGCTTGGTATTGGGTGTCACCGGCACGGTGCGCCTTAA

AATCAGTGGCGGTGCCATCTGGCTTCAAGCGTAGTAATACTTCTCGCTCTGGATCCCGGGTGGTTACTTGCCAACAACCC

CATATTttttAAGGTTGACCTCGGATCAGGTAGGGATACCCGCTGAACTTAA

>OTU_333

GAAATGCGATAAGTAGTGTGAATTGCAGAATTCAGTGAATCATCGAATCTTTGAACGCACATTGCGCCCTTTGGTATTCC

AAAGGGCATGCCTGTTCGAGCGTCATTTGTACCCTCAAGCTTTGCTTGGTGTTGGGCGTCTTGTCTCTAGCTTTGCTGGA

GACTCGCCTTAAAGTAATTGGCAGCCGGCCTACTGGTTTCGGAGCGCAGCACAAGTCGCACTctctATCAGCAAAGGTCT

AGCATCCATTAAGCCTTttttttCAACTTtttGACCTCGGATCAGGTAGGGGATACCCCGCTGAACTTAA

>OTU_334

GAAATGCGATAAGTAATGTGAATTGCAGAATTCAGTGAATCATCGAATCTTTGAACGCACATTGCGCCCTTTGGTATTCC

AAAGGGCATGCCTGTTCGAGCGTCATAAAATCAATCTCAAGCCTAGAAGCTTGGTGTTGGGTTGTCATGACTGCCCTTGC

ATTGGGTTGGTTATGTACCTGAAAATTAGTGGCAGTGCCTAAAGTGACCTCAAGCATAGTGGACACTCTTGTGCTTTGGA

GGCTTCATTAAGGATTGCTTGCCTTTTAACTGCTTTTGACAAAGGTTTGACCTCGGATCAGGTAGGGATACCCGCTGAAC

TTAA

>OTU_335

GAAATGCGATAAGTAATGTGAATTGCAGAATCCAGTGAATCATCGAATCTTTGAACGCACATTGCGCCCGCCAGTATTCT

GGCGGGCATGCCTGTCCGAGCGTCATTTCGACCATCAAGCCGTAGGCTTGTGTTGGAGCACTGCGGGCTGCCCGCAGGCT

CTGAAATACAGTGGCGGCCCTGTAGGCTTCCCGGGCGTAGTACTTGAAACACTCGTCTAGGGAGGGCTTGCAGCGCCTGC

TGACCCAAATTTTACAAGGTTGACCTCGGATCAGGTAGGAATACCCGCTGAACTTAA

>OTU_336

GAAATGCGATAAGTAATGTGAATTGCAGAATTCAGTGAATCATCGAATCTTTGAACGCACATTGCGCCCCTTGGTATTCC

GAGGGGCATGCCTGTTCGAGCGTCATTACACCACTCAAGCACAGCTTGGTATTGGGCGTCCGTCCccccAGGggggCgcg

cCTCAAAGACCTCGGCGAGGCCTCACCGGCTTTAGGCGTAGTAGAATTTATTCGAACGTCTGTCAAAGGAGAGGACTTCT

GCCGACTGAAACCTTTAAATTtttttCTAGGTTGACCTCGGATCAGGTAGGGATACCCGCTGAACTTAA

>OTU_337

GAAATGCGATAAGTAGTGTGAATTGCAGAATTCAGTGAATCATCGAATCTTTGAACGCACATTGCGCCCTATGGTATTCC

GTAGGGCATGCCTGTTCGAGCGTCATTCAAAACTTCAAGCTCTGCTTGGTGATGGGTGCCTGTCTTGCTTTATAGCGTGG

ACTCACCTCAAATACATTGGCGGCCGGCACGTTGGCTTCGAGCGCAGCAGAAACGCGAACTCGTCCCCAGTGGATCGGCT

CCCAGAAGCTAAaaaCCCATGAATTTGACCTCGGATCAGGTAGGGATACCCGCTGAACTTAA

>OTU_338

GAAATGCGATAAGTAATGTGAATTGCAGAATTCAGTGAATCATCGAATCTTTGAACGCACATTGCGCCCTCTGGTATTCC

GGggggCATGCCTGTTCGAGCGTCATTACAACCCTCAAGCTCTGCTTGGTGTTGGGCCTCGCCGGTTCCGGCGGGCCTCA

AAATCAGTGGCGGTGCCATCTGGCTTCAAGCGTAGTAATTCTTCTCGCTTCAGGAGACCCAGGTgtgtgCTTGCCAGCAA

CCcccAACTTCTATGGTTGACCTCGGATCAGGTAGGGATACCCGCTGAACTTAA

>OTU_339

GAAATGCGATAAGTAATGTGAATTGCAGAATTCAGTGAATCATCGAATCTTTGAACGCAACTTGCGCTctctGGTATTCC

GGAGAGCATGCCTGTTTGAGTGTCATGAAATCTCAACCATTAGGGTTTCTTAATGGCTTGGATTTGGGTGTTGCCAGTCT

CTGGCTCGCCTTAAAGGAGTTAGCGAGTTTAACAATGTCGTCTGGCGTAATAAGTTTCGCTGGTAAGACTTGTGAAGTTT

GCTTCTAATCGTCTTCGGACAATTACTTTGACTCTGGCCTCAAATCAGGTAGGACTACCCGCTGAACTTAA

>OTU_340

GAAATGCGATAAGTAATGTGAATTGCAGAATTCAGTGAATCATCGAATCTTTGAACGCACATTGCGCCcccTGGTATTCC

GGggggCATGCCTGTTCGAGCGTCATTTCAACCCTCAAGCTCTGCTTGGTATTGGGCGTCGCCGGGGAGTCTTCTCCGGC

GTGCCTCAAAGCTATCGGCGGTGGTGCCTTTCTCTAAGCGTGGTAGAATTtttATTCGCTTCTGAGTTGGGCTTCGCCTG

CCGGACAAACCGGGTCCTCGCGGCCCATCTTCTCAGGTTGACCTCGGATCAGGTAGGGATACCCGCTGAACTTAA

>OTU_341

GAAATGCGATACTTGGTGTGAATTGCAGAATCCCGTGAACCATCGAGTCTTTGAACGCAAGTTGCGCCCGAAGCCATCCG

GCCGAGGGCACGTCTGCCTGGGCGTCAcacacCGTTGCCcccccAAACCCCGTCGGGGAAaaaGGggggCGGATGCTGGC

CTCCCGTGAGCCTCGCCTCGCGGCTGGCCCAAATGCGAGATCTCTGCTGCGAGCGTCGCGGCGATGGTGGTTGTCGAATA

ACTCGGTGCCCCGTCGCGAACGCCCGCAGCGTGCTGTCTCGGAACGACCCCTGCgcgcgGCCTCGCGCCGCGCTTCCAAC

GCGACCCCAGGTCAGGCGGGGCTACCCGCTGAATTTAA

>OTU_342

GAAATGCGATAAGTAATGTGAATTGCAGAATTCAGTGAATCATCGAATCTTTGAACGCACATTGCGCCCGCCAGTATTCT

GGCGGGCATGCCTGTTCGAGCGTCATTACAACCCTCAGGCCcccGGGCCTGGCGTTGGGGATCGGCGGAAGCCcccTGTG

GGCACACGCCGTCCCTCAAATACAGTGGCGGTCCCGCCGCAGCTTCCATTGCGTAGTAGCTAACACCTCGCAACTGGAGA

GCGGCGCGGCCATGCCGTAAAACACCCAACTTCTGAATGTTGACCTCGAATCAGGTAGGAATACCCGCTGAACTTAA

>OTU_343

GAAATGCGATAAGTAATGTGAATTGCAGAATTCAGTGAATCATCGAATCTTTGAACGCACATTGCGCCCGCCAGCATTCT

GGCGGGCATGCCTGTTCGAGCGTCATTTCAACCCTCAAGCTCCGCTTGGTGTTGGGGCCCTACGGCGTACGTCGTAGGCC

CTTAAAGGTAGTGGCGGACCCTCCCGGAGCCTCCTTTGCGTAGTAACTAACGTCTCGCATCGGGATCCGGAGGGACTCTT

GCCGTAAAACCcccAACTTtttAACTGGTTGACCTCGGATCAGGTAGGAATACCCGCTGAACTTAA

>OTU_344

GAAATGCGATAAGTAATGTGAATTGCAGAATTCAGTGAATCATCGAATCTTTGAACGCACATTGCGCCCCTTGGTATTCC

GGggggCATGCCTGTTCGAGCGTCATTTCAACCCTCAAGCTTAGCTTGGTATTGAGTCTATGTCAGTAATGGCAGGCTCT

AAAATCGGTGGCgcgcCGCTGGGTGCCTGAACGTAGTAATATCtctcGTTACAGGTTCTCGGTGTGCTTCTGCCAAAACC

CAAATTtttCTATGGTTGACCTCGGATCAGGTAGGGATACCCGCTGAACTTAA

>OTU_345

GAAATGCGATAAGTAATGTGAATTGCAGAATTCAGTGAATCATCGAATCTTTGAACGCACATTGCGCCCTTTGGTATTCC

GAAGGGCATGCTTGTTCGAGCGTCATTTCAAACCTCAAGCTTCTGCTTGGTATTGGGCCTCGTCCGTCTAGGACGTGCCA

GAAACCCGTGGGCGGTGGTGCCGGTCTCCCAGCGTAGTCAATTCTACCCGCTTCGGAGCTTCAGGTGCTGCCCGCCGGAC

AACTCCAAATTtttCTATGGTTTGACCTCGGATCAAGTAGGGATACCCGCTGAACTTAA

>OTU_346

GAAATGCGATAAGTAATGTGAATTGCAGAATTCCGTGAATCATCGAATCTTTGAACGCACATTGCGCCCCTCGGTATTCC

GGggggCATGCCTGTTCGAGCGTCATTACACCAATCAAGCCCCGGCTTGGTCTTGGGCGTCGCGGTCTCCCGCGTGCCTC

AATGTCGCCGGCTGGTGCGTCCGTCTCCGAGCGTCGTGACTTCATTGCTCGCTTCTGGAGGTCGGACGGACTGCGCCGTC

AACCccccATATCTACAGGTTGACCTCGGATCAGGTAGGGATACCCGCTGAACTTAA

>OTU_347

GAAATGCGATAAGTAATGTGAATTGCAGAATTCAGTGAATCATCGAATCTTTGAACGCACATTGCGCCcccTGGTATTCC

GGggggCATGCCTGTTCGAGCGTCATTTCAACCAATCCAGCCCGGCTGGGTGTTGGGCGTCGCGGTCCTCCCCGcgcgcC

TCAAAGTCCTCGGCGGAAGCCGCCCGTTCCTCTGCGTGATGACACATCTTCGCTCGGGAGACGGgggTGAGCGCCCGTAC

AGCATCGGCGGAGACGTCGATTTCCAGGTTGACCTCGGATCAGGTAGGGATACCCGCTGAACTTAA

>OTU_348

GAAATGCGATAAGTAATGTGAATTGCAGAATTCAGTGAATCATCGAATCTTTGAACGCACATTGCGCCCGCCAGCATTCT

GGCGGGCATGCCTGTTCGAGCGTCATTTCAACCCTCAAGCTCTGCTTGGTGTTGGGGCTCTACGGTTGACGTAGGCCCTT

AAAGGTAGTGGCGGACCCTCTCGGAGCCTCCTTTGCGTAGTAACATTTCGTCTCGCATTGGGATTCGGAGGGACTCTAGC

CGTAAAACCcccAATTTTACTAAGGTTGACCTCGGATCAGGTAGGAATACCCGCTGAACTTAA

>OTU_349

GAAATGCGATAAGTAATGTGAATTGCAGAATTCAGTGAATCATCGAATCTTTGAACGCACCTTGCGCCTTTTGGTATTCC

GAAAGGCATGCCTGTTTGAGTGTCATGAAACATCAACCccccAGGTTtttCGCTTGGGAAGGTTGGATCTGGGCGCTGCC

GCCTGCTCGTCTGCGGGCCGGCTCGCCTCAAAAGCGTTAGTGGGATACCGCATAGCCCGACGTGATAAGTTTCGTCTGGT

GAAGCGCGACGCCTGCTTTACCCCAATTTCATTTGTACTCTGACCTCAAATCAGGTAGGACTACCCGCTGAACTTAA

>OTU_350

GAAATGCGATAACTAATGTGAATTGCAGAATTCAGTGAATCATCGAGTCTTTGAACGCACATTGCGCCCTCTGGTATTCC

GGAGGGCATGCCTGTCCGAGCGTCATTGCTGCCCTCAAGCCCGGCTTGtgtgtTGGGCCCCGTCCcccccGCCGGggggA

GGGCCCGAAAGCAGCCGGCGGCACCGCGTCCGGTCCTCGAGCGTATGGGGCTTCGTCACCCGCTCTAGTAGGCCCGGCCG

GCGCCAGCCGACCcccAACCTTTAATTATCTCAGGTTGACCTCGGATCAGGTAGGGATACCCGCTGAACTTAA

>OTU_351

GAAATGCGATAAGTAGTGTGAATTGCAGAATTCAGTGAATCATCGAATCTTTGAACGCACATTGCGCCTCCTGGTATTCC

GGGAGGCATGCCTGTTCGAGCGTCATTAAaaaCCACTCAAGCTCTTTTGCTTGGTATTGGGAGATGAGTATACTTTTCCT

AGTTTACTCACTCTCGAAATTCAAAGGCGTAGAGCCCCACATACCACAGCGTAGTAAGTTTTCTTTCGCTTGGAATGTGA

GGTGATATTGCCACAAACCcccAATTtttCTAGGTTGACCTCGGATCAGGTAGGGATACCCGCTGAACTTAA

>OTU_352

AAAATGCGATAAGTAATGTGAATTGCAGAATACGTGAATCATCAAATCTTCGAACGCATATTGCACTTTTCTAGTTTACT

AGAAAAGTATGTCTGTTTGAGTATCAGTAAAATATTCTCATAAaaaCATTGTTtttATGGTTATGAGTGTTTTACAACGA

TAAACAAGTTGTTTCACTTTAAATTGATTtttAAAAGCCAATTtttGGTTTTATCTAATAAAATTGGATAAAATTTTCCC

GTAAaaaGGAATAACTTTTCAGTTTTAATAGTTAAAACTTAATTGTGCCTAATTAAAAGGAAATTTAACTAAATAACTTG

ATCTCAAATCAGATAAGAGTACCCGCTGAACTTAA

>OTU_353

GAAATGCGATAATTAATGTGAATTGCAGAATTCAGTGAATCATCGAGTCTTTGAACGCACATTGCGCCcccTGGTATTCC

GGgggCATGCCTGTCCGAGCGTCATTGCTGCCCTCAAGCCCGGCTTGtgtgtTGGGTCTTGTCCcccccGGgggACAGGC

CCGAAAGGCAGTGGCGGCACGTCCGATCCTCGAGCGTATGGGGCTTTGTCACCCGCTCTGTAGGCCCGGCCGGCTGCTTG

CCGACCcccATCAATCTTtttttCAGGTTGACCTCGGATCAGGTAGGGATACCCGCTGAACTTAA

>OTU_354

AAAATGCGATAAGTAATGTGAATTGCAGAATTCAGTGAATCATCGAATCTTTGAACGCACATTGCGCCCGCCAGTATTCT

GGCGGGCATGCCTGTCTGAGCGTCATTTCAACCCTCGGGACCCCGTTCGCGGGACCCGGCGTTGGGGATCAGCCCGGAGC

CCTCTGCGGCCGGCGGCTGGCCCCGAAATCTAGTGGCGGTCTCCCCTGCGACCTCCTCTGCGTAGTAGTAACTCACCTCG

CAGCTGGACAGCGGGAAGGCCACGCCGTAAAACCccccAACTCTTCTAAGGTTGACCTCAGATCAGGTAGGAATACCCGC

TGAACTTAA

>OTU_355

GAAATGCGATAAGTAATGTGAATTGCAGAATTCAGTGAATCATCGAATCTTTGAACGCACATTGCGCCcccTGGTATTCC

GGggggCATGCCTGTTCGAGCGTCATTTCAACCAATCCAGCCCGGCTGGGTGTTGGGCGCCGCGGTTCGCCGcgcgcCTC

AAAGTCCTCGGCGGAAGCCGCCCGTTCCTCTGCGTGATGATCCATCCTCGCTCGGGAGACGGgggCGAGCGCCCGTACGC

GCCGGCGGAGACGCCGACTTCCAGGTTGACCTCGGATCAGGTAGGGATACCCGCTGAACTTAA

>OTU_356

GAAATGCGATAAGTAATGTGAATTGCAGAATTCAGTGAATCATCGAATCTTTGAACGCACATTGCGCCTCTGGAACTTCC

ATCCCGGAGGCATGCCTGTTCGAGCGTCCTTACAAaaaaCCTCAAGCCGTAGGCTTGTGTTGGAGCACTGCGGGCTGCCC

GCAGGCTCTGAAATACAGTGGCGGCCCTGTAGGCTTCCCGGGCGTAGTACTTGAAACACTCGTCTAGGGAGGGCTTGCAG

CGCCTGCTGACCCAAATTTTACAAGGTTGACCTCGGATCAGGTAGGAATACCCGCTGAACTTAA

>OTU_357

GAAATGCGATAAGTAATGTGAATTGCAGAATTCAGTGAATCATCGAATCTTTGAACGCACCTTGCGCCTTTTGGTATTCC

GAAAGGCATGCCTGTTTCAGTGTCATGAAATCTCAATCTAATATGTTTTCTGAACATGTTAGGCTTGGACTTGGGTGTCT

GCCAGCAATGGCTCACCTCAAATGACTTAGTGGAACATCCCACATCAGTGTTAGACGTAATAAGTTTCGTCtctcCTTGT

GGTGATGACTGCTCAAAACCTGCCATCGCTGCACCTTTTGACTTTGACCTGAATTACGGTGGGCTAACCGCTGAACTTAA

>OTU_358

GAAATGCGATACTTGGTGTGAATTGCAGAATCCCGTGAACCATCGAGTTtttGAACGCAAGTTGCGCCCAAAGCCACTCG

GCCGAGGGCACGTCTGCCTGGGCGTCACGTATCGCGTCGCCTCCAAAGCCCTCCCACCCGTGGGAAGGTGCTGCGGAAGC

GGAGATTGGCCTCCCGTGCTGATGCACGGCTGGCCTAAATGCAAGCCTGGgggTGACGAAAGGTCACGACAAGCGGTGGT

TGATACCTCGGCCCTTGTTGCGTCGTGCCCGCCCGTGCCTCTTGGAGCTCATCATCCCCGACGCTACGCCCCTATGGCGT

TTGCTTCGCGACCCCAGGTCAGGCGGGACTACCCGCTGAGTTTAA

>OTU_359

GAAATGCGATAAGTAGTGTGAATTGCAGAATTCAGTGAATCATCGAATCTTTGAACGCACATTGCGCCCCTTGGTATTCC

ATGGGGCATGCCTGTTCGAGCGTCATTTGTACCCTCAAGCTCTGCTTGGTGTTGGGTGTTTGTCCTCTCCCCTGTGTTTG

GACTCGCCTTAAAGCAATTGGCAGCCAGTGTTTTGGTATTGAAGCGCAGCACATTTTGCGCCTTGGGCCGATGACACTGG

CGTCCACAAAGCCTCTTtttACACTTTTGACCTCGGATCAGGTAGGGATACCCGCTGAACTTAA

>OTU_360

GAAATGCGATACGTAATGTGAATTGCAGAATTCAGTGAATCATCGAATCTTTGAACGCACATTGCGCCCATTAGTATTCT

AGTGGGCATGCCTGTTCGAGCGTCATTTCAACCCTTAAGCCTAGCTTAGTGTTGGGAATCTGTCCGCAAGGACAGTTCCT

CAAATCCAACGGCGGATCTGTGGTATCCTCTGAGCGTAGTAAATCTTTATCTCGCTTTTGTCAGGTGCCGCTGCTCTCAG

CCGCTAAACCccccAATTttttGTGGTTGACCTCGGATCAGGTAGGAATACCCGCTGAACTTAA

>OTU_361

GAAATGCGATAAGTAGTGTGAATTGCAGAATTCAGTGAATCATCGAATCTTTGAACGCACATTGCGCCCCTTGGTATTCC

ATGGGGCATGCTTGTTCGAGCGTCATTTGTACTCTCAAGCTATGCTTGGTATTGGGTGCTTGTCCTCCCCTTTGTAGTGG

ACTCACCTTAAATTCATTGGCAGCCAATGTATTGGTCTTGAAGCGCAGCACATTTTGCGTCTTCAGCCGATAATCATTCG

CGTCCATTAAGTATATTTAACACTTTTGACCTCGGATCAAGTAGGGATACCCGCTGAACTTAA

>OTU_362

GAAATGCGATAAGTAGTGTGAATTGCAGAATTCAGTAAATCATCAAATCTTTAAACGCACATTGCGCCCTTTGGTATTCC

TTAGGGCATGCCTGTTCAAGCGTCATCTAAACCTTCAAGCACTGCTTGGTGTTAGGTGCCTGTCCCGCCcccGcgcgTAG

ACTCACCTCAAATCCATTGGCGGCCCTCACGTCAGCTACGAGCGCAGCAGAAACGCGAACTTGTGGACCCAGCGGAGTGG

CTCCCAGAAGCTACATTCACCATTTTAACCTTGGATCAGGTAGAGATACCCGCTGAACTTAA

>OTU_363

GAAATGCGATAAGTAATGTGAATTGCAGAATTCAGTGAATCATCGAATCTTTGAACGCACCTTGCGCCTTTTGGTATTCC

GAAAGGCATGCCTGTTTGAGTGTCATGAAATCTCAATCCTGCCGGGTTTTGTTACCCGGTGCTGGACTTGGACCTGGGCG

TCTGCCAGTCAAATGGCTCGCCTCAAATGACTTAGTGGGATCTCCTAGACATCCGCGACAGACGTAATAAGTTTCGTCTC

GTCCCTTGTCTGACCGAGCCTGCTCACAACCCGCCATCGCGCACCTTtttGACTCTGACCTCAAATCAGGTAGGACTACC

CGCTGAACTTAA

>OTU_364

GAAACGCGATATGTAATGTGAATTGCAGAATTCAGTGAATCATCGAATCTTTGAACGCACATGGCGCCTTCCAGTATCCT

GGGAGGCATGCCTGTCCGAGCGTCGTTTCAACCCTCGAGCCcccGTGGCCCGGCGTTGGGGACCTGCCCAGGCAGTCCCC

GAAAACCAGTGGCGGACCCGACGGGCCCTTCCTTTGCGTAGTAACATCTGCCTCGCATCGGGAGCCcccGGGCTATCCGG

CCTCTAAACCccccTCAAGCCCGCTCCCGGCGGCACCAAGGTTGACCTCGGATCAGGTAGGAATACCCGCTGAACTTAA

>OTU_365

GAAATGCGATAAGTAATGCGAATTGCAGAATCCGTGAGTCATCGAATCTTTGAACGCATATTGCGCCCTTTGGTATTCCG

AAGGGCATGCCTGTTCGAGCGTCATTATCACCTCTCAAGCCCCGGCTTGGCGTTGGACTTAGTTACAGGTCACCTATAAC

TGGTCTCAAAGATAGTGACGGCGCCCCAGAGGGAACCGGTACGAGGAGCTTCTAGTTAAAGCATGTACGCGGGACGACTC

TGCGGGTGACGGTCTTttttCATTTCTCCAAGTGTTGACCTCGGATCAGGTAGGAATACCCGCTGAACTTAA

>OTU_366

GAAATGCGATAAGTAGTGTGAATTGCAGAATTCAGTGAATCATCGAATCTTTGAACGCACATTGCGCCCCTTGGTATTCC

ATGGGGCATGCTTGTTCGAGCGTCATTTGTACCCTCAAGCTTTGCTTGGTGTTGGGTGTTTGTCCGTTTCTTTGCAGATG

GACTCGCCTTAAATTCATTGGCAGCTAAAATATCGCGGCAATCGCAGCACAATTTGCGGGCGCAGTAGATAATGTTTGGC

CTCCAGTAAGCGTAAATAACACTTTTGACCTCGGATCAAGTAGGGATACCCGCTGAACTTAA

>OTU_367

GAAATGCGATAAGTAATGTGAATTGCAGAATTCCGTGAATCATCGAATCTTTGAACGCACATTGCGCCCTCTGGTATTCC

GGAGGGCATGCCTGTCCGAGCGTCATTGCAACCTTCAAGCGCGGCTTGtgtgtTGGGTCTCGTCCCCGTGGACGGGCCCG

AAAGGCAGTGGCGGCGTCCGTTTCGGTGCCCGAGCGTATGGGAACTCTTATACCGCTCAACGGCCCGGCGGCGCTGGTCG

AAACCATTtttttACCGGTTGACCTCGGATCAGGTAGGGATACCCGCTGAACTTAA

>OTU_368

GAAATGCGATAAGTAATGTGAATTGCAGAATTCAGTGAATCATCGAATCTTTGAACGCACATTGCGCCcccTGGTATTCC

GGggggCATGCCTGTTCGAGCGTCATTGCAACCAATCCAGCCTGGCTGGGTGTTGGGCGTCGCGGCCcccccGcgcgcCT

CAAAGTCCCCTCGGCGGAAGCCGCCCGTTCCTCTGCGTGATGCCACATCTTCGCTCGGGACACGGgggTGAGCGCCCGGA

AAACGTCGGCGGAGACGCCGACTTCAAGGTTGACCTCGGATCAGGTAGGGATACCCGCTGAACTTAA

>OTU_369

GAATTGCGATAAGTAATGTGAATTGCAGATACTCGTGAATCATCGAATCTTTGAACGCACATTGCGCCCCTTGGTATTCC

GGggggCATGCCTGTTCGAGCGTCATTTCAACCCTCAAGCTTAGCTTGGTATTGAGTCTATGTCAGTAATGGCAGGCTCT

AAAATCAGTGGCGGCGCCGCTGGGTCCTGAACGTAGTAATATCtctcGTTACAGGTTCTCGGTGTGCTTCTGCCAAaaaC

CCAAATTtttCTATGGTTGACCTCGGATCAGGTAGGGATACCCGCTGAACTTAA

>OTU_370

GAAATGCGATAAGTAGTGTGAATTGCAGAATTCAGTGAATCATCGAATCTTTGAACGCACATTGCGGCCATAGGTATTCC

TTTGGCCATGCCTGTTCGAGCGTCATTTACACCCTCAAGCCTAGCTTGGTGTTGGGCGTCTGTCCCGCCGCTTCTCGcgc

gcgGACTCGCCTCAAAGTCATTGGCCGCGGTCGTGCCAGCCcccTCGCGCAGCACATTTTGCGCTTCTCGGAGGCTCGGC

GGATCCCGCGCTCCAGCAAGGACCTTTCACGACTTGACCTCGGATCAGGTAGGAGTACCCGCTGAACTTAA

>OTU_371

GAAATGCGATAAGTAATGTGAATTGCAGAATTCAGTGAATCATCGAATCTTTGAACGCACATTGCGCCCCTTGGTATTCC

GAGGGGCATGCCTGTTCGAGCGTCATTATAACCCCTCAAGCTCAGCTTGGTGTTGGGGCCTGCTGCACTGGCAGCCCTTA

AAATCAGTGGCGGCGCCATCTGGCTCTAAGCGTAGTAATTCTTCTCGCTATGGAGTCCTGGTGGATGCTTGCCATCAACC

cccAATTtttCTATGGTTGACCTCGGATCAGGTAGGGATACCCGCTGAACTTAA

>OTU_372

GAAATGCGATAAGTAATGTGAATTGCAGAATTCAGTGAATCATCGAATCTTTGAACGCACCTTGCGCTCTTTGGTATTCC

GAAGAGCATGCCTGTTCGAGTGTTATTAAATTCTCAAACCAATAGCTTTATTGTCTATTGGCTTTGGACTTTGAGGGTTG

TTGGTGCAAATCGACTCCCTTTAAATatatTAGCTGGCATTGACTGCCAGACTATGGTGTGATAATTATCTACGCTATTG

GTCTGACATGTTGGCTGCTTCTAATCGTCTTCGGACAACACTCTTGAAATTTAACCTCGAATCAGGTAGGACTACCCGCT

GAACTTAA

>OTU_373

GAATTGCGATAAGTAATGTGAATTGCAGAATTCAGTGAATCATCGAATCTTTGAACGCACCTTGCACTCTTTGGTATTCC

GAAGAGTATGTCTGTTTGAGTGTCATGAAACTCTCAACCccccTATTTTGTAATGAGATGGGTGTGGGCTTGGATTATGG

TTGTCTGTCGGCGTAATTGCCGGCTCAACTGAAATACACGAGCAACCCTATTGAAATAAACGGTTTGACTTGGCGTAATA

ATTATTTCGCTAAGGACGTTTTCTTCAAATATAAGAGGTGCTTCTAATTCGCTTCTAATAGCATTTAAGCTTTAGACCTC

AAATCAGTCAGGACTACCCGCTGAACTTAA

>OTU_374

GAAATGCGATAAGTAATGTGAATTGCAGAATTCAGTGAATCATCGAATCTTTGAACGCACATTGCGCCCGGTGGTATTCC

GCCGGGCATGCCTGTTCGAGCGTCATTTCACCACTCAAGCCTGGCTTGGTATTGGGCGTCGCGGAGTCCCCGcgcgcCTC

AAAGTCGTCCGGCCGAGCTGTCCGTCTCCGAGCGTTGTGACTTTACTGTTTCGCTCTCGAGGTCGGGCgcgcgGCCGCTT

AAGAGCTTCTTtttttACAGGTTGACCTCGGATCAGGTAGGGATACCCGCTGAACTTAA

>OTU_375

GAAATGCGATAAGTAGTGTGAATTGCAGAATTCAGTGAATCATCGAATCTTTGAACGCACATTGCGCCCCTTGGTATTCC

ATGGGGCATGCCTGTTCGAGCGTCATTTGTACCTTCAAGCTCTGCTTGGTGTTGGGTGTTTGTCCCGCCTCTGCGCGTGG

ACTCGCCTTAAAGCAATTGGCAGCCGGCGTATAGATTTCGGAGCGCAGCACAAAACGCGCTTTGTAACCAATACGGTGGC

GTCCAGTAAGCCTTtttttAACTCTTGACCTCGGATCAGGTAGGGATACCCGCTGAACTTAA

>OTU_376

GAAATGCGATAACTAATGTGAATTGCAGAATTCAGTGAATCATCGAGTCTTTGAACGCACATTGCGCCCTCTGGTATTCC

GGAGGGCATGCCTGTCCGAGCGTCATTGCTGCCCTCAAGCACGGCTTGtgtgtTGGGCCCCGTCCccccGCTCCGGgggg

gACGGGCCCGAAAGGCAGCGGCGGCACCGCGTCCGGTCCTCGAGCGTATGGGGCTTCGTCACCCGCTCTTGTAGGCCCGG

CCGGCGCCAGCCGACCcccAACCTTTTATTATTTCTCAGGTTGACCTCGGATCAGGTAGGGATACCCGCTGAACTTAA

>OTU_377

GAAATGCGATAAGTAATGTGAATTGCAGAATTCAGTGAATCATCGAATCTTTGAACGCACATTGCGCCCCTTGGTATTCC

GAGGGGCATGCCTGTTCGAGCGTCATTATGACCACTCAAGCCTAGCTTGGTATTGGGGTTCGCGGTCTCGCGGCCCTTAA

AATCAGTGGCGGTGCCATCTGGCTCTAAGCGTAGTAATTCATCTCGCTATTGGGTCCGGTGGTTGCTTGCCAACAACCcc

cAACTTCTAAGGTTGACCTCGGATCAGGTAGGGATACCCGCTGAACTTAA

>OTU_378

GAAATGCGATAAGTAATGCGAATTGCAGAATTCAGTGAATCATCGAATCTTTGAACGCACATTGCGCCCGCCAGTACTCT

GGCGGGCATGCCTGTCTGAGCGTCATTTCAACCCTCGCACCCGGCTTCTGTCGGgggCGGTGTTGGGGATCGGCCTCCCG

TCATCGGGCGCCGGCCCGCAATAGAGTGGCGACCACGCCGTAGCCTCCTCTGCGTAGTAGTGAAACACTCGCGGGCGGAG

AGCGGTGCGGCCCGCCGTAAAACCcccAACTCTTTCTAAGGTTGACCTCAGATCAGGTAGGAATACCCGCTGAACTTAA

>OTU_379

GAAATGCGATACTTGGTGTGAATTGCAGAATCCCGTGAACCATCGAGTCTTTGAACGCAAGTTGCGCCCGAAGCCTTCTG

GCCGAGGGCACGTCTGCCTGGGCGTCACGCATCGCGTCTCCcccAACCCACCTACTTGTAGGTAGGATGTGGgggAGGAG

GATGGCTTCCCGCGCCTCACCGGGCGTGGATGGCCTAAATTAGGAGCCTCAGGTCACGACTGCTGCGGCGATTGGTGGTC

ATCATTTCTTTCCGTGCAGTGCGTGACCAAAGTGGACTCGTAGGACCCTGTGTTGTTCCCTTTTGGAGACAAACCGTTGC

GACCCCAGGTCAGGCGGGGTTACCCGCTGAGTTTAA

>OTU_380

GAAATGCGATAAGTAGTGTGAATTGCAGAATTCAGTGAATCATCGAATCTTTGAACGCACATTGCGCCCTTTGGTATTCC

TTAGGGCATGCCTGTTCGAGCGTCATTTCAAAATTCAAGCTCAGCTTGGTGATGGGTGTCTGTCCTGCCTTGCGCGTGTG

GACTCGCCTCAAAAGCAGTTGGCAGCTTCCTCTATGGGCTCTAAACGCAGCAGACTTGCGTTGCGTGCTCTGGAGCGAGG

CTTTCCAGCAAGCCAAAACCccccGGAATCGACCTCGGATCAGGTAGGGATACCCGCTGAACTTAA

>OTU_381

GAAATGCGATACGTAATGTGAATTGCAGAATTCAGTGAATCATCGAATCTTTGAACGCATATTGCGCTCTTTGGTATTCC

GAAGAGCATGCTTGTTTGAGTATCAGTAAACACCTCAAAGCCTTCAATTCAGTTTGGAAGTTTTGGACTTGAGCAATCCC

AACACCAGTCTTGCGACTGGCGGAGGGCTGCTTGAAATGCAGGTGCAGCTGGACATTCTCCTGAGCTATAAGCATATTTA

TTTAGTCCCGTCAAACGGATTATTACTTTTGCTGCAGCTAACATAAAGGGAGTCTGACCGTATTGGCTGACTGATGCAGG

ATTTCACAAGAGCTGGCAACAGCTTCTTGTAAAACTCGATCTCAAATCAAGTAAGACTACCCGCTGAACTTAA

>OTU_382

GAAATGCGATAAGTAATGTGAATTGCAGAATTCAGTGAATCATCGAATCTTTGAACGCACATTGCGCCcccTGGTATTCC

GGgggCATGCCTGTTCGAGCGTCATTTCACCACTCAAAGCCTCGTCTTTGGTATTGGGCATCGCGGTCCGCCGCGTGCCT

CAAATCGACCGGCTGGGTCTTCTGTTCCCCTAAGCGTTGTGGAAACTATTCGCTAAAGGGTGTTCGGGAGGCTACGCCGT

TAAAACAACCCCATTTCTAAGGTTTGACCTCGGATCAGGTAGGGATACCCGCTGAACTTAA

>OTU_383

GAAATGCGATAAGTAATGTGAATTGCAGAATTCAGTGAATCATCGAATCTTTGAACGCACCTTGCGCCTTTTGGTATTCC

GAAGGCATGCCTGTTTCAGTGTCATGAAATCTCAATCTATATGTTTTCTGAACATGTTAGGCTTGGACTTGGGTGTCTGC

CAGTGATGGCTCACCTCAAATGACTTAGTGGAACATCCCACATCAGTGTTAGACGTAATAAGTTTCGTCtctcCTTGTGG

TGATGACTGCTCAGAACCTGCCATCGCGCATCTTttttGACTTGACCTGAAAATCAGGTAGGGCTACCCGCTGAACTTAA

>OTU_384

GAAATGCGATAAGTAGTGTGAATTGCAGAATTCAGTGAATCATCGAATCTTTGAACGCACATTGCGCCCCTTGGTATTCC

ATGGGGCATGCCTGTTCGAGCGTCATTTGTACCCTCAAGCTTTGCTTGGTGTTGGGCGTCTTGTCTACCTCTTTAGCGGG

TAGACTCGCCTTAAAGTAATTGGCAGCCAGTGTTtttGGCAGTAAGCGCAGCACATTTTGCGTCTTAGTCCCTAAACAGT

GGCATCCACAAAGCCTCTTTCTCACTTTTGACCTCGGATCAGGTAGGGATACCCGCTGAACTTAA

>OTU_385

GAATTGCGATAAGTAATGTGAATTGCAGAATTCAGTGAATCATCGAATCTTTGAACGCACCTTGCGCCTCTTGGTATTCC

GAGAGGCATGCCTGTTTGAGTGTCTTATAAAATCAATCCCCTCGGGTTtttCGGAACCCGGCGGTGGACTTGGAGTTGGG

CGTTCTGCCGGTCACACGGCTCGCCTTAAAGACGTTAGTGGGACAGGTTGCCTACGGCTTGACGTAATAAGTTTCGTCAG

ACCCGGTGACGCTGGCCCGCTCAGAACCCGCCCTTGGGCAACTACTTTATGCTAGACCTCAAATCAGGTAGGACTACCCG

CTGAACTTAA

>OTU_386

GAAATGCGATAAGTAGTGTGAATTGCAGAATTCAGTGAATCATCGAATCTTTGAACGCACATTGCGCCCTTTGGTATTCC

TTAGGGCATGCCTGTTCGAGCGTCATTTAACACCGTCAAGCTCCGCTTGGTGTTGGGCGTCTGTCCCGCCTCTGGGCTGT

GGACTCGCCTTGAAATCATTGGCGGCCGGTACGTTGGCTTCGAGCGCAGCAGAAACGCGAACTCGAGGCCGGCGGGCCAG

CTCCCAGAAGCTACCccccATATTTTGACCTCGGATCAGGTAGGGATACCCGCTGAACTTAA

>OTU_387

GAAATGCGATACCTGGTGTGAATTGCAGAATCCCGCGAACCATCGAGTCTTTGAACGCAAGTTGCGCCCGAGGCCACTCG

GCCGAGGGCACGCCTGCCTGGGCGTCACGCCAAAACACGCTCCCACACCCCTCATTGGGAATCGGGATGCGGCATCTGGT

CCCTCGTCTCGCAAGGGGCGGTGGACCGAAGTTCGGGCTGCCGGCGTACCGTGTCAAACACAGCGCGTGGTGGGCGTCTT

TGCTTTATCAACGCAGTGCATACGACGCGTAGCCGGCATGATGGCCTCAAAACGACCCAACAAACGTAGCGCACGTCGCT

TCGACCGCGACCCCAGGTCAGGCGGGACTACCCGCTGAGTTTAA

>OTU_388

GAATTGCGATAAGTAATGTGAATTGCAGAATTCAGTGAATCATCGAATCTTTGAACGCACCTTGCACTCTTTGGTATTCC

GAAGAGTATGTCTGTTTGAGTATCATGAGGAAGGCAACCcccTTGTTTTGTAATGAGACAAGAGTGGGCTTGGTTTATGG

CTTTTGTCGGCGTAATCGCCGGCTCAGCTGAAATACACGAGCAACCCATTTGAAACAAACGGCTTGACTCGGCGTAATAA

TTATTTCGCTGAGGACGTTTTCTTCAAAAGTTAGTGGTGCTTCTAGTGCGCTTTTGTAGCAACTCAATTTAGATCTCAAA

TCAGTCAGGACTACCCGCTGAACTTAA

>OTU_389

GAAATGCGATAAGTAGTGTGAATTGCAGAATTCAGTGAATCATCGAATCTTTGAACGCACATTGCGCCCTTTGGTATTCC

TTAGGGCATGCCTGTTCGAGCGTCATTTAACCCCTCAAGCTCAGCTTGGTGTTGGGCGTCCTGTCCcccACCcccGCCAC

GGgggCGGGGACTCGCCTCGAATCGATTGGCGGCCGGTACGTTGGCTTCGAGCGCAGCAGAAAAGCGAACTCGGCGCCTG

GCGAGCCGGCTCCCACAAGCTACacacCCCATTTTGACCTCGGATCAGGTAGGGATACCCGCTGAACTTAA

>OTU_390

GAAATGCGATAAGTAGTGTGAATTGCAGAATTCAGTGAATCATCGAATCTTTGAACGCACATTGCGCCCCTTGGTATTCC

ATGGGGCATGCCTGTTCGAGCGTCATTTGTACCCTCAAGCTCTGCTTGGTGTTGGGTGTTTGTCCCGCCTTTGGTGCGTG

GACTCGCCTCAAAATAATTGGCAGCCGGCATATTGGCCTTGGAGCGCAGCACATTTTGCGCTTCTAGTCAATGATTGTTG

GCGTCCAGCAAGTACAAATCTCAACTTTTGACCTCGGATCAGGTAGGGATACCCGCTGAACTTAA

>OTU_391

GAATTGCGATACGTAATGCGAATTGCAGATTTTCGTGAGTCATCGAATCTTTGAACGCACATTGCGCCcccTGGTATTCC

GGggggCATGCCTGTTTGAGCGTCGTTACCTTCTCAAAGCGGTAGTTTTGGTGGTGGgggAAGTCTGGCACTCCCCTAAA

ACACAGGTACACGACAGCGGTGCTGTTTGTAGTAGTGGGTGTTTGTTTAGCGCACGCTGCAATGTCTTAAGGCAACCCCT

TTCATTGCTGGCAAGCGAGACACGACATCTCTGCAAGCTCGACCTCAAATCAGGTAGGACTACCCGCTGAACTTAA

>OTU_392

GAAATGCGATAAGTAATGTGAATTGCAGAATTCAGTGAATCATCGAATCTTTGAACGCACATTGCGCCCGCCAGTATTCT

GGCGGGCATGCCTGTTCGAGCGTCATTTCAACCATCAAGCCCAGCGCTTGTGTTGGGGCCCCGCGGCTGCCCGCGGGCCC

TTAAAGACAGTGGCGGTTCTCGCTGCAGCACCGAGCGTAGTAGAATACACCTCGCCCTGGGAGCTGCgcgcgcgcCCCGC

CGTAAAACCcccccACCTCTCACAAGGTTGACCTCGGATCAGGTAGGAATACCCGCTGAACTTAA

>OTU_393

GAATTGCGATAAGTAATGTGAATTGCAGAATTCAGTGAATCATCGAATCTTTGAACGCAGCTTGCGCTctctGGTATTCC

GGAGAGCATGCCTGTTTCAGTGTCATGAAATTTCAACCACTAGGGTTTCCTAATGGATTGGATTTGGGCGTCTGCGATCT

CTGATCGCTCGCCTTAAAAGAGTTAGCAAGTTTGACATTAATGTCTGGTGTAATAAGTTTCACTGGGTCCATTGTGTTGA

AGCGTGCTTCTAATCGTCCGCAAGGACAATTACTTTGACTCTGGCCTGAAATCAGGTAGGACTACCCGCTGAACTTAA

>OTU_394

GAAATGCGATAAGTAATGTGAATTGCAGAATTCAGTGAATCATCGAATCTTTGAACGCACATTGCGCCCTCTGGTATTCC

GGAGGGCATGCCTGTTCGAGCGTCATTTGCTAAACTCAAGCCTACTTGTAGGCCTGGTCTTGGGTGCTGTCCTTTACGGA

GGACACGCCTCAAATGCAATGGCGACACTGCCTGGCTTCAAGCGTCTAGGAAACTTCATCGCTTCTGTCGGATAAATGTC

TGCCACTCAACCCCAATTtttATTCTGGGTTGACCTCGGATCAGGTAGGGATACCCGCTGAACTTAA

>OTU_395

GAAATGCGATAAGTAGTGTGAATTGCAGAATTCAGTGAATCATCGAATCTTTGAACGCACATTGCGCCCCTTGGTATTCC

ATGGGGCATGCCTGTTCGAGCGTCATTTGTACCCTCAAGCTTTGCTTGGTGTTGGGTGTTTGTCCTCTCCCTCGCGTTTG

GACTCGCCTTAAAACAATTGGCAGCCAGTATTTTGGTAATGAAGCGCAGCACATTTTGCGATTCTAGCTATAATACTTGC

GTCCATAAGCCTTttttAACTTTTGACCTCGGATCAGGTAGGGATACCCGCTGAACTTAA

>OTU_396

GAAATGCGATAAGTAGTGTGAATTGCAGAATTCAGTGAATCATCGAATCTTTGAACGCACATTGCGCCCATTGGTATTCC

AATGGGCATGCCTGTTCGAGCGTCATTTGTACCCTCAAGCTTTGCTTGGTGTTGGGCGTTTGTCTTTTGAGACTCGCCTT

AAATTCATTGGCAGCCGGCGTACTGGCTAGAGCGCAGCACAAATTGCAATCTAGCCATGAATGTCGGCGTCCACGAAGCC

CATTTTCACCTTTGACCTCGGATCAGGTAGGGATACCCGCTGAACTTAA

>OTU_397

GAATTGCGATAAGTAATGTGAATTGCAGAATTCAGTGAATCATCGAATCTTTGAACGCACCTTGCACTCTTTGGTATTCC

GAAGAGTATGTCTGTTTGAGTATCATGAAACTCTCAACCccccTATTTTGTAATGAAATGGGCGTGGGCTTGGATTATGG

TTGTCTGTCGGCGTAATTGCCGGCTCAACTGAAATACACGAGCAACCCTATTGAAATAGACGGTTTGACTTGGCGTAATA

ATTATTTCGCTAAGGACGTCTTCTTCAAATATAAGAGGTGCTTCTAATGCGCTTTATAGCACTTTAAGCTTTAGATCTCA

AATCAGTCAGGACTACCCGCTGAACTTAA

>OTU_398

GAAATGCGATACGTAATGTGAATTGCAGAATTCAGTGAATCATCGAATCTTTGAACGCACATTGCGCCCATTAGTATTCT

AGTGGGCATGCCTGTTCGAGCGTCATTTCAACCCTTAAGCCTAGCTTAGTGTTGGGAATCTACTGAGCAATCGGTAGTTC

CCCAAATCCAACGGCGGATCTGTGGTATCCTCTGAGCGTAGTAATTtttATCTCGCTTTTGTTAGGTGCTGCAGCTCCCA

GCCGCTAAACCccccAATTtttttAATGGTGACCTCGGATCAGGTAGGAATACCCGCTGAACTTAA

>OTU_399

GAAATGCGATAAGTAATGTGAATTGCAGAATTCAGTGAATCATCGAATCTTTGAACGCACCTTGCGCTCCTTGGTATTCC

GAGGAGCATGCCTGTTTGAGTGTCATTAAATTCTCAACCTGCCAGCTTTTATTAGCTTGGTTCAGGCTTGGATGTGGggg

TTGCGGGCTTCATATAACGAGGTCGGCTCTCCTTAAATGCATTAGCGGAACTTTTGTGGACCGTCTATTGGTGTGATAAT

TATCTACGCCGTGGATGTGAAGCAGCTTTATAAAGTTCAGCTTCTAACCGTCCATTGACTTGGACAAATTTTGACAATTT

GACCTCAAATCAGGTAGGACTACCCGCTGAACTTAA

>OTU_400

GAAATGCGATAAGTAATGCGAATTGCAGAATTCAGTGAGTCATCGAATCTTTGAACGCACATTGCGCCCCGTGGTATTCC

GCGGGGCATGCCTGTTCGAGCGTCATTTCAACCCTCAAGCTCTGCTTGGTATCGGGCCGTACCGGTAACGGTGGGCCTTA

GAATCAGTGGCGGTGCCGTCTGGCTCTAAGCGTAGTAATTTCTCCTCGCTATAGGTGCCCGGTGGTATCCGGCCAGCAAC

CcccTATTTTCTATGGTTGACCTCGGATCAGGTAGGGATACCCGCTGAACTTAA

>OTU_401

AAAATGCGATAAGTAATGTGAATTGCAGAATTCAGTGAATCATCGAATCTTTGAACGCACATTGCGCCCGGGAGTAATCT

CCCGGGCATGCCTGTCCGAGCGTCATTTCAGCCCTCGAGCCGCTATTCTTAGTGGATCGGTGTTGGGGCACTACGGTAAA

ACGTAGGCCCCGAAATGAAGTGGCGGTCCCGCCGCGGCGTCCTATGCGTAGTAACTTTCAGTCTCGCATCGGGTCCCGGC

GGTGACCTGCCGTCAACCTAATTATTTTCTATGGTTTGACCTCGGATCAGGTAGGGTTACCCGCTGAACTTAA

>OTU_402

AAAATGCGATACTTGGTGTGAATTGCAGAATCCCGTGAACCATCGAGTTtttGAACGCAAGTTGCGCCTGAAGCCATTCG

GCCGAGGGCACGTCTGCCTGGGCGTCACGCATCGCGTCGCCCACAACCACGCCTCCCTCATGGGGATGTGTTTTGTTTGG

GGCGGAGAATGGTCTCCCGTGCTCATGGTGCGGTTGGCCTAAaaaGGAGTCCCCTTCGACGGACGCACGGCTAGTGGTGG

TTTTCCAAGGCCTTCGTATCGAGCTGTGCATACGCGAGGGAATCGCTCTCCAAAGACCCCAACGTGTCGTCTTGCGACGA

CGCTTCGACCGCGACCCCAGGTCAGGCGGGACTACCCGCTGAGTTTAA

>OTU_403

GAAATGCGATACGCAATGCGAATTGCAGAACCGCGAGTCATCAGATCTTTGAACGCAAGTGGCGATAGCTATGGCTATCA

TGTTTGTTTCAGTGTATCCGGTTAATTACATCAAAGAACTTAATGTGATTGAAGCAATTCtctcACCAAGTACGAGGTTA

CTTCAGAGTAACGCCTAACCGAAAGCAGTCACTAATCGGTGATCATCGTTTAGAGCAATACCCTGTAAAACAGTAACCTA

AACAGTATTTATCCAATCATGGTACCTGAAATAAGCAAGATTACCCGCTGAACTTAA

>OTU_404

GAAATGCGATAAGTAATGTGAATTGCAGAATTCAGTGAATCATCGAATCTTTGAACGCACATTGCGCCCCTTGGTATTCC

GAGGGGCATGCCTGTTCGAGCGTCATTGCAACCCTCAAGCTCTGCTTGGTATTGGGCCcccGCCCCTCGCGGGGCGGGCC

CGGAACCCATCGGCGGCTGTGCCCAGCTCCGAGCGTAGTCAACacacCCCGCTCTGGATGACTTGGGCGCCGCCCGCCGG

ACGAACACCccccGAATCTTTCTCAAGGTTTGACCTCGGATCAGGTAGGGATACCCGCTGAACTTAA

>OTU_405

CTGCAAAACGACCGTCTGGCACAGCAGGCAGAGGCACTGGAAAACCAGGGCAAATGGGCGCAGGCGGCAGCACTTCAGCG

GCAACGACTGGCGCTGGACCCCGGCAGTGTATGGATTACTTACCGACTTTCGCAGGATCTCTGGCAGGCCGGACAACGCA

GCCAGGCCGATACGTTAATGCGCAATCTGGCGCAGCAGAAGCCGAATGACCCGGAGCAGGTTTACGCTTACGGGCTGTAT

CtctctGGTCATGACCAGGACAGAGCGGCGCTGGC

>OTU_406

GAAATGCGATACGTAATGTGAATTGCAGAATTCAGTGAATCATCGAATCTTTGAACGCACATTGCGCCCCTTGGTATTCC

ATGGGGCATGCCTGTTCGAGCGTCATTTGTACCCTCAAGCTTTGCTTGGTGATGGGTGTTTGTTCCGCGGCAACGCGTGA

ACTCGCCTCAAATGAATTGGCAGCCGGCAATCTGGTGTTGGAGCGCAGAACATTTTGCGCTCTTCGCTGTGTATTGCGGG

CGTCCATGAAGCGTACATTTTCGCGCTTGACCTCGGATCAGGTAGGGATACCCGCTGAACTTAA

>OTU_407

GAAATGCGATAAGTAGTGTGAATTGCAGAATTCAGTGAATCATCGAATCTTTGAACGCACATTGCGCCCCTTGGTATTCC

ATGGGGCATGCCTGTTCGAGCGTCATTTGTACCTTCAAGCTCTGCTTGGTGTTGGGTGTTTGTCTCCTGTAGACTCGCCT

CAAAACAATTGGCAGCCGGCGTATTGATTTCGGAGCGCAGTACATCTCGCGCTTTGCACTCATAACGACGACATCCAAAA

GTACATTtttACACTCTTGACCTCGGATCAGGTAGGGATACCCGCTGAACT

>OTU_408

GAAATGCGATAACTAATGTGAATTGCAGAATTCAGTGAATCATCGAGTCTTTGAACGCACATTGCGCCCTCTGGTATTCC

GGAGGGCATGCCTGTCCGAGCGTCATTGCTGCCCTCAAGCACGGCTTGtgtgtTGGGCCcccGTCCccccTCTGCCGGgg

ggACGGGCCCGAAAGGCAGCGGCGGCACCGCGTCCGGTCCTCGAGCGTATGGGGCTTCGTCACCCGCTCTTGTAGGCCCG

GCCGGCGCCAGCCGACCCCAACCCTAAATTttttCAGGTTGACCTCGGATCAGGTAGGGATACCCGCTGAACTTAA

>OTU_409

GAAATGCGATAAGTAATGTGAATTGCAGAATTCAGTGAATCATCGAATCTTTGAACGCACCTTGCGCTCCTTGGTATTCC

GAGGAGCATGCCTGTTTGAGTGTCATTAATatatatatCAACCTTCTCTTtttGAGTGGTTTGGATGTGGgggTTTGCTG

GCCTTGTAAAGGGTTCAGCTCCCCTGAAATGCATTAGCAGAACAACCCTGTTCATTGGTGTGATAACTATCTACGCTATT

GAATGTAAGGgggCAGTTCAGCTTTCTAACAGTCCTCGGACAATTCATCATTAATGTGACCTCAAATCAGGTAGGACTAC

CCGCTGAACTTAA

>OTU_410

GAAATGCGATAAGTAATGTGAATTGCAGAATTCAGTGAATCATCGAATCTTTGAACGCACATTGCGCCCATTAGTATTCT

AGTGGGCATGCCTGTTCGAGCGTCATTTCAACCCTTAAGCCTAGCTTAGTATTGGGAATTGGCTTTGCTGCCACTCCTCA

AATTCAACGGCGGATTTATAGCAATCTCTGAACGTAGTAATATTttttCTCGTTtttGAAATACTGTAAATCTCAGCCGC

TAAACCcccAAATTTCTTATGGTTGACCTCGGATCAGGTAGGAATACCCGCTGAACTTAA

>OTU_411

GAAATGCGATAAGTAATGTGAATTGCAGAATTCAGTGAATCATCGAGTCTTTGAACGCACATTGCGCCcccTGGTATTCC

GGggggCATGCCTGTCCGAGCGTCATTGCTGCCCTCAAGCCCGGCTTGGTCTTGGGTGCCGCCcccccGGgggCgcgcCC

GAAAGGCAGCGGCGGCACCGCGTCCGGTCCTCGAGCGTATGGGGCTTTGTCACCCGCTTCGTAGGCCCGGCCGGCGCCAG

CCGACCccccAATCTTTCTTTAGGTTGACCTCGGATCAGGTAGGGATACCCGCTGAACTTAA

>OTU_412

GAAATGCGATAAGTAGTGTGAATTGCAGAATTCAGTGAATCATCGAATCTTTGAACGCACATTGCGCCCCTTGGTATTCC

ATGGGGCATGCCTGTTCGAGCGTCATTTGTACCCTCAAGCTTTGCTTGGTGTTGGGTGTTTGTCTTCCCTGGGAGACTCG

CCTTAAAACAATTGGCAGCCGGCATATTGGTATTGGAGCGCAGCACAAAACGCGCTTCCCCATAATTGTCGGCGTCCATC

AAGACCATTtttttCACTTGACCTCGGATCAGGTAGGGATACCCGCTGAACTTAA

>OTU_413

GAAATGCGATAAGTAATGTGAATTGCAGAATTCAGTGAATCATCGAATCTTTGAACGCACATTGCGCCCGCCAGTATTCT

GGCGGGCATGCCTGTTCGAGCGTCATTTCAACCCCTCAAGCCCTGCTTGGTGCTGGGGCCCTGCGGCTGCCCGCAGGCCC

CGAAAACCAGTGGCGGGCTCGCTAGTCACACCGAGCGCAGTAGCACATCTCGCTCAGGGCGTGCGGCGGGTCCCTGCCGT

GAAAACACCCCAACTTCCAAAGGTTGACCTCGGATCAGGTAGGAATACCCGCTGAACTTAA

>OTU_414

GAAATGCGAAAAGTAGTGTGAATTGCAGAATTCAGTGAATCATCGAATCTTTGAACGCACATTGCGCCCCTTGGTATTCC

ATGGGGCATGCCTGTTCGAGCGTCATTTGTACCTTCAAGCTCTGCTTGGTGTTGGGTGTTTGTCTCGCCGCGTGCGTGGA

CTCGCCTTAAAGCAATTGGCAGCCGGCATACTGGTCGAAAAGCGCAGCACAATTTGCGCTACTCGTCCGGTAGGTTGGCG

TCCATCAAGCCTATTtttCACGCTTGACCTCGGATCAGGTAGGGATACCCGCTGAACTTAA

>OTU_415

GAAATGCGATAAGTAGTGTGAATTGCAGAATTCAGTGAATCATCGAATCTTTGAACGCACATTGCGCCCCTTGGTATTCC

ATGGGGCATGCCTGTTCGAGCGTCATTTGTACCCTCAAGCTTTGCTTGGTGTTGGGTGTTTGTCCTCTCCTCGGTTTGGA

CTCGCCTTAAAGTAATTGGCAGCCGGCGTATTGGCCTTGGAGCGCAGCACATCTTGCGCTTCATGTCAACATACACTGGC

GTCCATTAAGCCTACTTTTCACTTTTGACCTCGGATCAGGTAGGGATACCCGCTGAACTTAA

>OTU_416

GAAATGCGATACTTGGTGTGAATTGCAGAATCCCGTGAACCATCGAGTCTTTGAACGCAAGTTGCGCCCAAAGCCATTAG

GCCGAGGGCACGCCTGCCTGGGTGTCAcacaTCGTCATCCCCATGCAAAAGCGCATCGGGGCGAAAGGTGGCCTCCCGCG

ACacacCCGTCGTGGTTGGTCGAAAACCAAGGTAACGTCGGGGTCCcccccGCCAGAAACGGTGGATGAGCAACGCTCGA

GACCAACGGCGGCGGCGGACACTCGGCAATCCAATCGATCGACCCTACAGCGTTCCTTGCATAATAAACAGGCAAAGACG

CTCTTAACGAGACCTCAGGTCAGGCGGGGCTACCCGCTGAGTTTAA

>OTU_417

GAAATGCGATAAGTAATGTGAATTGCAGAATTCAGTGAATCATCGAATCTTTGAACGCACATTGCGCCCGCCAGTATTCT

GGCGGGCATGCCTGTCCGAGCGTCATTTCAACCCTCGGGCCcccccccTTTTCCCCTCGCGGgggAGGgggCGGGCCCGG

CGTTGGGCCCAGGCGTCCTCCAAGGGCGCCTGTCCCCGAAACCCAGTGGCGGCCTCGCCGCTGCCTCCTCCGCGTAGTAG

CACAAACCTCGCGGGCGGAAGGCGGCGCGGCCACGCCGTAAAACCCCAAACTTTTACCAAGGTTGACCTCGGATCAGGTA

GGAATACCCGCTGAACTTAA

>OTU_418

GAAATGCGATAAGTAATGTGAATTGCAGAATTCAGTGAATCATCGAATCTTTGAACGCACCTTGCGCTCCTTGGTATTCC

GAGGAGCATGCCTGTTTGAGTGTCATTAATatatatCAACCACTTCTTGTTGAGTGGCTTGGATGTGGggggTTTGCTGG

CCTCTTGAAGGGTCAGCTCCCCTGAAATGCATTAGCAGAACCAACCTGTTCATTGGTGTGATAACTATCTACGCTATTGA

AAGGTAAGGCAGTTCAGCTTCCTAACCGTCCTTGGACAATTTATCATTTATGTGACCTCAAATCAGGTAGGACTACCCGC

TGAACTTAA

>OTU_419

GAAATGCGATACTTGGTGTGAATTGCAGAATCCCGTGAACCATCGAGTTtttGAACGCAAGTTGCGCCCGATGCCATTAG

GTTGAGGGCACGTCTGCCTGGGTGTCACATATCGAATCCCCTTGCCAATTTCCTATTTATTAGGTATTGCGTGCAGGGTG

AATATGTTGGCCTCCCGTGAGCTCTGTCTCGTGGTTGGTTGAAAATTGAGACCTTGGTAGGGTGTGCCATGATAGATGGT

GGATGtgtgACCCACGAGACCAAATCATGTGCAGCTCTATTGAACGTGGACTCTTTTACCCACATGTGTTTTATAACGCT

CGTGATGAGACCTCAGGTCAGGCGGGGCTACCCGCTGAATTTA

>OTU_420

CAAATGCGATAGATAGTGCGAACTGCAGATATTCTGAGCACTAAAATGTCGAATGCACATTGCGCCTTGGGATTTACATC

CCTCGGCATGCCTGGTTCAGGGTCGTTTCTCAAaaaGGCAGCAGACTGCGCTGCTTGAACGACCAGGCACGAGCACGCCC

AGCCGTGGGGCCGCTCCGGGCCTGGTCTTGTTGTGGTAGCTGTTGCGCAAGGACAAGGGTCCAAGGGCAACTGCCGGTCG

TGCATAAACacacaAAGATTGGCAGAGACTCGCCTAGGACTTCGTTCAAACGAGGCGCTCGATATCTCGACCTGAACTCA

GACATGATGACCCGCTGAACTTAA

>OTU_421

GAAATGCGATACGTAATGTGAATTGCAGAATTTTGTGAATCATCGAATCTTTGAACGCACATTGCACCTGGTGGTATTCC

GCCAGGTATGCTTGTTTGAGCGTCATTTCTTTCTCACTTTCAGTGGTTTTGCTGCTATCTTCCCCTTCTGGGGAAGTGCA

GTGAAAAGAAATGGCAGTTCTTTTATTTTGCTAAACGTGTTTTAGGTCTAACCAACTACATTACTAGGTATTATTTAAGA

AGGCTTGATAACTTTTCTCAAGTTTGACCTCAAATCAAGTAAGACTACCCGCTGAACTTAA

>OTU_422

GAAATGCGATAAGTAATGTGAATTGCAGAATTCAGTGAATCATCGAATCTTTGAACGCACATTGCGCCcccTGGTATTCC

GGgggCATGCCTGTTCGAGCGTCATTTCACCACTCAAGCCTCGCTTGGTATTGGGCATCGCGGTCCGCCGCGTGCCTCAA

ATCGACCGGCTGGGTCTTCTGTCCCCTAAGCGTTGTGGAAACTATTCGCTAAAGGGTGTTCGGGAGCTACGCCGTAAACA

ACCCATTCTAGTTGACCTCGGATCAGGTAGGGATACCCGCTGAACTTAA

>OTU_423

GAAATGCGATAAGTAATGTGAATTGCAGAATTCAGTGAATCATCGAATCTTTGAACGCACATTGCGCCCCGTGGTATTCC

GCGGGGCATGCCTGTTCGAGCGTCATTTCACCACTCGAGTCTGACTCGGTATTGGGCGTCGCGCATCGCCGcgcgcCTTA

AAGTCTCCGGCTGGGCGGCCCGTCTCCGAGCGTTGTGACATCACAGTTTCGCTAGGGAGTCGCGGGCGGCGTTGGCCGTT

AAATACCCCATCAAAGGTTGACCTCGGATCAGGTAGGGATACCCGCTGAACTTAA

>OTU_424

GAAATGCGATAAGTAATGTGAATTGCAGAATTCAGTGAATCATCGAATCTTTGAACGCACCTTGCGCCCTTTGGTATTCC

GAAGGGCATGCCTGTTTGAGTGTCATTAAATTCTCAACCTTGGAAGGCTTTACAAGTTtttCAAGGCTTGGATCTGGAGG

CTGCGGGCTTCTTCTGAAGTCGGCTCCTCTTAAATGCATTAGCTGGGTTTGCGCCTTCAGCCTATTGGTGTGATAATTAT

CTACGCCTCCTGGTCGTTAGCGTGTTATAGCTCTCCAGCTTCTAACCGTCTCCTGGAGACAATCTTTGACCATTTGACCT

CAAATCAGGTAGGACTACCCGCTGAACTTAA

>OTU_425

GAAATGCGATAAGTAATGTGAATTGCAGAATTCAGTGAATCATCGAATCTTTGAACGCACATTGCGCTCTTTGGTATTCC

GGAGAGCATGCCTGTTTGAGAATCAGTAAATTCATCAACTGCTTAATTGCAGTTGGATATGGGCCTTTTGAGACCTCACG

GTCACAATGGTCTGAAATTCAATGTTACGCTTCCTTGAACCCAACGGTTCTTGAGCATACGCATGAATGAGGTAACTCAC

AAGTACGTTTGTTCTCATGAATGGTGGGTTGGACCGGAGTGGGTAACTCAACTATTACTCAGCGGAGGTGACGTCAGTCA

TCAGCCTTCTCTGAACACTTGGTCTCAAATCAGGTAGGACTACCCGCTGAACTTAA

>OTU_426

GAAATGCGATAAGTAATGTGAATTGCAGAATTCAGTGAATCATCGAATCTTTGAACGCACATTGCGCCCCTTGGTATTCC

GAGGGGCATGCCTGTTCGAGCGTCATTACACCACTCAAGCTATGCTTGGTATTGGGCGTCGTCCTTAGTTGGGCgcgcCT

TAAAGACCTCTGGCGAGGCCACTCCGGTCTTTAGGCGTTAGTAGAATTTATTCGAACGTCTGTCAAAGGAGAGGAACTTC

TTGCCCGACTGAAACCTTTATTtttCTTAGGTTGACCTCGGATCAGGTAGGGATACCCGCTGAACTTAA

>OTU_427

GAAATGCGATAAGTAATGTGAATTGCAGAATTCAGTGAATCATCGAATCTTTGAACGCACCTTGCGCTCCTTGGTATTCC

GAGGAGCATGCCTGTTTGAGTGTCATGAAACTCTCCAATTCTATAGTTttttCTATAGAATCTGGGTCCTTGGGTTTGCA

TGGGTACCCCATGCTTACCTTAAATGTATTAGCTGAATACCAGCAGGGTTGTTAAGTTtttttCACCCTCGGAAGAAGGT

ATTTGCTTCCAATCTGCGCTGTCTTTTGTAGACAGGCATTTTGTATCTTCGGCCTCAAATCAGGTAGGATTACCCGCTGA

ACTTAA

>OTU_428

GAAATGCGATAAGTAATGTGAATTGCAGAATTCAGTGAATCATCGAATCTTTGAACGCACATTGCGCCCTTTGGCATTCC

GAAGGGCATGCCTGTTCGAGCGTCATTACACCAATCAAGCCTGGCTTGGTATTGGGCGACGGGGACGTCACACGCCcccG

CGCCCCAAAGACCTCTCCGGCGGGACGGACCGAATCTCAGCGTTGTGCAAATGTCGCTGGCGAGACGGGACGGACGTGCC

GTTAAACACCCCTTCTCAAGGTTGACCTCGGATCAGGTAGGGATACCCGCTGAACTTAA

>OTU_429

GAAATGCGATAAGTAATGTGAATTGCAGAATTCCGTGAATCATCGAATCTTTGAACGCACATTGCGCCCCTCGGTATTCC

GGgggCATGCCTGTTCGAGCGTCATTACACCAATCAAGCCCCGGCTTGGTCTTGGGCGTCGCGGTCCGCCGCGTGCCTCA

ATGTCGCCGGCTGGTGCGTCCGTCTCCGAGCGTCGTGACTTCATTGTTCGCTTCTGGAGGCCGGACGGGCCACGCCGTCA

ACCccccATATCTAAAGGTTGACCTCGGATCAGGTAGGGATACCCGCTGAACTTAA

>OTU_430

GAAATGCGATAAGTAGTGTGAATTGCAGAATTCAGTGAATCATCGAATCTTTGAACGCACATTGCGCCCATTGGTATTCC

AATGGGCATGCCTGTTCGAGCGTCATTTGTACCCTCAAGCCTTGCTTGGTGTTGGGTGTTGTCCCCTGGGACTCGCCTCA

AAATAATTGGCAGCCGGCATATTGGTTCGGAGCGCAGCACAAATTGCGGTCTTTCCATGAATGTCGGCGTCCATGAAGCC

CACTTTCCACTTTTGACCTCGGATCAGGTAGGGATACCCGCTGAACTTAA

>OTU_431

GAAATGCGATACGTAATGCGAATTGCACAACCGCGAGTCATCAAATCTTTGAACGCAAATGGCGATAGCGCAAGCTATCA

TGTTTGTTTCAGTGTATTTGGTTAATTACATCAAAGATCTAGATGCGATTGAAGTGATTCtctcGCTAAGTATGAGGCAC

CCCGATGGGTATAATGCCAAACGAAGTAGTCACGAATTAGTGATGTCGTCTTGGATCTTTATACACATCATTAAAGGTTG

CCTATCAAGATCATATCCTAAATatatTACCTGAAATAAGCAAGATTACCCGCTGAACTTAA

>OTU_432

GAAATGCGATAAGTAATGTGAATTGCAGAATTCAGTGAATCATCGAATCTTTGAACGCACCTTGCGCTCCTTGGTATTCC

GAGGAGCATGCCTGTTTGAGTGTCATGAAATCTTCAACCTATAAACCTTTGCGGGTTTGTAGGCTTGGACTTGGAGGCTT

TGTCGGCCTAATGGTCGGCTCCTCTTAAATGTATTAGCTTGATTCCTTGCGGATCGGCTGTCGGTGTGATAATTTTCTAC

GCCGCGACCGTGAAGCGTTTGGCGAGCTTCTAATCGTCTCGCATGAGACAATCTATTGACCTCTGACCTCAAATCAGGTA

GGACTACCCGCTGAACTTAA

>OTU_433

GAAATGCGATAAGTAGTGTGAATTGCAGAATTTAGTGAATTATTGAATCTTTAAACGTATATTGCGCCTTTTGGTATTCC

TTAGAGCATGCCTGTTTGAGCGTTATCTAAACCTTTAAGCACTGCTTGGTGTTAGGTGCCTGTTCTGCCcccGcgcgTGG

ACTCACCTTAAATCTATTGGCGGCTCTTACGTTAGCTACGAGCGCAGCAGAAACGCGAACTTGTGGACCTGGCGGAGTAG

CTTCTAGAAGCTACATTCACCATTTTGACCTCGGATCAGGTAGGGATACCCGCTGAACTTAA

>OTU_434

GAAATGCGATAAGTAATGTGAATTGCAGAATTCAGTGAATCATCGAATCTTTGAACGCACCTTGCGCCCTTTGGTATTCC

GAAGGGCATGCCTGTTTGAGTGTCATGAAACCTCACCCCACTTGGGTTtttGCCCGAGCGGTGGTGGATTGGGTGTTTGC

CGTCATTGGCTCACCTTAAAAGCATAAGCAACTTTGGATGTAATACGTTTCATCCTTTCGAGTGGCTGATAACCAACCAT

AACTCATGATCTGGCCTCAAATCAGGTAGGGCTACCCGCTGAACTTAA

>OTU_435

GAAATGCGATAAGTAGTGTGAATTGCAGAATTCAGTGAATCATCGAATCTTTGAACGCACATTGCGCCCCTTGGTATTCC

ATGGGGCATGCCTGTTCGAGCGTCATTTGTACTTTCAAGCTATGCTTGGTGTTGGGTGTTTGTCCAGCTTTCAGTGTTTG

GACTCGCCTTAAAGTGATTGGCAGCCAGTGTTTTGGTATAAAGCGCAGCACATTTTGCGATTTAATCCAGTAATATTAGC

AACCAACAAGCTCAATTTATCACTTTTGACCTCGGATCAGGTAGGGATACCCGCTGAACTTAA

>OTU_436

GAAATGCGATAAGTAGTGTGAATTGCAGAATTCAGTGAATCATCGAATCTTTGAACGCACATTGCGCCCCTTGGTATTCC

ATGGGGCATGCCTGTTCGAGCGTCATTTGTACCCTCAAGCTTTGCTTGGTGTTGGGCGTCTTGTCGGACTATCCGACTCG

CCTCAAATACATTGGCAGCCGGCACTTTGGCCTAGGAGCGCAGCACATTTTGCGATCGTAAGCCGTTGTGACAGCATCCA

TAAGATTTATTTTACCACGTTTGACCTCGGATCAGGTAGGGATACCCGCTGAACTTAA

>OTU_437

GAAATGCGATAAGTAATGTGAATTGCAGAATTCAGTGAATCATCGAATCTTTGAACGCACATTGCGCCCTCTGGTATTCC

GGggggCATGCCTGTTCGAGCGTCATTTACACCCCTCAAGCCTAGCTTGGTATTGGGAGCTGCCccccGGGGCACCcccG

AAATGTAGCGGCGGTGCGGCCGGATTTCCTAGCGCAGTGACTTTTCAATCTCGCTACAAGTAATTCGTGTCGTCCTTGCC

GGACAACAACCAAATTtttttACGATTGACCTCGGATCAGGTAGGGATACCCGCTGAACTTAA

>OTU_438

GAAATGCGATAAGTAATGTGAATTGCAGAATTCAGTGAATCATCGAATCTTTGAACGCACATTGCGCCCATTAGTATTCT

AGTGGGCATGCCTGTTCGAGCGTCATTTCAACCCCTAAGCACAGCTTACTGTTGGGACTCTACGGCCTCCGTAGTTCCCC

AAAGAAATTGGCGGAGTGGCAGTAGTCCTCTGAGCGTAGTAATTtttttttATCTCGCTTTTGTTAGGTGCTGCCccccc

GGCCGTAAAACCcccAAATTtttCTGGTTGACCTCGGATCAGGTAGGAATACCCGCTGAACTTAA

>OTU_439

GAAATGCGATACGTAATGTGAATTGCAGAATTGTGAATCATCGAATCTTTGAACGCACATTGCGCCTTTTGGTATTCCAA

AAGGCATACCTGTTTGAGAGTCATTTCATTCTCAAaaaCCTAGTTtttGGTATTGGTTCTTATCTTTCGCAAGAAAGTGA

ACTGAAATGAAATGGCAAAGCCTCGATTttttGTCAAAAGTGTCTTAGGTTTTACCAACTACACTTGCGTAACGAAATTT

CGAATTCAAGCTTGGCCTTACAACAGCCTTtttAAGTTTGACCTCAAATCAGGCAAGACTACCCGCTGAACTTAA

>OTU_440

GAAATGCGATAAGTAATGTGAATTGCAGAATTCAGTGAATCATCGAATCTTTGAACGCACATTGCGCCCTTTGGTATTCC

GAGGGGCATGCCTGTTCGAGCGTCATATAAGCCATCCAGCGCGGCTGGGTCTTGGGCACCGCTTCCGAGCGGGCCTCAAA

GGCAGTAGCGGGCCGCTAGGTCCCCGAGCGCAGTACGTATTCTCGCTATGGGAACGCTGGTGAGAGCACGCTTATTCAAA

CCCCTTTTATTCAGGTTGACCTCGGATCAGGTAGGGATACCCGCTGAACTTAA

>OTU_441

GAAATGCGATAAGTAGTGTGAATTGCAGAATTCAGTGAATCATCGAATCTTTGAACGCACATTGCGCCCTTCGGTATTCC

GTCGGGCATGCCTGTTCGAGCGTCGTTGAAATAACCTTCAAGCTCCGCTTGGCCTTGGGCGCCTGTTCGCGCCTCCGTGC

GTGCGACTCGCCTCAAATCCATTGGCAGCCGGCGGGTCGGCTTCGcgcgcAGCACATACGcgcgGCGATGCCAGCCCGTC

GCCTCCCCAACAAAGACGCATTGTCAGTCTGACCTCGGATCAGGTAGGGATACCCGCTGAACTTAA

>OTU_442

GAAATGCGATAAGTAGTGTGAATTGCAGAATTCAGTGAATCATCGAATCTTTGAACGCACATTGCGCCCTTTGGCATTCC

TTAGGGCATGCCTGTTCGAGCGTCATTTAACCCCTCAAGCTCAGCTTGGTGTTGGGCGTCCTGTCCCTCTGCCccccTCG

CGGCGGCGGGGACTCGCCTCAAATCGATTGGCGGCCGGTACGTTGGCTTCGAGCGCAGCAGAAACGCGAACTCGGTGCCC

AGCGAGCCGGCTCCCACAAGCCAcacaTCCTAAGTTTGACCTCGGATCAGGTAGGGATACCCGCTGAACTTAA

>OTU_443

GAAATGCGATACGTAGTGTGAATTGCAGAATTCAGTGAATCATCGAATCTTTGAACGCACATTGCGCCCTTTGGTATTCC

AAAGGGCATGCCTGTTCGAGCGTCATTTGTACCCTCAAGCTTTGCTTGGTGTTGGGCGTTttttGTCTTTGGTTGCCAAA

GACTCGCCTTAAAACGATTGGCAGCCGGCCTCCTGGTTTCGCAGCGCAGCACATTtttGCGCTTGCAATCAGCAACAGAG

GGCGGCACTCCATCAAGACTCCATTTTCACGTTTGACCTCGGATCAGGTAGGGATACCCGCTGAACTTAA

>OTU_444

GAAATGCGATACTTGGTGTGAATTGCAGAATCCCGTGAACCATCGAGTCTTTGAACGCAAGTTGCGCCCGAAGCCACTAG

GCCGAGGGCACGTCTGCCTGGGCGTCACACGCCGTTGCCcccATGTGCACTGCCAAAAGCGTGTTCAGGAGGGGCGGAGA

CTGGCTTCCCATGAGCATTGCCTTGTGGTTGGCCTAAATTCGAGTCGTCGGCCGCAATCGCCTCGACATTCGGTGGTTTT

CGATTATATCGGTGCCCCGTCGTGCGCGAATCCGTGACCGACTAGACCCGTACGACCCCAATGTGCTGCGAACGCAGTGC

CTTCAACGCGACCCCAGGTCAGGCGGGATCACCCGCTGAATTTAA

>OTU_445

GAAATGCGATAAGTAATGTGAATTGCAGAATTCAGTGAATCATCGAATCTTTGAACGCACATTGCGCCcccTGGTATTCC

GGggggCATGCCTGTTCGAGCGTCATTTCACCACTCAAGCCTCGCTTGGTATTGGGCGTCGCGAGTCCCTCGcgcgcCTC

AAAGTCTCCGGCTGAGCGGTTCGTCTCCCAGCGTTGTGGCAACTATTTCGCAGTGGAGTTCGAGTCGTCGCGGCCGTTAA

ATCTTTCAAAGGTTGACCTCGGATCAGGTAGGGATACCCGCTGAACTTAA

>OTU_446

GAAATGCGATAAGTAATGTGAATTGCAGAATTCAGTGAATCATCGAATCTTTGAACGCACCTTGCGCCTTTTGGCTATTC

CGAAGGGCATGCCTGTTTGAGTATCATGAACACCTCAACCCTCATGGTTTGCCGTGACGGGCTTGGACTTTGGAGGTCTT

GCAGGCTTATGGCCGGCTCCTCTCAAATGAATTAGCTTACCGGTGTACGGTGGCATCACGGGTGTGATAATTATCTGCGC

TTGTGGTTGTCTGCCAGGTAACCTTCAACCTTGGAGGTTTGCTGGGGCTTACAAATGTCtctcCTCAGTGAAGACAGCTT

TCTGAACGTTTGATCTCAAATCAGGTAGGACTACCCGCTGAACTTAA

>OTU_447

GAAATGCGATAAGTAATGTGAATTGCAGAATTCAGTGAATCATCGAATCTTTGAACGCACCTTGCGCTCCTTGGTATTCC

GAGGAGCATGCCTGTTTGAGTGTCATTAATatatCAACCTCCTCAAGTTTTGACTTGTCGAGTGTTTGGATGTGGggggT

CTTTTGCTGGTCTCTTTTGAGGTCGGCTCCCCTAAAATGCATTAGCGGAACAATTTGTTGACCCGTTTATTGGTGTGATA

ACTATCTACGCTTTTGACGTGAAACAGGTTCAGCTTCTAACGGTCCATTGACTTGGACAATTtttCATTAATGTGACCTC

AAATCAGGTAGGACTACCCGCTGAACTTAA

>OTU_448

GAAATGCGATAAGTAATGTGAATTGCAGAATTCAGTGAATCATCGAATCTTTGAACGCACATTGCGCCCTCTGGTATTCC

GGAGGGCATGCCTGTTCGAGCGTCATTACAACCCTCAAGCCTGGCTTGGTGTTGGGCCTCCGTCCACTGGACGGGCCTGA

AAATCAGTGGCGATGCCCTTCCAACTTCAAGCGTAGTAGATCTATCTTCGTCTTGAGTGGTTGGATGGTGTGGTTTGCCA

AAACCAAACCTCTGGTTTTATCATCTGGTTGACCTCGGATCAGGTAGGAATACCCGCTGAACTTAA

>OTU_449

GAAATGCGATAAGTAGTGTGAATTGCAGAATTCAGTGAATCATCGAATCTTTGAACGCACATTGCGCCCTCTGGTATTCC

GGggggCATGCCTGTTCGAGCGTCATTACAACCCTCAAGCTTTGCTTGGTATTGGACATTGCCAGTTTCTGGCAGGTCTT

AAAATCAGTGGCGGTGCCATTTGGCTTCAAGCGTAGTAATTCTTCTCGCTTTGGAGATCCAGGTGGTTACTTGCCAATAA

CCcccAATTttttCAGGTTGACCTCGGATCAGGTAGGGATACCCGCTGAACTTAA

>OTU_450

GAAATGCGATAAGTAGTGTGAATTGCAGAATTCAGTGAATCATCGAATCTTTGAACGCACATTGCGCCCCTTGGTATTCC

ATGGGGCATGCCTGTTCGAGCGTCATTTGTACCCTCAAGCTTTGCTTGGTGTTGGGCGTCTTGTCGTATTACGACTCGCC

TTAAATACATTGGCAGCCGGCACTTTGGCCTAGGAGCGCAGCACATTTTGCGATCGTAGCCCGTTGTACTGGCGTCCATC

AAGAACATTTACCACGTTTGACCTCGGATCAGGTAGGGATACCCGCTGAACTTAA

>OTU_451

GAAATGCGATAAGTAGTGTGAATTGCAGAATTCAGTGAATCATCGAATCTTTGAACGCACATTGCGCCCTTTGGTATTCC

TTAGGGCATGCCTGTTCGAGCGTCATTTCAACCCTCAAGCCTAGCTTGGTGTTGGGCGTCTGTCCCGCCTCCGcgcgcCT

GGACTCGCCTCAAAAGCATTGGCGGCCGGTTCCCAGCAGGCCACGAGCGCAGCAGAGCAAGCGCTGAAGTGGCTGCGGGT

CGGCGCACCATGAGCCcccccACACCAGAATTTTGACCTCGGATCAGGTAGGGATACCCGCTGAACTTAA

>OTU_452

AAAATGCGATAAGTAATGTGAATTGCAGAATTCAGTGAATCATCGAATCTTTGAACGCACATTGCGCCCGCCAGTATTCT

GGCGGGCATGCCTGTTCGAGCGTCATTTCAACCATCAAGCCCCAGGCTTGTGTTGGAGCCCTGCGGCTGCCGCAGCCTCC

CAAAACCAGTGGCGGGCTCGCTATCACACCGAGTGCAGTAGTTTACTCTTCGCTCAGGGCGTGTGGCGGGTGCTAGCCGT

GAAACCcccAACTTCTCAAGGTTGACCTCGGATCAGGTAGGAATACCCGCTGAACTTAA

>OTU_453

GAAATGCGATAAGTAGTGTGAATTGCAGAATTCAGTGAATCATCGAATCTTTGAACGCACATTGCGCCCCTCGGTATTCC

GTGGGGCATGCCTGTTCGAGCGTCATTTACACCCTCAAGCTCTGCTTGGTGTTGGGCGTCTGTCCCGCTCCCGTAGCGCG

GACTCGCCTCAAAGTCATTGGCGGCGGTCGTGCCGGCTCCTCGCGCAGCACATTTGCGCTTCTCGGAGGCCCGGCGGATC

AGCGTCCAGCAAGACACCTTCATGACTTGACCTCGGATCAGGTAGGGATACCCGCTGAACTTAA

>OTU_454

GAAATGCGATACTTGGTGTGAATTGCAGGATCCCGCGAACCACCGAGTCTTTGAACGCAAGTTGCGCCCGGAGCCTTCTG

GCCGAGGGCACGTCTGCCTGGGCGTCACGCATCGCTGCCcccACATACAACACCCACTTAGGTCTGTTGCATTGGCGGgg

gCACATGTTGGCCTCCCGTGCGCAATCGTCGCACGGATGGCTTAAAATTGAGTCCTCGGCATCTGTTGTCGTGACACTAC

GGTGGTTGATTCAACCTCGGTACCGTGTCTCGATCTCAGCTTGCGTGCCTCCTCTTTGCGAGTGAGGGAGGAATCTTATG

TTGACCCTTTGAACATTGTCCCCAAAGACGATGCTCTCGACGCGACCCCAGGTCAGGCGGGACTACCCGCTGAGTTTAA

>OTU_455

GAAATGCGATAAGTAATGTGAATTGCAGAATTCAGTGAATCATCGAATCTTTGAACGCACATTGCGCCCCTTGGTATTCC

GGggggCATGCCTGTTCGAGCGTCATTTCAACCCTCAAGCTCTGCTTGGTATTGGGCTCCGTCCTCCTCGGACGCGCCTC

AAAGACCTCGGCGGTGGCGTCTTGCCTCAAGCGTAGTAGAAACACCTCGCTTTGGAGTGCATGGCGTCGCCCGCCGGACG

AAACCTTTTGTATTTTCTCAAAGGTTGACCTCGGATCAGGTAGGGATACCCGCTGAACTTAA

>OTU_456

CTTGACTGACGCGTTCGACGCGGCGGAACAAATGCTCCTGTCGCGTTTTGCCGAGGTGAAGCTTTCCGATATCGcgcgTG

ATTTTGATCTTCGATTCCGAAATTTAAACGAGGAGCCTGCCCAGGTGATTTGATTGGTCAGCGCGGTTTCAGGGAACGCA

ATAAAGCCTTTCCCAGTCTCGCATTGAGATAACCCTGAGGCCGAAGGCGACTGCTTTTCCTGTCTGATGGAGGCAATGGT

TTGCCCAATATCGGCTTGGCCTGATGCAGACTGCATGAGGAAGCTGGACTTGTGGCGATAAGCCATTTGTGCCAGAAGGC

TATGAGTTCAGCGTCACCTTGGAAAGTTATAGCCCGTTATGGAATTG

>OTU_457

GAAATGCGATAAGTAATGTGAATTGCAGAATTCAGTGAATCATCGAATCTTTGAACGCACATTGCGCCCTGTGGTATTCC

GCAGGGCATGCCTGTTCGAGCGTCAGTACAACACTCAAGCTCAGCTTGGTCTTGAGCCTAGCCGGTCACGGTGGGCTTTA

AAATTAGTGGCGGTGCCGCCTGGCTCTAAGCGTAGTAATACTCCTCGCTATTGAGTCCTGGTGGACGCTGGCCAGCCAAC

CCCAACTTTCTAAGTTTGACCTCGGATCAGGTAGGGATACCCGCTGAACTTAA

>OTU_458

GAAATGCGATAAGTAATGTGAATTGCAGAATTCAGTGAATCATCGAATCTTTGAACGCACCTTGCGCCTTTTGGTATTCC

GAAAGGCATGCCTGTTTCAGTGTCATGAAATCTCAATCTAATATGTTTTCTGAACATGTTAGGCTTGGACTTGGGTGTCT

GCCAGCAATGGCTCACCTCAAATGACTTAGTGGAACATCCCACATCAGTGTTAGACGTAATAAGTTTCGTCtctcCTTGT

GGTGATGACTGCTCAAAACCTGCCATCGCGCACCTTtttGACTTTGACCTGAAATCAGGTAGGGGCTACCCCGCTGAACT

TAA

>OTU_459

GAAATGCGATAAGTAATGTGAATTGCAGAATTCAGTGAATCATCGAATCTTTGAACGCACATTGCGCCCCTTGGTATTCC

GAGGGGCATGCCTGTTCGAGCGTCATTACACCACTCAAGCTATGCTTGGTATTGGGGCCGTCGGTCCTTAGTTGGGACAG

CGCCGTTAAAGACCTCGGCGAGGCCACTCCGGCTTTAGGCGTAGTAGAATTTATTCGAACGTCTGTCAAAGGAGAGGAAC

TCTGCCGACTGAAACCTTTATTtttCTAGGTTGACCTCGGATCAGGTAGGGATACCCGCTGAACTTAA

>OTU_460

GAAATGCGATAAGTAGTGTGAATTGCAGAATTCAGCGAATCATCGAATCTTTGAACGCACATTGCGCCCTTCGGTTATTC

CTTAGGGCATGCCTGTTCGAGCGTCATTTCAACCTTCAAGCCTGGCTTGGTGTTGGGCGCTGTCCCGCCTCCGcgcgcgG

ACTCGCCCCAAATGAATTGGCAGTCGCACCCTCCGAGCCGCGAGCGCAGCACAAGTCGcgcgGGCGGAACTCTGGggggA

CGGACGCTCCACAAGACCCTTTCTCAGTCTTGACCTCGGATCAGGTAGGGATACCCGCTGAACTTAA

>OTU_461

GAAATGCGATAAGTAATGTGAATTGCAGAATTCAGTGAATCATCGAATCTTTGAACGCACATTGCGCCCCTTGGTATTCC

GAGGGGCATGCCTGTTCGAGCGTCATTATAACCACTCAAGCCTCGGCTTGGTCTTGGGGTTCGCGGTCTCGCGTCCCTTA

AAATCAGTGGCGGTGCCGTCTGGCTCTAAGCGTAGTAATTCtctcGCTATAGGGTCCCGGCGGTTGCCTGCCAGAACCcc

ccATTttttACGGTTGACCTCGGATCAGGTAGGGATACCCGCTGAACTTAA

>OTU_462

GAAATGCGATACGTAGTGTGAATTGCAGAATTCAGTGAATCATCGAATCTTTGAACGCACATTGCGCCCTTTGGTATTCC

TTAGGGCATGCCTGTTCGAGCGTCATCTACACCTTCAAGCACTGCTTGGTGTTGGGTGCCTGTCCCGCCCCGcgcgTGGA

CTCACCTCAAATCCATTGGCGGCCcccACGTCGGCTCCGAGCGCAGCAGAAACGCGAACTCGTGGGCCCGGCGGGGTGGC

TCCCAGAAGCTACATCAACCATTTTGACCTCGGATCAGGTAGGGATACCCGCTGAACTTAA

>OTU_463

GAAATGCGATAAGTAATGTGAATTGCAGAATTCAGTGAATCATCGAATCTTTGAACGCACATTGCGCCCCGTGGTATTCC

GCGGGGCATGCCTGTTCGAGCGTCATTTCACCACTCGAGTCTGACTCGGTATTGGGCGCCGCGATACGTCGcgcgcCTCA

AAGTTTCCGGCTGGGCGACCCGTCTCCGAGCGTTGTGGCATCAAAGTCTCGCTAGGGAGCCGCGGGTGGCGTTGGCCGTT

AAATACCCCACCAAAGGTTGACCTCGGATCAGGTAGGGATACCCGCTGAACTTAA

>OTU_464

GAAATGCGATAAGTAATGTGAATTGCAGAATTCAGTGAATCATCGAATCTTTGAACGCACCTTGCGCTCCTTGGTATTCC

GAGGAGCATGCCTGTTTGAGTGTCATTAAATTCTCAACCTTCCAGCTTTTATTAGCTTGGTTAGGCTTGGATGTGGgggT

TGCAGGCTTCATTACTGAGGTCAGCTCTCCTTAAATACATTAGCGGAACTTTTGTGAACCGTCTATTGGTGTGATAATTA

TCTACGCCGTGGATGTGAAGCAGCTTTTATGAGGTTCAGCTTCTAACCGTCCATTGACTTGGACAAATTTTGACAATTTG

ACCTCAAATCAGGTAGGACTACCCGCTGAACTTAA

>OTU_465

GAAATGCGATAAGTAATGTGAATTGCAGAATTCAGTGAATCATCGAATCTTTGAACGCACATTGCGCCCTTTGGTATTCC

GAAGGGCATGCCTGTTCGAGCGTCATTATCAACCCTCAAGCTCGGCTTGTTATTGGGTCCTTATCGTTAAAGATAGGCCC

GAAAGATAATGGCGGCGTCATAAATGACCCCAGATGCAGCGAGCTTATAGCATACATCGAAAGGTTtttATGGCCCGGCC

TTAACGAGAAGCAATTCTCAATTTATTTAAAGGTTGACCTCGGATCAGGTAGGAATACCCGCTGAACTTAA

>OTU_466

GAAATGCGATAAGTAGTGTGAATTGCAGAATTCAGTGAATCATCGAATCTTTGAACGCACATTGCGCCCCTTGGTATTCC

ATGGGGCATGCCTGTTCGAGCGTCATTTGTACCCTCAAGCTTTGCTTGGTGTTGGGTGTTTGTCCTCTCCTAGCGTTTGG

ACTCACCTTAAAGTAATTGGCAGCCAGTGTTTTGGTATAGAAGCGCAGCACATGTCGCAATTCTAGCCCTACACTTGCGT

CCACAAGCCTATTTCACGTTTGACCTCGGATCAGGTAGGGATACCCGCTGAACTTAA

>OTU_467

GAAATGCGATAAGTAATGTGAATTGCAGAATTCAGTGAATCATCGAATCTTTGAACGCACCTTGCGCTCCTTGGTATTCC

GAGGAGCACGCCTGTTCGAGTGTCGTGAAACTCTCAAGCTAGATGCTTTAGTGTCATCTGCTTGGTTATTGGACTCTGCT

GTCTCCTTGTCTGGAACGGCTGGTCTCAAATGTATTAGCTGGCTCTTGTTGTGGGAATTGGTTCTACTCAGCGTGATAAT

atatGACCGCTGAGGACATCtctcGGGATGGCCAAGCCTGCCTTGAACTGCTTCTAACTCATCATTCATGATATTtttCA

ACTTTGACCTCGAATCAGGCGGGACTACCCGCTGAACTTAA

>OTU_468

GAAATGCGATAAGTAGTGTGAATTGCAGAATTCAGTGAATCATCGAATCTTTGAACGCACATTGCGCCCTCCGGTATTCC

GTTGGGCATGCCTGTTCGAGCGTCATTAGGAACTTCAAGCTCAGCTTGGTGTTGGGTGCTTGTCCCCGCCCCGCGGTGGA

CTCACCTCAAATGCATTGGCGGCCGGTATGTTGGCTACAGCGCAGTACAACGCGTCTGGCGTCTTGATATACTGGTCCTC

CAGAAGCCCCTTCTTTTGACTTGACCTCGGATCAGGTAGGGATACCCGCTGAACTTAA

>OTU_469

GAAATGCGATAAGTAGTGTGAATTGCAGAATTCAGTGAATCATCGAATCTTTGAACGCACATTGCGCCCTTTGGCATTCC

ATAGGGCATATCTGTTCGAGCGTCATTTGGACCCTCAAGCCCTGCTTGGTGTTGGGTGCTTGTCCCGCCTCTGCgcgcAG

GACTCGCCTTAAATCCATCGGCGCAGCACTGTCAGCCTCGAACGCAGCAGAGATGTGTCTCGAAGCTGGGAGCCTGCATG

CCGTAGCCTCATTTCGCAAGCTTGACCTCGGATCAGATAGGGATACCCGCTGAACTTAA

>OTU_470

GAAATGCGATACGTAGTGTGAATTGCAGAATTCAGTGAATCATCGAATCTTTGAACGCACATTGCGCCCTTTGGTATTCC

AAAGGGCATGCCTGTTCGAGCGTCATTTGTACCTTCAAGCTTTGCTTGGTGTTGGGCGTCTTttttttttGTCTTGGGGC

CTTTTGTGCCCTGAGACTCGCCTTAAAACGATTGGCAGCCGGCCTACTGGTTTCGGAGCGCAGCACATTtttGCGCTTGC

AATCAGCAAAAGAGGCCAGCCATCCATCAAGACCATTCTTCTCACTTTTGACCTCGGATCAGGTAGGGATACCCGCTGAA

CTTAA

>OTU_471

GAAATGCGATAAGTAATGTGAATTGCAGAATTCAGTGAATCATCGAATCTTTGAACGCACATTGCGCCCCTTGGTATTCC

GAGGGGCATGCCTGTTCGAGCGTCATTACAACCCTCAAGCTCTGCTTGGTATTGGGCGTCGTCCTCTTGCGGACGCGCCT

CAAAGACCTCGGCGATGGCGTCTAGCCCTCAAGCGTAGTAGAATACACCTCGCTTTGGAGTGGTTGGCGTTGTCCGCCGG

ACGAACCTTCTGAATTTTCTCAAGGTTGACCTCGGATCAGGTAGGGATACCCGCTGAACTTAA

>OTU_472

GAAATGCGATACGTAATGTGAATTGCAGAATTCAGTGAATCATCGAATCTTTGAACGCACCTTGCACTCTTTGGTATTCC

GAAGAGTATGTCTGTTTGAGTGTCATGAAACTCTCAACCcccTCATTTTGTAATGAAATGAGTTGTGGGCTTGGTTTATG

GCTGTTGCCGGCTTAAGTGCCGACTCAGCTGAAATACACGAGCAACCCAGTTTGAATACTTGACGGCTTGACTCGGCGTA

ATAATTATTATCGCTGAGGACGTCTACCTTTCAAATCGTTAGTGGTGCTTCTAATGCTTCATCTAGAACTTTTAAGCTTT

AGACCTCAAATCAGTCAGGACTACCCGCTGAACTTAA

>OTU_473

GAAATGCGATAAGTAATGTGAATTGCAGAATTCAGTGAATCATCGAATCTTTGAACGCACCTTGCGCCTCTTGGTATTCC

GAGAGGCATGCCTGTTTGAGTGTCTTATAAAATCAATCCCCACGGGTTTCTGACCCGGCGGTGGACTTGGAGTTGGGCGT

CTGCCGGTCACACGGCTCGCCTCAAAGACCTTAGTGGGAACATCGGCCTATGGCTTGACGTAATAAGTTTCGTCTCGCAC

GGTCGGTGACGCCTGCTCACAACCAGCCCTCGGGCAATTACTTtttGCTAGACCTCAAATCAGGTAGGACTACCCGCTGA

ACTTAA

>OTU_474

GAATTGCGATAAGTAATGTGAATTGCAGATTCTCGTGAATCATTGAATTtttGAACGCACATTGCGCCCTCTGGTATTCC

AGGgggCATGCCTGTTTGAGCGTCATTTCCTTCTCAAAATCTCGATTTTGGTTGTGAGTGATACTCTGTTACAGGGTTAA

CTTGAAAGTGCTATTGCCCTAGCTACTCTTttttttACTTGCTAAGAAaaaGATTtttGGATAATTTCAATGTATTTAGG

TATTTATACCGACTTTCATTGGATGCTGAGAGTCTTGTCTAAGCGCTTTTGTGAGATTTGAGCAGAAGGGATTAACAGTA

TTCATAAAGTTTGACCTCAAATCAGGTAGGATTACCCGCTGAACTTAA

>OTU_475

GAAATGCGATACGCAATGCGAATTGCAGAAATCCGCGAGACATTAGATTTTCGAACGCACTCTGCGCCAAGGGTCAGATA

CCTTTGGCATACTTGTTTCAGCGTAGATGATATTAGAAAAGCTGAATGCGGTTGTTCTCCTTCCTGGATGAACTCCCGTT

AAGTCTGGACAAGCGTGGCGCGACGGCCTCGCACACGAAGTAGTCATTGTCTCATCGTGGACGATGATCTCGAGTGCTGA

GTACGGCGTGATGTATGTCCGCTTTGCCTTGTGAGCGAAAGTGACAAACTCAATACCTGAAATCAAGTAAGAAGACCCGC

TGGAGTTAA

>OTU_476

GAAATGCGATAAGTAATGTGAATTGCAGAATTCAGTGAATCATCGAATCTTTGAACGCACCTTGCGCCCCGTGGCTTTCC

ACGGGGCACGCCCGTTCGAGTGTCACGTCAACCTTCGCCAACCCGATAGCAATATCGGGAGAAGCGGACTTGGACGCTGC

CGGTCGCGAGATCGGCTCGTTTCGAAATGCATTAGCGGAGTGAAGGCGCGAGCGCGACGTTGATAATCGTCCACGCCTCG

CGAGCGTCTGACCTCGCTTCCAATCGCCGTGAGGCAATTTATCCGAGTTTGACCTCGAATCGGGTGGGACTACCCGCTGA

ACTTAA

>OTU_477

GAAATGCGATAAGTAATGTGAATTGCAGAATTCAGTGAATCATCGAATCTTTGAACGCACCTTGCGCCCTTTGGTATTCC

GAAGGGCATGCCTGTTTGAGTGTCATGAAACCTCACCCCTCCCGGGTTttttGCCCGGTTGGTCTGGTGGATTGGGCGCC

TGCCGATGTTATACTGGCTCGCCTGAAAAGCATAAGCGCCTTGGATGTAATACGTTTCATCCTCTCGGGTGGCTGATGAA

CCCCACATATCTCATGATCTGGCCTCAAATCAGGTAGGGCTACCCGCTGAACTTAA

>OTU_478

GAAATGCGATAACTAATGTGAATTGCAGAATTCCGTGAATCATCGAGTCTTTGAACGCACATTGCGCCcccTGGTATTCC

GGggggCATGCCTGTCCGAGCGTCATTGCTGCCCATCAAGCACGGCTTGtgtgtTGGGTCCTCGTCCccccccGGgggAC

GTGCCCGAAAGGCAGCGGCGGCACCGCGTCCGGTCCTCGAGCGTATGGGGCTTTGTCACCCGCTCTGCAGGCCCGGCCGG

CGCTGGCCGACGCGAAAGCAACCATTttttCTCCAGGTTGACCTCGGATCAGGTAGGGATACCCGCTGAACTTAA

>OTU_479

GAAATGCGATAAGTAATGTGAATTGCAGAATTCAGTGAATCATCGAATCTTTGAACGCACATTGCGCCCATCAGTATTCT

GGTGGGCATGCCTGTTCGAGCGTCATTTCAACCCTTAAGCCTAGCTTAGTGTTGGGAATCTACTGTACTGTAGTTCCTTA

AAGACAGTGGCGGAGCGATAGTTGTCCTCTGAGCGTAGTAAATTTATTTCTCGCTTCTGCAAGGCTCTGTCCTCCCGCCA

TAAAACCcccAATTttttAGTGGTTGACCTCGGATCAGGTAGGAATACCCGCTGAACTTAA

>OTU_480

GAAATGCGATAAGTAATGTGAATTGCAGAATTCAGTGAATCATCGAATCTTTGAACGCACATTGCGCCCGCCAGTATTCT

GGCGGGCATGCCTGTCCGAGCGTCATTTCAACCATCAAGCCcccGGGCTTGTGTTGGGGACCTGCGGCTGCCCGCAGGCC

CTGAAAACCAGTGGCGGGCTCGCTGTCACACCGAGCGTAGTAGTAGCAATCACCTCGCTCAGGGCGTGCTGCGGGTTCCG

GCCGTTAAACAGCCTTACAAACCCCAAGGTTGACCTCGGATCAGGTAGGAAGACCCGCTGAACTTAA

>OTU_481

GAAATGCGATACGCAATGCGAATTGCAGAATTCCGCGAATCATCAGATTCTTGAACGCAACTGGCGCTGGAGGGATATCC

TTCCAGCATGTCTGTTTCAGTGCCTGAAAACTTCCAAATCAATTTAATGTGTTTGACGAGGAGCATTTCTTTATGGAGTG

CCTTTTCCAAGTCTTACATCAAAGATGTATATGTGGGTCTTTGGATCCATCGAATTGGTCACTGATTCTTCGGTGATTTC

GATTGGATTTCACCAATTACTCGTATACATATAGAAATTAGACAATACATACTTTATCATCTGCATCTGAAATCAGGCAA

GAAAACCCGCTGAACTTAA

>OTU_482

GAAATGCGATAAGTAGTGTGAATTGCAGAATTCAGTGAATCATCGAATTtttGAACGCACATTGCGCCCCTTGGTATTCC

ATGGGGCATGCCTGTTCGAGCGTCATTTGTACTCTCAAGCTCTGCTTGGTGTTGGGTGCTTGTCCTTTCATTACTGACTG

GACTCACCTTAAATGAATTGGCAGCCAATGATTTAGTCTTGGAGCGCAGCACAATTTGCGTTtttGGTCTTAGCATTTGC

ATCCAGTAAGCAATatatCACTTTTGACCTCGGATCAGGTAGGGATACCCGCTGAACTTAA

>OTU_483

GAAATGCGATACGTAGTGTGAATTGCAGAATTCAGTGAATCATCGAATCTTTGAACGCACATTGCGCCCTTTGGTATTCC

AAAGGGCATGCCTGTTCGAGCGTCATTTGTACCCTCAAGCTTTGCTTGGTGTTGGGCGTCTTATTGTCtctcCGTCTCGG

GGAGACTCGCCTTAAAACAATTGGCAGCCGGCCTACTGGTTTCGGAGCGCAGCACAAATTTGCGCTTGCAATCAGCCAAG

GGCGGCATCCATGAAGCCTTttttCtctcACATTTTGACCTCGGATCAGGTAGGGATACCCGCTGAACTTAA

>OTU_484

GAAATGCGATAAGTAGTGTGAATTGCAGAATTCAGTGAATCATCGAATCTTTGAACGCACATTGCGCCCCTTGGTATTCC

ATGGGGCATGCCTGTTCGAGCGTCATTTGTACCCTCAAGCTATGCTTGGTATTGGGCGATTTTGTCCGGCTCCCACGCCT

GGACTCGCCTCAAAGCGATTGGCAGCCGGCGTTtttGATGGCAGTGGAGCGCAGCACATTTTGCGCTCCGGATGCCcccG

TAAAGAAAACGACGGCGTTCCATGAAGCCATGTTTCTCACGTTTGACCTCGGATCAGGTAGGGATACCCGCTGAACTTAA

>OTU_485

GAAATGCGATAAGTAATGTGAATTGCAGAATTCAGCGAATCATCGAATCTTTGAACGCACATTGCGCCTCCGGGTATTCC

TGGAGGCATGCCTGTTCGAGCGTCGTTTAGACCATGAGGCTTTGCCTTGCGATGACGTTTGGGAAGTTTTCCTTTATGGA

GAATCCATCCGTTTAAACATATTGGCATCTGAAATTGGCCCAGCACAGCAAAATGTGTTTTGTGAGTTAATAGAAAGATG

CCTTGTATAATTCAATGTTTTAGCAATAGAACATGTATTGGTCGACCTCGGATCAGGCAAGATTACCCGCTGAACTTAA

>OTU_486

GAAATGCGATAAGTAGTGTGAATTGCAGAATTCAGTGAATCATCGAATCTTTGAACGCACATTGCGCCCCTTGGTATTCC

AGGgggCATGCCTGTTCGAGCGTCATTTGTACCCTCAAGCTCTGCTTGGTGTTGGGTGATTGTCCGGCCTTTGCGCCTGG

ACTCGCCTTAAAGCAATTGGCAGCCGGTGTTTTGGTAGTAGAGCGCAGCACATTTTGCGCCCTATCTCCTCAACGAAGGC

GTCCATGAAGCGCACATCTCACGTTTGACCTCGGATCAGGTAGGGATACCCGCTGAACTTAA

>OTU_487

GAAATGCGATAAGTAATGTGAATTGCAGAATTCAGTGAATCATCGAATCTTTGAACGCACATTGCGCTCTTTGGTATTCC

GGAGAGCATGCCTGTTTGAGAATCAGTAAATTCATCAATCCAAGGTTATTGGAATTTATTCCTTGAACCTTTGGGTTGGA

TCTGGGCACTTTGCTGCTTCGGCAGCAATAGCCTGAAATTGAATGTCACGCTTTCATGGACTGAAAAGTCTTGAGCAAAC

GCATGAATGGGGAAACTCAGAAGTACGCTAGCTTGAGGCCTTGATTTTCTTGATTGTGCGACTCAACTGATCAATCAGTA

TTTGCCGGTATCAACCAGCTCATTCTGAACACTTGGTCTCAAATCAGGTAGGAAAACCCGCTGAACTTAA

>OTU_488

GAAATGCGATAAGTAGTGTGAATTGCAGAATTCAGTGAATCATCGAATCTTTGAACGCACATTGCGCCCCTTGGTATTCC

ATGGGGCATGCCTGTTCGAGCGTCATCTACACCCTCAAGCTCTGCTTGGTGTTGGGCGTCTGTCCCGCCTCTGCgcgcgG

ACTCGCCCCAAATTCATTGGCAGCGGTCCTTGCCTCCTCTCGCGCAGCACAATTGCGTGGCGGggggggCGCGGTTCGCG

TCCACGAAGCAACATTTACCGTCTTTGACCTCGGATCAGGTAGGGATACCCGCTGAACTTAA

>OTU_489

GAAATGCGATAAGTAATGCGAATTGCAGAATTCAGTGAGTCATCGAATCTTTGAACGCACATTGCGCCCTTTGGTATTCC

GAAGGGCATGCCTGTTCGAGCGTCATTATCAACGTATCAAGCTTGGCTTGTCGTTGGGCCCTTTGTCACCTGGTGATAGG

TCCAAAAGATAATGACCGGTGTCGTAAAGACTCTATATGCAACGAGCTTATGACAGGCACGCATTTAGTAGCTATAATGA

CCCGGTTTTCACCCCTTTATTTCTCAAGGTTGACCTCGGATCAGGTAGGAATACCCGCTGAACTTAA

>OTU_490

GAAATGCGATAAGTAATGTGAATTGCAGAATTCAGTGAATCATCGAATCTTTGAACGCATCTTGCGCTCCTTGGTATTCC

GAGGAGCATGCCTGTTTGAGTGTCATTATATTCTCAACTCTCCAATACTTTGTTGTAAAGGAGAGCTTGGATTGTGGAGG

TTTGCTGGCCCCTTACTTGGGGTCAGCTCCTCTGAAATGCATTAGCGGAACCGTCTGCGATCTGCCACAAGTGTGATAAC

TTATCTACACTGGCGAGGGGATTGCTTTCTGATGTTCAGCTTCTAATCGTCTAAGGACAATTTCTTGAATGCTTGACCTC

AAATCAGGTAGGACTACCCGCTGAACTTAA

>OTU_491

GAAATGCGATAAGTAATGTGAATTGCAGAATTCCGTGAATCATCGAATCTTTGAACGCACATTGCGCCCCTGGCATTCCG

GggggCATGCCTGTCCGAGCGTCATTTCTGCCCTCAAGCGCGGCTTGtgtgtTGGGTGTGGTCCccccGGTGTTGGgggg

ACCTGCCCGAAAGGCAGCGGCGACGTCCCGTCTAGGTCCTCGAGCGTATGGGGCTTTGTCACCCGCTCGGGAGGGGCCTG

CGGGCGTTGGCCACCCACGATATTtttttACCGTTGACCTCGGATCAGGTAGGAGTTACCCGCTGAACTTAA

>OTU_492

GAAATGCGATACTTGGTGTGAATTGCAGAATCCCGTGAACCATCGAGTCTTTGAACGCAAGTTGCGCCCAAAGCCATTAG

GCCGAGGGCACGCCTGCCTGGGTGTCAcacaTCGTCATCCCCATGCAAAAGCGCATCGGGGCGAAAGGTGGCCTCCCGCG

ACacacCCGTCGTGGTTGGTCGAAAACCAAGGTAACGTCGGGGTCCcccccGCCAGAAACGGTGGATGAGCAACGCTCGA

GACCAACAACGGCGGCGGCGGAGACCCGGCAATCCAATCGATCGATGACCCTACAGCGTTCCTTGCTTAATAAATAGGCA

AAGACGCTCTTAACGAGACCTCAGGTCAGGCGGGGCTACCCGCTGAGTTTAA

>OTU_493

GAAATGCGATAAGTAATGTGAATTGCAGAATTCAGTGAATCATCGAATCTTTGAACGCACATTGCGCCcccTGGTATTCC

GGgggCATGCCTGTTCGAGCGTCATTTCACCACTCAAGCCTCGCTTGGTATTGGGCAACGCGGTCCGCCGCGTGCCTCAA

ATCGACCGGCTGGGTCTTCTGTCCCTAAGCGTTTGTGGAAACTATTCGCTAAAGGGTGCTCGGGAGGCTACGCCGTAAAA

CAAACCCATTTCTAAGGTTGACCTCGGATCAGGTAGGGATACCCGCTGAACTTAA

>OTU_494

GAAATGCGATAAGTAATGTGAATTGCAGAATTCAGTGAATCATCGAATCTTTGAACGCACATTGCGCCCTTTGGTATTCC

GGAAGGGCATGCCTGTTCGAGCGTCATTACAACCCTCAAGCTCTGCTTGGTATTGGGCACCGTCCTTTGCGGGCgcgcCT

CAAAGACCTCGGCGGTGGCGGTCTTTGCCTCAACGCGTTAGTAGAACATACATCTCGCTTCGGAGCGCAGGGCGTCGCCC

GCCGGACGAACCTTCTGAACTTTTCTCAAGGTTGACCTCGGATCAGGTAGGGATACCCGCTGAACTTAA

>OTU_495

GAAATGCGATAAGTAATGTGAATTGCAGAATTCAGTGAATCATCGAATCTTTGAACGCACCTTGCGCCTCTTGGTATTCC

GAGAGGCATGCCTGTTTGAGTGTCATGAAATCTCAATCCCCTCGGGTTTTAGGACCCGGGTCGGACTTGGACATGGGCGT

CTGCCGGTTAAACGGCTCGCCTCAAATGACTTAGTGGATCtctcAGCATCCGTGACAGACGTAATAAGTTTCGTCTTGTC

CCTTGCAGATGAGTCCGCTCACAACCTGCCATCGCGCACTTTTAGACTCTGACCTCAAATCAGGTAGGACTACCCGCTGA

ACTTAA

>OTU_496

GAAATGCGATAAGTAATGTGAATTGCAGAATTCAGTGAATCATCGAATCTTTGAACGCACATTGCGCCCGCTAGTATTCT

GGCGGGCATGCCTGTCCGAGCGTCATTTCAACCCTCAGGCCCTGGTTGCCTGGTGTTGGGGCgcgcgTCCcccAGCGGGC

GCGGGCCCCGAAAGTCAGTGGCGGGCTCGCCAGGACTCCGAGCGCAGTAGTTTACACCTCGCTGCGGAGCGCCTGGCGGG

TTTTACCCAGCCGTAAAACACCCCAAATCTTAAAGGTTGACCTCGGATCAGGTAGGAATACCCGCTGAACTTAA

>OTU_497

GAAATGCGATAAGTAGTGTGAATTGCAGAATTCAGTGAATCATCGAATCTTTGAACGCACATTGCGCCCTGTGGTATTCC

GCAGGGCATGCCTGTTCGAGCGTCATTTAACCcccTCAAGCTCTGCTTGGTGTTGGGCGTTTGTCTTAGCTTCTAGCATA

GACTCGCCTTAAAATTATTGGCAGTCACCTctctGGCTTCGAGCGCAGCACTATTGCGCCCAGTCTTTGGGAGTGTGGTT

ACCCAGAAGCCCTCTTTTCGGTTGACCTCGGATCAGGTAGGGATACCCGCTGAACTTAA

>OTU_498

GAAATGCGATAAGTAATGTGAATTGCAGAATTCAGTGAATCATCGAATCTTTGAACGCATCTTGCGCTCCTTGGTATTCC

ATGGAGCATGCCTGTTTGAGTGTCACGTAAACCCTCACCCTTGCGATGTAACAGTCGCCCGTGGTGGACTTGGACTGTGC

CGTAACCGGCTCGTCTTGAAATGCATTAGCTGGCGCTTTTAGAGTGCTGGGCGACGGTGTGATAATTATCTGGGCCAATG

CCTTAGGCCTCTTCAGCGGTGCTGCTTACAGCCGTCCCTCTGTGGACACATTATTtttAAAGCTTTGGCCTCAAATCAGG

TAGGACTACCCGCTGAACTTAA

>OTU_499

GAAATGCGATAAGTAGTGTGAATTGCAGAATTCAGTGAATCATCGAATCTTTGAACGCACATTGCGCCCCTTGGTATTCC

ATGGGGCATGCTTGTTCGAGCGTCATTTGTACTCTCAAGCTTTGCTTGGTGTTGGGTGCTTGTCCTCTCCACTGCGGTTG

GACTCACCTTAAATTCATTGGCAGCCGATatatTGGTTTTGAAGCGCAGCACATGTAGCGATTCAGGCTGGATatatCAG

CGTCCAGCAAGGCTATTTAACACTTTTGACCTCGGATCAAGTAGGGATACCCGCTGAACTTAA

>OTU_500

GAAATGCGATAAGTAATGTGAATTGCAGAATTCAGTGAATCATCGAATCTTTGAACGCATATTGCACCTTCTGGTATTCC

GGGAGGTATGCCTGTTCGAGCGTCATTGCGACCAACAAGCTTTAAGCTTGGTATTGGATGTCACTATTTCAGGTGCATCT

TAAAGTCAAGGAGCAGGACTCTCAGCTTCCAAGCGCAGTAATTTAATCTCGCTTCTGGAGCCTGGATGATGTTCTGCTGA

ACCGTTAAATTTATCATGTTTGACCTCGGATCAGGTAGGGATACCCGCTGAACTTAA

>OTU_501

GAAATGCGATAAGTAGTGTGAATTGCAGAATTCAGTGAATCATCGAATCTTTGAACGCACATTGCGCCCCTTGGTATTCC

GGggggCATGCCTGTTCGAGCGTCATCTACACCCTCAAGCTCTGCTTGGTGTTGGGCGTCTGTCCCGCCTCCGcgcgTGG

ACTCGCCCCAAATCCATTGGCAGCGGTCCTTGCCcccTCTCGCGCAGCACATTGCGCTTCACGAGGgggCGTGGGTCGCG

TCCACGAAGCACCATCACCGTCTTTGACCTCGGATCAGGTAGGGATACCCGCTGAACTTAA

>OTU_502

GAAATGCGATAAGTAATGTGAATTGCAGAATTTAGTGAATCATCGAATCTTTGAACGCACATTGCGCCCCTTGGTATTCC

GAGGGGCATGCCTGTTCGAGCGTCATAACACCcccTCCAGCTGCCGTTGtgtgGCTGCGGTGTTGGGGCTCGTCGCGATG

CGGCGGCCCTTAAAGACAGTGGCGGTCCCGGCGTGGGCTCTACGCGTAGTAACTTGCTTCTCGCGACAGAGTGACGCCTG

GTGGCTTGCCAGAACAACCCCTATTGGGTCCAGTCACATGGATCACAGGTTGACCTCGAATCAGGTAGGAATACCCGCTG

AACTTAA

>OTU_503

GAATTGCGATAAGTAATGTGAATTGCAGATACTCGTGAATCATTGAATTtttGAACGCACATTGCGCCCTTGAGCATTCT

CAGGGGCATGCCTGTTTGAGCGTCATTTCCTTCTCAAAAGATAATTTATTATTttttGGTTGTGGGCGATACTCAGGGTT

AGCTTGAAATTGGAGACTGTTTCAGTCTTttttAATTCAACACTTAGCTTCTTTGGAGACGCTGTTCTCGCTGTGATGTA

TTTATGGATTTATTCTTTTACTTACAAGGGTAAATGGGTAACGTACCTTAGGCAAAGGGTTGCTTTTAATATTCATCAAG

TTTGACCTCAAATCAGGTAGGATTACCCGCTGAACTTAA

>OTU_504

GAAATGCGATAACTAGTGTGAATTGCAGATTTCCGTGAATCATCGAGTCTTTGAACGCACATTGCGCCCCTTGGTTTACC

AGGgggCATGTCTATCCGAGCGTCGTTACACCCCTCAAGCCTGGCTTGGTCTTGGGGCTCGTCCTGCCTAGCAGGGCCGC

CCTTAAATGTAACTCGACGTGCCTTTTGTTCGGGAGCGCAGCAGTTTTGCGGCTTTCGCTGGTCTCGTAGAGCCGCCTCT

AGCGGATCtctctGGTTGACCTCGGATTAGATAAGGATACCCGCTGAACTTAA

>OTU_505

GAAATGCGAAAAGTAGTGTGAATTGCAGAATTCAGTGAATCATCGAATCTTTGAACGCACATTGCGCCCCTTGGTATTCC

ATGGGGCATGCCTGTTCGAGCGTCATTTGTACCTTCAAGCATTGCTTGGTGTTGGGTGTTTGTCCCCGTTtttACACGTG

GACTCACCTTAAAGTGATTGGCAGCCGGCGTACTCGCCTCGGAGCGCAGCACATTTTGCGCCCCTTGACTTGAACGATGG

CGTCCATTAAGTCTACATTtttGCTCTTGACCTCGGATCAGGTAGGGATACCCGCTGAACTTAA

>OTU_506

GAAATGCGATAAGTAATGCGAATTGCAGAATCCGTGAGTCATCGAATCTTTGAACGCATATTGCGCCCTCTGGTATTCCG

GggggCATGCCTGTTCGAGCGTCATTATCACCCCTCAAGCCCGGCTTGTTGTTGGATGCGGCGGCGGCCTTTAGTGGCGT

ACCGCCCGTCTCAAAGATAATGACGGCGTCTGTGAGGACTCCTGTACACTGAGCTTTCGGGCACGTACTAGGCTGATACT

CGCAGACCCGGTCTTTAGGGCTCGCTTCGGCGGCCCAACTTTTACCAAGGTTGACCTCGGATCAGGTAGGAATACCCGCT

GAACTTAA

>OTU_507

GAATTGCGATAAGTAATGTGAATTGCAGAATTCAGTGAATCATCGAATCTTTGAACGCAGCTTGCGCTCTTTGGTATTCC

AAAGAGCATGCCTTtttGAGTGTCGTTAATCTTCTTCAATTGCGTTtttGCAcacaAaaaTGCATGCGAGTCCTTGGGTT

TGATTGACATGCCCTGTCAATCTTACCTTAAATGTATGAGCTGGAGATCAGTCAGGTTGCTAAGGCATTTTAAACCTGCG

TAAACGGTCTCTGCTTtttAAATTAAACTATTAACTACTGGCCTCAAATAAGGTAGGACTACCCGCTGAACTTAA

>OTU_508

GAAATGCGATAAGTAATGCGAATTGCAGAATTCCAGTGAGTCATCGAATCTTTGAACGCACATTGCGCCCTTTGGTATTC

CGAAGGGCATGCCTGTTCGAGCGTCATTTTCACCCCTCAAGCCcccGGCTTGGTGTTGGACGGTTTGGTTTAGGTACCCC

TAGACCCCTCCTAAAGACAATGACGGCGGCTGTTGAACCcccGGTACACTGAGCTTCTTAACTGAGCACGTATCGGATCT

AGGGTTTAGCGGCACCCGGTCTATACCTTttttGCTTACAAGGTTGACCTCGGATCAGGTAGGAATACCCGCTGAACTTA

A

>OTU_509

GAAATGCGATAAGTAATGTGAATTGCAGAATTCAGTGAATCATCGAATCTTTGAACGCACATTGCGCCCGCCAGTATTCT

GGCGGGCATGCCTGTCTGAGCGTCATTTCAACCCTCAGCCcccGTCCGCGGGGCGCTGGCGTTGGGGATCGGCCGTCCTT

CGCGGCGGCCGGCCCCGAAACACAGTGGCGGTCTCCTGCAGACTCCCCTGCGTAGTAGCACTACCTCGCAGAAGGGACGA

GCGGGCTGGCCACGCCGTAAAACCcccAACTTCTCAAGGTTGACCTCAGATCAGGTAGGAATACCCGCTGAACTTAA

>OTU_510

AAAATGCGATACTTGGTGTGAATTGCAGAATCCCGTGAATCATCGAGTCTTTGAACGCAAGTTGCGCCCGAAGCCATTAG

GCCGAGGGCACGCCTGCCTGGGCGTCACGCACCCGTCGCCcccACCACCTCCCTCCcccTCCGGCACGGGAGGCGGAGAG

GgggCGGACATTGGCCTCCCGTGGGCGCCCCAGCCCGCGGTTGGCCGAAAATCGGTCCCGCGGCGACGTACGCCACGACG

AGCGGTGGATTTCGCACGGCTCGGCGTCGcgcgcgTCACGTCGCCTCAGGG

>OTU_511

GAAATGCGATAAGTAATGTGAATTGCAGAATTCAGTGAATCATCGAATCTTTGAACGCACATTGCGCCCCTTGGTATTCC

GAGGGGCATGCCTGTTCGAGCGTCATTACACCACTCAAGCTATGCTTGGTATTGGGCGTCGTCTTAAAGACCTCGGCGAG

GCCACTCCGGCTTTAGGCGTAGTAGAATTTATTCGAACGTCTGTCAAAGGAGAGGAACTCTGCCGACTGAAACCTTTATT

tttCTAGGTTGACCTCGGATCAGGTAGGGATACCCGCTGAA

>OTU_512

GAAATGCGATAAGTAGTGTGAATTGCAGAATTCAGTGAATCATCGAATCTTTGAACGCACATTGCGCCCCTTGGTATTCC

ATGGGGCATGCCTGTTCGAGCGTCATTTACACCCTCAAGCTCAGCTTGGTGTTGGGCGTCTGTCCCGCTTCGTGCGCGGA

CTCGCCCCAAAGGTATTGGCAGCGGTCCTTGCCAGCTTCTCGCGCAGCACATTGCGCTTCTCGAGGCTGGCGGATCGGCG

TCCACCAAGCCCACTTCCACAGTTTGACCTCGGATCAGGTAGGGATACCCGCTGAACTTAA

>OTU_513

GAAATGCGATACGTAATGTGAATTGCAGAATTCAGTGAATCATCGAATCTTTGAACGCACCTTGCGCTCTTGGGTATTCC

TAAGAGCATGTCTGTTTGAGTGTCGTGAATCtctcAAAACCcccTTTTGTTTAATTACGAAAGAGTTTTCGGGCTTTGGA

TGATGACCGTTTGCCGTTTTCATTAATGGCTCGGTCGAAATATAACAGCTGAAGCTAATAATCGTATTATTGCTTACTCT

GGACTTGGCGTGATAATTATTTCGCTAAAGAACGAGTTTGTAGACTTTTATTAAGCGGGCTTATAAAACACAACTTTTAC

ATTAGACCTCAAATCAGACAGGACTACCCGCTGAACTTAA

>OTU_514

GAAATGCAATAAATAATGTGAATTGCAGAATTCAGTAAATCATCAAATCTTTAAACGCACATTGCGCCCGCCAGCATTCT

AGCAGGCATGCCTATTTAAGCGTCATTTCAACCCTTAAGCTCCGCTTAGTGTTAGGGCCTTACAGCTGATGTAGGCCCTC

AAAAGTAGTAGCAAACCTTCTTAGAGCCTCCTTTGCGTAGTAACTTTACATCTCACACTAGGATCTAGAGGGACTCTTGC

TATAAAACCcccTAATTTTCTAAAGGTTAACCTTAGATCAAATAGGAATACCTGCTGAACTTAA

>OTU_515

GAAATGCGATAAGTAGTGTGAATTGCAGAATTCAGTGAATCATCGAATCTTTGAACGCACATTGCGCCCCTTGGTATTCC

ATGGGGCATGCCTGTTCGAGCGTCATTTGTACCCTCAAGCTCTGCTTGGTGTTGGGTGTTTGTCCACTGTCGTGGACTCG

CCTTAAAGTCATTGGCAGCCAGTGTTTTGGTATTGAAGCGCAGCACATTTTGCGCCTCTAGCCTAGAGCACTCGCGTCCA

GTAAGCCTTCTTCCACTTTTGACCTCGGATCAGGTAGGGATACCCCGCTGAATTAA

>OTU_516

AAAATGCGATACTTGGTGTGAATTGCAGAATCCCGTGAACCATCGAGTTtttGAACGCAAGTTGCGCCCGAAGCCTTTTG

GCCGAGGGCACGTCTGCCTGGGCGTCACGCATCGCGTCGCCcccACCAATCTCTGTAAAGGGAACTCGTGTTTTGGgggC

GGATAATGGTCTCCCGTGCTCATGGCGTGGTTGGCCGAAATAGGAGTCCCTTCGATGGACGCACGAACTAGTGGTGGTCG

TAAaaaCCCTCGTCTTTTGTTTCGTGCCGTTAGTCGCAAGGGAAACTCTAAGAAAACCCCAACGTGTCGTCTCTTGACGA

CGCTTCGACCGCGACCCCAGGTCAGGCGGGACTACCCGCTGAGTTTAA

>OTU_517

GAAATGCGATAAGTAATGTGAATTGCAGAATTCAGTGAATCATCGAATCTTTGAACGCACATTGCGCCCACTAGTATTCT

GGTGGGCATGCCTGTTCGAGCGTCATTTCAACCCTCAAGCCTTAGTTGCTTGGTGTTGGGAGCTTATCTTGCCGAAAGGC

GGACAACTCCTTAAAACTATTGGCGGAGTCGCGGTGACCCCAAGCGTAGTAAATTCTTtttttCTCGCTTTAGGTGTTAA

CGCTGGCTTTCTAGCCGTTAAACCCTCTAAATTtttAGTGGTTGACCTCGGATCAGGTAGGACTACCCGCTGAACTTAA

>OTU_518

GAAATGCGATAAGTAGTGTGAATTGCAGAATTCAGTGAATCATCGAATCTTTGAACGCACATTGCGCCCTTTGGTATTCC

AAAGGGCATGCCTGTTCGAGCGTCATTTGTACCCTCAAGCTCTGCTTGGTGTTGGGCGTCTTTGTCTTCCCAGAAGACTC

GCCTTAAAGTCATTGGCAGCCGCCTACTGGTTTCGGAGCGCAGCACAAGTCGCGCTctctTCCAGCCAAGGTCAGCGTCC

ACAAAGCCTTtttttCAACTTTTGACCTCGGATCAGGTAGGGATACCCGCTGAACTTAA

>OTU_519

GAAATGCGATAAGTAATGTGAATTGCAGAATTCAGTGAATCATCGAATCTTTGAACGCACATTGCGCTCTTTGGTATTCC

GGAGAGCATGCCTGTTTGAGAATCAGTAAATTCATCAACCGTTTAATACCGGTTGGATATGGGCCCTTTGAGACCTCGCG

GTCACAATGGCCTGAAaaaaaaTGTTACGCAACCTTGAACCAACGGTTCTGGAGCATACGCATGAATGGGGAAACTCACC

AATACGTTTGTTCTTTATGAAGTGTTGGCACATTGGGAAGTGTGACTCAACTATTATCCAGGGTTGACTGGCGTTAGCAA

GCCTTCCCTGAACACTTGGTCTCAAATCAGGTAGGAAAACCCGCTGAACTTAA

>OTU_520

GAAATGCGATAAGTAATGTGAATTGCAGAATTCAGTGAATCATCGAATCTTTGAACGCACATTGCGCCCCTTGGTATTCC

GAGGGGCATGCCTGTTCGAGCGTCATTACACCACTCAAGCTATGCTTGGTATTGGGCGTCGTCCTTAGTTGGGCgcgcCT

TAAAGACCTCGGCGAGGCCACTCCGGCTTTAGGCGTAGTAGAATTTATTCGAACGTCTGTCAAAGGAGAGGAACTCTGCC

GACTGAAAACCTTTATTttttCTAGGTTGACCTCGGATCAGGTAGGGGATACCCCGCTGAACTTAA

>OTU_521

GAAATGCGATAAGTAATGCGAATTGCAGAATTTCCGTGAGTCATCGAATCTTTGAACGCACATTGCGCCCACTGGTATTC

CGGTGGGCATGCCTGTTCGAGCGTCATTATCCTCCCTCAAACCTCGGGTTTGGTGTTGGACCCAAGTTGtgtgAACAACT

GGTCTTAAAGACAATGACGGCGTCCGTGGGACCTCGGTGCAACGAGCTTTTAGGAGCACGCGTCGAGTTTCAAGGACCTT

CCGGGCCGGTCTCCTTTACCATTTACAAGGTTGACCTCGGATCAGGTAGGAATACCCGCTGAACTTAA

>OTU_522

GAAATGCGATAAGTAATGTGAATTGCAGAATTCAGTGAATCATCGAATCTTTGAACGCACATTGCGCCCCTTGGTATTCC

GGggggCATGCCTGTTCGAGCGTCATGTAAACCCTCAAGCCTAGCTTGGTGTTGGGGCCTGCTGTCACCGGCAGCCCTTA

AAATCAGTGGCGGTGCCACCTGGCTCTAAGCGTAGTAATTCTTCTCGCTCCAGGGTCCTGGTGGATGCTTGCTAACAACC

TCTGATTTTCTATGGTTGACCTCGGATCAGGTAGGGATACCCGCTGAACTTAA

>OTU_523

GAAATGCGATAAGTAGTGTGAATTGCAGAATTCAGTGAATCATCGAATCTTTGAACGCACATTGCGCCTCCTGGTATTCC

GGGAGGCATGCCTGTTCGAGCGTCATTAAATACCTCTCAAGCAATTGCTTGGTCGTGGAAGATGAGTATGCTTGCATTCT

CACTTCTGAAATTCAAAGGCGGATGATCTCATATCCCCAGGTGTAGTAAGTTTATCTTTTCGCTTGGTGCATGCGATTGT

CCTGCCGTTGACCcccATGTTTTCAAGGTTGACCTCGGATCAGGTAGGGATACCCGCTGAACTTAA

>OTU_524

GAAATGCGATAAGTAATGTGAATTGCAGAATTCAGTGAATCATCGAATCTTTGAACGCACATTGCGCCCGCCAGTATTCT

GGCGGGCATGCCTGTTCGAGCGTCATTTCAACCATCAAGCCcccGGCTTGTGTTGGGGACCTGCGGCTGCCGCAGGCCCT

GAAAAGCAGTGGCGGGCTCGCTGTCACACCGAGCGTAGTAGCATACATCTCGCTCTGGGCGTGCTGCGGGTTCCGGCCGT

AAAACCACCTTCATAACCCAAGGTTGACCTCGGATCAGGTAGGAAGACCCGCTGAACTTAA

>OTU_525

GAAATGCGATAAGTAGTGTGAATTGCAGAATTCAGTGAATCATCGAATCTTTGAACGCACATTGCGCCCCTTGGTATTCC

ATGGGGCATGCCTGTTCGAGCGTCATTTGTACCCTCAAGCTCTGCTTGGTGTTGGGTGTTTGTCCTGCCTCTGCGTATGG

ACTTGCCTGAAAGCAATTGGCAGCCGGCACATTAGCCTGTAGCGCAGCACATTTTGCGATGCTGGCTAAGGTTGTTGGCG

TCCATGAAGCCTATACCTTTGCATTGACCTCGGATCAGGTAGGGATACCCGCTGAACTTAA

>OTU_526

GAAATGCGATAAGTAATGTGAATTGCAGAATTCAGTGAATCATCGAATCTTTGAACGCATATTGCACCCTCTGGTATTCC

GGAGGGTATGCCTGTTTGAGTGTCATTAATCATCTCAAGGCAAAGGGATTCTTCTTtttGCAATTGGATGTTGAAGGTCT

TGCTGGCTTTCTTAGTTGGCTCCTTTTAAATACATTAGCAGGACTTtttGCAGGACTTGCTCCAGTTTGATACATTTTGC

TATCTGAGCTGTTGCTGGGCCAGCACCAAAGTGGTCTTCTGCTTCAAATTGTCTTTGGACACTACCTTGAACTTGACCTC

AAATCAGGTAGGACTACCCGCTGAACTTAA

>OTU_527

GAAATGCGATAAGTAATGTGAATTGCAGAATTCAGTGAATCATCGAATCTTTGAACGCACATTGCGCCCGCCAGTATTCT

GGCGGGCATGCCTGTTCGAGCGTCATTTCAACCCTCAAGCCcccGGGCTTGGTGTTGGGGACCGGCCACCGCGGACTTCT

GTCCGCAGGCCGCCcccTAAATCTAGTGGCGGTCACGCCGCGGCTTCCTCTGCGTAGTAGCACACCTCGCAGCCGGATCG

CGGTGCGGCCACGCCGTAAAACCCCAACTCTGAAATGTTGACCTCGGATCAGGTAGGAATACCCGCTGAACTTAA

>OTU_528

GAAATGCGATACGTAATGTGAATTGCAGAATTCAGTGAATCATCGAGTCTTTGAACGCACATTGCGCACTCTGGTATTCC

GGggggCATGCCTGTCCGAGCGTCATTGCTGCCCTCAAGCACGGCTTGtgtgtTGGGCCcccGTCCCGGTTCTTTAAGCC

GGGACGGGCCCGAAAGGCAGCGGCGGCACCGTGTCCGGTCCTCGAGTGTATGGGGCTCTGTCACCCACTCGTGTAGGTCC

GGCCGGCGGCCAGCCTCTTCAACCAAACTTTTAACCAGGTTGACCTCGGATCAGGTAGGGATACCCGCTGAACTTAA

>OTU_529

GAAATGCGATAAGTAATGTGAATTGCAGAATTCAGTGAATCATCGAATCTTTGAACGCACATTGCGCCCTTCGGTATTCC

GGggggCATGCCTGTTCGAGCGTCATTACACCACTCCAGCCTCGCTGGGCCTTGGGCGCCGCCGCCTCGCCGGCgcgcCC

CAATGTCTCCGGCTGAGCCGTCCGTCTCTAAGCGTTGCGACAACTCAATCCGCTTGTGGGACCGGGAGGTTCGACGCCGT

TAAACCTCAcacaAGGTTGACCTCGGATCAGGTAGGGATACCCGCTGAACTTAA

>OTU_530

GAAATGCGATAAGTAATGTGAATTGCAGATACAGTGAATCATCGAATCTTTGAACGCAAATGGCACTCTATGGTATTCCG

TAGAGTACGTCTGTTTGAGCGTCGCGAACATCTCCATAATTAGTTttttttAAATTGATTGTGGGTTTTGAGGTTGTCAT

ATAAACAATGACTCCCTTTAAAATAATTAGTGATGACCTTATGAATGGGTTAATACTGtgtgtTATAATGGATTACATCC

ATCACCAGTCAGagagTAATCTCGCCTTAGTAATTTGTAGTGATTGCTTCTAACTGCCATTGGCAAACAAACTGATCAAA

TCGACCTCAAATCAGATGGGATTACCCGCTGAACTTAA

>OTU_531

GAAATGCGATAAGTAATGTGAATTGCAGAATTCAGTGAATCATCGAATCTTTGAACGCACATTGCGCCCTCTGGTATTCC

GGAGGGCATGCCTGTTCGAGCGTCATTTACACCCCTCAAGCCTAGCTTGGTGTTGGGGAGGCTTGTTCGACCCCGCGAGG

GAgagagCAACCcccccGAAATGCAGCGGCGGTGCGGCCGGACCCCTTAGCGCAGTGACTTTCAAATCTCGCTACCGGgg

gCTCGTGCCTGCCTCGGCCGGACAACAACCCTTATTTTCTATACGATTGACCTCGGATCAGGTAGGGATACCCGCTGAAC

TTAA

>OTU_532

GAAATGCGATAAGTAGTGTGAATTGCAGAATTCAGTGAATCATCGAATCTTTGAACGCACATTGCGCCCTTTGGTATTCC

TTAGGGCATGCCTGTTCGAGCGTCATTTACAAATTCAAGCTCAGCTTGGTGATGGGTGTCTGTCCCGCCTTCGTGCGTGG

ACTCGCCTCAAATGCAGTTGGCAGCTTATCCCTCGGCTCTAAACGCAGCAGACTTGCGTCGAGCGTCGTGTGGACGGGCT

CTCCAGTAAGCAAACCccccAAAATTGACCTCGGATCAGGTAGGGATACCCGCTGAACTTAA

>OTU_533

GAAATGCGATAAGTAGTGTGAATTGCAGAATTCAGTGAATCATCGAATCTTTGAACGCACATTGCGCCCCTTGGTATTCC

ATGGGGCATGCCTGTTCGAGCGTCATCTACACCCTCAAGCTCTGCTTGGTGTTGGGCGTCTGTCCCGCCTTCGcgcgcgG

ACTCGCCCCAAATTCATTGGCAGCGGTCCTTGCCTCCTCTCGCGCAGCACAATTGCGTCTGCGGgggggCGTGGCCCGCG

TCCACGAAGCAACATTACCGTCTTTGACCTCGGATCAGGTAGGGATACCCGCTGAACTTAA

>OTU_534

GAAATGCGATAAGTAATGTGAATTGCAGAATTCAGTGAATCATCGAATCTTTGAACGCACATTGCGCCCCTTGGTATTCC

GGggggCATGCCTGTTCGAGCGTCATTACACCCCTCAAGCTCAGCTTGGTCTTGGGGCCTGCCCGTCACAGGGCAGCCCT

TAAAACTAGTGGCGGTGCCATCTGGCTCTACGCGTAGTAATTCTTCTCGCGATGGAGTCCCGGTGGTGTCTTGCCAGAAC

CCCAAACTTtttAATGATTGACCTCGGATCAGGTAGGGATACCCGCTGAACTTAA

>OTU_535

GAAATGCGATAAGTAGTGTGAATTGCAGAATTCAGTGAATCATCGAATCTTTGAACGCACATTGCGCCCTATGGTATTCC

GTAGGGCATGCCTGTTCGAGCGTCATTCAACCCTTCAAGCTCTGCTTGGTGTTGGGCGTCTGTCCCGCCTCACGGCGCGG

ACTCGCCTCAAATCTATTGGCGGCCGGCACGTTGGCTTCGAGCGCAGCAGAAACGCGAACTCGAGGCCCGGCGGATCGGC

TCCCAGAAGCTACCcccTATGAATTTGACCTCGGATCAGGTAGGGATACCCGCTGAACTTAA

>OTU_536

GAAATGCGATAAGTAATGTGAATTGCAGAATTCAGTGAATCATCGAATCTTTGAACGCACATTGCGCCcccTGGTATTCC

GGggggCATGCCTGTTCGAGCGTCATTTCACCACTCAAGCCTCGCTTGGTATTGGGCAACGCGGTCCGCCGCGTGCCTCA

ATCGACCGGCTGGGCTCTTCTGTCCCCTAAGCGTTGTGGAAACTATTCGCTAAAGGGTGCTCGGGAGGCTACGCCGTAAA

ACAAACCCATTTCTAAGGTTGACCTCGGATCAGGTAGGGATACCCGCTGAACTTAA

>OTU_537

GAAATGCGATAAGTAATGTGAATTGCAGAATTCCGTGAATCATCGAATCTTTGAACGCACATTGCGCCCTCTGGTATTCC

GGAGGGCATGCCTGTCCGAGCGTCATTGCATACCTTCAAGCACGGCTTGtgtgATGGGCTGTCGTCCCGACGATATACTG

TTTGGGACGGGTCCGAAAAGCAGTGGCGGCGTTGCGGTTTTCCGATCAAGCCTGAGTGTATGGGGATTCTATCAcacacT

CGCGGCGCGGACCGACGCTGGCCTATTTATTTATTCTTTCTTAGGTTGACCTCGGATCAGGTAGGGATACCCGCTGAACT

TAA

>OTU_538

GAAATGCGATAAGTAATGTGAATTGCAGAATTCAGTGAATCATCGAATCTTTGAACGCACATTGCGCCCTTTGGTATTCC

GAAGGGCATGCCTGTTCGAGCGTCATTATCAACCATCAAGCTCTGCTTGGCATTGGGTGTCAACCTCCCCTAACCGGGCG

GTCGCGCCTCAAACTGTTCGGCGGTGGCTCAGGGCCTCAAGCGTTAGTAATACTTCCCGCTTCAGAGAAACTGAGTTGCC

TGCCTCTAGAAACCCACATCTTAAGGTTGACCTCGGATCAGGTAGGGATACCCGCTGAACTTAA

>OTU_539

GAAATGCGATAAGTAATGTGAATTGCAGAATTCAGTGAATCATCGAATCTTTGAACGCACATTGCGCCCCTTGGTATTCC

GAGGGGCATGCCTGTTCGAGCGTCATTACACCACTCAAAGCTATTGCTTTGGTATTGGGCGTCGTCCTTAGTTGGGCgcg

cCTTAAAGACCTCGGCGAGGCCACTCCGGCTTTAGGCTGTAGTAGAATTTATTCGAACGTCTGTCAAAGGAGAGGAACTC

TGCCGACTTGAAACCTTTTATTttttCTAGGTTGACCTCGGATCAGGTAGGGATACCCGCTGAACTTAA

>OTU_540

GAAATGCGATAAGTAGTGTGAATTGCAGAATTCAGTGAATCATCGAATCTTTGAACGCACATTGCGCCCCTTGGTATTCC

ATGGGGCATGCCTGTTCGAGCGTCATTTGTACCCTCAAGCTCTGCTTGGTGTTGGGTGTTTGTCCCGCCTTTTGTGCGTG

GACTCGCCTTAAAGCGATTGGCAGCCGGCATATTGGCCTTGGAGCGCAGCACATTTTGCGCTTCTTGTCAATTATTGTTG

GCATCCATCAAGTACAATTCTAACTTTTGACCTCGGATCAGGTAGGGATACCCGCTGAACTTAA

>OTU_541

GAAATGCGATAAGTAATGTGAATTGCAGAAGTGAATCATCGAATCTTTGAACGCATCTTGCGCTCCCGGCAGATCTAATC

TGGGGAGCATGCCCGTCTGAGGGCCGCGAATGGTTTCGAACGTTAGCTTttttCGTAAGAGAATAGCTGACGGATCGGTA

TTGAGGGTTTTGCCATTTACAGTGGCTCCCTCGAAATACATTAGCATGACATTAGAATTTCGAGTGGGTGAATGGCCGAC

CGGGTTTTGATAACATCAAGGCTTCGGTGGCAGGACATCACTTGAGAAAAGTTCATCGCTTCTAAACCCGCCCTTACTTG

CCATTAGGAAGTAATGGGATATTTTATTTTATTTGATCGGCCTCAGATCGGTAGGACTACCCGCTGAACTTAA

>OTU_542

GAAATGCGATACTTGGTGTGAATTGCAGAATCCCGTGAACCATCGAGTCTTTGAACGCAAGTTGCGCCTGAAGCCATTAG

GTTGAGGGCACGCCTGCCTGGGTGTCAcacaTTGTCACCCCAATGCAAATGTGCATTGAGGTGAAAGTTGGCTTCCCGCG

AGGCATTCCTCGTGGTTGGTTCAAAACGAAGTTAATCGCGAAGTCCCTCGTCATAAATGGTGGATGAGTAAATCTCGAGA

CCAATCGTGACTGTGGTGCTTTGGGGATGTAATTGGTTGGCTCTGTTGTGTTTTGTGTTCATGTTGAAGAAGACGCTTTT

ATCGAGACCTCAGGTCAGGCGGGGCTACCCGCTGAGTTTAA

>OTU_543

GAAATGCGATAAGTAATGTGAATTGCAGAATTCAGTGAATCATCGAATCTTTGAACGCACATTGCGCCTATTAGTACTCT

AGTGGGCATGCCTGTTCGAGCGTCATTTCAACCCTTAAGCCTAGCTTAGTATTGGGACTTTACCTTTGGGTAATTCCTCA

AATCCAACGGCGGATCTGTGGTATCCTCTGAGCGTAGTAATTtttATCTCGCTTTTGTTAGGTGCTGCAGCTCCCAGCCG

CTAAACCcccAAATTtttAATGGTTGACCTCGGATCAGGTAGGAATACCCGCTGAACTTAA

>OTU_544

GAAATGCGATAAGTAATGTGAATTGCAGAATTCAGTGAATCATCGAATCTTTGAACGCACATTGCGCCCCGTGGTATTCC

GCGGGGCATGCCTGTTCGAGCGTCATTACACCACTCAAGCCTCGCTTGGTATTGGGCGACGCGGTTAGCCGcgcgcCTCA

AATCTTCCGGCTGGGTCTGAATCGTCCCTCAGCGTTGTGGAAACTATTCGCTAAAGGTGCACGTTTCGGTCACGCCGTTA

AACAAACCCATTCATTAAAGGTTGACCTCGGATCAGGTAGGGATACCCGCTGAACTTAA

>OTU_545

GAAATGCGATAAGTAATGTGAATTGCAGAATTCAGTGAATCATCGAATCTTTGAACGCACATTGCGCCCGCTAGTATTCT

GGCGGGCATGCCTGTTCGAGCGTCATTTCAACCATCAAGCCCCAGGCTTGCGTTGGgggCCTGCGGCTGCCGCAGCCCCT

GAAAAGCAGTGGCGGGCTCGCTGTCATTCCGAGCGTAGTAGTTACATCTCGCTCAAGGCGTGGCGGTGGGCACCTGCCGT

GAAACATTCTTTCTAAGGTTGACCTCGGATCAGGTAGGAATACCCGCTGAACTTAA

>OTU_546

GAAATGCGATAAGTAATGCGAATTGCAGAATTCAGTGAGTCATCGAATCTTTGAACGCACATTGCGCCCCGTGGTATTCC

GCGGGGCATGCCTGTTCGAGCGTCATTTCAACCCTCAAGCTCTGCTTGGTATCGGGCCGCACCGGTAGCGGTGGGCCTCA

GAATCAGTGGCGGTGCCGTCTGGCTCTAAGCGTAGTAATTCTCCTCGCTATAGGTGTCTGGCGGTGTCTGGCCAGCAACC

cccAATTTTCTATGGTTGACCTCGGATCAGGTAGGGATACCCGCTGAACTTAA

>OTU_547

GAAATGCGATAAGTAATGTGAATTGCAGAATTCAGTGAATCATCGAATCTTTGAACGCACATTGCGCCCATTAGTATTCT

AACGGGCATGCCTGTTCGAGCGTCATTTCAACCCTCAAGCCCTGCTTGGTGTTGGGGCACTATTTTCGTATATGCCCTGT

AACGTAGTGGCGGATCTGCTGTCATCCTGAGCGTAGTAATACACGTCGCTTTGGGATTTCAGTGGGCTTCTGCCGTTAAA

CCcccACTTTATAAGGTTGACCTCGGATCAGGTAGGAATACCCGCTGAACTTAA

>OTU_548

AAAATGCGATAAGTAATGTGAATTGCAGAATTCAGTGAATCATCGAATCTTTGAACGCACATTGCGCCCGCCAGTATTCT

GGCGGGCATGCCTGTCTGAGCGTCATTTCAACCCTCGGGACCCCGTTCGCGGGACCCGGCGTTGGGGATCAGCCCTCTCC

GGggggCTGGCCCCGAAATCTAGTGGCGGTCCGCCAGCGATCTCCTCTGCGCAGTAGTTGATACCTCGCAGCTGGATAGC

GGTCGGGCCACGCCGTAAAACCcccAACTTCTCAAGGTTGACCTCAGATCAGGTAGGAATACCCGCTGAACTTAA

>OTU_549

GAATTGCGATAAGTATTGTGAATTGCAGATTTTCGTGAATCATCGAATCTTTGAACGCACATTGCGCCCTCTGGTATTCC

AGAGGGCATGCCTGTTTGAGCGTCATTTCCTTCTCAAACCTTAGGGTTTGGTAGTGAGTGATACTCTTTCTAGGGTTAAC

TTGAAAATGCTGGCCATCTGGCTGTTGCTGGCTGAGGCTTTAGTCCAGTCTGCTGATACTCTGCGTATTAGGTTTTACCA

ACTCGTGGgggCTTGAGCGGACGCTACAAAGACTTTTGCTAAAAGTACAGACAACCTGGCGAACAGTATTCACTAAGTTT

GACCTCAAATCAGGTAGGATTACCCGCTGAACTTAA

>OTU_550

GAAATGCGATAAGTAATGTGAATTGCAGAATTCCGTGAATCATTGAATCTTTGAACGCATATTGCGCCCTCTGGTATTCT

GGggggCATGCCTGTCCGAGCGTCATTACCACCTTCAAGCGCAGCTTGGTTGTTGAGCCAATTGTTCCTTTAATGGACAG

GCTTGAAATGCAGTGGCGATGTCGAGTCTTGATGCCCGAGCGTATGGAGCTCTGTCACACGCTTTAGTGGCTCAGCCGGC

ATTCTGTCGATTGTTTATTTGGGCCTAAaaaCCTAAGATTTAACCTCGGATCAGGTAAGGATACCCGCTGAACTTAA

>OTU_551

GAAATGCGATAAGTAATGTGAATTGCAGAATTCAGTGAATCATCGAATCTTTGAACGCACATTGCGCCCTCTGGTATTCC

GGgggCATGCCTGTTCGAGCGTCATTATAACCACTCAAGCTCTCGCTTGGTATTGGGGTTCGCGGTTTCGCGGCCCCTAA

AATCAGTGGCGGTGCCTGTCGGCTCTACGCGTAGTAATACTTCTCGCTACAGGGTCCCGGTGGATGCTTGCCATCAACCC

CTAATTTTCTATGGTTGACCTCGGATCGGGTAGGGATACCCGCTGAACTTAA

>OTU_552

GAAATGCGATAAGTAGTGTGAATTGCAGAATTCAGTGAATCATCGAATCTTTGAACGCACATTGCGCCCCTTGGTATTCC

ATGGGGCATGCCTGTTCGAGCGTCATTTGTACCTTCAAGCTTTGCTTGGTGTTGGGTGTTTGTCCATTCTCTTGCAGAGG

GACTCGCCTTAAAGTAATTGGCAGCCAGTGTTTTGGTTTTGAAGCGCAGCACAAGTCGCGATTCAACGCTATACGCCAGC

TTCCACAAGCCTTtttCACTTTTGACCTCGGATCAGGTAGGGATACCCGCTGAACTTAA

>OTU_553

GAAATGCGATAAGTAATGTGAATTGCAGAATTCAGTGAATCATCGAATCTTTGAACGCACATTGCGCCCCGTGGTATTCC

GCGGGGCATGCCTGTTCGAGCGTCAGTACAACACTCAAGCTCTGCTTGGTATTGGGCCCTGCCGGCAACGGCAGGCCTTA

AAATCAGTGGCGGCGCCATCTGGCTCTAAGCGTAGTAATACTCCTCGCTACAGAGTCCCGGTGGATGCTTGCCAGCAACC

cccAACTTCTAAGTTTGACCTCGGATCAGGTAGGGATACCCGCTGAACTTAA

>OTU_554

GAAATGCGATAAGTAGTGTGAATTGCAGAATTCAGTGAATCATCGAATCTTTGAACGCACATTGCGCCCTTTGGTATTCC

AAAGGGCATGCCTGTTCGAGCGTCATTTGTACCCTCAAGCTTTGCTTGGTGTTGGGCGTCTTGTCTCTAGCTTTGCTGGA

GACTCGCCTTAAAGTAATTGGCAGCCGGCCTACTGGTTTCGGAGCGCAGCACAAGTCGCACTctctATCAGCAAAGGTCT

AGCATCCATTAAGCCTTtttttCAACTTTTGTACCTCGGATCAGGTATGGGACTACCGCTAACTTAA

>OTU_555

GAAATGCGATAAGTAATGTGAATTGCAGAATTCAGTGAATCATCGAATCTTTGAACGCACATTGCGCCCCGTGGTATTCC

GCGGGGCATGCCTGTTCGAGCGTCATTTCACCACTCGAGTCTGACTCGGTATTGGgggCCGCGATCCGTCGCGCCCCTTG

AAGTCTCCGGCTGGGCGGTCCGTCTCCGAGCGTTGTGGCATCTCGTCTCGCTAGGGAGGTACGGGCGGCGTCGGCCGTTA

AACACCCCATCAAAGGTTGACCTCGGATCAGGTAGGGATACCCGCTGAACTTAA

>OTU_556

GAAATGCGATAAGTAATGTGAATTGCAGAATTCAGTGAATCATCGAATCTTTGAACGCACCTTGCGCCCTTTGGTATTCC

GAAGGGCATGCCTGTTTGAGTGTCATGAAACCTCACCCCACTTGGGTTtttGCCTGAGCGGTGGTGTATTGGGTGTTGCC

TTGCCAAAGGCTCGCCTTAAAACATAAGCACCTTGGATGTAATACGTTTCAATCCTTCTTGGGCTGGCTGGATAACCCCA

CCATATTCATGATCTGCCTCAATCAAGGTAGGGCTACCCGCTGAACTTAA

>OTU_557

GAAATGCGATAAGTAATGTGAATTGCAGAATTCAGTGAATCATCGAATCTTTGAACGCACCTTGCGCTCCTTGGTATTCC

GAGGAGCATGCCTGTTTGAGTGTCATTAATATCTATATCAACCccccTTCTCTTGTGTAGGAGTGGTTTGGATTTGGggg

TTTGCTGGCCTCTTAAaaaGGTTCAGCTCTCCTGAAATGCATTAGCAGAACAATCCTTGTTCATTGGCGTGATAACTATC

TATGCTATTGAATGTGAGGTGCAGTTCAGCTTTCTAACAGTCCTTTGGACAAATTCATCATTAATGTGACCTCAAATCAG

GTAGGACTACCCGCTGAACTTAA

>OTU_558

GAAATGCGATACGTAATGTGAATTGCAGAATTCCGTGAATCATCGAATCTTTGAACGCACATTGCGCCCCTTGGTATTCC

AGGgggCATGCCTGTTTGAGCGTCATTTCCTTCTCAAATACTTGTATTTGGTTGTGAGTGACACTCAGTCTATGCATTGA

GTTAACTTGAAATTGTTGGCCGTAGCGGTTGTTGCAGCTTTAGTTCATTTCTGTGATGGTATGTTTTCTTTACTATTAAA

CAGGATGCTGAGCATGTCGTATTAGGTTTTACCAACTCCGGCAGACTCTGGTGTCTTGGAAGAGCGTGCTTGCAGTGTGA

CCGCAAACTGTGCTTCTGTGGCAAACAGTACTCTTTAAGTTTGACCTCAAATCAGGTAGGAATACCCGCTGAACTTAA

>OTU_559

GAATTGCGATAAGTAGTGTGAATTGCAAATTCTGTGAATCATCGAATCTTTGAACGCATATTGCGCTctctGGTATTCCG

GAGAGCATGCCTGTTTGAGGGTAATAAaaaGAAATCTTTGACTTTCGAGTCAAGGGAAATGGGTATTCCGCATTtttAGC

GGTAGCCTGAAATGTATGGTTGTCTCATCTATTCGAGATGCGTAATAATATTATTTCGTAAAATCTTTGATATGACGAGC

TACCAAGTCTTGACGTGTACGCTTCATTGTTGTATGCGATAATACAAATATTtttGACATTACCTCAAATCAGGTAGGAA

TACCCGCTGAACTTAA

>OTU_560

GAAATGCGATAAGTAGTGTGAATTGCAGAATTCAGTGAATCATCGAATTtttGAACGCATATTGCGCCTTCCGGTATTCC

GGAAGGCATGCCTGTTCGAGCGTCATAATGACCCATCAATCATGAATTTATTCATGCTTGGCATTGGGCAAGGATAGAGC

TCGGCCCATTCCTGTCTGAAATTCAATGGCGAAGTCATATGTTCCATTCAGTGTAGTAATCAACGTTATGTTTCACTGCT

GAACATGATCGGCTTCTGTTACAAATTGTTTCCcccATTCTAAACGTTTGACCTCGGATCAGGTAGGTATACCCGCTGAA

CTTAA

>OTU_561

GAAATGCGATAATTAATGTGAATTGCAGAATTCAGTGAATCATCGAGTCTTTGAACGCACATTGCGCCcccTGGTATTCC

GGggggCATGCCTGTCCGAGCGTCATTGCTGCCCTCAAGCACGGCTTGtgtgtTGGGCTTCCGTCCCTGGCAACGGGGAC

GGGCCCAAAAGGCAGTGGCGGCACCATGTCTGGTCCTCGAGCGTATGGGGCTTTGTCACCCGCTCCCGTAGGTCCAGCTG

GCAGCTAGCCTCGCAACCAATCTTtttAACCAGGTTGACCTCGGATCAGGTAGGGATACCCGCTGAACTTAA

>OTU_562

GAAATGCGATACGTAGTGTGAATTGCAGAATTCCGTGAACCATCGAATCTTTGAACGCATATTGCGCCTGAGGCCTCGGC

CAAGGGCATGTCTGCCTCAGCGTCGGCATAACCCCATCACCCACAAACCTTGTGAGTGGACTTGGCAGTCCCAATCCGGG

TCTGCCGAATTGCAGCAGTTGCTATCAGGACCCGCCTTTGGCGTGGCCATTCAGTTTGGTAGGTAACCCCTGGTTGCATG

CCACTGTTGGCCATTGTGGCCTGTGTAGCATACTGGCAGGAAACCAAGGGCCcccGAGCCCTTTCTCATTTCGACCTGAG

GTCAGGCAAGATTACCCGCTGAACTTAA

>OTU_563

GAATTGCGATAAGTAATGTGAATTGCAGAATTCAGTGAATCATCGAATCTTTGAACGCATCTTGCGCTCTTTGGTATTCC

GAAGAGCATGCCTGTTTGAGTGTCATGAAACTCTCACCTGCAGCACTCTTTTATTAGAGGGCTGGAGCGTGGACGTGAGT

GCTGCTGGTGCCCTCGCTGCATCGGCTCACTTGAAATTTATTAGCTGAATCCTCTAGTGTTGGTTCTACTCGACGTGATA

AGATCTCCGTCGAGGACAGTGCTCCTTGTGGGCGTTGGCCACCGCTAGCTGTTGATACGCTCCTAATTAGCGCAGACTTC

GAGTGCTGGCAACTTTTGACAACTTGGCCTCAAATCAGGTAGGACTACCCACTGAACTTAA

>OTU_564

GAAATGCGATAAGTAATGCGAATTGCAGAATTCAGTGAGTCATCGAATCTTTGAACGCATATTGCGCCCTTTGGTATTCC

GAAGGGCATGCCTGTTCGAGCGTCATTATCAACCATCAAGCCTGGCTTGTCGTTGGACTTtttttGTTGCTCATGCGACA

GGTCCGAAAGATAATGAACGGTGTCATGGTGGCCCTAAATGCAACGAGCTTtttACAGCACGCATCTAGTGGTTAGCAGG

CCCGGTCTTAACCCTTAATTtttCAAGGTTGACCTCGGATCAGGTAGGAATACCCGCTGAACTTAA

>OTU_565

GAAATGCGATACGTAGTGTGAATTGCAGAATTCCGTGAATCATCGAATCTTTGAACGCATATTGCGGTCGAGGCTTCGGC

TGAGACCATGTCTGCCTCAGCGTCGGTGTGAAACCTCACCCTCCAAACATTGGGGAGTGGAACTGGTCTTCCCAGCGGTG

CTTGCGCCGCTGGGCTGGCTGAAATGCAGAGGTACGCGCTTGGACCTGTATGGCAACAGCAAGGTAGGTAGCTCGTCTAC

TCCAGCTGATGCTTTGGGCTCTTGCGCTGACCCGCAGGAAATTCTCACCTTTTCGACCTGAGATCAGGCAAGGCTACCCG

CTGAACTTAA

>OTU_566

GAAATGCGATAAGTAATGTGAATTGCAGAATTCAGTGAATCATCGAATCTTTGAACGCACATTGCGCCCATTAGTATTCT

AGTGGGCATGCCTGTTCGAGCGTCATTTCAACCCTCAAGCCCTAGTTGCTTGGTGTTGGGGCCCTGGGAAGCTTATACTC

TCCAGGCCTCGAAAGACAGTGGCGGGCGCCCCGCGACCCCGAGCGTAGTAATTTCGTCTCGTTTAGGGAGTCCGGTGGTG

CTTGGCCGTTAAACCcccAATCTTTCTGAATTTTGACCTCGGATCAGGTAGGAATACCCGCTGAACTTAA

>OTU_567

GAAATGCGATAAGTAGTGTGAATTGCAGAATTCAGTGAATCATCGAATCTTTGAACGCACATTGCGCCCTTTGGTATTCC

TTAGGGCATGCCTGTTCGAGCGTCATCTAACCcccTCAAGCACTGCTTGGTGTTGGGCGCTTTGTCCCGCCTCGGGCGCG

GACTCGCCTCAAAAGCATTGGCGGCCTGTGTATTGGCTTCGAGCGCAGCAGACTCGCGCCTCGCGAGCCTCTGGCACGGG

CGTCCATCAAGACGCACCCCAAATTTTGACCTCGGATCAGGTAGGGATACCCGCTGAACTTAA

>OTU_568

GAAATGCGATAAGTAATGTGAATTGCAGAATTCAGTGAATCATCGAATCTTTGAACGCACATTGCGCCCCTTGGTATTCC

GAGGGGCATGCCTGTTCGAGCGTCATTACACCACTCAAGCATCGCTTGGTATTGGGCATCGTCGCCGTAAAGGCGGGCGT

GCCTCGAAGACCTCGGCGGGGTTTCTCCAACTTCGGGCGTAGTAGAGTTAAATCAAAACGTCTTATAAGCTTGGTGAGAT

CTCATTGCCGTTAAACCTTTTAtatatTTCAGGTTGACCTCGGATCAGGTAGGGATACCCGCTGAACTTAA

>OTU_569

GAAATGCGATAAGTAATGTGAATTGCAGAATTCAGTGAATCATCGAATCTTTGAACGCACATTGCGCCCCTTGGTATTCC

GGgggCATGCCTGTTCGAGCGTCATTTCAACCCTCAAGCTTAGCTTGGTATTGAGTCTATGTCAGTAATGGCAGGCTCTA

AATCAGTGCGGCGTCCGCTGGGTCCTGAACGTAGTAATATCtctcGTTACAGGTTCTCGGTGTTGCTTCTGCCAAAACCC

AAATTtttCTATTGGTTGACCTCGGATCAGGTAGGGATACCCGCTGAACTTAA

>OTU_570

GAAATGCGATAAGTAATGTGAATTGCAGAATTCCGTGAATCATCGAATCTTTGAACGCATATTGCGCCCTCTGGTATTCC

AGAGGGCATGCCTGTTCGAGCGTCATTTCAAATCCTCAAGCCCGGCTTGTGTAATGGGCTTTAGCGCCTGCTTTAGGGCA

GGTGAGCCTGAAAGATAGTGGCAGTGTAGTGCGAGCAGCTACCGTGTATGGGGACTTTTGTCAcacacTGGCAGCGCGTA

CCCACGCTTGTCTCATCTTCTTCTGTGGTTTGACCTCGGATCAGGTAGGGATACCCGCTGAACTTAA

>OTU_571

GAAATGCGATAAGTAATGTGAATTGCAGAATTCAGTGAATCATCGAATCTTTGAACGCATCTTGCGCTCCTTGGTTAATC

CAAGGAGCATGCCTGTTTGAGTGTCATTAATGTCTCAACCACACCGTGAGGTGTGGCTTGGATTTGGgggTTACAGGCTT

AGTCTGCTCCCCTGAAATGCATAGTGGTAACGACCTCTCCATTAAGATGTGATACATATCTGCATCTAGTTGAGGGTTTT

GCTGCTATACAAGAGTTGGATTTGATAAACTTGACCTCAAATCAGGTAGGACCACCCGCTGAACTTAA

>OTU_572

GAAATGCGATAAGTAATGCGAATTGCAGAATTCAGTGAATCATCGAATCTTTGAACGCACATTGCGCCCGCTAGTATTCT

GGCGGGCATGCCTGTCTGAGCGTCATTTCAACCCTCGCCcccGGCTTTTGCTGGGAGCGGTGTTGGGGATCGGCCGCCCG

TCACTGGGAGGCCGGCCCCGAAATAGAGTGGCGACCACGCCGTGTGCTCCTCTGCGTAGTAGTAAATCACCTCGCAGGCG

GACAGCGGTGCGGCCTGCCGTAAAACCcccAACTCTTTCTTTGGTTGACCTCAGATCAGGTAGGAATACCCGCTGAACTT

AA

>OTU_573

GAAATGCGATAAGTAATGTGAATTGCAGAATTCAGTGAATCATCGAATCTTTGAACGCACCTTGCGCCCTCTGGTATTCC

GGAGGGCATGCCTGTTTGAGTGTCATGAACACCTCACCcccTCGGGTTTCCGATCGGGTGGATTTGGGCGTTGCCAGTCA

CATGGCTCGCCTTAAATGCATTAGCGAATGTTGACGTAATAAGTTTCGTCTGACCATCCGCTGCGAATCTCCTTCGGGAT

GTCCCTCGGACACTTCATGATCTGACCTCAAATCAGGTAGGACTACCCGCTGAACTTAA

>OTU_574

GAAATGCGATAAGTAATGTGAATTGCAGAATTCAGTGAATCATCGAATCTTTGAACGCACATTGCGCCCGCTAGTATTCT

GGCGGGCATGCCTGTTCGAGCGTCATTTCAACCATCAAGCCCTAGGCTTGCGTTGGAGCCCTGCGGCCGCCGCAGCCTCC

TAAAACCAGTGGCGGGCTCGCTATCACACCGAGTGCAGTAGTTTACTCTTCGCTCAGGGCGTGTGGCGGGTGCTGGCCGT

AAAACCcccTACTTCTTAAGGTTGACCTCGGATCAGGTAGGAATACCCGCTGAACTTAA

>OTU_575

GAAATGCGATACCTGGTGTGAATTGCAGAATCCCGCGAACCATCGAGTCTTTGAACGCAAGTTGCGCCCGAGGCCACTCG

GCCGAGGGCACGCCTGCCTGGGCGTCACGCCAAAACACGCTCCCAACCACCCTCAACGGGAATCGGGATGCGGCATCTGG

TCCCTCGTCtctcAAGGGACGGTGGACCGAAGATTGGGCTGCCGGCGTACCGCGCCGGACACAGCGCATGGTGGGCGTCC

TCGCTTTATCAATGCAGTGCATCCGGCgcgcAGCTGGCATTATGGCCTTTGAACGACCCAACAAACGAAGCGCACGTCGC

TTCGACCGCGACCCCAGGTCAGGCGGGACTACCCGCTGAGTTTAA

>OTU_576

GAAATGCGATAGCTAGTGTGAATTGCAGATTTACGTGAATCATCGAGTCTTTGAACGCACATTGCGCCCCTCGGTTCTCC

GGggggCATGTCTGTCCGAGCGTCGTTGCAACTCTCAAGCTTGCTTGGTCATGGGGATCGTGCCGCCCGGCGGTGCGTCC

CTCAAACGCAACTCGACTGCCTTGTCAGCGGAGCGCAGTAGAATGCCGCCCCGTGAGGTGCCACCCGGGCTCTTCGGAGA

CTACCATGTTGACCTCGGATCAGATAGGGATACCCGCTGAACTTAA

>OTU_577

GAAATGCGATAAGTAATGTGAATTGCAGAATTCAGTGAATCATCGAATCTTTGAACGCACATTGCGCCCGCCGGCACTCC

GGCGGGCATGCCTGTCCGAGCGTCATTTCAACCCTCAGGGCCcccTTTCGGgggggACCTGGTGCTGGGGATCAGCGGCC

CTCGGGCCCCTGTCCcccAAATACAGTGGCGGTCGCGCCGCAGCCTCCCCTGCGTAGTAGCACACCTCGCACCGGAGAGC

GGTTCGACCACGCCGTAAAACCcccAACTTTCCAAGGTTGACCTCGGATCAGGTAGGAATACCCGCTGAACTTAA

>OTU_578

GAAATGCGATAAGTAATGTGAATTGCAGAATTCCGTGAATCATCGAATCTTTGAACGCATATTGCGCCCTCTGGTATTCC

GGAGGGCATGCCTGTCCGAGCGTCATTACTATCTTCAAGCACGGCTTGGTTGTTGAGCCAATTGTCCTTTTAATGGACAG

GCTTAAAATTCAGTGGCGATGCGGAGTCCTGGTGCCCGAGCGTATGGAGCTCTGTCACACGCTTTAGTGGTCCAGCCGGC

ATTTTCGTCGATTAAACCTTAAGAAaaaCCTAAGATTTGACCTCGGATCAGGTAAGGATACCCGCTGAACTTAA

>OTU_579

CCGAGCCGCCGCCGTCGCCAGAGGCGCTGGCCAGGCGGAATACGCCAGATTTTGACTTCCACATGACGGGCAGCAGCCCG

GGATGGAAGCCTCTGCGGATCTACCGCGAGGGCGATCATACCTACATTCAGTTTCCGCCCGGTGGGATCAACAGCGCCCC

TCGTCTGGTTGTGCTGTCACCCTCCACCACCGCAACCCAGCCGTACCGAACGGTCGGTGACTCTTACGTCGTCGATCTAC

CCGTCACTGACGCCCTGTTGATCGGTAACGACCCCGGCTCTCCTACAATTC

>OTU_580

GAAATGCGATAAGTAATGTGAATTGCAGGATTCAGTGAATCATCGAATCTTTGAACGCACATTGCGCCCTCTGGTATTCC

GGggggCATGCCTGTTCGAGCGTCATTATGACCAATCACGCAAGTGGTGTTGGGGCTTGCCGTCGGCATCCCTTAAAATT

AGTGGCAGTGCTATAGGCTCTCAGCGCAGTAATTATTTCGCTCTTGAGTCCTAGACCCACCCGCCAAAACCTCAACTTTC

TTAAGGTTGACCTCGGATCAAGTAGGGATACCCGCTGAACTTAA

>OTU_581

GAAATGCGATAAGTAATGTGAATTGCAGAATTCAGTGAATCATCGAATCTTTGAACGCACATTGCGCCTAGTGGCATTCC

GCTAGGCATGCCTGTTCGAGCGTCATTTCAACCCTCAAGCCTAGCTTGGTATTGGGTGTCACCGGCATGGTGCGCCTTAA

AATCAGTGGTGGTGCCAGCTGGCTTCAAGCGTAGTAATACTCCTCGCTCTGAATGTCCAGGTGGTCGCTTGCCAAAACCC

CATACTTTCTCAAGGTTGACCTCAGATCAGGTAGGGATACCCGCTGAACTTAA

>OTU_582

GAATTGCGATAAGTAATGTGAATTGCAGAATTCAGTGAATCATCGAATTtttGAACGCACCTTGCGCCTTTGGGTATTCT

CAAAGGCATGCCTGTTTGAGTGTCATAACAATCTCATCtctcAACTTttttGTAAAAGAAGGTTGCTTGGAGATGGTGAT

GGGCGCTTGCCATGCTGTACAAGTCTGGCTCGCCTTAAAGACATCAGTAGCTCTTATGGAGTCCGGTCTGACTATGtgtg

ATAATTTGATCGCATAGGATGTGCTTGCACAACCGGTCTTGTTGTTAAGAGTCTGCTTCTAACCTGGAATGGTATGAGAG

TAACTTTCATCCATCGCAGCTTTATTTGTCTGACCTCAAATCAGGTAGGACTACCCGCTGAACTTAA

>OTU_583

GAAATGCGATAAGTAGTGTGAATTGCAGAATTCAGTGAATCATCGAATCTTTGAACGCACATTGCGCCCCTTGGTATTCC

ATGGGGCATGCTTGTTCGAGCGTCATTTGGACTCTCAAGCTTTGCTTGGTGTTGGGTGCTTGTCTTATTCtctctGATTT

GACTCACCTTAAATGTATTGGCAGCCTATGTATTGGACTTATGCGCAGCACATTTTGCGAGTAAGCCTAATTACacacGC

ATCCATTAAGCAATTTAACACGTTTGACCTCGGATCAAGTAGGGATACCCGCTGAACTTAA

>OTU_584

GAAATGCGATAAGTAATGTGAATTGCAGAATTCAGTGAATCATCGAATCTTTGAACGCACATTGCGCCCATTAGTATTCT

AGTGGGCATGCCTGTTCGAGCGTCATTTCAACCCTTAAGCCTAGCTTAGTGTTGGGAGACTGCCTAATACGCAGCTCCTC

AAAACCAGTGGCGGAGTCTGTTCGTGCTCTGAGCGTAGTAATTCTTTATCTCGCTTCTGCAAGCCGATTAGACAACAGCC

ATAAACCGCACCCTTCGGgggCACTTtttAATGGTTGACCTCGGATCAGGTAGGAATACCCGCTGAACTTAA

>OTU_585

GAAATGCGATAAGTAATGTGAATTGCAGAATTCAGTGAATCATCGAATCTTTGAACGCACATTGCGCCCCTTGGTATTCC

GGggggCATGCCTGTTCGAGCGTCATTTATACCAATCTAGCCTGGCTAGGTGTTGGGCTTCGCCGGTTGGCGGGCCTTAA

AACCAGTGGCTGTGCTCTTAGGCTCTACGCGTAGTAATTTTCTCGCTATAGGGTCCTGGGAGATGCTTGCCAGCAACCCC

AAATTtttCTAGGTTGACCTCGGATCAGGTAGGGATACCCGCTGAACTTAA

>OTU_586

GAAATGCGATAAGTAATGTGAATTGCAGAATTCAGTGAATCATCGAGTCTTTGAACGCACATTGCGCCcccTGGTATTCC

GGggggCATGCCTGTCCGAGCGTCATTGCTGCCCTCAAGCACGGCTTGtgtgtTGGGCCCCGTCCCCTCCcccGGGGACG

GGCCCGAAAGGCAGCGGCGGCACCGCGTCCGATCCTCGAGCGTATGGGGCTTTGTCACCCGCTCTGTAGGCCCGGCCGGC

GCCAGCCGACACCCAACTTTTATTTCTAAGGTTGACCTCGGATCAGGTAGGGATACCCGCTGAACTTAA

>OTU_587

GAAATGCGATAAGTAATGCGAATTGCAGAATTCAGTGAGTCATCGAATCTTTGAACGCACATTGCGCCCTGTGGTATTCC

GCAGGGCATGCCTGTTCGAGCGTCATTTCAACCCTCAAGCTCTGCTTGGTGTTGGGCCCCGCCCGCTCGCGCCGGCCCTA

AAGACAGTGGCGGCAGCGTCTGGCTCCAAGCGTAGTACAATCCTCGCTCTGGTGCTAGGCGGTGGCCTGCCAGAACCccc

cTTCTATGGTTGACCTCGGATCAGGTAGGGATACCCGCTGAACTTAA

>OTU_588

GAAATGCGATACGTAGTGTGAATTGCAGAATTCCGTGAATCATCAAATCTTTGAACGCAAATTGCGCCCAAGGCTTCGGC

TTAGGGCATGTTTGCCTCAGCGTCGGGTTAATCTCACAAACCCTCTCCCCAAGCGGAGCAGAGGGCTAGTGGATCTGGCT

GTTCCAGAACTTGGATGCATTCACTTGTGTCCGTTCAAAGGTCTGGATCAGCTGAAGCACAGAGGCTAGCTCACGACCCG

CTAAGGGCCGCAACTGGGTAGGCAGCTCTCGCTCGCTATTTCTAGTTGTTGGCTTGGTGTCATGGGCTTGGCCCTCAAAC

AGGAAACCCTTttttCTCGACCTGAGCTCAAGCAAGAACACCCGCTGAACTTAA

>OTU_589

GAAATGCGATAACTAATGTGAATTGCAGAATTCAGTGAATCATCGAGTCTTTGAACGCACATTGCGCCcccTGGTATTCC

GGggggCATGCCTGTCCGAGCGTCATTGCTGCCCTCAAGCACGGCTTGtgtgtTGGGCTCCGTCCccccGGGGACGGGTC

CGAAAGGCAGCGGCGGCACCGAGTCCGGTCCTCGAGCGTATGGGGCTTTGTCACCCGCTCTGTAGGCCCGGCCGGCGCCA

GCCGACAACCAATCATCCTTttttCAGGTTGACCTCGGATCAGGTAGGGATACCCGCTGAACTTAA

>OTU_590

GAAATGCGATAAGTAGTGTGAATTGCAGAATTCAGTGAATCATCGAATCTTTGAACGCACATTGCGCCCCTTGGTATTCC

GAGGGGCATGCCTGTTCGAGCGTCATTTGTACCTCAAGCTCTGCTTGGTGTTGGGTGTTTGTCTAGCCTCTGCGGCTAGA

CTCGCCTTGAATCTATTGGCAGCTAGAGTATTGGCTTTGGAGCGCAGCACATTTTGCGTCCCTGGCTGATACTATAGCAC

CCATGAAGCTACCATCACGTTTGACCTCGGATCAGGTAGGGATACCCGCTGAACTTAA

>OTU_591

GAAATGCGATAAGTAGTGTGAATTGCAGAATTCAGTGAATCATCGAATCTTTGAACGCACATTGCGCCCCTCGGTATTCC

GTGGGGCATGCCTGTTCGAGCGTCATTTACACCCTCAAGCTCTGCTTGGTGTTGGGCGTCTGTCCCGCGTTTCCGCGTGG

ACTCGCCTCAAAGTCATTGGCAGCGGTCTTTGCCAGCTTCTTGCGCAGCACAATTGCGCTCACAGGGGCTCTGGCGGATC

GGCGTCCATCAAGCTATTCTATGACTTGACCTCGGATCAGGTAGGGATACCCGCTGAACTTAA

>OTU_592

GAAATGCGATAAGTAGTGTGAATTGCAGAATTCAGTGAATCATCGAATCTTTGAACGCACATTGCGCCCCTTGGTATTCC

ATGGGGCATGCCTGTTCGAGCGTCATTTGTACCTTCAAGCTCTGCTTGGTGTTGGGTGTTTGTCCTTGCCCTAGTGGCGG

GACTCGCCTTAAAGTAATTGGCAGCCAGTGTTTTGGTTTTGAAGCGCAGCACAAGTCGCGATTCAAGTCTATACGCTAGT

TTCCACAAGTCTTTTATCACTTTTGACCTCGGATCAGGTAGGGATACCCGCTGAACTTAA

>OTU_593

GAAATGCGATAAGTAATGTGAATTGCAGAATTCAGTGAATCATCGAATCTTTGAACGCACATTGCGCCCGCCAGTATTCT

GGCGGGCATGCCTGTTCGAGCGTCATTTCAACCATCAAGCCCCGGGCTTGTGTTGGGGACCTGCGGCTGCCGCAGACCCC

GAAAACCAGTGGCGGGCTCGCTGTCACCCCGAGCGTAGTAGTTTACACCTCGCCCAGGGCGCGACGCGGGTTCCCGGCCG

TTAAACCcccACCATCTCAAGGTTGACCTCGGATCAGGTAGGAAGACCCGCTGAACTTAA

>OTU_594

GAAATGCGATAAGTAGTGTGAATTGCAGAATTCAGTGAATCATCGAATCTTTGAACGCACATTGCGCCCCTTGGTATTCC

GAGGGGCATGCCTGTTCGAGCGTCATTTGTTCCTCAAGCTCTGCTTGGTGTTGGGTGTTTGTCCAGCCTCTGTGGCTAGA

CTCGCCTTGAATACATTGGCAGCTAGAGCGTTGGCTTTGGAGCGCAGCACAATTTGCGTCCCTGGCTGAGGCTATAGCAT

CCATGAAGCCATCATTACTTTTGACCTCGGATCAGGTAGGGATACCCGCTGAACTTAA

>OTU_595

GAAATGCGATAAGTAATGTGAATTGCAGAATTCAGTGAATCATCGAATCTTTGAACGCACATTGCGCCCGCCAGTATTCT

GGCGGGCATGCCTGTTCGAGCGTCATTTCAACCATCAAGCCcccGGCTTGTGTTGGGGACCTGCGGCTGCCCGCAGGCCC

TGAAAACCAGTGGCGGGCTCGCTGTCACCCCGAGCGTAGTAGCAATCACCTCGCTCAGGGCGTGCCGCGGGCGCCGGCCG

TTAAAGCTGCCTTCGCGCAACACCCAAGGTTGACCTCGGATCAGGTAGGAAGACCCGCTGAACTTAA

>OTU_596

GAAATGCGATAAGTAGTGTGAATTGCAGAATTCAGTGAATCATCGAATCTTTGAACGCACATTGCGCCCTTTGGTATTCC

TTAGGGCATGCCTGTTCGAGCGTCATCTAAACCCTCAAGCCCTGCTTGGTGTTGGGTGCCTGTCCCGCCcccGTGCGTGG

ACTCACCTCAAATTCATTGGCGGCCCTCGTGCCGGCCCCGAGCGCAGCAGAAACGCGGTCTCGTGGCCCGGCGGAGCGTG

CCcccAGAAGCAACATTCACCGTTTTGACCTCGGATCAGGTAGGGATACCCGCTGAACTTAA

>OTU_597

GAAATGCGATAAGTAGTGTGAATTGCAGAATTCAGTGAATCATCGAATCTTTGAACGCACATTGCGCCCCATGGTATTCC

GTGGGGCATGCCTGTTCGAGCGTCATTTACCcccTCAAGCTCCGCTTGGTGTTGGGCGTTTGTCCCGCTTCGcgcgcgGA

CTCGCCCCAATGGTATAGGCAGCGGTCGTGCCAGCTTCTCGCGCAGCACATTGCGCTTCTCGAGGCACCGGCGGCCCGCG

TACATGAAGCTCCACCCCAGTTTGACCTCGGATCAGGTAGGGATACCCGCTGAACTTAA

>OTU_598

GAAATGCGATAAGTAATGTGAATTGCAGAATTCAGTGAATCATCGAATCTTTGAACGCACATTGCGCCCCTTGGTATTCC

GAGGGGCATGCCTGTTCGAGCGTCATTACAACCCTCAAGCACTGCTTGGTATTGGGCTCCGCTGGTTCCAGCGGGCCTTA

AAATCAGTGGCGGCGCCGTTTGGGCTCTGAGCGTAGTAAATATCCTCGCTACGGAGACCTGACGGCGGTAGTCATCAACC

CCTAACTTTCTAAGTTTGACCTCGGATCAGGTAGGGATACCCGCTGAACTTAA

>OTU_599

GAAATGCGATACGTAATGTGAATTGCAGAATTCCGTGAATCATCGAATCTTTGAACGCACATTGCGCCCCTTGGTATTCC

AGGgggCATGCCTGTTTGAGCGTCATTTCCTTCTCAAACATTCGTGTTTGGTAGTGAGTGATACTCTGTTtttCATTTGG

GTTAACTTGAAATTGCAAGCCTTTTGGGGAACGCGTGTGGGTGAGTTTTAGGCGGAAACGTCTTGCTCTCCTCTTTCCTA

ACCAAATGTCGTATTAGGTTTTACCGACTCCGACAGACGGGACTAGGAGATTGGGTGAGTGATAGCAATATCGAGCTCTG

CCTA

>OTU_600

GAAATGCGATAAGTAATGTGAATTGCAGAATTCAGTGAATCATCGAATCTTTGAACGCACATTGCGCCCCTTGGTATTCC

GAGGGGCATGCCTGTTCGAGCGTCATTACACCACTCAAGCTATGCTTGGTATTGGGCGTCGTCCTTAGTTGGGCgcgcCT

TAAGACTCGCGAGGCAGCTCCGCGTTTAGGCGTAGTAGAATTTATTCGAACGTCTGTCAAAGGAGAGGAACTCTGCCGAC

TGAAACCTTTATTtttCTAGGTTGACCTCGGATCAGGTAGGGATACCCGCTGAACTTAA

>OTU_601

GAAATGCGATAAGTAATGTGAATTGCAGAATTCAGTGAATCATCGAATCTTTGAACGCACATTGCGCCCCGTGGTATTCC

GCGGGGCATGCCTGTTCGAGCGTCATTTCACCACTCGAGTCTGACTCGGTATTGGGTGCCGcgcgTGAGCgcgcgcCTCA

AAGTCTCCGGCTGCGCGGACCCGTCCCCGAGCGTTGTGGCATCACAGTTCTCGCTGGGGAGCGGCGGGCCCGCCCGCGGC

CGTTAAACacacCCATCAAAGGTTGACCTCGGATCAGGTAGGGATACCCGCTGAACTTAA

>OTU_602

GAATTGCGATAAGTATTGTGAATTGCAGATTTTCGTGAATCATCGAATCTTTGAACGCATATTGCGCCCACTGGTATTCC

AGTGGGCATGCCTGTTTGAGCGTCATTTCCTTCTCAAACCTCGGTTTGGTAGTGAGTGATACTCGGTCGTAAGACAAGGT

TAACTTGAAAATGCTGGCCATGGGCGGAGCTTGCGCGGACTGCGGTCTTAGCTAGCTTTTACACTGCGTATTAGGTTTCG

ACCGGATCGTGGAGTATAAGCTGGCGTTTGAGGAACGTATGGACAAACAAGACCTCTCAGGCGAATAGTATTCCTAAAGT

TTGACCTCAAATCAGGTAGGATTACCCGCTGAACTTAA

>OTU_603

GAAATGCGATAAGTAATGTGAATTGCAGAATTCAGTGAATCATCGAATCTTTGAACGCACCTTGCGCTCCTTGGTATTCC

GAGGAGCATGCCTGTTTGAGTGTCATTAAATTCTCAATCTCAACATTCTTGTAATGCTTGCAGATTGGATGTGGgggTTG

TTGTGGGGTTCTTTTACAAGTTCCACTCCCCTGAAATatatTAGCTGGAATGCCTTTGCAAACCAACTATCAGTATGATA

ATTATCTATGCTGGAGTTGATGCAAATAGGGAATCTGCTTCTCAATCGTCTTTCACTAAGACAACTTGAACCCTTTTGAC

CTCAAATCAGGTAGGACTACCCGCTGAACTTAA

>OTU_604

GAAATGCGATAAGTAGTGTGAATTGCAGAATTCAGTGAATCATCGAATCTTTGAACGCACATTGCGCCCCTTGGTATTCC

ATGGGGCATGCTTGTTCGAGCGTCATTTGTACCCTCAAGCTTTGCTTGGTGTTGGGTGTTTGTCCTTTCCTCGCGTTTGG

ACTCGCCTTAAAAGTATTGGCAGCCAGTGTATTGGTTTGAAGCGCAGCACAATTTGCGATTTAAGCTGGTAATCAGTGGC

CTCCATCAAGCCATCTAACACTTTTGACCTCGGATCAAGTAGGGATACCCGCTGAACTTAA

>OTU_605

GAAATGCGATAAGTAGTGTGAATTGCAGAATTCAGTGAATCATCGAATCTTTGAACGCACATTGCGCCCTTCGGTATTCC

GTTGGGCATGCCTGTTCGAGCGTCATTTAAACCCTTCAAGCTCTGCTTGGTGTTGGGCGTTTGTTCCGCCTCCGcgcgTG

GACTCGCCTCAAATTCATTGGCAGCCGGTCAGTTGGCTTTCGTGCGCAGCACATTGCGTAGCGGCTCCAGCTTGCCTCCT

ACCATTAAGCTACTCTACGTCTTTTGACCTCGGATCAGGTAGGGATACCCGCTGAACTTAA

>OTU_606

GAAATGCGATACTTGGTGTGAATTGCAGAATCCCGTGAACCATCGAGTCTTTGAACGCAAGTTGCGCCCCAAGCCGTTAG

GCCGAGGGCACGTCTGCCTGGGTGTCACGCACCGTTGCCcccccACCCCTCGCCTCACACGAGGCGGGGAGATGTGTCGG

gggCAGATGTTGGCCTCCCGTGCGCCGTAGCTCGCGGTTGGCCCAAATACGAGTCCTCGGCGACGGACGCCGTGACGTTC

GGTGGTGGAATAAACCTCGATATCCCGTCGcgcgcACGTCGACGATACAGTGCTCTTTGATCCCGAAGCGTTGTGTACAC

GGCGCAAACATTGCGACCCCAGGTCAGGCGGGATTACCCGCTGAATTTAA

>OTU_607

GAAATGCGATAAGTAATGTGAATTGCAGAATTCAGTGAATCATCGAATCTTTGAACGCACATTGCGCCTCCTGGTATTCC

AGGGAGCATGCCTGTTCGAGCGTCATTTAGCAACCATCGAGCGAGTTTTGCCTCGCTTGGGTCTGGgggATGGTTGAACC

TCGGTGCACCCACCcccGAAATACAACGGCGGACAACCCACCCGTGGAACTGCAGCGTAGTAAGTCATTCTTTCGCGAGT

GGCCACGGAGGGACTGTCCGGCCGGTGAAACCccccAAATCCAATGGTTGACCTCGGATCAGGTAGGGATACCCGCTGAA

CTTAA

>OTU_608

GAAATGCGATAAGTAGTGTGAATTGCAGAATTCAGTGAATCATCGAATCTTTGAACGCACATTGCACCTCTCGGTATTCC

GGGAGGTATGCCTGTTCGAGCGTCATTTGTACCCTCAAGCTCTGCTTGGTGTTGGGTGTTTGTTCCGCCTTTGCGTGTGA

ACTCGCCTTAAAACAATTGGCAGCCGGCGTACTTGCTTCGGAGCGCAGCACATTTTGCGTTCAGATCAATGTACTCTAGC

ATCCAAGAAGCCTTATTTAACGCTCTTGACCTCGGATCAGGTAGGGATACCCGCTGAACTTAA

>OTU_609

GAAATGCGATAAGTAATGCGAATTGCAGAATTCAGTGAATCATCGAATCTTTGAACGCACATTGCGCCCGCTAGTATTCT

GGCGGGCATGCCTGTCTGAGCGTCATTTCAACCCTCGCGCCCGGCTCCTGTCGGGTGCGGTGTTGGGGATCGGCCTGCCG

CCTGGCGGTGGGCCGTCCCTGAAATGGAGTGGCGACCACGCCGTAGCCTCCTCTGCGTAGTAATCCAATTCTCGCAGGCG

GACAGCGGTGCGGTCATGCCGTAAAACACCCCAAACTTTACCAAGGTTGACCTCAGATCAGGTAGGAATACCCGCTGAAC

TTAA

>OTU_610

GAAATGCGATAAGTAATGTGAATTGCAGAATTCAGTGAATCATCGAATCTTTGAACGCACATTGCGCCCCTTGGTATTCC

GGggggCATGCCTGTTCGAGCGTCATTATAACCCTCAAGCCTAGCTTGGTGTTGGAGCCTGCCTCTGGGCAGCTCTTAAA

ATCAGTGGCGGTGCCGTCTGGCTCTAAGCGTAGTAATTCTTCTCGCTACAAGGTCCCGGCGGTTGCTTGCCAACAACCCC

AAATTTTCTATGGTTGACCTCGGATCAGGTAGGGATACCCGCTGAACTTAA

>OTU_611

GAAATGCGATACGTAATGTGAATTGCAGAATTCCGTGAATCATCGAATCTTTGAACGCACCTTGCGCCTCTTGGTATTCC

GAGGGGCATGCCTGTTTGAGTGTCATTGAATCCTCAAAAGAAAAGCTTAACTGTTTCTCTTTTGGAACTGGAGCGTGCTG

GCCTTTCGTTAGGAAGCCCAGCTCCTCTGAAATACATTAGCGATGTGACATCCGCATGGCACCACGCTTCCCTGGCATTG

ATAGTATCTTCATGCCTGAGCGTGTTTGTGCAGCTGTGGAAAGTTTCGCTCGCTCATAACCTGATGGGTGTCAGCCCATC

CTTGACCAATTTAACCTCAAATCAGGTAGGATTACCCGCTGAACTTAA

>OTU_612

GAAATGCGATAAGTAATGTGAATTGCAGAATTCAGTGAATCATCGAATCTTTGAACGCAACTTGCGCCTTTTGGTATTCC

GAAAGGCATGCCTGTTTGAGTGTCATATAAACCTCATCAATACTGTTTTCTGAACTAGTATCTGATGCTTTGGGCGTCTG

CCATTCGTGGCTCGCCTTAAATGTCTAAGTGGAATGCGTAATAAGTCTTCGCAACCCTCCACTCTAATACACTTTATATT

CTGACCTCAAATCAGGTAGGACTACCCGCTGAACTTAA

>OTU_613

GAAATGCGATAACTAATGTGAATTGCAGAATTCAGTGAATCATCGAGTCTTTGAACGCACATTGCGCCcccTGGTATTCC

GGggggCATGCCTGTCCGAGCGTCATTTCTCCCCTCCAGCCCCGCTGGTTGTTGGGCCGCGCCcccccGGgggCGGGCCT

CGAgagaAACGGCGGCACGTCCGGTCCTCGAGCGTATGGGGCTCTGTCACCCGCTCTATGGGCCGGCCGGGGCTTGGCCT

CGACCCCAACTCTTCTCAGATTGACCTCGGATCAGGTAGGGATACCCGGCTGAACTTA

>OTU_614

GAAATGCGATAAGTAATGTGAATTGCAGAATTCAGTGAATCATCGAATCTTTGAACGCACCTTGCGCCCTTTGGTATTCC

GAAGGGCATGCCTGTTTGAGTGTCATATAAACCTCACCTCCACTGTTtttCATTAGACATGTGGATCGGTGCTTTGGGTG

TCTGCCGTTTACTCTGGCTCGCCTTAAATGTCTAAGTGGAATGCGTCATAAGTTCTTTCGCAATCCTCCACTCTAATCTA

CAACCTTTATATTCTGACCTCAAATCAGGTAGGACTACCCGCTGAACTTAA

>OTU_615

GAAATGCGATAAGTAATGTGAATTGCAGAATTCAGTGAATCATCGAATCTTTGAACGCACCTTGCGCCCTTTGGTATTCC

GAGGGGCATGCCTGTTTGAGTGTCATTAAATACCATCAACCCTCTTTTGACTTCGGTCTCGAGAGTGGCTTGGAAGTGGA

GGTCTGCTGGAGCCTAACGGAGCCAGCTCCTCTTAAATGTATTAGCGGATTTCCCTTGCGGGATCGCGTCTCCGATGTGA

TAATTTCTACGTCGTTGACCATCTCGGGGCTGACCTAGTCAGTTTCAATAGGAGTCTGCTTCTAACCGTCTCTTGACCGA

GACTAGCGACTTGTGCGCTAACTTTTGACTTGACCTCAAATCAGGTAGGACTACCCGCTGAACTTAA

>OTU_616

GAAATGCGATAAGTAGTGTGAATTGCAGAATTCAGTGAATCATCGAATCTTTGAACGCACATTGCGCCCTTTGGTATTCC

TTAGGGCATGCCTGTTCGAGCGTCATTTCAAAATTCAAGCTCAGCTTGGTGATGGGCGTCTGTCCCGCCTCCGcgcgcgG

ACTCGCCTCAAAAGTAGTTGGCAGCTCCCTTATCGGCACTGAACGCAGCAAATTTGCGGGACGCACCGAAGAAAGGGGCT

TACCAGTAAGCAAACCACCCCAGATTGACCTCGGATCAGGTAGGGATACCCGCTGAACTTAA

>OTU_617

GAAATGCGATAAGTAATGTGAATTGCAGAATTCAGTGAATCATCGAATCTTTGAACGCACCTTGCGCTCCTTGGTATTCC

GAGGAGCATGCCTGTTTGAGTGTCATTAATatatCAACCTTCTCAAGTTTTGCTTGTTAAGTGTTGGATGTGGgggTCTT

TTGCTGGTCtctctTTTCTGAGGTCGGCTCCCCTGAAATGCATTAGCGGAACAATTTGTTGACCGTTCATTGGTGTGATA

ACTATCTACGCTATTGACGTGAAGCAGGTTCAGCTTCTAACAGTCCATTGACTTGGACAAATCTTCATTAATGTGACCTC

AAATCAGGTAGGACTACCCGCTGAACTTAA

>OTU_618

GAAATGCGATAAGTAATATGAATTGCAGATTTTCGTGAATCATCGAATCTTTGAACGCACATTGCGCCCTCTGGTATTCC

AGAGGGCATGCCTGTTTGAGCGTCATTTCtctctcAAACCTTCGGGTTTGGTATTGAGTGATACTCTTAGTTAAACTGGG

CGTTTGCTTGAAATGTATTGGCATGAGTGGTACTGGATAGTGCTATATGACTTTCAATGTATTAGGTTTATCCAACTCGT

TGAATAGTTGAATGGTATATTTCCTAGTATTCTAGGCTCGGCCTTACAATGCAACAAACAAGTTTGACCTCAAATCAGGT

AGGAGTACCCGCTGAACTTAA

>OTU_619

GAAATGCGATAAGTAATGTGAATTGCAGAATTCAGTAAATCATTAAATCTTTGAACGCATATTGCGCCTGCCAGCATTCT

AGCAGGCATACCTGTTTGAGCGTTATTTTAACCCTTAAGCTCCGCTTAGTGTTAGGGCTCTATAGCTAATGTAGGCCCTC

AAAGGTAGTAGCAGACCCTCCTAGAGCCTCCTTTGCATAGTAACTTTACGTCTCACACTAGGATCTAGAGGAACTCTTGC

TATAAAACCccccAATTTTCCAAAGGTTGACCTCAGATCAAGTAGGAATACCTGCTAAACTTAA

>OTU_620

GAAATGCGATAAGTAATGTGAATTGCAGAATTTAGTGAATCATCGAATCTTTGAACGCACATTGCGCCTTCTGGTATTCC

AGGAGGCATGCCTGTTCGAGCGTCATTACAACCCTCAAGCTCTGCTTGGTATTGGGTGTCACCTTGCCTGGTGCATCTTA

AAATCAGTGGTGGTGCCATTTGGCTTCAAGCATAGTAATACTTCTTGCTTTGGAGGTTGGGTgtgtgCTTGCCAGTAACC

CCTAATTATCAAAGGTTGACCTCGGATCAGGTAGGGATACCCGCTGAACTTAA

>OTU_621

GAAATGCGATACGTAATGTGAATTGCAGAATTCAGTGAATCATCGAATCTTTGAACGCACCTTGCGCTCCTTGGTATTCC

GAGGAGCATGCCTGTTTGAGTGTCATGAAATCTTCAACCCACCTCTTTCTTAGTGAATCTGGTGGTGCTTGGTTTCTGAG

CGCTGCTCTGCTTCGGCTTAGCTCGTTCGTAATGCATTAGCATCCGCAACCGAACTTCGGATTGACTTTGGGCGTAAATA

GACTATTCGCTGAGGATTCTAGTTTTACTAGAGCCGAGTTGGGTTAAAAGGAAGCTCCTAATCCTAAAAGTCTATTtttt

tGTTAGATCTCAAATCAGGTAGGACTACCCGCTGAACTTAA

>OTU_622

GAAATGCGATAAGTAGTGTGAATTGCAGAATTCAGTGAATCATCGAATCTTTGAACGCACATTGCGCCCCTTGGTATTCC

ATGGGGCATGCCTGTTCGAGCGTCATTTGTACCCTCAAGCTTTGCTTGGTGTTGGGCGTCTTGTCCCGCGTTGTCGCGTG

GACTCGCCTTAAAGCGATTGGCAGCCGGCATATTTGGCCTTGGAGCGCAGCACATTTTGCGCCTCTTGCCTTGAATGTTG

GCGTCCATTAAGCCTATTACATTTTGCATTTTGACCTCGGATCAGGTAGGGATACCCGCTGAACTTAA

>OTU_623

GAAATGCGATAAGTAATGTGAATTGCAGAATTCAGTGAATCATCGAATCTTTGAACGCACATTGCGCCcccTGGTATTCC

GGggggCACACCTGTTCGAGCGCCATTTCAACCCTCAAGCCTAGCTTGGTGATGGGCCACGTCCGCAGGGGCGAGCCTGA

AACTCGTAGGGCGCTGTCCGCCGGCTCTGAGCGTAGCAAGAGAAATCCCTCGCTCTGGATAACCGTCGGGTGGCCGCCTC

GAAAACCCACGAAATATTACAAGGTTGGCCTCGGATCAGGTGGGGATACCCGCTGAACTTAA

>OTU_624

GAAATGCGATAAGTAATGTGAATTGCAGAATTCAGTGAATCATCGAATCTTTGAACGCACATTGCGCCTATTAGTATTCT

AGTAGGCATGCCTGTTCGAGCGTCATTTCAACCCTTAAGCCTAGCTTAGTGTTGGGAGTCCACTGTATTGTGGTTCCTGA

AAGACAACGGCGGATTCGCAGTATCCTCTGAGCGTAGTAATTtttCTAAAATCTCGCTTTGGTTAGGTGCTGTGGTTCCT

GCCGCTAAACCcccAAATTtttAATGGTTGACCTCGGATCAGGTAGGAATACCCGCTGAACTTAA

>OTU_625

GAAATGCGATAAGTAATGTGAATTGCAGAATTCAGTGAATCATCGAATCTTTGAACGCACATTGCGCCCGCCAGCATTCT

GGCGGGCATGCCTGTTCGAGCGTCATTTCAACCCTCAAGCACCGCTTGGCGTTGGGGCCCTACGGCTTCCGTAGGCCCCG

AAATACAGTGGCGGACCCTCCCGGAGCCTCCTTTGCGTAGTAACATACCACCTCGCACTGGGATCCGGAGGGACTCCTGC

CGTAAAACCccccAATTTTCCAAAGGTTGACCTCGGATCAGGTAGGAATACCCGCTGAACTTAA

>OTU_626

AAAGAGATCGTGGCCCGTCTGCGCAAGATTAATGCGGTGGCGCAGATCCATGAGGCGCAGCATGGCGGCGTGAAGCTGAC

GGATATTCTGGACCGTGGCGGCTTTGATCTGACGCGTGCGCTCAAGACGATGCCGGACTTTCTGGAGAACGAGCATCATT

CGCATGAGGAAGGCATCACCAGCATTTCCCTGAGCATCAAGGACCTGATCGACCAGCGCCGTTTTCAGGCCTGGATCGGC

GCGATCCTTCAGGAGCAGGGTGCGGATATCCTGCGGGCCAAGGGCATCCT

>OTU_627

GAAATGCGATACGCAATGCGAATTGCAGAATTCCGCGAGTCATCAGATCTTTGAACGCAAGTGGCGCTGGTTTAACCGCC

AGCATGTTTGTTTCAGTgtgtTAGGAATCATACAACTCAATGCGATTGATGCGATAAAGGCCTCAAaaaCCTTTAAAACT

TCtctcGTTAAACATGGAGGCGCCGAGCCGCCGTCGAACGAAGTAGTCACTTCTCGTAAGTGATCTCGTGCGATAAGCgc

gcTCACTCAGGCACTCACATAACAACAACCTCACTCCACAACACCTGAAATCAAGCAAGATCACCCGCTGAACTTAA

>OTU_628

GAAATGCGATACGTAATGTGAATTGCAGAATTCCGTGAATCATCGAATCTTTGAACGCACATTGCGCCCCTTGGTATTCC

AGGgggCATGCCTGTTTGAGCGTCATTTCCTTCTCAAACATTCTGTTTGGTAGTGAGTGATACTCTTTGGAGTTAACTTG

AAATTGCTGGCCTTTTCATTGGATGTTtttttttttCCAAAGagagGTTTCTCTGCGTGCTTGAGGTATAATGCAAGTAC

GGTCGTTTTAGGTTTTACCAACTGCGGCTAATCTTttttATACTGAGCGTATTGGAACGTTATCGATAAGAAGagagCGT

CTAGGCGACAATGTTCTTAAAAGTTTGACCTCAAATCAGGTAGGAGTACCCGCTGAACTTAA

>OTU_629

GAAATGCGATAAGTAATGTGAATTGCAGAATTCAGTGAATCATCGAATCTTTGAACGCACCTTGCGCTCCTTGGTATTCC

GAGGAGCATGCCTGTTTGAGTGTCATTAAATTCTCAACCTTTACAACTTTGTGTTGTCTAGATTGGAAGTGGgggCTGCT

TGtgtgGAATCCAATTCCTCACTCCCCTTAAATGTATTAGCTGGAATGCCTCTGCATATCTGACTATCAGTGTGATAATT

ATCTATGCTGTAGTTGTTGCATGAGGGTATCTGCTCAATCAATCGTCGCAAGACAATTATTGACCATCTTGACCTCAAAT

CAGGTAAGACTACCCGCTGAACTTAA

>OTU_630

GAAATGCGATAAGTAGTGTGAATTGCAGAATTCAGTGAATCATCGAATCTTTGAACGCACATTGCGCCCCTCGGCATTCC

GTGGGGCATACCTGTTCGAGCGTCATTTCAACCCTTAGGCGGTCCTTTTGGTCTGCCTGGACTTGGGCGTCGTCCAAGGG

CGTGCCTTAAaaaTATTGGCGGCGTAGGTGCGGCTTCGAGCGTAGCAGATGCGATTCGCTTTGGAAGCCGGCGCTCACCT

TGCCAGAATAATCTCTGAATGGCTGACCTCGGATCAGGTAGGGATACCCGCTGAACTTAA

>OTU_631

GAAATGCGATAAGTAATGTGAATTGCAGAATTCAGTAAATCATCAAATCTTTGAACGCACATTACGCCCGCCAGCATTCT

GGCAGGCATGCCTGTTCAAGCGTCATTTCAACCCTCAAGCTCCGCTTGGTGTTAGGGCCCTACAGCTGATGTAGGCCCTC

AAAGGTAGTGGCGGACCCTCCTGGAGCCTCCTTTGCGTAGTAACTTTACGTTTCGCACTGGGATCCAGAGGGACTCTTGC

CGTAAAACCccccAATTTTCCAAAGGTTGACCTCAGATCAGGTAGGAATACCTGCTGAACTTAA

>OTU_632

GAAATGCGATACGTAGTGTGAATTGCAGAATCTCGTGAATCATCGAATCTTTGAACGCACATTGCGCCCTATGGTATTCC

GTAGGGCATGCCTGTCTGAGCGTCAGCATCACCCTCCCAAGTGGTATTGCATTTGGGGACTCATTGGATAAGAAGGTTAA

GCCTCCCATGGGTGCAATTCTTCTGTCCAGAAATCTATAGGCAGTATTGGTTTGTTCTCCTATACTGGGCGTAATAATTG

GTTTTATTCTCGTCTGGAAAAGGTGAATAAGTGCTTGCCTTGAACCCACAAATTTATCTTAACTGGGTGACCTCAGATCA

GGTAGGGATACCCGCTGAACTTAA

>OTU_633

GAAATGCGATAAGTAATGTGAATTGCAGAATTCAGTGAATCATCGAATCTTTGAACGCACATTGCACCCGGTGGTATTCT

GCCGGGTATGCCTGTTCGAGCGTCATTTCTACCCTCAGCCCTTGCTGCTCAGGCGGCGTAGGTGCTGGTGTTGGGACTTT

ACAGCGCTGGTTTTCAGCGCTGTAGCCCCTAATTCCATCGGCGTACGCGTGTACGTTGCCcccTGCGTAGTGGTTCTTTC

TCGCATCGGGCCGTGCCGCCGTGCTGCCGCAACCTACATTCAATAAGTTTGACCTCGAATCAGGTAGGGTTACCCGCTGA

ACTTAA

>OTU_634

GAAATGCGATAAGTAGTGTGAATTGCAGAATTCAGTGAATCATCGAATCTTTGAACGCACATTGCGCCCATTGGTATTCC

AATGGGCATGCCTGTTCGAGCGTCATTTGTACCCTCAAGCCTTGCTTGGTGTTGGGCGTTTGTCTTTTGAGACTCGCCTT

AAAACGATTGGCAGCCGGCATACTGGTTTCGGAGCGCAGCACAAATTGCGGTCTATCCACGAATGTTGGCGTCCATGAAG

CCcccATTTTCCACTTTTGACCTCGGATCAGGTAGGGATACCCGCTGAACTTAA

>OTU_635

GAAATGCGATAAGTAGTGTGAATTGCAGAATTCAGTGAATCATCGAATCTTTGAACGCACATTGCGCCCCTTGGTATTCC

ATGGGGCATGCCTGTTCGAGCGTCATTTGTACCCTCAAGCTTTGCTTGGTGTTGGGTGTTTGTCCTTTCCcccGCGTTTG

GACTCGCCTGAAAACAATTGGCAGCCAGTGTTTTGGTATTGAAGCGCAGCACATCTTGCGCCTCGGGCCGACGAGACTGG

CGTCCACAAAGCCTCTTtttACACTTTTGACCTCGGATCAGGTAGGGATACCCGCTGAACTTAA

>OTU_636

GAAGTGCGATAAGCAATGCGAATTGCAGAATTCCGCGAATCATCAGATCTTTGAACGCAAATGGCGCCAGAGGGATATCC

TTCCGGCATGTCTGTTTCAGTGTCTGAGAGGTTTCACACTTATCTTAATGTGATTGACTTGTTGGATGGTGGAAGCTTCA

CTGTTTCTACCTTCTCAATCGTCtctcATGAAAGATGCGCGAGTGGATCTTTCGGTCCAATGAAGTGGTCACATCACTCG

CATGTGATTTCATTGGAAGCACCGAATTATCCGCATCCTCGCGGTTCCACAGCACTACTCACACTAGTGCTACCCTCCAT

ACCCTGCATCTGAGACAGGCAAGATCACCCGCTGAACTTAA

>OTU_637

GAAATGCGATAAGTAATGTGAATTGCAGAATCAGTGAATCATCGAGTCTTTGAACGCATCTTGCGCCCTTTGGTATTCCG

AAGGGCATGCCTGTTTGAGTGTCATGAAAACCTCAATCCCTCCGGTTTTGATTAACCGGTTGGACTTGGACTTGGGTGCT

GCCGCGGCTCGCGCCGTCGGCTCGCCTTAAAAGTGTTAGTGGGACGGTGAACACCCGTCAGCCTGGCGTAATAAGTTTCG

CTGGGCCTCTGGGGTCGTTCGACGGCTTGCTCATAACAAACCTTTTATTTCTATGACTCTGACCTCAAATCAGGTAGGGC

TACCCGCTGAACTTAA

>OTU_638

GAAATGCAATAAGTAATGTGAATTGCAGAATTCAGTAAATCATTAAATCTTTGAACGCATATTACGCCTGCTAGCATTCT

AGCAGGTATGCCTATTTAAGCGTCATTTCAACCCTCAAGCTCTACTTAGTGTTAGAGCCCTACAGCTAATATAGGCCCTC

AAAAGTAGTGGCAGACCCTCCTAGAGCCTCCTTTGCATAGTAACTTTACGTCTCACACTAGGATCCAGAGGAACTCTTAC

TATAAAACCcccTAATTTTCTAAAGGTTGACCTCAGATCAAGTAGAAATACCCGCTAAACTTAA

>OTU_639

GAAATGCAATAAGTAATGTGAATTGCAGAATTCAGTGAATCATCAAATCTTTGAACGCACATTGCGCCCGCCAGCATTCT

GGCGGGCATGCCTGTTCAAGCGTCATTTCAACCCTCAAGCTCCACTTAGTGTTAGGGCCCTACAGCTAATATAGGCCCTC

AAAAGTAGTGGCAGACCCTCCTAGAGCCTCCTTTACGTAGTAACTTTACGTCTCGCACTAGGATCCAGAGGGACTCTTGC

CATAAAACCccccAATTTTCCAAAGGTTGACCTCAGATCAAGTAGGAATACCCGCTGAACTTAA

>OTU_640

GAAATGCGATAAGTAATGTGAATTGCAGAATTCAGTGAATCATCGAATCTTTGAACGCACATTGCGCCCCGTGGTATTCC

GCGGGGCATGCCTGTTCGAGCGTCATTTCACCACTCGAGTCTGACTCGGTATTGGGCGCCGCGCACCTGCCGcgcgcCTC

AAAGTCTCCGGCTGGGCAGCCCGTCTCCGAGCGTTGTGGCATCAAGTCTCGCTAGGGAGTCGCGGGCGGCGTCGGCCGTA

AAACACCCCATCAAAGGTTGACCTCGGATCAGGTAGGGATACCCGCTGAACTTAA

>OTU_641

GAAATGCGATAAGTAATGTGAATTGCAGAATTCAGTGAATCATCGAATCTTTGAACGCACCTTGCGCCCCTTGGTATTCC

GAGGGGCACGCCTGTTCGAGTGTCGTGAAATTCTCAACTCAATCCTCTTGTTATGAGAGGGCTGGGCTTGGACTTGGAGG

TCTTGCCGGTGCTCCCTCGGGAAGTCGGCTCCTCTTGAATGCATGAGTGGATCCCTTTTGTAGGGTTTGCCCTTGGTGTG

ATAATTATCTACGCCGCGGGTAGCCTTGCGTTGGTCTGCTTCTAACCGTCTTCGGACAACTTTCATCTCAACTTGACCTC

GAATCAGGCGGGACTACCCGCTGAACTTAA

>OTU_642

GAAATGCGATAAGTAATGTGAATTGCAGAATTCAGTGAATCATCGAATTCTTGAACGCACATTGCGCCCCTTGGTATTCC

GAGGGGCAGTGCCTGTTCGATGCGTCATTACATCCACTCAAAGCCTATTGCTTGGTATTGGGCGTCGTCCTTAGTTGGGC

gcgcCTTAAAGAACCTCGGCGAGGCCCATCTCCGGCCTTTAGGCGTAGTAGGAATTTATTCGAACGTCTGTCAAAGGAGA

GGAACTCTGCCGACTTGAAACCTTTATTtttCTAGGTTGACCTCGGATCAGGTAGGGATACCCGCTGAACTTAA

>OTU_643

GAAATGCGATAAGTAATGTGAATTGCAGAATTCAGTGAATCATCGAATCTTTGAACGCACATTGCACCCTCTGGTATTCC

AAGGGGTATGCCTGTTCGAGCGTCATTACAACCCTCAAGCACTGCTTGGTTTTGGATGTTACCATTGTGGTGCATCTCAA

AAGTATTGGCAGTAGCATTCAGCTTCTAGCGTAGTAGATTTCCTCGCTTTGGAGTTGAAGTGTCTAATTGCTAGAACCCT

TAATTTGTCAAAGGTTGACCTCGGATCAGGTAGGGATACCCGCTGAACTTAA

>OTU_644

GAAATGCGATAAGTAGTGTGAATTACAGAATTTAGTGAATTATCGAATCTTTAAACGCATATTGCGCCCCTCGGTATTCC

GTAGGGCATGCCTGTTTGAGCGTTATTTACCCCTTAAGCTCTGCTTGGTGTTGGGCGTTGTCCCGCTTTGCGCGAGAACT

CGCCTTAAAGGTATTGGCAGCAGTCGTGCCACCCCTCGCGCAGCATATTGCGCTTCTTAAGGCGGTTAATTAGCGTCTAC

AAAGCTTATATTACATCTTAACCTCGGATTAGGTAGGGATACCCGCTGAACTTAA

>OTU_645

GAAACGCGAAAGGTAATGCGAATTGCAGAATCAGTGAGTCATCGAATCTTTGAACGCACATTGCGCCTCTCGGTATTCCG

GGAGGCACGCCTGTTCGAGCGCCATTACAAACTCCAAGCGTGGCTTGATGTTGGGCGATGTCTTtttGCGAGAGCGGAGA

CACGTCTGGAACGCGTTGGCGCCGCCGCCGGACCACGAGCGTAGCAAGACGAGAGTCAATCGCATCGATTTGGTTTCCGA

CGGAGGTCCGCCTCTGGATTGGACTTGTCCATTCCTACAACTTCTCAAGTTTGGCCTCGGATCAGGCGGGGATACCCGCT

GAACTTAA

>OTU_646

GAAATGCGATAAGTAATGTGAATTGCAGAATTCCGTGAATCATCGAGTCTTTGAACGCACATTGCGCTCCCTGGTATTCC

GGGGAGCATGCCTGTCCGAGCGTCATTTTCAACCCTCAAGCCTGGCTTGtgtgATGGGCCAGCGTCCCCACGACCTCCCG

GGGAGAGGGACGGGCCCGAAATGCAGTGGCGGCGTCCAGGTGATCGACCCGAGTGTATGGGGCTTTGTCACACGCTCGCG

GTATGCCTTCCGGCGCCTGCCTTTAAACCCTCTTTCTAAGGTTGACCTCGGATCAGGTAGGGATACCCGCTGAACTTAA

>OTU_647

GAAATGCGATAAGTAATGTGAATTGCAGAATTCAGTGAATCATCGAATCTTTGAACGCACCTTGCGCTCCTTGGTATTCC

GAGGAGCATGCCTGTTTGAGTGTCATGAAACTCTCACCCTCTAGCTTTCTTAATCGTGGCTAGCGGCGTGGACGTGAGCG

CTGCTGCTTTGTTGCGGCTCGCTCGAAATGCATTAGCAGACCCTTTTCGTAATCGGTTCCACTCAACGTGATAAGTATTT

CGTTGAGGACAGTTGCAGCAATGCGGCTGGCCGGGATAAGAAAGGCATAGTTGTCAGCTTCTAATCGCCCTTGGGCAATT

ttttATGATCTGGCCTCAAATCAGGTAGGACTACCCGCTGAACTTAA

>OTU_648

GAAATGCGATAAGTAGTGTGAATTGCAGAATTCAGTGAATCATCGAATCTTTGAACGCACATTGCGCCCCTTGGTATTCC

ATGGGGCATGCCTGTTCGAGCGTCATTTGTACCTTCAAGCTCTGCTTGGTGTTGGGTGTTTGTCAGAATGTATTttttGA

CTCGCCTTAAATGTATTGGCAGCCGGTATATTGGCTTATGAGCGCAGCACATTTTGCGATCATCTCCAGTGGACTAGCCc

ccAATAAGCTTACATTttttACTCTTGACCTCGGATCAGGTAGGGATACCCGCTGAACTTAA

>OTU_649

GAAATGCAATAAGTAATGTGAATTGCAGAATTCAGTGAATTATTAAATCTTTAAACGCATATTACGCCTGCTAGCATTCT

AGCAGGCATGCTTATTTAAGCGTCATTTCAACTCTTAAGTTCTGCTTAGTGTTAGGGCTCTACAGCTGATGTAGGCCTTT

AAAGGTAGTAGTAGACCTTCTTAGAGCCTCCTTTGTGTAGTAACTTTACGTCTCACACTAGGATCTAGAGGGACTCTTGC

TATAAAACCCTCTAATTTTCTAAAGGTTAACCTCAGATCAAGTAGAAATACCTACTGAACTTAA

>OTU_650

GAAATGCGATAAGTAGTGTGAATTGCAGAATTCAGTGAATTATCGAATCTTTAAACGTATATTGCGCCTCTTGGTATTCC

GTAGGGCATGCCTGTTCGAGCGTTATTTACCCCTTAAGCTCTGCTTGGTGTTGGGCGTTGTCCCGCTTCGcgcgAGGACT

CGCTCTAAAGGTATTAGCAGCGGTCGTGCTACCCCTCGCGCAGTATATTACGCTTCTTAAGGCGGTTGATTAGCGTCTAC

AAAGCTTACATTATATCTTAACCTCGGATTAGGTAGGGATACCCGCTGAACTTAA

>OTU_651

GAAATGCGATAAGTAGTGTGAATTGCAGAATTCAGTGAATCATCGAATCTTTGAACGCACATTGCGCCCCTTGGTATTCC

ATGGGGCATGCCTGTTCGAGCGTCATTTGTACCCTCAAGCTCTGCTTGGTGTTGGGTGTTTGTCATACGACTCGCCTTAA

AACAATTGGCAGCCGGCACGATAGCCTGAAGCGCAGCACATTTTGCGCCTCTTGCTATTGCTGTTGGCATCCATCAAGAT

CTTTTGCTCTTGACCTCGGATCAGGTAGGGATACCCGCTGAACTTAA

>OTU_652

GAAATGCGATACGTAATGTGAATTGCAGAATTCAGTGAATCATCGAATCTTTGAACGCATATTGCGCTCTTTGGTATTCC

GAAGAGCATGCTTGTTTGAGTATCAGTAAACACCTCAAAGACTTTAATTTGTTTTGAATTCTTTGGACTTGAGCAATCCC

AACACCAGTCTTTACGATCGGCGGAGGGTTGCTTGAAATGCAGGTGCAGCTGGACATTCTCCTGAGCTATAAGCATATTT

ATTTAATCCCGTCAAACGGATTATTACTTTTGCTGCAGCTAACATAAAGGGAGTTTGACCGTATTGGCTGACTGATGCAG

GATTTCACAAGGGTCGGCAACGACTCTTGTAAAACTCGATCTCAAATCAAGTAAGACTACCCGCTGAACTTAA

>OTU_653

GAAATGCGATAAGTAATGTGAATTGCAGAATTCAGTGAATCATCGAATCTTTGAACGCACCTTGCGCTCCTTGGTATTCC

GAGGAGCATGCCTGTTTGAGTGTCATTAAATTCTCAACCTTACCATCTTTTGATGAAAAGGCTTGGACTTGGgggTTCTA

TTTGCAGGCTTCAATCATATGAAGTTAGCTCCCCTTAAATGTATTAGCCGGAAAACCCTAGCGGTCCGTCTATTGGTGTG

ATAATTATCTACGCCGTGGACTTAGGACTGCCTTTTGTAATGGGATTTCTGCTTCAAATCCGTCCTTAGGGACAATTATT

GACCATTTGACCTCAAATCAGGTAGGACTACCCGCTGAACTTAA

>OTU_654

GAAATGCGATAAGTAATGTGAATTGCAGAATTCAGTGAATCATCGAATCTTTGAACGCACATTGCGCCCTCCGGTACTCC

GGgggCATACCTGTTCGAGCGCCGTTGTACTACTGAGGCTTAGCCTTGCGGTGGCCGATGCTCTGACCCAGCGATGGGGC

GGAGGACGGCTCAAACGAATGGGCGTTTCCTGAGGTCTGGCCAGCGTAGCAAGTCTATTCGCTGAGGCAGAGGTTTGGGG

AGCGCCAGTTTTACGACACTTGGCCTCGGATCAGGTAGGGATACCCGCTGAACTTAA

>OTU_655

GAAATGCGATAAGTAATGTGAATTGCAGAATTCAGTGAATCATCGAATCTTTGAACGCACATTGCGCCCCTTGGTATTCC

GAGGGGCATGCCTGTTCGAGCGTCATTACACCACTCAAGCTATGCTTGGTATTGGGCGTCGTCCTTAGTTGGGCgcgcCT

TAAAGACCTCGGCGAGGCCACTCCGGCTTTAGGCGTAGTAGAATTTATTCGAACGTCTGTCAAAGGAGAGGAACTCTGCC

GACTGAAACCTTTATTTCTTGTACGTCGCTCGAGGTAGGGATACCCGCTGAACTTAA

>OTU_656

GAAATGCGATAAGTAGTGTAAATTGCAGAATTTAGTAAATTATCGAATCTTTAAACGCACATTACGCCCCTCGGTATTCC

GTAGGGCATGCCTATTCAAGCGTTATTTACCTCTTAAGCTCTGCTTAGTGTTAGGCGTTGTCCCGCTTCGcgcgAGGACT

CGCCCTAAAGGTATTGGTAGCGGTTATGCTACCCCTCGCGCAGCATATTACGCTTCTTAAGGCGGTTAATTAGCGTCCAC

AAAGCTTATATTATATCTTAACCTCGAATTAGGTAGGGATACCCGCTAAACTTAA

>OTU_657

GAAATGCGATAAGTAATGTGAATTGCAGAATTCAGTGAATCATCGAATCTTTGAACGCACATTGCGCCCGCTAGTATTCT

GGCGGGCATGCCTGTTCGAGCGTCATTTCAACCATCAAGCCCCAGGCTTGTGTTGGGGCCCTGCGGCCGTCCGCAGCCcc

cGAAAAGCATTGGCGGGCTCGTTGTCACACCGAGCGCAGTAGTATATCTTCGCTAGGACGTGATTGCGGGTTCCGGCCGT

GAAACCCTCTAATTTATCAAGGTTGACCTCGGATCAGGTAGGAATACCCGCTGAACTTAA

>OTU_658

GAAATGCGATAAGTAATGTGAATTGCAGAATTCAGTGAATCATCGAATCTTTGAACGCACATTGCGCTCCCTGGTATTCC

GGGGAGCATGCCTGTTCGAGCGTCATTATCAACCCTCAAGCCCTGCTTGGTATTGGGTGCCGTCCCTGCTGTGGACGCGC

CCTAAAGACGTTGGCGGCGGTGTCATACCTCAAGCGTAGTAAAATCTCCAGCTTTCTTGGTGTGACATTGCTTTGCCTCC

AGAAACCCATCTTtttACAGGTTGACCTCGGATCAGGTAGGGATACCCGCTGAACTTAA

>OTU_659

GAAATGCGATAAGTAATGTGAATTGCAGAATTCAGTGAATCATCGAATCTTTGAACGCACATTGCGCCCCTTGGTATTCC

GAGGGGCATGCCTGTTCGAGCGTCATTACAACCCATCAAGCTCTGCTTGGCCTTGGGCTCCGCCcccGCGAGGGGACGGG

CTCTAAAGAACTCAGCGGTGCCGTGAGGCCCCGAGGCGTAGTAAATTCTATCCCGCTTCGTGGACCTCGCGTGTGCCCGC

TTCCAACGACCAACACCTTCTAGGTTGACCTCGGATCAGGTAGGGATACCCGCTGAACTTAA

>OTU_660

GAAATGCGATAAGTAATGTGAATTGCAGAATTCAGTGAATCATCGAATCTTTGAACGCACATTGCGCCCCGTGGTATTCC

GCGGGGCATGCCTGTTCGAGCGTCATTTCACCACTCAAGCCTCGCTTGGTATTGGGCGCCGCGTGATTTCCACGcgcgcC

TCAAAGTCTTTCCGGCTGAGTCGTCCGTCTCCCAGCGTTGTGATACATTTTCGCTTCGGAGTGCGGGTGCGCGGCCGCTA

AGTCTCCTCTCAAAGGTTGACCTCGGATCAGGTAGGGATACCCGCTGAACTTAA

>OTU_661

AAGCATTGGCTGTCTGAAGGCGCTCAGCCGACAGATCGCGTGGCACGCTTCCTTGGTAACGCAGGTCTGGCACCTAAGCC

CGCTTATCGTGAACAGCCTAAaaaGTCTGCTCCGAAGAAGAAGGCACAGGCACGCGCTGCTGAAGCCGCTAAAGCTGCTG

AAGCCGCTGCTGCCTAAAGCCTGAAACCATCATGTGCTCAAACCTCATTCTGATGGGCGTTGTTGGTAAACCGCATGGTG

TGCGCGGCCTTGTGCGTGTGCgcgcTTATGCGGAAAACCCGCAAACGCTGGAGCAGTGTGGG

>OTU_662

GAAATGCGATAAGTAATGTGAATTGCAGAATTCAGTGAATCATCGAATCTTTGAACGCACATTGCGCCCATCAGTATTCT

GGTGGGCATGCCTGTTCGAGCGTCATTTCAACCCTTAAGCCTAGCTTAGTGTTGGGAATCGACCGTAGGGTCGTTCCTTA

AAGACAGTGGCGGAGCGGCAGTGGTCCTCTGAGCGTAGTAAATTTATTTCTCGCTTTTGTCAGGCCCTGTCCTCCCGCCA

TAAAACCcccAATTttttAGTGGTTGACCTCGGATCAGGTAGGAATACCCGCTGAACTTAA

>OTU_663

GAAATGCGATAAGTAATGTGAATTGCAGAATTCCGTGAATCATCGAATCTTTGAACGCACATTGCGCCcccTGGTATTCC

GGggggCATGCCTGTCCGAGCGTCATTACAAACCCTCAAGCACGGCTTGtgtgATGGGCTGTAGCGTCCCTTTCCTTGAG

GGACGGGCCCCAAATGTAGTGTGCGGCGCGGTGCGCTTGGCTGCAGTGTATGGGAACTTCTCACACTCGCGGTGCGTCAC

CACACCTGCCATCTCTATCATTCTTCTCGGTTTGACCTCGGATCAGGTAGGGATACCCGCTGAACTTAA

>OTU_664

GAAATGCGATAAGTAATGTGAATTGCAGAATTCAGTGAATCATCGAATCTTTGAACGCACATTGCGCCcccTGGTATTCC

GGggggCATGCCTGTTCGAGCGTCATTTCACCACTCAAGCTCGCTTGGTATTGGCACGCGGTCCGCCGCGTGCCTCAAAT

CGACCGGCTGGGTCTTCTGTCCCCTAAGCGTTGTGGAAACTATTCGCTAAAGGGTGCTCGGGAGCTACGCCGTAAACAAA

CCATTCTCAGTTAGACCTCGGATCAGGTAGGGATACCCGCTGAACTTAA

>OTU_665

AAAATGCGATAAGTAATGTGAATTGCAGAATTGTGAATCATCGAATCTTTGAACGCACATTGCGCCTTTTGGTATTCCAA

AAGGCATGCCTGTTTGAGCGTGATTACATCTTCTTAAATCAACTTTATTGTTTGAATTTTAAGGTATTGGAAATTTTGTA

CTAATTttttAGTACAATTTTCTGAAATACATTGGTGATCTTTGTGAAATTCCAATTTATAAaaaaCGTATTAGGTTTTA

TCCACTCGTTtttAATTATATTATTGACTTTTCGCAAATTTTATAGATCTGTCCAAAATTTGTTTTATCACTTTCCACCT

CAAATCAAGTAGGACTACCCGCTGAACTTAA

>OTU_666

GAAATGCGATAAGTAATGTGAATTGCAGAATTCAGTGAATCATCGAATCTTTGAACGCACCTTGCGCTCCTTGGTATTCC

GAGGAGCATGCCTGTTTGAGTGTCATGAAATCTTCAACCTACAAGCTTTTGTGGTTTGTAGGCTTGGACTTGGAGGCTTG

TCGGCCGTTATCGGTCGGCTCCTCTTAAATGCATTAGCTTGGTTCCTTGCGGATCGGCTCTCGGTGTGATAATGTCTACG

CCGCGACCGTGAAGCGTTTGGCGAGCTTCTAACCGTCTTATAAGACAGCTTTATGACCTCTGACCTCAAATCAGGTAGGA

CTACCCGCTGAACTTAA

>OTU_667

GAAATGCGATACCTAGTGTGAATTGCAGCCATCGTGAATCATCGAGTTCTTGAACGCACATTGCGCCCGTCGGTATTCCG

GCGGGCATGCCTGTCTGAGCGTCGTTTCCTTCTTGAAGCTTtttttttAAAAGTTTAAATATCAGAATTGGCCGTGCCAC

TGGCCCGGCCGAAAAGAAACGTTGCGGACGAAGCGAACTACATCGGGACGCTTTTGCCGCCGAGCGAAAATatatCATTG

AGCTCGACCTCAGATCAGGTAGGAGTACCCGCTGAACTTAA

>OTU_668

GAAATGCGATAAGTAATGTGAATTGCAGAATTCAGTGAATCATTCGAATCTTTGAACGCACATTGCGCCCCTTGGTATTC

CGAGGGCATGCCTGTTCGAGCGTCATTACACCACTCAAGCTATTGCTTGGTATTGGGCGTCCGTTCCTTAGTTTGGGACG

CGCCTTAAAGACCTCGGCGAGGCCACTCCGGCTTTTAGGCGTAGTTAGAATTTATTCGAACGTCTGTCAAAGGAGAGGAA

CTCTGCCGACTGAAACCTTTATTtttCTAGGTTGACCTCGGATCAGGTAGGGATACCCGCTGAACTTAA

>OTU_669

AAGTGCATCGATGAAGAACGCATCAAGTGCATCGATGAAGAACGCATCAAGTGCATCGATGAAGAACGCATCAAGTGCAT

CGATGAAGAACGCATCAAGTGCATCGATGAAGAACGCATCAAGTGCATCGATGAAGAACGCATCAAGTGCATCGATGAAG

AACGCATCAAGTGCATCGATGAAGAACGCATCAAGTGCATCGATGAAGAACGCATCAAGTGCATCGATGAAGAACGCATC

AAGTGCATCGATGAAGAACGCATCAAGTGCATCGATGAAGAACGCA

>OTU_670

GAAATGCGATAAGTAGTGTGAATTGCAGAATTCAGTGAATCATCGAATCTTTGAACGCACATTGCGCCCCTTGGTATTCC

ATGGGGCATGCCTGTTCGAGCGTCATTTGAACTCTCAAGCTTTGCTTGGTGTTGGGTGTTTGTCCTTTTAGGACTCGCCT

CAAAATAATTGGCAGCCAGCGTATTAGTCTTGGAGCGCAGCACATTTTGCGATCTAGCTAGTAAATTGGCAACCATCAAG

CCTATTTTAACACTTGACCTCGGATCAGGTAGGGATACCCGCTGAACTTAA

>OTU_671

GAAATGCGATAACTAATGTGAATTGCAGAATTCAGTGAATCATCGAGTCTTTGAACGCACATTGCGCCcccTGGTATTCC

GGgggCATGCCTGTCCGAGCGTCATTGCTGCCCTCAAGCCCGGCTTGtgtgtTGGGTCGCCGTCCCCTCTCCGGgggACG

GGCCCGAAGGCAGCAGGCGGCACCGCGTCCGATCCTCGAGCGTATGGGGCTTTGTCACATGCTCTGGTAGGATTGGCCGG

CGCCTGCCGACGTTTTCCAACCATTCTTTCCAGGTTGACCTCGGATCAGGTAGGGATACCCGCTGAACTTAA

>OTU_672

GAAATGCGATAAGTAGTGTGAATTGCAGAATTCAGTGAATCATCGAATCTTTGAACGCACATTGCGCCCTTTGGTATTCC

AAAGGGCATGCCTGTTCGAGCGTCATTTGTACCCTCAAGCTTTGCTTGGTGTTGGGCGTCTTtttGTCCcccccccTTGC

GGGGAGACTCGCCTTAAAGTCATTGGCAGCCGGCCTACTGGTTTCGGAGCGCAGCACAAGTCGCGCTctctTCCAGCCCC

AAGGTCTAGCATCCAACAAGCCTTtttttCAACTTTTGACCTCGGATCAGGTAGGGATACCCGCTGAACTTAA

>OTU_673

GAAATGCGATACTTGGTGTGAATTGCAGAATCCCGTGAACCATCGAGTCTTTGAACGCAAGTTGCGCCCAAAGCCATTAG

GCTGAGGGCACGCCTGCCTGGGTGTCAcacaTCGTCACCCCTTCCTCCACACTTAACCAGTTAAATGTGAGGCAGTGGGT

GAAAGTTGACCTCCCGCGAGCCAGTTCCTCGTGGTTGGTTGAAAAGCAAGTTCGGGACGAGTCCcccACGATAACGGTGG

ATGAGCCCACGCTCGAGACCAATCGTGCCTGTGGGACTCCGACGTAACGGACTTATCGACCCCACACGCGCCCTCTGTGC

AAACCGAGTGTGCCATCTACGAGACCTCAGGTCAGGCGGGGCTACCCGCTGAGTTTAA

>OTU_674

GAAATGCGATAAGTAATGTGAATTGCAGAATTCAGTGAATCATCGAATCTTTGAACGCACATTGCGCCCCTTGGTATTCC

GAGGGGCATGCCTGTTCGAGCGTCATTACACCACTCAAGCTATGCTTGGTATTGGGCGTCGTCCTTAGTTGGGCgcgcCT

TAAAGACCTCGGCGAGGCCACTCCGGCTTTAGGCGTAGTAGAATTTATTCGAACGTCTGTCAAAGGAGAGGAAACTCTGC

CGACTGAAACCTTTATTtttCTTAGGTTGAACCTTCTGGATCAGGTAGGGGATACCCCGCTGAACTTA

>OTU_675

GAAATGCGATAACTAATGTGAATTGCAGAATTCAGTGAATCATCGAGTCTTTGAACGCACATTGCGCCcccTGGTATTCC

GGggggCATGCCTGTCCGAGCGTCATTGCTGCCCTCAAGCCCGGCTTGtgtgtTGGGCCCTCGTCCcccGGCTCCCGGgg

gACGGGCCCGAAAGGCAGCGGCGGCACCGCGTCCGGTCCTCGAGCGTATGGGGCTTCGTCTTCCGCTCCGTAGGCCCGGC

CGGCGCCCGCCGACGCATTTGTTTGCAACTTGTTtttttCCAGGTTGACCTCGGATCAGGTAGGGATACCCGCTGAACTT

AA

>OTU_676

GAAATGCGATAAGTAATGTGAATTGCAGAATTCCGTGAATCATCGAATCTTTGAACGCACATTGCGCCcccTGGTATTCC

GGggggCATGCCTGTCCGAGCGTCATTTCAAACCCTCAAGCACGGCTTGtgtgATGGGCCATCCGTCCCCGTAGCAGGGG

ACGGGCCCCAAATGGAGTGTGCGGCGCGGTGCGCTTAGCTGCAGTGTATGGGAACCTCTCACACTCGCGGTGCGTCACCA

CGCCTGCCATCCACCTAATCTTCTCGGTTTGACCTCGGATCAGGTAGGGATACCCGCTGAACTTAA

>OTU_677

GAAATGCGATACGTAATGTGAATTGCAGAATTCCGTGAATCATCGAATCTTTGAACGCACCTTGCGCCCCTTGGTATTCC

GAGGGGCATGCCTGTTTGAGTGTCATTGTAATCtctcAACTTTGGCCTTTTGGTTtttCCTTAACCCAAGGCCTGTAGTT

TGGATGGTGGAGGTGTGCTGGCTTGCTCCCCTGTCGGTCAGCTCCTCTGAAAAGAATCAGCAAAGGGGAACGCACCTGTT

CCTGCATCTTGTGCTTCTGGCATTGATATAATTGTCTATGCCTGTTATGGCTCAGATGCCTTTTGGATCGGTTGAGTGTC

TTTGCTTACAGTTGTCCATCGGACATTtttGACAAATCACCTCAAATCAGGTAGGACTACCCGCTGAACTTAA

>OTU_678

GAAATGCGATAAGTATTGTGAATTGCAGATATTCGTGAATCATCGAATCTTTGAACGCACATTGCGCCCTCTGGTATTCC

AGGgggCATGCCTGTTTGAGCGTCATTTCCTTCTCAAACCTCGGTTTGGTAGTGAGTGGTACTCTTTCTGGGTTAACTTG

AAAATGCTGGCCATCTGGCTGTTGCTGACTGAGGTTTTAGTCCAGTCCGCTGATACTCTGCGTATTAGGTTTTACCAACT

CGTAGTGGCGTTAGTAGGCGTTTTAAAGGCTTTTACTGAAAGTACAGACAGTCTGGCAAACAGTATTCATAAAGTTTGAC

CTCAAATCAGGTAGGATTACCCGCTGAACTTAA

>OTU_679

GAAATACGATAAGTAGTGTAAATTACAGAATTTAGTAAATTATTAAATCTTTAAACGTATATTACGCTCCTCGGTATTCC

GTAGAGCATGCCTATTTAAGCGTTATTTACCTCTTAAGCTCTGCTTAGTGTTAGGCGTTATCCCGCTTCGCGTAAGAACT

TGCTCTAAAGGTATTAGTAGCGGTCGTACTACCCCTCGCGTAGTATATTACGCTTCTTAAGGCGGTTAATTAGCGTCTAT

AAAGCTTATATTATATCTTAACCTTAAATTAGGTAGAAATACCCGCTAAACTTAA

>OTU_680

GAAATGCGATAAGTAGTGTGAATTGCAGAATTCAGTGAATCATCGAATCTTTGAACGCACATTGCGCCCCTTGGTATTCC

ATGGGGCATGCCTGTTCGAGCGTCATTTATACCTTCAAGCTATGCTTGGTGTTGGGTGTTTGTCCTCTCCCCTGCGTTTG

GACTCGCCTTAAAGTCATTGGCAGCCTGTATTTTGGTTTTGAGCGCAGCACATTTTGCGTCTTGCATCTAGTAACACTAG

CATCCATCAAGCCCATTATCACTTTTGACCTCGGATCAGGTAGGGATACCCGCTGAACTTAA

>OTU_681

GAAATGCGATAAGTAATGTGAATTGCAGAATTCAGTGAATCATCGAATCTTTGAACGCACATTGCGCCCCTTGGTATTCC

GAGGGGCATGCCTGTTCGAGCGTCATTACACCACTCAAGCTATGCTTGTATTGGTCGGTCGTCCTTACGTTGGCTGGCGC

CTTAAAGACCTCGGCGAGGCCACTCCGGCTTTAGGCGTAGTAGAATTTATTCGAACGTCTGTCAAAGGAGAGGAACTCTG

CCGACTGAAACCTTTATTtttCTAGGTTGACCTCGGATCAGGTAGGGATACCCGCTGAACTTAA

>OTU_682

TTGACCTTGAAGCTACCTTCAAGGTTAGGCTCTGACCAGTATCCCGTTATTGATCGGCCAGGAGGCGTATCATGTCCTTT

GTCACCACATCTGACGGTGTAGAGATTTTCTATAAGGACTGGGGATCGAAGGATGCCCAGCCGATTGTTTTCCATCATGG

CTGGCCGCTGAGTGCCGATGACTGGGACGCGCAGATGCTGTTCTTCCTGAGCAAGGAATTTCGCGTCATCGCGCATGATC

GTCGCGGTCACGGACGGTCGGCACAGGTGAGTGACGGTCATGACATGGACCATTACGCGGCTGACGCATCGGCCGTCGTG

GAACACCTCGATCTGCAAAATGCCATTCATGTCGG

>OTU_683

GAAATGCGATAAGTAATGTGAATTGCAGAATTCAGTGAATCATCGAATCTTTGAACGCACCTTGCGCCCTTTGGCCATTC

CGAAGGGCATGCCTGTTTGAGTATCATGAACATCTCAACTCTCATGGTTtttCCATGATGAGCTTGGACTTTGGgggTCT

TGCTGGCCGGTGGTCGGCTCCTCTCAAATGCATCAGCTTACCAGTGTTTGGTGGCATCGCAGGTGTGATAACTATCTACG

CTTATGATTTTCCACCAGGTGACCTTCAGCGATGGAGGCTCACTGGAGCTCACAGACGTCtctctTCAGTGAGGACAGCC

CTTTGAATGTTTGATCTCAAATCAGGTAGGATTACCCGCTGAACTTAA

>OTU_684

GAAATGCGATACGTAGTGTGAATTGCAGAATTCAGTGAATCATCGAATCTTTGAACGCACATTGCGCCCTTTGGTATTCC

AAAGGGCATGCCTGTTCGAGCGTCATTTGTACCCTCAAGCTTTGCTTGGTGTTGGGCGTCTTGTCTCCAGTCCGCTGGAG

ACTCGCCTTAAAGTCATTGGCAGCCGCCTACTGGTTTCGGAGCGCAGCACAAGTCGCACTCTTTTCCAGCCAAGGTCAGC

GTCCAACAAGCCTTtttCAACTTTTGACCTCGGATCAGGTAGGGATACCCGCTGAACTTAA

>OTU_685

GAAATGCGATAAGTAGTGTGAATTGCAGAATTCAGTGAATCATCGAATCTTTGAACGCACATTGCGCCCCTTGGTATTCC

AGTGGGGCATGCCTGTTCGAGCGTCATTTGTACCTTCAAGCTCTGCTTGGTGTTGGGTGTTTGTCTCGCCTCTGCGCGTA

GACTCGCCTCAAACAATTGGCATGCCGGCGTATTGATTTCGGAGCGCAGTACATCTCGCGTCTTTGCACTCATAACGACG

ACGTCCAAAAGTACATTtttACACTCTTGACCTCGGATCAGGTAGGGATACCCGCTGAACTTAA

>OTU_686

GAAATGCGATAAGTAATGTGAATTGCAGAATTTAGTGAATTATTAAATCTTTGAACGCATATTGCGCCTGCTAGCATTTT

AGTAGGTATGCCTATTTGAGCGTTATTTTAACCCTCAAGTTCTGCTTAGTGTTAAGGCTCTACAGCTGATATAGGCTTTT

AAAGGTAGTAGCAGACCTTtttGGAGCCTCCTTTGCGTAGTAACTTTACGTCTCGCACTAGGATCTGGAGGGACTTTTGC

TGTAAAACCCCTTAATTTTCTAAAGGTTAACTTTAGATTAGGTAGGAATACCTGCTGAACTTAA

>OTU_687

GAAATGCGATAAGTAATGTGAATTGCAGAATTCAGTGAATCATCGAATCTTTGAACGCACATTGCGCCCCTTGGTATTCC

GGggggCATGCCTGTTCGAGCGTCATTTCAACCCTCAAGCTTAGCTTGGTATTGAGTCTATGTCAGTAATGGCAGGCTCT

AAAATCGGTGCgcgcCGCTGGTGCTGACGTAGTAATATCtctcGTTACAGGTTCTCGGTGTGCTTCTGCCAAAACCCAAA

TTtttCTATGGTTGACCTCGGATCAGGTAGGGATACCCGCTGAACTTAA

>OTU_688

GAAATGCGATAAGTAATGTGAATTGCAGATTTTCAGTGAGTCATCGAATCTTTGAACGCACCTTGCGCTCCTTGGTATTC

CGAGGAGCATGCCTGTTTGAGTGTCATTAAATTCTCAACTCCAAGGGTTTCACGACCTTTCGGAGATTGGACTTGGAGGT

GCTGGCCGCATATTGGTCGGCTCCTCTCAAATGCATCAGCGGAATTCAACCTTTGGCTTCCGGATGTCAGTGTGATAATC

ATGTTGCGCTGTCGTTTGATCCCTCAGAGTCCGCTTATAATGGTCTTCGGACAAAATCATCATCAAATTTGACCTCAAAT

CAGGTAGGACTACCCGCTGAACTTAA

>OTU_689

GAAATGCGATACGTAGTGTGAATTGCAGAATTCAGTGAATCATCGAATCTTTGAACGCACATTGCGCCCTTTGGTATTCC

AAAGGGCATGCCTGTTCGAGCGTCATTTGTACCTTCAAGCTTTGCTTGGTGTTGGGTGTTttttGTCTCCCTCTTTCTGG

GAGACTCGCCTTAAAACGATTGGCAGCCGGCCTACTGGTTTCGGAGCGCAGCACATTttttGCGCTCTGTATCAGGAGAA

AAGGACGGTACTCCATCAAGACCTCTACATTtttAACTTTTGACCTCGGATCAGGTAGGGATACCCGCTGAACTTAA

>OTU_690

GAAATGCGATAAGTAATGTGAATTGCAGAATTCAGTGAATCATCGAATCTTTGAACGCACATTGCGCTCCCTGGTATTCC

GGGGAGCATGCCTGTTCGAGCGTCATTACAACCCTCAAGCTCTGCTTGGTATTGGGCTCTGCTGGAAACGGCAGGCCTTA

AAATCAGTGGCGGTGCCTCTCGGCTCCAAGCGTAGTAATTCTTCTCGCTTCGGATCACCGGGTgtgtgCTTGCCAACAAC

CcccAATTttttAAGGTTGACCTCGGATCAGGTAGGGATACCCGCTGAACTTAA

>OTU_691

GAAATGCGATAAGTAATGTGAATTGCAGAATTCAGTGAATCATCGAATCTTTGAACGCACATTGCGCCCGGTGGTATTCC

GCCGGGCATGCCTGTTCGAGCGTCATTTAAACCAATCCAGCCTCGTGCTCGGGTCTTGGGCCTTTCGCCTCTGGGCGGGC

CTCAAAATCAGTGGCGGTGCCCTCTGGCTCTAAGCGTAGTAATACTCCTCGCTATAGACGTCCGGTGGTGTCTTGCCAAC

AACCcccAATTTTCAAAGGTTGACCTCGGATCAGGTAGGGATACCCGCTGAACTTAA

>OTU_692

GAAATGCGATAAGTAATGTGAATTGCAGAATTCAGTGAATCATCGAATCTTTGAACGCACATTGCGCCCGCCAGTATTCT

GGCGGGCATGCCTGTCTGAGCGTCATTTCAACCCTCAGCCcccGTTCGCGGGGCGCTGGCGTTGGGGCCCGGCCGTCCTC

GCGGCGGCCGTCCCCGAAACACAGTGGCGGTCTCCCACAGACTCCCCTGCGTAGTAACACTACCTCGCAGAAGGGACGAG

CGGGCTGGCCACGCCGTAAAACACCCCACTTCTCAAGGTTGACCTCAGATCAGGTAGGAATACCCGCTGAACTTAA

>OTU_693

GAAATGCGATACTTGGTGTGAATTGCAGAATCCCGTGAACCATCGAGTCTTTGAACGCAAGTTGCGCCCGATGCCATTAG

GTTGAGGGCACGTCTGCCTGGGCGTCACATATCGAAGCCTCTCGCCAATTTCCTATATTGATAGGGGTATTGTGCAGGGC

GAATGTTGGCCTCCCGTGAGCTTTATTGCCTCATGGTTGGTTGAAAATCGAGACCTTGGTAGGGTGTGCCATGATAGGTG

GTGGCTGTGTTACGCACGAGACCAAGTAAGTCATGTGCTGCTCTATTGAATTTAGGCCTCTTTTACCCACATGCGTTTCG

AAACGCTCGTGATGAGACCTCAGGTCAGGCGGGGCTACCCGCTGAATTTAA

>OTU_694

GAAATGCGATACGTAATGTGAATTGCAGAATTCAGTGAATCATCGAATCTTTGAACGCACCTTGCACTCTTTGGTATTCC

GAAGAGTATGTCTGTTTGAGTGTCATGAAACTCTCAACCcccTAATTTTGTAATGAGATTAGTGTGGGCTTGGATCATGG

TTGTTGCCGGCTTCATTGTCGGCTCGACTGAAATACACGAGCTACCCATTTGAAATAGACGGTTTGACTCGGCGTAATAA

TTATTTCGCTGAGGACGTCTTCTTCAAAAGTCAGTGGTGCTTCTAATGCATTATAAATTTAAGCTTTAGACCTCAAATCA

GTCAGGACTACCCGCTGAACTTAA

>OTU_695

AAAATGCGATAAGTAGTGTGAATTGCAGAATTCAGTGAATCATCGAATCTTTGAACGCACATTGCGCCCCTTGGTATTCC

TTGGGGCATGCCTGTTCGAGCGTCATTTGACCCCTCAAGCCCAGCTTGGTGATGGGCGTCTGTCCCCGCCcccGcgcgAG

GACTCGCCCCAAAATCATTGGCGGCCGGTACGTTGGCTTCGAGCGCAGCAGAAACGCACCTCGAGCCCGGCGGACCGGCT

CCCAGAAGCTTTACCATCTTGACCTCGGATCAGGTAGGGATACCCGCTGAACTTAA

>OTU_696

GAAATGCGATAAGTAATGTGAATTGCAGAATTCAGTGAATCATCGAATCTTTGAACGCACATTGCGCCCATTAGTATTCT

AGTGGGCATGCCTGTTCGAGCGTCATTTCGACCCTTAAGCCTAGCTTAGTGTTGGGAATCTACCCTGTAGCTACCCTGTA

GTTACCCTGTAGTTCCTCAAATCGAACGGCGGAGTTGTGATATCCTCTGAGCGTAGTAGCTTATATCTCGCTTTTGTAGG

TGTTGCGGCCCCTGCCGGAAAACCCTTATTtttAATGGTTTGACCTCGGATCAGGTAGGAATACCCGCTGAACTTAA

>OTU_697

GAAATGCGATAAGTAATGTGAATTGCAGAATTTAGTGAATCATCGAATCTTTGAACGCACATTGCACCTCTTGGTACTCC

TTGAGGTATACCTGTTCGAGCGCCGTTTAAATCTAAGGCTTTGCCTTGTTTTGATGTTTTCTAAACCTCTTAATAGAGGT

TCATCTAAaaaTATTGGCGTCTTTCTTtttGACCCGAAACGCAGCAAaaaTGCGAATTAAGAGTTAAAAGATAAAGATAG

CCTTGTTATCTAtataAACATGGCCTCGGATCAGGTAGGGATACCCGCTGAACTTAA

>OTU_698

GAAATGCGATAAGTAGTGTGAATTGCAGAATTCAGTGAATCATCGAATCTTTGAACGCACATTGCGCCCCTTGGTATTCC

ATGGGGCATGCCTGTTCGAGCGTCATTTGTACCCTCAAGCTCTGCTTGGTGTTGGGTGTTTGTCTACTATCGTGGACTCG

CCTTAAAGTCATTGGCAGCCAGTGTTTTGGTATTGGAGCGCAGCACATTTTGCGCCTCTAGCCTAGAATACTAGCGTTCC

AGTTAAGCCTTtttCCACTTTTGACCTCGGATCAGGTAGGGATACCCGCTGAACTTAA

>OTU_699

AAAGTGCGATAACTAGTGTGAATTGCATATTCAGTGAATCATCGAGTCTTTGAACGCAACTTGCGCTCAATGGTATTCCA

TTGAGCACGCCTGTTTCAGTATCAAaaaCACCCCACATTCATAATTTTGTTGTGAATGGAAATGAGAGTTTCGGCTTTAT

TGCTGAATTCTTTAAAATTATTAGGCCTGAACTATTGTTCTTTCTGCCTGAACATTtttttAATATAAAGGAATGCTCTA

GTAAaaaGACTATCTCTGGGGCCTCCCAAATAAATCATTCTTAAATTTGATCTGAAATCAGGCGGGATTACCCGCTGAAC

TTAA

>OTU_700

GAAATGCGATACCTAGTGTGAATTGCAGCCATCGTGAATCATCGAGTTCTTGAACGCACATTGCGCCCGTCGGTATTCCG

GCGGGCATGCCTGTCTGAGCGTCGTTTCCTTCTTGGAACTTTTGTTAAAGAAAGATCCAGAGCTGGCCGTGCCACTGGCC

CGGCCGAAAAGAAACGTTGCGGACGAAGCGAACTACATCGGGACGCTTTTGCCGCCGAGCGAAAATatatCATGAGCTCG

ACCTCAGATCAGGTAGGAGTACCCGCTGAACTTAA

>OTU_701

GAAATGCGATAACTAATGTGAATTGCAGAATTCAGTGAATCATCGAGTCTTTGAACGCACATTGCGCCcccTGGTATTCC

GGggggCATGCCTGTCCGAGCGTCATTGCTGCCCTCAAGCACGGCTTGtgtgtTGGGCCCCGTCCCCGGTACCcccGGGG

ACGGGCCCGAAAGGCAGCGGCGGCACCGCGTCCGGTCCTCGAGCGTATGGGGCTTTGTCACCCGCTCTGCAGGCCCGGCC

GGCGCCAGCCGACCAACCCAACCATTttttACAGGTTGACCTCGGATCAGGTAGGGATACCCGCTGAACTTAA

>OTU_702

CAGCTGCGAGACTTGGTGTGAATTGCAGGACACATTGAGCACTGATTtttCGAACGCACATTGCGGCCTCGGGTCCTACC

CGGGGCCACGTCTGTCTGAGGGTCGGATGAATGCATGCCTAGGGAACTATTCCCACGTGGTCGCACCGGAAGCTCGTCTT

TCGCCGACCTAAGGCTTGGAGATTCCCACCGGTCGAGTGAGCGTCCCGCGTAGGATGCCCGACGGCGGAGTGGCGGAATG

CTAAACACTCTGTTCGTCGGGACTGCGGTGATAGACGCCGGAACCGGAAGTCTCGAAAACGGACCGTAAAATGGTCTAAT

TTTGTCGACCTCAGATCAGGCGAGACGACCCGCTGAATTTAA

>OTU_703

GAATTGCGATATGTAATGTGAATTGCAGAATTCAGTGAATCATCGAATCTTTGAACGCACATTGCACCCTCTGGTATTCC

GGAGGGTATGCCTGTTTGAGTGTCATTAATATCTCAACACAGACAGGTTTTATCACCTGTTCTGATTGCTTGGATGCTGG

AGGTTTGCTGGTGAATACTCTTTATCAGCTCCTTTTAAATGCATTAGCAGAATACAATGCAAATTAATCTGGCTTCAGTC

TGATAAAGCACTTACCTGTTtttGCTGTTGCTAGTGGAAAAGCTATTGTTTCAGCTTCTAACTGTCCATATCGTGGACTG

TCTCTTTGGAGGCATAGAATTTGACACTTGACCTCAAATCAGGTAGGACTACCCGCTGAACTTAA

>OTU_704

AAAATGCGATAAGTAATGTGAATTGCAGAATTCAGTGAATCATCGAATCTTTGAACGCACATTGCGCCCGCCAGTATTCT

GGCGGGCATGCCTGTTCGAGCGTCATTTCAACCCTCAAACCcccGGGTTTGGTGTTGGAGATCGGTCAGCTTTTCTGCTC

ACCGGCTCCGAAATCTAGTGGCGGTCTCGCTGCAGCCTCCATTGCGTAGTAGATAACACCTCGCAACTGGAACGCGGCGC

GGCCAAGCCGTTAAACCcccAACTTCTGAATGTTGACCTCGGATCAGGTAGGAATACCCGCTGAACTTAA

>OTU_705

GAAATGCGATAAGTAATGTGAATTGCAGAATTCAGTGAATCATCGAATCTTTGAACGCACATTGCGCCCGCTGGTATTCC

GGCGGGCATGCCTGTTCGAGCGTCATTATGACCAATCAAGCTCTGCTTGGCCTTGGGGCCCGCTGTACCGGCGGCCCTTA

AAATTAGTGGCGGTGCCGTCTGGCTCTAAGCGTAGTAATACTTCTCGCTACAGGGTCCAGCCGTCCACCCGCCAGAACCc

ccAACTTTCTTAGGTTGACCTCGGATCAGGTAGGGATACCCGCTGAACTTAA

>OTU_706

GAAATGCGATAAGTAATGCGAATTGCAGAATTCCAGTGAGTCATCGAATCTTTGAACGCACATTGCGCCCTTTGGTATTC

CGAAGGGCATGCCTGTTCGAGCGTCATTATCACCCCTCAAGCCCCTGGCTTGGTGTTGGACGGTTTGGTGGAGGTCCCCT

CGGgggCTCCTGCCCCTCCCAAAGACAATGACGGCGGCCTCGTTGGACCcccGGTACACTGAGTTCTTCACGGGACACAT

ATCGGACACATGGGTTTACGGGACACGGTCTGCCTCCCCTCAGGGAGAATCTTTCTAAGGTTGACCTCGGATCAGGTAGG

GATACCCGCTGAACTTAA

>OTU_707

GAAATGCGATAAGTAATGTGAATTGCAGAATTCAGTGAATCATCGAATCTTTGAACGCACATTGCGCCCTTTGGCATTCC

GAGGGGCATGCCCGTTCGAGCGTCATTACACCACTCAAGCCTGGCTTGGTATTGGGCGTCGGGTCAACCCGCGCCTCCAT

TGAAATTCGGCTGTGGTCTGAAACTCATCCCTGCGTCGTGAGAATTTATTTCCTTTCGCTCGGGTGGGCGGCCGGCACGC

CGTTAAACCATTTTATTACAGGTTGACCTCGGATCGGGTAGGGATACCCGCTGAACTTAA

>OTU_708

GAAATGCGATAACTAATGTGAATTGCAGAATTCAGTGAATCATCGAGTCTTTGAACGCACATTGCGCCcccTGGTATTCC

GGggggCATGCCTGTCCGAGCGTCATTGCTGCCCTCAAGCCCGGCTTGtgtgtTGGGTCGCCGTCCcccTCTCCGGgggA

CGGGGCCCGAAAGGCAGCGGCGGCACCGCGTCCGATCCTCGAGCGTATGGGGCTTTGTCACATGCTCTGTAGGATTGGCC

GGCGCCTGCCGACGTTTTCCAACCATTCTTTCCAGGTTGACCTCGGATTCCAGGTTAGGGATACCCGCTGAACTTAA

>OTU_709

GAAATGCGATACGTAATGTGAATTGCAGAATTCAGTGAATCATCGAGTCTTTGAACGCACATTGCGCCcccTGGTATTCC

GGggggCATGCCTGTCCGAGCGTCATTGCTGCCCTCAAGCACGGCTTGtgtgtTGGGCCcccGTCCcccTCCCAGGAAGG

GGACGGGCCCGAAAGGCAGCGGCGGCACCGTGTCCGGTCCTCGAGCGTATGGGAAGCAACTTtttGTCACCCGCTCCTGT

AGGTCCGGCCGGCGGCCTGCCCAACCCCAACCTTtttttAACCAGGTTGACCTCGGATCAGGTAGGGATACCCGCTGAAC

TTAA

>OTU_710

GAAATGCGATAAGTAATGTGAATTGCAGAATTCAGTGAATCATCGAATCTTTGAACGCACCTTGCGCCTTTTGGTATTCC

GAAAGGCATGCCTGTTTGAGTGTCATGAAATCTCAATCCCATCTGGTTTATGACCAGGTGTTGGACTTGGACATGGGTGT

CTGCCAGTCACATGGCTCACCTTAAATGACTTAGTGGGATCGCTTGCATCTGTGACAGACGTAATAAGTTTCGTCTTGTC

CTTTGCTTTCAATATCCTGCTTACAACCCTGGACCTTGTACACTTCGGTGTGCTCGGCCAAACCTTTGACTCTGACCTCA

AATCAGGTAGGACTACCCGCTGAACTTAA

>OTU_711

GAAATGCGATAAGTAATGTGAATTGCAGAATTCAGTGAATCATCGAATCTTTGAACGCACATTGCGCCCTTTGGTATTCC

GAAGGGCATGCCTGCTCGAGCGTCATTTAAACCCTCAAGCCCTGCTTGATGTTGGGTGCGGTCCTAGGACCCTCCCGTAA

CTCGTTGGCGGCACCGTCCGGCTTCAAGCGTAGCAAGATTTAAAATCCCTCGCTTCTGAAGCTTCCGGTCGGCGCCTGCC

GGTTAACCTCAACCTAAAGTTTGACCTCGGATCAGGTAGGGATACCCGCTGAACTTAA

>OTU_712

GAAATGCGATAAGTAATGTGAATTGCAGAATTCAGTGAATCATCGAATCTTTGAACGCACCTTGCGCTCCTTGGTATTCC

GAGGAGCATGCCTGTTTGAGTGTCATTAAATTCTCAACCTCCCCAACTTTGTTGTGCTTGGAGGCTTGGAAGTGGgggTT

TGCAGGTCCGCACCCGTGGTCTGCTCCTCTGAAATatatTAGTGGGTTAGTCCCCTATCTATTGGTGTGATAATTATCTA

CGCCGTGGATTGGGATGGCTGTTGAAGACCTGCTTCTAACCGTCCTTCTTGGACAATTCTTGACAATTTTGACCTCAAAT

CAGGTAAGACTACCCGCTGAACTTAA

>OTU_713

GAAATGCGATAAGTAGTGTGAATTGCAGAATTCAGTGAATCATCGAATCTTTGAACGCACATTGCGCCCCTTGGTATTCC

ATGGGGCATGCCTGTTCGAGCGTCATTTGTACCTTCAAGCTTTGCTTGGTGTTGGGTGTTTGTCTCGCCTCTGCGTGTAG

ACTCGCCTTAAAGCAATTGGCAGCCGGCGTATTGTTTTCGGAGCGCAGCACAATTTGCGCTTTGTATTCATAACGGTGGC

ATCCAAAAGTCATACTTTACACTCTTGACCTCGGATCAGGTAGGGATACCCGCTGAACTTAA

>OTU_714

GAAATGCGATAAGTAATGTGAATTGCAGAATTCAGTGAATCATCGAATCTTTGAACGCACCTTGCGCCCTTTGGTATTCC

GAAGGGCATGCCTGTTTGAGTGTCATGAAACCTCACCCCACCTGGGTTtttGCCCGGATGGCGGTGGATTGGGTGTTGCC

TTTACCGGCTCGCCTCAAAAGCATAAGCGCCTTGGATGTAATACGTTTCATCCTCTTGGGTGGCTTACAACCCCACATAT

CTCATGATCTGGCCTCAAATCAGGTAGGGCTACCCGCTGAACTTAA

>OTU_715

GAAATGCGATAAGTAATGTGAATTGCAGAATTCAGTGAATCATCGAATCTTTGAACGCACATTGCGCCCGCCGGCACTCC

GGCGGGCATGCCTGTCCGAGCGTCATTTCAACCCTCGGGCCCACCCCTCGCGGGGAACGGGCCCGGCGTTGGGGACCGGA

GGCCGCCCCGGCGGCACCCGCCcccTAAATTCAGTGGCGGTCGCGCCGCAGCCTCCCCTGCGTAGTAGCACACCTCGCAC

CGGAGAGCGGCACGGCCACGCCTCGAGACCccccAATTtttCAGGTTGACCTCGGATCAGGTAGGAATACCCGCTGAACT

TAA

>OTU_716

GAATTGCGATAAGTAATGTGAATTGCAGATACTCGTGAATCATTGAATTtttGAACGCACATTGCGCCCTTGAGCATTCT

CAAGGGCATGCCTGTTTGAGCGTCATTTCCTTCTCAAAAGATAATTtttttATTtttttGGTGTGGGCGATACTCAGGGT

TAGCTTGAAATTGGAGACTGTTTCAGTCTTtttttAATTCAACACTTAGCTTCTTTGGAGACGCTGTTCTCGCTGTGATG

TATTTATGGATTTATTCGTTtttACTTTACAAGGGAAATGGTAATGTACCTTAGGCAAAGTGTTGCTTTTAATATTCATC

AAGTTTGACCTCAAATCAGGTAGGATTACCCGCTGAACTTAA

>OTU_717

GAAATGCGATACTTGGTGTGAATTGCAGAATCCCGTGAACCATCGAGTCTTTGAACGCAAGTTGCGCCCGAAGCCACTAG

GCCGAGGGCACGCCTGCCTGGGCGTCACACGCCGTTGCCcccccccATCTACTCCTTCGGGATTGCGGgggggCGGATGA

TGGCCTCCCGTGTTCCTCGCCGcgcgGTTGGCATAAATACCAAGTCCTCGGCGACGCATGCCACGACAATCGGTGGTTGC

GAAACCTCGGTTGCCCGTCGTGTGCGTTCGTCGCGCATCGAGGGCTCGAAaaaaTGCTCGGCTCCGGCTCGGCTTTCAAC

GCGACCCCAGGTCAGGCGGGGTTACCCGCTGAATTTAA

>OTU_718

GAAATGCGATAAGTAATGTGAATTGCAGAATTCAGTGAATCATCGAATCTTTGAACGCACATTGCGCCCATTAGTATTCT

AACGGGCATGCCTGTTCGAGCGTCATTTCAACCCTCAAGCCCTGCTTGGTGTTGGGGCACTATTtttGTATAGGCCCTGT

AACATAGTGGCGGATCTGCTGTTATCCTGAGCGTAGTAATACACGTCGCTTTGGGCTTACAGTGGGCTTCTGCCGTTAAA

CCcccACTTttttAAGGTGACCTCGGATCAGGTAGGAATACCCGCTGAACTTAA

>OTU_719

GAAACGCGATAGGTAATGTGAATTGCAGAATTCAGTGAATCATCGAATCTTTGAACGCACCTTGCGCTCCTTGGTATTCC

GAGGAGCATGCCTGTTTGAGTGTCATGAATCCCTCAAATCCCAATGTTTTGTAAAATTCATTGCTTGGATTTGGTTGTGG

GCCTTCTTGCTGTTGCAATACACAGCTGGCCTAAAAGAGATTAGCTGGACTATCCGACTCTTGGTAGTTTCCACTTGATG

TGATAAAATTGCATCGGTGGGTCAAAGACTGCTATAGGAATGTGAAAGTCTGCTTCAAATTCGGCGCGAGGTTCCATCCT

CAACCCATTCATTTACATCTCTGGCCTCAAATCAGGTAGGATTACCCGCTGAACTTAA

>OTU_720

GAAATGCGATAAGTAGTGTGAATTGCAGAATTCAGTGAATCATCGAATCTTTGAACGCACATTGCGCCCCTTGGTATTCC

ATGGGGCATGCCTGTTCGAGCGTCATTTGTACCCTCAAGCTTTGCTTGGTGTTGGGTGTTTGTCCCGCGCTCTTTTGCGC

AAGACTCGCCTTAAATCAATTGGCAGCCGGCATGTTAGCCTGGAGCGCAGCACATTTTGCGCCCCTTGCTTGCTTGTGTT

GGCCcccATCAAGTCCATatatTTGCTCTTGACCTCGGATCAGGTAGGGATACCCGCTGAACTTAA

>OTU_721

AATGTCACCCGCACCAACGGACTTCACTTCCTGACGCTTGTCAGCATGCATCAGGAACATGCGGCCAACGCGTTCCTTAT

GACCCTTCGTCGTGTTCAGGATCGTATCACCAGCGTTCAGAACGCCACGGTAAACGCGGACGAAGGTCAGCGTGCCGTAC

TTGTCGGAGATGATCTTGAACGCAAGGCCAGCGAACTTGCCGTCCGGATCAACCGGAACGATCGGCAGGAAGTTCTCGTC

GACCTCTTCACCTTCCGGCGGAGCGATACGGATGCCTTCGACTTCATCCGGAGCCGGCAGGTAGTCGATAACG

>OTU_722

GAAATGCGAAAAGTAGTGTGAATTGCAGAATTCAGTGAATCATCGAATCTTTGAACGCACATTGCGCCCTTTGGTATTCC

AAAGGGCATGCCTGTTCGAGCGTCATTTGTACCTTCAAGCACTGCTTGGTGTTGGGTGTTTGTCCCCGTTTTATGCAGGG

ACTCGCCTTAAAGCTATTGGCAGCCGGCATACTGGCCTTGGAGCGCAGCACATTTTGCGCCTCTTGGCCGGGACGTTGGC

GTCCATCAAGCCTACACTTTTGCTCTTGACCTCGGATCAGGTAGGGATACCCGCTGAACTTAA

>OTU_723

GAAATGCGATAAGTAATATGAATTGCAGATATTCGTGAATCATCGAATCTTTGAACGCACATTGCGCCCTGTGGTATTCC

GCAGGGCATGCCTGTTTGAGCGTCATTTCtctctcAAACCCTTGGGTTTGGTATTGAGTGATACTCTTAGTCAGACTAAG

CGTTTGCTTGAAATGTATCGGCGTGGTGGTGGAGCAGCTACCGACGAATTCAATGTATTAGGTTCTACCAAGTCGTTGTA

TCTTCGGAGGAAAACTTCCcccAGGCTTCGCCTAACAAATCACCAAACAAGTTTGACCTCAAATCAGGTAGGATTACCCG

CTGAACTTAA

>OTU_724

GAAATGCGATAAGTAATGTGAATTGCAGAATTCAGTGAATCATCGAATCTTTGAACGCACATTGCGCCCGCCAGTATTCT

GGCGGGCATGCCTGTTCGAGCGTCATTTCAACCATCAGGCCCTAGGGCCTGTGTTGGGGCACTGCgcgcCAGTCGCGCAG

GCCCTCAAAACCAGTGGCGGGCTCGCTGTCGCACCGAGCGTAGTAATACATCTCGCTCCGGACGTGCGGCGGGCGCTTGC

CGTAAAACACCcccTTCTCAAGTTTGACCTCGGATCAGGTAGGAATACCCGCTGAACTTAA

>OTU_725

GAAATGCGATAAGTAATGTGAATTGCAGAATTCAGTGAATCATCGAATCTTTGAACGCACATTGCGCCCGCCAGTATTCT

GGCGGGCATGCCTGTTCGAGCGTCATTTCAACCCTCAAGCTCTGTCTTGCGCTTGGTGTTGGGGATCGGCCCTGCCCGCC

AGCCCGGGCGGGCCGCCcccGAAATGAATCGGCGGTCTCGTCGCAGCCTCCTCTGCGTAGTAATTCAGTTATCCTCGCAC

TTGGAGCGCGGCGCGGCCACTGCCCGTAAAACGCCCAACTTCTCAAGAGTTGACCTCGAATCAGGTAGGAATACCCGCTG

AACTTAA

>OTU_726

GAAATGCGATAAGTAATGTGAATTGCAGAATTCAGTGAATCATCGAATCTTTGAACGCACATTGCGCCCGCCAGTATTCT

GGCGGGCATGCCTGTCCGAGCGTCATTTCAACCCTCAGGCCCTGGTTGCCTGGTGTTGGGGCGCCGCGCACCCTCTGCGG

GCGCGGGCCCCGAAAGTCAGTGGCGGGCTCGCCAGGACTCCGAGCGCAGTAATTTTCtctcGCTGTGGAGCGCCTGGTGG

GTTACCAGCCGTAAAACACCCCAAATTCCAAAGGTTGACCTCGGATCAGGTAGGAATACCCGCTGAACTTAA

>OTU_727

GAAATGCGATAAGTAGTGTGAATTGCAGAATTCAGTGAATCATCGAATCTTTGAACGCACATTGCGCCCCTTGGTATTCC

TTGGGGCATGCCTGTTCGAGCGTCATTAAAACCACTCAAGCTGCTTTGCTTGGTTATGGAAGAAGAGTCTGTCTGTCAGT

CTCCCTTTTGAATTTCAATGGCGGAAGGTCCTCCGGTGCCTGGCGTAGTAATAACTGTTCGCTTTGCATCTGGATGTGTT

TCCTGCCCCAAACCcccACTTCAAGGTTGACCTCGGATCAGGTAGGGATACCCGCTGAACTTAA

>OTU_728

GAAATGCGATAAGTAATGTGAATTGCAGAATTCAGTGAATCATCGAATCTTTGAACGCACATTGCGCCcccTGGTATTCC

GGgggCATGCCTGTTCGAGCGTCATTTCACCACTCAAGCCTCGCTTGGTATTGGGCAACGCGGTCCGCCGCGTGCCTCAA

ATCGACCGGCTGGGTCTTTCTGTCCCCTAAGCGTTGTGGAAACTATTCGCTAAAGGGTGTTCGGGGAGGTACTACCGCCG

TAAAACAAACCCCATTTCTAAGGTTTGACCTCGGATCAGGTAGGGATACCCGCTGAACTTAA

>OTU_729

GAAATGCGATACTTGGTGTGAATTGCAGAATCCCGTGAACCATCGAGTCTTTGAACGCAAGTTGCGCCCGAAGCCTTTAG

GTCGAGGGCACGCCTGCCTGGGCGTCACGCATCGCGTCTCCccccACCCACCTTCAGTGGGAGGGGCGGAGGATGATGGC

CTCCCATGCCTCACCGGGTGTGGATGGCCTAAATACGGAGCCcccGGTTATGAAGTGCCGCGGCATAGGTGAGATACAAG

GCCTAGCCTAGGATACATCGGAGTCGTGCACTTtttAGCTTTTGAGGACTCGCAGGACCCTAAGTTTGTTTGCCCATTGG

GGCATCAAAACCGTTGCGACCCCAGGTCAGGCGGGGCTACCCGCTGAGTTTAA

>OTU_730

GAAATGCGATAAGTAATGTGAATTGCAGAATTCAGTGAATCATCGAATCTTTGAACGCACATTGCGCCCATTAGTATTCT

AGTGGGCATGCCTGTTCGAGCGTCATTTCAACCCTCAAGCACTGCTTGGTGTTGGGGCGTCTGCAGCTCCGGCTGCAGGC

CCTGAAaaaCAGTGGCGGGCTCGCTATAACTCCGAGCGTAGTAATatatCCTCGCTTTGGAAGTGTAGCGGTTCCCGGCC

GTTAAACCccccAATTTCTGAATGTTGACCTCGGATCAGGTAGGAATACCCGCTGAACTTAA

>OTU_731

GAAATGCGATAAGTAGTGTGAATTGCAGAATTCAGTGAATCATCGAATCTTTGAACGCACATTGCGCCTCCTGGTATTCC

GGGAGGCATGCCTGTTCGAGCGTCATTAAAATCACTCAAGCTTAGGTTTACCTATTGCTTGGTCTTGGAGATGGAAGCCA

ATTTATTGGAATCCTCTTCGAAATTCAATGGCGAAGACCCTTGCTCTCCCAAGTGTAGTAATAACTTATGTCACTGAAGG

AAGCGAGAAATCTTCTGCCGTAACCcccATATTTTCTATGATTGACCTCGGATCAGGTAGGGATACCCGCTGAACTTAA

>OTU_732

GAAATGCGATAAGTAATGTGAATTGCAGAATTCAGTGAATCATCGAATCTTTGAACGCACATTGCGCCCCTTGGTATTCC

GAGGGGCATGCCTGTTCGAGCGTCATTATAACCACTCAAGCCTGGCTTGGTATTGGGGTTTTCGCGTGTTCGCGGCCCTT

AAAATCAGTGGCGGTGCCGTCTGGCTCTAAGCGTAGTAATTTCtctcGCTATAGGGTTCCGGTGGTTCACTTGCCAAAAC

CcccccATTttttCTAGGTTGACCTCGGATCAGGTAGGGATACCCGCTGAACTTAA

>OTU_733

GAAATGCGATAAGTAATGCGAATTGCAGAATTCAGTGAGTCATCGAATCTTTGAACGCACATTGCGCCCTGTGGTATTCC

GCAGGGCATGCCTGTTCGAGCGTCATTTCAACCCTCAAGCTCCGCTTGGTGTTGGGCCCTGCCcccGCGGCCGGCCCCAA

AGACAGTGGCGGCGCCGCTTGGCCCTAAGCGTAGTACATCtctcGCTCTAGGACCCGGCGTGGCCTGCCAGAACCCCAAA

CTCATGTGGTTGACCTCGGATCAGGTAGGGATACCCGCTGAACTTAA

>OTU_734

AAAATGCGATACTTGGTGTGAATTGCAGAATCCCGTGAACCATCGAGTTtttGAACGCAAGTTGCGCCCGAAGCCATTAG

GCCGAGGGCACGTCTGCCTGGGCATCTCACAATGCGTCGCTCCccccACTCACCTTGTGGTGTCGTGTGAGGTGCGGATA

TTGGCTCCCCGTGCCCTACAGTGTGCGGTTAGCCTAAAAGCGAATCCCGACAATCGACGTCGCGGCGAGTGGTGGTTGAC

AAAACCGTTGCATGATGCCGTGTGCGCTCCGATCGCCCTCGGTGATTCCTTGACCCCAAAGGTCCATAGGAATGGTGCCT

CGTTCGAGACCCCAGGTCAGGCGGGATTACCCGCTGAGTTTAA

>OTU_735

GAAATGCGATAAGTAATGTGAATTGCAGAATTCAGTGAATCATCGAATCTTTGAACGCACCTTGCGCTCCTTGGTATTCC

GAGGAGCATGCCTGTTTGAGTGTCATTAAATTCTCAACCTTCCATGTTTTATTAACATGGTTAGGCTTGGATGTGGgggT

TGCGGGCTTCATCAATGATGTCGGCTCTCCTTAAATGCATTAGCGGAACTTTTGTGGACCGTCTATTGGTGTGATAGTTA

TCTACGCCGTGGATGTGATGCAGCTTTATGAAGTTCAGCTTCTAACCGTCCATTGACTTGGACAATTTTGACAATTTGAC

CTCAAATCAGGTAGGACTACCCGCTGAACTTAA

>OTU_736

GAAATGCGATACGTAGTGTGAATTGCAGACTCCAGTGAATCATCGAATCTTTGAACGCACATTGCGCCCACTGGTATTCC

GGTGGGCATGCCTGTCTGAGCGTCAGTATAATCTCAAGCAACTGCTTGGTATTGGAAGATGATCTGCCTAGCAGTTCCCT

TCTTAAACTGATTGGCAAGTTTACATAGCAGCAAGCGTAGTAGAAATACCATCAGCTTAGTTACTCTGTATAGCTTTGTC

AAAATCAAACTATTtttAAGGTTTGACCTCAGATCAGGTAAGGATACCccccTGAACTTAA

>OTU_737

GAAATGCGATAAGTAGTGTGAATTGCAGAATTCAGTGAATCATCGAATCTTTGAACGCACATTGCGCCCCTTGGTATTCC

ATGGGGCATGCCTGTTCGAGCGTCATTTGTACCCTCAAGCTCTGCTTGGTGTTGGGTGTTTGTCACAGGGGTGCCGTCAC

ACCCCGCCGACTCGCCTGAAAACAATTGGCAGCCAGGATTTTGGTATAGTAGCGCAGCACAATTTGCGTCTCTAGCCAAA

AGATTGGCGTCCACAAAGCTCTCGTCACCTTTTGACCTCGGATCAGGTAGGGATACCCGCTGAACTTAA

>OTU_738

GAAATGCGATAAGTAGTGTGAATTGCAGAATTCAGTGAATCATCGAATCTTTGAACGCACATTGCGCCCCTTGGTATTCC

ATGGGGCATGCCTGTTCGAGCGTCATTTGTACTCTCAAGCTCTGCTTGGTGTTGGGTGTTTGTCCTCTCCCTCGTGTTTG

GACTCGCCTTAAAATAATTGGCAGCCAGTGTTTTGGTATTGAAGCGCAGCACAAGTCGCGATTCTTAGCAAATACTTGCG

TCCACAAGCCTTtttttAACTTTTGACCTCGGATCAGGTAGGGATACCCGCTGAACTTAA

>OTU_739

GAAATGCGATAAGTAGTGTGAATTGCAGAATTCAGTGAATCATCGAATCTTTGAACGCACATTGCGCCCTTTGGTATTCC

AAAGGGCATGCCTGTTCGAGCGTCATTTGTACCCTCAAGCTTTGCTTGGTGTTGGGCGTCTTGTCtctctcCCGAGACTC

GCCTTAAAATCATTGGCAGCCGGCCTACTGGTTTCGGAGCGCAGCACATTATTTGCGCTCTTGTCCAGCCGCGGTCGcgc

gTCCATGAAGCTTCTTTCAAACTTTTGACCTCGGATCAGGTAGGGATACCCGCTGAACTTAA

>OTU_740

AAAATGCGATAAGTAATGTGAATTGCAGAATTCAGTGAATCATCGAATCTTTGAACGCACATTGCGCCCGCCAGTATTCT

GGCGGGCATGCCTGTCTGAGCGTCATTTCAACCCTCAGGACCCCGTTTCGCGGGACCTGGCGTTGGGGATCAGCCCGGAG

CCTCGCGGCCGGCGGCTGGCCCCTAAATCTAGTGGCGGTCCTCCCGACGACCTCCTCTGCGCAGTAGTTAACTCGCCTCG

CAGCTGGAAAGCGGGACGGCCACGCCGTAAAACCcccAACTCACGCAAGGTTGACCTCAGATCAGGTAGGAATACCCGCT

GAACTTAA

>OTU_741

GAAATGCGATAAGTAATGTGAATTGCAGAATTCAGTGAATCATCGAATCTTTGAACGCACATTGCGCCCGCTAGTATTCT

GGCGGGCATGCCTGTTCGAGCGTCATTTCAACCATCAAGCCTCAGGCTTGTGTTGGGGCCCTGCGGCTGCCGCAGGCCCC

TAAAAGCAGTGGCGGACTCGCTGTCATACCGAGCGCAGTAACACATCTCGCTCAGGGCGTGGCGGCGGGCACCAGCCGTA

AAACTCTTACACCTAAGGTTGACCTCGGATCAGGTAGGAATACCCGCTGAACTTAA

>OTU_742

GAAATGCGATACGTAATGTGAATTGCAGAATTCAGTGAATCATCGAATCTTTGAACGCACCTTGCGCTCCATGGTATTCC

GTGGAGCATGCCTGTTTGAGTGTCATGAATTCTTCAACCCACCTCTTTCTTAGTGAATCAGGCGGTGTTTGGATTCTGAG

CGCTGCTGGCCTCACGGCCTAGCTCGCTCGTAATGCATTAGCATCCGCAATCGAACTTCGGATTGACTCGGCGTAATAGA

CTATTCGCTGAGGATTCTGGTCTCTGACTGGAGCCGGGTAAGATTAAAGGAAGCTACTAATCCTCATGTCTATCTTGAGA

TTAGACCTCAAATCAGGTAGGACTACCCGCTGAACTTAA

>OTU_743

GAAATGCGATAAGTAGTGTGAATTGCAGAATTCAGTGAATCATCGAATCTTTGAACGCACATTGCGCCCCTTGGTATTCC

ATGGGGCATGCCTGTTCGAGCGTCATTTGTACCCTCAAGCTTTGCTTGGTGTTGGGCGTTTGTCTTGCGAGACTCGCCTT

AAAATCATTGGCAGCCGGCATACTAGTATCGGAGCGCAGCACAAGTCGCGCTTTGTACCTGCATGCGGGCGTCCAAGAAG

CCTCATTTTCCACTTTTGACCTCGGATCAGGTAGGGATACCCGCTGAACTTAA

>OTU_744

GAAATGCGATAAGTAATGTGAATTGCAGAATTCAGTGAATCATCGAATCTTTGAACGCACATTGCGCCCGGTGGTATTCC

GCCGGGCATGCCTGTTCGAGCGTCATTTCACCACTCAAGCCTGGCTTGGTATTGGGCGTCGCGGAGCTTCCCGcgcgcCT

CAAAGTCGTCCGGCCGAGTCGTCCGTCTCCGAGCGTTGTGACTTTACTGTTTCGCTCTCGAGGTCGGGTGCCGCGGCCGT

TAAACTTCTTtttttACAGGTTGACCTCGGATCAGGTAGGGATACCCGCTGAACTTAA

>OTU_745

GAAATGCGATAAGTAGTGTGAATTGCAGAATTCAGTGAATCATCGAATCTTTGAACGCACATTGCGCTCCCTGGTATTCC

GGGGAGCATGCCCGTTTCAGAATCATTAAATATCTCACTTACGACTGGGTGTTTTGCATCCAGGGTGTAGGTGGATGTTG

GATGACTTGAACATTGTTTGTTCAATCGTCTTAAAGTGATGGTGCGTGCTCCAGCTGCTGTCAGTTGGCTGTCAAGGTGC

AACAGATAATATCTATTCACTTTTGGGCTTGCTTGACGTAGCCGGACGTGCGTACTCCTGGCCACCTACGATTTGGGCTG

CAGACGCAGTTTGAATTGGTAACTTGGTCTGAAATCGGGCAAGAAAACCCGCTGAACTTAA

>OTU_746

GAAGTGCGATAAGCAATGCGAATTGCAGAACCGTGAGTCATCAGATTtttGAACGCAACTGGCGCCGGCTGGCTCTCCAG

TCGGCATGCTTGTTTCAGTGTCTTtttATCTCATCACCCGAATCTTAATGCGAgagaTATCCTTCTCTTGTTAAGCATGA

AGGCACACTGCGCTCTGCGAGCGACTTTGACTTGTTCTTAGTCGCACTCAATGCGGCCGTCACATTTCACAATGTGAACT

CATTGAGAGCTGAGGCAGTGCAGCCTTACAGCTGCCAGTGCTTAGACacacaAACTTTCTTTGTATCTGAAATCAAGCAG

GATCACCCGCTGAACTTAA

>OTU_747

CAGCTGCGAGACTTGGTGTGAATTGCAGGACACATTGAGCACTGATTtttCGAACGCGCATTGCGGTCTCGGGTACTACC

CGGGACCACGTCTGTCTGAGGGTCGGATAAACGCTTGCATAAGGAGGCTCTCGCCTCCTGATTGGTCGCTTCGGAGACTC

GCTCTCCGTCGACCCAAGTCTTGCGTGATCTGCCACCGAGAGGCGTCCCGCAGTGAGGATGCCCGGGAGTGGGAGTGGCG

GAAGTGCCTGGCTCCCTTGTGCCCGGGACGGCGGTCAGACGCGAGGAGTGGCGGACGCGGAACCGTTGGGTTCGGATCCT

TTCCGTTCCTTTAACTATGTCGACCTCAGATCAGGCGAGACGACCCGCTGAATTTAA

>OTU_748

GAAATGCGATAAGTAATGTGAATTGCAGAATTCAGTGAATCATCGAATCTTTGAACGCACATTGCGCCcccTGGTATTCC

GGgggCATGCCTGTTCGAGCGTCATTTCACCACTCAAGCCTCGCTTGGTATTGGGCAACGCGGTCCGCCGCCGTGCCCTC

AAATCGACCGGCTGGGTCTTCTGTCCCCTAAGCGTTGTGGAAACTATTCGCTAAAGGGTGCTCGGGAGGCTACGCCGTAA

AACAAACCCATTTCTCAAGGTTGACCTCGGATCAGGTAGGGATACCCGCTGAACTTAA

>OTU_749

GAAATGCGATAAGTAGTATAAATTACAGAATTCAGTAAATTATTAAATCTTTAAACGTATATTGCGCCCCTCGGTATTCC

GTAGAGCATGCCTGTTTAAGCGTTATTTACCCCTTAAGCTCTGCTTAGTGTTAGGCGTTGTTTCGCTTCGCGTAAGAACT

CGCTCTAAAGGTATTAGCAGCGGTTGTGCTACCCCTTACGTAGTATATTACGCTTCTTAAGGCGGTTGATTAGCGTTTAT

AAAGCTAATATTATATCTTAACCTTAGATTAGGTAGGGATACCCGCTAAACTTAA

>OTU_750

GAAATGCGATAAGTAATGTGAATTGCAGAATTCAGTGAATCATCGAATCTTTGAACGCACATTGCGCCCCTTGGTATTCC

GGggggCATGCCTGTTCGAGCGTCATTTCAACCCTCAAGCTCTGCTTGGTATTGAACTTCGTCAGTAATGGCGGGTTCTA

AAATCAGTGGCGGTGCCGCTGGGTCCTGAGCGTAGTAATACTTCTCGCTACTGGTCTCCAGTGTGCTTCTTGCCATAACC

CCAAATTttttCAGGTTGACCTCGGATCAGGTAGGGATACCCGCTGAACTTAA

>OTU_751

GAAATGCGATAAGTAATGTGAATTGCAGAATTCAGTGAATCATCGAATCTTTGAACGCACCTTGCGCTCCTTGGTATTCC

GAGGAGCATGCCTGTTTGAGTGTCATGAAAATATCAACCAAGACTTGGGTTtttGCTCTTGTTCTTGGCTTGGAATTGGG

TGCTTGCAATCTTTACAGATGGCTCACCTTAAAAGAATTAGCTGGATCTGTTCTGAGAATTGGTTTGACTTGGCGTAATA

AGTATTTCGCTAAGGACATCTTCGGATGGCCAATACTCTTAACGAATGTCCGCTTTCTAATCCTGTTTCCTTTGGAGACT

ATACTTTTATGATCTGGCCTCAAATCAGGTAGGACTACCCGCTGAACTTAA

>OTU_752

GAAATGCGATAAGTAGTGTGAATTGCAGAATTCAGTGAATCATCGAATCTTTGAACGCACATTGCGCCTCCTGGTATTCC

GGGAGGCATGCCTGTTCGAGCGTCATTAAATCACTCAAGCGTAGGTTTGCCTATCGCTTGGTCTTGAAGATGGAAGCTAA

GCAATTAGAATCCTCTTCGAAATCCAATGGCGAAGACCCTTGCTCTCCCAAGTGTAGTAATAACTTATTCACCGAAGGAA

GCGAGTAATCTTCTGCCGTAACCcccATATTTTCTATGATTGACCTCGGATCAGGTAGGGATACCCGCTGAACTTAA

>OTU_753

GAAATGCGATAAGTAATGTGAATTGCAGAATTCAGTGAATCATCGAATCTTTGAACGCACCTTGCGCCCATTGGTATTCC

GATGGGCATGCCTGTTTGAGTGTCTTGAAATCATCAAGCCCGTTCGGTTTCACCGCCGAACGTGGCTTGGACTTGGgggC

TTGCCGTTTCGCTGCGGCTCCCCTTAAATGCATCGGTTTCGGGTCTCTGTGGGAGTAACCCCTTGGGCGTTGTATAACCC

TCGCCGTCTGGGAACTCCTCGCGACGTCCGCGTCGTCGAAGGGCCTTGAAGGAACCTCGACCTGCTGTAGAGCGGCGGTC

AATGTTAAATCGAGATCTGACCTCAAATCAGGTAGGACTACCCGCTGAACTTAA

>OTU_754

GAAATGCGATAAGTAGTATAAATTACAGAATTTAGTAAATTATCGAATCTTTAAACGCATATTACGCCCCTTAGTATTCC

GTAGAGCATGCCTATTCGAGCGTTATTTACCCCTTAAGCTCTGCTTAGTATTAGGCGTTGTCCCGCTTCGCGTAAGGACT

CGCCCTAAAGGTATTAGCAGCGGTTATACCACCCCTTACGCAGTATATTACGCTTCTTAAGGCGGTTAATTAGCGTCTAC

AAAGCTTACATTATATCTTAACCTTAGATTAGGTAGGGATACCCGCTAAACTTAA

>OTU_755

GAAATGCGATAAGTAATGTGAATTGCAGAATTCAGTGAATCATCGAATCTTTGAACGCACATTGCGCCCTCTGGTATTCC

GGAGGGCATGCCTGTTCGAGCGTCATTTCAACCCTCAAGCATTGCTTGGTGTTGGGGCACTGCCTGTAAAAGGGCAGGCC

CTGAAATCTAGTGGCGAGCTCGCTAGGACCCCGAGCGTAGTAGTTAAACCCTCGCTTTGGAAGGCCCTGGCGTGCCCTGC

CGTTAAACCcccAACTTTTGAAAATTTGACCTCGGATCAGGTAGGAATACCCGCTGAACTTAA

>OTU_756

AAAATGCGATAAGTAATGTGAATTGCAGAATTCCGTGAATCATCAAATCTTTGAACGCACATTGCGCCTTCTGGTAATCC

GGAAGGCATGCCTGTCTGAGCGTCATTACACCATTCAAACCTTTCTTTTATTAGAAGTGGCTTGGTTTTGGAAGTAAATT

GTCAGTTTTACTACTGCGGTTTCTTCTGTAATGCATTGACGAGCGTCTACTGTTCCTTAGTTGTAGCAAGCATTTTCTCA

ACTTCAAGGTAGCAGTAGATGTCCTACTTTCTATTGTCTTGTCCTTTGGGATTAACATTTTAAAGTTTGACCTCAGATCA

GGTAGGAATACCCGCTGAACTTAA

>OTU_757

AAAATGCGATACTTGGTGTGAATTGCAGAATCCCGTGAACCATCGAGTTtttGAACGCAAGTTGCGCCCGAAGCCATTAT

GCTGAGGGCACGTCTGCCTGGGCGTCACGCACAACGTCGCCCTCCACCTCGGCCCGCAGAGGGATCGTGGGTGTTGGGTC

GGATACTGGCCTCCCGTGCGCATCGGCgcgcgGCCGGCCTAAATGCGATTCGGCATCGACGCGTGTCGCGACGATTGGTG

GTTGAAGAACTCAACTCGcgcgTCGTCGCGACGGACAGCGTCGTTCGGCTCGAACGTGCTGACCCCGACGTGCTTCGCAC

CTCGACAGCGACCCCAGGTCAGGCGGGATTACCCGCTGAGTTTAA

>OTU_758

GAAATGCGAAAAGTAGTGTGAATTGCAGAATTCAGTGAATCATCGAATCTTTGAACGCACATTGCGCCCCTTGGTATTCC

ATGGGGCATGCCTGTTCGAGCGTCATTTGTACCCTCAAGCTCTGCTTGGTGTTGGGTGTTTGTCCCGCCTTCGcgcgTGG

ACTCGCCTTAAAGTGATTGGCAGCCGGAATAATTCTGGGGAACGCAGCACAACTGCAGCCTCCATTTTACGCCGAGCGTC

CAGTAAGCCTTttttCAACGTTTGACCTCGGATCAGGTAGGGATACCCGCTGAACTTAA

>OTU_759

GAAATGCGATAAGTAGTGTGAATTGCAGAATTCAGTGAATCATCGAATCTTTATGAACGCACATTGCGCCCTTTGGTATT

CCAAAGGGCATGGCCTGTTCGAGCGTCATTTGTACCCTCAAAGCTTTGCTTGGTGTTTGGGCGTCTTGTCTCTAGCTTTG

CTGGAGACTCGACCTTAAAGTAATTGGCAGCCGCCTACTTGGTTTCGGAGCGCAGCACAAGTCGCACTctctATCAGCAA

AGGTCTAGCATCCATTAAGCCTTtttttCAACTTT

>OTU_760

AAATTGCGATAACTAGTGTGAATTGCAAATTCAGTGAATCATCGAGTCTTTGAACGCATCTTGCGCTCATTGGTATTCCA

GTGAGCACGCCTGTTTCAGTATCAAaaaCAACCCTCATTCAAAATTtttttttGAATGGTCATGAAGGAAGCTAGCAATG

GCGACCTTTTAAATTGAGTAAGGCCTGAATCTGTTCATCTAGCCTGAACTTttttttAATATAAAGGAAAGCTCTTGCGA

CTTGGACTTTGTTGGGGCCTCCCAAATAAAACTCTTTCATCTTGATCTGAAATCAGGTGGGACTACCCGCTGAACTTAA

>OTU_761

GAAATGCGATAAGTAATGTGAATTGCAGAATTCAGTGAATCATCGAATCTTTGAACGCACCTTGCGCCTTTTGGTATTCC

GAAAGGCATGCCTGTTTGAGTGTCATGAAATCTCAATCCCACCGGGTTTTACTATCCGGTGCTGGACTTGGACCTGGGCG

TCTGCCACGCAAGTGGCTCGCCTCAAATGACTTAGTGGGATCTCCTAGACATCCGCGACAGACGTAATAAGTTTCGTCTC

GTCCCTTGTCTGACCGAGCCTGCTCATAACCTGCCATCGCGCACCTTtttGACTCTGACCTCAAATCAGGTAGGACTACC

CGCTGAACTTAA

>OTU_762

GAAATGCGATAAGTAATGTGAATTGCAGAATTCAGTGAATCATCGAATCTTTGAACGCACATTGCGCCCTTTGGCATTCC

GAAGGGCATGCCTGTTCGAGCGTCATTACACCAATCACGCCTGGCGTGGTATTGGGCGACGGGGCCGTCACACGCCCCGC

GCCCCAAAGACCTTCCGGCGGGACGGACCGAATCTCAGCGTTGTGTAAACATGTCGCTGGCGAGACGGGACGGCCGGCCG

TAACCcccACTCTTAAGGTTGACCTCGGATCAGGTAGGGATACCCGCTGAACTTAA

>OTU_763

GAAATGCGATAAGTAGTGTGAATTGCAGAATTTAGTAAATCATCGAATCTTTAAACGCACATTGCGCCCCTCGGTATTCC

GTAAGGCATGCCTGTTCGAGCGTCATTTACCTCTTAAGCTCTGCTTAGTGTTGGGCGTTGTCCCGCTTCGcgcgAGGACT

CGCCCTAAAGGTATTAGCAGCGGTCGTGCCACCCCTCGCGCAGCACATTACGCTTCTTAAGGCGGTTGATTAGCGTCCAC

AAAGCTCACATCACATCTTAACCTCGGATCAGGTAGGGATACCCGCTGAACTTAA

>OTU_764

GAAATGCGATAAGTAGTGTGAATTGCAGACTTTAGTGAATCATCGAATCTTTGAACGCACATTGCGCCCCTTGGTATTCC

ATTGGGCATGCCTGTTCGAGCGTCATTTACACCCTCAAGCTCATGCTTGGTGTTGGGCGTCTGTCCGCGCTTTCTGCGCG

GACTCGCCCCAAATACATTGGCAGCGGCTTCGCCGGCTTCTTGCGCAGCACTTTGCGCTCTTGAAGGCTTTCGGCGAGCT

TGCGTCCATCAAGTAACCCCTCATTTTGACCTCGAATCAGGTAGGGATACCCGCTGAACTTAA

>OTU_765

GAAATGCGATAAGTAATGTGAATTGCAGAATTCAGTGAATCATCGAATCTTTGAACGCACATTGCGCCcccTGGTATTCC

GGggggCATGCCTGTCCGAGCGTCATTACACCAATCAAGCCTGGCTTGGTATTGGGCGTCGCGGGTCTGACCCGcgcgcC

TTAAAGTCTCACCGGCTGAGCGGCGTCGTCTCTAAGCGTTGTGGAAACTATTCGCGGAGAGGAGGCGCGGCGTGGCCGTT

AAATACCCCATCAAAGGTTGACCTCGGATCAGGTAGGGATACCCGCTGAACTTAA

>OTU_766

GAAATGCGATAAGTAATGTGAATTGCAGAATTCAGTGAATCATCGAATCTTTGAACGCACATTGCGCCCATTAGTATTCT

AGTGGGCATGCCTGTTCGAGCGTCATTTCGACCCTCAAGCCTCAGTTGCTTGGTTTTGGGAGCCTATCTTCGGATAGCTC

CTCAAATAGAGTGGCGGAGTCGTGCTGACCCCAAGCGTAGTAATTttttCTCGCTTCAGGTGCCGGTGCTGACGTCCTGC

CGTGAAACCcccTATTTTAAAAGGTTGACCTCGGATCAGGTAGGAATACCCGCTGAACTTAA

>OTU_767

GAAATGCGATAAGTAATGTGAATTGCAGAATTCAGTGAATCATCGAATCTTTGAACGCACCTTGCGCCCTTTGGTATTCC

GAAGGGCATGCCTGTTTGAGTGTCATGAAACCTCACCCCACTTGGGTTtttGCCCGAGCGGTGGTGGATTGGGCGTCTGC

CGTCAATGGCTCGCCTGAAAAGCATAAGCAACTTTGGATGTAATACGTTTCATCCTTTCGAGTGGCTGACAACCCACATA

TCTCATGATCTGGCCTCAAATCAGGTAGGGCTACCCGCTGAACTTAA

>OTU_768

GAAATGCGATAAGTAATGTGAATTGCAGAATTCAGTGAATCATCGAATCTTTGAACGCACCTTGCGCCCTTTGGTATTCC

GAAGGGCATGCCTGTTTGAGTGTCATGAAACCTCACCCCACTTGGGTTtttGCCTGAGCGGTGGTGTATTGGGTGTTGCC

TTGCCAAAGCTCGCCTTAAAACATAAGCACCTTGGATGTAATACGTTTCATCCTTCTTGGGCTGGCTGGATAACCCCACC

ATATTCATTGATCTGGCCTCCAAATCAAGGTAGGGCTACCCGCTGAACTTAA

>OTU_769

GAAATGCGATAAGTAGTGTGAATTGCAGACTTTAGTGAATCATCGAATCTTTGAACGCACATTGCGCCCCTTGGTATTCC

ATTGGGCATGCCTGTTCGAGCGTCATTTAACCCCTCAAGCACTGCTTGGTGTTGGGCGTTTTGTCTACGGACTCGCCCCA

AATCTATTGGCAGCAGCCTTACCAGCTTCTTGCGCAGTACAATGCGCTTCGAGAAGCCTGTGTAGTGGTGTGCAATCCAG

TAAGCAACTCTTTATTTTGACCTCGAATCAGGTAGGGATACCCGCTGAACTTAA

>OTU_770

GAAATGCGATAAGTAATGTGAATTGCAGAATTCAGTGAATCATCGAATCTTTGAACGCAACTTGCGCTCCTTGGTATTCC

GAGGAGCATGCCTGTTTGAGTGTCATTTGAAACCTCTCAACCACATCAAACGGTGTGGCTTGGAAATGGGAGGAAGTGCA

GGCCAACAGAGGTCTGCTCTCCTGAAATGCATTAGCGGTGTCTAGGCAGACTTTACCACAGGTGTGATAACCATTCAATG

CCTTGGTACTGCAGATTGCTGCCGCTTCCAATGACCTTCTTTATGACCAACTTGACCTCAAATCAGGTAGGATTACCCGC

TGAACTTAA

>OTU_771

GAAATGCGATACGTAATGTGAATTGCAGAATTCAGTGAATCATCGAATCTTTGAACGCATATTGCGCTctctGGTATTCC

GGAGAGCATGCTTGTTTGAGTATCAGTAAACACCTCAACTCCCTTTTCTTtttttGAAATGGGAGCTGGACTTGAGTAAT

CCCAACGCTTTTCTCACCGAGAAGTGGCGGGTTACTTGAAATGCAGGTGCAGCTGGACTTTTCTCTGAGCTAAAAGCATA

TCTATTTAGTCTGCCTAAaaaaCAGATTATTACCTTTGCTGCAGCTAACATAAAGGAGACTGGTTCTTGTGCTGACTGAT

GCAGGATTCACAGAGACGGCTTCGGCTGGCTTTGTAAACTCGATCTCAAATCAAGTAAGACTACCCGCTGAACTTAA

>OTU_772

GAAATGCGATAAGTAATGTGAATTGCAGAATTCAGTGAATCATCGAATCTTTGAACGCACATTGCGCCCATTAGTATTCT

AGTGGGCATGCCTGTTCGAGCGTCATTTCAACCCCTAAGCACAGCTTATTGTTGGGAACCTACGGCTTCGTAGTTCCTCA

AAGACATTGGCGGAGTGGCAGTGGTCCTCTGAGCGTAGTAATCTTTTATCTCGCTTCTGTTAGGTGCTGCCcccccGGCC

GTAAAACCCCAATTttttCTGGTTGACCTCGGATCAGGTAGGAATACCCGCTGAACTTAA

>OTU_773

GAAATGCGATAAGTAGTGTGAATTGCAGAATTCAGTGAATCATCGAATCTTTGAACGCACATTGCGCCTTTGGTATTCCA

AAAGGGCATGCCTGTTCGAGCGTCATTTGTACCCTAAGCTTTGCTGGTGTTGGGCGTCTTGTCTCTAGCTTTGCTGGAGA

CTCGCCTTAAAGTAATTGGCAGCCGCCTACTGGTTTCGGAGCGCAGCACAAGTCGCACTctctATCAGCAAAGGTCTAGC

ATCCATTAAGCCTTttttttCAACTTTTGACCTCGGATCAGGTAGGGATACCCGCTGAACTTAA

>OTU_774

GAAATGCGATAAGTAATGTGAATTGCAGAATTCAGTGAATCATCGAATCTTTGAACGCACCTTGCGCCTTTTGGTATTCC

GAAAGGCATGCCTGTTTGAGTGTCATGAAATCTCAACCCCTCTGGTTTTCTGAACTGGTTGGGCTGGACTTGGGTGATCT

GCCAGCAATGGCTCGCCTCAAATGACTTAGTGGAACATCCCGCATCAGTGTTAGACGTAATAAGTTTCGTCtctcCTTGT

GGCTGATGACTGCTTACAACCTGCCATCGCGCACTTTTGACTTTGACCTCAAATCAGGTAGGGCTACCCGCTGAACTTAA

>OTU_775

GAAATGCGATAAGTAATGTGAATTGCAGAATTCAGTGAATCATCGAATCTTTGAACGCACATTGCGCCcccTGGTATTCC

GGggggCATGCCTGTTCGAGCGTCATTTCACCACTCAAGCCTCGCTTGGTATTGGGCAACGCGGTCCGCCGCGTGCCTCA

AATCGACCGGCTGGGTCTTCTGTCCCCTAAGCGTTGTGGAAACTATTCGCTAAAGGGTGCTCGGGAGGCTACGCCGTAAA

ACAAACCCATTTCTAAGGTTGACCTCGGATCAGGTAGGGACTACCCGCTAACTTAA

>OTU_776

GAAATGCGATAAGTAGTGTGAATTGCAGAATTACCTGAATCATCGAATCTTTGAACGCACATTGCGCCcccTGGTATTCC

TGGgggCATGCCTGTTCGAGCGTCATTAACAACCAATCGGGCGGGGCTTTGCATGCTCCCGCTCGGCCATGGgggATGGA

CGTCGTCGGACGCCCACCcccGAAATGCAACGGCGGACGGCCACCCGTGGCACTGCGGCGTAGTAAGTTAACCCTTCGcg

cgcgcCCACGGAGTGGACTGCCCGGCCGCTTGAACCcccccACATCCTACGGTTGACCTCGGATCAGGTAGGGATACCCG

CTGAACTTAA

>OTU_777

GAAATGCGATAAGTAGTGTGAATTGCAGAATTCAGTGAATCATCGAATCTTTGAACGCACATTGCGCCCCTTGGTATTCC

ATGGGGCATGCCTGTTCGAGCGTCATCTACACCCTCAAGCTCTGCTTGGTGTTGGGCGTCTGTCCCGCCTCCGcgcgTTG

GACTCGCCCCAAATTCATTGGCAGCGTTTCCTTGCCcccTCTCGCGCAGCACATCGCGCTTCTCGAGGGCGGCTCCGGGA

CGCGACCCACGAAAGATGACCACCGTCTTTGACCTCGGATCAGGTAGGGATACCCGCTGAACTTAA

>OTU_778

GAAATGCGATAAGTAATGTGAATTGCAGAATTCAGTGAATCATCGAATCTTTGAACGCACCTTGCGCCTTTTGGTATTCC

GAAAGGCATGCCTGTTTGAGTGTCATGAACTCTCAACCcccTCGGGTTTCCGATCGGGTTGGACTTGGGTGCTGCCATCA

TGGCTCACCTTAAATGACTTAGTGGATGTCTCCcccAGTCAATGTAATAAGTTTCATTGGCGAAGGGTTCAGTCTGCTCA

CAACCGCCTTTACTGGCAACATTTGTACTCTGACCTCAAATCAGGTAGGGCTACCCGCTGAACTTAA

>OTU_779

GAAATGCGATAAGTAGTGTAAATTACAGAATTTAGTGAATTATCGAATCTTTAAACGCATATTGCGCCCCTTAGTATTCC

GTAGGGCATGCCTATTCAAGCGTCATTTACCCCTTAAGCTCTGCTTAGTGTTGGGCGTTATCCCGCTTCGCGTAAGAACT

CGCCCTAAAGGTATTGGCAGCGGTCGTGCCACCCCTCGCGCAGTATATTGCGCTTCTTAAGGCGGTTAATTAGCGTCTAT

AAAGCTTATATTATATCTTAACCTTGGATCAGGTAGGAATACCCGCTGAACTTAA

>OTU_780

GAAATGCGATAAGTAATGTGAATTGCAGAATTCAGTGAATCATCGAATCTTTGAACGCACATTGCGCCCGCCAGCATTCT

GGCGGGCATGCCTGTTCGAGCGTCATTTCAACCCTCGAGCTCGTCTTCATTGACGGGATCGGTGTTGGGACCCGGCGAGC

GGGGACTTTTGTCCTCTGCCGGCCCCGAAATTCAGTGGCGGCCCGTTGCGGCGACCTCTGCGTAGTAACTCAACCTCGCA

CCGGTAACAGCATCGTGGCCACGCCGTAAAACCccccGACTTTTATAAGGTTGACCTCGAATCAGGTAGGACTACCCGCT

GAACTTAA

>OTU_781

GAAATGCGATAAGTAATGTGAATTGCAGAATTCCGTGAATCATCGAGTCTTTGAACGCACATTGCGCTCCCTGGTATTCC

GGGGAGCATGCCTGTCCGAGCGTCATTTTCAACCCTCAAGCCTGGCTTGtgtgATGGGCCAGCGTTCCCACGACCTTCTG

GGACGGGGACGGGCCCGAAATGCAGTGGTGGTGTCTAGGTGAACGACCCGAGTGTATGGGGCTCTGTCACACGCTCGCGG

TATGCCTCTCGACGCCCGCCTTTGAACCCTCTTTCTAAGGTTGACCCCGGATCAGGTAGGGATACCCGCTGAACTTAA

>OTU_782

GAAATGCGATACGTAATGTGAATTGCAGGTTTTCGTGAATCATCGAATCTTTGAACGCATATTGCGCCCTTCGGTATTCC

GAAGGGCATGCCTGTTTGAGCGTCATTTCtctctcAAACCTCGGGTTTGGTATTGAGTGATACTctctTTCTGGGTTAAC

TTGAAATACTGTGGCAAGAGCTGCTTTGCAGGATCTCCTCTGAACTAACGTTTCTAGGTTCTACCAATTCGTTATGGACA

GTgtgtgCTGAACGCATGGCTTTTGGGCTCGGCCTAACAATTTCTTCATAAGTTTGACCTCAAATCAGGTAGGATTACCC

GCTGAACTTAA

>OTU_783

GAAATGCGATAAGTAGTGTGAATTGCAGAATTCAGTGAATCATCGAATCTTTGAACGCACATTGCGCCCCTTGGTATTCC

ATGGGGCATGCCTGTTCGAGCGTCATTTGTACCCTCAAGCTTTGCTTGGTGTTGGGCGCCTGTCTTTCCTTTGGGGAGAC

TCGCCTTAAAACAATTGGCAGCCGGCATAGGAGCCTGGAGCGCAGCACAATTTGCGCCTCTTGCTTTGATTGTTGGCACC

CATCAAGACCTTtttATCAGCTCTTGACCTCGGATCAGGTAGGGATACCCGCTGAACTTAA

>OTU_784

GAAATGCGATAAGTAGTGTGAATTGCAGAATTCAGTGAATCATCGAATCTTTGAACGCACATTGCGCCCCTTGGTATTCC

ATGGGGCATGCCTGTTCGAGCGTCATTTGTACCTTCAAGCCTTGCTTGGTGTTGGGTGTTTGTCCTTGCCACTGTGCCTG

GACTCGCCTTAAAAGAATTGGCAGCCAGTGTTTTGGTGTAGAAGCGCAGCACAATTTGCGACTCTGCTCTCAGCACTTGC

ATCCACAAGCCTCTTATCACGTTTGACCTCGGATCAGGTAGGGATACCCGCTGAACTTAA

>OTU_785

GAAATGCGATAAGTAATGTGAATTGCAGAATTCAGTGAATCATCGAATCTTTGAACGCACATTGCGCCCCGTGGTATTCC

GCGGGGCATGCCTGTTCGAGCGTCATTTCACCACTCAAGCCTGGCTTGGTATTGGGCGCCGCGTGTCCTCGACACGcgcg

cCTCAAAGTCTTCCCGGCTGCGTCGTCCGTCTCCCAGCGTTGTGACTTCACGTTTTCGCTTCGGAGCGCGGGCgcgcgcC

GCTAATCTTTCATTCAGGTTGACCTCGGATCAGGTAGGGATACCCGCTGAACTTAA

>OTU_786

GAAACGCGATAGGTAATGTGAATTGCAGAATTCAGTGAATCATCGAATCTTTGAACGCACCTTGCGCTCCTTGGTATTCC

GAGGAGCATGCCTGTTTGAGTGTCATGAATACCTCAAATCCCAATGTTTTGTAAAATTCATTGTTTGGATTTGGTTGTGG

GCTTTTGCTATTTAACTATAGCTAGCCTAAAAGAGATTAGCTGGACTTTCAACTCTTAAGATAGTTACCACTTGATGTGA

TAAAATTACATTGGTGGGTCAATGACTATAGTAAGGATCGAGAAGTCTGCTTCGAATCCGGCGCGAGGTTCCATCCTCAA

CCCATTTAATTTACATCTCTGGCCTCAAATCAGGTAGGATTACCCGCTGAACTTAA

>OTU_787

GAAATGCGATAAGTAGTGTGAATTGCAGAATTCAGTGAATCATCGAATCTTTGAACGCACATTGCGCCCCTTGGTATTCC

ATGGGGCATGCCTGTTCGAGCGTCATTTGTACTCTCAAGCCTTGCTTGGTGTTGGGTGTTTGTCTTCGCCTCGCTTAGAC

TCGCCTTAAATatatTGGCAGCCGGTATACTGGTTTGAAGCGCAGCACAATTGCATTTCAGGCTCTTAGTACTAGCATCC

ACAAAGATTATCAACACTTTTGACCTCGGATCAGGTAGGGATACCCGCTGAACTTAA

>OTU_788

GAAATGCGATAAGTAGTGTGAATTGCAGAATTCAGTGAATCATCGAATCTTTGAACGCACATTGCGCCCTTCGGTATTCC

GTTGGGCATGCCTGTTCGAGCGTCATTTAAACATTCAAGCTCAGCTTGGTGTTGGGTGCTTGTCCTCCcccGCGGTGGAC

TCACCTCAAATGCATTGGCGGCCGGTATGTTGGCTTCGAGCGCAGTAGAAACGCGCCAGACGTCCTGACATGCTGGTCCc

ccACAAGACCTTCTTTTATCTTGACCTCGGATCAGGTAGGGATACCCGCTGAACTTAA

>OTU_789

GAAATGCGATAAGTAGTGTGAATTGCAGAATTCAGTGAATCATCGAATCTTTGAACGCACATTGCGCCCCTTGGTATTCT

ATGGGGCATGCCTGTTCGAGCGTTATTTGTACCCTCAAGCTCTGCTTAGTGTTGGGTGTTTGTCTACTCCTTGGTAGTTG

GACTCGCCTTAAAACAATTGGTAGCCAGTGTTTTGGTATTAAAGCGCAGCACATTTTGCGGTTCTGGCCCTGAACACTGG

CATCTAGTAAGCTCTTTTCTACTTTTAACCTTGGATCAGGTAGGGATACCCGCTGAACTTAA

>OTU_790

AAAATGCGATAGCTGGTGTGAATTGCAGAATCCCGTGAACCATCGAGTCTTTGAACGCAAGTTGCGCCTGAAGCCGTCAG

GCCGAGGGCACGCCTGCCTGGGTGTCACCCTCTCCCTTGCCCAAACACAAGCCTCCTCCCACCGAAaaaTGGGATGAGTG

ATTTGGGCGGATAAATGGCCTCCCGTGGCGAATGTTTTCGTGGTTGGCCCAAAATTAGCACTTGGCTCTTAGGAAGCCAC

GACAAGCATTGGCGAAGTACTTCGGTGATCGTTGTTGTCGTGCTAGACTTTGTAGCGTGCTGTAGAATACCTCGGTGCTT

GCATCCTAAGATTGCGACCCCAGGTCAGGCGGGAACACCTGCTGAGTTTAA

>OTU_791

AAAATGCGATAAGTAGTGTGAATTGCAGAATACGTGAATCATCGAATCTTCGAACGCATATTGCACTTttttAGTTTACT

AAaaaaGTATGTCTGTTTGAGTATCAGTAAAATATTCTCATAAaaaCTTttttGTTtttATGGTGATGAGTGTTTTACAA

CGATACAAGTTGTTTCACTTTAAATTttttGATTATTGAAATTttttCTAATTTTAATATAAAATTGGATAAAATTTTCC

CGTAAaaaGGAATAACCTAAACAATTTTGTATTtttttAGAAaaaaTCCAACAAAGGAAATTTAATTAAATAACTTGATC

TCAAATCAGATAAGAGTACCCGCTGAACTTAA

>OTU_792

GAAATGCGATAAGTAATGTGAATTGCAGAATTCAGTGAATCATCGAATCTTTGAACGCACATTGCGCCCCTTGGTATTCC

ATGGGGCATGCCTGTTCGAGCGTCATTTGTACCCTCAAGCTCTGCTTGGTGTTGGGTGTTTGTCCACTCCCTAGTGTTTG

GACTCGCCTCAAAGCAATTGGCAGCCAGTGTTTTGGTATTGAAGCGCAGCACATTTTGCGATTCTAGCCTAGAATACTAG

CGTCCAGTAAGCCTTtttCCACTTTTGACCTCGGATCAGGTAGGGATACCCGCTGAACTTAA

>OTU_793

GAAATGCGATAAGTAGTGTGAATTGCAGAATTCAGTGAATCATCGAATCTTTGAACGCACATTGCGCCTTCCGGTATTCC

GGGAGGCATGCCTGTCCGAACGTCATCAAGCCCCTCGCCCGCTTCTTTTGCTTGCAAAAGTGGCAAGGGCGGCACATGGC

GAATGGCTGCCTCAGAGCGGCCATCGCCGAAATCCATTGGCAGACCCTCCATGGCTGTGATTGTGATAAGATTTTACTTA

CAATTGCCAGACTGGTAGGCGCCTTGCCGAAAGCAGCCGGGCGTCCTCCACGGGACCGAACGCTATACCACATCTTTTGA

CTTCGGATCAGGTAGGGATACCCGCTGAACTTAA

>OTU_794

GAAATGCGATAAGTAATGTGAATTGCAGAATTCAGTGAATCATCGAATCTTTGAACGCACATTGCGCCCCTTGGTATTCC

GGgggCATGCCTGTTCGAGCGTCATTATCACCCCTCAAGCTCAGCTTGGTGTTGGGGCCTGCCTGTCACAGGGCAGCCCT

TAAAAGCAGTGGCGGTGCCATCTGGCTCTACGCGTAGTAATACTTTTCGCGACAGAGTCCCGGTGGTGTCTTGCCAGAAC

CccccATATTtttAATGAATTGACCTCGGATCAGGTAGGGATACCCGCTGAACTTAA

>OTU_795

AACAAGACACATGTCGACTTCAAGGCGCTCAGGGAATGGGGTTCCATCGGCCGCAGCAGCTTGTTCGTTTCTTACGATGC

CTGGAAGAATGCACGTAGCAACCCCTATACGCTCGCAGCCTATGAAACCGCAGCAAAGGCGAACAATAATTTCTCCGTAG

GAAACTACGCTTCCAGCTATATCCCTGGAGTGACGACCAACTACTGGAAGAACTACCTTTATCACCGCAACAGCGTTCTG

ATTTCATTCCAGAATGAGTTCTCGCTTGCGCGTCACCTGAACCT

>OTU_796

GAAATGCGATAAGTAATGTGAATTGCAGAATTCAGTGAATCATCGAATCTTTGAACGCACCTTGCGCCCTTTGGTATTCC

GAAGGGCATGCCTGTTTGAGTGTCATTAAATTATCAACCTTGCTCGCTTTTACCGGCTTGAGTTAGGCTTGGATGTGAGG

GCTTGCTGGCTTCCTTTAGTGGATGGTCTGCTCCCTTTAAATGCATTAGTGGGATCTCTTGTGGACCGTCACTTGGTGTG

ATAATTATCTATGCCACTTGACCTTGAAGCAAAACTTATGGGAACCCGCTCATAACCGTCTTCGGACAACTTTTGACATT

TTGACCTCAAATCAGGTAGGACTACCCGCTGAACTTAA

>OTU_797

GAAATGCGATAAGTAATGTGAATTGCAGAATTCAGTGAATCATCGAATCTTTGAACGCACATTGCGCCCGCCAGTATTCT

GGCGGGCATGCCTGTTCGAGCGTCATTTCAACCATCAAGCCCCAGCGCTTGTGTTGGGGACCCGCGGCTCGTCCGCGGAC

CCTTAAAGACAGTGGCGGTTCTCGCTGCAGCACCGAGCGTAGTAGCATACACCTCGCCCTGGGAGCTGCGCGTGCGCCCG

CCGTAAAACCccccACCTCTCACAAGGTTGACCTCGGATCAGGTAGGAATACCCGCTGAACTTAA

>OTU_798

GAAACGCGATATGTAATGTGAATTGCAGAATTCAGTGAATCATCGAATCTTTGAACGCACATTGCGCCTTCCAGTATTCT

GGGAGGCATGCCTGTCCGAGCGTCGTTTCAACCCTCAAGCCCTCGTGGTTTGGTGTTGGAGTCCTACAGTCTTGTAGTCT

CCCAAACCAGTGGCGGACCGTCGCGGCCCTTCCTTTGCGTAGTAGCATTCGCCTCGCATCGGGAGCCCACGGGACTTTCT

GGCCTCTAAACCccccACAAGTCCGCTCCGGCGGCACAAGGTTGACCTCGGATCAGGTAGGAATACCCGCTGAACTTAA

>OTU_799

GAAATGCGATAAGTAATGTGAATTGCAGAATTCAGTGAATCATCGAATCTTTGAACGCACATTGCGCCCGCCGGCACTCC

GGCGGGCATGCCTGTCCGAGCGTCATTTCAACCCTCAGGGCCcccTTTCGGgggCGGCACCTGGTTCTGGGGATCAGCGG

CCCTTCGGGGCCCCTGTCCcccAAATTGAGTGGCGGTCGCGCCGCAGCCTCCCCTGCGTAGTAGCACACCTCGCACCGGA

GAGCGGCTCGGCCACGCCGTAAAACCcccAATTtttACAGGTTGACCTCGGATCAGGTAGGAATACCCGCTGAACTTAA

>OTU_800

GAAATGCGATAAGTAATGTGAATTGCAGAATTCAGTGAATCATCGAATCTTTGAACGCACCTTGCGCTCCTTGGTATTCC

GAGGAGCATGCCTGTTTGAGTGTCATTAAATTCTCAACCTTACCATCTTTGATGACAAGGTTTGGACTTGGgggTTTCAT

TTTGCAGGCTTCAACCGGAGTCGGCTCCCCTTAAATGAATTAGCCGGAAAACCCTTGTGGCCCCGTCTATTGGTGTGATA

ATTATCTACGCCGCGGGATAGGACTGCACTTTGTAATGGGATTTCTGCTTCCAGCTGTCCTTTTGGGACAATCATTGACT

ATTTGACCTCAAATCAGGTAGGACTACCCGCTGAACTTAA

>OTU_801

AAAATGCGATAAGTAATGTGAATTGCAGAATTCAGTGAATCATCGAATCTTTGAACGCACATTGCGCCCGCCAGTATTCT

GGCGGGCATGCCTGTCTGAGCGTCATTTCAACCCTCAGGACCCGTTCGCGGGACCTGGCGTTGGGGATCAGCCTGCCCCT

GGCGGCGCCGGCCCTGCAATCCACGTGGCGGTTCCCTCGCGAACTCCTCCGTGCAGTAATTAAACCTCTCGCGGCAGGAT

AGCGGTTGAACCACGCCGTTAAACCccccACTTCTCAAGGTTGACCTCAGATCAGGTAGGAATACCCGCTGAACTTAA

>OTU_802

GAAATGCGATAAGTAATGTGAATTGCAGAATTCAGTGAATCATCGAATCTTTGAACGCACATTGCGCCCGCTAGTACTCT

AGCGGGCATGCCTGTTCGAGCGTCATTTCAACCCTCAAGCCCTGCTTGGTGTTGGGGCCCTACGGCTGCCGTAGGCCCTG

AAAGGAAGTGGCGGGCTCGCTACAACTCCGAGCGTAGTAATTCATTATCTCGCTAGGGACGTTGCGGCgcgcTCCTGCCG

TTAAAGACCATCTTTAACTCAAGGTTGACCTCGGATCAGGTAGGAATACCCGCTGAACTTAA

>OTU_803

GAAGTGCGATAAGCAATGCGAATTGCAGAATTCCGCGAATCATCAGATCTTTGAACGCAAATGGCGCCAGAGGGATATCC

TTCCGGCATGTCTGTTTCAGTGTCTGAGAGGTTTCACACTTATCTTAATGTGATTGACTTGTTGGTGAGCAGAGTCTTTA

TTGACTTCGCTctctcAATCGTCtctcATGAAAGATGCGCGAGTGGATCTTTCGGTCCAATGAAGTGGTCACATCACTCG

CATGTGATTTCATTGGAAGCACCGAATTATCCGCATCCTCGCGGTTCCACAGCACTACTCACACTAGTGCTACCCTCCAT

ACCCTGCATCTGAGACAGGCAAGATCACCCGCTGAACTTAA

>OTU_804

GAAATGCGATAAGTAATGTGAATTGCAGAATTCAGTGAATCATCGAATCTTTGAACGCATATTGCGCCCAGTAGTATTCT

ACTGGGCATGCCTATTCGAGCGTCATTTCAACCCTTACGCCCTGTAGCGTAGTGTTAGGACTCTACTCTTTCGAGAGCAG

TTCCCTAAAACCAGTGGCAGTGTTCGGTACACTCATAGCGTAGTAATTCTTCTCGCTTCTGCAGTGGCCCGTACTATTCG

CCGTAAAACCCCTAATTTTCTAATGGTTGACCTCGGATTAGGTAGGAATACCCGCTGAACTTAA

>OTU_805

GAAATGCGATAAGTAATGTGAATTGCAGAATTCCGTGAATCATCGAATCTTTGAACGCACATTGCGCCCCTGGCATTCCG

GggggCATGCCTGTCCGAGCGTCATTTCTGCCCTCAAGCGCGGCTTGtgtgtTGGGTGCGGTCCccccGGGGACCTGCCC

GAAAGGCAGCGGCGACGTCCGTTGGGTCCTCGAGCGTATGGGGCTTTGTCACTCGCTCGGGGACGGACCCGCGGgggTTG

GTCACCACCAAAATTTTACCACGGTTGACCTCGGATCAGGTAGGAGTTACCCGCTGAACTTAA

>OTU_806

GAAATGCGATACTTGGTGTGAATTGCAGAATCCCGTGAACCATCGAGTTtttGAACGCAAGTTGCGCCCGAAGCCTTTGG

CCAGGGCACGTCTGCCTGGGCGTCACGCATTGCGTCTCCcccAACCCGCCTAGATGTGGGAGGGGCGAGGAGGATGGTCT

CCCATGCCTCACCGGGCGTGGATGGCCTAAAACAGGAGCCCACGGTTGCGAGCTGCTGCGGCGATTGGTGGTGTGCAAGG

CCTAGCCTAGAATGCAATCGCGTCGCACAGTGCGTGGACCTTGTGGCCTTGAGGACCCTAGAGTGTTGCCCGAGGGCGAC

CAACCACTGCGACCCCAGGTCAGGCGGGACTACCCGCTGAGTTTAA

>OTU_807

GAAATGCGATACCTAGTGTGAATTGCAGCCATCGTGAATCATCGAGTTCTTGAACGCACATTGCGCCCGTCGGTATTCCG

GCGGGCATGCCTGTCTGAGCGTCGTTTTCCTTCTTGAAGCTTtttttttAAAAGAATTCAGAATTGGCCGTGCCACTGGC

CCGGCCGAAAAGAAACGTTGCGGACGAAGCGAACTACATCGGGACGCTTTGGCCGCCGAGCGAAAATATCATTGAGCTCG

ACCTCAGATCAGGTAGGAGTACCCGCTGAACTTAA

>OTU_808

AAAATGCGATAAGTAGTGTAAATTACAGAATTTAGTAAATTATTAAATCTTTAAACGCACATTGCGCCCCTCGGTATTCC

GTAGGGCATGCCTGTTCAAGCGTCATTTACCCCTTAAGCTCTACTTAGTATTAGGCGTTATCCCACTTCGcgcgAGGACT

TGCTCTAAAGGTATTAGCAGCGGTCGTGCCACCTCTCGCGCAGTATATTACGCTTCTTAAGGCGGTTAATTAGCGTCTAC

AAAGCTTACATTATATCTTAACCTTAGATTAGGTAGGAATACCCGCTAAACTTAA

>OTU_809

GAAATGCGATAAGTAATGTGAATTGCAGAATTCAGTGAATCATCGAATCTTTGAACGCAACTTGCGCTctctGGTATTCC

GGAGAGCATGCCTGTTTGAGTATCATGAAATCTCAACCATTAGGGTTTCTTAATGGCTTGGATTTGGGCGCTGCCACTTG

CCTGGCTCGCCTTAAAAGAGTTAGCGTGTTTAACTTGTCGATCTGGCGTAATAAGTTTCGCTGGTGTAGACTTGAGAAGT

GCGCTTCTAATCGTCTTCGGACAATTCTTGAACTCTGGTCTCAAATCAGGTAGGGCTACCCGCTGAACTTAA

>OTU_810

GAAATGCGATAAGTAATGTGAATTGCAGAATTCAGTGAATCATCGAATCTTTGAACGCACATTGCGCCCGCCAGCACTCT

GGCGGGCATGCCTGTTCGAGCGTCATTCAAACCCTCAGGCCCTGCCTGGCGTTGGGGCCCTGCgcgcgcAGGCCCTCAAA

GACAGCGGCGGGTGCGCCCGGGACCGAACGTAGTCATTCTTCTCGTTCTGGGACCGCGGCgcgcTCCGGCCGTTAAACCc

ccAACTTACAGTTCGTTGACCTCGGATCAGGTAGGAATACCCGCTGAACTTAA

>OTU_811

GAAATGCGATAAGTAATGTGAATTGCAGAATTCAGTGAATCATCGAATCTTTGAACGCACATTGCGCCCTTTGGTATTCC

GAAGGGCATGCCTGTTCGAGCGTCATTTCAAACCTCAAGCTCACGCTTGGTATTGGGCGTCGTCCGCTAGGACGTGCCAG

AAACCCGTAGGCAGTGGTGCCCGCCTCCCAGCGTAGTCAATCTACCCGCTTCGGAGCgcgcgGTGCCGCCCGCCGGACGA

ACAACCACTTttttCTATGGTTTGACCTCGGATCAGGTAGGGATACCCGCTGAACTTAA

>OTU_812

GAAATGCGGTAAGTAATGTGAATTGCAGAATTCAGCGAACCATCGAATCTTTGAACGCACATTGCGCCCATTAGTATTCT

ATTGGGCATGCCTGTTCGAGCGTCATTTCAACCCTCAGGCTTTGGTCTGGTGCTGGGGCATTGCACTGCAAGCCCTGAAT

AATAGTGGCGGACTCACTGAAATCCGAGCGTAGTAACTTtttCTTGCTATGGACTTTCAATGATGTACCTGCCGTAAaaa

GCCcccTATATTACAAGGTTGACCTCGGATCAGGTAGGAATACCCGCTGAACTTAA

>OTU_813

GAAATGCGATAAGTATTGTGAATTGCAGATTTTCGTGAATCATCGAATCTTTGAACGCACATTGCGCCCTCTGGTATTCC

GGAGGGCATGCCTGTTTGAGCGTCATTTCtctctcAAACCTTTGGGTTTGGTATTGAGTGATACTCTTAGTCAGACTAAG

CGTTTGCTTGAAAAGTATTGGCACTGAGTGGTATTAATAGTACTTTCTTTGGAACTTCAATGTATTAGGTTTGTCAACTC

GTTGAAACTGAGGAACAGTAAATTTTAGTGCTTAGGCTCGGCCTTACAAGACAACAAACAAGTTTGACCTCAAATCAGGT

AGGACTACCCGCTGAACTTAA

>OTU_814

GAATTGCGATAAGTAATGTGAATTGCAGATACTCGTGAATCATTGAATTtttGAACGCACATTGCGCCCTTGAGCATTCT

CAGGGGCATGCCTGTTTGAGCGTCATTTCCTTCTCAAAAGATAATTTTATTTTGATTtttGGTTGGTTGGCGATACTCAG

GGTTAGCTTGAAATTGGAGACTGTTTCAGTCTTttttAATTCAACACTTAGCTTCTTTGGAGACGCTGTTCTCGCTGTGA

TGTATTTATGGATTTATTCGTTTTACTTTACAAGGGAAATGGTAACGTACCTTAGGCAAAGGGTTGCTTTTAATATTCAT

CAAGTTTGACCTCAAATCAGGTAGGATTACCCGCTGAATTAA

>OTU_815

GAAATGCGATAAGTAATGTGAATTGCAGAATTCAGTGAATCATCGAATCTTTGAACGCACATTGCGCCCGCCAGTATTCT

GGCGGGCATGCCTGTTCGAGCGTCATTTCAACCCTCAAGCCcccGGGCTTGGTGTTGGGGATCGGCGTGCCCTCGCGGCg

cgcCGTCCCCTAAATCTAGTGGCGGTCTCGCTGTAGCTTCCTCTGCGTAGTAGCAACACTCGCACTGGATCGCAGCGCGG

CCACGCCGTTAAACCccccACTTCTGAAAGTTTGACCTCGGATCAGGTAGGAATACCCGCTGAACTTAA

>OTU_816

GAAATGCGATAAGTAGTGTGAATTGCAGAATTCAGTGAATCATCGAATCTTTGAACGCACATTGCGCCCCTTGGTATTCC

ATGGGGCATGCCTGTTCGAGCGTCATTTGTACTCTCAAGCCTTGCTTGGTGTTGGGTGTTTGTCTTTGCTCTGCCTAGAC

TCGCCTTAAATatatTGGCAGCCGGTATTTTGGTTTGAAGCGCAGCACATTTGCTTTTCAAATCTTTATTATTGGTACCC

ATAAGCATTTTAACACTTTTGACCTCGGATCAGGTAGGGATACCCGCTGAACTTAA

>OTU_817

GAAATGCGATAAGTAGTGTGAATTGCAGAATTCAGTGAATCATCGAATCTTTGAACGCACATTGCGCCCATTGGTATTCC

AATGGGCATGCCTGTTCGAGCGTCATTTGTACCCTCAAGCCTTGCTTGGTGTTGGGCGTTTGTCCTCTGGGACTCGCCTT

AAaaaGATTGGCAGCCGGCAGATTGGTTCGGAGCGCAGCACAAATTGCGGTCtctcCATGAATGTCGGCGTCCATGAAGC

CCACTTTTCACTTTTGACCTCGGATCAGGTAGGGATACCCGCTGAACTTAA

>OTU_818

GAAATGCGATAAGCAATGCGAATTGCAGAATTCCGCGAGTCATCAGATCTTTGAACGCAGCCCGCGCTGGAGAGCTATCT

CTTCAGCATGTTTGTTTCAGTgtgtTTGGTTGACATCAAACAACTCAATGCGATTGAAGCCCCTCTTCGCAAGGAGAATG

GGTTTCtctcGTGAAAGAGGAAAGCGTGGGACTCGCCCGCCAACGATGTGGTCACTGTCTAACAGTGAAATTCGTTGGGT

TCATCGAGCGTTTCCATTTTAAACAAaaaTACGCTTtttATAATAAaaaaaCTCTTCAATCCGCACCTGAAATCAAGCAA

GGTCACCCGCTGAACTTAA

>OTU_819

GAAATGCGATAAGTAATGTGAATTGCAGAATGGACGTGAATCATCGAATCTTTGAACGCACCTTGCGCTCGCTGGTATTC

CGGCGAGCATGCCTGTTTGAGTATCGCGCATTCTCGCGAGAGGAACGTGTTTTCCTCTTTGCGGTGCGTGGGCGGTTGTC

GACGTCCGCGTCGACTCGCCTGAAATGCATGAGCACGTGTATATGCGCACGCACCACCGTCTAGGTAAAGCCGAGAGCGG

AGGAGCGAGCGGATCTCGCGGCCGAATAGGTGTCTCGCGACACCAACTGATCGAACGCACTGGTCTCAAATCAGGTAGGA

CTACCCGCTGAACTTAA

>OTU_820

GAAATGCGATAAGTAGTGTGAATTGTAGAATTTAGTAAATTATTGAATCTTTGAACGTATATTGCGCCCCTCGGTATTCC

GTAGGGCATGCCTGTTTAAGCGTTATTTACCCCTTAAGCTCTGCTTGGTGTTAGGCGTTGTCCCGCTTCGcgcgAGAACT

TACCCTAAAGGTATTAGCAGCGGTCGTACTACCCCTTACGCAGTATATTACGCTTCTTAAGGCAGTTGATTAGCGTTTAC

AAAGCTTATATTATATCTTAACCTTAAATTAGGTAGGGATACCCGCTGAACTTAA

>OTU_821

GAAATGCGATAAGTAGTGTGAATTGCAGAATTCAGTGAATCATCGAATCTTTGAACGCACATTGCGCCCCTTGGTATTCC

ATGGGGCATGCTTGTTCGAGCGTCATTTGTACTCTCAAGCTTTGCTTGGTGTTGGGTGCTTGTCCTATTCtctctGATTG

GACTCACCTTAAATTTATTGGCAGCCCAAGTATTGGACTTATGCGCAGCACATTTTGCGAATAAGTCTAATTACATACGC

GTCCATTAAGCATTTTAACACTTTTGACCTCGGATCAAGTAGGGATACCCGCTGAACTTAA

>OTU_822

GAAATGCGATAAGTAATGTGAATTGCAGAATCAGTGAATCATCGAGTCTTTGAACGCACCTTGCGCCCTTTGGTATTCCG

AAGGGCATGCCTGTTTGAGTGTCATGAAAACCTCAATCCCTCCGGTTTTCACGAACCGGTTGGACTTGGATTTGGGTGTT

GCCGCGGCTCGCGCCGTCGGCTCGCCTCAAAAGTGTTAGTGGGACGGTGAACACCCGTCAGCCTGGCGTAATAAGTTTCG

CTGGGCCTCTGGGGTCGTCGACGGCTTGCTCATAACAAAACCTATTTCTATGACTTCTGACCTCAAATCAGGTAGGGCTA

CCCGCTGAACTTAA

>OTU_823

GAAATGCGATAAGTAGTGTGAATTGCAGAATTCAGTGAATCATCGAATCTTTGAACGCACATTGCGCCCCTTGGTATTCC

ATGGGGCATGCCTGTTCGAGCGTCATTTGAACCCTCAAGCTTTGCTTGGTGTTGGGTGTTTGTCTCGCTTTTGCGCGTGG

ACTCGCCTTAAAGTTATTGGCAGCCATGTAATTCGGCTTTGAGCGCAGCACATTGCGATCtctctGCTGGTACATTGGCA

TCCAGAAGCCTTtttttAATCTTGACCTCGGATCAGGTAGGGATACCCGCTGAACTTAA

>OTU_824

GAAATGCGATAAGTAATGTGAATTGCAGAATTCAGTGAATCATCGAATCTTTGAACGCACATTGCGCCTTTTGGTATTCC

GAAAGGCATGCCTGTTCGAGCGTCATTTAAAACTTCAAGCTCAGCTTGGTTTTGAGAGCTGTCAAAAGACACTTTCGCAA

CTAGTTGGCGTGATTACATTTCATCAAACGTAGCAATGTTATCGTTTAGTTGTAATGTGGTACCGACCATTTAATAAAAT

CTCAAAGTTTGACCTCGGATCAGGTAGGGATACCCGCTGAACTTAA

>OTU_825

GAAACGCGATAGGTAATGTGAATTGCAGAATTCCGTGAATCATCGAATCTTTGAACGCACATTGCGCCTGCTGGTATTCC

GGTAGGCATGCCTGTTTGAGCGTCAGCAAaaaCCTCTCAGCAATTTATTTTGTTGGTTATGGGTAAAGAAGTATGGCTAA

CTGCGCCCATTCTTACCTTAAACTTGCTGCACGACCGCAGGCTTtttATGCTAGGTTAATGGAGTGGTGTTTACAATTAT

TCCCAGCTGCCTGCATAGCGAGTGGCGTTAAATCTGATGTTGCCTCTGCTGCAACTGAAGTTTTGACCTCAAATCAGACA

AGGATACCCGCTGAACTTAA

>OTU_826

GAAATGCGATAAGTAATGTGAATTGCAGAATTCAGTGAATCATCGAATCTTTGAACGCACATTGCGCTCGCCAGTATTCT

GGCGGGCATGCCTGTTCGAGCGTCATTTCAACTATCAAGCCCCAGGCTTGTATTGGAGCCCTGCGGCTGCCGCAGCCTCC

CAAAATCAGTGGCGGGCTCGCTATCACACCGAGTGCAGTAGTTTACTCTTCGCTTAGGTTCGTATAGCGGGTGCTAGCCG

TGAAACCccccACTTCTCAAGGTTGACCTCGGATCAGGTAGGAATACCCGCTGAACTTAA

>OTU_827

GAAATGCGATAAGTAATGTGAATTGCAGAATTCAGTGAATCATCGAATCTTTGAACGCACCTTGCGCTCCTTGGTATTCC

GAGGAGCATGCCTGTTTGAGTGTCATGGAATTCTCAACCTTATCAGCTTttttGTTGACTAGGCTTGGATGTGGGAGTTT

GCGGGCTTCTCAGAAGTCGGCTCTCCTTAAATGCATTAGCGGGACCCTTGTTGCCTAGCTTTGGTGTGATAATTATCTAC

GCCATTGTCTTATACATGGGGTACAGCTTCTAACCGTCtctctAGGGACAACTTCTGACAATTTGACCTCAAATCAGGTA

GGACTACCCGCTGAACTTAA

>OTU_828

GAAATGCGATACGTAGTGTGAATTGCAGAATTCAGTGAATCATCGAATCTTTGAACGCACATTGCGCCCTTTGGTATTCC

AAAGGGCATGCCTGTTCGAGCGTCATTTGTACCCTCAAGCTTTGCTTGGTGTTGGGCGTTttttGTCTTGCTCTCAGCAA

GACTCGCCTTAAAATGATTGGCAGCCGGCCTACTGGTTTCGGAGCGCAGCAGATTCTTtttGCGCTTGCAACCAGCAAAA

GAGGTTGGCGATCCAGCAAGTACATCTTCTCACTTTTGACCTCGGATCAGGTAGGGATACCCGCTGAACTTAA

>OTU_829

GAAATGCGATAAGTAATGTGAATTGCAGAATTCAGTGAATCATCGAATCTTTGAACGCACCTTGCGCTCCTTGGTATTCC

GAGGAGCATGCCTGTTTGAGTGTCGTGAAATTATCAACTCCTATTCTTTATTGAATGGTGAGCTTGGATTTGGAGATTGC

TGGTGAAAATCGGCTTCTCTTGAATGAATTAGCTGGAATTTGATTCGCAGCATatatGCGGTGTGATAATGTCGTCACTG

TGTAGCTCGGATTGTCTGGCTTCTAATCGTCTTTCATGGACAATTTGATCATTTTGACCTCAAATCAGGTAGGACTACCC

GCTGAACTTAA

>OTU_830

GAAATGCGATACGTAATGTGAATTGCAGAATTCCGTGAATCATCGAATCTTTGAACGCACATTGCGCCCCTTGGTATTCC

AGGggggCATGCCTGTTTGAGCGTCATTTCCTTCTCAAACATTCTGTTTGGTAGTGAGTGATACTCTTTGGAGTTAACTT

GAAATTGCTGGCCTTtttCATTGGATGTTtttttttCCAAAGagagGTTTCTCTGCGTGCTTGAGGTATAATGCAAAGTA

CGGTCGTTtttAGGTTtttACCAACTGCGGCTAATCTTttttttATACTGAGCGTATTGGAACGTTATCGATAAGAAGag

agCGTCTAGGCGAACAATGTTCTTAAAAGTTTTGACCTCAAATCAGGTAGGAGTACCCGCTGAACTTAA

>OTU_831

GAAATGCGATAAGTAATGTGAATTGCAGAATTCAGTGAATCATCGAATCTTTGAACGCACATTGCGCCCCTTGGTATTCC

GAGGGGCATGCCTGTTCGAGCGTCATTACAACCCCTCAAGCTCAGCTTGGTGTTGGGGCCTGCCGCGCTGGCAGCCCTTA

AAATCAGTGGCGGTGCCACCTGGCTCTAAGCGTAGTAACTTTTCTCGCTACAGTGTCCTGGTGAGCGCTTGCCATAACCT

ATATTTTCTATGGTTGACCTCGGATCAGGTAGGGATACCCGCTGAACTTAA

>OTU_832

GAAATGCGATACGTAATGTGAATTGCAGATTTCAGTGAATCATCGAATCTTTGAACGCACATTGCGCCCGCTAGTATTCT

GGCGGGCATGCCTGTCCGAGCGTCATTTCAACCATCAAGCGCTGCGCTTGTGTTGGAGCCCTGCGGCTGCCGCAGGCTCC

CAAACCCAGTGGCGGGCTCGTCGTCGTACCGAGTGCAGTAAACTTACCACGCTCAGGGCACGCGACGGGTGACCGGCCGT

AAAACCccccccTTCTCAAGGTTGACCTCGGATCAGGTAGGAATACCCGCTGAACTTAA

>OTU_833

GAAATGCGATAAGTAGTGTGAATTGCAGAATTCAGTGAATCATCGAATCTTTGAACGCACATTGCGCCCCTTGGTATTCC

ATGGGGCATGCCTGTTCGAGCGTCATTTGTACCCTCAAGCTCTGCTTGGTGTTGGGTGGTTGTCTCTGCTCTGCTTAGAC

TCGCCCTAAATatatTGGCAGCCGGTATATTGGTTTGAAGCGCAGCACAATTGCAATTTGAGCCTATATTACTGGTATCC

ATTAAGCAAATTATCACTTTTGACCTCGGATCAGGTAGGGATACCCGCTGAACTTAA

>OTU_834

GAAATGCGATAAGTAATGTGAATTGCAGAATTCAGTGAATCATCGAATCTTTGAACGCACATTGCGCCCCTCGGTATTCC

GGggggCATGCCTGTTCGAGCGTCATTACACCACTCAAGCCTCGCTTGGTCTTGGGCGTCCGCGGTCCGCCGcgcgcCCC

AATGTCTCCGGCTGAGCCGTCCGTCTCTAAGCGTTGTGATAAACTGTTCGCTTGCGAGGCCGGGCTGGCTTGTTTGCCGT

TAAACCccccGTTtttACAGGTTGACCTCGGATCAGGTAGGGATACCCGCTGAACTTAA

>OTU_835

AAAATGCGATAAGTAGTGTGAATTGCAGAATTCAGTGAATCATCGAATCTTTGAACGCACATTGCGCCCCTTGGTATTCC

ATGGGGCATGCCTGTTCGAGCGTCATTGACACCCTCAAGCTCTGCTTGGTGTTGGGCCGTTGTCGTTTCCTCGCCccccc

ccGGGCGACACAGCGACACCCCTCAAAGTCATTGGCGGCTTTCCTGCCCAGCTTTCCCGCGCAGCAGTTCGCGCTTCGGA

GACCACTTGGCAGGTTGGCATCCATCAAGCTGACCCCTCACGCTTGACCTCGGATCAGGTAGGGACTACCCGCTGAACTT

AA

>OTU_836

GAAATGCGATAAGTAGTGTAAATTACAGAATTTAGTAAATTATCGAATCTTTAAACGTATATTACGCCCCTTAGTATTCC

GTAGAGCATACCTGTTTAAGCGTTATTTACCCCTTAAGCTCTGCTTAGTGTTAGGCGTTATCCCGCTTCGcgcgAGGACT

TACTCTAAAGGTATTAGCAGCGGTCGTGCCACCCCTCGCGCAGTATATTACGCTTCTTAAGGCGGTTAATTAGCGTTTAC

AAAGCTTACATTATATCTTAACCTTAGATTAGGTAGAGATACCCGCTAAACTTAA

>OTU_837

GAAATGCGATAAGTAGTGTGAATTGCAGAATTCAGTGAATCATCGAATCTTTGAACGCACATTGCGCCTCCTGGCATTCC

GGGAGGCATGCCTGTTCGAGCGTCATAAAATACCTCTCAAGCATATTTGCTTGGTTGTGGAAGAAGAGTATGCCAGCATT

CTCCCTTCTGAAATTGAAAGGCGGATCATAGCATATTCCGTGGCGTAGTAAGTTTATCTTGTCGCTCGGAGTGTGCGCAA

TTGTCCTGCCATCAAACCcccAATTCTTCAAGGTTGACCTCGGATCAGGTAGGGATACCCGCTGAACTTA

>OTU_838

GAAATGCGATAAGTAATGTGAATTGCAGAATTCAGTGAATCATCGAATCTTTGAACGCACCTTGCGCTCCTTGGTATTCC

GAGGAGCATGCCTGTTTGAGTGTCATTAATatatCAACCTCTTTAAATTtttGAGAGTGTTTGGATGTGGgggTTATTTT

GCTGGCCTTCTCCAAGGTCAGCTCCCCTGAAATGTATTAGCGGAACAATTTGTTGACCGTTCATTGGTGTGATAGCATAT

CTACGCTATTGACGTAAAGCAAGTTCAGCTTATAACAGTCCATTGACTTGGACAATTCTCTATTTATTAATGTGACCTCA

AATCAGGTAGGACTACCCGCTGAACTTAA

>OTU_839

GAAATGCGATAAGTAGTGTGAATTGCAGAATTTAGTGAATTATCGAATCTTTAAACGCACATTGCGCCCCTTAGTATTCT

ATAGGGCATGCCTGTTCAAGCGTTATTTGTACCCTTAAGCTCTGCTTAGTGTTAGGTGTTTGTCTCCTCTAGGAGACTCG

CCTCAAAACAATTAGCAGCCGGCATATTAGTATTAGAGCGCAGCACAAGTCGCGCTTCTGTCTATGTTTATTAGCATCTA

GTAAGACCATTtttCACTCTTAACCTCGGATTAGGTAGGGATACCCGCTGAACTTAA

>OTU_840

GAAATGCGATAAGTAATGTGAATTGCAGAATTCAGTGAATCATCGAATCTTTGAACGCACATTGCGCCCGCCAGCATTCT

GGCGGGCATGCCTGTTCGAGCGTCATTTCAACCCTCAAGCTCTGCTTGGTGTTGGGGCCCTACAGCTGATGTAGGCCCTC

AAAGGTAGTGGCGGACCCTCCCGGAGCTCCTTTCGTAGTAACTTTACGTCTCGCACTGGGATCCGGAGGACTCTTGCCGT

AAACCCCAATTTTCCAAAGGTTGACCTCGGATCAGGTAGGAATACCCGCTGAACTTAA

>OTU_841

GAAATGCGATAAGTAGTGTGAATTGCAGAATTCAGTGAATCATCGAATCTTTGAACGCACATTGCGCCCCTTGGTATTCC

ATGGGGCATGCCTGTTCGAGCGTCATTTGTACCCTCAAGCTCTGCTTGGTGTTGGGTGTTTGTCCTCTCCATTGCGGTTG

GACTCGCCTTAAAGTAATTGGCAGCCAGTGTATTGGTCTTGAAGCGCAGCACAATTTGCGATTCTTGCTAATCATCACTA

GCATCCATAAAGCCTTttttATCACTTTTGACCTCGGATCAGGTAGGGATACCCGCTGAACTTAA

>OTU_842

GAAATGCGATAAGTAATGTGAATTGCAGAATTCAGTGAATCATCGAATCTTTGAACGCACATTGCGCCcccTGGTATTCC

GGggggCATGCCTGTTCGAGCGTCATTTCACCACTCAAGCTCGCTTGGTATTGGGCAACGCGGTCCGCCGCGTGCCTCAA

ATCGACCGGCTGGGTCTTCTGTCCCCTAAGCGTTGTGGAACTATTCGCTAAGGGTGCTCGGGAGCTACGCCGTAAACAAC

CATTTCTAGGTTGACCTCGGATCAGGTAGGGATACCCGCTGAACT

>OTU_843

GAAATGCGATAAGTAATGTGAATTGCAGAATTCAGTGAATCATCGAATCTTTGAACGCACATTGCGCCCTTTGGTATTCC

GAAGGGCATGCCTGTTCGAGCGTCATTTCACCACTCAAGCCTGGCTTGGTATTGGGCGTCGCGGTGTCCGcgcgcCTTAA

AGTCTCCGGCTGAGCTGTCCGTCTCTAAGCGTTGTGATACATATTCGCTTCGGAGCGCGGGTGGCCGCGGCCGTTAAAAC

TTATTCAAAGGTTGACCTCGGATCAGGTAGGGATACCCGCTGAACTTAA

>OTU_844

GAAATGCGATAAGTAGTGTGAATTGCAGAATTCAGTGAATCATCGAATCTTTGAACGCACATTGCGCCTTCTGGTACTCC

GGGAGGCATGCCTGTTCGAGCGTCATCAAACACATCCTCAAGCATCTTTTGCTTGGTCTTGGgggAAGATGCCCGCATCt

ctcCCGAAATCTGTCAGCGGAAAAGCCATGTCAGTCTACAGGTGTGATACTTACTGTTCACCTGGTGACTCCGATGGTGT

TCTTGCTGTTAATAGCCccccAGCTTCTCATGAGTTTGACCTCGGATCAGGTAGGGATACCCGCTGAACTTAA

>OTU_845

GAAATGCGATAAGTAGTGTGAATTGCAGAATTCAGTGAATCATCGAATCTTTGAACGCACATTGCGCCCCTTGGTATTCC

ATGGGGCATGCCTGTTCGAGCGTCATTTGTACCTTCAAGCTCTGCTTGGTGTTGGGTGTTTGTCTCGCCTCTGCGCGTAG

ACTCGCCTCAAAACAATTGGCAGCCGGCGTATTGATTTCGGAGCGCAGTACATCTCGCGCTTTGTGACTCAACCGCGGCG

GCGTCCAGAAGTACATTtttttACACTCTTGACCTCGGATCAGGTAGGGATACCCGCTGAACTTAA

>OTU_846

GCCTGCTTGATGCGCTCGGGGTAGTGCTCGTAGGGCTGGTGGGCGGAGAGCATGTCGTTGTAGGCGGTCACGATGGCGAC

GTTGGCGGCGTCCATCAGGCGCAGGCGCTGCTTGTCCTCGCCACCGCAGCCGGCTACGCCGTGGGCGAAGTTGGCGCACT

GCAGCTTGCCGCGCTGCGGGCCCTTGCTGGCGGCCTCGCGGATCATCGCCAGGTAGCGCTCGCGGGTGGGACGGCTGCGG

GCGATAAGGCGTTCGGTTACTTCGATAATGCGGGGAT

>OTU_847

GAAATGCGATAAGTAATGTGAATTGCAGAATTCAGTGAATCATCGAATCTTTGAACGCACCTTGCGCTCTTTGGTATTCC

GAAGAGCATGCCTGTTTGAGTGTCATTAAATTCTCAACCTCACTAGTTTTCTGATGAGGCTTGGATGtgtgGgggTTTGT

GCAGGCTGCCTCAGCGCGGTCTGCTCCCCTAAAATGCATTAGCGAGTTCATACTGAGCTCCGTCTATTGGTGTGATAATT

ATTACGCCGTGGATTGGGCTTAGACTTGCTTCTAACCGTCCGCAAGGACAACTCTATGACAATTTGACCTCAAATCAGGT

AGGACTACCCGCTGAACTTAA

>OTU_848

AAAATGCGATAAGTAGTGTAAATTGCAGAATTTAGTAAATTATTAAATCTTTAAACGCATATTACGCCCCTTAGTATTCT

ATAGGGCATGCCTGTTTAAGCGTTATTTGTACCCTTAAGCTCTGCTTAGTGTTAGGTGTTTATCTCCTCTAGGAGACTCG

CCTTAAAACAATTGGCAGCCAGCATATTAGTATTAGAGCGCAGCACAAGTCGCGCTTCTGTCTATGTTTGTTAGCATCTA

GCAAGACTATTtttCACTCTTAACCTTAGATTAGGTAGGGATACCCGCTAAACTTAA

>OTU_849

GAAATGCGATAAGTAATGTGAATTGCAGAATTCAGTGAATCATCGAATCTTTGAACGCACATTGCGCCCCTTGGTATTCC

GAGGGGCATGCCTGTTCGAGCGTCATTACACCACTCAAGCTATGCTTGGTATTGGGCGTCGTCCTTAGTTGGGCgcgcCT

TAAAGACCTCGGCGAGGCCACTCCGGCTTTAGGCGTAGTAGAATTATTCGAACGTCTGTCAAGGAGAGGAACTCTGCCGA

CTGAACCTTTATTTTCTAGGTTGACCTCGGATCAGGTAGGATACCGCTGAACTTAA

>OTU_850

GAAATGCGATAAGTAATGTGAATTGCAGAATTCAGTGAATCATCGAATCTTTGAACGCATATTGCGCCCCTTGGTATTCC

GGggggCACACCTGTTCGAGCGTCATTACAACACTCAAGCACCGCTTGGTATTGGGCGCCGCCTTCTACGGGCgcgcCTT

AAACTCCTCGGCGTCGCCGTCCGTCTTTAAGCGCAGCGAGATTtttCCAGCGCCTTCAAAGCCGGGCCTCGTCGCCGGAT

AACGTCTTATCTCTTCAAGGTTGACCTCGGATCAGGTGGGGATACCCGCTGAACTTAA

>OTU_851

GAAATGCGATAAGTAATGTGAATTGCAGAATTCAGTGAATCATCGAATCTTTGAACGCACATTGCGCTCCCTGGTATTCC

GGGGAGCATGCCTGTTCGAGCGTCATTACAACCCTCAAGCTCTGCTTGGTATTGGGCTCTGCTGGAAACGGCAGGCCTTA

AAATCAGTGGCGGTGCCTCTTGGCTCCAAGCGTAGTAATTCTTCTCGCTTTGGATCACCAAGTgtgtgCTTGCCAGTAAC

CcccAATTtttAAAGGTTGACCTCGGATCAGGTAGGGATACCCCGTGAACTTAA

>OTU_852

GAATTGCGATAAGTAATGTGAATTGCAGATACTCGTGAACTTGATTTTGAATTACGCACATTGCGCCCTTGAGCATTCTC

AGGGGCATGCCTGTTTGAGCGTCATTTCCTTCTCAAAAGATAATTTATTATTttttGGTTGTGGGCGATACTCAGGGTTA

GCTTGAAATTGGAGACTGTTTCAGTCTTttttAATTCAACACTTAGCTTCTTTGGAGACGCTGTTCTCGCTGTGATGTAT

TTATGGATTTATTCGTTTTACTTTACAAGGGAAATGGTAACGTACCTTAGGCAAAGGGTTGCTTTTAATATTCATCAAGT

TTGACCTCAAATCAGGTAGGATTACCCGCTGAACTTAA

>OTU_853

GAAATGCGATAAGTAATGTGAATTGCAGAATTCAGTGAATCATCGAATCTTTGAACGCACATTGCGCCcccTGGTATTCC

GGggggCATGCCTGTCCGAGCGTCATTACAACCCTCAAGCCTAGCTTGGTGTTGGGCCCTGCCGCGTGGCAGGCCCTAAA

GTCAGTGGCGGTGCCGTCCGGCTCCGAGCGTAGTAACTCTTCTCGCTCTGGAGGCCCGGGCGTGTGCCTGCCAGCAACCc

cccATTtttCAGGCTTGACCTCGGATCAGGTAGGGATACCCGCTGAACTTAA

>OTU_854

GAAATGCGATACGTAATGTGAATTGCAGAATTCAGTGAATCATCGAGTCTTTGAACGCACATTGCGCCCTCTGGTATTCC

GGAGGGCATGCCTGTCCGAGCGTCATTGCTGCCCTCAAGCACGGCTTGtgtgtTGGGCTCCGTCCTCCTTCCGGgggACG

GGCCCGAAAGGCAGCGGCGGCACCGCGTCCGGTCCTCAAGCGTATGGGGCTTTGTCACCCGCTTTGTAGGACTGGCCGGC

GCCTGCCGATCAACCAAACTTtttCCAGGTTGACCTCGGGATCAGGTAGGGATACCCGCTGAACTTAA

>OTU_855

AAACTGCGATAAGTAGCGTGAATTGCAGACGCTTTGAACGTTAAACTTTCGAACGCACATTGCGCCGTAGGAGTCCTACC

CTGCGGCACATCTGGTTGAGGGTCGTGATCAAaaaCAGCAGGACTATGGCTGTAATAGAAATAGCTGGTGAATCACGAGT

TCGGCCTACCGCCTCTGGCTGAACGTGTTTTGCCATAATGCTAGCAGAGGACAATCGCTCTTACGAgagaTTGCCTACCA

CACGGATGATCTATTAGCTGAAGCTATAGGTCTCGGTCACCGGGTATCATGCTTAtataCTCTGTGGCGTCTGAGATACA

GCTAGTGGGCTTCATACGTGCCTGGTATTGAGAGTATTCGCTCTCCCGACCTCAACTCAGGTGTGATTACCCGCTGA

>OTU_856

GAAATGCGATAAGTAGTGTGAATTGCAGAATTCAGTGAATCATCGAATCTTTGAACGCACATTGCGCCCCTCGGCATTCC

GTGGGGCATACCTGTTCGAGCGTCATTTCAACCCTCAGGCGGCTCTTTTGGTCTGTCTGGTCTTGGGCATCGTCCAAGGA

CGTGCCTTAAAAGAATTGGCGGCGTAGGTGCGGCTTCGAGCGTAGCAGATGCGATTCGCTTTGGAAGCCGGCGCTCACCT

TGCCAGAATAATTTCTGAATGGTTGACCTCGGATCAGGTAGGGATACCCGCTGAACTTAA

>OTU_857

GAAATGCGATAAGTAGTGTGAATTGCAGAATTCAGTGAATCATCGAATCTTTGAACGCACATTGCGCCCCTTGGTATTCC

ATGGGGCATGCCTGTTCGAGCGTCATTTGTACCCTCAAGCTATGCTTGGTGTTGGGTGTTTGTCCTCTCCATTGCGTTTG

GACTCGCCTTAAAGCAATTGGCAGCCAGTGTTTTGGTTTTAAGCGCAAGCACATTTTGCGTCTTACTCCCTTGAATACTA

GCATCCACTAAGCCTTtttATTACTTTTGACCTCGGATCAGGTAGGGATACCCGCTGAACTTAA

>OTU_858

GAAATGCGATAAGTAGTGTGAATTGCAGAATTTAGTGAATTATCGAATCTTTAAACGCACATTGCGCCCTTTGGTATTCC

AAAGGGCATGCCTGTTCGAGCGTCATTTGTACCCTCAAGCTTTGCTTAGTGTTAGGCGTCTTGTCTCTAGCTTTGCTAGA

GACTCGCCTTAAAGTAATTAGCAGCCGGCCTACTAGTTTCAGAGCGCAGCACAAGTCGCACTctctATCAGCAAAGGTCT

AGCATCCATTAAGCCTTttttttAACTTTTAACCTCGGATCAAGTAGGGATACCCGCTGAACTTAA

>OTU_859

GAAATGCGATAAGTAGTGTGAATTGCAGAATTCAGTGAATCATCGAATCTTTGAACGCACATTGCGCCCTTTGGTATTCC

AAAGGGCATGCCTGTTCGAGCGTCATTTGTACCCTAAGCTTTGCTTGGTGTTTGGGCGTTCTTGTCTCTAGCTTTGCTGG

AGACTTCGCCTTTAAAGTAATTGGCAGCCGGCCTACTGGTTTCGGAGCGCAGCACAAGTCGCACTctctATCAGCAAAGG

TCTAGCATCCATTAAGCCTTtttttCAACTTTTGACCTCGGATCAGGTAGGGATACCCGCTGAACTTA

>OTU_860

AAAATGCGATAAGTAATGTAAATTGCAGAATTTAGTGAATTATTGAATCTTTGAACGCATATTGCGCTTGCTAGCATTCT

GGCAGGCATACCTATTTGAGCGTTATTTCAACCCTCAAGCTCCGCTTAGTGTTAGGGCCCTACAGCTGATATAGGCCTTT

AAAGGTAGTGGCAGACCCTCCTAGAGCCTCCTTTGCGTAGTAACTTTACGTCTTGCACTAGGATCTAGAGGGACTCTTGC

TATAAAACCcccTAATTTTCTAAAGGTTAACCTTAGATCAAGTAGGAATACCTGCTGAACTTAA

>OTU_861

GAAATGCGATAAGTAATGCGAATTGCAGAATTCAGTGAATCATCGAATCTTTGAACGCACATTGCGCCCGCCAGTATTCT

GGCGGGCATGCCTGTCTGAGCGTCGTTTCGACCCTCGCGCCCGGCTTCTGTCGGgggCGGTGTTGGGGATCGGCCACACC

CTTCAGTGGGAGGCCGGCCCCTAAATTCAGTGGCGACCACGCTGTAGCCTCCCCTGCGTAGTACTAAAACCACCTCGCAG

GCGGAGAGCGGTGCGGCCCGCCGTAAAACCccccAACTTTTACAAGGTCGACCTCAGCATCAGGTAGGAATACCCGCTGA

ACTTAA

>OTU_862

GAAATGCGATAAGTAGTGTGAATTGCAGAATTCAGTGAATCATCGAATCTTTGAACGCACATTGCGCCCCTTGGTATTCC

ATGGGGCATGCCTGTTCGAGCGTCATTTGTACCCTCAAGCTCTGCTTGGTGTTGGGTGTTTGTTCCCTGAACTCACCTTA

AAACAATTGGCAGCCGGCATGTTGGCCTGGAGCGCAGCACAATTTGCGTCCCATTCCAGTAGTGTTGGCACCCATCAAGA

CCTATATTTTGCTCTTGACCTCGGATCAGGTAGGGATACCCGCTGAACTTAA

>OTU_863

GAAATGCGATAAGTAATGTGAATTGCAGAATTCAGTGAATCATCGAATCTTTGAACGCACATTGCGCCCCTTGGTATTCC

GGggggCATGCCTGTTCGAGCGTCATTTCAACCCTCAAGCTTAGCTTGGTATTGAGTCTATGTCAGTAATGGCAGGCTCT

AAAATCAGTGGCGGCGCCGCTGGGTCCCTGAACGTAGTAATATCtctcGATTAGCAGGTTCGTCGGTGTGCTTCTAGCCA

AAACCCCAAATTtttCTATGGTTGACCTCGGATCAGGTAGGGATACCCGCTGAACTTAA

>OTU_864

GAAATGCGATAAGTAATGTGAATTGCAGAATTCCGTGAATCATCGAATCTTTGAACGCACATTGCGCCcccTGGTATTCC

GGggggCATGCCTGTCCGAGCGTCATTTCAAACCCTCAAGCGCGGCTTGtgtgtTGGGCTGCAGCGTCCCTTtttCTAAA

AGGACGGGCCCCAAATGTAGTGTGCGGCGCGGTGCGCTTAGCTGCAGTGTATGGGAACCTCTCACACTCGCGGTgtgtCA

CCACGCTCGCCACCTCTCACTctctTCTCGGTTTGACCTCGGATCAGGTAGGGATACCCGCTGAACTTAA

>OTU_865

GAAATGCGATAAGTAATGTGAATTGCAGAATTCAGTGAATCATCGAATCTTTGAACGCACATTGCGCCCCGTGGTATTCC

GCGGGGCATGCCTGTTCGAGCGTCATTTCACCACTCAAGCCTCGCTTGGTATTGGGCGCCGCGGCCTCGTCCGcgcgcCT

CGAAATCTCCGGCTGAGCCGTCCGTCTCCCCGCGTTGTGAACTTTACTGTTTCGCTGCGGAGCACGGGCGACCCCGGCCG

TTAAATCTCATCAAAGGTTGACCTCGGATCAGGTAGGGATACCCGCTGAACTTAA

>OTU_866

GAAATGCGATAAGTAGTGTGAATTGCAGAATTCAGTGAATCATCGAATCTTTGAACGCACATTGCGCCCCTTGGTATTCC

ATGGGGCATGCCTGTTCGAGCGTCATTTGTACTCTCAAGCTCTGCTTGGTGTTGGGTGTTTGTCTAGACCTTGCGTATAG

ACTCGCCTTAAATatatTGGCAGCCGATATTTTGGTTTGAAGCGCAGCACATTTTGCGATTCGGCCTCTATTATTAGCGT

CCAAGAAGTATAATTAACACGCTTGACCTCGGATCAGGTAGGGATACCCGCTGAACTTAA

>OTU_867

GAAATGCGATACTTGGTGTGAATTGCAGAATCCCGTGAACCATCGAGTCTTTGAACGCAAGTTGCGCCCGAGGCCTCCTG

GTCGAGGGCACGTCTGCCTGGGTGTCACGCATCGTCGGCCCCTCTCCCCTCGGCTCGCGAGGGCGGgggCGGATACTGGT

CTCCCGcgcgcTCCCGCTCGCGGTTGGCCCAAAATCGAGTCCTCGGCGACGTCGCCACGACGAGCGGTGGTTGAgagaCC

CTCGGACACGGTCGTGCgcgcgcCTGTCGCCcccGGGATCTCCTGGACCCTCAGGCATCGAATTTCTAGGATGCTCTCGT

TGCGACCCCAGGTCAGGCGGGACTACCCGCTGAGTTTAA

>OTU_868

GAAATGCGAAAAGTAGTGTGAATTGCAGAATTCAGTGAATCATCGAATCTTTGAACGCACATTGCGCCCCTTGGTATTCC

ATGGGGCATGCCTGTTCGAGCGTCATTTGTACCTTCAAGCTCTGCTTGGTGTTGGGTGTTTGTCCCGCTTtttGCGTGGA

CTCGCCTCAAAGCAATTGGCAGCCGGCGTACTAGCCTGGAGCGCAGCACATTTTGCGCATCTTGTCTTGAACGCTTGCGT

CCATTAAGCACATATTTTAACTCTTGACCTCGGATCAGGTAGGGATACCCGCTGAACTTAA

>OTU_869

GAAATGCGATAAGTAGTGTGAATTGCAGAATTCAGTGAATCATCGAATCTTTGAACGCACATTGCGCCCTTTGGTATTCC

TTAGGGCATGCCTGTTCGAGCGTCATTTACAAATTCAAGCTCAGCTTGGTGATGGGCGCCTGTCTCCCGCAACGCGCAGA

TTCCTCAGCTTGCTGACGATGGGTCTTTGCAGCGTGTAGGTAGACTCGCCTAAaaaCAGTTGGCAGCCTCTACTTCAGTT

CCAAACGCAGCGGAATGGCGGATCGTACTGCAAGAATAGGCTCTCCAGTAAGCAAAATTCCACAATTTGACCTCGGATCA

GGTAGGGATACCCGCTGAACTTAA

>OTU_870

GAAATGCGATAAGTAATGTGAATTGCAGAGTTCAGTGAATCATCGAATCTTTGAACGCACATTGCGCCCGGCAGTAATCT

GCCGGGCATGCCTGTCCGAGCGTCATTTCACCCCTCGAGCCGCTTTTAGTGGATCGGTGTTGGGGCACTACGGTAAAACG

TAGGCCCCGAAATGAAGTGGCGGTCCCGCCGCGGTGCCcccATGCGTAGTAACGTTTAGTCTCGCATCGGGTCCCGGCGG

AGGCCTGCCGTCAAGCCTATTTATTCTTAGAATAGTTTGACCTCGGATCAGGTAGGGTTACCCGCTGAACTTAA

>OTU_871

GAAATGCGATAAGTAGTGTGAATTGCAGAATTCAGTGAATCATCGAATCTTTGAACGCACATTGCGCCCCTTGGTATTCC

ATGGGGCATGCCTGTTCGAGCGTCATTTGTACTCTCAAGCCTTGCTTGGTGTTGGGTGTTTGTCTCTGCTCTGCTTAGAC

TCGCCTTAAATACATTGGCAGCCGGTATACTGGTTTGAAGCGCAGCACATTTGCAATTCAAGCCTGCTATTATTGGTATC

CACAAAGAATATTAACACTTTTGACCTCGGATCAGGTAGGGATACCCGCTGAACTTAA

>OTU_872

GAAATGCGATAAGTAATGTGAATTGCAGAATTCAGTGAATCATCGAATCTTTGAACGCACATTGCGCCCTTTGGTATTCC

GAAGGGCATGCCTGTTCGAGCGTCATTTACACCCCTCGAGCCcccGGGCTCGGTCTTGGGCGGTCGTCCTTCCccccGGG

AGGACGCCcccGAAATGCAGCGGCGGTGCGGCCCGCGACCcccAGCGCAGTGACGAGCCcccAGGGCACATCTCGCTGGC

GGCTCCCGGGCGTCCACCCGCCGGACAACAACCAAAATTCTATACACGATTGACCTCGGATCAGGTAGGGATACCCGCTG

AACTTAA

>OTU_873

GAAATGCGATAAGTAATGTGAATTGCAGAATTCAGTGAATCATCGAATCTTTGAACGCACCTTGCGCCTCTTGGTATTCC

GAGAGGTATGCCTGTTTGAGTGTCATGAAACCTCACCcccTCTGGGTTtttGACCTGGTCGGCGGTGGATTGGGCGCCTG

CCGTCACTGGCTCGCCTGAAAAGCATAAGCACCTTGGATGTAATACGTTTCATCCTCTCGGGTGGCTGACAACCCCACAT

ACTTCATGATCTGGCCTCAAATCAGGTAGGGCTACCCGCTGAACTTAA

>OTU_874

GAAATGCGATAAGTAATGTGAATTGCAGAATTCAGTGAATCATCGAATCTTTGAACGCACATTGCGCCcccTGGTATTCC

GGgggCATGCCTGTTCGAGCGTCATTTCACCACTCAAGCCTCGTCTTGGTATTGGGCAACGCGGTCCGCCGCGTGCCTCA

AATCTGACCGGCTGGGTCTTTTCATGTCCCCTAAGCGTTGTGGAAACTATTCGCTAAAGGGTGCTCGGGGAGGCTTACCG

CCGTAAAACAAACCCATTTCTAAGGTTTTGACCTCGGATCAGGTAGGGATACCCGCTGAACTTAA

>OTU_875

GAAATGCGATAAGTAGTGTGAATTGCAGAATTTAGTAAATTATTAAATCTTTAAACGCATATTGCGCCCCTTAGTATTCC

GTAGGGTATGCCTATTTAAGCGTTATTTACCTCTTAAGCTCTGCTTAGTGTTAGGCGTTATCCTGCTTCGCGTAAGAACT

CGCTCTAAAGGTATTAGCAGCGGTCGTGCTACCCCTCGCGTAGTATATTACGCTTCTTAAGGCGGTTAATTAGCGTCTAC

AAAGCTTATATTATATCTTAACCTTAGATTAGGTAGGGATACCCGCTAAACTTAA

>OTU_876

GAAATGCGATAAGTAGTGTAAATTGCAGAATTTAGTAAATTATTAAATCTTTAAACGCACATTGCGCCCCTTAGTATTCT

ATAGGGCATGCCTGTTCAAGCGTTATTTGTACCCTTAAGCTCTGCTTGGTGTTAGGTGTTTGTCTCCTCTAGGAGACTCG

CCTTAAAACAATTAGTAGCTAGCATATTAGTATTAGAGCGCAGCACAAGTCGCGCTTCTATCTATATTTATTAGCATCTA

GCAAGACTATTttttACTCTTAACCTTAGATTAGGTAGAGATACCCGCTAAACTTAA

>OTU_877

GAAACGCGATAGTTAATGTGAATTGCAGAATTCAGTGAATCATCGAGTCTTTGAACGCACATTGCGCCTTTTGGTATTCC

GAGAGGCACGCCTGTCTGAGCGTCAGTAATACCTTCGGTTTGACGATTTATTGTCTCGCCGGCGTTGGCGGTTTTGGGAT

ACGTGGGGCAACCCGCGCCATTCCGCTTAAACACTGACAGCGGTCGAGTTCTTACCccccACCGATTAGTAGAAATATTT

ATCGTTTAAGGATTTGAACTCTTCGCAATACAAAATAGGGATCTAATCCCATAATTTTGACCTCAGATCAGACGAGGATA

CCCGCTGAACTTAA

>OTU_878

GAAATGCGATAAGTAATGTGAATTGCAGAATTCAGTGAATCATCGAATCTTTGAACGCACATTGCGCCCCTTGGTATTCC

GGAGGGCATGCCTGTTCGAGCGTCATTACACCACTCAAGCTATGCTTGGTATTGGGCGCTCGTCCTTAGTTCGGGCGACG

CCTTAAAGGACCTCGGCGAGGCCACTCCGGCTTTAGGCGCTAGTAGAATTTATTCGAACGTCTGTCAAAGGAGAGGAACT

CTGCCGACTGAAACCTTTATTtttCTAGGTTGACCTCGGATCAGGTAGGGATACCCGCTGAACTTAA

>OTU_879

GAAATGCGATAAGTAATGTGAATTGCAGAATTCAGTGAATCATCGAATCTTTGAACGCACATTGCGCCCATTAGTATTCT

AGTGGGCATGCCTGTTCGAGCGTCATTTCAACCCTCAGGCTCTATCTGCTTGGTGTTGGGGTTCtctctACAGTATCTGT

AGGCCCCAAaaaTCAGTGGCGGACTTGCTGAAGCTCCGAGCGTAGTAATATTTCTCGCTTAGGGTGCTGCAGTGAGCTTT

CTAGCCGTTAAATCTCCATTtttACAAGGTTGACCTCGGATCAGGTAGGAATACCCGCTGAACTTAA

>OTU_880

GAAATGCGATAAGTAGTGTAAATTGCAGAATTTAGTAAATTATTAAATCTTTAAACGCATATTACGCCCCTTAGTATTCC

GTAGGGCATGCCTGTTTAAGCGTTATTTACCTCTTAAGCTCTACTTAGTGTTAGGCGTTGTTCCGCTTCGcgcgAGAACT

CGCTCTAAAGGTATTAGTAGCGGTTGTGCTACCCCTTGCGTAGTATATTACGCTTCTTAAGGCGGTTAATTAGCGTCTAT

AAAGCTTATATTATATCTTAACCTTAGATTAGGTAGGGATACCCGCTAAACTTAA

>OTU_881

GAAATGCGATACCTAGTGCGAATTGCAGCCATCGTGAATCATCGAGTTCTTGAACGCACATTGCGCCCGTCGGTATTCCG

GCGGGCATGCCTGTCTGAGCGTCGTTTCCTTCTTTGAAGCTTttttttAAGGAAAGATTCAGAGCTGGCCGTGCCACTGG

CCCGGCCGAAAAGAAACGTTGCGGACGAAGCGAACTACATCGGGACGCTTTTGCCGCCGAGCGAAAATatatCATTGAAC

TCGACCTCAGATCAGGTAGGAGTACCCGCTGAACTTAA

>OTU_882

GAAATGCGATACGTAATGTGAATTGCAGAATTCAGTGAATCATCGAATCTTTGAACGCACATTGCACCCTCTGGTATTCC

GGggggTATGCCTGTTCGAGCGTCATTTCAACCCTCAAGCTCAGCTTGGTATTGGGTGTCACCAGACAATGGTGCACCTC

AAAATTAGTGGCGGTGCTGTCTGGCTTCAAGCGTAGTAATTCTTTTGCTTTGGAGGTTTGGATATGTGCTTGCCATCAAC

CTCTAATTTATCAAAGGTTGACCTCGGATCAGGTAGGGATACCCGCTGAACTTAA

>OTU_883

GAAATGCGATAAGTAATGTGAATTGCAGAATTCAGTGAATCATCGAATCTTTGAACGCAACTTGCGCTCCTTGGTATTCC

GAGGAGCATGCCTGTTTGAGTGTCATTAAATTCtctcGAAAGCATGTTtttAACGTGATTTTGGATTCTGGGAGCCTGCT

GGCGGGATATGAAAGCCAGCTCTCCTGAAAAGAATTAGCTGGgggCTGTGTCAATGCTTGAATCCCTctctctGGCGTGA

TAACTTGTCAAAGCCATTGGTGGGTCAGGATGGAATCCAGTCTCTGCTTTCTAATTGGCGTCTGGCTTGGCTGGACAAGA

TCAACTTTGACCTCAAATCAGGTAGGACTACCCGCTGAACTTAA

>OTU_884

GAAATGCGATAAGTAATGTGAATTGCAGAATTCAGTGAATCATCGAATCTTTGAACGCACCTTGCGCTCCTTGGTATTCC

GAGGAGCATGCCTGTTTGAGTGTCATTAAATTCTCAACCTTATTAGCTTTTGCTGATAATGGATTGGATGTGGgggTTTC

TTTTGCCGGCTTCTTCACAGAGGTCAGCTCCCCTTAAATGCATTAGCTGGTGCCCCGCGTGGACCATCTATTGGTGTGAT

AATTATCTACGCCGTGGATGTCTGCAATCATATGGGTTTTGCACTGCTTCTAACCGTCTGTTCATTCGGACAATACATGA

CAATTTGACCTCAAATCAGGTAGGGCTACCCGCTGAACTTAA

>OTU_885

GAAATGCGATAAGTAATGTGAATTGCAGAATTCAGTGAATCATCGAATCTTTGAACGCACCTTGCGCTCCTTGGTATTCC

GAGGAGCATGCCTGTTTGAGTGTCATTAAATTCTCAACCTCACCCATTTTGTGATGAAGTgtgtgtgtAGGCTTGGATGT

GGAGGCTTGCAGGCCCGGCTctctGAGTTGGTCTGCTCCTCTGAAATGCATTAGTGGGTTGTGCCCCTCATCTATTGGTG

TGATAATTATCTACGCCGTGGATCAGTGTTGGCAACAATGTGACCTGCTTCCAACCGTCCGACTCGGACAACTTGACATT

tttGGCCTCAAATCAGGTAAGACTACCCGCTGAACTTAA

>OTU_886

GAAATGCGATAAGTAGTGTGAATTGCAGAATTCAGTGAATCATCGAATCTTTGAACGCACATTGCGCCCCTTGGTATTCC

ATGGGGCATGCCTGTTCGAGCGTCATTACACCCTCAAGCTCTGCTTGGTGTTGGGCGCCTGTCCCGCCTCGCGTGTGGAC

TCGCCCTAAATGTATTGGCAGCGGTTTGCCAGCCCGTAGCGTAGCACTATTGCGCCTGCGAGCATTGGCAGACTAGCGTC

CACCAAGTGACCACCCACAGTTTGACCTCGGATCAGGTAGGGATACCCGCTGAACTTAA

>OTU_887

GAAATGCGATAAGTAATGTGAATTGCAGAATTCAGTGAATCATCGAATCTTTGAACGCACATTGCGCCCGCCAGTACTCT

GGCGGGCATGCCTGTCTGAGCGTCATTTCAACCCTCAGGGCACCCTCTCACGGgggTCGCCCTGGCGTTGGGGATCGGCC

GCCccccGCGGCgcgcCGGCCCCGAAATGCAGTGGCGGTCCGTCCCGCGACCTCCTCCGTGCAGTAGCGATACCTCGCGG

CTGGATGGCAGGTCGGGCCACGCCTCGAAACCcccAACTTCTCAAGGTTGACCTCAGATCAGGTAGGAATACCCGCTGAA

CTTAA

>OTU_888

GAAATGCAATAAGTAATGTGAATTGCAGAATTCAGTGAATTATTAAATCTTTGAACGCATATTGCGCCTGCTAGCATTCT

GGCAGGCATGCCTATTCAAGCGTCATTTCAACTCTCAAGCTCTGCTTAGTGTTAGGGCCCTACAGCTGATATAGGCCCTC

AAAGGTAGTGGCAGACCCTCCTAGAGCCTCCTTTGCGTAATAACTTTACGTCTCGCACTAGGATCTAGAGGGACTTTTGC

TGTAAAACCcccTAATTTTCTAAAGGTTAACCTCAGATCAGGTAGGAATACCTACTGAACTTAA

>OTU_889

GAAATGCGATAAGTAATGTGAATTGCAGAATTCAGTGAATCATCGAATCTTTGAACGCACCTTGCGCTCCTTGGTATTCC

GAGGAGCATGCCTGTTTGAGTGTCATTAAATTCTCAACCTTACCATCACTGATGGCAAGGCTTGGACTTGGgggTTTCAT

TTTGCAGGCTTCAACTGAAGTCGGCTCCCCTTAAATGAATTAGCCGGAAAACCCTTGTGGACCCGTCTATTGGTGTGATA

ATTATCTACGCCGCAGGATAGGACTGCACTTTTGTAATGGGATTTCTGCTTCAAACCGTCCTTTGGGACAACCATTTGAC

CATTTGACCTCAAATCAGGTAGGACTACCCGCTGAACTTAA

>OTU_890

GAAATGCGATAAGTAGTGTGAATTGCAGAATTCAGTGAATCATCGAATCTTTGAACGCACATTGCGCCCTTTGGTATTCC

TTAGAGCATGCCTGTTCGAGCGTCATTTAACCCCTCAAGCTCAGCTTGGTGTTGGGCGGCTGTCCCGCATCCcccGCCCG

GgggCCTGGGACTCGCCTCAAATCGATTGGCGGCCGGTACGTTGGCTTCGAGCGCAGCAGAAACGCGAACTCGGGGCCTG

GCGTGCTGGCTCCCACAAGCATatatCTTATTtttGACCTCGGATCAGGTAGGGATACCCGCTGAACTTAA

>OTU_891

GAAATGCGATACGTATTGTGAATTGCAGATTTTCGTGAATCATCGAATCTTTGAACGCACATTGCACCCTCTGGTATTCC

AGAGGGTATGCCTGTTTGAGCGTCATTTCtctctcAAACCTCGGGTTTGGTATTGAGTGATACTCTGTCAAGGGTTAACT

TGAAATATTGACTTAGCAAGAGTGTACTAATAAGCAGTCTTTCTGAAATAATGTATTAGGTTCTTCCAACTCGTTATATC

AGCTAGGCAGGTTTAGAAGTATTTTAGGCTCGGCTTAACAACAATAAATCTAAAAGTTTGACCTCAATCAGGTAGGACTA

CCCGCTGAACTTAA

>OTU_892

GAAATGCGATAAGTAATGTGAATTGCAGAATTCCGTGAATCATCGAATCTTTGAACGCACATTGCGCCCTCTGGTATTCC

GGAGGGCATGCCTGTCCGAGCGTCATTGCTGTACCTCAAGCCCGGCTTGtgtgtTGGGCCTACCGTCCCCTGGACGGTGA

CCCGAAAGGCAGTGGCGGCGTCGCGTCCCGGTCCTTGAAGCGTATGGGGCTTTGTCACCCGCTTGGAGGCCGGCCGACCC

CGTAGCCCATCGTGGATCTTCCACATTTATCTGATGGACCTCGGATCAGGTAGGGATACCCGCTGAACTTAA

>OTU_893

GAAATGCGATAAGTAGTGTGAATTGCAGAATTCAGTGAATCATCGAATCTTTGAACGCACATTGCGCCCCACGGCACTCC

GTGGGGCATACCTGTTCGAGCGTCATCTACACCCTCAAGCTCTGCTTGGTGTTGGGCGTCTGTCCCGCCTCTCCGcgcgc

gGACTCGCCCCAAATCCATTGGCGGCGGACCTTGCCTCCCTCTCGCGCAGCACATTGCGCTTCTCGAGGCGCGGCGGCCC

GCGTCCAGCAAGCAACCTTCACACCGTCTTTGACCTCGGATCAGGTAGGGATACCCGCTGAACTTAA

>OTU_894

GAACTGCGATAAGTAATGTGAATTGCAGAATTCAGTGAATCATCGAGTCTTTGAACGCACATTGCGCCcccTGGCATTCC

GGggggCATGCCTGTCCGAGCGTCATTGCTGCCCATCAAGCCCGGCTTGtgtgtTGGGTCGTCGTCCcccccGGgggACG

GGCCCGAAAGGCAGCGGCGGCACCGTGTCCGGTCCTCGAGCGTATGGGGCTTTGTCACCCGCTCGACTAGGGCCGGCCGG

GCGCCAGCCGACGTCTCCAACCATTtttCTTCAGGTTGACCTCGGATCAGGTAGGGATACCCGCTGAACTTAA

>OTU_895

GAAATGCGATAAGTAATGTGAATTGCAGAATTCAGTGAATCATCGAATCTTTGAACGCACATTGCGCCCGGTGGTATTCC

GCCGGGCATGCCTGTTCGAGCGTCATTTCACCACTCAAGCCTGGCTTGGTATTGGGCGTCGCGGAGTCCGcgcgcCTCAA

AGTCGTCCGCCGAGCCGTCCGTCTCCGAGCGTTGTGACTTCACTGTTTCGCTCTCGAGGTCGGGCgcgcgcgcTGAGACT

TCTTttttACAGGTTGACCTCGGATCAGGTAGGGATACCCGCTGAACTTAA

>OTU_896

GAAGTGCGATAAGCAATGCGAATTGCAGAACCGTGAGTCATCAGATTtttGAACGCATCTGGCGCCGGCTGGGTCTCCAG

TCGGCATGCTTGTTTCAGTGTCTTGTTTTCTCCTCACCCAAATCTTAATGCGAgagaTGCCCTTCTCTTGCCAAGCAAGA

AAGCACTCTGCGCTCTGCGAGCGGCTTCGACTCGATCGAGGGCCACACTCAATGCAGCAGTCACATTGCTCACAATGTGA

ACTCATTGAGAGCAGAGGCAGTGCAGCAGCCGCTGCCAGTGCTTAGTCAcacaAACTTTCTTTGCATCTGAAATCAAGCA

GGACCACCCGCTGAACTTAA

>OTU_897

GAAATGCGATAAGTAATGTGAATTGCAGAATTCAGTGAATCATCGAATCTTTGAACGCACATTGCGCCCGTCAGTATTCT

GGCGGGCATGCCTGTTCGAGCGTCATTACGCCCCTCAAGTCCCCTGTGGACTTGGTGTTGGGGATCGGCGAGGCTGGTTT

TCCAGCACAGCCGTCCCTTAAATTAATTGGCGGTCTCGCCGTGGCCCTCCTCTGCGCAGTAGTAAAACACTCGCAACAGG

AGCCCGGCGCGGTCCACTGCCGTAAAACCccccAACTTtttATAGTTGACCTCGAATCAGGTAGGACTACCCGCTGAACT

TAA

>OTU_898

GAAATGCGATAAGTAGTGTGAATTGCAGAATTCCGTGAATCATCGAATCTTTGAACGCACATTGCGCCTCCTGGCATTCC

TGGgggCATGCCTGTTCGAGCGTCATTAAAAGGCCTTCAGGCGAGCTTGAGTCGCCTGGTCCTGAAGGAATGGTCACCTT

TCGATTGCCCATCCcccGAAACCCAACGGCGGACAACTCCCGGTGGAGCTGCTGGCGTGGTGAGGAATACTTTCGTCAGT

GGTCTTCGAGGGACAGTCCTGCCGGTTGAACCcccAcacaTTCCAACGGTTGACCTCGGATCAGGTAGGGATACCCGCTG

AACTGAA

>OTU_899

CAAATGGTGGGCGCTTTGACGGTGTGGTTGGTGTTCTAGCTGGGTTCGAGGTTCTGGAAACATTGGAAGACCATAGTATT

GAGACCGCACAACCCATCGAAGTTGCTGTTTGGACCAATGAAGAAGGAGCCCGGTTTCATCCTGCAATGATGGGGTCTGG

GGTTCATTGCGGCATTCAATCACTCGATGAAGCGCTGGCAACAGAAGATATGGCCGGCATCAGCGTTCGGGATGAACTCA

GGAAGAATGGCTATCTCGATGGCCTTTCGCCTGGGTCTCACGCAATCGACAAATatatCGAGCT

>OTU_900

GAAATGCGATACGTAATGTGAATTGCAGATTTCAGTGAATCATCGAATCTTTGAACGCACATTGCGCCCGCCAGTATTCT

GGCGGGCATGCCTGTTCGAGCGTCATTTCAACCATCAAGCCTTCGCTTGTGTTGGGGCCCCGCGGCTGCCGCGGTCCCCT

AAATCCAGTGGCGGGCTCGTCGTCGTACCGAGCGCAGTAGACATCCTCGCTCCGGGAACCCGACGGGTGCCGGCCGTGAA

ACCccccACATCAAGGTTGACCTCGGATCAGGTAGGACTACCCGCTGAACTTAA

>OTU_901

GAAATGCGATAAGTAGTGTAAATTGCAGAATTCAGTGAATTATTAAATCTTTAAACGCACATTGCGCCCCTCGGTATTCC

GTAGGGCATGCCTATTCGAGCGTCATTTACCCCTTAAGCTCTGCTTAGTGTTAGGCGTTGTCCCGCTTCGcgcgAGGACT

CGCCCTAAAGGTATTAGCAGTAGTCGTGCCACCCCTCGCGCAGCATATTACGCTTCTTAAGGCGGTTAATTAGCGTCCAC

AAAGCTTACATTACATCTTAACCTCGGATTAGGTAGGGATACCCGCTGAACTTAA

>OTU_902

GAAATGCGATAAGTAATGCGAATTGCAGACTTCAGTGAATCATCGAATCTTTGAACGCACATTGCGCCCGCCAGTATTCT

GGCGGGCATGCCTGTCCGAGCGTCACTACACCACTCAGCACCcccTAACCCTGGggggCTGGCGTTGGgggATCGGCCGC

CCTCCGCGGCGCCCGTCCcccAAATCCAGTGGCAGTCCGGCGTCGTTTCCCCTGCGCAGTAGCAATGCGACGCAGAAGGT

CTCCCGTCGGCGCTCGCCGTAGTACCCCGACTTTATTCTAAAGTTGACCTCGGATCAGGTAGGACTACCCGCTGAACTTA

A

>OTU_903

AAATTGCGATAACTAGTGTGAATTGCAAATTCAGTGAATCATCGAGTCTTTGAACGCATCTTGCGCTCATTGGTATTCCA

GTGAGCACGCCTGTTTCAGTATCAAaaaCAACCCTCATTCAAATTtttttttGAATGGTCATGAAGGAAGCTACTTGTTG

GCGACCTTTTAAATTGAGTAAGGCCTGAATTTGTTTATCTAGCCTGAACTTttttttAATATAAAGGAAAGCTCTTTGCG

ACTTGAACTTTGTTGGGGCCTCCCAAATAAAGCTTtttCATCTTGATCTGAAATCAGGTGGGACTACCCGCTGAACTTAA

>OTU_904

AAAATGCGATAAGTAATGTGAATTGCAGAATTCAGTGAATCATCGAATCTTTGAACGCACATTGCGCCCGCCAGTATTCT

GGCGGGCATGCCTGTCTGAGCGTCATTTCAACCCTCAGGACCCGTTCGCGGGACCTGGCGTTGGGGATCAGCCTGCCCCT

GGCGGCGGCTGGCCCTGAAATCCAGTGGCGGTTCCCTCGCGAACTCCTCCGTGCAGTAATTAAACCTCTCGCGGCAGGAT

AGCGGTTGAACCACGCCGCTTAAACCccccACTTCTTAAAGGTCCGACTTCAGATCAGGTAGGAATACCCGCTGAACTTA

A

>OTU_905

GAAATGCGATAAGTAATGTGAATTGCAGAATTCAGTGAATCATCGAATCTTTGAACGCACATTGCGCCCGCCAGCACTCT

GGCGGGCATGCCTGTTCGAGCGTCATTTCAACCACCAAGCCCTGCTTGTGTTGGGGCCCTGCGGCTGCCCGCAGGCCCTG

AAAACCAGTGGCGGGCTCGCTAGTCACACCGAGCGCAGTAAGCACATTCTCGCTCAGGGCGTGCGGCGGGTTCTTGCCGT

GAAACCcccccTGTAACTCACAAGGTTGACCTCGGATCAGGTAGGAATACCCGCTGAACTTAA

>OTU_906

GAAATGCGATAAGTAATGTGAATTGCAGAATTCAGTGAATCATCGAATCTTTGAACGCACCTTGCGCTCCTTGGTATTCC

GAGGAGCATGCCTGTTTGAGTGTCATTAAATTCTCAACCTTATTAGCTTTTGCTGATAATGGCTTGGACTTGGgggTCTT

ttttGCTGGCTTTCATTAGTCTGCTCCCCTTAAATGTATTAGCCGGTGCCCCGCAGTGGAACCGTCTATTGGTGTGATAA

TTATCTACGCCGTGGACGTCTGCTATAATGGGTTTGCGCTGCTTCTAACCGTCtctcGGGACAACACAAATGACAATTTG

ACCTCAAATCAGGTAGGACTACCCGCTGAACTTAA

>OTU_907

GAAACGCGATAGTTAATGTGAATTGCAGAATTCAGTGAATCATCGAGTCTTTGAACGCACATTGCGCCcccTGGTATTCC

GGggggCATGCCTGTCCGAGCGTCATTGCTGCCCATCAAGCGCGGCTTGtgtgtTGGGCCACCGTCCccccTCGGggggA

CGGGCCCGAAAGGCAGCGGCGGCACCGCGTCCGGTCCTCGAGCGTATGGGGCTCTGTCACCCGCTCTGGAGGCCTGGTCG

GCGCCTGCCGACCCCAACCTTttttAACCAGGTTGACCTCGGATCAGGTAGGGATACCCGCTGAACTTAA

>OTU_908

GAAATGCGATAAGTAATGTGAATTGCAGAATTCAGTGAATCATCGAATCTTTGAACGCACCTTGCGCCCTTTGGTATTCC

GAAGGGCATGCCTGTTTGAGTGTCATGAAACCTCACTCCACTTGGGTTtttGCCTGAGTGGTAGTGTATTGGGTGTTGCC

TTGCCAAAGGCTCGCCTTAAAGAATAAAGCACCTTGGATGTAATACGTTTCATCCTTCTGGGTGGCTAATAACCCCAGCA

TAACTCACTGATCTGGCCTCAAATCAGGTAGGGCTACCCGCTGAAACTTAA

>OTU_909

GAAATGCGATAAGTAGTGTGAATTGCAGAATTCAGTGAATCATCGAATCTTTGAACGCACATTGCGCCCCTTGGTATTCC

ATGGGGCATGCCTGTTCGAGCGTCATTTGTACCCTCAAGCTCTGCTTGGTGTTGGGTGTTTGTCCTTTCCATTGCGTTGG

GACTCGCCTTAAAGTCATTGGCAGCCGGTGTATTGGTCTTGAAGCGCAGCACAATTTGCGGTTCCAGCCAAaaaCGCCAG

CCTCCATTAAGTACATTTAACACTTTTGACCTCGGATCAGGTAGGGATACCCGCTGAACTTAA

>OTU_910

GAAATGCGATAAGTAGTGTGAATTGCAGAATTCAGTGAATCATCGAATCTTTGAACGCACATTGCGCCCTTTGGTATTCC

TTAGGGCATGCCTGTTCGAGCGTCATCTAAACCTTCAAGCCCTGCTTGGTGTTGGGTGCCTGTCCCGCCTCCGcgcgTGG

ACTCACCTCAAATCCATTGGCGGCCcccGCGTCGGCCACGAGCGCAGCAGAAACGCGAACTCGTGAGCCCGCCCGGGCGG

CTCCCACGAAGCTAACCcccAACTTtttGACCTCGGATCAGGTAGGGATACCCGCTGAACTTAA

>OTU_911

GAAATGCGATAAGTAATGTGAATTGCAGAATTCAGTGAATCATCGAATCTTTGAACGCACATTGCGCCCGCCAGCACTCT

GGCGGGCATGCCTGTCCGAGCGTCATTTCAACCCTCAGGCCccccGTCCGCGGGGAACCGGCCTGGCGTTGGGGCTCAGC

GGCCTTCACCGGCCCCTGTCCCCGAAATTCAGTGGCGGTCGCGCCGCAGCCTCCCCTGCGTAGTAGCACACCTCGCACCG

GAGAGCGGCACGGCCACGCCGTAAAACCccccAGTTTTACCAAGGTTGACCTCGGATCAGGTAGGAATACCCGCTGAACT

TAA

>OTU_912

GAAATGCGATAAGTAGTGTGAATTGCAGAATTCAGTGAATCATCGAATCTTTGAACGCACATTGCACCTCTCGGTATTCC

GGGAGGTATGCCTGTTCGAGCGTCATTTGTACCCTCAAGCTCTGCTTGGTGTTGGGTGTTTGTTCCGCCTTTTGCGCGTG

AACTCGCCTTAAAACAATTGGCAGCCGGCGTACTTGTTTCGGAGCGCAGCACATTTTGCGCCCCAGATCAGCGTATGTTG

GCATCCAAGAAGCCTTATTTTAACGCTCTTGACCTCGGATCAGGTAGGGATACCCGCTGAACTTAA

>OTU_913

GAAATGCGATAAGTAATGTGAATTGCAGAATTCAGTGAATCATCGAATCTTTGAACGCACATTGCGCCcccTGGTATTCC

GGggggCATGCCTGTTCGAGCGTCATTATCAACCATCAAGCTCTGCTTGGCATTGGGCGTCCGTCCcccGGGACGCGCCC

TAAAGATGTAGGCGGTGACCCCGAGTCTCAAGCGTAGTACAATCTTTAGCTTGGAAGACCCGGCGGCTGCTCGCCCCTAG

AACCCATCACATCAAGGTTGACCTCGGATCAGGTAGGGATACCCGCTGAACTTAA

>OTU_914

GAAATGCGATAAGTAATGTGAATTGCAGAATTCAGTGAATCATCGAATCTTTGAGCGCACATTGCGCCCGCTAGTATTCT

GGCGGGCATGCCTGTTCGAGCGTCATTTCAACCATCAAGCCCTAGGCTTGTGTTGGGGCCCTGCGGCCGTCCGCAGCCcc

cGAAAAGCAGTGGCGGGCTCGCTGTCACACCGAGCGCAGTAGTATATCTTCGCTAGGACGTGATTGCGGATTCCGGCCGT

TAAACACCCCAATTTACCAAGGTTGACCTCGGATCAGGTAGGAATACCCGCTGAACTTAA

>OTU_915

GAAATGTGATAAGTAATGTGAATTGCAGAATTCAGTGAATCATCGAATCTTTGAACGCATCTTGCACTCCTTGGTATTCC

GAGGAGTATGTCTGTTTGAGTGTCATGAATTCTTCAACCCTctctTTTCTTAGTGAATCGAGAGGTGTTTGGATTCTGAG

TGTTGCTCCTTACCCGGGCTCATTCGTAATGCATTAGCATCCATATTCGAATTTCGGATTGACTTGGCGTAATAGACTAT

TCGCTGAGGAATCTAACTTCGGTTAGAGCCGGATTTGAACTAGGAAGCTTATAATCTAGCTTAGTCTACTTTAAGTTTAG

ATCTCAAATCAGATAGGATTACCCGCTGAACTTAA

>OTU_916

GAAATGCGATAAGTAATGTGAATTGCAGAATTCAGTGAATCATCGAATCTTTGAACGCACATTGCGCCCTGTGGTATCCC

GCAGGGCATGCCTGTTCGAGCGTCATTTCAACCCTCAAGCTCTGCTTGGTGTTGGGCTCCGCTGCTAACCCGGCGGGCCT

TAAAATGAGTGGCGGCGCCATCGGGCTCTGAGCGTAGTAAATTTCCTCGCTACGGAGTCCTGGTGACGGTGGCCATCAAC

CcccAACTTTCTACGTTTGACCTCGGATCAGGTAGGGATACCCGCTGAACTTAA

>OTU_917

GGAATGTGATAAGTAATGTGAATTGCAGAACTTAGTGAATTATCAAATCTTTAAATGCATATTGCGCCTGCTAGCATTCT

AGCAGGCATACCTATTCAAGCATCATTTCAACCCTCAAGCTCCACTTAGTGTTAGAGCCCTACAGCTGATATAGGCCCTC

AAAAGTAGTAGCAGACCCTCCTAGAGCCTCCTTTGCGTAGTAACTTTACATCTCACACTAGGATCTAGAGGGACTCTTGC

TGTAAAACCccccAATTTTCCAAAGGTTAACCTCAGATCAAGTAGGAATACCTGCTGAACTTAA

>OTU_918

GAAATGCGATAAGTAGTGTGAATTGCAGAATTCAGTGAATCATCGAATCTTTGAACGCACATTGCGCCCCTTGGTATTCC

ATGGGGCATGCCTGTTCGAGCGTCATTTGTACCCTCAAGCTCTGCTTGGTGTTGGGTGTTTGTCTATGCTTTTGCTTGGA

CTCACCTCAAAGCAATTGGCAGCCAGTGTTTTGGCATAGAAGCGCAGCACATCTTGCACTTCCAGTCTTAAACACCAGCT

TCCAGTAAGTCCCTTTACGTTTGACCTCGGATCAGGTAGGGATACCCGCTGAACTTAA

>OTU_919

GAAATGCGATAAGTAGTGTGAATTGCAGAATTCAGTGAATCATCGAATCTTTGAACGCACATTGCGCCCTTCGGTATTCC

GTTGGGCATGCCTGTTCGAGCGTCATTTAATCATTCAAGCTCTGCTTGGTGTTGGGTGTTTGTTCCGCCTCAGCGCGTGG

ACTCGCCTTAAATTCATTGGCAGCCGGTATGTTGGCTTCGTGCGCAGCACATTGCGTCGCGATTCTGGCAGGCCTCCTCC

CATTAAGCTTCTTTTAAGTTTGACCTCGGATCAGGTAGGGATACCCGCTGAACTTAA

>OTU_920

GAAATGCGATAAGTAATGTGAATTGCAGAATTCAGTGAATCATCGAATCTTTGAACGCACATTGCGCCCATTAGTATTCT

AGTGGGCATGCCTGTTCGAGCGTCATTACAACCCTTAAGCCTAGCTGCTTAGCGTTGGGAACTTAGCCTCCAAAGCTAAT

TCCTTAAAGACATTGGCAGAGTTGCGGCATACTCTAAGCGTAGTAATTTATTCTCGCTTCTGAAGGTGTCGTGACGATCG

CCACAAACCTCTATATTTCTAGTGGTTGACCTCGGATCAGGTAGGAATACCCGCTGAACTTAA

>OTU_921

GAAATGCGATAAGTAATGTGAATTGCAGAATTCAGTGAATCATCGAATCTTTGAACGCACCTTGCGCTCCTTGGTATTCC

GAGGAGCATGCCTGTTTGAGCGTCGTGAATCtctcAACCCACGAATTTATTtttGtgtgGCTTGGAATTGGAGGCTTTGC

TGGCGACGGTCAGCTCCTCTCAAATGCATTAGCTGGGCTTTAGCTCGGAGGAACGGTCGGTGTGATAATTATCTGCGCCT

TAACCTACTACTTCGGATCCCGTTTCTAATCGTCCGCAAGGACACTATTTCATGAATCTGACCTCAAATCAGGTAGGACT

ACCCGCTGAACTTAA

>OTU_922

AAAATGCGATAAGTAATGTGAATTGCAGAATTCAGTGAATCATCGAATCTTTGAACGCACATTGCGCCCGCCAGTATTCT

GGCGGGCATGCCTGTTCGAGCGTCATTTCAACCCTCAAACCcccGGGTTTGGTGTTGGGGATCGGGCTGCCAGTCAGCCC

GGCCCCGAAATCTAGTGGCGGTCACGCTGCAGCTTCCATTGCGTAGTAGATAACACCTCGCAACTGGTACGCGGCGCGGC

CAAGCCGTTAAACCcccAACTTCTGAATGTTGACCTCGGATCAGGTAGGAATACCCGCTGAACTTAA

>OTU_923

GAAATGCGATAAGTAATGTGAATTGCAGAATTCAGTGAATCATCGAATCTTTGAACGCACATTGCGCCCCTTGGTATTCC

GAGGGGCATGCCTGTTCGAGCGTCATTACAACCCATCAAGCCCTGCTTGGCCTTGGGCGCCGCCcccGCGAGGGGACGCG

CCCCAAAGATCTCAGCGGTGCCGCGAGGCCCCGAGGCGTAGTAAaaaCAACTATCCCGCTTCGTGGGGCCCTTCGGTCGC

CCGCTTCCAATGACCAACACCTTCTAGGTTGACCTCGGATCAGGTAGGGATACCCGCTGAACTTAA

>OTU_924

GAAATGCGATAAGTAATGTGAATTGCAGAATTCCGTGAATCATCGAATCTTTGAACGCACATTGCGCCCCTCGGTATTCC

GGgggCATGCCTGTTCGAGCGTCATTACACCAATCAAGCCCGGCTTGGTCTTTGGGCGTCGCGGTCTCCCGCGTGCCTCA

ATGTCGCCGGCTGGTGCGACCGTCTCTAAGCGTTGTGAATCAACAGTCGCTCCGGAGGTCGGTCGGGCTGCGCCGTCAAG

CCTTttttACAGGTTGACCTCGGATCAGGTAGGGATACCCGCTGAACTTAA

>OTU_925

GAAATGCGAAAAGTAGTGTGAATTGCAGAATTCAGTGAATCATCGAATCTTTGAACGCACATTGCGCCCCTTGGTATTCC

ATGGGGCATGCCTGTTCGAGCGTCATTTGTACCTTCAAGCATTGCTTGGTGTTGGGTGTTTGTCCGCGTTTCTACACGCG

GACTCGCCTTAAAGTGATTGGCAGCCGGCGTACTCGCCTCGGAGCGCAGCACATTTTGCGCCCTTGACCTGAACGATGGC

GTCCACTAAGCCTATACTTTTGCTCTTGACCTCGGATCAGGTAGGGATACCCGCTGAACTTAA

>OTU_926

GAAATGCGATAAGTAATGTGAATTGCAGAATCAGTGAATCATCGAGTCTTTGAACGCAACTTGCGCCCTTTGGTATTCCG

AAGGGCATGCCTGTTTGAGTGTCATGAAAACCTCAATCCCTCGGGTTTCTTAGCGAACCCGTTGGACTTGGATCTGGGTG

TCTGCCGCGACCCTCCGGGGACGTCGCTCGCCTTAAATGTGTTAGTTGGACGGTGACTGCCCGTCAGCCTGGCGTAATAA

GTTTCGCTGGGCCTTTGGGGCTTGGTCAGTCGGCCTGCTCATAACAACCAACTTTCTATGACTCTGACCTCAAATCAGGT

AGGGCTACCCGCTGAACTTAA

>OTU_927

GAAATGCGATAAGTAGTGTGAATTGCAGAATTCAGTGAATCATCGAATCTTTGAACGCACATTGCGCCCCTTGGTATTCC

ATGGGGCATGCCTGTTCGAGCGTCATTTGTACTCTCAGGCCTTGCTTGGTGTTGGGTGTTTGTCTATGCTTAGCCTAGAC

TCGCCTTAAATatatTGGCAGCCGGTATACTGGCTTGAAGCCCAGCACAATTGCAATTCAGGTTGGCTTATTAGCATCCA

TAAAGATCATCAACACTTTTGACCTCGGATCAGGTAGGGATACCCGCTGAACTTAA

>OTU_928

GAAATGCGAAAAGTAGTGTGAATTGCAGAATTCAGTGAATCATCGAATCTTTGAACGCACATTGCGCCCCTTGGTATTCC

ATGGGGCATGCCTGTTCGAGCGTCATTTGTACCCTCAAGCTCTGCTTGGTGTTGGGTGTTTGTCCCGCTATTGCGTGTGG

ACTCGCCTTAAAGCAATTGGCAGCCGGCATATTGGCCTGGAGCGCAGCACAATTTGCACTTCTTGTCTTTGAATGTCGAG

CATCCAGAAGCAACTCTTtttGCTTTTGACCTCGGATCAGGTAGGGATACCCGCTGAACTTAA

>OTU_929

GAAATGCGATACGTAATGTGAATTGCAGAATTCCGTGAATCATCGAATCTTTGAACGCATATTGCGCCTTCTGGTACTCC

AGGAGGCATGCCTGTCTGAGCGTCATTACATCATTTAAACCAACTTGTTGTGTTTAGACTTGGGAGTTTGATTACTGAAC

TGGCATTTGCTGGTTTGCTGTATATCTCTTCTGTAATGTATTGATAGCGATACAAGCCTTCAGTCGTAACAAGTTTACTT

tttGTTTACATTCGACTTTGATTGCCTGTGTTACATTGTCTATTTCTTTTCGACATGGTTTTGCCATTGTCAACCTAAAG

ATTTGACCTCAGATCAGGTAGGAATACCCGCTGAACTTAA

>OTU_930

GAAATGCGATAACTAGTGTGAATTGCAGAATTCAGGGAATCATCGAGTCTTTGAACGCACATTGCAGCCGCCGGCATTCC

GGCGGCTATGCCTGTCCGAGCGCTACTAACACCCCTCAGGCCcccGTGCCTGGCGTTGGggggCCGCTAATCTTGGGCGG

CCCTCTCAAAGTTAGTGGCGGCAGCGCCCGCGGCGCGACGCGCAAAGTAGCACTACTGGCGCGACGTCCCCGGCGCTGAC

CAGCCGAgagaACCTCACTACCAcacaAAGTAGTTGGCCTCGGATCAGGTAGGATTACCCGCTGAACTTAA

>OTU_931

GAAATGCGATAACTAGTGTGAATTGCAGATTTACGTGAATCATCGAGTCTTTGAACGCACATTGCGCTCCTTGGTTTTCC

AGGGAGCATGTCTATCCGAGCGTCGTTGCAACTCTCAAGCCTGGCTTGGTCCTGGCAGGCGTCCCGCTCCGGCGGGGCCC

CGCCCAAACGCAACTCACGTCGTGCCAACTCGGTCGGAGCGCAGCAGATTTGCAGCCCCGGCTGGCCGCGTCGCGAGCCG

CCcccGTGCGGACTTCTCAAGGTTGACCTCGGATTAGATAAGGATACCCGCTGAACTTAA

>OTU_932

GAAATGCGATAAGTAATGTGAATTGCAGAATTCAGTGAATCATCGAATCTTTGAACGCACATTGCGCTCCCTGGTATTCC

GGGGAGCATGCCTGTTCGAGCGTCATTACACCACTCAAGCACTGCTTGGTATTAGGCCATCGTCCcccGAAAGGTGGGCG

TGCCTCAAACACCTCGGCGGAGCCTCACCGGCTTTGGGCGTAGTAGAATTTCTCAACGTCTTATAAGTACCGGTCTGACT

CCTTTGCCGTTAAACCCCAAACTTTTAAAGGTTGACCTCGGATCAGGTAGGGATACCCGCTGAACTTAA

>OTU_933

GAAATGCGATAAGTAATGTGAATTGCAGAATTCAGTGAATCATCGAATCTTTGAACGCACATTGCGCCCTTTGGTATTCC

GAAGGGCATGCCTGTTCGAGCGTCATTTCACCACTCAAGCCTGGCTTGGTATTGGGCGTCGCGGTTTTCCGcgcgcCTTA

AAGTCTTCCGGCTGAGCTGTCCGTCTCTAAGCGTTGTGGATTtttCAATTCGCTTCGGAGTGCGGATGCCGCGGCCGTTA

AATCTTTATTCAAAGGTTGACCTCGGATCAGGTAGGGATACCCGCTGAACTTAA

>OTU_934

GAAATGCGATAAGTAATGTGAATTGCAGAATTCAGTGAATCATCGAGTCTTTGAACGCACCTTGCGCCCTCTGGTATTCC

GGAGGGCATGCCTGTTTGAGTGTCATGAAACTCTCAACCccccGGGTTttttGATCCGTCGGGTTGGACTTGGGTGTTGC

CGCTTCACTGCCGGCTCGCCTTAAAGGTCTTAGTGGGAATAGTTGGACCCATAGCTTGGCGTAATAAGTTTCGCTGGGCT

CGTGGCCGACGACCGTCCGCTTACAACAACCAATACTTTTATGATCTGACCTCAAATCAGGTAGGGCTACCCGCTGAACT

TAA

>OTU_935

AAAATGCGATAAGTAGTGTAAATTACAGAATTTAGTAAATTATCAAATCTTTAAACGCACATTGCGCCCCTTAGTATTCT

ATAGAGCATGCCTGTTCAAGCGTTATTTGTACCCTTAAGCTCTGCTTAGTGTTAGGTGTTTATCTCCTCTAGGAGACTCG

CCTTAAAACAATTAGTAGCTAGCATATTAGTATTAGAGCGCAGCACAAGTCGCGCTTCTGTCTATGTTTATTAGCAGCTA

GCAAGACCACTTtttACTCTTAACCTTAGATTAGGTAGGGATACCCGCTAAACTTAA

>OTU_936

GAAATGCGATAAGTAATGTGAATTGCAGAATTCCGTGAATCATCGAATCTTTGAACGCACATTGCGCCCGGTGGCATTCC

GCCGGGCACGCCTGTCCGAGCGTCATTGCAACAATCAAGCCTGGCTTGGTGTTGGgggCCCGTCGCGCCTGGCGCGACCc

ccccGGAACCCGTTGGCGGTGGCTctctGCCCCGATCGTCGCGCATGTTACGACGGGACGGAGGGCCACCCGCCGAACCc

cccACGACGCGAAGTTTGACCTCGGATCAGCGGGGATACCCGCTGAACTTAA

>OTU_937

GAAATGCGATAAGTAGTGTGAATTGCAGAATTTAGTAAATTATCGAATCTTTAAACGCATATTGCGCCCCTCGGTATTCC

GTAGAGCATGCCTGTTCGAGCGTTATTTACCTCTTAAGCTCTGCTTGGTGTTAGGCGTTATCCCGCTTCGCGCAAGGACT

TGCCCCAAAGGTATTAGTAGCGGTTGTGCCACCCCTCGCGCAGCACATTGCGCTTCTTAAGGCGGTTGATTAGCGTCTAT

AAAGCTTACATTATATCTTAACCTCGGATTAGGTAGGGATACCCGCTAAACTTAA

>OTU_938

GAAATGCGATACCTGGTGTGAATTGCAGAATCCCGCGAACCATCGAGTCTTTGAACGCAAGTTGCGCCCGAGGCCACTCG

GCCGAGGGCACGCCTGCCTGGGCGTCACGCCAAAACACGCTCCCAACCACCCTCATCGGGAATCGGGACGCGGCATCTGG

TCCCTCGTCTCGCAAGGGGCGGTGGACCGAAGATCGGGCTGCCGGTGTACCGCGCCGGACACAGCGCATGGTGGGCGTCC

TCGCTTTATCAACGCAGTGCATCCGACGCGCAGCCGACATTATGGCCTCAGAACGACCCAGCAAACGAAGCGCACGTTGC

TTCGACCGCGACCCCAGGTCAGGCGGGACTACCCGCTGAGTTTAA

>OTU_939

GAAATGCGATAAGTAATGTGAATTGCAGAATTCAGTGAATCATCGAATCTTTGAACGCACATTGCGCCcccTGGTATTCC

GGggggCATGCCTGTTCGAGCGTCATTACAACCCTCAAGCTCTGCTTGGTATTGGGTGTCACCcccGGGTGCGCCTTAAA

ATCAGTGGCGGTGCCGTCTGGCTTCAAGCGTAGTAATACTTCTCGCTTTGGAGTCCGGGCGAGCGTCCTGCCAAAACCcc

cATATTttttCAGGTTGACCTCGGATCAGGTAGGGATACCCGCTGAACTTAA

>OTU_940

GAAATGCGATACTTGGTGTGAATTGCAGAATCCCGTGAACCATCGAGTCTTTGAACGCAAGTTGCGCCCGAAGCCATTAG

GCCGAGGGCACGTCTGCCTGGGTGTCACGCATCGTGTTGCCCTTGACCAAACATCTTCTTAGGAGATATTTCGGTTTAGG

GGCGGATATTGGCCTCCCGTGCCTTGTTCGCGGTTGGCTCAAaaaTGAGTCTTTGGTGATGGACATCGTGACATCGGTGG

TTGTAACAAGACCTTCTTGTGTTGTCACGTATGCTTGTCACAACAGTTAGCTCAAGGGCCCTTAGACGCCATAAaaaTGt

gtgCGTTTCGATTGTGACCCCAGGTCAGGCGGGACTACCCGCTGAGTTTAA

>OTU_941

GAAATGCGATAAGTAATGTGAATTGCAGAATTCAGTGAATCATCGAATCTTTGAACGCACATTGCGCCcccTGGTATTCC

GGgggCATGCCTGTTCGAGCGTCATTTCACCACTCAAGCCTCGCTTGGTATTGGGGCCAACGGCGGTCCGCCGACGTGCC

GTCAAATCGTCCGGCTGGGTCTTCTGTCCCCTAAGCGTTGTGGAAACTATTCGCTAAAGGGTGTTCGGGAGGCTACCGCC

GTAAAACAACCCCATTTCTAAGGTTGACCTCGGATCAGGTAGGGATACCCGCTGAACTTAA

>OTU_942

GAAATGCGATAAGTAATGTGAATTGCAGAATTCAGTGAATCATCGAATCTTTGAACGCACATTGCGCCCGCCAGCATTCT

GCGGGCATGCCTTGTTCGAGCGTCATTTCAACCCTCAAGCTCCGCTTGGTGTTGGGGCCCTACAGCTGATGTAGGCCCTC

AAAGGTAGTGGCGGACCCTCCTGGAGCCTCCTTTGCGTAGTAACTTTACGTCTCGCACTGGGATCCGGAGGGACTCTTGC

CGTAAACCccccAATTTTCCAAAGGTTGACCTCGGATCAGGTAGGAATACCCGCTGAACTTAA

>OTU_943

AAAATGCGATAAGTAGTGTAAATTGCAGAATTCAGTGAATCATCAAATCTTTGAACGCACATTGCGCCCCTTAGTATTCC

ATAGGGCATGCCTGTTCAAGCGTCATTACACCCTCAAGCTCTGCTTAGTGTTAGGCGCCTGTCCCGCCTTGTGCGTAGAC

TCGCCCTAAATGTATTAGCAGCAGCTTGCCAGCCCGTAGCGTAGCACTATTGCGCCTGCGAGCTTTAGCAGTTTAGCGTC

CACTAAGTAACCACCCACAGTTTAACCTCAGATCAGGTAAGGATACCCGCTGAACTTAA

>OTU_944

GAAATGCGATAAGTAATGTGAATTGCAGAATTCAGTGAATCATCGAATCTTTGAACGCACATTGCGCCCCTTGGTATTCC

GGAGGGCATGCCTGTTCGAGCGTCATTACACCACTCAAGCTATGCTTGGTATTGGGTGTCGTCCTTAGTTGGGCgcgcCT

TAAAGAACCTCGGCGAGGCCTCCCCGGCTTTAGGCGTAGTAGAATTTATTCGAACGTCTGTCAAAGGAGAGGAACTCTGC

CGACTGAAAACCTTTTATTtttACAGGTTGACCTCGGATCAGGTAGGGATACCCGCTGAACTTAA

>OTU_945

GAAATGCGATAAGTAATGTGAATTGCAGAATTCAGTGAATCATCGAATCTTTGAACGCACATTGCGCCCTTTGGTATTCC

AAAGGGCATGCCTGTTCGAGCGTCATTTGTACCTTCAAGCTTTGCTTGGTGTTGGGCGTTtttGTCTCCCTCTTTCTGGG

AGACTCGCCTTAAAACGATTGGCAGCCGGCCTACTGGTTTCGGAGCGCAGCACATAATTTGCGCTTTGTATCAGGAGAAA

GGACGTAATCCATCAAGACTCTACATTtttAACTTTTGACCTCGGATCAGGTAGGGATACCCGCTGAACTTAA

>OTU_946

GAAATGCGATAAGTAATGTGAATTGCAGAATTCAGTGAATCATCGAATCTTTGAACGCACCTTGCGCTCTTTGGTATTCC

GAAGAGCATGCCTGTTTGAGTGTCATTAAATTCTCAACCTCTACTCGGTTTTGCGACTGATGTTAGTAGGCTTGGACTTG

GAGGCTGCCGGCATCTGTAAaaaGAAGTCAGCTCCTCTTAAAAGTATTAGCAGTAACTAGATAACTGTTCCTTGATGTGA

TAATTATCTACGTCTTGGTCAATGGAAATCATTATAACGTTATGCTTCTAACTGTCTGTTAACTCAGACTGTTTGCTTGT

CAAACATTATGACTCTTGACCTCAAATCAGGTAGGACTACCCGCTGAACTTAA

>OTU_947

GAAATGCGATAAGTAATGTGAATTGCAGAATTCAGTGAATCATCGAATCTTTGAACGCACCTTGCGCTCCTTGGTATTCC

GAGGAGCATGCCTGTTTGAGTGTCATTAAATTCTCAACCTCACCCGTTTTCTGAACGGGTCGAGGCTTGGATGTGGgggT

TTGTGCAGGCTGCCTTCACTGGCGGTCTGCTCCCCTGAAATGCATTAGTGAGGTTCATGCTGAACTTCCGTCTATTGGTG

TGATAATTATCTACGCCGTGGACGAGGATCAGACTCGCTTCTAACCGTCCGCGAGGACAATACCTTGACAATTTGACCTC

AAATCAGGTAGGACTACCCGCTGAACTTAA

>OTU_948

GAAATGCGATAAGTAGTGTAAATTACAGAATTTAGTGAATCATCAAATCTTTAAACGCATATTACGCCCCTCGGTATTCC

GTAGGGCATGCCTGTTCGAGCGTTATTTACCCCTTAAGCTCTGCTTAGTATTAGGCGTTATCCCGCTTCGcgcgAGAACT

CGCCCTAAAGGTATTAGCAGCGGTCGTGCCACCCCTCGCGCAGTATATTACGCTTCTTAAGGCGGTTAATTAGCGTCTAC

AAAGCTTACATTATATCTTAACCTCGGATCAGGTAGGGATACCCGCTGAACTTAA

>OTU_949

GAAATGCGATAAGTAATGTGAATTGCAGAATTCAGTGAATCATCGAATCTTTGAACGCACATTGCGCCCCTTGGTATTCC

GAGGGGCATGCCTGTTCGGAGCGTCATTACCACCACTCCAAGCTATGCCTTGGCTATTGGGCGTCGGTCCTTAGTTGGGC

GGCGCCTTAAAGACCTCCGGCGAGGCCACCTCCGGCCTTTAGGCGTAAGTAGAATTTATTCCGAACGTCTGTCAAAGGAG

AGGAACTCTGCCGACTTGAAACCTTTATTtttCTAGGTTGACCTCGGATCAGGTAGGGATACCCGCTGAACTTAA

>OTU_950

GAAATGCGATAAGTAATATGAATTGCAGATATTCGTGAATCATCGAATCTTTGAACGCACATTGCGCCCTGTGGTATTCC

GCAGGGCATGCCTGTTTGAGCGTCATTTCtctctcAAACCCTTGGGTTTGGTATTGAGTGATACTCTTAGTCAGACTAAG

CGTTTGCTTGAAATGTATCGGCGTGGTGGTGGAGCAGCTACCGACGAATTCAATGTATTAGGTTCTACCAAGTCGTTGTA

TCTTCCGAGGAAAACTTCCCTGCCAGGACCCCGCCTAACAAATCACAAACAAGTTTGACCTCAAATCAGGTAGGATTACC

CGCTGAACTTAA

>OTU_951

GAAATGCGATAAGTAATGTGAATTGCAGAATTCAGTGAATCATCGAATCTTTGAACGCACATTGCGCCTGGCAGTATTCT

GCCAGGCATGCCTGTCCGAGCGTCATTTCACCACTCAAGCTCTGCTTGGTGTTGGAGGACCCGCGTTACCGCGGGCCGCC

GAAATGCATCGGCTGTTGTATTTGCAGCTTCCCTGTGTAGTAATGCTTAGCTTACACTTTGAAACTCTTATATGACATGC

CGGTAAACCCTCAATTtttGAAAGGTTGACCTCGGATCAGGTAGGAATACCCGCTGAACTTAA

>OTU_952

GAAATGCGATAAGTAATGTGAATTGCAGAATTCAGTGAATCATCGAATCTTTGAACGCACATTGCGCCCCTCGGTATTCC

GGggggCATGCCTGTTCGAGCGTCATTACACCACTCAAGCCTCGCTTGGTCTTGGGCGTCGCGGTTCCGCCGcgcgcCCC

AATGTCTCCGGCTGAGCCGTCCGTCTCTAAGCGTTGTGATAAACTGTTCGCTTGCGGGATCGGGCTGGCTTGATCGCCGT

TAAACCccccATTttttCAGGTTGACCTCGGATCAGGTAGGGATACCCGCTGAACTTAA

>OTU_953

GAAATGCGATAAGTAATGTGAATTGCAGAATTTAGTGAATCATCGAATCTTTGAACGCACATTGCGCCCCTTGGTATTCC

GAGGGGCATGCCTGTTCGAGCGTCGTCACACCCCTCAAGCCGTGCCCTGtgtgtgGTTTGGTGTTGGGGTTCGCCAGTCT

GGCGGCCCTTAAAGACAGTGGCGGTGCCGGTGAGGTCTCTACGCGTAGTACAATTCTCGCGACAGAACTCCAGCGGCTGC

TAGCCAATCAATCGTTCATTTCAAGGTTGACCTCGAATCAGGTAGGGATACCCGCTGAACTTAA

>OTU_954

GAAATGCGATAAGTAATGTGAATTGCAGAATTCAGTGAATCATCGAACCTTTGAACGCACATTGCGCCCCTTGGTATTCC

GAGGGGCATGCCTGTTCGAGCGTCATTGTCAAAATCTCAAGCCTGGCTTGGTGTTGGGCTCTCGCTCCTCTCCCACAAGT

GTAGGgggTTATGGggggCGTGCCTGAAAATCATCGGCGGTGCCTCGATGGCCTCAAGCGTAGTGGACTACTCGCGCTTT

GGAGCGTCCGTCGTAGGCTCACTAGCCTGGATGTGTCATTAtataAAAGAAGCTTGACCTCGGATCAGGTAGGGATACCC

GCTGAACTTAA

>OTU_955

GAAATGCGATAAGTAATGTGAATTGCAGAATTCAGTGAATCATCGAATCTTTGAACGCACATTGCGCCCCTTGGTATTCC

GAGGGGCATGCCTGTTCGAGCGTCATTACAACCCTCAAGCACTGCTTGGTATTGGGCTCCGCTGGCTCCAGCGGGCCTTA

AAATCAGTGGCGGCGCCGTTTGGGCTCTGAGCGTAGTAAATATCCTCGCTATAGAGACCAGGCGGTTCTTGTCATCAACC

cccAACTTTCTAAGTTTGACCTCGGATCAGGTAGGGATACCCGCTGAACTTAA

>OTU_956

GAAATGCGAAACGTAATGCGAATTGCAGCATCTGTGAGTCATCGAGTCTTTGAACGCACCTGGCGCGGAGCGGCCAATGC

TCCGCACACCTGCCTGAGTGGgggAATGCACTAGTGGCTGTTAGCGGgggAGAGTGAATAGTGAATAACTATCCCATCCA

CGCTATCGGCTGGAAGGAATGtgtgGGCGCCGCTGGCCGTGTGACTGCAGGACGTAGTTCTGGAGCATGGGCGGCGGGGA

GAGTCCACAGAGTGGGGTgtgtATTGACTAGACTATACTAGGCTAGACGCTATACACCCATCCTATACCATCCCCTCAGG

TCAGGCGAGATTACCCGCTGAACTTAA

>OTU_957

GAAATGCGATAAGTAATGTGAATTGCAGAATTCAGTGAATCATCGAATCTTTGAACGCACATTGCGCCCATTAGTATTCT

AGTGGGCATGCCTGTTCGAGCGTCATTTCAACCCTCAAGCTctctTGCTTGGTGTTGGGGCTTCTGCGGCTTCGGCCGCA

GGCCCTGAAaaaCAGTGGCGGGCTCGCTATAACTCCGAGCGTAGTAATCtctctcGCTTTGGAAGTGTGGCGGTTCCCGG

CCGTTAAACCccccAATTTCTGAATGTTGACCTCGGATCAGGTAGGAATACCCGCTGAACTTAA

>OTU_958

GAAATGCGATACGTAATGCGAATTGCAGAACTCCGTGAGTCATCGAATCTTTGAACGCACATTGCGCCcccTGGTACTCC

GGggggCATGCCTGTTCGAGCGTCATTTCAACCCTCAAGCTCTGCTTGGTATTGGGCTATGTCGGTGCGCCGACGAGCTT

CAAAATTAGTGGCGGTGCCGTCCTGGCTCCACGCGTAGTAGTATTATCGcgcgTCCGGATGCCCAGGTGGTGCTAGCCAG

CAACCccccTCTTTTACAAAGGTTGACCTCGGATCAGGTAGGGATACCCGCTGAACTTAA

>OTU_959

GAAATGCGATAAGTAATGTGAATTGCAGAATTCAGTGAATCATCGAATCTTTGAACGCACATTGCGCCCCTTGGTATTCC

GGggggCATGCCTGTTCGAGCGTCATTATAACCCTCAAGCCTAGCTTGGTGTTGGAGCATGCTACCTAAGCAGCTCTTAA

AATCAGTGGCAGTGCCCTTCGGCTCTAAGCGTAGTAACTTCtctcGCTATGGAACCCGGAGGGTACGCGCCAGAACCccc

AACTTTCTATGGTTGACCTCGGATCAGGTAGGGATACCCGCTGAACTTAA

>OTU_960

GAAATGCGATACGTAGTGTGAATTGCAGAATTCAGTGAATCATCGAATCTTTGAACGCACATTGCGCCCTTTGGTATTCC

AAAGGGCATGCCTGTTCGAGCGTCATTTGTACCTTCAAGCTTTGCTTGGTGTTGGGCGTCTTtttGTCTTTGGCCTCGCC

CAAAGACTCGCCTTAAAACGATTGGCAGCCGGCCTACTGGTTTCGCAGCGCAGCACATTtttGCGCTTGCAATCAGCAAa

aaGGACGGCAATCCATCAAGACTACATTtttACGTTTGACCTCGGATCAGGTAGGGATACCCGCTGAACTTAA

>OTU_961

GAAATGCGATAAGTAATGTGAATTGCAGAATTCAGTGAATCATCGAATCTTTGAACGCACATTGCGCCCCGCCAGCATTG

CTGGCGGGCATGCCTGTTCGAGCGTCATTTCAACCCTCAAGCTCCGCTTGGTGTTGGGGCCCTACAGCTGATGTAGGCCC

TCAAAGGTAGTGGCGGACCCTCCTGGAGCCTCCTTTGCGTAGTAACTTTTACGTCTCGCACTGGGATCCGGAGGGACTCT

TGCCGTAAAACCccccAAATTTTCCAAAGGTTTGACCTCGGATCAGGTAGGAATGACCCGCCTGAACTTAA

>OTU_962

GAAATGCGATACGTAGTGTGAATTGCAGAATTCAGTGAATCATCGAATCTTTGAACGCACATTGCGCCCTTTGGTATTCC

AAAGGGCATGCCTGTTCGAGCGTCATTTGTACCCTCAAGCTTTGCTTGGTGTTGGGCGTTGTTGTCTTTGGTTTGCCCAA

AGACTCGCCTTAAAACGATTGGCAGCCGGCCTACTGGTTTCGCAGCGCAGCACATTtttGCGCTTGCAATCACAAGAGGA

CGGCACTCCATCAAGACTCCTTCTCACGTTTGACCTCGGATCAGGTAGGGATACCCGCTGAACTTAA

>OTU_963

GAAATGCGATAAGTAATGTGAATTGCAGAATTCAGTGAATCATCGAATCTTTGAACGCACATTGCGCCCCTTGGTATTCC

GGgggCATGCCTGTTCGAGCGTCATTTCAACCCTCAAGCTTAGCTTGGTATTGAGTCTATGTCAGTGAATGGCAGGCTAC

TAAATCAGTGGCgcgcCGCTGGGTCCTGAACGTAGTAATATCtctcGTTACAGGTTCTCGGTGTGCTTCTGCCAAAACCC

AATTtttCTTATGGTTGACCTCGGATCAGGTAGGGATACCCGCTGAACTTAA

>OTU_964

GAAATGCGATAAGTAATGTGAATTGCAGAATTCAGTGAATCATCGAATCTTTGAACGCACATTGCGCCcccTGGTATTCC

GGgggCATGCCTGTTCGAGCGTCATTTCACCACTCAAGCCTCGCTTGGTATTGGGCAACGCGGTCCGCCGCGTGCCTCAA

ATCGACCGGCTGGGTCTTCTGTCCCCTAAGCGTTGTGGAAACTATTCGCTAAAGGGTGTTCGGGAGGCTACGCCGTAAAC

AACCCATTCCTTAGTTAGTACCTCGGATCAGGTAGGGATACCCGCTGAACTTAA

>OTU_965

GAAATGCGATAAGTAGTGTGAATTGCAGAATTTAGTGAATCATCGAATCTTTGAACGCACATTGCGCCCTTCGGTATTCC

GTTGGGCATGCCTGTTCGAGCGTCATTAAaaaCCTTCAAGCTAGGCTTGGTGTTGGGTGTTTGTTCTGCCTCTGTGCATG

GACTCGCCTCAAATTCATTGGCAGCTAGTAGATTTGGCTTCGTGCGCAGCACATTGCGCAGCGGCTCCAGCCTACCATCT

TCCAGTAAGCTATTCTTTTATTTGACCTCGGATCAGGTAGGGATACCCGCTGAACTTAA

>OTU_966

GAAATGCGATAAGTAATGTGAATTGCAGAATTTAGTGAATCATCGAATCTTTGAACGCACATTGCGCCccccGGCATTCC

GAGGGGCATGCCTGTTCGAGCGTCAGAACACCCCTCAAGCCTGGCTTGGTCTTGGGGCTCGCCGGCTCGGCGGCCCCTAA

ACGCAGTGGCGGTGCTGTTGTGCTCTCCGCGTAGTCATGTATCTCGCGACAGAGCGGCGACGGCACCCGCCAGAACCccc

cAGTCTTTGGATGACCTCGGATCAGGTAGGGATACCCGCTGAACTTAA

>OTU_967

GAATTGCGATATGTAATGTGAATTGCAGAATTCAGTGAATCATCGAATCTTTGAACGCACATTGCACCCTCTGGTATTCC

GGAGGGTATGCCTGTTTGAGTGTCATTAATCtctcAACACAGACAAGTTTTACTACTTGTTCTGTTGCTTGGATGTTGAA

GGCTTGCTGGACTGTCAGCTCCTTTTAAATGTATTAGCAGAAaaaTGCAGAACTGGCTTCAGTTTGATAAATGACTTATC

TGTGTTGCTGTTGCTAGGATAATGCCAATCTTTTCAGCTTCTAATAGTCtctcCATGAGACTGTCTCATTTGAGGCATAT

AAAACTTGACACTTGACCTCAAATCAGGTAGGACTACCCGCTGAACTTAA

>OTU_968

GAAATGCGATAAGTAATGCGAATTGCAGAATTCAGTGAGTCATCGAATCTTTGAACGCACATTGCGCCCTGTGGTATTCC

GCAGTGCATGCCTGTTCGAGCGTCATTTCAACCCTCAAGCTCTGCTTGGTGTTGGGCCCTGCCCGCTGCGGCCGGCCCCA

AAGACAGTGGCGGCGCCGCCTGGCCCTGAGCGTAGTACGTCtctcGCTCCAGCGCCCGGCGGTGGCCTGCCAGAACCccc

GACTctctTCGTTGACCTCGGATCAGGTAGGGATACCCGCTGAACTTAA

>OTU_969

GAAACGCGATAGGTAATGTGAATTGCAGAATTCAGTGAATCATCGAATCTTTGAACGCATCTTGCGCTTGCTGGTATTCC

GGCAAGCATGCCTGTTTGAGTGTCATGAAAATTATCAACCATGACTTGGGTTTAGGCCCTCGTTCTTGGCTTGGATTTGG

GTGCTTGCGGTCTTGTGCTGCTCACCTTAAAAGGATTAGCTGGACATGTCTTTTGACTTGGTTCTACTTGGCGTAATAAG

TATTTCGCTGAGGACATCCCTTCTGGGGTGGCCAAGACTCTGGCAAACGTCTGCTTTCTAACTCGACTGTCCTCGGACAT

CACTTtttATGATCTGGCCTCAAATCAGGTAGGACTACCCGCTGAACTTAA

>OTU_970

GAAATGCGATAAGTAATGTGAATTGCAGAATTCAGTGAATCATCGAATCTTTGAACGCACATTGCGCCCGCCAGTATTCT

GGCGGGCATGCCTGTTCGAGCGTCATTTCAACCCTCAGGCCcccAGTGCCTGGCGTTGGGGATCGGCGCGGGCGGCGCAC

CCTCCGGGGTGTGCCCGCCTGCCGGCCCCGAAATTCAGTGGCGGTCTCGCTGTAGTCCcccTCTGCGTAGTAGCACAACT

CGCATTGGAGCTCGGCGGTGGCCATGCCGTAAAACACCCCACTTCTGAAAGTTGACCTCGGATCAGGTAGGAATACCCGC

TGAACTTAA

>OTU_971

GAAATGCGATACTTGGTGTGAATTGCAGAATCCCGTGAACCATCGAGTCTTTGAACGCAAGTTGCGCCCGAAGCCATTAG

GCCGAGGGCACGCCTGCCTGGGTGTCAcacaTCGTTGCCCCAATGCCAGTGCCTCTTGCTAGGTCCTGAGCGGGGCGAAT

GTTGGCTTCCCGTGAGCCTTGTCTCGCGGTTGGTTAAaaaaTGTGTCTGTGGTGGAGAGCACCACGATGGATGGTGGCTG

AGTAAAATCTCGAGACCAATCGCGTgtgtCTCTTTGCCGGTTTTGGACTATGTGACCCACGGAGCATCATATACGATCGC

CCCATAACGAGACCTCAGGTCAGGCGGGGCTACCCGCTGAGTTTAA

>OTU_972

GAATTGCGATAAGTATTGTGAATTGCAGATTTTCGTGAATCATCGAATCTTTGAACGCATATTGCACTCTATAGTATTCT

GTAGAGTATGCTTGTTTGAGCGTCATTTCATTCTTAAACCCCTAGGTTTAGTATTGGAGGATGTAATTCTTTATTGAATA

ACTCTTCTGAAATGACTTGGCATCTTTACCTTGTGTAAATCTAAGCTTGTATGAACTTATTATTAGGTCTAACCAACTAA

TGAGTACTCATCTGGCGTCTTACGCAAAGATTTATTAAAGTGCCTAACAAAATTTATCATTAATTTTGACCTCAAATCAA

GTAAGGATACCCACTGAACTTAA

>OTU_973

GAAATGCGATAAGTAATGTGAATTGCAGAATTCAGTGAATCATCGAATCTTTGAACGCACATTGCGCCCGCTAGTATTCT

GGCGGGCATGCCTGTTCGAGCGTCATTTCAACCCTTAAGCCCTGTTGCTTAGTGTTGGGAGCCTACTGCCCTGTAGCTCC

CCAAAGTTATTGGCGGAGTCGGTCAcacacTCTGGATGTAGTAATTtttttCTCGTCTATAGTTGGGCCGGTCCCCTGCC

GTAAAACCccccATTTTAAAGGTTGACCTCGGATCAGGTAGGAATACCCGCTGAACTTAA

>OTU_974

GAAATGCGATAAGTAATGTGAATTGCAGAATTCAGTGAATCATCGAATCTTTGAACGCACATTGCGCCCCTTGGTATTCC

GAGGGGCATGCCTGTTCGAGCGTCATTACACCACTCAAGCTATGCTTGGTATTGGGCGTCGTCCTTAGTTGGGCGCTACT

AAACGCCGTCGGCGGACGCACTCCGGCTTTAGGCGTAGTAGAATTTATTCGAACGTCTGTCAAAGGAGAGGAACTCTGCC

GACTGAAACCTTTATTtttCTAGGTTGACCTCGGATCAGGTAGGGATACCCGCTGAACTTAA

>OTU_975

GAATTGCGATAAGTAATGTGAATTGCAGAATTCAGTGAATCATCGAATCTTTGAACGCACCTTGCGCCTTTGGGTATTCT

CAAAGGCATGCCTGTTTGAGTGTCATAACAATCTCATCTCCCAATCTTtttGAAAGAAAAGGTTGCTTGGAGGTGGTGAT

GGGCGCTTGCCATGCTGAATAAGTCTGGCTCGCCTTAAAGACATCAGTAGCTCTTATCGAGTCCGGTCTGACTATGtgtg

ATAATTTGATCGCATAGGATGTGCTTGCACAACCGGTCTTATTTTAAGAGTCTGCTTCTAACCTGGAATGGTATGAGAGT

AACTTTCATCCATCGCAGCTTTATTTGTCTGACCTCAAATCAGGTAGGACTACCCGCTGAACTTAA

>OTU_976

GAAATGCGAAAACTAATGTGAATTGCAGAATTCCGTGAATCATCGAGTCTTTGAACGCACATTGCGCCCTTTGGTATTCC

GAAGGGCATGCCTGTTCGAGCGTCATTGCACCCCTCAAGCATTGCTTGGTATTGGGCTTCGCCCCTGTGGCGGGCCTGGA

ATTCAGTGGCGGTCCAGCGAGACTTCAAGCGTAGCAAGATTTCACGATCCCGCTTTGGAAGGTTCCTGCTGCGTGCCGGC

CAGATAACCACAATTTCTTTCAATGATTGACCTCGGATCAGGTAGGGATACCCGCTGAACTTAA

>OTU_977

GAAATGCGATAAGTAGTATAAATTACAGAATTTAGTAAATCATCGAATCTTTAAACGCACATTACGCCCCTTAGTATTCC

GTAGGGCATGCCTATTCAAGCGTCATTTACCCCTTAAGCTCTGCTTAGTATTAGGCGTTATTCCGCTTCGCGCAAGGACT

CGCCCTAAAGGTATTAGCAGCAGTCGTGCCACCCCTCGCGCAGTATATTACGCTTCTTAAGGCGGTTGATTAGCGTTCAC

AAAGCTTATATTATATCTTAACCTTAGATTAGGTAGGGATACCCGCTAAACTTAA

>OTU_978

GAAATGCGATAAGTAGTGTGAATTGCAGAATTCAGTGAATCATCGAATCTTTGAACGCACATTGCGCCGCATAGTATTCT

GTGCGGCATGCCTGTTCGAGCGTCATTTGTACCTTCAAGCACTGCTTGGTGTTGGGTGATTGTCTATGTAACTTGCTAGA

CACGCCTTAAATGAATTGGCAGCTGGTATTCTGGTTGAAGCGTAGCACAATTACGTCTTTCTCTAAGTGAGCCGCATCCA

TAAGTATTtttCACGTTTGACCTCGGATCAGGTAGGGATACCCGCTGAACTTAA

>OTU_979

GAATTGCGATAAGTAATGTGAATTGCAGATACTCGTGAATCATTGAATTtttGAACGCACATTGCGCCCTTGAGCATTCT

CAGGGGCATGCCTGTTTGAGCGTCATTTCCTTCTCAAAAGATAATTTATTATTttttGGTTGTGGGCGATACTCAGGGTT

AGCTTGAAATTGGAGACTGTTTCAGTCTTttttAATTCTAACACTTAGCTTCTTTGGAGACGCTGTTCTCGCTGTGATGT

ATTTATGGATTTATTTCGTTTTACTTTACTAAAGGGAAATGGGTAACGTACCTTAGCAAAGGGTTGGCTTTTAATTATTC

ATCAAGTTTGACCTCAAATCAGGTAGGATTACCCGCTGAACTTA

>OTU_980

AACCGGTTGATCGCGGTATCTGGGGAGACGGTCTGGAACAACGTCATAGTTGCCGCTCCGAAGCCGACTCCCATCAACAG

CGCAATGCTACTATTGATGGCCTCAGCGAAGTCTCCGCCATAAACAGGACCCAACCCGACGAGGATCGGGACAGTCAGCG

TGATGCCAAGCGCCATGAAGGCGGTCGACGGCCGAGCCTGCAACGATCCTCCGAATAGAAACGCCGGTGCGAGGACCGCG

ATCAACACCCAGAACTCGAAGACACGCGGCAGGACGGTAAAGCTGTAGATCAGGCTGATCGCGATACCATAGACTGAGCC

AA

>OTU_981

GAAATGCGATAAGTAATGTGAATTGCAGAATTCAGTGAATCATCGAATCTTTGAACGCACATTGCGCCCCTTGGTATTCC

GAGGGGCATGCCTGTTCGAGCGTCATTACACCACTCAAGCTATGCTTGGTATTGGGCGTCGTCCTTAGTTGGGCgcgcCT

TAAAGACCTCGGCGAGGCCACTCCGGCTTTAGGCGTAGTAGAATTTATTCGAACGTCTGTCAAAGGAGAGGAACTCTGCC

GACTACGTAATTCCTTTATTTTCGTAGGTGACCTCGGATCAGGTAGGGATACCCGCTGAACTTAA

>OTU_982

CAGCTGCGACACTTGGTGTGAATTGCAGGACACATTGAGCACTGATTtttCGAACGCACATTGCGGCCTCGGGTCCTACC

CGGGGCCACGTCTGTCTGAGGGTCGGATGAATGCATGCCTAGGAATCTCTTCCCACGTGGTCGCACCGGAAGTTCCGTCT

TCCGCCGACCTAAGGCTTGGAGGTTCCCACCGGTCGAgagagCGTCCCGCGTAGGATGCCCGACGTACGAGTGGCGGGAT

GCTAAACGCTCTGCATGTCGGGACTGCGGTCGTATAAGACGCCAGAACCGGAAGTCTCGTGATCGACCGTTTCATGGTCT

TATTATTGTCGACCTCAGATCAGGCGAGACGACCCGCTGAATTTAA

>OTU_983

GAAATGCGATAAGTAATGTGAATTGCAGAATTCAGTGAATCATCGAATCTTTGAACGCACATTGCGCCCTCTGGTATTCC

GGAGGGCATGCCTGTTCGAGCGTCATTTCAACCCTCAAGCCTGGCTTGGTGTTGGGGCACTGCCTCCTCGCGGgggCAGG

CCCTGAAATACAGTGGCGAGCTCGCCAGGACTCCGAGCGCAGTAGTTAAACCCTCGCTCTGGAAGGCCTGGCGTGCCCTG

CCGTTAAACCCCAACTCCTGAAAATTTGACCTCGGATCAGGTAGGAATACCCGCTGAACTTAA

>OTU_984

GAATTGCGATAAGTAATGTGAATTGCAGAATACTCGATGAATCAATTGTAATTTTGAACGCACATTGCGCCCCTTGGTAT

TCCGAGGGGCATGCCTGTTCGAGCGTCATTACACCACTCAAGCTATGCTTGGTATTGGGCGTCGTCCTTAGTTGGGCgcg

cCTTAAAGACCTCGGCGAGGCCACTCCGGCTTTAGGCGTAGTAGAATTTATTCGAACGTCTGTCAAAGGAGAGGAACTCT

GCCGACTGAAACCTTTATTtttCTAGGTTGACCTCGGATCAGGTAGGGATACCCGCTGAACTTAA

>OTU_985

GAAATGCGATAAGTAATGTGAATTGCAGAATTCAGTGAATCATCGAATCTTTGAACGCACATTGCGCCCTGTGGTATTCC

GCAGGGCATGCCTGTTCGAGCGTCATTTCAACCAATCAAGCCTAGGCTTGGTCTTGGGGCATGCGGGTTTTCGCAGCCTC

TAAAATCAGTGGCGGTGCTAGTTAGCTCTGAGCGTAGTAATACTCCTCGCTATAGAGTCTAGGTAGTGTCTTGCCAACAA

CCCCTAATTttttCAGGTGACCTCGGATCAGGTAGGGATACCCGCTGAACTTAA

>OTU_986

GAAATGCGATAAGTAATGCGAATTGCAGAATTTCCGTGAGTCATCGAATCTTTGAACGCACATTGCGCCCACTGGTATTC

CGGTGGGCATGCCTGTTCGAGCGTCATTATCCTCCCTCAAACCTCTGTGTTTGGTGTTGGGCCGCGTTGGCGTCACAGCC

AACTGGTCTCAAAGACAATGACGGCGTCCGTGGGACCCTCTTCGCAACGAGCATCTTCGAAGCACGCGTCGAGTCTAAAG

GACCTTCCGGGCCGGTCTACCCTTTATCTTTCTAAGGTTGACCTCGGATCAGGTAGGAATACCCGCTGAACTTAA

>OTU_987

GAAATGCGATAAGTAATGTGAATTGCAGAATTCCGTGAATCATCGAATCTTTGAACGCACATTGCGCCCCTCGGTATTCC

GGggggCATGCCTGTTCGAGCGTCATTACACCACTCAACCTCGGCTTGGTCTTGGGCGTCGCGGTCCCCGCGTGCCTCAA

TGTCGCCGGCTGGTGCGTCCGTCTCTAAGCGTTGTGATTTACAGCTCGCTCTGGAGGCCGGACGGGCCCCGCCGTCAAGC

CCTTCTTTACAGGTTGACCTCGGATCAGGTAGGGATACCCGCTGAACTTAA

>OTU_988

GAAATGCGATAAGTAATGTGAATTGCAGAATTCAGTGAATCATCGAATCTTTGAACGCACATTGCGCCCCTTGGTATTCC

GAGGGGCATGCCTGTTCGAGCGTCATTACAACCCTCAAGCTCTGCTTGGCAATTAGGCTTCCGCGGTTTACGCCGCGGGC

CCTAAAATCAGTGGCGGCGTCTTCGGGCTCTGAACGTAGTAAACATTCCTCGTTACAGAGTCCTGGCGGCGGTAGCCATC

AACCccccccAACTTTCTAAGTTTGACCTCGGATCAGGTAGGGATACCCGCTGAACTTAA

>OTU_989

GAAATGCGATAAGTAATATGAATTGCAGATTTTCGTGAATCATCGAATCTTTGAACGCACATTGCGCCCTTTGGTATTCC

AAAGGGCATGCCTGTTTGAGCGTCATTTCtctctcAAATCTTCGGATTTGGTTTTGAGTGATACTCTTAGTCAGACTAAG

CGTTTGCTTGAAATGTATTGGCATGAGTGGTACTAGATAGTGCTGAACTGTTTTCAATGTATTAGGTTTATCCAACTCGT

TGACCAGTAAAGTATTTGTTTATTACACAGGCTCGGCCTTACAACAACAAACAAAGTTTGACCTCAAATCAGGTAGGACT

ACCCGCTGAACTTAA

>OTU_990

GAAATGCGATAAGTAATGTGAATTGCAGAATTCAGTGAATCATCGAATCTTTGAACGCACATTGCGCCCCTTGGTATTCC

GAGGGGCATGCCTGTTCGAGCGTCATTACACCACTCAAGCCTATGCTTGGTATTGGGCGTCGTCCGTTAGTTGGGCCGCG

CCCTTAAAGACCTCGGCGAGGCCACTCCGGCTTTAGGCGTAGTAGAATTTATTCGAACGTCTGTCAAAGGAGAGGAACTC

TGCCGACTGAAACCTTTTATTtttCCTAGGTTGACCTCGGATCAGGTAGGGATACCCGCTGAACTTAA

>OTU_991

GAAATGCGATAAGTAATGTGAATTGCAGAATTCAGTGAATCATCGAATCTTTGAACGCACATTGCGCCCACCAGTATTCT

GGTGGGCATGCCTGTCCGAGCGTCATTTCAACCCTCAGGCCCAGCCTGGTGTTGGGGATTGGCTTCGCGGCCACCcccGA

AATGCAGTGGCGGCCCTCCGCGAACTCCTCTGTGCAGTAGTGATACCTCGCATTGGATAGTGGTTGCGCCTCGCCGTAAA

ACCTCCAACTTCTCAAGGTTGACCTCGGATCAGGTAGGAATACCCGCTGAACTTAA

>OTU_992

GAAATGCGATAAGTAATGTGAATTGCAGAATTCAGTGAATCATCGAATCTTTGAACGCACATTGCGCCCCGTGGTATTCC

GCGGGGCATGCCTGTTCGAGCGTCATCTACACCCTCAAGCTCTGCTTGGTGTTGGGCGTCTGTCCCGCCTCTGCgcgcgG

ACTCGCCCCAAATACATTGGCAGCGGTCCTTGCCTCCTCTCGCGCAGCACAATTGCGTTTGAAAGGGAGCGTGGCCCGCA

TCCACAAAGCAACATTACCGTCTTTGACCTCGGATCAGGTAGGGATACCCGCTGAACTTAA

>OTU_993

GAAATGCGATAAGTAGTGTGAATTGCAGAATTCAGTGAATCATCGAATCTTTGAACGCACATTGCGCCCTTTGGTATTCC

TTAGGGCATGCCTGTTCGAGCGTCATTTAAACCTTCAAGCTCTGCTTGGTGTTGGGCATGTCACGCCccccTCCGCGGgg

gCGTGCGCCTCAAATatatTGGCGGCCGGTACGTTGGCTTCGAGCGCAGCAGAAACGCGAACTCGGGGACTCCGGATCGG

CCCTCCACAAGCTATTTTACCATTTTGACCTCGGATCAGGTAGGGATACCCGCTGAACTTAA

>OTU_994

GAATTGCGATAAGTAATGTGAATTGCAGATACTCGTGAATCATTGAATTtttGAACGCACATTGCGCCCTTGAGCATTCT

CAAGGGCATGCCTGTTTGAGCGTCATTTCCTTCTCAAAAGATAATTttttATTttttGGTGTGGGCGATACTTAGGGTTA

GCTTGAAATTGGAGACTGTTTCAGTCTTtttttAATTCAACACTTAGCTTCTTGGAGACGCTGTTCTCGCTGTGATGTAT

TTATGGATTTATTCGTTTTACTTTACAAGGGAAATGGTAATGACCTTAGGCAAAGGGTTGCTTTTAATATTCCTAGTCAA

GTTTTGACCTCAAAATCAGGTAGGATTACCCGCTGAACTTA

>OTU_995

GAAATGCGATAAGTAATGTGAATTGCAGAATTCAGTGAATCATCGAATCCTTGAACGCACATTGCGCCCTCTGGTATTCC

GGggggCATGCCTGTTCGAGCGTCATTATAACCACTCAAGCTCTCGCTTGGTATTGGGGTTCGCGGTTTCGCGGCCCCTA

AAATCAGTGGCGGTGCCTGTCGGCTCTACGCGTAGTAATACTCCTCGCGATTGAGTCCGGTAGGTCTACTTGCCAGCAAC

CCCTAATTtttttAAGGTTGACCTCGGATCAGGTAGGGATACCCGCTGAACTTAA

>OTU_996

GAAATGCGATAAGTAGTGTGAATTGCAGAATTCAGTGAATCATCGAATCTTTGAACGCACATTGCGCCCCTTGGTATTCC

ATGGGGCATGCCTGTTCGAGCGTCATTTGTACCTTCAAGCTATGCTTGGTGTTGGGTGTCTGTCTCTGCTTTTGTGCCTA

GACTCGCCCTAAATACATTGGCAGCTAATatatTTGTTTCAAGCGCAGCACAATTTGCCTTGGAGCCTATTGATATTCGC

TTCCATTAAGACTATTTAACACTTTTGACCTCGGATCAGGTAGGGATACCCGCTGAACTTAA

>OTU_997

ACATCCTCAACCTCATATCGCTCTGCCGTGGCAAGATTGATGACTCTTGTGCGCAGGTCACAAAACTCTCCCACGTGCTT

CCATTAAAATAGTGATCCTGAAATGCCACGCACAGAATCTCATCGCTCACATACGTGATCTCATAGTCCACATAGCATGC

CAGATACGGGTAGTCTTCATCCAGATACAAATATTCTTCCTGCGGCTCCGGATACATGGTATCCAGCTGCGCACAGGCGG

CATTCCGCAGAATCTCATTTACTCCGGTCGCATCTCTTCCATCC

>OTU_998

GAAATGCGATATGTAATGTGAATTGCAGAATTCAGTGAATCATCGAATCTTTGAACGCACCTTGCACCTTTTGGTATTCC

GAAAGGTATGCCTGTTTGAGTGTCATGAAATCTCAATCCCCTCGGGTTTTACGACCTGATCGGACTTGGACTTGGGCGTC

TGCCATCCGTGGCTCGCCTTAAATGGATTAGTGGGAATAGCACTTAGCGATTAGACGTAATAAGTTTCGTCTGGTCCGTg

tgtgACGCTCGCTCATAACAACCAATTTACTTCATGTCTCTGACCTCAAATCAGGTAGGGCTACCCGCTGAACTTAA

>OTU_999

GAAATGCGATAAGTAGTGTGAATTGCAGAATTCAGTGAATCATCGAATCTTTGAACGCACATTGCGCCCCTTGGTATTCC

ATGGGGCATGCCTGTTCGAGCGTCATTTGTACCCTCAAGCTCTGCTTGGTGTTGGGCGTTTGTCTTGTGAGACTCGCCTT

AAAATAATTGGCAGCCAGCATACTGGTATTGGAGCGCAGCACAAGTCGCGCTTTGTATCTGAATGCGGGCGTCCAGAAGC

CTCTTttttCAACTTTTGACCTCGGATCAGGTAGGGATACCCGCTGAACTTAA

>OTU_1000

GAAATGCGATAAGTAATGCGAATTGCAGAATTCAGTGAGTCATCGAATCTTTGAACGCATATTGCGCCCTTTGGTATTCC

GAAGGGCATGCCTGTTCGAGCGTCATTATCAACCATCAAGCTTATGGGCTTGTCGTTGGACCTTTATAATTGAAATatat

TATAGGTCCGAAAGATAATGACGGTGTCATGTTTTGACCCTAAATGCAACGAGCTTTAATAGCACGCATTTGAAGTGGTC

GGACTGTACCCGGTCTTAACTAATTATTTTCTTAAGGTTGACCTCGGATCAGGTAGGAATACCCGCTGAACTTAA

>OTU_1001

GAAATGCGATAAGTAATGTGAATTGCAGAATTCAGTGAATCATCGAATCTTTGAACGCACATTGCGCCCATTAGTATTCT

AGTGGGCATGCCTGTTCGAGCGTCATTTCAACCCCTAAGCACAGCTTATTGTTGGGCGTCTACGTCTGTAGTGCCTCAAA

GACATTGGCGGAGCGGCAGCAGTCCTCTGAGCGTAGTAATTCTTTATCTCGCTTCTGTTAGGCGCTGCCcccccGGCCGT

AAAACCcccAATTttttCTGGTTGACCTCGGATCAGGTAGGAATACCCCGCTGAACTTAA

>OTU_1002

GAAATGCGATAAGTAGTGTGAATTGCAGAATTCAGTGAATCATCGAATCTTTGAACGCACATTGCGCCCCTTGGTATTCC

ATGGGGCATGCCTGTTCGAGCGTCATTTGTACCTTCAAGCTTTGCTTGGTGTTGGGTGCTTGTCTTtttGTTAAGACTCA

CCTCAAAGTCATTGGCAGCCAGTGTTTTGGTAGTAAGCGCAGCACATTTTGCGTCTTGGTCCCTCAACAGCGGCATCCAT

CAAGCCATTTTCTCACTTTTGACCTCGGATCAGGTAGGGATACCCGCTGAACTTAA

>OTU_1003

GAAATGCGATAAGTAATGTGAATTGCAGAATTCAGTGAATCATCGAATCTTTGAACGCACCTTGCGCTCCTTGGTATTCC

GAGGAGCATGCCTGTTTGAGTGTCTTGAAATCATCAAACCCCTCCGGTTTCTGCTGGAACGGGTTTGGACTTGGgggTTt

ttGCTGCCTTCTTGGTGGCTCCCCTTAAATGAATCGGTCGGGCTTTCGCGTGGCTTGTCCCTTGGCGTTGTAACAACCTC

GTCGCTGGACGGCTCGCGAGTTTTGCGCCCCTCCAAGACCAGCCTCTGGAGTGGCGGTCAACTTCAATTtttGATCTGAC

CTCAAATCAGGTAGGACTACCCGCTGAACTTAA

>OTU_1004

GAAATGTGATAAGTAATGTGAATTGCAGAATTCAGTGAATCATCGAATCTTTGAACGCATCTTGCACTctctGGTATTCC

GGAGAGTATGTCTGTTTGAGTGTCATGAATTCTTCAACCcccTCTTTTCTTAGTGAATTGAGAGGAGCTTGGATTCTGAG

TGTTGCCTCTAACCCGGGCTCATTCGTAATGTATTAGCATCCATATTCGAATTTCGGATTGACTTGGCGTAATAGACTAT

TCGCTGAGGAATCTAACTTCGGTTAGAGCCGTGTTTGAACTAGGAAGCTTCTAATCTAGCTTAGTCTACCTTTAGATTAG

ATCTCAAATCAGGCAGGATTACCCGCTGAACTTAA

>OTU_1005

GAAATGCGATACGTAATGTGAATTGCAGAATTCAGTGAATCATCGAATCTTTGAACGCACCTTGCACTCTTTGGTATTCC

GAAGAGTATGTCTGTTTGAGTGTCATGAAACTCTCAACCccccTGTTTTGTAATGAGATAGGCGCGGGCTTGGATTGTGG

CTGTCTGCTGGCTTATAATGAGCCTGCTCAGCTGAAATATACGAGCAACCCAGTTTGAAATACAGACGGTTTGACTTGGC

GTAATAATCTATTTCGCTGAGGACGTCGCTTtttCAATAATTCAGTGGTGCTTCTAATGCAATTACTATTAAGCTTTAGA

CCTCAAATCAGTCAGGACTACCCGCTGAACTTAA

>OTU_1006

GGAATGCGATAAGTAATGTGAATTGCAGAATTCAGTGAATCATCGAATCTTTGAACGCACATTGCGCCCTTTGGTATTCC

GAAGGGCATGCCTGTCTGAGCGTCAATAAATCAATCAAACCTTGGTTTGGTATTGGGAGAAGTGGCATTGCCTctctcCT

TTAAGCTATTGGCGACGCTATTTCAGCTTGTAAGACGTAGTAAGTAATTTCTCGTTAAAGCAACTGTATAGATGTCTGCC

AACTGAACGATTTATTTTATAGCTTGACCTCAGATCAGGTAGGGATACCcccTGAACTTAA

>OTU_1007

GAAATGCGATAAGTAGTGTGAATTGCAGAATTCAGTGAATCATCGAATCTTTGAACGCACATTGCGCCCCTCGGTATTCC

GTGGGGCACGCCTGTTCGAGCGTCATTTACACCCTCAAGCGCAGCTTGGTGTTGGGCGTCTGTCCCGCTCTCCGTAGCGC

GGACTCGCCTCAAAGTCATTGGCGGCGGTCGTGCCGGCTCCTCGCGCAGCACATTTGCGCTTCTCGGAGGCCCGGCGGAT

CAGCATCCAGCAAGCTACTTtttATGACTTGACCTCGGATCAGGTGAGGATACCCGCTGAACTTAA

>OTU_1008

GAAATGCGATAAGTAGTGTGAATTGCAGAATTCAGTGAATCATCGAATCTTTGAACGCACATTGCGCCCCTTGGTATTCC

ATGGGGCATGCCTGTTCGAGCGTCATTTGTACCCTCAAGCTTTGCTTGGTGTTGGGCGTGTTGTCTTtttATTCAGACTC

GCCTTAAAGTAATTGGCAGCCAGTGTTTTGGTAGTAAGCGCAGCACATTTTGCGTCTTAGTCCCCTAACAGCGGCATCCA

TAAAGCCTCTTTCTCACTTTTGACCTCGGATCAGGTAGGGATACCCGCTGAACTTAA

>OTU_1009

GAAATGCGATAAGTAGTGTGAATTGCAGAATTCAGTGAATCATCGAATCTTTGAACGCACATTGCGCCCCTTGGTATTCC

ATGGGGCATGCCTGTTCGAGCGTCATTTGTACCCTCAAGCTTTGCTTGGTGTTGGGTGTTTGTCTCGTGTTACGCGTAGA

CTCGCCTTAAAGCGATTGGCAGCCGGCATATTGGCCTTGGAGCGCAGCACATTTTGCGCTTCTAGTCATGAATGTTGGCG

TCCATTAAGCCTATACATTTGCTCTTGACCTCGGATCAGGTAGGGATACCCGCTGAACTTAA

>OTU_1010

GAAATGCGATAAGTAATGTGAATTGCAGAATTCAGTGAATCATCGAATCTTTGAACGCAGATTGCGCCCTTTGGTATTCC

GAAGGGCATGCCTGTTTGAGTGTCTTATAAACCTCACCTCTATTGTTTTACGACAATTAGATTGGTGCTTTGGGTGTCTG

CCGTTTACTCTGGCTCGCCTTAAATGTCTAAGTGGAATGCGTAATAAGTCTTCGCAACCCTCCACTCTAATACAACTTTT

ATATTCTGACCTCAAATCAGGTAGGACTACCCGCTGAACTTAA

>OTU_1011

GAAATGCGATACGTAATGTGAATTGCAGAATTCAGTGAATCATCGAATCTTTGAACGCACATTGCGCTctctGGTATTCC

GGAGAGCATGCTTGTTTGAGTATCAGTAAAACACCTCAAGTCCGCTCTTCTTtttGAAGACGGATTTGGACTTGAGCGAT

CCCAACAGGAGGGGCTTTCCCCTCTGGCGGGTTGCTTGAAATGCAGGTGCAGCTGGATTTTCATCTGGGCTATAAGCATA

TTTATTTAGTCCCGTTAAACGGATTATTACTTtttGCTGCAGCCAACGTAAAGGTTGATTGTCCTCTTGCTGACTGATGC

AGGAATTTTCGGgggATCGGCAACGATTACCCGTTAAACTCGATCTCAAATCAAGTAAGACTACCCGCTGAACTTAA

>OTU_1012

GAAATGCGATAAGTAATGTGAATTGCAGAATTCCGTGAATCATCGAATCTTTGAACGCATATTGCGCCCTCTGGTATTCC

GGggggCATGCCTGTCCGAGCGTCATTACAACCTTCAAGCACGGCTTGGCTGTTGAGCGGTCTGTTTTGACGAACAGGGC

TCGAAATGCAGTGGCGACGGCGAGATTGGTGCCCGAGCGTATGGGGCTCTGTCACACGCTCTGGTGGCTCCGCTTACAGT

CTGCCATAGACCAAATCAATCTGATTGACCTCGGATCAGGTAGGAACACCCGCTGAACTTAA

>OTU_1013

GAAATGCGATAAGTAGTGTGAATTGCAGAATTCAGTGAATCATCGAATCTTTGAACGCACATTGCGCCTTCTGGTATTCC

GGGAGGCATGCCTGTTCGAGCGTCATCAAaaaCCTCAGTCAACAATTTATTGTCGATTGGTCTTGCATTGGAGGTGAAAG

CTTCCTGTGCGAAATTCAATGGCGATGAGCCACGCAGCCAAAGCGTAGTAAATTACTCTCGTAATGGTGAAGTGGAAGCA

TCCGCCGAAACCccccccATATCTAAGTTtttGACCTCGGATCAGGTAGGGATACCCGCTGAACTTAA

>OTU_1014

GAAATGCGATAAGTAGTGTGAATTGCAGAATTCAGTGAATCATCGAATCTTTGAACGCACATTGCGCCCCTTGGTATTCC

ATGGGGCATGCCTGTTCGAGCGTCATTTGTACCCTCAAGCTCTGCTTGGTGTTGGGTGTTTGTCTTTTCCTTTGCGATTA

GACTCGCCTTAAAGCAATTGGCAGCCAGTGTTTTGGTATTAAGCGCAGCACAATTTGCGTCTTGAGCCCTAAGCCATGGC

AACCATAAAGCCTCTTCTCACTTTTGACCTCGGATCAGGTAGGGATACCCGCTGAACTTAA

>OTU_1015

GAAATGCGATAAGTAATGTGAATTGCAGAATTCAGTGAATCATCGAATCTTTGAACGCACATTGCGCCCCTTGGTATTCC

GAGGGGCATGCCTGTTCGAGCGTCATTACACCACTCAAGCTATGCTTGGTATTGGGCGTCGTCCTTAGTTGGGCgcgcCT

TAAAGACCTCGGCGAGGCCACTCCGGCTTTAGGCGTAGTAGAATTTATTCGAACGTCTGTCAAAGGAGAGGAACTCTGCC

GACTGAAACCTTTATTTTCTAGTTGACGTCGGCATCGAGGTAGGGATACCCGCTGAACTTAA

>OTU_1016

GAAATGCGATACGTAGTGTGAATTGCAGAATTCCGTGAACCATCGAATCTTTGAACGCATATTGCGCTCGAGCCCTCGGG

CAAGAGCATGTCTGCCTCAGCGTCGGTTTATACCCTCACCCCTctctcCTTTGGGAGAGCTGGTTAGCTTCTTGCTGGCC

TTAGGAGTGGATCTGGCTTTCCCATTTGGTTTATTCTGAATGGGTTGGCTGAAGCTTAGAGGCTTAAGCAAGGACCCAAT

ATGGGCTTCAACTGGATAGGTAGCACCGGCTTCTGCCGACTACACGAAGTTGTGGCTTGTGGACTTTGCTAGAGGCCAAG

CAGGAAACATGCTTTGCATGTCTTAAACTTTCGACTGAGCTCAGGCAAGGCTACCCGCTGAACTTAA

>OTU_1017

GAAGTGCGATAAGCAATGCGAATTGCAGAACCGTGAGTCATCAGATTtttGAACGCAACTGGCGCCGACTGGCTCTCCAG

TCGGCATACTTGTTTCAGTGTCTCTTTATCTCCTAACCCATATCATAATGCGAGCGATATCCTTCTCTTGCTAAACATGA

AAGCACTCTGCGCTCAGCGAGCGGCTCCGACTTGTTCGGGACCGCACTCAATGCAGCAGTCACATTGCTCACAATGTGAA

CTCATTGAGAGCTGGGGCAGTGCAGCGTAAGCTGCCAGTGCTTAGTCAcacaAACTTACTTGCATCTGAAATCAAGCAGG

ATCACCCGCTGAACTTAA

>OTU_1018

GAAATGCGATAAGTAATGCGAATTGCAGAATTCAGTGAGTCATCGAATCTTTGAACGCACATTGCGCCCTGTGGTATTCC

GCAGGGCATGCCTGTTCGAGCGTCATTTCAACCCTCAAGCTCTGCTTGGTGTTGGGCCCTGCCccccGCGGCCGGCCCTA

AAGACAGTGGCGGCGCCGTCTGGCTCTAAGCGTAGTACATCtctcGCTCTAGCGCCCGGCGGTGGCCTGCCAGAACCCCA

ACTCTGTGGTTGACCTCGGATCAGGTAGGGATACCCGCTGAACTTAA

>OTU_1019

GAAATGCGATAAGTAGTGTGAATTGCAGAATTCAGTGAATCATCGAATCTTTGAACGCACATTGCGCCcccTGGCATTCC

GGggggCATACCTGTTCGAGCGTCATCGCACCCCTCAAGCGCTGCTTGGTGTTGGGCGTCTTGTCCccccGTTGCGGggg

ACTCGCCCCAAAGACATCGGCGCTTGCACGGCGTCCGTGGCCCACAGCACAACGTGGTTGCCCGCAGACGCGCCCGCAAG

CGTGCCGCAAACGACGCCcccTTTTCGTGGCTCCTGGACGGACCTCGGATCAGGTAGGAATACCCGCTGAACTTAA

>OTU_1020

GAAATGCGATAAGTAATGTGAATTGCAGAATTCAGTGAATCATCGAATCTTTGAACGCACATTGCGCCCGGCAGTATTCT

GCCGGGCATGCCTGTCCGAGCGTCATTTCAACCCTCGAGCCGCTTTCAGTGGATCGGTGTTGGGGCACTGCCGTCAAACG

CAGGCCCTGAAATGAAGTGGCGGTCCCGCCGCGGCGCCcccATGCGTAGTAACGTCTAGTCTCGCATCGGGTCCCGGCGG

AGGCCTGCCGTCAAGCCTATTCTTAACTCAGAACAGTTTGACCTCGGATCAGGTAGGGTTACCCGCTGAACTTAA

>OTU_1021

GCTTGACCGTCTGATTTCAGCAGGATGCATGAACGGTATCAGGCAGAATACGATGAAAACCGCTTCGCTTGATCATCAGG

ACGGCTCTTCCGCCTGTGAAGGTTCCGGCGTGACGGCATGGGGCACAGCGTATGGTTCCTGGGGCCATAATGGCGGCGGT

GGCAATGCCAGTGGGTTGCATCATCAGCTTGGTGGTTTTGTTATGGGAGCTGACGCAGAAGCTTTCAGAACCTGGCGCAT

CGGTGGGCTTGTCAGTTATGGACATTCAGCCTTCAGCAGCGGAGCCATCCAGTCCTCGGGACATAGCAATGATGTCAGTG

TCGGTGGCTATGCCGGTACacacTGGGG

>OTU_1022

GAAATGCGATAAGTAATGTGAATTGCAGAATTCAGTGAATCATCGAATCTTTGAACGCACATTGCGCCCGCCGGCACTCC

GGCGGGCACGCCTGTCCGAGCGTCATTTCAACCCTCGGGCCCACCCCTCGCGGAGCAGGGCCCGGCGTTGACGGAGGCCG

CCCCGGCGGCACCCGCCcccTAAATTCAGTGGCGGTCGCGCCGCAGCCTCCCCTGCGTAGTAGCACACCTCGCACCGGAG

AGCGGCACGGCCACGCCTCGAAACCccccAATTtttCAGGTTGACCTCGGATCAGGTAGGAATACCCGCTGAACTTAA

>OTU_1023

GAAATGCGATAAGTAATGTGAATTGCAGAATCCAGTGAATCATCGAATCTTTGAACGCACCTTGCGCCCCTTGGTATTCC

GAGGGGCACACCTGTTGGAGTGTCATGAATACCTCAACCCTTATATGTTTTATCACTTATAAGGTGTTGGTTGTGAGTGT

TTGCCGGTCGCAAGATCGGCTCGCTTTAAAATCATTAGCTGGATCCACTTTGACCTGGTTCTACTCGGCGTAATAAGTTA

TTATCGTTGAGGACACTTTTCTTAGGAATAGTGGCCACAGTAAAGGTTGCGAACCGCTTCTAATACGTCTGCTTAGTCTT

CGGGCTATGCAACACTTTTATCTCTGACCTCCAATCAGGTAGGACTACCCGCTGAACTTAA

>OTU_1024

GAAATGCGATAAGTAGTGTGAATTGCAGAATTCAGTGAATCATCAAATCTTTGAACGCACATTGCGCCCCTTGGTATTCC

ATAGGGCATGCCTGTTCAAGCGTCATTTGTACCCTCAAGCTCTGCTTAGTGTTAGGTGTTTGTCTACTCCTTAGTGGTTG

GACTCACCTTAAAACAATTGGCAGCCAGTGTTTTAGTATTGAAGCGCAGCACATTTTGCAGTTCTGGCCCTGAACACTGG

CATCCAGTAAGCTCTTTTCCACTTTTGACCTCAGATTAGGTAGGGATACCCGCTGAACTTAA

>OTU_1025

GAAATGCGATAAGTAGTGTGAATTGCAGAATTCAGTGAATCATCGAATCTTTGAACGCACATTGCGCCCTTCGGTATTCC

GTTGGGCATGCCTGTTCGAGCGTCATTTAAACCTTCAAGCTATGCTTGGTGTTGGGTGTTTGTTCCGCCTCAGTGCGTGG

ACTCGCCTCAAATTCATTGGCAGCCGGTATGTTGGCTTCGTGCGCAGCACATTGCAATCGACTTCAGCAGACCcccTTCC

ACAAGACCTTttttAGTTTGACCTCGGATCAGGTAGGGATACCCGCTGAACTTAA

>OTU_1026

GAAATGCGATAAGTAATGTGAATTGCAGAATTCAGTGAATCATCGAATCTTTGAACGCACATTGCGCCCCTTGGTATTCC

GAGGGGCATGCCTATTCGAGCGTCATTATCACCCCTCAAGCCTAGCTTGGTGTTGAGACCTGCTGTCAAGGCAGTCTCTA

AAATCAGTGGCAGTGCTGTCAGGCTCTAAGCGTAGTAAATTCATCGCTATAGACACCTGGTGGACACTCGCCAGAACCcc

cccATTttttAATGATTGACCTCGGATTAGGTAGGGATACCCGCTGAACTTAA

>OTU_1027

GAAATGCGATACTTGGTGTGAATTGCAGAATCCCGTGAACCATCGAGTCTTTGAACGCAAGTTGCGCCCGAAACCTTCGG

GTCGAGGGCACGTCTGCCTGGGCGTCACGCATCGCGTCTCCcccTACCCACCTTGtgtgGGAAGGgggAGGAGGATGGCT

TCCCGTGCCTCACCGGGCGTGGTTGGCCTAAaaaaGGAGCCTCAAGTTATGCACTGTTGCGGCAATTGGTGGTAGACAAG

GCCTTGGCCTCGAATGCAATCTTGTGTCGTGCAGTACATGACAATTGTGGGCTCGTAGGACCCTGAGTTGTTCCCAATTG

GAAACAAACCGTTGCGACCCCAGGTCAGGCGGGGTTACCCGCTGAGTTTAA

>OTU_1028

GAAACGCGATAGGTATTGTGAATTGCAGAATCAGGGAATCATCGAATTtttGAACGCACCTTGCGCTCCCTGGTATTCCT

AGGAGCATGCCTGTTTGAGTGTTGATAGCCTCTCCAAACCTTGGTTttttATTAAATCGAGTGCTTTGGGTCCCTGGGCC

TGTAGCGGCGACGTTACTTGCCTTAAAAGGATCAAAGAAGACCCAATCGGATGTTAAGCATGATATCCTTTGGGGTTttt

GAACAAAATCAAAACTAATAACATACAACCTCAAATCAGGATAGGACTACCCGCTGAACTTAA

>OTU_1029

GAAATGCGATAAGTAATGTGAATTGCAGAATTCAGTGAATCATCGAATCTTTGAACGCATATTGCGCCCCTTGGTATTCC

GGggggCACACCTGTTCGAGCGCCATTTACACCCCTCAAGCATTGCTTGGTGTTGGGTTAGGTCCTTTTAGGGGACCCAC

CCGTAACTTGTAGGTGATCATAGGTGACCcccccTAGCGTAGCAGGCTAAACGACCCGCTCGGgggTGGCCCCTGTGGCC

CCGCCTAAaaaCTTTTATATCCAAAGGTTTGGCCTCGGATCAGGTGGGGATACCCGCTGAACTTAA

>OTU_1030

GAAATGCGATAAGTAATGTGAATTGCAGAATTCAGTGAATCATCGAATCTTTGAACGCACCTTGCGCCCTTTGGTATTCC

GAAGGGCATGCCTGTTTGAGTGTCATGAAACCTCACCCCACCTGGGTTtttCTAGGTGGTCGGTGGATTGGGCGTCTGCC

GTTACTGGCTCGCCTGAAATGCATAAGCGCCTTGGATGTAATACGTTTCATCCTCTCGGGTGGCTGATAACCCCACATCT

CATGATCTGGCCTCAAATCAGGTAGGGCTACCCGCTGAACTTAA

>OTU_1031

GAAATGCGATAAGTAATGTGAATTGCAGAATTCAGTGAATCATCGAATCTTTGAACGCACCTTGCGCTCCTTGGTATTCC

GAGGAGCATGCCTGTTTGAGTGTCATTAAATTCTCAACCTCACCCGTTTTCTGAACGGTTCTCCGAGGCTTGGATGTGGg

ggTTTGTGCAGGCTGCCTCAGCGCGGTCCGCTCCCCTGAAATGCATTAGCGAGTTCGTACTGAGCTCCGTCTATTGGTGT

GATAATTATCTACGCCGTGGACAGGGTTTAGACTCGCTTCTAACCGTCCGCATGGACAATACCTTTGACAATTTGACCTC

AAATCAGGTAGGACTACCCGCTGAACTTAA

>OTU_1032

GAATTGCGATAAGTAATGTGAATTGCAGAATTCAGTGAATCATCGAATCTTTGAACGCACCTTGCACTCTTTGGTATTCC

GAAGAGTATGTCTGTTTGAGTGTCATGAAACTCTCAACCccccTGTTTTGTAACGAGATAGGCGTGGGCTTGGATTATGG

TTGTCTGTCGGCGTAAAGTCGGCTCAACTGAAATATACGAGCAACCCATTTGAAATAGACGGTTTGACTCGGCGTAATAA

TTATTTCGCTGAGGACGTCTTCTTTAAAGTCAGTGGTGCTTCTAATGCGTATTATACATCATAAGCTTTAGACCTCAAAT

CAGTCAGGACTACCCGCTGAACTTAA

>OTU_1033

GAAATGCGATAAGTAATGTGAATTGCAGAATTCAGTGAATCATCGAATCTTTGAACGCATATTGCACCTCTGAGTATTCT

CTGAGGTATACCTGTTTGAGCGTCGTTTAACACTATAAAGCTTTGCTTtttATTAGAATTTGGTTAGAGATATCCAATTT

CTCAAACAAAGTGGCGTTATTTTCTTGTCCGATATACAGTGAAAATGTATACTTGACCATAAAATAATCATTTCGCCTAT

CTTACATTAAATTTGTTCGACCTCAAATCAAGTAAGACTACCCGCTGAACTTAA

>OTU_1034

GAAATGCGATAAGTAATGTGAATTGCAGAATTCAGTGAATCATCGAATCTTTGAACGCACCTTGCGCTCCTTGGTATTCC

GAGGAGCATGCCTGTTTGAGTGTCATGAAACTCTCAACCTTCAACTTttttATTAAGGCTGAAGGCTTGGACTTGAGCGT

TGCTGGTTtttATTAACCGGCTCGCTTGAAATGAATTAGCAGATCCTTtttGTAATCGGTTCCACTCGACGTGATAAGTA

TTTCGCCGAGGACATACGAAAGTATGGCCGAGATAAAGAAAGTCTTTAGATCCGCTTCTAATTCTTAGATAGAGCTTGCT

CTACTAAACCCCATTTTATGATCTGGCCTCAAATCAGGTAGGACTACCCGCTGAACTTAA

>OTU_1035

GAAATGCGATAAGTAATGTGAATTGCAGAATTCAGTGAATCATCGAATCTTTGAACGCACATTGCGCCCGCCAGTATTCT

GGCGGGCATGCCTGTTCGAGCGTCATTTCAACCCTCAAGCCTCGCTTGGTGTTGGGGATCGGCTCCTTCCGGGAGCCGCC

cccGAAATCTAGTGGCGATCATACCAACGCATCCTTAGCGTAGTAACATACCCTCGCTCTGGAACGTGGTGCGGTCACGC

CGTTAAACACCCCACTTCTCAAGGTTGACCTCGGATCAGGTAGGAATACCCGCTGAACTTAA

>OTU_1036

GAAATGCGATAAGTAATGTGAATTGCAGAATTCAGTGAATCATCGAATCTTTGAACGCACATTGCGCCcccTGGTATTCC

GGggggCATGCCTGTCCGAGCGTCATTACAACCCTCAAGCCTAGCTTGGTGTTGGGCTCCGCGGCCTAGCGGGCCCTAAA

ATCAGTGGCGGTGCCGTCCAGCTCCGAGCGTAGTAACTCTTCTCGCTCTGGAGGCCTGGGCGCGTGCTTGCCAGCAACCc

cccAATTTTATCAGGTTGACCTCGGATCAGGTAGGGATACCCGCTGAACTTAA

>OTU_1037

GAAATGCGATAAGTAATGTGAATTGCAGAATTCAGTGAATCATCGAATCTTTGAACGCACATTGCGCCCCTTGGTAGTTC

CGAGGGGCATGCCTGTTCGAGCGTCATTACCACCACTCAAGCCTATGCTTGGTATTGGGCGTCGGTCCTTAGTTGGGCGT

GCCTTAAAGACCTCGGCGAGGCCACTCCGGCCTTTAGGCGTAGTAGAATTTATTCGAACGTCTGTCAAAGGAGAGGAACT

CTGCCGACTGAAACCTTTATTtttCTAGGTTGACCTCCGGATTCAGGTAGGGATACCCGCTGAACTTAA

>OTU_1038

GAAATGCGATAAGTAGTGTGAATTGCAGAATTCAGTGAATCATTGAATCTTTGAACGCATATTGCGCCCCTCGGTATTCC

GTGGGGCATGCCTGTTCAAGCGTTATTTACCTCTTAAGCTCTGCTTGGTGTTGGGCGTTGTCCCGCTTCGcgcgAGGACT

TGCCTCAAAGGTATTGGCAGCGGTCGTGCTACCCCTCGCGTAGTATATTGCGCTTCTTGAGGCGGTCGATTAGCGTTTAT

AAAGCTTATATTATATCTTGACCTCGGATCAGGTAGGGATACCCGCTGAACTTAA

>OTU_1039

AAAGCGCGATAGGTAATGCGAATTGCAGACGTGAATCATCGAATCTTTGAACGCACATTGCGCCCCTTGGTATTCCGAGG

GGCATGCCTGTTCGAGCGTCATTACACCACTCAAGCTATGCTTGGTATTGGGCGTCGTCCTTAGTTGGGCgcgcCTTAAA

GACCTCGGCGAGGCCACTCCGGCTTTAGGCGTAGTAGAATTTATTCGAACGTCTGTCAAAGGAGAGGAACTCTGCCGACT

GAAACCTTTATTtttCTAGGTTGACCTCGGATCAGGTAGGGATACCCGCTGAACTTAA

>OTU_1040

GAAATGCGATACGTAATGTGAATTGCAGAATTCAGTGAATCATCGAATCTTTGAACGCACCTTGCACTCTTTGGTATTCC

GAAGAGTATGTCTGTTTGAGTGTCATGAAACTCTCAACCcccTCATTTTGTAATGAAGTGTAGCGTGGGCTTGGATTATG

GCTGTCTGTCGGCTTAACCGCCGGCTCAGCTGAAATACACGAGCAACCCAGTTTAAAACCCTGACGGCTTGACTCGGCGT

AATAATTATTTCGCTGAGGACGTCTAACCTTTAAATCGTTAGTGGTGCTTCTAATGCATTATTCATTAAGCTTTAGACCT

CAAATCAGGCAGGACTACCCGCTGAACTTAA

>OTU_1041

GAAATGCGATAAGTAGTGTGAATTGCAGAATTTAGTAAATTATTGAATCTTTAAACGCACATTGCGCCCCTTGGTATTCT

ATAGGGCATGCCTGTTTAAGCGTTATTTATACCCTTAAGCTCTGCTTAGTGTTAGGTGTTTGTCTCCTCTAGGAGACTCG

CCTCAAAACAATTAGCAGCTAGCATATTAGTATTAGAGCGTAGCACAAGTCGCGCTTCTGTCTATATTTGTTAGCATCTA

GCAAGACTATTtttCACTCTTGACCTTGGATTAGGTAGGGATACCTGCTGAACTTAA

>OTU_1042

GAAATGCGATACTTGGTGTGAATTGCAGAATCCCGTGAACCATCGAGTCTTTGAACGCAAGTTGCGCCCCAAGCCTTCTG

GCCGAGGGCACGTCTGCCTGGGTGTCACAAATCGTCTCCTCACCATCATCCTTTGCTGATGCGGGACGGAAGCTGGTCTC

CCGTgtgtTACCGCACGCGGTTGGCCTAAATCCGAGCCAAGGACGCCTGGAGCGTACCGACATGCGGTGGTGAACTTGAT

CCATTACATTTTATCGGTCGCTCTTGTCCGGAAGCTGTAGATGACCCAAAGTCCATATAGCGACCCCAGGTCAGGCGGGA

TTACCCGCTGAGTTTAA

>OTU_1043

GAAATGTGATAAGTAGTGTGAATTGCAGAATTTAGTGAATTATTGAATCTTTGAACGTATATTGCGCCCCTTGGTATTCT

ATGGGGCATGCCTGTTTGAGCGTTATTTGTACCTTTAAGCTCTGCTTGGTGTTGGGTGTTTGTCTACTCCTTGGTAGTTG

GACTTGCCTTAAAACAATTGGCAGCCAGTGTTTTAGTATTGAAGCGCAGCACATTTTGCGGTTCTGGCCCTGAACACTGG

CATCTAGTAAGCTCTTTTCTACTTTTAACCTTGGATCAGGTAGGGATACCTGCTGAACTTAA

>OTU_1044

GAAATGCGATAAGTAATGTGAATTGCAGAATTCAGTGAATCATCGAATCTTTGAACGCACATTGCGCCCCTCGGTATTCC

GGggggCATGCCTGTTCGAGCGTCATTACACCACTCAAGCCTCGCTTGGTCTTGGGCGTCTCGCGGTCCGCCGcgcgcCC

CAAAGTCTCCGGCTGAGCCGTCTGTCTCGAAGCGTTGTGATCTATCAAATCGCTTGCGGGATCGGTGGCCTCGGGCCGTT

AAACCccccATATTTTATCAGGTTGACCTCGGATCAGGTAGGGATACCCGCTGAACTTAA

>OTU_1045

GAAGTGCGATAAGCAATGCGAATTGCAGAACCGTGAGTCATCAGATTtttGAACGCAATTGGCGCCGGTTGGTTCTCCAG

CCGGCATGCTTGTTTCAGTGTCTTGTTTTCTCCTCACCCAAATCTTAATGTGAgagaTACCCTTCTCTTACCAAGTACGA

AAGCACTCTGAGCTCTGCACGCGGCTTCATTTCGGTGAATCCGCACTCAATGCAGCAGTCACATTTTACAATGTGAACTC

ATTGAGAGTTGAGTCAGCGCAGCTTGTCTGCAAGTGTTTAGATCTCAAAACTCTTCTTGTATCTGAAATCAAGCAGGATC

ACCCGCTGAACTTAA

>OTU_1046

GAAATGCGATACTTGGTGTGAATTGCAGAATCCCGTGAACCATCGAGTCTTTGAACGCAAGTTGCGCCCTAAGCCTTCTG

GCCGAGGGCACGTCTGCCTGGGTGTCACAAATCGTCGTCCcccccATCCTCTCGAGGATATTGGACGGAAGCTGGTCTCC

CGTgtgtTACCGCACGCGGTTGGCCAAAATCCGAGCAAAGGACGCAAGGAGCGTCCCGACATGCGGTGGTGAATTAAAAC

TTCGTCATACCGTCGGCCGCTCCTGTCTGGATGCTCTTGATGACCCAAAGTCCTCAACGCGACCCCAGGTCAGGCGGGAT

CACCCGCTGAGTTTAA

>OTU_1047

GAAATGCGATACGTAATGTGAATTGCAGAATTCCGTGAATCATCGAATCTTTGAACGCACATTGCGCCCCTTGGTATTCC

AGGgggCATGCCTGTTTGAGCGTCATTTCCTTCTCAAACATTCTGTTTGGTAGTGAGTGATACTCTTTGGAGTTAACTTG

AAATTGCTGGCCTTTTCATTGGATGTTtttttttCCAAAGagagGTTTCTCTGCGTGCTTGAGGTATAATGCAAGTACGG

TCGTTTTAGGTTTTACCTAACTGCGGCTAATCTTtttttATACTTGAGCGTATTGGAACGTTATCGATAAGAAGagagCG

TCTAGGCGAACAATGTTCTTAAAGTTTGACCTCAAATTCAGGTTAGGAGTACCCGCTGAACTTAA

>OTU_1048

GAAATGCGATAAGTAGTGTGAATTGTAGAATTTAGTGAATTATCGAATCTTTAAACGTATATTGCGCCCCTTGGTATTCT

ATAGGGCATGCCTGTTCGAGCGTTATTTATACCTTTAAGCTCTGCTTGGTGTTAGGTGTTTGTCTCGCCTCTGCGTGTAG

ACTCGCCTTAAAATAATTGGTAGCCAGCATATTGATTTTAGAGTGTAGTACATCTCGCGCTTTGTACTTATAACAACGAC

GTCTAAAAGTACATTtttACACTCTTAACCTTGGATTAGGTAGGGATACCCGCTGAACTTAA

>OTU_1049

GAAATGCGATAAGTAATGTGAATTGCAGAATTCAGTGAATCATCGAATCTTTGAACGCACATTGCGCCcccTGGTATTCC

GGggggCATGCCTGTCCGAGCGTCATTACTGCCCTCAAGCGCGGCTTGtgtgtTGGGCCCTCGTCCccccccGGgggACG

TGCCCGAAAGGCAGTGGCGGCGCCGTGCCCGGACCTCGAGCGTATGGGGCTTTGTCACCCGCTCTGGAGACCCGGCCGGC

GGCCGTCGGACCACCCCAACCTTtttAACCGGTTGACCTCGGATCAGGCAGGGATACCCGCTGAACTTAA

>OTU_1050

GAAATGCGATAAGTAGTGTAAATTGCAGAATTCAGTGAATTATCAAATCTTTAAACGCACATTGCGCCCCTTAGTATTCT

ATAGGGCATGCCTATTCGAGCGTCATTTATACCCTTAAGCTTTGCTTAGTGTTAGGTGTTTGTCCTCTCCTAGCGTTTAG

ACTCACCTTAAAGTAATTAGCAGCTAGTATTTTAGTATAGAAGCGCAGCATatatCGCAATTCTAGCTCTATACTTGCGT

CTATAAGCCTATTTCACGTTTGACCTCAGATTAGGTAGAGATACCTGCTGAACTTAA

>OTU_1051

GAAATGCGATAAGTAGTGTGAATTGCAGAATTCCGTGAATCATCGAATCTTTGAACGCACATTGCGCCCCTTGGTATTCC

TTGGGGCATGCCTGTTCGAGCGTCATTTACACCCTCAAGCTCTGCTTGGTGTTGGGCGTCTGTCCCGCCTCCCGGGCGTG

GACTCGCCTCAAAGCGATTGGCGGCTGGTATGATGGCCTGGAGTGCAGCAGGATTGCGCCCCTTGTCCGGAGGCCGGCTC

CCACAAGCTAAACCcccACTATTtttGACCTCGAATCAGGTAGGGATACCCGCTGAACTTAA

>OTU_1052

AAACTGCGTTACTTAATGCGAATTGCAGACACATTGAGCATCGACACTTTGAACGCATATTGCGGCATTAGGATTTATTC

CTGGTGCCACGTCTGTCTGAGGGCTGTTTAACAGAGAAGTCATAACTATCAACTAGGCTTCCTCCAGAGCCTTGATAGTC

GCTTGCACTTAGCTACTTTTCTGTCCATAGCAGATCGGTACCTTTTGCTGGACGTGTAATGCATAGCCCTAGGTGCAAaa

aCacacaTGACAGAGGTGGCAACAACTGTTGGCGGTCTCCcccccGTGCGCGGATAGGGATTCAAGCGTCGTTAAGTGAG

CTACTGATGTTTTCTGTCTTCTAGCCTCAGATCAGACGTGACAACCCGCTGAACTTAA

>OTU_1053

GAAATGCGATAAGTAATGTGAATTGCAGAATTCAGTGAATCATCGAATCTTTGAACGCACCTTGCGCCTTTTGGTATTCC

GAAAGGCATGCCTGTTTGAGTGTCATGAAATCTCAATCtctcAAGTTCTTTTGAACTGGTTGGATTGGACTTGGGTGATC

TGCCAGCAATGGCTCGCCTCAAATGACTTAGTGGAACATCCCACATCAGTGTCAGACGTAATAAGTTTCGTCTTTCCTTG

TGGTGATGACTGCTTAGAACCTGCCATCGCGCAACCTTTTGACTTTGACCTCAAATCAGGTAGGGCTACCCGCTGAACTT

AA

>OTU_1054

GAAATGCGATAAGTAGTGTGAATTACAGAATTCAGTAAATTATTAAATCTTTAAACGCACATTGCGCCCCTTAGTATTCT

GTAGGGCATGCCTGTTCGAGCGTTATTTACCTCTTAAGCTCTGCTTAGTGTTAAGCGTTGTCCCGCTTCGcgcgAGGACT

CGCCCTAAAGGTATTAGCAGCGGTTGTGCCACCCCTCGCGCAGCATATTACGCTTCTTAAGGCGGTTGATTAGCGTCTAC

AAAGCTTACATTATATCTTAACCTCGGATCAGGTAGGGATACCCGCTGAACTTAA

>OTU_1055

GAAACGCGATAGGTATTGTGAATTGCAGAATCAGGGAATCATCGAATTtttGAACGCACCTTGCGCTCCCTGGTATTCCT

AGGAGCATGCCTGTTTGAGTGTTGATAGCCTCTCCAAACCTTGGTTttttATTAAATCGAGTGCTTTGGGTCCCTGGGCC

TGTAGCGGCGCGTTACTTGCCTTAAaaaGGATCAAAGAAGACCCAATCGGATGTTAAGCATGATATCCTTTGGGGTCTTT

GAACGACTATAAACTATACATACAACCTCAAATCAGGTAGGACTACCCGCTGAACTTAA

>OTU_1056

GAAATGCAATAAGTAGTGTAAATTGCAGAATTTAGTAAATCATCAAATCTTTAAACGCACATTACGCCCCTCGGTATTCC

GTAGAGTATGCCTGTTCAAGCGTTATTTACCCCTTAAGCTCTGCTTAGTATTAGGCGTTGTCCCGCTTCGcgcgAGGACT

TGCCCTAAAGGTATTAGCAGCGGTCGTGCCACCCCTCGCGCAGCATATTACGCTTCTTAAGGCGGTCGATTAGCGTCTAT

AAAGCTTATATTACATCTTAACCTCGGATTAGGTAGGAATACCCGCTAAACTTAA

>OTU_1057

GAAATGCGATAAGTAGTGTGAATTGCAGAATTCAGTGAATCATCGAATCTTTGAACGCACATTGCGCCCTATAGTATTCT

GTAGGGCACTCCTGTTCGAGCGTCATTTGTACCTTCAAGCTTTGCTTGGTGTTGGGTGTTTGTCCTTTCCCTCGTGTTTG

GACTCGCCTTAAAATGATTGGCAGCCAGTGTTTTGGTATTGAACGCAGCACAATTTGCGATTCTGACTGGAAATACTTGC

GTCCAGAAGCATTtttCACGTTTGACCTCGGATCAGGTGGGGATACCCGCTGAACTTAA

>OTU_1058

GAAATGCGATAAGTAGTGTGAATTGCAGAATTCAGTGAATCATCGAATCTTTGAACGCACATTGCACCCCTCGGTATTCC

GGggggTATGCCTGTTCGAGCGTCATTACacacCTCAAGCTCCGCTTGGTATTGGGTTCCCGTCCGCTCGGACGCGCCCG

AAACTCCTCGGCGGCGGCGCCTTTGCTTCAAGCGTAGTAAAAGATTCCATCTCCTCGCTTTGGAAGCGGAGGTTCTGCCC

GCCGGACGACCACCCATCCTTTCTCAAAGGTTGACCTCGGATCAGGTAGGGATACCCGCTGAACTTAA

>OTU_1059

GAAATGCGATAAGTAATGTGAATTGCAGAATTCAGTGAATCATCGAATCTTTGAACGCACATTGCGCCCGTTAGCATTCT

AGCGGGCATGCCTGTCCGAGCGTCATTTCAACCCTCAGGGTCCCCGTTCCGGCGGGGAACCTGGTGTTGGGGATCGGCCC

GCCCCGTGCGGCGCCGTCCcccAAATTCAGTGGCGGTCTCGCTGCAGCCTCCCCTGCGTAGTAGTTACAACCTCGCATCG

GAGCTCAGCGCGGCCACGCCGTAAAACCcccGACTTTCTGAACGTTGACCTCGGATCAGGTAGGAATACCCGCTGAACTT

AA

>OTU_1060

GAAATGCGATAAGTAATGTGAATTGCAGAATTCAGTGAATCATCGAATCTTTGAACGCACATTGCGCCCTCTGGTATTCC

GGgggCATGCCCGTTCGAGCGTCATTACACCACTCAAGCCTCGCTTGGTATTGGGCGTCGCGACATCGCGTGCCTCTAAT

TTCTCGGCTGGACGGTCTGAATCTCAGCGTTGTGGATTATTCAATTCGCTGGCGAGGACGACCGGACGCGCCGTTAAACC

CTTCTTTACAGGTTGACCTCGGATCGGGTAGGGATACCCGCTGAACTTAA

>OTU_1061

GAACGcgcgAAACGTAATGTGAATTGCAGAATTCAGTGAATCATCGAATCTTTGAACGCACCTTGCGCTCCTCGGCATTC

CGAGGAGCATGCCTGTTTGAGTGTCGTGAAGTCTCATCGGAAAGGgggTCCCCTCTGTGGGTTTCCATTTTCGGAATTGG

ACGGTCTTTTGCCTCTCGCCGGAGGCTCGTCTTCAAATTCATCGGCGAGGTTTCGAGTCCccccATTGAAGACGCGTGAT

AAGTCTCGCGTCGTCCCCGGGGAGACGCGCCCCGCCcccGTCACTCTATTCTTTAACGCCTTGACCTCAAATCAGGCAGG

ATTACCCGCCGAACTTAA

>OTU_1062

GATAAAAGCAGGAGAAGAGACGCGGAGGTGGCTGTCAAATGACGCTTAATATAGAGTTTATGATGGAAACCATACCGCTG

GTTTTAAAGGCCCTGCCCCTTACGGCGTTTATATCCGTGGCCTCAATGGCAATAGGAGGGCTTCTGGGACTTTTAATAGG

GCTTATAAGATATTACAGGGTGCCTGTTTTAAACCAGATTtttAAAATATACGTATCCCTTGTAAGGGGATTTCCGCTTG

TTATACAGCTTTATATTGCTTATTTCTGTATTCCGTATTATGTGAACATGTACA

>OTU_1063

GAAATGCGATACGTAATGTGAATTGCAGAATTCAGTGAATCATCGAATCTTTGAACGCACATTGCGCCCTTTGGTATTCC

GAAGGGCATGCCTGTTCGAGCGTCATTATCAACCATCAAGCTCTGCTTGGCATTGATTGTCACCccccTCCCCGGggggC

GCGATCTAAACTGGTCGGCTGTGGTCTGTGAACCGTTGGCGTTAGTAAaaaCTTCCCGCTACTCGGTAACAGGTCGCAAG

CCTCCAAAACCCAACACCTCAAGGTTGACCTCGGATCAGGTAGGGATACCCGCTGAACTTAA

>OTU_1064

GAAATGCGATAACTAATGTGAATTGCAGAATTCAGTGAATCATCGAGTCTTTGAACGCACATTGCGCCCTTTGGCATCCC

GAAGGGCATGCCTGTTCGAGCGTCATTTCACCAATCGAGCCCTTTGGCTTGGTGTTGGGTGCCGTCCCGCCTCCGcgcgc

gGACGCTCCCTAAAATCATCGGCGGTGCAGTACCGGCTCTAAGCGCAGCAGATGTTCGCTTGGTGGGCTCGGTGCAGCAG

CGGCCGCGACGACAACCTCTAGGTTGACCTCGGATCAGGTAGGGATACCCGCTGAACTTAA

>OTU_1065

GAAGCGCGAAATGTAGTGTGAATCGCAGAATTTGTGAATCATCGAATCTTTGAACGCACATTGCGCCTCTCGTGAGGGAG

GCATGCCTGTCTGAGCGTCGTCTAAACCTCTAGGCTTCATTCCTTAGGTGGAGCCTGTCTTGGGTCTCGCCccccGCGGC

GTCGCCTGAATGTCATTGGTCTCATCCGTGGACGAGTCCCAGTAGGGCGGACCATGGTGGGACCCTAAGCCCCTCTGAAT

TTGACCTCAGATCAGGTAGGAACACCGCCTGAACTTAA

>OTU_1066

GAAATGCGATAAGTAATGTGAATTGCAGAATTCAGTGAATCATCGAATCTTTGAACGCACCTTGCGCTCCTTGGTATTCC

TTGGAGCATGCCTGTTTGAGTGTCTTGAATATCTCCTCTCATCCATTtttttAATTAAAGATGTGGTCGAGAGGGTGCTT

GGGCTTGCATGGTATTACTCCTTATGCTTGCCTTAAATGCATTAGCTGGATTTCAGTAGAGTTGTTAAGTAACATTGAAA

ACTCTTGTGATCGAAATCTGCTTCTAATTCATCTTGAGCAATCGAGATatatatTTCAACTTTGGCCTCAAATCAGGTAG

GACTACCCGCTGAACTTAA

>OTU_1067

GAAATGCGATAAGTAGTGTGAATTGCAGAATTCAGTGAATCATCGAATCTTTGAACGCACATTGCGCCCCTTGGTATTCC

ATGGGGCATGCCTGTTCGAGCGTCATTTACACCCTCAAGCTCTGCTTGGTGTTGGGCGTCTGTCCCGCTTCGTGCGCGGA

CTCGCCCCAAACGTATTGGCAGCGATCTTTGCCAGCTTCTCGCGCAGCACATTGCGTTTCTTGAGGCTTTGGCGGATCGG

CGTCCATCAAGATTCTACAGTTTGACCTCGGATCAGGTAGGGATACCCGCTGAACTTAA

>OTU_1068

GAAATGCGATAAGTAGTGTGAATTGCAGAATTCAGTGAATCATCGAATCTTTGAACGCACATTGCGCCCCTTGGTATTCC

ATGGGGCATGCCTGTTCGAGCGTCATTTGTACCCTCAAGCTTTGCTTGGTGTTGGGTGTTTGTCCTTTCCCCAGCGTTTG

GACTCGCCTTAAAACAATTGGCAGCCAGTGTTTTGGTATTGAAGCGCAGCACAAGTCGCGATTTAATCCAGTTTCACTTG

CGTCCATAAGCCTTtttttCACTTTTGACCTCGGATCAGGTAGGGATACCCGCTGAACTTAA

>OTU_1069

GAAATGCGATAAGTAGTGTGAATTGCAGAATTCAGTGAATCATCGAATCTTTGAACGCACATTGCGCCCCTCGGTATTCC

GTGGGGCATGCCTGTTCGAGCGTCATTTAACCCCTCAAGCCTAGCTTGGTATTGGGTGCTTGTCCCGCCTCTCGcgcgGC

GACTCACCTCAAAGTCATTGGCAGCCCGCATCTCGCCGGCCGTGAGCGCAGCACAGACGCGCTCTTGGCAACGACGGATC

GGCTCTCCAAAAGCTTATTTCAACCACTGACCTCGGATCAGGTAGGGATACCCGCTGAACTTAA

>OTU_1070

GAAATGCGATAAGTAGTGTGAATTGCAGAATTCAGTGAATCATCGAATCTTTGAACGCACATTGCGCCCCATGGCATTCC

GTGGGGCATACCTGTTCGAGCGTCATTTCAACCCTCGGGCGGTCCTTTTGGTCCGTCTGGACTTGGGTGTCGTCTAATGG

CGTGCCTTAAAAGAATTGGCGGTGTAGGTGCCGGCTTCGAGCGTAGCAGATGCGATTCGCTCTGGGAGCTGGCGCTCACC

TTGCCAGAAAAGTCTCTGAAGAGCTGACCTCGGATCAGGTAGGGATACCCGCTGAACTTAA

>OTU_1071

GAAATGCGATAAGTAATGCGAATTGCAGAATCCGTGAGTCATCGAATCTTTGAACGCATATTGCGCCCTCTGGTATTCCG

GggggCATGCCTGTTCGAGCGTCATTATCACCCCTCAAGCCCGGCTTGTTGTTGGACGTCGGCGGCGGCCTTCAGTGGCG

TACCGCCCGTCTCAAAGATAATGACGGCGTCTGTGAGGACACCTGTACACTGAGCTTCCGGGCACGTACTAGGCTGACAC

TCGCAGACCCGGTCTCTAGGGTTCGCCTCGGCGACCCTAACTTtttACCAAGGTTGACCTCGGATCAGGTAGGAATACCC

GCTGAACTTAA

>OTU_1072

GAAATGCGATACTTGGTGTGAATTGCAGAATCCCGCGAATCATCGAGTCTTTGAACGCAAGTTGCGCCCGAAGCCATCTG

GTCGAGGGCACGTCTGCCTGGGTGTCACGCATCGTTGCCcccAACCCCATCGCCTCTCCAAGAGACGAGGGCGGTCTGCG

GGGCGGACATTGGCCTCCCGTGAGCTTTCGCTCGCGGCTGGCCTAAAAGCGAGTCCTCGGCGACGAGCGCCACGACAATC

GGTGGTTGACAAACCCTCGTGTCCCGTCGTGCGCGGCTCGCCGCTCGTCTTGTGCTCTGTGACCCTGTAGCGTCGCGCTC

GCGACTCCTCCGACGCGACCCCAGGTCAGGCGGGACTACCCGCTGAATTTAA

>OTU_1073

GAAATGCGATAAGTAATGTGAATTGCAGAATTCAGTGAATCATCGAATCTTTGAACGCACATTGCGCCCGCTAGTATTCT

GGCGGGCATGCCTGTTCGAGCGTCATTTCAACCCTCAAGCCcccGGGCTTGGTGTTGGGGATCGGCGAGCCTCCGCGCCC

GCCGTCCCCTAAATCTAGTGGCGGTCTCGCTGTAGCTTCCTCTGCGTAGTAGCACACCTCGCACTGGGAAACAGCGCGGC

CACGCCGTTAAACACCCAACTTCTGAAAGTTTGACCTCGGATCAGGTAGGAATACCCGCTGAACTTAA

>OTU_1074

GAAATACAATAAGTAGTGTGAATTGCAGAATTTAGTAAATTATTGAATCTTTGAACGTATATTACGCCCCTTAGTATTCC

GTAGGGCATGCCTGTTCGAGCGTTATTTACCTCTTAAGCTCTGCTTAGTGTTGGGCGTTGTCCCGCTTTGCGCGAGAACT

TGCCCTAAAGGTATTGGTAGCGGTCGTACCACCCCTCGCGCAGCATATTACGCTTCTTAAGGCGGTCGATAGCGTCTACA

AAGCTTACATTATATCTTAACCTCGGATTAGGTAGGGATACCCGCTGAACTTAA

>OTU_1075

GAATTGCGATATGTAATGTGAATTGCAGAATTCAGTGAATCATCGAATCTTTGAACGCACATTGCACCCTCTGGTAATCC

AGAGGGTATGCCTGTTTGAGTGTCATTAACAACATCAAATCCAGATTtttGCTGTGATTTGGATCTTGAAGGTTTTGCTG

ACTGACAAAGTTGGCTCCTTTTAAATGCATTAGCAGATTGCAAACAACTGGCCTCAGTTTGATAGATTGGATTATCCTTG

CTGTCGCTAGAAATTGTTTGATTGCTTCAAATTGTCTTTGGACTGCCATTAGGCAATACATTTTAAGTCTTGACCTCAAA

TCAGGTAGGATTACCCGCTGAACTTAG

>OTU_1076

GAAATGCGATAAGTAGTGTGAATTGCAGAATTCAGTGAATCATCGAATCTTTGAACGCACATTGCGCCTCCTGGTATTCC

GGGAGGCATGCCTGTCCGAGCGTCATTAAAACAAACTCAAACATGATTTTATTTTGTTTGGTCTTGAgagaTGAGCAGCA

AGATTTATTCTAAACTGCTCACTCATCGAAATCTAATGGCAGATTAATAAAGTATGGATTGTCCTAGTGTGATAAGATTT

TCATGTCACTTGGTTAGCCTATTCTACAATCTTGCTGATTTATACTCCCATTtttATAATAAACGACCTCGGATCAGGTA

GGGATACCCGCTGAACTTAA

>OTU_1077

GAAATGCGATACTTGGTGTGAATTGCAGAATCCCGTGAACCATCGAGTCTTTGAACGCAAGTTGCGCCCGAGGCCTTTGG

CTGAGGGCACGTCTGCCTGGGCGTCACGCATCGCAGCCcccTCACTCCCCACCCTTAGTGGGATGGGGAGGAAGATGGTT

TCCCGTGCCTCACCGGGCACGGTTGGCCTAAAATTGGAGCCTAAGGCATTGAGTTGTCGCGGCAATAGGTGGTGAACAAG

GCCTTGGCCGTGCAAGCAACCAGTCGTGCAGCCCTTTGACAATCGAAGCTCGTAGGAGACCCTATGATGTTGCCTTTTGG

TGACACAAACTGTTGCGACCcccAGGTCAGGCGGGGCTACCCGCTGAATTTAA

>OTU_1078

GAATTGCGATAAGTAATGTGAATTGCAGAATTCAGTGAATCATCGAATCTTTGAACGCACCTTGCGCCTTTTGGTATTCC

GAAAGGCATGCCTGTTTGAGTGTCATGAAATCTCAATCCCCTCGGGTTtttCGACCTGCTGGGTGACTTGGATGTGGGCG

TCTTGCCAGCTTTCTGTCTGGCTCGCCTCAATGTTGTCAGTGGGATACCTTTGCCCAGCATCAGACGTAATAAGTTTCGT

TTGGTCGGGGTGTCGGGGCCTGCTCATAACCCATCGCGTGCATTTCGGTGCgcgcTTTACTCTTCGATCTGACCTCAAAT

CAGGTAGGGCTACCCGCTGAACTTAA

>OTU_1079

GAAATGCGATAAGTAGTGTGAATTGCAGAATTCAGTGAATCATCGAATCTTTGAACGCACATTGCGCCCCTTGGTATTCC

ATGGGGCATGCCTGTTCGAGCGTCATTTGTATCCTCAAGCTTAGCTTGGTGTTGGGTGTTTGTCTATGCTTGTATTTAGA

CTCACCTCAAAGTAATTGGCAGCTAGTGTCCTGGTATAGAAGTGCAGCACATTTTGCAACTCTAGCCTAGAACACTGGTG

TCCAGTAAGCTTCTCCACTTTTGACCTCGGATCAGGTAGGGATACCCGCTGAACTTAA

>OTU_1080

GAAATGCGATACGTAGTGTGAATTGCAGAATTCCGTGAACCATCGAATCTTTGAACGCATATTGCGCTCGAGCCTCGGCA

AGAGCATGTCTGCCTCAGCGTCGGTTTATAACCTCACCCCTCTCCTCCTTTTGGACGTAGCTGGTTAGCTTCTAGCTGGC

CTTAGGAGTGGATCTGGCTTTCCCATTTGGTTTATTCTGAATGGGTTGGCTGAAGCTTAGAGGCTTAAGCAAGGACCCGA

TATGGGCTTCAACTGGATAGGTAGCACCGGCTTCTGCCGACTACACGAAGTTGTGGCTTGTGGACTTTGCTAGAGGCCAA

GCAGGAAACATGCTTTGCATGTCTTAAACTTTCGACTGAGCTCAGGCAAGGCTACCCGCTGAACTTAA

>OTU_1081

GAAATGCGATACGCAATGCGAATTGCAGAATTCCGCGAGTCATCAGATCTTTGAACGCAACTGGCGCCGGTTTACCGGCA

TGTTTGTTTCAGTgtgtTTGGAGAGTTAAACAATCACTTCCAGCAGAATGCGATTGAGGAGCTTGCTCTTCtctcGTTAA

ATGCGAAGGTGTGAGGCTTTGTTCCTTACACTAGACACGAAGTAGTCACGCTTGTCCGTGATCTCGTATCTGGTGTTTTA

AGGGAACTCATGCGTACCACCTGAAGTTCTTTTCTCCATATTCGACACCTGAAATCAAGCAAGGCTACCCGCTGAACTTA

A

>OTU_1082

GAAATGCGATAAGTAGTGTGAATTGCAGAATTCAGTGAATCATCGAATCTTTGAACGCACATTGCGCCCCTTGGTATTCC

ATGGGGCATGCCTGTTCGAGCGTCATTTGTACCCTCAAGCTCTGCTTGGTGTTGGGCGTTtttGTCTTTTATTAGACTCG

CCTTAAAGTAATTGGCAGCCAGTGTTtttGGTAGTAAGCGCAGCACAATTTGCGTCTTGGTCCCTAAACAGTGGCATCCA

CAAAGCCTCTTTATCACTTTTGACCTCGGATCAGGTAGGGATACCCGCTGAACTTAA

>OTU_1083

GAAATGCGATAAGTAATGTGAATTGCAGAATTCAGTGAATCATCGAATCTTTGAACGCACATTGCGCCCTCTGGTATTCC

GGAGGGCATGCCTGTCCGAGCGTCATTGCTGCCCTCAAGCACGGCTTGtgtgtTGGGCCCCGTCCTCCTTCCGGgggACG

GGTCCGAAAGGCAGCGGCGGCACCGCGTCGGTCCTCAAGCGTATGGGGCTTTGTCACTCGCTTTGTAGGCCTGGCCGGCG

CTTGCCGATCAACCAAACTTtttATCAGGTTGACCTCGGATCAGGTAGGGATACCCGCTGAACTTAA

>OTU_1084

GAAATGCGATACTTGGTGTGAATTGCAGAATCCCGTGAACCATCGAGTCTTTGAACGCAAGTTGCGCCTGATGCCATTAG

GTTGAGGGCACGTCTGCCTGGGCGTCACATATCGAAGCCTCTTGCCAATTTCCTATTGATTGGTATTGTGCAAGATGATG

TTGGCCTCCCGTGAGCACCATCGCCTCATGGTTGGTTGAAAATCGAGACCTTGGTAGAGTGTGCCATGATAAATGGTGCA

TGTGTTAAGCACGAGACCAAACAATCATGTGCTGCTCTATTGAATTTAGCCTCTTTTACCCACATGCGTGTCTAAACGCT

CGTGATGAGACCTCAGGTCAGGCGGGCTACCCGCTGAATTTAA

>OTU_1085

GAAATGCGATAAGTAATGTGAATTGCAGAATTCAGTGAATCATCGAATCTTTGAACGCACATTGCGCCCCTTGGTATTCC

GGAGGGCATGCCTGTTCGAGCGTCATTACACCACTCAAGCTATGCTTGGTATTGGGCGTCGTCCTTAGTTGGGCgcgcCT

TAAAGACCTCGGCGAGGCCAGCTCCGGCTTTAGGCGCTAGTAGAATTTTATTCGGAACGTCTGTACAAAGGGAGAGGGAA

CTCTGCCGACTAGAAACCTTTATTtttCGTAGGTTGACCTCGGATCAGGTAGGGATACCCGCTGAACTTAA

>OTU_1086

GAAATGCGATAAGTAATGTGAATTGCAGAATTCAGTGAATCATCGAATCTTATGAACGCACATTGCGCCcccTGGTATTC

CGGgggCATGGCCTGTTCGAGCGTCATTTCACCACTCAAAGCCTCGCTTGGTTATTGGGCAAGCGCGGTCCGCCGCGTGC

CTCAAATCGACCGGCTGGGTCTTCTGTTCCCTAAGCGTTGTGGAAACTATTCGCTAAAGGGTGCTCGGGAGGCTACGCCG

TTAAAACAAACCCATTTCTAAGGTTTGACCTCGGATCAGGTAGGGATACCCGCTGAACTTAA

>OTU_1087

GAAATGCGATAAGTAATGTGAATTGCAGAATTCAGTGAATCATCGAATCTTTGAACGCACCTTGCGCTCCTTGGTATTCC

TTGGAGCATGCCTGTTTGAGTATCATGAAATTATCAAAATAATTCTTTTGTTCATTCGATTGAATTTATTTTGGACTTGG

AGGTCTGCAGATTCACGTCTGCTCCTCTTAAATAAATTAGCTGGATCTCAGTGAGCTCGGTTCCACTCGGCGTGATAAGT

ATCACTCGCTGAGGACACTGTAAAAGGTGGCCGGGATTGCGGATGAACCGCTTCTAATCGTCCATTCGCTTGGACAACAA

TACTTTATGATCTGATCTCAAATCAGGTAGGACTACCCGCTGAACTTAA

>OTU_1088

GAAATGCGATAAGTAATGTGAATTGCAGAATTCAGTGAATCATCGAATCTTTGAACGCACCTTGCGCCTTTTGGTATTCC

GAAAGGCATGCCTGTTTCAGTGTCATGAAATCTCAATCTAATATGTTTTCTGAACATGTTAGGCTTGGACTTGGGTGTCT

GCCAGCAATGGCTCACCTCAAATGACTTAGTGGAACATCCCACATCAGTGTTAGACGTAATAAGTTTCGTCtctcCTTGT

GGTGATGACTGCTCAAAACCTGCCATCGCTGCACCTTTTGACTTTGACCGTGCAATTACGTAGGGCCTAACCGCTGACTT

A

>OTU_1089

GAAATGCGATAAGTAATGTGAATTGCAGAATTCAGTGAATCATCGAATCTTTGAACGCACATTGCGCCCCTTGGTATTCC

GAGGGGCATGCCTGTTCGAGCGTCATTACACCACTCAAGCTATGCTTGGTGATTGGGCGTCGTCCTTAGTTGGGCgcgcC

TTAAAGACCTTACGGACGTAGGCCACTCCGGCTTTAGGCGTTAGTAGAATTTATTCGAACGTCTGTCAAAGGAGAGGAAC

TCTGCCGACTGAAACCTTTATTtttCTTAGGTTGACCTTCGGATCAGGTAGGGATACCCGCTGAACTTAA

>OTU_1090

GAAATGCGATAAGTAATGTGAATTGCAGAATTCAGTGAATCATCGAATCTTTGAACGCACATTGCGCCCCTTGGTATTCC

GGgggCATGCCTGTTCGAGCGTCATTTCAACCTCAAGCTTAGCTTGGTATTGGAGTCTATGTCAGTAATGGCAGGCTCTA

AATCAGTGGCGGCGCCGCTGGTGCCTGAACGTAGTAATATCtctcGTTACAGGTTCTCGGTGTGCTTCTGCCAAAACCCA

AATTtttCTATTGGTGACCTTCGGATCAGGTAGGGATACCCGCTGAACTTAA

>OTU_1091

GAAATGCGATAAGTAGTGTGAATTACAGAATTTAGTAAATTATTAAATCTTTAAACGCACATTGCGCTCCTTAGTATTCC

GTAGGGCATGCCTGTTCGAGCGTTATTTACCCCTTAAGCTCTGCTTAGTATTAGGCGTTATCCCGCTTTGCGTAAGGACT

TGCTCTAAAGGTATTAGCAGCGGTCGTGCTACCCCTCGCGCAGTATATTACGCTTCTTAAGGCGGTTAATTAGCGTCCAT

AAAGCTTACATTATATCTTAACCTTAGATTAGGTAGGGATACCCGCTAAACTTAA

>OTU_1092

GAAATGCGATAAGTAATGTGAATTGCAGAATTCAGTGAATCATCGAATCTTTGAACGCACATTGCGCCCACCAGTACTCT

GGTGGGCATGCCTGTCCGAGCGTCATTTCAACCCTCAGGGCCCGTTCGCGGGACCTGGTGTTGGGGATCGGCCCCACCGG

CCGGCCCCGCAAATACAGTGGCGGCACACCCGCGACCTCCTCTGCGTAGTAGCAATGCCTCGCAGCTGGATAGCGGTTGC

GCCTCGCCGTAAAACCccccACTTCTCCAAAAGGCTTGATCCTCGGGTACCAGG

>OTU_1093

GAAATGCGATAAGTAATGTGAATTGCAGAATTCAGTGAATCATCGAGTCTTTGAACGCACATTGCGCCcccTGGTATTCC

GGggggCATGCCTGTCCGAGCGTCATTGCTGCCCTCAAGCCCGGCTTGtgtgtTGGGCGCGTCCccccGGGGACGGGCCC

GAAAGGCAGCGGCGGCACCGCGTCCGGTCCTCGAGCGTATGGGGCTCTGTCACCCGCTCTGCAGGCCCGGCCGGCGCCAG

CCGACCCCATCAACCCTTCTTttttCAGGTTGACCTCGGATCAGGTAGGGATACCCGCTGAACTTAA

>OTU_1094

AAAATGCGATAAGTAGTGTAAATTGCAGAATTCAGTGAATTATTAAATCTTTAAACGCATATTGCGCTCCTCGGTATTCC

GTAGGGCATGCCTGTTCAAGCGTTATTTACCCCTTAAGCTCTGCTTAGTGTTAGGCGTTATTCCGCTTCGCGCAAGAACT

CGCCCTAAAGGTATTAGCAGCGGTCGTGCCATCCCTCGCGCAGCATATTACGCTTCTTAAGGCAGTCAATTAGCGTCTAC

AAAGCTTACATTATATCTTAACCTTAGATTAGGTAGGAATACCCGCTAAACTTAA

>OTU_1095

CGACCGGTGGGAAATTCGACGGGATTGTCGGTGTTCTGGGTGGTCTAGCGGTTCTGCGGACGCTTCATGAAACGGGACGG

GAGACCCGTCATCCGATCGAACTGATCAACTGGACGAACGAGGAAGGATCGCGTTTTGCGCCCGCCATGCTGTCCTCAGG

TgtgtTTGCGGGTGTTtttACGGAGCAGGACGCGCTTGATACGAAGGATCGGGAAGGCGTGCGTTTCGGGGATGCGCTTG

AGGGCATCGGGTACCGCGGTGTGGAGGTCTGTGGCACGCATCCTGTGGCGGCGTATTTTGAACT

>OTU_1096

GAAATGCGATAAGTAATGTGAATTGCAGAATTCCGTGAATCATCGAATCTTTGAACGCATATTGCGCCCTCTGGTATTCC

GGAGGGCATGCCTGTCCGAGCGTCATTACTATCTTCAAGCACGGCTTGTTTGTTGAGCCAATTGTTCTTTAATGGGACAG

GCCTGAAATGCAGTGGCGATGCCGAGTTTAGGTGCCCGAGTGTATGGGGCTTTGTCACACGCTTtttGTGGCCCAGCCGG

CATTCTGTCGACCTTGTGATTTACCTAAGATTTAACCTCGGATCAGGTAGGAATACCCGCTGAACTTAA

>OTU_1097

GAAATGCGATACGTAGTGTGAATTGCAGAATTCAGTGAATCATCGAATCTTTGAACGCACATTGCGCCCTTTGGTATTCC

AAAGGGCATGCCTGTTCGAGCGTCATTTGTACCCTAAGCTTTGCTTGGTGTTGGGCGTTCTTGTCTCCCTCTTTGTAGGG

AGACTCGCCTCAAAACGATTGGCAGCCGGCCTACTGGTTTCGGAGCGCAGCACATTttttGCGCTTTGTATCAGGAGAAA

AGGACGGTACTCCATCAAGACTGTTACATTTTCAACTTTTGACCTCGGATCAGGTAGGGATACCCGCTGAACTTAA

>OTU_1098

CGCCGCGTCACCATCGAACTGAAGAAACCGACCCGCGACGGCGAGACGGAAATCCACCTCTTGACGAACCTTCCCGCCAA

GGCTGCCAATGCCCGCACCGTCGCCGACCTCTATCTCCGTCGCTGGACAGTGGAAAAGGCATTTCACGAGTTGGACCAGG

CGTTGCACGGCGAGATCAAGACCTTGGGTTATCCcccAGCGGCATTGTTGAGCTTCTGCGTAGCGTTGCTGGCCTACAAC

GTGATCAGCGTGGTAAAGACTGGTTTGACCGCAGTCCCTGGAGCGAAGGTAAAACGTGAAGACCTctctGGCTATTATCT

G

>OTU_1099

GAATTGCGATAAGTAATGTGAATTGCAGATACTCGTGAATCATTGAATTttttGAACGCACATTGCGCCCTTGAGCATTC

TCAGGGGCATGCCTGTTTGAGCGTCATTTCCTTCTCAAAAGATAATTTATTATTtttttGGTTGTGGGCGATACTCAGGG

TTAGCTTGAAATTGGAGACTGTTTCAGTCTTtttttAATTCAACACTTAGCTTCTTTGGAGACGCTGTTCTCGCTGTGAT

GTATTTTATGGATTTTATTCGTTtttACTTTTACAAAGGGGAAAATGGTAACGTACCTTTAGGCAAAAGGGGTTGCTTtt

tAATATTCATCAAGTTTTGACCTCAAATCAGGTAGGATTACCCGCTGAA

>OTU_1100

GAAATGCGATAAGTAATGTGAATTGCAGAATTCAGTGAATCATCGAGTCTTTGAACGCACATTGCGCCcccTGGTATTCC

GGggggCATGCCTGTCCGAGCGTCATTGCACCCCTCAAGCCCGGCTTGTCCTTGGTTATGTCCTCCGTTCCGGAGGGAGC

AGGCCGTCCAAATGACAATGGCGGCGAGCCGCGTCCGGTCCTCGAGCGTATGGgggCTTTTGTCACCCGCTTTGTAGGCC

GGCCGGTCGCTTGCCCTTCAAGCACAACTTCTTATGTTGACCTCGGATCAGGTAGGGATACCCGCTGAACTTAA

>OTU_1101

GAAATGCGATAAGTAGTGTGAATTGCAGAATTCAGTGAATCATCGAATCTTTGAACGCACATTGCACTCCTTGGTATTCC

ATGGAGTATGCCTGTTCGAGCGTCATTTGTACCCTCAAGCCTTGCTTGGTGTTGGGCGTTTGTCTTGCGAGACTCGCCTT

AAAACGATTGGCAGCCGGCCTACTGGTTTCGGAGTGCAGCACAAATTGCGCTCTGTCCACGCAGGTTGGCGTCCACAAAG

CCcccATATTCCACTTTTGACCTCGGATCAGGTAGGGATACCCGCTGAACTTAA

>OTU_1102

GAAATGCGATAAGTAATGTGAATTGCAGAATTCAGTGAATCATCGAATCTTTGAACGCACCTTGCGCTCCTTGGTATTCC

TTGGAGCATGCCTGTTTGAGTATCATGAAATCCTCAAAACAAGTCTTTCGTTCATTCGGCTGACTTTTGTTTTGGACTTG

GAGGTCTTGCAGGTTCACGTCTGCTCCTCTCAAAAGCAATTAGCTGGATCTCGGAGCTCGGTTCCACTCGGCGTGATAAA

TATCACTCGCTGAGGACACCCAGGTGGCCGGGTTTGCGGATTGAGCCGCTTCTAACCGTCCATTTGCTTGGACAACAACA

CTTTATGATCTGATCTCAAATCAGGTAGGACTACCCGCTGAACTTAA

>OTU_1103

GAAATGCGATAACTAATGTGAATTGCAGAATTCAGTGAATCATCGAGTCTTTGAACGCACATTGCGCCccccTGGTATTC

CGGggggCATGCCTGTCCGAGCGTCATTGCTGCCCTCAAGCCCGGCTTGtgtgtTGGGTCGCGTCCcccTCTCCGGgggg

ACGGGCCCGAAAGGCCAGCGGCGGCACCGCGTCCGATCCTCGAGCGTATGGGGCTTTGTCACATGCTCTGTAGGATTGGC

CGGCGCTGCCGACGTTTCCAACCATTttttCCAGGTTGACCTCGGATCAGGTAGGGATACCCGCTGAACTTAA

>OTU_1104

GAAATGCGATAAGTAATGTGAATTGCAGAATTCAGCGAATCATCGAATCTTTGAACGCACATTGCGCCCATTAGTATTCT

AGTGGGCATGCCTGTTCGAGCGTCATTTCAACCCTCGGGCCCGTATGGGACCGGCGTTGGGGCCTTtttttGCCGTATGG

CAAGCCCCGAAAGAAAGTGGCGGGCCTGCTGAGACCCCGAGCGTAGTAATTTACATCGCTCCGGGGCGCCCGGTGGGCGG

CACCAGCCGTTAAAGCACTTCAAGTCTTACAAGGTTGACCTCGGATCAGGTAGGAATACCCGCTGAACTTAA

>OTU_1105

GAAAGGCGATAAGTAATGTGAATTGCAGAATTCAGTGAATCATCGAATCTTTGAACGCACATTGCACCCTCTGGTATTCC

AAGGGGTATGCCTGTTCGAGCGTCATTACAACCCTCAAGCACTGCTTGGTTTTGGATGTTACCATTGTGGTGCATCTCAA

AAGTATTAGCAGTAGCATTCAGCTTCTAGTGTAGTAAATTTCTCGCTTTGGGGCTAAGTGTCTAATTGTTAGAACTCCTA

ATTTATCAAAGGTTGACCTCGGATCAGGTAGGGATACCCGCTGAACTTAA

>OTU_1106

AAAGTGCGAAAAGTGTTGCGATTTGCACGAATCTGTGAGTCATCTAATTtttGAACGCGAATGGCACTGTTACGCAAGTA

GCAGTATGTCTGTTTGAGAATCGCAAAACAAGATCAACTCTAGTTGAGAGTTGGGATTGGCCTCATGTACCTTTGGGTGT

ATTTGGCTTGAATGCATCGAGTTGATGAGCTCCcccAATGCGGCTCAATGtgtgCTAGCGAGACAGTCGTCCCGATCTCA

CATTTCCGCCGCGTTGCAGAGTTCTACGTACAACCCGCCAACATACGTATATTTATCAAATACGATCTCAAATCAGGCAA

GATTACCCGCTGAACTTAA

>OTU_1107

GAAATGCGATAAGTAATGTGAATTGCAGAATTCAGTGAATCATCGAATCTTTGAACGCACATTGCGCCCCTTGGTATTCC

GAGGGGCATGCCCGTTCGAGCGTCATTATAACCCCTCAAGCCTAGCTTGGTGTTGGGGCCTGCTGTTACCAGCAGCCCTT

AAAATCAGTGGCGGTGCCATCTGGCTCTAAGCGTAGTAATACTTCTCGCTACAGGGTCCCGGTGGAGCTTGCCATCAACC

CCTAATTTTCTATGGTTGACCTCGGATCGGGTAGGGATACCCGCTGAACTTAA

>OTU_1108

GAAATGCGATAAGTAATGCGAATTGCAGAATTTCCGTGAGTCATCGAATCTTTGAACGCACATTGCGCCCATTGGTATTC

CGATGGGCATGCCTGTTCGAGCGTCATTATCCTCCCTCAAACCTCGTTTGGTGTTGGACCGCGTTGGCCGAGCGACCAAC

TGGTCTCAAAGACAATGACGGCGTCCGTGGGGCCTCGGTGCAACGAGCTTCTAAGGAGCgcgcgTCGAGTTTCAAGGACC

CTCCGGGCCGGTCTTGACCTCTATCTTCTCAGGTTGACCTCGGATCAGGTAGGAATACCCGCTGAACTTAA

>OTU_1109

GAAATGCGATAAGTAATGTGAATTGCAGAATTCAGTGAATCATCGAATCTTTGAACGCACATTGCGCCCACCAGTACTCT

GGTGGGCATGCCTGTCCGAGCGTCATTTCAACCCTCAGGGCCCGTTCGCGGGACCTGGTGTTGGGGATCGGCCCCACCGC

CGGCCCCGCAAATACAGTGGCGGCACACCCGCGACCTCCTCTGCGTAGTAGCAATGCCTCGCAGCTGGATAGCGGTTGCG

CCTCGCCGTAAAACCccccACTTCTCAAAAGGCTTGACCTCGGACAGGTAGGATACCCGCTGGAACTTAAC

>OTU_1110

GAAATGCGATAAGTAGTGTGAATTGCAGACTTTAGTGAATCATCGAATCTTTGAACGCACATTGCGCCCCTTGGTATTCC

ATTGGGCATGCCTGTTCGAGCGTCATTTAAACCCTCAAGCTCACGCTTGGTGTTGGGCGTCTGTCCGCGCTTTCGGCGCA

GACTCGCCCCAAATCCATTGGCAGCGGCTTCGCCGGCTTCTCGCGCAGCACATTGCGTTCTTGAAGACTTTCGGCGGGCT

TGCATCCATCAAGTAACCTCTTATTTTGACCTCGAATCAGGTAGGGATACCCGCTGAACTTAA

>OTU_1111

GAAATGCGATAAGTAGTGTGAATTGCAGAATTCAGTGAATCATCGAATCTTTGAACGCACATTGCGCCCTTTGGTATTCC

AAAGGGCATGCCTGTTCGAGCGTCATTTGTACCCCAGCATTGCTTGGCTGTTGGCTGGCTTCTGTCTCTAGCTTTGCTGG

AGACTCGCCTTAAAGTAATTGGCAGCCGGCCTACTGGTTTCGGAGCGCAGCACAAGTCGCACTctctATCAGCAAAGGTC

TAGCATCCATTAAGCCTTtttttCAACTTTTGACCTCGGATCAGGTAGGGATACCCGCTGAACTTAA

>OTU_1112

GAAATGCGATACGTAATGTGAATTGCAGAATTCAGTGAATCATCGAATCTTTGAACGCACATTGCGCCCTCTGGTATTCC

GGggggCATGCCTGTTCGAGCGTCATTATAACCACTCAAGCTCTCGCTTGGTATTGGGGTTCGCGGTTTCGCGGCCCCTA

AAATCAGTGGCGGTGCCTGTCGGCTCTACGCGTAGTAATACTCCTCGCGATTGAGTCCCGGTGGGTGCTTGCCATCAACC

TTAAAACTTtttCTATGGTTGACCTCGGATCAGGTAGGGATACCCGCTGAACTTAA

>OTU_1113

GAAATGCGATAAGTAATGTGAATTGCAGAGTTCAGTGAATCATCGAATCTTTGAACGCACATTGCGCCCTCTGGTATTCC

GGggggCATGCCTGTTCGAGCGTCATTACAACCCTCAAGCTCTGCTTGGTATTAAGCTTCACCTGAAAAGGCGGGCTCTA

AAATCAGTGGCGGTGCCATTCGGCTTCAAGCGTAGTAATTtttCTCGCTTTGGATGACCGGTTGCGTGCCTGCCATAACC

CAAATTTTATCAAAGGTTGACCTCGGATCAGGTAGGGATACCCGCTGAACTTAA

>OTU_1114

GAAATGCGATAACTAATGTGAATTGCAGGCATCGTGAATCATCGAGTTCTTGAACGCACATTGCGCCCTGTGGTATTCCG

CAGGGCATGCCTGTTTGAGCGTCATTTCACCACTCAAGCCTCGCTTGGTATTGGGCAACGCGGTCCGCCGCGTGCCTCAA

ATCGTCCGGCTGGGTCTTCTGTCCCCTAAGCGTTGTGGAAACTATTCGCTAAAGGGTGCTCGGGAGGCTACGCCGTAAAA

CAAACCCATTTCTAAGGTTGACCTCGGATCAGGTAGGGATACCCGCTGAACTTAA

>OTU_1115

GAATTGCGATAAGTAATGTGAATTGCAGATTCTCGTGAATCATTGAATTtttGAACGCACATTGCGCCCTCTGGTATTCC

AGAGGGCATGCCTGTTTGAGCGTCATTTCCTTCTCAAAACCCAGTTtttGGTTGTGAGTTGATACTCTGTTACAGGGTTA

ACTTGAAAATGCTATGCCCATTTGGCTGCCCCTTCTCTGAGGGGACTGCGCGTCTGTGCAGGATGTAACCAATGTATTTA

GGTATTCATACCAACTTTCATTGTGCGCGTCTTATGCAGTTGCAGTCCACCCAACCTCGGACacacTGGGCTGGCTGGGC

CAACAGTATTCATAAAGTTTGACCTCAAATCAGGTAGGAGTACCCGCTGAACTTA

>OTU_1116

GAAATGTGATAAGTAATGTGAATTGCAGAATTCAGTGAATCATCGAATCTTTGAACGCACCTTGCGCCTTTTGGTATTCC

AAAAGGCACACCTGTTTGAGTGTCATGAAACCCTCTCATTAAATAATTTTGATTAATTATTTTCAATGGATGTTGAGTGC

TGCTGTAATTAGCTCACTTTAAATatataAGTCACTTTTCTATAAGTTGGATTGACTTGGTGTAATAATTTTATCATCAC

ATCAAGGATTGTAGCAATACTGCCATCTTATTTAAGGGAGACTCCTAAaaaCCCAATTTTAACCTTAAGACCTCAAATCA

GGTGGGACTACCCGCTGAACTTAA

>OTU_1117

GAAATGCGATAAGTAATGTGAATTGCAGAATTCAGTGAATCATCGAATCTTTGAACGCACATTGCGCCccccTGGTATTC

CGGgggggCATGCCTGTTCGAGCGTCATTTCACCACTCAAGCCTCGCTTGGTATTGGGCAACGCGGTCCGCCGCGTGCCT

CAATCGTCCGGCTGGGTCTTCTGTCCCCTAAGCGTTGTGGAACTATTCGCTAAAGGGTGTTCGGGAGCTACGCCGTAAAC

ACCCATTCTAGGTTGACCTCGGATCAGGTAGGGATACCCGCTGAACT

>OTU_1118

GAAACGCGATAAGTAATGTGAATTGCAGAATTTAGTGAATCATCGAACTTTTGAACGCACATTGCGCCCGGCGGCACTCC

GCCGGGCATGCCTGTCCGAGCGTCATTATACTGCCCTCGACTCTCCGAGTCGGTGTTGGGAGCCTCTGCGCCTGTACGCA

GCTCCCCAAGTCCACTGGCGTCGCGCCACGGTGCCcccTGCGTAGTAATTTCACCTCGCTTGGGACCCTGGGCGCCGGCT

GCCTTCCCTCTCGCAGCAGGCTCTTCGAGTCGGCTCCAGCACAGTTGACCTCGGATCAGGTAGGGTTACCCGCTGAACTT

AA

>OTU_1119

GAAATGCGATAAGTAATGTGAATTGCAGAATTCAGTGAATCATCGAATCTTTGAACGCACATTGCGCCCCTTGGTATTCC

GGggggCATGCCTGTTCGAGCGTCATTTCAACCCTCAAGCTTAGCTTGGTATTGAGTCTATGTCAGTAATGGCAGGCTCT

AAAATCAGTGGCGGCGCCGCTGGGTCCTGAACGTAGTAATATCtctcGTTTACAGGGTTCTCGGTGTGCTTCTGCCAAAA

CCCAAATTttttCTATGGTTGACCTCGGATCAGGTAGGGATACCCGCTGAACTTAA

>OTU_1120

GAAATGCGATAAGTAATGTGAATTGCAGAATTCAGTGAATCATCGAATCTTTGAACGCACATTGCGCCCGCCAGTATTCT

GGCGGGCATGCCTGTCCGAGCGTCATTTCAACCCTCGGGCCccccccTTTTCCCCTGCGGgggAGGGGCGGGCCCGCGTT

GGGGCCCAGGCGTCCTCAAGGGCGCCTGTCCCCGAAACCCAGTGGCGGCCTCGCCGCTGCCTCCTCCGCGTAGTAGCACA

AACCTCGCGGGCGGAAGGCgcgcgGCCACGCCGTAAAACCCCAACTTTTACCAAGGTTGACCTCGGATCAGGTAGGAATA

CCCGCTGAACTTAA

>OTU_1121

GAAATGCGATAAGTAGTGTGAATTGTAGAATTTAGTGAATTATTGAATCTTTGAACGCACATTGCGCCCCTTGGTATTCT

ATAGGGCATGCCTGTTTGAGCGTTATTTGTACCCTTAAGCTCTGCTTGGTGTTAGGTGTTTGTCTCCTCTAGGAGACTCG

CCTTAAAATAATTGGCAGCTAGTATATTGGTATTGGAGCGCAGCACAAGTCGCGCTTCTGTCTATATTTGTTAGCATCTA

GCAAGACTATTtttACTCTTAACCTTAGATTAGGTAGGGATACCCGCTAAACTTAA

>OTU_1122

GAAATGCAATAAGTAATATAAATTGCAGAATTTAGTGAATTATTAAATCTTTGAATGCATATTGCGCCCGCCAGCATTCT

AGCAGGCATACCTATTTAAGCGTTATTTCAACCCTCAAGCTCTACTTAGTGTTGAGGCTCTACAGTTGATATAGGCTCTT

AAAGGTAGTAGTAGACCCTCCTAAAGCCTCCTTTGCGTAGTAACTTTACGTCTCACACTAGGATCTAGAGGGACTTTTGC

TGTAAAACCCCTTAATTTTCTAAAGGTTAACCTTAGATCAAGTAGGAATACCCGCTGAACTTAA

>OTU_1123

GAAATGCGATAAGTAGTGTAAATTGCAGAATTTAGTAAATTATTAAATCTTTAAACGTATATTACGCCCCTTGGTATTCC

GTAGGGCATGCCTGTTTAAGCGTTATTTACCCCTTAAGCTCTACTTAGTGTTAGGCGTTATCCCGCTTCGcgcgAGGACT

TGCTCTAAAGGTATTAGTAGCGGTCGTACCACCCCTTACGCAGTATATTACGCTTCTTAAGGCGGTTAATTAGCGTCTAT

AAAGCTTATATTATATCTTAACCTTAGATTAGGTAGAGATACCCGCTAAACTTAA

>OTU_1124

GAAATGCGATAAGTAATGTGAATTGCAGAATTCCGTGAATCATTGAATCTTTGAACGCACATTGCGCCcccTGGCATTCC

GGggggCATGCCTGTCCGAGCGTCATTGCTAACCCTCAAGCTCGGCTTGtgtgtTGGGCCTCGTCCcccTCGGGGACGGG

CCTGAAAGGCAGCGGCGGCGTCGCGTCCGGTCCTCGAGCGTATGGGGCTCTGTCACCCGCTCAGGAGGCCGGCCGGGGCT

CGCCATCAACCCACATCATAACAAGGTTGACCTCGGATCAGGTAGGGATACCCGCTGAACTTAA

>OTU_1125

GAAATGCGATAAGTAATGTGAATTGCAGAATTCAGTGAATCATCGAATCTTTGAACGCACATTGCGCCCGCCAGTATTCT

GGCGGGCATGCCTGTCCGAGCGTCATTTCAACCCTCGAACCCCTCCGGgggTCGGGCGTTGGGGATCGGCCCTGCCTTGG

CGGTGGCCGTCTCCGAAATACAGTGGCGGTCTCGCCGCAGCCTCTCCTGCGCAGTAGTTTGCACACTCGCATCGGGAGCG

CGGCGCGTCCACAGCCGTTAAACACCCAACTTCTGAAATGTTGACCTCGGATCAGGTAGGAATACCCGCTGAACTTAA

>OTU_1126

GAAATGCGATACGTAATGTGAATTGCAGAATTTCTGAATCATCGAATCTTTGAACGCACCTTGCACTCTGTGGTTATTCC

GCAGAGTATGTTTGTTTGAGTGTCGCGATACTTCTCAACCCCGCTTCATTAACTTGAAGTAGTTTGGGCTTGGATCATGG

TCGTTGCCGGCTTAAGGCCAGGCTCGATTGAAATACAACAAGCTGGATGATGCCATTAGCGGCTTGAACTTAACGTTGTA

ATTACTTATTCGTTAGGCTACGCTTTTGGAGTGTTCAATGAGCTTACCAATTACTTAAGAACTTTATTGTTCAATTTGAC

CTCAAATCAAGCAGGACTACCCGCTGAACTTAA

>OTU_1127

GAAATGCGATAAGTAGTATAAATTACAGAATTCAGTAAATTATTAAATCTTTAAACGCATATTGCGCCCCTTAGTATTCC

GTAGGGCATGCCTGTTTAAGTGTTATTTACCCCTTAAGCTCTGCTTAGTGTTAGGCGTTATCCCGCTTTGCGCGAGGACT

CGCTCTAAAGGTATTAGCAGCGGTTGTGCTACCCCTTACGCAGCACATTACGCTTCTTAAGGCGGTTAATTAGCGTTTAT

AAAGCTTACATTATATCTTAACCTTAGATTAGGTAGGGATACCCGCTAAACTTAA

>OTU_1128

GAAATGCAATAAGTAATGTGAATTGCAGAATTCAGTAAATCATCAAATCTTTGAACGCACATTGCGCCCACCAGCATTCT

AGCAGGCATGCCTATTCAAGCGTCATTTCAACCTCAAGCTCCACTTAGTGTTAGGGCCCTACAGCTGATATAGGCCCTCA

AAAGTAGTGGCAAACCCTCCTGAAGCCTCCTTTGCGTAGTAACTTTACGTCTCACACTAAGATCCAGAGGGACTCTTGCC

GTAAAACCccccAATTTTCCAAAGGTTAACCTCAGATCAAGTAGGAATACCCACTAAACTTAA

>OTU_1129

GAAATGCGATAAGTAATGTGAATTGCAGAATTCAGTGAATCATCGAATCTTTGAACGCACCTGGCGCTCCCTGGTATTCC

GGGGAGCATGTCTGTTTGAGTGTCATTAAATTCTCAACCTTTCCTGCTTTTGCGAGCTTGGTCAGGCTTGGATGTGGggg

TTGCGGGCTTCTCAGAAGTCGGCTCTCCTTAAATGCATTAGTGAAACCTTTGTTGATCATCTCTTGGTGTGATAATTATC

TACGCCATTGGAGTGTCAGCCTCAACTCTGTTGAGAGGTTCTGCTTCTAACTGTCCCTTTAGGGACAACTTCTGACCTTT

TGACCTCAAATCAGGCAAGACTACCCGCTGAACTTAA

>OTU_1130

GAAATGCGATAAGTAATGTGAATTGCAGAATTCAGTGAATCATCGAATCTTTGAACGCACCTTGCGCCTTCTGGTATTCC

GGAAGGCATGCCTGTTTGAGTGTCATGAACACCTCACCcccTCGGGTTTCCGATCGGGTGGACTTGGGCGCCTGCCATTC

ACTTGGCTCGCCTTAAATGCATTAGCGGTCGTTGACGTAATAAGTTTCGTCGGTCTGGCCGCTGACAATCTCCCTTCACT

GGGATGTCTCGCGAGGGACACTTTCATGATCTGACCTCAAATCAGGTAGGACTACCCGCTGAACTTAA

>OTU_1131

GAAATGCGATAAGTAATGTGAATTGCAGAATTCAGTGAATCATCGAATCTTTGAACGCACCTTGCGCCCTCTGGTATTCC

GGAGGGCATGCCTGTTTGAGTGTCATGTAGACTCAACCccccTGGTTTATGATCGGGAAGGGTTGGATGTGGGCGCTGCC

GATCCCCGGCTCGCCTTAAATGTCTTAGCGGCTCAGAAGCCCCGACCTAGCGTAATAAGTTTCGCTGGAGAGGGTGTGGA

TGACTGCTTACAATCGCCTCGGCATCTTTGACTCTGGCCTCAAATCAGGTAGGACTACCCGCTGAACTTA

>OTU_1132

GAAATGCGATAAGTAATGTGAATTGCAGAATTCAGTGAATCATCGAATCTTTGAACGCACATTGCGCCCCTTGGTATTCC

GAGGGGCATGCCTGTTCGAGCGTCATTACACCACTCAAGCTATGCTTGGTATTGGCGCTCGTCCGTTACGTTGGAGCTGG

CgcgcTTAAAAGACCTCGGCGAGGCCACTCCGGCTTTAGGCGTAGTAGAATTTATTCGAACGTCTGTCAAAGGAGAGGAA

CTCTGCCGACTGAAACCTTTATTtttCTAGGTTGACCTCGGATCAGGTAGGGATACCCGCTGAACTTAA

>OTU_1133

GAATTGCGATAAGTAATGTGAATTGCAGATACTCGTGAATCATTGAATTTTGAACGCACATTGCGCCCTTGAGCATTCTC

AAGGGCATGCCTGTTTGAGCGTCATTTCCTTCTCAAAAGATAATTttttATTttttGGTTGTGGGCGATACTCAGGGTTA

GCTTGAAATTGGAGACTGTTTCAGTCTTttttAATTCAACACTTAGCTTCTTTGGAGACGCTGTTCTCGCTGTGATGTAT

TTATGGATTTATTCGTTTTACTTACAGCAAGGAATGGTAATGTACCTTAGGCAAAGGGTTGCTTTTAATATTCATCAAGT

TTGACCTCAAATCAGGTAGGATTACCCGCTGAACTTAA

>OTU_1134

GAAATGCGATAAGTAATGTGAATTGCAGAATTCAGTGAATCATCGAATCTTTGAACGCACATTGCGCCcccTGGTATTCC

GGgggAGCATGCCTGTTCGAGCGTCATTTCACCACTCAAGCCTCGCTTGGTGATTGGGCATCGCGGTCCGCCTGCGTGCC

TCAAATACGACCGGCTGGGTCTTCTGTCCCCTAAGCGTTGTGGAAACTATTCGCTAAAGGGTGTTCGGGAGGCTACGACC

GTAAACAACCCCATTTCTAAGGTTTGACCTCGGATCAGGTAGGGATACCCGCTGAACTTAA

>OTU_1135

GAAATGCGATAAGTAGTGGTAGTAATTGCAAGAATTCAGTAGAATCATCGAATCTTTGAACGCACATTGCGCCCTTTGGT

ATTCCAAAGGGCATGCCTGTTCGAGCGTCATTTGTACCCTCAAGCTTTGCTTGGTGTTGGGCGTCTTGTCTCTAGCTTTG

CTGGAGACTCGCCTTAAAGTAATTGGCAGCCGGCCTACTGGTTTCGGAGCGCAGCACAAGTCGCACTctctATCAGCAAA

GGTCTAGCATCCATTAAGCCTTtttttCAACTTTTGACCTCGGATCAGGTAGGGATACCCGCTGAACTTAA

>OTU_1136

GAAATGCGATAAGTAATGTGAATTGCAGAATTCCAGTGAATCATCGAAACTTTGAACGCACATTGCGCCCGCCAGTATTC

TGGCGGGCATGCCTGTTCGAGCGTCATTTCAACCATCAAGCCCCAGGCTTGCGTTGGgggCCTGCGGCTGCCGCAGCCcc

cTAAAAGCAGTGGCGGGCTCGCTGTCATTCCGAGCGCAGTAGTTACATCTCGCTCCGGGCGTGGCGGCGGGCACCTGCCG

TGAAACACTCCTTCTAAGGTTGACCTCGGATCAGGTAGGAATACCCGCTGAACTTAA

>OTU_1137

GAAATGCGATAAGTAATGTGAATTGCAGATACAGTGAATCATCGAATCTTTGAACGCAAATGGCACTCTATGGTATTCCG

TAGAGTACGTCTGTTTGAGCGTCGCGAACATCTCCACAATTAGTTtttttAATTAGTTGAGGGTTTTGAGGTTGTCATAT

AAACAGTGACTCCCTTTAAAATAATTAGTGATGACCTTATGAATGGGTTAATACTGtgtgtTATAATGGATTACATCCAT

CACCAGTCAGagagTAATCTCGCCTTAGTAATTTGTAGTGATTGCTTCTAACTGCCAATTGGCAACAACCTGATCAAATC

GACCTCAAATCAGATGGGATTACCCGCTGAACTTAA

>OTU_1138

GAAATGCGATAAGTAGTGTGAATTGCAGAATTCAGTGAATCATCGAATCTTTGAGCGCACATTGCGCCCTTTGGTATTCC

AAAGGGCATGCCTGTTCGAGCGTCATTTGTACCCTAAGCTTTGCTGGTGTTGGGCTCTTGTCTCTAGCTTTGCGGAGACT

CGCCTTAAGTAATTGGCAGCCGGCCTACTGGTTTCGGAGCGCAGCACAAGTCGCACTctctATCAGCAAAGGTCTAGCAT

CCATTAAGCCTTtttttCAACTTTTGACCTCGGATCAGGTCATGGGATACCCGTCTGAACTTAA

>OTU_1139

GAAATGCGATAAGTAATGTGAATTGCAGAATTCAGTGAATCATCGAATCTTTGAACGCACATTGCGCCCCTTGGTATTCC

GGgggCATGCCTGTTCGAGCGTCATTTCAACCCTCAAGCTTAGCTTGGTATTGAGTCTATGTCAGTGAATGGCAGGCTAC

TAAAATCAGTGGCGGCGCCGCTGGGTCCTGAACTGTAGTAATATCtctcGTTAACAGGTTCGTCGGTGTGCCTTCTGCCA

AaaaCCCAAATTtttCTTATGGTTGACCTCGGATCAGGTAGGGATACCCGCTGAACTTAA

>OTU_1140

AAAATGCGATAAGTAGTATAAATTACAGAATTTAGTAAATTATTAAATCTTTAAACGTATATTGCGCCCCTTAGTATTCC

GTAGGGCATGCCTGTTTAAGCGTTATTTACCCTTTAAGCTCTGCTTAGTGTTAGGCGTTATCCCGCTTCGCGTAAGAACT

CGCTCTAAAGGTATTAGCAGCGGTCGTGCTACCCCTCGCGCAGTATATTACGCTCCTTAAGGCGGTTAATTAGCGTCTAT

AAAGCTTATATTATATCTTAACCTTAGATTAGGTAGGGATACCCGCTAAACTTAA

>OTU_1141

GAAATGCGATAAGTAATGTGAATTGCAGAATTCCGTGAATCATCGAATCTTTGAACGCACATTGCGCCCCTCGGTATTCC

GGggggCATGCCTGTTCGAGCGTCATTACACCACTCAAGCCTCGGCTTGGTCTTGGGCGTCGCGGTCCCCGCGTGCCTCA

ATGTCGCCGGCTGGTGCGTCCGTCTCTAAGCGTTGTGAATCAACAGTCGCTCCGGAGGTCGGACGGAACCGCGCCGTCAA

GCCTTtttCTACAGGTTGACCTCGGATCAGGTAGGGATACCCGCTGAACTTAA

>OTU_1142

GAAATGCGATACTTGGTGTGAATTGCAGAATCCCGTGAACCATCGAGTCTTTGAACGCAAGTTGCGCCCGAAGCCTTCGG

GTTGAGGGCACGCCTGCCTGGGCGTCAcacaTCGCGTCTCCcccAACCCACCGAGAGTGGGGAGGAGGAGGAGGATGGCT

TCCCATGCCTCACCGGGCGTGGATGGCCTAAaaaTGGAGCCcccGGTTACGAGCTGCCGCGGCGATTGGTGGTATACAAG

GCCTTGCCTAGGATGCATCGCGTCGTGCAGCACGTAGCTAGTGAGGTCTCGTAGGACCCTTAGTTGTTGCCAATATTGGG

CGACAAAACCGTTGAGACCCCAGGTCAGGCGGGGCTACCCGCTGAGTTTAA

>OTU_1143

GAAATGCGATAAGTAGTATAAATTATAGAATTTAGTAAATTATCAAATCTTTGAACGTACATTGCGCCCCTTAGTATTCT

ATAGGGCATGCCTGTTTGAGCGTTATTTATACCCTTAAGCTCTACTTAGTGTTAGGTATTTGTCTCCTCTAGGAGACTCG

CCTCAAAACAATTAGCAGCTAGCATATTAGTATTAGAGCGCAGCACAAGTCGCGCTTCTATCTATATTTATTAGCATCTA

GCAAGACTATTttttACTCTTAACCTTAGATTAGGTAGGGATACCCGCTAAACTTAA

>OTU_1144

GAAATGCGATAAGTAATGTGAATTGCAGAATTCAGTGAATCATCGAATCTTTGAACGCACATTGCGCCCGCCAGTATTCT

GGCGGGCATGCCTGTCCGAGCGTCATTTCAACCATCAAGCCCCGGGCTTGTGTTGGGGACCTGCGGCTGTCCGCAGGCCC

TGAAAACCAGTGGCGGGCTCGCTGTCACCCCGAGCGTAGTAGTATTATCTCGCTAAGGGCGTGCTGCGGGTTCCGGCCGT

TAAACAGCCTTCATAACCCAAGGTTGACCTCGGATCAGGTAGGAAGACCCGCTGAACTTAA

>OTU_1145

GAAATGCGATAACTAGTGTGAATTGCAGATTTCCGTGAATCATCGAGTCTTTGAACGCACATTGCGCCCCTTGGTTTACC

AGGgggCATGTCTATCCGAGCGTCGTTACACCCCTCAAGCCTGGCTTGGTCTTGGGGTATGTCCTGCCTAGCAGGGCTGC

CCTCAAATGTAACTCGACTGCCTTTTGTTCGGGAGCGCAGCAGTTTTGCGGCTTTCGCTGGTTCCGTGGAGCCGCCCCTG

GCGGACCTctctGGTTGACCTCGGATTAGATAAGGATACCCGCTGAACTTAA

>OTU_1146

GAAATGCGATAAGTAATGTGAATTGCAGAATTCAGTGAATCATCGAATCTTATGAACGCACATTGCGCCCCTTGGTATTC

CGAGGGGCATGGCCTGTTCGAGCGTCATTACATCCACTCAAAGCTATGCTTGGTTATTGGGCGTCGTCCTTAGTTGGGCg

cgcCCTTTAAAGACCTCGGCGAGGCCACTCCGGCTTTAGGCTGTAGTAGAATTTATTCGAACGTCTGTCAAAGGAGAGGA

ACTCTGCCGACTGAAACCTTTTATTtttCTAGGTTGACCTCGGATCAGGTAGGGATACCCGCTGAACTTA

>OTU_1147

GAAATACGATAAGTAGTGTAAATTATAGAATTTAGTAAATTATCGAATCTTTAAACGCACATTGCGCTCCTCGGTATTCC

GTAGGGCATGCCTATTTGAGCGTTATTTACCCCTTAAGCTCTGCTTAGTATTAAGCGTTATCCCGCTTTGCGCAAGGACT

CGCTCTAAAGGTATTAGCAGCGGTTGTACCACCCCTTACGCAGTATATTACGCTTCTTAAGGCGGTTAATTAGCGTCCAT

AAAGCTTATATTATATCTTAACCTCGGATTAGGTAGGAATACCCGCTAAACTCAA

>OTU_1148

GAAATGCGATACGTAGTGTGAATTGCAGAATTCAGTGAATCATCGAATCTTTGAACGCACATTGCGCCCTTTGGTATTCC

AAAGGGCATGCCTGTTCGAGCGTCATTTGTACCCTCAAGCTTTGCTTGGTGTTGGGCGTTttttGTCCcccccAAaaaGG

GGACTCGCCTTAAAAGGATTGGCAGCCGGCCTACTGGTTTCGCAGCGCAGCACATTtttGCGCTTGCAATCAGCAAAAGA

GGACGGCAATCCATCAAGACTCCTTCTCACGTTTGACCTCGGATCAGGTAGGGATACCCGCTGAACTTAA

>OTU_1149

GAAATGCGATAAGTAATGTGAATTGCAGAATTCAGTGAATCATCGAATCTTTGAACGCACATTGCGCCCGCCAGTATTCT

GGCGGGCATGCCTGTTCGAGCGTCATTTCAACCCTCAAGCCcccGCGGTTTGGTGTTGGgggCCGGCGATTGTCAGCTGG

GCCGCTCAGGCGGTTCCCTGCGGCGCCGCCcccGAAATGAATTGGCGGCCCCGTCGCGGCCTCCTCTGCGTAGTAGCACA

ACCTCGCAACAGGAGCGCGGCGCGGCCACTGCCGTAAAACGCACAAACTTCTCCAAGAGTTGACCTCGAATCAGGTAGGA

ATACCCGCTGAACTTAA

>OTU_1150

GAAATGCGATACGTAGTGTGAATTGCAGAATTCAGTGAATCATCGAATCTTTGAACGCACATTGCGCCCTTTGGTATTCC

AAAGGGCATGCCTGTTCGAGCGTCATTTGTACCCTCAAGCTTTGCTTGGTGTTGGGCGTCTTGTCTCCCCGTTTTGCGGG

AGACTCGCCTTAAAGTCATTGGCAGCCGGCCTACTGGTTTCGGAGCGCAGCACAAGTCGCGCTctctTCCAGCCAAGGTC

CAGCGTCCAGTAAGCCTTtttttCAACCTTTGACCTCGGATCAGGTAGGGATACCCGCTGAACTTAA

>OTU_1151

CAGCTGCGAGACTTGGTGTGAATTGCAGGACACATTGAGCACTGATTtttCGAACGCGCATTGCGGTCTCGGGTACTACC

CGGGACCACGTCTGTCTGAGGGTCGGATAAACGCTTGCAGAAGGAGGTCTCCTCCTGATTGGTCGCTTCGGAAGCAATTC

CGTCGACCTAAGTCTTGCGTAGATCTGTCGCCAGGAGGCGTCCCGCGTAGGACGCCCGGGAGCGGGAGTGACGGAATCGT

AGACTCCcccGAGCCCGGGCCAGCGGTAGATGCGAGGAGCGACGGACGATACGGACAGATCGACGAGAGTCGACTCCGTC

GTTATATGATGTCGACCTCAGATCAGGCGAGACGACCCGCTGAATTTAA

>OTU_1152

GAAATGCGATAAGTAGTGTGAATTGCAGAATTCAGTGAATCATCGAATCTTTGAACGCACATTGCGCCCTTGGTATTCCG

AAGGCATGCCTGTTCGAGCGTCATTTGTACCCTCAAGCTTTGCTTGGTGTTGGGCGTCTTGTCTCTAGCTTTGCTGGAGA

CTCGCCTTAAAGTAATTGGCAGCCGCCTACTGGCTTTCGGAGCGCAAGCACAAGTCGCAGCTctctATCAGCAAAGGTCT

AGCCATCCATTAAGCCTTtttttCAACTTTTGAGCCTCGGATCAGGTAGGGATACCCGCTGA

>OTU_1153

GAAATGCGATAAGTAATGTGAATTGCAGAATTCAGTGAATCATCGAATCTTTGAACGCACCTTGCGCTCCTTGGTATTCC

GAGGAGCATGCCTGTTTGAGTGTCATGAATTCTCAACCCTCAATGCTTTATTGCATTGCTTGGGGTTGGTCCTTGGGTGC

TTGCCGTATGAATTGTACAGCTCACCTCAAATGTATTAGCTGGACCCGTACTTCTTGTGTAGGTGTCCTCAACGTGATAA

AGTCATTCGTTGAGGCGTGTGCAGGCAACTGCTCCCTTACCTTTCTGTACAAGCGTCTGCTTCTAACCCGGCGAACGACT

CTTTGCAGTCTGACCCATGACTTAAAGCTCTGGCCTCAAATCAGGTAGGATTACCCGCTGAACTTAA

>OTU_1154

GAATCGCGATATGTAATGTGATCTGCATTTAGTGAATCATCGAATCTTTGAACGCATCTTGCGCCCAAGGGTAATCCTTT

GGGCACGCCTGTTTCAGTGTTATGTTAAACCCAAAATCTCTTttttGGTGATGTAACGTTGGGCTTGCCAATCATTtttt

tGATCGGTCTTGCTTGGAATGATATCAATTCCAGTCGGATCACCTTATTCTTCAGATCAGGCTTTGGTAGACTACCGAAA

GTCGCGTTTGAACGGATTTAGGCCCGAATTGATGGGATTtttAAACATTTTCAATATTTAACCTGAAATCAGGCGGGACT

ACCCGCTGAACTTAA

>OTU_1155

GAAATGCGATAAGTAGTGTGAATTGCAGAATTTAGTAAATTATTAAATCTTTAAACGTATATTACGCCCCTCGGTATTTC

GTAGGGTATGCCTGTTCGAGCGTTATTTACCTCTTAAGCTCTGCTTAGTGTTGAGCGTTATTCTGCTTTGCGCGAGGACT

CGCCCTAAAGGTATTAGTAGCGGTCGTGCTACTTCTTGCGTAGTATATTACGCTTCTTAAGGCGGTTAATTAGCGTCTAC

AAAGCTTATATTATATCTTAACCTTAAATTAGGTAGGGATACCCGCTAAACTTAA

>OTU_1156

GAAATGCGATAAGTAGTGTGAATTGCAGAATTTAGTAAATTATTAAATCTTTGAACGTATATTGCGCCCCTCGGTATTCC

GTAGGGCATGCCTGTTCGAGCGTTATTTACCCCTTAAGCTCTGCTTAGTGTTGGGCGTTGTTCCGCTTCGcgcgAGGACT

CGCCCTAAAGGTATTAGTAGCGGTTGTGCCACCCCTCGCGCAGTATATTGCGCTTCTTAAGGCGGTTAATTAGCGTCTAC

AAAGCTTACATTATATCTTAACCTTAGATTAGGTAGGGATACCCGCTGAACTTAA

>OTU_1157

GAAATGCGATAAGTAATGTGAATTGCAGAATTCAGTGAATCATCGAATCTTTGAACGCACATTGCGCCCCTTGGTATTCC

GAGGGGCATGCCTATTCGAGCGTCATTATCACCCCTCAAGCTTCGGCTTGGTGTTGAGGCCTGCTGTAAAGGCACCCTCT

AAAATCAGTGGCAGTGCTGTCAGGCTCTAAGCGTAGTAATTTCATCGCTATAGGGTCCTGGTGGATACTCGTCAAAACCc

ccccATTtttAATGATTGACCTCGGATTAGGTAGGGATACCCGCTGAACTTAA

>OTU_1158

GAAATGCGATACGTAATGTGAATTGCAGAATTCAGTGAATCATCGAGTCTTTGAACGCACATTGCGCCCTCTGGTATTCC

GGAGGGCATGCCTGTCCGAGCGTCATTGCTGCCCTCAAGCACGGCTTGtgtgtTGGGCTCCGTCCTCCTTCCGGggggAC

GGGCCCGAAAGGCAGCGGCGGCACCGCGTCCGGTCCTTCAAAGCGTATGGGGCTTTGTCACCCGCTTTGTAGGACTGGCC

GGCGCCTGCCGATTCAACCAAAACTTttttCCAGGTTGACCTTCGGATCAGGTAGGGATACCCGCTGAACTTAA

>OTU_1159

GAAATGCGATAAGTAATGTGAATTGCAGATTTTCAGTGAGTCATCGAATCTTTGAACGCACCTTGCGCTCCTTGGTATTC

CGAGGAGCATGCCTGTTTGAGTGTCATTAAATTCTCAACTCCAAGGGTTTCACGACCTTTCGGAGATTGGACTTGGAGGT

GCTGGCCGCATATTGGTCGGCTCCTCTCAAATGCATCAGCGGAATTCAACCTTTGGCTTCCGGATGTCAGTGTGATAATC

ATGTTGCGCTGTTGTCTGCCAGGTAACCTTTGGTAACAAAGGTTTGCTGGAGCTCACAGATGTCtctcCTCAGCGAGGAC

AACTTtttttAACGTTCGACTCAAATCAGGTAGGACTACCCGCTGAACTTAA

>OTU_1160

GAAATGCGATAAGTAATGTGAATTGCAGAATTCAGTGAATCATCGAATCTTTGAACGCACCTTGCGCCCTTTGGTATTCC

GAAGGGCATGCCTGTTTGAGTGTCATGAAGCCTCACCCCACTTGGGTTtttGCCTGAGCGGTGGTGTATTGGGTGTTGCC

TTGCCAAAGCTCGCCTTAAAACATAAGCACCTTGGATGTAATACGTTTCATCCTTCTGGGTGGCTGATCAACCCCAGCAT

ATTCATGACTCTGGCCTCAAATCAGGTAGGGCTACCCGCTGAAACTTAA

>OTU_1161

GAAATGCGATAAGTAATGTGAATTGCAGAATTCAGTGAATCATCGAATCTTTGAACGCACATTGCGCCCGCCAGTATTCT

GGCGGGCATGCCTGTTCGAGCGTCATTTCAACCCTCGCAAAGCCCTTCGCGGCCGAGCGGTGTTGGGGACCGGCACAACC

CTCCCAGGAGGGCgcgcCcccGAAATACAGTGGCGGTCTCGCTGCAGCCTCCCTTGCGTAGTAGCACACCTCGCATTGGA

GCGCGGCTCGCCACGCCGTAAAACCcccGACTCTCCTGAGTTTGACCTCGGATCAGGTAGGAATACCCGCTGAACTTAA

>OTU_1162

GAAATGCGATAAGTAATGTGAATTGCAGAATTCAGTGAATCATCGAATCTTTGAACGCACATTGCGCCcccTGGTATTCC

GGgggCATGCCTGTTCGAGCGTCATTTCACCACTCAAGCCTCGTCTTGGTATTGGGCAACGCGGTCCGCCTGCGTGCCTC

AAATCTGTCCGGCTGGGTCTTTTCATGTCCCCTAAGCGTTGTGGAAACTATTCGCTAAAGGGTGTTCGGGAGGTCTTACC

GCCGTAAAACAACCCCATTTCTAGGTTTGACCTCGGATCAGGTAGGGATACCCGCTGAACTTAA

>OTU_1163

GAAATGCGAAAAGTAGTGTGAATTGCAGAATTCAGTGAATCATCGAATCTTTGAACGCACATTGCGCCCCTTGGTATTCC

ATGGGGCATGCCTGTTCGAGCGTCATTTTGTACCCTCAAGCACTGCTTGGTGTTGGGCGTTTGTCCTGCAAAGGACTCGC

CTGAAAGCGATTGGCGGCCAACGTACTGGTGGTAGAGCGCAGCACAATTTGCGTCtctcCCTTCTACGTCGGCGTCCATG

AAGCCTTttttCAACGTTTGACCTCGGATCAGGTAGGGATACCCGCTGAACTTAA

>OTU_1164

GAAATGCGATAAGTAGTGTAAATTACAGAATTTAGTAAATTATCGAATCTTTAAACGCACATTGCGCCTCTCGGTATTCC

GTAGAGTATACCTGTTTGAGCGTTATTTACCCCTTAAGCTCTGCTTAGTATTAGGCGTTGTCCCGCTTCGcgcgAGGACT

CGCCCTAAAGGTATTAGCAGCGGTCGTGCCACCcccTCGCGCAGTATATTACGCTTCTTAAGGCGGTTAATTAGCGTCCA

CAAAGCTCACATTACATCTTAACCTTAGATTAGGTAGGGATACCCGCTGAACTTAA

>OTU_1165

GAAATGCGATAAGTAGTGTGAATTGCAGAATTCAGTGAATCATCGAATCTTTGAACGCACATTGCGCCCCTTGGTATTCT

ATGGGGCATGCCTGTTCGAGCGTTATTTGTACCCTTAAGCTCTGCTTGGTGTTGGGTGTTTGTCTACTCCCTGGTGTCTG

GACTCGCCTTAAAGCAATTGGCAGCCAGTGTTTTGGTATTGAAGCGTAGTACATTTTGCGTCTCCAGCCTAGAACGCTTG

CGTCTAGTAAGCCTTtttCCACTTTTAACCTCGGATTAGGTAGGGATACCCGCTAAACTTAA

>OTU_1166

GAAATGCGATAAGTAATGTGAATTGCAGAATTCAGTGAATCATCGAATCTTTGAACGCACATTGCGCCCCGTGGTATTCC

GCGGGGCATGCCTGTTCGAGCGTCATTTCACCACTCGAGTCTGACTCGGTATTGGGCGCCGCGGTCTGTCCGcgcgcCTC

AAAGTCGCCGGCTAGGTGGGTCTGTCCCCGAGCGTTGTGGCCTCACAGTCTCGCTTGGGAGCGCGGATCCATCAAGGCCG

TTAAACACCCCATCAAAGGTTGACCTCGGATCAGGTAGGGATACCCGCTGAACTTAA

>OTU_1167

CCTATTTGATAGGTATTGCACCATGTAGTTATGTATTTCAAGTGTGGTTGTCAAGATTACAACATAGCGTGTTTCATCAA

ACTTCTGGTCCAAATGGCCTGGTTCTCAATGGCTTTCAGATTCTCCCTACATGTAGTCGCAATGTCAAAATCGCAGCGTT

CAATATTGTAGCCTCATCCCTATCACATACTTCCTTACAATGTCGTAGACCAGCAGTAGACTTTGGAATATCAAACATGG

TGATCCGAATTTGGGCAAGCATGTCAAGTGACTAtataCATGCATATAAACACATGTGCATGCATGCATGCACCTG

>OTU_1168

GAAATGCGATAAGTAGTGTGAATTGCAGAATTCAGTGAATCATCGAATCTTTGAACGCACATTGCGCCCTTTGGATACTA

CGAAGGCATGCCTGTTCGAGCGTCATTTGTACCCTAAGCTTTGCTGGTGTTGGGCGTCTTGTCTCTAGCTTTGCTGGAGA

CTCGCCTTAAAGTAATTGGCAGCCGGCCTACTGGTTTCGGAGCGCAGCACAAGTCGCACTctctATCAGCAAAGGTCTAG

CATCCATTAAGCCTTtttttCAACTTTTGACCTCGGATCAGGTAGGGATACCCGCTGAACTTAA

>OTU_1169

GAAATGTAATAAGTAGTGTAAATTACAGAATTTAGTAAATTATTAAATCTTTAAACGCACATTGCGCCCCTTAGTATTCC

GTAGGGCATGCCTATTTAAGCGTTATTTACCTCTTAAGCTCTGCTTAGTGTTAGGCGTTATCCCGCTTCGcgcgAGAACT

TGCTCTAAAGGTATTAGCAGCGGTCGTACCACCCCTCGCGCAGTATATTACGCTTCTTAAGGCGGTTAATTAGCGTCTAC

AAAGCTTATATTATATCTTAACCTTGGATTAGGTAGAGATACCCGCTAAACTTAA

>OTU_1170

GAAATGCGATAAGTAATGTGAATTGCAGAATTCAGTGAATCATCGAATCTTTGAACGCACATTGCGCCCCTTGGCATTCC

GGggggCATGCCTGTTCGAGCGTCATTTCAACCCTCAAGCTTAGCTTGGTATTGAGTCTATGTCAGTAATGGCAGGCTCT

AAAATCAGTGGCGGCGCCGCTGGGTCCTGGAACGTAGTAATATCTCATCGTTACAGGTTCTCGGTGTGCTTCTGCCAAAC

CAAAATTtttCCTTATGGTTGACCTCGGATCAGGTAGGGATACCCGCTGAACTTAA

>OTU_1171

GAATTGCGATAAGTAATGTGAATTGCAGATACTCGTGAATCATTGAATTTTGAACGCACATTGCGCCCTTGAGCATTGCT

CAGGGGCATGCCTGTTTGAGCGTCATTTCCTTCTCAAAAGATAATTTATTATTttttGGTTGTGGGCGATACTCAGGGTT

AGCTTGAAATTGGAGACTGTTTCAGTTCTTttttAATTCAACATCTTTAGCTTCTTTGGAGACGCTGTTCTCGCTGTGAT

GTATTTATGGATTTTATTTCGTTTTACTTTACAAGGGAA

>OTU_1172

AAAATGCGATAGCTGGTGTGAATTGCAGAATCCCGTGAACCATCGAGTCTTTGAACGCAAGTTGCGCCTGAAGCCGTCAG

GCCAAGGGCACGCCTGCCTGGGTGTCACCCTACCCCTTGCCGAAATACACGCCTCCTCCCACTTAAaaaTGAGAAGAGCG

GGTGAAATTTGGGCGGATAAATGGCCTCCCATGGTGAATTATTTCGTGGTTGGCCCAAAAGCAGCACTTGGCTCTTAGGA

AGCCACGACAAGCATTGGCGAAaaaCTCCGGTGATTCCTTGATCGTTGTTGTCGTTGCTGTACTTTGTAGCGTGCTATAA

aaaaTACCTCGGTGCTTGCATCCTAATATTGCGACCcccAGGTCAGGCGGGAACACCTGCTGAGTTTAA

>OTU_1173

GAAATGCGATAAGTAATGTGAATTGCAGAATTCAGTGAATCATCGAATCTTTGAACGCACATTGCGCCCGCTAGTATTCT

GGCGGGCATGCCTGTCCGAGCGTCATTTCAACCCCTCGAGCCcccGGGCCCGGTGTTGGGGAGCGACCCAAGCCCTGCCG

CCTCGGCGGCGGGCGGTCGTCCCTGAAATCTAGTGGCGGTCACGTCGCGGACCCTCTGCGTAGTAGCAACACCTCGCACT

GGAGAGCGGCgcgcCCACGCCCCAAACCACTTttttAAAGGTTGACCTCGGATCAGGTAGGAATACCCGCTGAACTTAA

>OTU_1174

GAAATGCGATAAGTAATGTGAATTGCAGAATTCAGTGAATCATCGAATCTTTGAAGCACATGTGCCCTGGCCTGATTCCG

AGGGGCATGCCTGTTCGAGCGTCATTACACCACTCAAGCTATGCTTGGTATTGGGCGTCGTCCTTAGTTGGGCgcgcCTT

AAAGACCTCGGCGAGGCCACTCCGGCTTTAGGCGTAGTAGAATTTATTCGAACGTCTGTCAAAGGAGAGGAACTCTGCCG

ACTGAAACCTTTATTtttCTAGGTTGACCTCGGATCAGGTAGGGATACCCGCTGAACTTAA

>OTU_1175

GAAATGCGATAAGTAATGTGAATTGCAGAATTCAGTGAATCATCGAATCTTTGAACGCACATTGCGCCCATTAGTATTCT

AGTGGGCATGCCTGTTCGAGCGTCATTTCGACCATCAAGCCCTATTTGCTTGGCGTTGGGAGCTTAGCCTGCTGTTGCGG

GATAACTCCTCAAATatatTGGCGGAGTCGCGAAGACCCCAAGCGTAGTAATTttttCTCGCTTCCGGTgtgtCGGCGCT

GGCGTCCAGCCACTAAACCcccTAATTtttAAAGGTTTGACCTCGGATCAGGTAGGAATACCCGCTGAACTTAA

>OTU_1176

GAAATGCGATAAGTAATGTGAATTGCAGAATTCAGTGAATCATCGAATCTTTGAACGCACATTGCGCCCCTCGGTATTCC

GGgggCATGCCTGTTCGAGCGTCATTACACCAATCAAGCCTCGCTTGGTCTTGGGCGTCGCGGTCCGCCGcgcgcCCTAA

TGTCTCCGGCTGGGTCGTCTGTCCCGAAGCGTTGTGATTCATCAAATCGCTTCTGGGGTGGGTCGGTCCAATCGCCGCTA

AACCTTTATCTATCAGGTTGACCTCGGATCAGGTAGGGATACCCGCTGAACTTAA

>OTU_1177

GAAATGCGATAAGTAATGTGAATTGCAGAATTCAGTGAATCATCGAATCTTTGAACGCACATTGCGCCCCGTGGTATTCC

GCGGGGCATGCCTGTTCGAGCGTCATTTCACCACTCAAGCCTCGCTTGGTATTGGGCGACGCGGTCCGCCGcgcgcCTCA

AATCGACCGGCTGGGTCGATCGCACCCTCAGCGTTGTGGAAACTATTCGCTAAAGGGATCGTGGTCGGTCACGCCGTGAA

ACAACCCAATTtttCAATGGTTGACCTCGGATCAGGTAGGGATACCCGCTGAACTTAA

>OTU_1178

GAAATGCGATAAGTAGTGTAAATTACAGAATTTAGTAAATTATTAAATCTTTAAACGCATATTACGCCCCTTAGTATTCC

GTAGGGCATGCCTGTTTAAGCGTTATTTACCCCTTAAGCTCTGCTTAGTGTTGGGCGTTATTCCGCTTCGCGCAAGGACT

CGCTCTAAAGGTATTAGCAGCGGTTGTGCTACCCCTTGCGCAGTATATTACGCTTCTTAAGGCGGTTAATTAGCGTCTAT

AAAGCTTATATTATATCTTAACCTTAGATTAGGTAGGGATACCCGCTAAACTTAA

>OTU_1179

GAAATGCGATAAGTAATGTGAATTGCAGAATTTAGTGAATCATCGAATCTTTGAACGCACATTGCGCCCCTGGGAATTCC

CAGGGGCATGCCTGTTCGAGCGTCCGTAACAACCTCTCAAGCCTGGCTTGGTCTTGGGACACGCTGCTCTAGCGGCGGTC

CTTAAAAGCAGTGGCGGGCCTATGTAACTCTCCGCGTAGTAATATACATCTCGCGATAGAGAAGCAGAAGGACTTGCCAA

aaaTCCTTAATTGCTCAGGTTGACCTCGAATCAGGTAGGGATACCCGCTGAACTTA

>OTU_1180

GAAGTGCGATAAGCAATGCGAATTGCAGAACCGCGAGTCATCAGATTtttGAACGCAAGTGGCGGCTGCTCGGCAGTCAT

GTTTGTTTCAGTgtgtCCGGTTAATTACATCAAAGAATATAATGTGATTGAAGAAATTCtctcACAAAATATGGAATAAT

GTCAATGAAGACTCCAAACGAAATAGTCACTAAATGGTGATAGTCGTTTGAGGATCGGAATTGGTGAATCATTATTCAAa

aaCAAATTATCCAATTATGGCACCTGAAATAAGCAAGAGTACCCGCTGAACTTAA

>OTU_1181

GAAATGCAATAAGTAGTGTAAATTGTAGAATTTAGTAAATTATTAAATCTTTAAACGCACATTGCGCCCCTTAGTATTCT

ATAGGGCATGCCTATTTAAGCGTTATTTGTACTCTTAAGCTCTGCTTAGTGTTAGGTGTTTATCTCCTCTAGGAGACTCG

CCTTAAAATAATTAGCAGCTAGCATATTAGTATTAAAGCGCAGCATAAGTCGCGCTTCTGTCTATATTTATTAGTATCTA

GCAAGACTATTttttACTCTTAACCTTTAGATTAGGTAGGGATACCCGCTAAACTTAA

>OTU_1182

GAAATGCGATAAGTAATGTGAATTGCAGAATTCAGTGAATCATCGAATCTTTGAACGCACATTGCGCCcccTGGTATTCC

GGggggCATGCCTGTTCGAGCGTCATTTCACCACCTCAAGCCTCGCTTGGTATTGGGCACGCGGTCCGCCGCGTGCCTCA

AATCGACCGGCTGGGTCTTCTGCTCCCCTAAGCGTTGTGGAACTATTCGCTAAGGGTGCTCGGGAGGCTACCGCCGTAAA

ACCAAATCCCATTTCTAAGGTTGACCTCGGATCAGGTAGGGACTACCCGCTGAACTTAA

>OTU_1183

GAAATGCGATAAGTAGTGTGAATTGCAGAATTCAGTGAATCATCGAATCTTTGAACGCACATTGCGCCTTCTGGTATTCC

GGGAGGCATGCCTGTTCGAGCGTCATCAAaaaCCTCAATCAGCAATTTATTGTCGATTGGTCTTGCATTGGAGGCGCAAG

CTTCCTGTGCGAAATTCAATGGCGATGAGCCACGCAACCAAAGCGTAGTAAATTACTCTCGTGATGGTGAAGTGGATGCA

TCCGCCGGAACCccccATATCTAAGTTtttGACCTCGGATCAGGTAGGGATACCCGCTGAACTTAA

>OTU_1184

GAATTGCGATAAGTAATGTGAATTGCAGATACTCGTGAATCATTGAGTTtttGAACGCACATTGCGCCCTTGAGCATTCT

CAGGGGCATGCCTGTTTGAGCGTCATTTCCTTCTCAAAAGATAATTTTATTATTttttGGTTGTGGGCGATACTCAGGGT

TAGCTTGAAATTGGAGACTGTTTCAGTCTTttttAATTCAACACTTAGCTTCTTTGGAGACGCTGTTCTCGCTGTGATGT

ATTTATGGATTTATTCGTTTTACTTTACAAGGGAAATGGTAACGTTACCTTAAGGGTCAAAGGGTTGCTTTTAATATTCA

TCTAAGTT

>OTU_1185

GAAATGCGATAAGTAGTGTAAATTGTAGAATTTAGTAAATTATTAAATCTTTAAACGTATATTACGCTCCTCGGTATTCC

GTAGGGCATACCTATTTAAGCGTTATTTACCCCTTAAGCTCTGCTTAGTGTTAGGCGTTATCCCGCTTCGCGCAAGGACT

CACTCTAAAGGTATTAGCAGCGGTCGTGCTACCCCTCGCGCAGCATATTACGCTTCTTAAGGCGGTTAATTAGCGTCTAC

AAAGCTTATATTATATCTTAACCTTAGATTAGGTAGGGATACCCGCTGAACTTAA

>OTU_1186

GAATTGCGATAAGTAATGTGAATTGCAGATACTCGTGAATCATTGAATTtttGAACGCACATTGCGCCCTTGAGCATTCT

CAGGGGCATGCCTGTTTGAGCGCATTCCTTCTCAAAGAATAATTTATTATTtttGTTTGTGGCGGATATCAGGGTTAGCT

TGAAATTGGAGACTGTTTCAGTCTTttttAATTCAACACTTAGCTTCTTTGGAGACGCTGTTCTCGCTGTGATGTATTTA

TGGATTTATTCGTTTTACTTTACAAGGGAAATGGTAACGTACCTTAGGCAAAGGGTTGCTTTTAATATTCATCAAGTTTG

ACCTCAAATCAGGTAGGATTACCCGCTGAACTTAA

>OTU_1187

GAAATGCGATAAGTAGTATAAATTACAGAATTTAGTAAATTATCAAATCTTTAAACGTATATTGCGCCCCTTAGTATTCC

GTAGGGCATGCCTGTTTAAGCGTTATTTACCCCTTAAGCTCTGCTTAGTGTTAAGCGTTATCCCGCTTCGcgcgAGAACT

CGCTCTAAAGGTATTAGCAGCAGTCGTGCTACCCCTTACGCAGTATATTACGCTTCTTAAGGCGGTTAATTAGCGTCTAT

AAAGCTTATATTACATCTTAACCTTAGATTAGGTAGGGATACCCGCTGAACTTAA

>OTU_1188

GAAATGCGATAAGTAATGTGAATTGCAGAATTCAGTGAATCATCGAGTCTTTGAACGCACATTGCGCCcccTGGTATTCC

GGggggCATGCCTGTCCGAGCGTCATTGCACCCCTCAAGCCCGGCTTGTCCTTGGTTATGTCCTCCGTTCCGGAGGGAGC

AGGCCTCAAATGACAATGGCGGCGAGCCACGCGTCCCGGGTCCTCGAGCGTATGGgggCTTTGTCACCCGCTTTGTAGGC

CGGCCGGTCGCTTGCCCTTCAAGCACAACTTCTTATGTTGACCTCGGATCAGGTAGGGATACCCGCTGAACTTAA

>OTU_1189

GAAATGCGATAAGTAATGTGAATTGCAGAATTCAGTGAATCATCGAATCTTTGAACGCACATTGCGCCCTTTGGTATTCC

GAAGGGCATGCCTATTCGAGCGTCATTATCAACCCTCAAGCTCGGCTTGTTATTGGGTCTTATCGTTCAAGATAGGTCCG

AAAGATAATGACGGCGTCGTGAATGACCCCAGGTGCAGCGAGCTTATAGCATACACTGAGGTGGTCATCTCGGCCCGGTC

TTTCGTTTCGGTTCTGTCAAAGGAATCGAAACACTTtttAAAGGTTGACCTCGGATTAGGTAGGAATACCCGCTGAACTT

AA

>OTU_1190

AAAATGCGATACCTGGTGTGAATTGCAGAATCCCGTGAACCATCGAGTTtttGAACGCAAGTTGCGCCCGAGGCCTTCTG

GTCGAGGGCACGCCTGCCTGGGCGTCACGTCAAAAGACACTCCCAACCCATCCAAGGGGAGGGACGTGGTGTTTGGCCTC

CCGTGCCGCAAGGCGCGGTTGGTCGAAGTTGAGGCTGCCGGCATACCGTGTCGGGCACCGCACGTGGTGGGCGACTTCAA

GTTGTTCTCGGTGCAGCGCCCCGGCACGTAGCTAGCGTGTTGCCCTAAGGACCCATCGACCGTAGCGCTTGCCGCTCGGA

CCGCGACCCCAGGTCAGACGAGACTACCCGCTGAGTTTAA

>OTU_1191

GAAATGCGATAAGTAATGTGAATTGCAGAATTCAGTGAATCATCGAATCTTTGAACGCACATTGCGCCcccccGGAATAC

CAGGgggCATGCCTGTTCGAGCGTCATTTCACCACTCAAGCCTCGCTTGGTATTGGGCAACGCGGTCCGCCGCGTGCCTC

AAATCGACCGGCTGGGTCTTCTGTCCCCTAAGCGTTTGTGGAAACTATTCGCTAAAGGGTGCTCGGGAGGCTACGCCGTA

AAACAAACCCATTTCTAAGGTTGACCTCGAATCAGGTAGGGATACCCGCTGAACTTAA

>OTU_1192

GAAATGCGATAAGTAGTGTGAATTGCAGAATTCAGTGAATCATCGAATCTTTGAACGCACATTGCGCCCCTTGGTATTCC

ATGGGGCATGCCTGTTCGAGCGTCATTTGTCAATTCAAGCTTTGCTTGGTGTTGGGTGTTTGTCCCGCGTTGCGCGTGGA

CTCGCCTTAAAGCGATTGGCAGCCGGCATATTGGCCTTGGAGCGCAGCACAAGTCGCGCTTCTAGTCATAATTGTTGGCG

TCCATTAAGCCTATACATATTCGCTTGACCTCGGATCAGGTAGGGATACCCGCTGAACTTAA

>OTU_1193

GAAATGCGATAAATAATGTGAATTGCAGAATTCAGTGAATCATTAAATCTTTGAACGCATATTACGCCTACCAGCATTCT

AGCAGGCATGCTTATTCAAGTGTCATTTCAACCTTCAAGCTCCACTTAGTGTTAAAGCTCTACAGCTAATATAAGCCCTC

AAAGGTAGTAGCAGACCCTCCTAGAGCCTCCTTTGCATAGTAACTTTACGTCTCACACTAAGATCTAGAGGGACTTTTGC

TGTAAAACCccccAATTTTCTAAAGGTTAACCTCAGATCAAATAGGAATACCTGCTAAACTTAA

>OTU_1194

GGCGACCACCGACCTCGTGCATCGATGAAGAACGCAGCAAAGCGCGATAGGTAATGCGAATTGCAGACGTGAGTCATTGA

ATTtttGAACGCATATTGCGCTATTAGTTTGTCTAATAGCATGCTTGTTGGAGTGATAATCTTCCTCTCAACCATTtttG

GTATGAGGTCTTGCTCCTTTTAGGAGTTAAAATCATGGAAGTGCACACGTTAATTAACTCTGTGCAGTTATACACTTTTC

ATCCTCCAATCAAGCAAGGTTACCCGCTGAACTTAG

>OTU_1195

GAAATGCGATAAGTAATGTGAATTGCAGAATTCAGTGAATCATCGAATCTTTGAACGCACATTGCGCCcccTGGTATTCC

GGgggCATGCCTGTTCGAGCGTCATTTCACCACTCAAGCCTCGCTTGGTATTGGGCAACGCGTCCGCCGCGTGCCTACAA

ACTCGTCCGGCTGGGTCTTCTGTCCCTAAGCGTTGTGGAAACTATTCGCTAAAGGGTGTTCGGGAGGCTACGCCGTAAAC

AACCCCATTTCTTAAGGTTGACCTCGGATCAGGTAGGGATACCCGCTGAACTTAA

>OTU_1196

GAAATGCGATAAGTAGTGTGAATTGCAGAATTCAGTGAATCATCGAATCTTTGAACGCACATTGCGCCCTTTGGTATTCC
[truncated: 138,962 more chars]
