# Supplementary material for: The Biogeography of Fungal Communities Across Different Chinese Wine-Producing Regions Associated With Environmental Factors and Spontaneous Fermentation Performance
Source: Front Microbiol. 2022 Feb 25;12:636639. doi: 10.3389/fmicb.2021.636639 (PMC8914289; doi:10.3389/fmicb.2021.636639)
Supplement: Supplementary Data Sheet 2 — Taxonomic assignment and species relative proportion of the 166 Marselan must and fermented samples. [file Data_Sheet_2.DOCX]

OTU_1 k__Eukaryota;p__Ascomycota;c__Dothideomycetes;o__Dothideales;f__Saccotheciaceae;g__Aureobasidium;s__Aureobasidium pullulans 7e-168 KY294714.1

OTU_10 k__Eukaryota;p__Ascomycota;c__Dothideomycetes;o__Botryosphaeriales;f__Botryosphaeriaceae;g__Lasiodiplodia;s__Lasiodiplodia pseudotheobromae 5e-169 KY419553.1

OTU_100 k__Eukaryota;p__Ascomycota;c__Saccharomycetes;o__Saccharomycetales;f__Saccharomycodaceae;g__Hanseniaspora;s__Hanseniaspora uvarum 0.0 MG020690.1

OTU_1000 k__Eukaryota;p__Unclassified;c__Unclassified;o__Unclassified;f__Unclassified;g__Unclassified;s__uncultured fungus 1e-117 KX220453.1

OTU_1001 k__Eukaryota;p__Unclassified;c__Unclassified;o__Unclassified;f__Unclassified;g__Unclassified;s__fungal endophyte 6e-162 KY022835.1

OTU_1002 k__Eukaryota;p__Unclassified;c__Unclassified;o__Unclassified;f__Unclassified;g__Unclassified;s__uncultured fungus 3e-164 MF569267.1

OTU_1003 k__Eukaryota;p__Unclassified;c__Unclassified;o__Unclassified;f__Unclassified;g__Unclassified;s__uncultured fungus 0.0 AY627794.1

OTU_1004 k__Eukaryota;p__Unclassified;c__Unclassified;o__Unclassified;f__Unclassified;g__Unclassified;s__uncultured fungus 0.0 GQ921798.1

OTU_1005 k__Eukaryota;p__Basidiomycota;c__Cystobasidiomycetes;o__Unclassified;f__Unclassified;g__Buckleyzyma;s__Buckleyzyma phyllomatis 2e-107 NR_077097.1

OTU_1006 k__Eukaryota;p__Unclassified;c__Unclassified;o__Unclassified;f__Unclassified;g__Unclassified;s__uncultured fungus 6e-159 AB520242.1

OTU_1007 k__Eukaryota;p__Unclassified;c__Unclassified;o__Unclassified;f__Unclassified;g__Unclassified;s__uncultured fungus 7e-168 MF570081.1

OTU_1008 k__Eukaryota;p__Ascomycota;c__Dothideomycetes;o__Pleosporales;f__Phaeosphaeriaceae;g__Phaeosphaeria;s__Phaeosphaeria sp. MUT 2127 2e-155 KF993414.1

OTU_1009 k__Eukaryota;p__Unclassified;c__Unclassified;o__Unclassified;f__Unclassified;g__Unclassified;s__uncultured fungus 2e-165 JX984758.1

OTU_101 k__Eukaryota;p__Unclassified;c__Unclassified;o__Unclassified;f__Unclassified;g__Unclassified;s__fungal sp. TZ-2015b 2e-159 KR698833.1

OTU_1010 k__Eukaryota;p__Unclassified;c__Unclassified;o__Unclassified;f__Unclassified;g__Unclassified;s__uncultured fungus 6e-122 JX042762.1

OTU_1011 k__Eukaryota;p__Unclassified;c__Unclassified;o__Unclassified;f__Unclassified;g__Unclassified;s__uncultured fungus 0.0 JX546606.1

OTU_1012 k__Eukaryota;p__Unclassified;c__Unclassified;o__Unclassified;f__Unclassified;g__Unclassified;s__uncultured fungus 5e-110 KT195128.1

OTU_1013 k__Eukaryota;p__Unclassified;c__Unclassified;o__Unclassified;f__Unclassified;g__Unclassified;s__fungal sp. 1e-138 KU325111.1

OTU_1014 k__Eukaryota;p__Unclassified;c__Unclassified;o__Unclassified;f__Unclassified;g__Unclassified;s__uncultured fungus 1e-150 KX193635.1

OTU_1015 k__Eukaryota;p__Unclassified;c__Unclassified;o__Unclassified;f__Unclassified;g__Unclassified;s__uncultured fungus 9e-152 GU174335.1

OTU_1016 k__Eukaryota;p__Unclassified;c__Unclassified;o__Unclassified;f__Unclassified;g__Unclassified;s__uncultured fungus 5e-49 KX194631.1

OTU_1017 k__Eukaryota;p__Basidiomycota;c__Agaricomycetes;o__Unclassified;f__Unclassified;g__Unclassified;s__uncultured Agaricomycetes 0.0 FJ553511.1

OTU_1018 k__Eukaryota;p__Ascomycota;c__Leotiomycetes;o__Unclassified;f__Myxotrichaceae;g__Oidiodendron;s__Oidiodendron muniellensis 4e-154 AJ634700.1

OTU_1019 k__Eukaryota;p__Unclassified;c__Unclassified;o__Unclassified;f__Unclassified;g__Unclassified;s__uncultured fungus 2e-143 KX195808.1

OTU_102 k__Eukaryota;p__Ascomycota;c__Eurotiomycetes;o__Eurotiales;f__Aspergillaceae;g__Penicillium;s__Penicillium fluviserpens 1e-169 NR_137612.1

OTU_1020 k__Eukaryota;p__Ascomycota;c__Sordariomycetes;o__Microascales;f__Microascaceae;g__Lophotrichus;s__Lophotrichus sp. UAMH 11809 1e-175 KM580494.1

OTU_1022 k__Eukaryota;p__Ascomycota;c__Sordariomycetes;o__Hypocreales;f__Ophiocordycipitaceae;g__Hirsutella;s__Hirsutella sp. 6e-156 MG561950.1

OTU_1023 k__Eukaryota;p__Basidiomycota;c__Geminibasidiomycetes;o__Geminibasidiales;f__Geminibasidiaceae;g__Basidioascus;s__Basidioascus sp. 0.0 KY321827.1

OTU_1024 k__Eukaryota;p__Ascomycota;c__Dothideomycetes;o__Pleosporales;f__Phaeosphaeriaceae;g__Neosetophoma;s__Neosetophoma italica 3e-111 KP711356.1

OTU_1025 k__Eukaryota;p__Ascomycota;c__Dothideomycetes;o__Unclassified;f__Unclassified;g__Unclassified;s__uncultured Dothideomycetes 4e-154 FJ553040.1

OTU_1026 k__Eukaryota;p__Ascomycota;c__Unclassified;o__Unclassified;f__Unclassified;g__Unclassified;s__uncultured Tetracladium 2e-162 HG935189.1

OTU_1027 k__Eukaryota;p__Unclassified;c__Unclassified;o__Unclassified;f__Unclassified;g__Unclassified;s__uncultured fungus 1e-12 MF571135.1

OTU_1028 k__Eukaryota;p__Basidiomycota;c__Exobasidiomycetes;o__Exobasidiales;f__Cryptobasidiaceae;g__Acaromyces;s__Acaromyces ingoldii 2e-152 KT998903.1

OTU_1029 k__Eukaryota;p__Unclassified;c__Unclassified;o__Unclassified;f__Unclassified;g__Unclassified;s__uncultured fungus 2e-131 JX456888.1

OTU_103 k__Eukaryota;p__Ascomycota;c__Dothideomycetes;o__Pleosporales;f__Phaeosphaeriaceae;g__Neosetophoma;s__Neosetophoma samarorum 2e-168 KJ173536.1

OTU_1030 k__Eukaryota;p__Unclassified;c__Unclassified;o__Unclassified;f__Unclassified;g__Unclassified;s__uncultured fungus 3e-145 HQ257441.1

OTU_1031 k__Eukaryota;p__Basidiomycota;c__Agaricomycetes;o__Agaricales;f__Psathyrellaceae;g__Coprinopsis;s__Coprinopsis atramentaria 0.0 MG132086.1

OTU_1032 k__Eukaryota;p__Basidiomycota;c__Microbotryomycetes;o__Sporidiobolales;f__Sporidiobolaceae;g__Rhodotorula;s__Rhodotorula sp. YM24636 0.0 JQ320370.1

OTU_1033 k__Eukaryota;p__Ascomycota;c__Dothideomycetes;o__Unclassified;f__Unclassified;g__Unclassified;s__uncultured Sclerostagonospora 4e-123 KP843520.1

OTU_1034 k__Eukaryota;p__Unclassified;c__Unclassified;o__Unclassified;f__Unclassified;g__Unclassified;s__fungal sp. TZ-2015b 0.0 KR698914.1

OTU_1035 k__Eukaryota;p__Unclassified;c__Unclassified;o__Unclassified;f__Unclassified;g__Unclassified;s__uncultured fungus 7e-168 KX193132.1

OTU_1036 k__Eukaryota;p__Ascomycota;c__Leotiomycetes;o__Unclassified;f__Pseudeurotiaceae;g__Geomyces;s__Geomyces sp. 23WI14 2e-155 JX270604.1

OTU_1037 k__Eukaryota;p__Ascomycota;c__Dothideomycetes;o__Dothideales;f__Saccotheciaceae;g__Aureobasidium;s__Aureobasidium pullulans 3e-136 KY294714.1

OTU_1038 k__Eukaryota;p__Unclassified;c__Unclassified;o__Unclassified;f__Unclassified;g__Unclassified;s__uncultured fungus 1e-70 FJ237229.1

OTU_1039 k__Eukaryota;p__Ascomycota;c__Dothideomycetes;o__Dothideales;f__Saccotheciaceae;g__Aureobasidium;s__Aureobasidium pullulans 1e-147 KY294714.1

OTU_104 k__Eukaryota;p__Basidiomycota;c__Tremellomycetes;o__Tremellales;f__Bulleraceae;g__Bullera;s__Bullera alba 0.0 KC460892.1

OTU_1040 k__Eukaryota;p__Basidiomycota;c__Cystobasidiomycetes;o__Unclassified;f__Unclassified;g__Symmetrospora;s__Symmetrospora sp. DMKU5-4 0.0 LC216897.1

OTU_1041 k__Eukaryota;p__Ascomycota;c__Dothideomycetes;o__Pleosporales;f__Didymellaceae;g__Phoma;s__Phoma betae 3e-93 KX239986.1

OTU_1042 k__Eukaryota;p__Ascomycota;c__Sordariomycetes;o__Xylariales;f__Xylariaceae;g__Xylaria;s__Xylaria sp. B101 1e-172 KJ512146.1

OTU_1043 k__Eukaryota;p__Unclassified;c__Unclassified;o__Unclassified;f__Unclassified;g__Unclassified;s__uncultured fungus 2e-94 MF571255.1

OTU_1044 k__Eukaryota;p__Ascomycota;c__Dothideomycetes;o__Capnodiales;f__Teratosphaeriaceae;g__Xenoteratosphaeria;s__Xenoteratosphaeria jonkershoekensis 4e-132 EU707864.1

OTU_1045 k__Eukaryota;p__Unclassified;c__Unclassified;o__Unclassified;f__Unclassified;g__Unclassified;s__uncultured fungus 0.0 MF570521.1

OTU_1046 k__Eukaryota;p__Unclassified;c__Unclassified;o__Unclassified;f__Unclassified;g__Unclassified;s__uncultured fungus 2e-131 MF569277.1

OTU_1047 k__Eukaryota;p__Unclassified;c__Unclassified;o__Unclassified;f__Unclassified;g__Unclassified;s__uncultured fungus 0.0 KR535406.1

OTU_1048 k__Eukaryota;p__Ascomycota;c__Unclassified;o__Unclassified;f__Unclassified;g__Unclassified;s__ascomycete sp. 16606 2e-106 EF120417.1

OTU_1049 k__Eukaryota;p__Unclassified;c__Unclassified;o__Unclassified;f__Unclassified;g__Unclassified;s__uncultured fungus 1e-132 MF571360.1

OTU_105 k__Eukaryota;p__Unclassified;c__Unclassified;o__Unclassified;f__Unclassified;g__Unclassified;s__uncultured fungus 2e-11 KX195142.1

OTU_1050 k__Eukaryota;p__Unclassified;c__Unclassified;o__Unclassified;f__Unclassified;g__Unclassified;s__uncultured fungus 1e-70 KX192511.1

OTU_1051 k__Eukaryota;p__Ascomycota;c__Dothideomycetes;o__Pleosporales;f__Unclassified;g__Unclassified;s__Pleosporales sp. 22 KB-2015 2e-165 KR909181.1

OTU_1053 k__Eukaryota;p__Basidiomycota;c__Tremellomycetes;o__Trichosporonales;f__Trichosporonaceae;g__Cryptococcus;s__Cryptococcus sp. 1 AM07 7e-156 KM246162.1

OTU_1054 k__Eukaryota;p__Unclassified;c__Unclassified;o__Unclassified;f__Unclassified;g__Unclassified;s__fungal sp. CA151RZ 1e-49 KP403992.1

OTU_1055 k__Eukaryota;p__Basidiomycota;c__Exobasidiomycetes;o__Exobasidiales;f__Cryptobasidiaceae;g__Acaromyces;s__Acaromyces ingoldii 4e-157 HM595575.1

OTU_1056 k__Eukaryota;p__Unclassified;c__Unclassified;o__Unclassified;f__Unclassified;g__Unclassified;s__uncultured fungus 4e-52 FJ362303.1

OTU_1057 k__Eukaryota;p__Ascomycota;c__Dothideomycetes;o__Pleosporales;f__Phaeosphaeriaceae;g__Neostagonospora;s__Neostagonospora elegiae 4e-120 KF251164.1

OTU_1058 k__Eukaryota;p__Unclassified;c__Unclassified;o__Unclassified;f__Unclassified;g__Unclassified;s__fungal sp. ARIZ AZ1052 4e-114 HM123697.1

OTU_1059 k__Eukaryota;p__Ascomycota;c__Sordariomycetes;o__Hypocreales;f__Stachybotryaceae;g__Stachybotrys;s__Stachybotrys chlorohalonata 9e-180 MG250466.1

OTU_106 k__Eukaryota;p__Unclassified;c__Unclassified;o__Unclassified;f__Unclassified;g__Unclassified;s__uncultured fungus 9e-158 KF800549.1

OTU_1060 k__Eukaryota;p__Unclassified;c__Unclassified;o__Unclassified;f__Unclassified;g__Unclassified;s__uncultured fungus 2e-149 KC966024.1

OTU_1061 k__Eukaryota;p__Unclassified;c__Unclassified;o__Unclassified;f__Unclassified;g__Unclassified;s__uncultured fungus 0.0 KX193432.1

OTU_1063 k__Eukaryota;p__Unclassified;c__Unclassified;o__Unclassified;f__Unclassified;g__Unclassified;s__uncultured fungus 7e-168 KX516275.1

OTU_1064 k__Eukaryota;p__Ascomycota;c__Dothideomycetes;o__Hysteriales;f__Hysteriaceae;g__Rhytidhysteron;s__Rhytidhysteron rufulum 4e-126 KP162181.1

OTU_1065 k__Eukaryota;p__Unclassified;c__Unclassified;o__Unclassified;f__Unclassified;g__Unclassified;s__uncultured fungus 2e-91 KT196646.1

OTU_1066 k__Eukaryota;p__Basidiomycota;c__Exobasidiomycetes;o__Exobasidiales;f__Exobasidiaceae;g__Exobasidium;s__Exobasidium reticulatum 2e-128 KY038487.1

OTU_1067 k__Eukaryota;p__Unclassified;c__Unclassified;o__Unclassified;f__Unclassified;g__Unclassified;s__uncultured fungus 2e-156 KC966096.1

OTU_1068 k__Eukaryota;p__Ascomycota;c__Dothideomycetes;o__Pleosporales;f__Phaeosphaeriaceae;g__Camarosporioides;s__Camarosporioides phragmitis 5e-144 KX572340.1

OTU_1069 k__Eukaryota;p__Ascomycota;c__Dothideomycetes;o__Pleosporales;f__Arthopyreniaceae;g__Arthopyrenia;s__Arthopyrenia sp. 5e-169 MF671827.1

OTU_107 k__Eukaryota;p__Ascomycota;c__Dothideomycetes;o__Pleosporales;f__Coniothyriaceae;g__Coniothyrium;s__Coniothyrium sp. 7e-168 MG182687.1

OTU_1070 k__Eukaryota;p__Ascomycota;c__Unclassified;o__Unclassified;f__Unclassified;g__Unclassified;s__fungal endophyte MUT 2727 2e-119 AF373063.1

OTU_1071 k__Eukaryota;p__Unclassified;c__Unclassified;o__Unclassified;f__Unclassified;g__Unclassified;s__uncultured fungus 0.0 MF569065.1

OTU_1072 k__Eukaryota;p__Unclassified;c__Unclassified;o__Unclassified;f__Unclassified;g__Unclassified;s__uncultured fungus 2e-94 KM493601.1

OTU_1073 k__Eukaryota;p__Ascomycota;c__Sordariomycetes;o__Hypocreales;f__Nectriaceae;g__Dactylonectria;s__Dactylonectria hordeicola 2e-171 MF440368.1

OTU_1074 k__Eukaryota;p__Unclassified;c__Unclassified;o__Unclassified;f__Unclassified;g__Unclassified;s__fungal sp. CA151RZ 1e-42 KP403992.1

OTU_1075 k__Eukaryota;p__Basidiomycota;c__Agaricomycetes;o__Agaricales;f__Clavariaceae;g__Unclassified;s__uncultured Clavariaceae 1e-126 KX115917.1

OTU_1076 k__Eukaryota;p__Unclassified;c__Unclassified;o__Unclassified;f__Unclassified;g__Unclassified;s__uncultured fungus 3e-164 FN298776.1

OTU_1077 k__Eukaryota;p__Unclassified;c__Unclassified;o__Unclassified;f__Unclassified;g__Unclassified;s__uncultured fungus 1e-46 MF571285.1

OTU_1078 k__Eukaryota;p__Basidiomycota;c__Tremellomycetes;o__Tremellales;f__Bulleraceae;g__Pseudotremella;s__Pseudotremella lacticolor 7e-14 AB375774.1

OTU_1079 k__Eukaryota;p__Ascomycota;c__Dothideomycetes;o__Pleosporales;f__Leptosphaeriaceae;g__Ampelomyces;s__Ampelomyces sp. 2e-165 KX216856.1

OTU_108 k__Eukaryota;p__Unclassified;c__Unclassified;o__Unclassified;f__Unclassified;g__Unclassified;s__uncultured fungus 7e-168 KX515692.1

OTU_1080 k__Eukaryota;p__Unclassified;c__Unclassified;o__Unclassified;f__Unclassified;g__Unclassified;s__uncultured fungus 6e-21 KX221600.1

OTU_1081 k__Eukaryota;p__Unclassified;c__Unclassified;o__Unclassified;f__Unclassified;g__Unclassified;s__uncultured fungus 3e-10 MF570992.1

OTU_1082 k__Eukaryota;p__Unclassified;c__Unclassified;o__Unclassified;f__Unclassified;g__Unclassified;s__uncultured fungus 1e-157 MF569520.1

OTU_1083 k__Eukaryota;p__Unclassified;c__Unclassified;o__Unclassified;f__Unclassified;g__Unclassified;s__uncultured fungus 1e-166 FJ820800.1

OTU_1084 k__Eukaryota;p__Zoopagomycota;c__Unclassified;o__Unclassified;f__Unclassified;g__Unclassified;s__uncultured Entomophthoromycota 1e-175 MF483809.1

OTU_1085 k__Eukaryota;p__Ascomycota;c__Dothideomycetes;o__Dothideales;f__Saccotheciaceae;g__Aureobasidium;s__Aureobasidium sp. BESC15b 4e-126 KC007152.1

OTU_1086 k__Eukaryota;p__Ascomycota;c__Dothideomycetes;o__Capnodiales;f__Cladosporiaceae;g__Cladosporium;s__Cladosporium tenuissimum 7e-128 MG572368.1

OTU_1087 k__Eukaryota;p__Basidiomycota;c__Agaricomycetes;o__Cantharellales;f__Ceratobasidiaceae;g__Ceratobasidium;s__Ceratobasidium sp. 0.0 KY014293.1

OTU_1088 k__Eukaryota;p__Basidiomycota;c__Tremellomycetes;o__Tremellales;f__Rhynchogastremataceae;g__Papiliotrema;s__Papiliotrema flavescens 3e-155 MF783893.1

OTU_1089 k__Eukaryota;p__Ascomycota;c__Dothideomycetes;o__Dothideales;f__Saccotheciaceae;g__Aureobasidium;s__Aureobasidium pullulans 2e-137 KY294714.1

OTU_109 k__Eukaryota;p__Unclassified;c__Unclassified;o__Unclassified;f__Unclassified;g__Unclassified;s__uncultured fungus 7e-168 EF505582.1

OTU_1090 k__Eukaryota;p__Ascomycota;c__Leotiomycetes;o__Helotiales;f__Sclerotiniaceae;g__Unclassified;s__uncultured Botryotinia 4e-132 GU055559.1

OTU_1091 k__Eukaryota;p__Unclassified;c__Unclassified;o__Unclassified;f__Unclassified;g__Unclassified;s__uncultured fungus 1e-49 FJ362303.1

OTU_1092 k__Eukaryota;p__Unclassified;c__Unclassified;o__Unclassified;f__Unclassified;g__Unclassified;s__fungal sp. 3e-142 KY945013.1

OTU_1093 k__Eukaryota;p__Ascomycota;c__Eurotiomycetes;o__Eurotiales;f__Aspergillaceae;g__Penicillium;s__Penicillium indicum 1e-166 NR_121311.1

OTU_1094 k__Eukaryota;p__Unclassified;c__Unclassified;o__Unclassified;f__Unclassified;g__Unclassified;s__uncultured fungus 1e-45 FJ237229.1

OTU_1095 k__Bacteria;p__Proteobacteria;c__Alphaproteobacteria;o__Rhodospirillales;f__Acetobacteraceae;g__Gluconacetobacter;s__Gluconacetobacter diazotrophicus 3e-16 CP001189.1

OTU_1096 k__Eukaryota;p__Unclassified;c__Unclassified;o__Unclassified;f__Unclassified;g__Unclassified;s__uncultured fungus 4e-120 MF568820.1

OTU_1097 k__Eukaryota;p__Ascomycota;c__Dothideomycetes;o__Pleosporales;f__Pleosporaceae;g__Bipolaris;s__Bipolaris bamagaensis 1e-169 KX452447.1

OTU_1099 k__Eukaryota;p__Ascomycota;c__Saccharomycetes;o__Saccharomycetales;f__Saccharomycodaceae;g__Hanseniaspora;s__Hanseniaspora uvarum 2e-147 JX188163.1

OTU_11 k__Eukaryota;p__Ascomycota;c__Saccharomycetes;o__Saccharomycetales;f__Saccharomycodaceae;g__Hanseniaspora;s__Hanseniaspora vineae 0.0 KY103581.1

OTU_110 k__Eukaryota;p__Ascomycota;c__Saccharomycetes;o__Saccharomycetales;f__Saccharomycetaceae;g__Kazachstania;s__Kazachstania exigua 0.0 KY103632.1

OTU_1100 k__Eukaryota;p__Ascomycota;c__Eurotiomycetes;o__Eurotiales;f__Aspergillaceae;g__Penicillium;s__Penicillium sp. QTYC19 7e-128 KM103307.1

OTU_1101 k__Eukaryota;p__Ascomycota;c__Dothideomycetes;o__Pleosporales;f__Pleosporaceae;g__Pleospora;s__Pleospora incompta 6e-125 KU973716.1

OTU_1102 k__Eukaryota;p__Basidiomycota;c__Agaricomycetes;o__Cantharellales;f__Ceratobasidiaceae;g__Unclassified;s__uncultured Ceratobasidium 0.0 JX630384.1

OTU_1103 k__Eukaryota;p__Ascomycota;c__Eurotiomycetes;o__Eurotiales;f__Aspergillaceae;g__Aspergillus;s__Aspergillus niger 4e-154 KX766387.1

OTU_1104 k__Eukaryota;p__Unclassified;c__Unclassified;o__Unclassified;f__Unclassified;g__Unclassified;s__uncultured fungus 4e-148 GU083131.1

OTU_1105 k__Eukaryota;p__Unclassified;c__Unclassified;o__Unclassified;f__Unclassified;g__Unclassified;s__uncultured fungus 8e-140 KF618047.1

OTU_1106 k__Eukaryota;p__Unclassified;c__Unclassified;o__Unclassified;f__Unclassified;g__Unclassified;s__uncultured fungus 1e-172 GU366678.1

OTU_1107 k__Eukaryota;p__Unclassified;c__Unclassified;o__Unclassified;f__Unclassified;g__Unclassified;s__uncultured fungus 6e-156 GU366684.1

OTU_1108 k__Eukaryota;p__Ascomycota;c__Eurotiomycetes;o__Unclassified;f__Unclassified;g__Unclassified;s__Eurotiomycetes sp. 1 KO-2013 2e-156 AB847025.1

OTU_1109 k__Eukaryota;p__Unclassified;c__Unclassified;o__Unclassified;f__Unclassified;g__Unclassified;s__fungal sp. 1e-141 KY945013.1

OTU_111 k__Eukaryota;p__Ascomycota;c__Eurotiomycetes;o__Eurotiales;f__Aspergillaceae;g__Penicillium;s__Penicillium adametzioides 7e-171 KY558605.1

OTU_1110 k__Eukaryota;p__Unclassified;c__Unclassified;o__Unclassified;f__Unclassified;g__Unclassified;s__uncultured fungus 8e-140 KX193279.1

OTU_1111 k__Eukaryota;p__Unclassified;c__Unclassified;o__Unclassified;f__Unclassified;g__Unclassified;s__uncultured fungus 1e-144 JX984715.1

OTU_1112 k__Eukaryota;p__Ascomycota;c__Leotiomycetes;o__Helotiales;f__Unclassified;g__Cadophora;s__Cadophora orchidicola 4e-126 KY271872.1

OTU_1113 k__Eukaryota;p__Ascomycota;c__Leotiomycetes;o__Unclassified;f__Unclassified;g__Unclassified;s__uncultured Leotiomycetes 2e-158 KT581723.1

OTU_1114 k__Eukaryota;p__Unclassified;c__Unclassified;o__Unclassified;f__Unclassified;g__Unclassified;s__uncultured fungus 7e-131 GQ851634.1

OTU_1115 k__Eukaryota;p__Ascomycota;c__Saccharomycetes;o__Saccharomycetales;f__Saccharomycodaceae;g__Hanseniaspora;s__Hanseniaspora vineae 0.0 KY103581.1

OTU_1116 k__Eukaryota;p__Basidiomycota;c__Pucciniomycetes;o__Pucciniales;f__Pucciniaceae;g__Puccinia;s__Puccinia striiformis 0.0 KR230394.1

OTU_1117 k__Eukaryota;p__Ascomycota;c__Dothideomycetes;o__Capnodiales;f__Cladosporiaceae;g__Cladosporium;s__Cladosporium herbarum 1e-123 KX664384.1

OTU_1118 k__Eukaryota;p__Unclassified;c__Unclassified;o__Unclassified;f__Unclassified;g__Unclassified;s__uncultured fungus 4e-157 GQ924068.1

OTU_1119 k__Eukaryota;p__Ascomycota;c__Unclassified;o__Unclassified;f__Unclassified;g__Unclassified;s__uncultured Ascomycota 4e-151 FR682154.1

OTU_112 k__Eukaryota;p__Unclassified;c__Unclassified;o__Unclassified;f__Unclassified;g__Unclassified;s__fungal sp. 7e-168 KY945013.1

OTU_1120 k__Eukaryota;p__Ascomycota;c__Sordariomycetes;o__Hypocreales;f__Unclassified;g__Trichothecium;s__Trichothecium roseum 7e-162 MF288732.1

OTU_1121 k__Eukaryota;p__Ascomycota;c__Dothideomycetes;o__Pleosporales;f__Didymellaceae;g__Phoma;s__Phoma betae 5e-98 EU003450.1

OTU_1122 k__Eukaryota;p__Ascomycota;c__Sordariomycetes;o__Glomerellales;f__Glomerellaceae;g__Colletotrichum;s__Colletotrichum sp. MST 6-3 8e-69 KJ883602.1

OTU_1123 k__Eukaryota;p__Ascomycota;c__Dothideomycetes;o__Pleosporales;f__Leptosphaeriaceae;g__Leptosphaeria;s__Leptosphaeria biglobosa 9e-07 FO906144.1

OTU_1124 k__Eukaryota;p__Unclassified;c__Unclassified;o__Unclassified;f__Unclassified;g__Unclassified;s__uncultured fungus 5e-169 MF570038.1

OTU_1125 k__Eukaryota;p__Ascomycota;c__Sordariomycetes;o__Hypocreales;f__Hypocreaceae;g__Trichoderma;s__Trichoderma harzianum 3e-170 MG572207.1

OTU_1126 k__Eukaryota;p__Unclassified;c__Unclassified;o__Unclassified;f__Unclassified;g__Unclassified;s__uncultured fungus 0.0 MF570485.1

OTU_1127 k__Eukaryota;p__Ascomycota;c__Dothideomycetes;o__Pleosporales;f__Leptosphaeriaceae;g__Leptosphaeria;s__Leptosphaeria biglobosa 9e-07 FO906071.1

OTU_1128 k__Eukaryota;p__Ascomycota;c__Sordariomycetes;o__Glomerellales;f__Glomerellaceae;g__Colletotrichum;s__Colletotrichum gloeosporioides 1e-104 KX906602.1

OTU_1129 k__Eukaryota;p__Basidiomycota;c__Agaricomycetes;o__Agaricales;f__Lyophyllaceae;g__Lyophyllum;s__Lyophyllum shimeji 0.0 KY684031.1

OTU_113 k__Eukaryota;p__Ascomycota;c__Dothideomycetes;o__Pleosporales;f__Phaeosphaeriaceae;g__Chaetosphaeronema;s__Chaetosphaeronema sp. 2e-146 KX774114.1

OTU_1130 k__Eukaryota;p__Basidiomycota;c__Tremellomycetes;o__Tremellales;f__Bulleribasidiaceae;g__Vishniacozyma;s__Vishniacozyma dimennae 4e-157 KM246182.1

OTU_1131 k__Eukaryota;p__Basidiomycota;c__Tremellomycetes;o__Tremellales;f__Trimorphomycetaceae;g__Saitozyma;s__Saitozyma flava 2e-156 NR_073218.1

OTU_1132 k__Eukaryota;p__Ascomycota;c__Dothideomycetes;o__Dothideales;f__Saccotheciaceae;g__Aureobasidium;s__Aureobasidium pullulans 1e-138 KY294714.1

OTU_1133 k__Eukaryota;p__Unclassified;c__Unclassified;o__Unclassified;f__Unclassified;g__Unclassified;s__uncultured fungus 0.0 KU163885.1

OTU_1134 k__Eukaryota;p__Unclassified;c__Unclassified;o__Unclassified;f__Unclassified;g__Unclassified;s__uncultured fungus 1e-138 KX515855.1

OTU_1135 k__Eukaryota;p__Ascomycota;c__Dothideomycetes;o__Pleosporales;f__Pleosporaceae;g__Alternaria;s__Alternaria sp. U53 2e-152 KR906710.1

OTU_1136 k__Eukaryota;p__Ascomycota;c__Sordariomycetes;o__Sordariales;f__Unclassified;g__Unclassified;s__Sordariales sp. MR31-1 6e-153 KT220678.1

OTU_1137 k__Eukaryota;p__Unclassified;c__Unclassified;o__Unclassified;f__Unclassified;g__Unclassified;s__uncultured fungus 0.0 KT799189.1

OTU_1138 k__Eukaryota;p__Ascomycota;c__Dothideomycetes;o__Pleosporales;f__Pleosporaceae;g__Alternaria;s__Alternaria alternata 2e-134 KY065361.1

OTU_1139 k__Eukaryota;p__Ascomycota;c__Leotiomycetes;o__Helotiales;f__Sclerotiniaceae;g__Botrytis;s__Botrytis cinerea 3e-124 KY419551.1

OTU_114 k__Eukaryota;p__Unclassified;c__Unclassified;o__Unclassified;f__Unclassified;g__Unclassified;s__uncultured fungus 3e-164 DQ420828.1

OTU_1140 k__Eukaryota;p__Ascomycota;c__Dothideomycetes;o__Pleosporales;f__Leptosphaeriaceae;g__Leptosphaeria;s__Leptosphaeria biglobosa 4e-09 FO906245.1

OTU_1141 k__Eukaryota;p__Unclassified;c__Unclassified;o__Unclassified;f__Unclassified;g__Unclassified;s__uncultured fungus 8e-146 JX136171.1

OTU_1142 k__Eukaryota;p__Unclassified;c__Unclassified;o__Unclassified;f__Unclassified;g__Unclassified;s__uncultured fungus 3e-90 MF571135.1

OTU_1143 k__Eukaryota;p__Ascomycota;c__Dothideomycetes;o__Pleosporales;f__Leptosphaeriaceae;g__Leptosphaeria;s__Leptosphaeria biglobosa 3e-25 FO906089.1

OTU_1144 k__Eukaryota;p__Ascomycota;c__Sordariomycetes;o__Sordariales;f__Chaetomiaceae;g__Chaetomium;s__Chaetomium madrasense 3e-167 KP055593.1

OTU_1145 k__Eukaryota;p__Ascomycota;c__Unclassified;o__Unclassified;f__Unclassified;g__Phaeodactylium;s__Phaeodactylium stadleri 1e-157 HF678526.1

OTU_1146 k__Eukaryota;p__Ascomycota;c__Dothideomycetes;o__Dothideales;f__Saccotheciaceae;g__Aureobasidium;s__Aureobasidium namibiae 5e-135 KY472292.1

OTU_1147 k__Eukaryota;p__Ascomycota;c__Dothideomycetes;o__Pleosporales;f__Leptosphaeriaceae;g__Leptosphaeria;s__Leptosphaeria biglobosa 4e-18 FO906080.1

OTU_1148 k__Eukaryota;p__Ascomycota;c__Dothideomycetes;o__Pleosporales;f__Pleosporaceae;g__Curvularia;s__Curvularia hawaiiensis 1e-172 KF897857.1

OTU_1149 k__Eukaryota;p__Unclassified;c__Unclassified;o__Unclassified;f__Unclassified;g__Unclassified;s__uncultured fungus 0.0 MF569645.1

OTU_115 k__Eukaryota;p__Unclassified;c__Unclassified;o__Unclassified;f__Unclassified;g__Unclassified;s__uncultured fungus 1e-116 EU144632.1

OTU_1150 k__Eukaryota;p__Ascomycota;c__Dothideomycetes;o__Pleosporales;f__Pleosporaceae;g__Alternaria;s__Alternaria sp. IOC-1546 2e-168 AB975300.1

OTU_1151 k__Eukaryota;p__Unclassified;c__Unclassified;o__Unclassified;f__Unclassified;g__Unclassified;s__uncultured fungus 5e-12 KP889943.1

OTU_1152 k__Eukaryota;p__Unclassified;c__Unclassified;o__Unclassified;f__Unclassified;g__Unclassified;s__uncultured fungus 7e-131 EF504733.1

OTU_1153 k__Eukaryota;p__Unclassified;c__Unclassified;o__Unclassified;f__Unclassified;g__Unclassified;s__uncultured fungus 0.0 MF570161.1

OTU_1154 k__Eukaryota;p__Mucoromycota;c__Unclassified;o__Mucorales;f__Cunninghamellaceae;g__Gongronella;s__Gongronella butleri 0.0 KX911872.1

OTU_1155 k__Eukaryota;p__Unclassified;c__Unclassified;o__Unclassified;f__Unclassified;g__Unclassified;s__uncultured fungus 2e-35 FJ237229.1

OTU_1156 k__Eukaryota;p__Ascomycota;c__Dothideomycetes;o__Capnodiales;f__Mycosphaerellaceae;g__Septoria;s__cf. Septoria sp. CPC 19311 3e-53 KF251241.1

OTU_1157 k__Eukaryota;p__Ascomycota;c__Unclassified;o__Unclassified;f__Unclassified;g__Unclassified;s__uncultured Tetracladium 2e-158 HG935221.1

OTU_1158 k__Eukaryota;p__Ascomycota;c__Eurotiomycetes;o__Eurotiales;f__Aspergillaceae;g__Unclassified;s__uncultured Penicillium 2e-149 KF385279.1

OTU_1159 k__Eukaryota;p__Basidiomycota;c__Agaricomycetes;o__Atheliales;f__Atheliaceae;g__Piloderma;s__Piloderma sphaerosporum 3e-07 MF926559.1

OTU_116 k__Eukaryota;p__Ascomycota;c__Sordariomycetes;o__Glomerellales;f__Glomerellaceae;g__Colletotrichum;s__Colletotrichum acutatum 2e-159 MF170676.1

OTU_1160 k__Eukaryota;p__Basidiomycota;c__Tremellomycetes;o__Tremellales;f__Bulleribasidiaceae;g__Vishniacozyma;s__Vishniacozyma carnescens 2e-131 MG250423.1

OTU_1161 k__Eukaryota;p__Unclassified;c__Unclassified;o__Unclassified;f__Unclassified;g__Unclassified;s__fungal sp. P4 1e-169 KF887075.1

OTU_1162 k__Eukaryota;p__Ascomycota;c__Dothideomycetes;o__Capnodiales;f__Cladosporiaceae;g__Cladosporium;s__Cladosporium herbarum 1e-123 KF588637.1

OTU_1163 k__Eukaryota;p__Ascomycota;c__Dothideomycetes;o__Pleosporales;f__Leptosphaeriaceae;g__Leptosphaeria;s__Leptosphaeria sp. 1e-163 MF327242.1

OTU_1164 k__Eukaryota;p__Unclassified;c__Unclassified;o__Unclassified;f__Unclassified;g__Unclassified;s__uncultured fungus 5e-64 FJ362304.1

OTU_1165 k__Eukaryota;p__Unclassified;c__Unclassified;o__Unclassified;f__Unclassified;g__Unclassified;s__fungal sp. 603 AI-2013 5e-144 KC662236.1

OTU_1166 k__Eukaryota;p__Ascomycota;c__Dothideomycetes;o__Capnodiales;f__Mycosphaerellaceae;g__Mycosphaerella;s__Mycosphaerella sp. 13E036 7e-165 LC163515.1

OTU_1168 k__Eukaryota;p__Unclassified;c__Unclassified;o__Unclassified;f__Unclassified;g__Unclassified;s__uncultured fungus 5e-147 EF504700.1

OTU_1169 k__Eukaryota;p__Ascomycota;c__Dothideomycetes;o__Pleosporales;f__Leptosphaeriaceae;g__Leptosphaeria;s__Leptosphaeria biglobosa 9e-07 FO906079.1

OTU_117 k__Eukaryota;p__Basidiomycota;c__Tremellomycetes;o__Tremellales;f__Rhynchogastremataceae;g__Papiliotrema;s__Papiliotrema laurentii 0.0 KY445944.1

OTU_1170 k__Eukaryota;p__Ascomycota;c__Unclassified;o__Unclassified;f__Unclassified;g__Unclassified;s__uncultured Ascomycota 2e-143 AM901946.1

OTU_1171 k__Eukaryota;p__Ascomycota;c__Saccharomycetes;o__Saccharomycetales;f__Saccharomycodaceae;g__Hanseniaspora;s__Hanseniaspora uvarum 1e-122 KY366247.1

OTU_1172 k__Eukaryota;p__Unclassified;c__Unclassified;o__Unclassified;f__Unclassified;g__Unclassified;s__uncultured fungus 5e-37 MF570568.1

OTU_1173 k__Eukaryota;p__Ascomycota;c__Unclassified;o__Unclassified;f__Unclassified;g__Acremonium;s__Acremonium sp. 2e-140 KX788188.1

OTU_1174 k__Eukaryota;p__Ascomycota;c__Dothideomycetes;o__Dothideales;f__Saccotheciaceae;g__Aureobasidium;s__Aureobasidium pullulans 2e-140 KY294714.1

OTU_1175 k__Eukaryota;p__Ascomycota;c__Sordariomycetes;o__Xylariales;f__Diatrypaceae;g__Eutypella;s__Eutypella sp. 1e-172 KX828160.1

OTU_1176 k__Eukaryota;p__Ascomycota;c__Dothideomycetes;o__Capnodiales;f__Teratosphaeriaceae;g__Teratosphaeria;s__Teratosphaeria sp. CPC 13963 1e-147 EU707886.1

OTU_1177 k__Eukaryota;p__Ascomycota;c__Dothideomycetes;o__Capnodiales;f__Cladosporiaceae;g__Toxicocladosporium;s__Toxicocladosporium strelitziae 2e-158 KU255041.1

OTU_1178 k__Eukaryota;p__Ascomycota;c__Dothideomycetes;o__Pleosporales;f__Leptosphaeriaceae;g__Leptosphaeria;s__Leptosphaeria biglobosa 9e-07 FO906071.1

OTU_1179 k__Eukaryota;p__Unclassified;c__Unclassified;o__Unclassified;f__Unclassified;g__Unclassified;s__uncultured fungus 3e-164 KX193689.1

OTU_118 k__Eukaryota;p__Ascomycota;c__Dothideomycetes;o__Capnodiales;f__Schizothyriaceae;g__Zygophiala;s__Zygophiala qianensis 6e-159 KF806030.1

OTU_1180 k__Eukaryota;p__Unclassified;c__Unclassified;o__Unclassified;f__Unclassified;g__Unclassified;s__uncultured fungus 8e-143 MF570623.1

OTU_1181 k__Eukaryota;p__Ascomycota;c__Dothideomycetes;o__Pleosporales;f__Leptosphaeriaceae;g__Leptosphaeria;s__Leptosphaeria biglobosa 6e-45 FO906080.1

OTU_1182 k__Eukaryota;p__Ascomycota;c__Dothideomycetes;o__Capnodiales;f__Cladosporiaceae;g__Cladosporium;s__Cladosporium sp. 2e-125 MG572462.1

OTU_1183 k__Eukaryota;p__Ascomycota;c__Unclassified;o__Unclassified;f__Unclassified;g__Unclassified;s__uncultured Ascomycota 1e-144 FR682424.1

OTU_1184 k__Eukaryota;p__Ascomycota;c__Saccharomycetes;o__Saccharomycetales;f__Saccharomycodaceae;g__Hanseniaspora;s__Hanseniaspora uvarum 2e-159 KT758330.1

OTU_1185 k__Eukaryota;p__Ascomycota;c__Dothideomycetes;o__Pleosporales;f__Leptosphaeriaceae;g__Leptosphaeria;s__Leptosphaeria biglobosa 9e-07 FO906079.1

OTU_1186 k__Eukaryota;p__Ascomycota;c__Saccharomycetes;o__Saccharomycetales;f__Saccharomycodaceae;g__Hanseniaspora;s__Hanseniaspora uvarum 9e-171 MG020690.1

OTU_1187 k__Eukaryota;p__Ascomycota;c__Dothideomycetes;o__Pleosporales;f__Leptosphaeriaceae;g__Leptosphaeria;s__Leptosphaeria biglobosa 9e-07 FO906071.1

OTU_1188 k__Eukaryota;p__Ascomycota;c__Eurotiomycetes;o__Eurotiales;f__Aspergillaceae;g__Penicillium;s__Penicillium sp. QTYC19 5e-129 KM103307.1

OTU_1189 k__Eukaryota;p__Ascomycota;c__Eurotiomycetes;o__Chaetothyriales;f__Unclassified;g__Unclassified;s__uncultured Chaetothyriales 3e-127 KJ828668.1

OTU_119 k__Eukaryota;p__Ascomycota;c__Dothideomycetes;o__Dothideales;f__Saccotheciaceae;g__Aureobasidium;s__Aureobasidium pullulans 2e-168 KX009459.1

OTU_1190 k__Eukaryota;p__Unclassified;c__Unclassified;o__Unclassified;f__Unclassified;g__Unclassified;s__uncultured fungus 4e-111 MF570027.1

OTU_1191 k__Eukaryota;p__Unclassified;c__Unclassified;o__Unclassified;f__Unclassified;g__Unclassified;s__uncultured fungus 2e-146 GQ851639.1

OTU_1192 k__Eukaryota;p__Ascomycota;c__Dothideomycetes;o__Pleosporales;f__Coniothyriaceae;g__Coniothyrium;s__Coniothyrium hakeae 3e-139 KY173397.1

OTU_1193 k__Eukaryota;p__Ascomycota;c__Sordariomycetes;o__Glomerellales;f__Glomerellaceae;g__Colletotrichum;s__Colletotrichum gloeosporioides 3e-71 KX906602.1

OTU_1194 k__Eukaryota;p__Ascomycota;c__Saccharomycetes;o__Saccharomycetales;f__Unclassified;g__Unclassified;s__uncultured Candida 5e-141 GU138079.1

OTU_1195 k__Eukaryota;p__Ascomycota;c__Dothideomycetes;o__Capnodiales;f__Cladosporiaceae;g__Cladosporium;s__Cladosporium sp. 1 BRM-2015 7e-137 KP309884.1

OTU_1196 k__Eukaryota;p__Ascomycota;c__Dothideomycetes;o__Pleosporales;f__Pleosporaceae;g__Alternaria;s__Alternaria sp. Utah 10 5e-144 KJ541481.1

OTU_1197 k__Eukaryota;p__Ascomycota;c__Dothideomycetes;o__Dothideales;f__Saccotheciaceae;g__Aureobasidium;s__Aureobasidium pullulans 8e-134 KY294714.1

OTU_1198 k__Eukaryota;p__Ascomycota;c__Dothideomycetes;o__Pleosporales;f__Didymellaceae;g__Epicoccum;s__Epicoccum sp. 4e-157 KX721521.1

OTU_1199 k__Eukaryota;p__Ascomycota;c__Dothideomycetes;o__Pleosporales;f__Phaeosphaeriaceae;g__Parastagonospora;s__Parastagonospora nodorum 4e-12 CP022842.1

OTU_12 k__Eukaryota;p__Ascomycota;c__Saccharomycetes;o__Saccharomycetales;f__Unclassified;g__Candida;s__Candida quercitrusa 0.0 MF574303.1

OTU_120 k__Eukaryota;p__Ascomycota;c__Eurotiomycetes;o__Eurotiales;f__Trichocomaceae;g__Talaromyces;s__Talaromyces verruculosus 7e-168 MG551571.1

OTU_1200 k__Eukaryota;p__Ascomycota;c__Dothideomycetes;o__Capnodiales;f__Mycosphaerellaceae;g__Pseudocercospora;s__Pseudocercospora proiphydis 6e-79 NR_147282.1

OTU_1201 k__Eukaryota;p__Unclassified;c__Unclassified;o__Unclassified;f__Unclassified;g__Unclassified;s__uncultured fungus 1e-09 MF568705.1

OTU_1202 k__Eukaryota;p__Unclassified;c__Unclassified;o__Unclassified;f__Unclassified;g__Unclassified;s__uncultured fungus 9e-72 KY978242.1

OTU_1203 k__Eukaryota;p__Ascomycota;c__Dothideomycetes;o__Pleosporales;f__Unclassified;g__Unclassified;s__Pleosporales sp. XJ7 1e-166 KF143782.1

OTU_1204 k__Eukaryota;p__Unclassified;c__Unclassified;o__Unclassified;f__Unclassified;g__Unclassified;s__uncultured fungus 3e-161 KX194779.1

OTU_1205 k__Eukaryota;p__Ascomycota;c__Dothideomycetes;o__Dothideales;f__Saccotheciaceae;g__Aureobasidium;s__Aureobasidium pullulans 2e-140 KY294714.1

OTU_1206 k__Eukaryota;p__Ascomycota;c__Unclassified;o__Unclassified;f__Unclassified;g__Unclassified;s__uncultured Trichocladium 2e-155 KM062090.1

OTU_1207 k__Eukaryota;p__Unclassified;c__Unclassified;o__Unclassified;f__Unclassified;g__Unclassified;s__uncultured fungus 1e-117 KX515738.1

OTU_1209 k__Eukaryota;p__Basidiomycota;c__Agaricomycetes;o__Boletales;f__Suillaceae;g__Suillus;s__Suillus brevipes 0.0 KX230622.1

OTU_121 k__Eukaryota;p__Ascomycota;c__Saccharomycetes;o__Saccharomycetales;f__Pichiaceae;g__Pichia;s__Pichia kudriavzevii 7e-171 MG388319.1

OTU_1210 k__Eukaryota;p__Unclassified;c__Unclassified;o__Unclassified;f__Unclassified;g__Unclassified;s__uncultured fungus 2e-161 KX194413.1

OTU_1211 k__Eukaryota;p__Unclassified;c__Unclassified;o__Unclassified;f__Unclassified;g__Unclassified;s__uncultured fungus 1e-169 MF569885.1

OTU_1212 k__Eukaryota;p__Unclassified;c__Unclassified;o__Unclassified;f__Unclassified;g__Unclassified;s__uncultured fungus 3e-136 FN298731.1

OTU_1213 k__Eukaryota;p__Ascomycota;c__Leotiomycetes;o__Unclassified;f__Myxotrichaceae;g__Oidiodendron;s__Oidiodendron sp. CC 04-26 2e-155 KF359578.1

OTU_1214 k__Eukaryota;p__Unclassified;c__Unclassified;o__Unclassified;f__Unclassified;g__Unclassified;s__uncultured fungus 2e-143 KC978042.1

OTU_1215 k__Eukaryota;p__Unclassified;c__Unclassified;o__Unclassified;f__Unclassified;g__Unclassified;s__uncultured fungus 1e-126 MF570555.1

OTU_1216 k__Eukaryota;p__Unclassified;c__Unclassified;o__Unclassified;f__Unclassified;g__Unclassified;s__uncultured fungus 0.0 MF570072.1

OTU_1217 k__Eukaryota;p__Unclassified;c__Unclassified;o__Unclassified;f__Unclassified;g__Unclassified;s__uncultured fungus 7e-54 KX193383.1

OTU_1218 k__Eukaryota;p__Unclassified;c__Unclassified;o__Unclassified;f__Unclassified;g__Unclassified;s__uncultured fungus 9e-78 FJ237229.1

OTU_1219 k__Eukaryota;p__Unclassified;c__Unclassified;o__Unclassified;f__Unclassified;g__Unclassified;s__fungal sp. 9e-143 KY945013.1

OTU_122 k__Eukaryota;p__Ascomycota;c__Dothideomycetes;o__Capnodiales;f__Mycosphaerellaceae;g__Mycosphaerella;s__Mycosphaerella polygoni-cuspidati 7e-94 AB434910.1

OTU_1220 k__Eukaryota;p__Ascomycota;c__Saccharomycetes;o__Saccharomycetales;f__Saccharomycetaceae;g__Kluyveromyces;s__Kluyveromyces lactis 0.0 CP021242.1

OTU_1221 k__Eukaryota;p__Ascomycota;c__Sordariomycetes;o__Glomerellales;f__Glomerellaceae;g__Colletotrichum;s__Colletotrichum gloeosporioides 5e-144 KX906602.1

OTU_1222 k__Eukaryota;p__Ascomycota;c__Dothideomycetes;o__Pleosporales;f__Melanommataceae;g__Phragmocephala;s__Phragmocephala atra 4e-132 KP698721.1

OTU_1223 k__Eukaryota;p__Basidiomycota;c__Microbotryomycetes;o__Sporidiobolales;f__Sporidiobolaceae;g__Rhodotorula;s__Rhodotorula glutinis 9e-177 KY611826.1

OTU_1224 k__Eukaryota;p__Unclassified;c__Unclassified;o__Unclassified;f__Unclassified;g__Unclassified;s__fungal sp. CA151RZ 1e-45 KP403992.1

OTU_1225 k__Eukaryota;p__Unclassified;c__Unclassified;o__Unclassified;f__Unclassified;g__Unclassified;s__uncultured fungus 2e-100 KX221236.1

OTU_1226 k__Eukaryota;p__Ascomycota;c__Sordariomycetes;o__Hypocreales;f__Unclassified;g__Acremonium;s__Acremonium sclerotigenum 4e-148 KY929277.1

OTU_1227 k__Eukaryota;p__Unclassified;c__Unclassified;o__Unclassified;f__Unclassified;g__Unclassified;s__uncultured fungus 6e-88 GU312824.1

OTU_1228 k__Eukaryota;p__Unclassified;c__Unclassified;o__Unclassified;f__Unclassified;g__Unclassified;s__uncultured fungus 2e-152 KX515551.1

OTU_1229 k__Eukaryota;p__Ascomycota;c__Saccharomycetes;o__Saccharomycetales;f__Saccharomycodaceae;g__Hanseniaspora;s__Hanseniaspora vineae 2e-168 KY103581.1

OTU_123 k__Eukaryota;p__Unclassified;c__Unclassified;o__Unclassified;f__Unclassified;g__Unclassified;s__uncultured fungus 4e-166 MF568850.1

OTU_1230 k__Eukaryota;p__Unclassified;c__Unclassified;o__Unclassified;f__Unclassified;g__Unclassified;s__uncultured fungus 0.0 HM044627.1

OTU_1231 k__Eukaryota;p__Unclassified;c__Unclassified;o__Unclassified;f__Unclassified;g__Unclassified;s__uncultured fungus 0.0 MF568812.1

OTU_1232 k__Eukaryota;p__Ascomycota;c__Dothideomycetes;o__Pleosporales;f__Didymellaceae;g__Phoma;s__Phoma betae 4e-86 KC460811.1

OTU_1233 k__Eukaryota;p__Unclassified;c__Unclassified;o__Unclassified;f__Unclassified;g__Unclassified;s__fungal sp. d3 4e-157 GQ922553.1

OTU_1234 k__Eukaryota;p__Basidiomycota;c__Agaricomycetes;o__Thelephorales;f__Thelephoraceae;g__Unclassified;s__uncultured Thelephoraceae 0.0 JF960834.1

OTU_1235 k__Bacteria;p__Proteobacteria;c__Alphaproteobacteria;o__Rhodospirillales;f__Acetobacteraceae;g__Gluconobacter;s__Gluconobacter oxydans 9e-177 LT900338.1

OTU_1236 k__Eukaryota;p__Ascomycota;c__Dothideomycetes;o__Dothideales;f__Saccotheciaceae;g__Aureobasidium;s__Aureobasidium pullulans 5e-135 KF887089.1

OTU_1237 k__Eukaryota;p__Ascomycota;c__Dothideomycetes;o__Unclassified;f__Unclassified;g__Scleroramularia;s__Scleroramularia abundans 8e-174 FR716675.1

OTU_1238 k__Eukaryota;p__Ascomycota;c__Dothideomycetes;o__Dothideales;f__Saccotheciaceae;g__Aureobasidium;s__Aureobasidium pullulans 8e-143 KX249734.1

OTU_1239 k__Eukaryota;p__Ascomycota;c__Sordariomycetes;o__Hypocreales;f__Nectriaceae;g__Fusicolla;s__Fusicolla acetilerea 2e-174 KF494020.1

OTU_124 k__Eukaryota;p__Unclassified;c__Unclassified;o__Unclassified;f__Unclassified;g__Unclassified;s__uncultured fungus 3e-69 MG162171.1

OTU_1240 k__Eukaryota;p__Basidiomycota;c__Tremellomycetes;o__Tremellales;f__Bulleribasidiaceae;g__Vishniacozyma;s__Vishniacozyma carnescens 7e-131 MG250423.1

OTU_1241 k__Eukaryota;p__Unclassified;c__Unclassified;o__Unclassified;f__Unclassified;g__Unclassified;s__uncultured fungus 3e-155 MF568823.1

OTU_1242 k__Eukaryota;p__Unclassified;c__Unclassified;o__Unclassified;f__Unclassified;g__Unclassified;s__uncultured fungus 4e-06 FJ362304.1

OTU_1243 k__Eukaryota;p__Basidiomycota;c__Agaricomycetes;o__Cantharellales;f__Ceratobasidiaceae;g__Rhizoctonia;s__Rhizoctonia sp. AG-I 0.0 KX964588.1

OTU_1244 k__Eukaryota;p__Unclassified;c__Unclassified;o__Unclassified;f__Unclassified;g__Unclassified;s__uncultured fungus 3e-127 EF505445.1

OTU_1245 k__Eukaryota;p__Unclassified;c__Unclassified;o__Unclassified;f__Unclassified;g__Unclassified;s__uncultured fungus 7e-42 JN853786.1

OTU_1246 k__Eukaryota;p__Unclassified;c__Unclassified;o__Unclassified;f__Unclassified;g__Unclassified;s__uncultured fungus 3e-164 MF976666.1

OTU_1247 k__Eukaryota;p__Ascomycota;c__Arthoniomycetes;o__Lichenostigmatales;f__Phaeococcomycetaceae;g__Phaeococcomyces;s__Phaeococcomyces aff. nigricans P40D010 1e-157 JX188194.1

OTU_1248 k__Eukaryota;p__Ascomycota;c__Dothideomycetes;o__Pleosporales;f__Thyridariaceae;g__Unclassified;s__Roussoellaceae sp. MUT 5381 9e-152 KU314959.1

OTU_1249 k__Eukaryota;p__Unclassified;c__Unclassified;o__Unclassified;f__Unclassified;g__Unclassified;s__uncultured fungus 3e-13 MF571014.1

OTU_1250 k__Eukaryota;p__Unclassified;c__Unclassified;o__Unclassified;f__Unclassified;g__Unclassified;s__uncultured fungus 1e-12 KP889943.1

OTU_1251 k__Eukaryota;p__Ascomycota;c__Dothideomycetes;o__Pleosporales;f__Leptosphaeriaceae;g__Leptosphaeria;s__Leptosphaeria biglobosa 9e-07 FO906144.1

OTU_1252 k__Eukaryota;p__Ascomycota;c__Dothideomycetes;o__Pleosporales;f__Pleosporaceae;g__Alternaria;s__Alternaria alternata 2e-149 KR149267.1

OTU_1253 k__Eukaryota;p__Ascomycota;c__Sordariomycetes;o__Sordariales;f__Lasiosphaeriaceae;g__Unclassified;s__uncultured Cercophora 7e-165 HG936673.1

OTU_1254 k__Eukaryota;p__Ascomycota;c__Dothideomycetes;o__Pleosporales;f__Leptosphaeriaceae;g__Leptosphaeria;s__Leptosphaeria biglobosa 9e-07 FO906072.1

OTU_1255 k__Eukaryota;p__Ascomycota;c__Dothideomycetes;o__Pleosporales;f__Phaeosphaeriaceae;g__Chaetosphaeronema;s__Chaetosphaeronema sp. 1e-160 KX611055.1

OTU_1256 k__Eukaryota;p__Unclassified;c__Unclassified;o__Unclassified;f__Unclassified;g__Unclassified;s__uncultured fungus 2e-131 KX193160.1

OTU_1257 k__Eukaryota;p__Basidiomycota;c__Agaricomycetes;o__Boletales;f__Rhizopogonaceae;g__Unclassified;s__uncultured Rhizopogon 0.0 AB839387.1

OTU_1258 k__Eukaryota;p__Ascomycota;c__Dothideomycetes;o__Pleosporales;f__Didymellaceae;g__Phoma;s__Phoma sp. 4e-129 KY977449.1

OTU_1259 k__Eukaryota;p__Basidiomycota;c__Agaricomycetes;o__Agaricales;f__Psathyrellaceae;g__Coprinopsis;s__Coprinopsis scobicola 2e-162 NR_148065.1

OTU_126 k__Eukaryota;p__Ascomycota;c__Dothideomycetes;o__Pleosporales;f__Pleosporaceae;g__Curvularia;s__Curvularia spicifera 5e-172 KT351794.1

OTU_1260 k__Eukaryota;p__Unclassified;c__Unclassified;o__Unclassified;f__Unclassified;g__Unclassified;s__uncultured fungus 0.0 GQ921822.1

OTU_1261 k__Eukaryota;p__Ascomycota;c__Sordariomycetes;o__Xylariales;f__Xylariaceae;g__Ascotricha;s__Ascotricha erinacea 3e-170 KT224876.1

OTU_1262 k__Eukaryota;p__Basidiomycota;c__Tremellomycetes;o__Tremellales;f__Bulleribasidiaceae;g__Vishniacozyma;s__Vishniacozyma carnescens 8e-143 KY105818.1

OTU_1263 k__Eukaryota;p__Unclassified;c__Unclassified;o__Unclassified;f__Unclassified;g__Unclassified;s__uncultured fungus 3e-59 FJ362304.1

OTU_1264 k__Eukaryota;p__Unclassified;c__Unclassified;o__Unclassified;f__Unclassified;g__Unclassified;s__uncultured fungus 2e-14 MF571135.1

OTU_1265 k__Eukaryota;p__Unclassified;c__Unclassified;o__Unclassified;f__Unclassified;g__Unclassified;s__uncultured fungus 9e-75 FJ237229.1

OTU_1266 k__Eukaryota;p__Ascomycota;c__Dothideomycetes;o__Pleosporales;f__Leptosphaeriaceae;g__Leptosphaeria;s__Leptosphaeria biglobosa 4e-09 FO906072.1

OTU_1267 k__Eukaryota;p__Basidiomycota;c__Tremellomycetes;o__Tremellales;f__Bulleribasidiaceae;g__Vishniacozyma;s__Vishniacozyma dimennae 4e-160 KF981862.1

OTU_1268 k__Eukaryota;p__Ascomycota;c__Leotiomycetes;o__Unclassified;f__Myxotrichaceae;g__Oidiodendron;s__Oidiodendron maius 1e-147 KU382495.1

OTU_1269 k__Eukaryota;p__Ascomycota;c__Sordariomycetes;o__Glomerellales;f__Glomerellaceae;g__Colletotrichum;s__Colletotrichum gloeosporioides 8e-140 KX906602.1

OTU_127 k__Eukaryota;p__Basidiomycota;c__Microbotryomycetes;o__Sporidiobolales;f__Sporidiobolaceae;g__Rhodotorula;s__Rhodotorula paludigena 0.0 KX811211.1

OTU_1270 k__Eukaryota;p__Basidiomycota;c__Tremellomycetes;o__Unclassified;f__Unclassified;g__Cryptococcus;s__Cryptococcus sp. SY2S01 6e-150 FJ153165.1

OTU_1271 k__Eukaryota;p__Ascomycota;c__Dothideomycetes;o__Pleosporales;f__Leptosphaeriaceae;g__Leptosphaeria;s__Leptosphaeria biglobosa 9e-07 FO906079.1

OTU_1272 k__Eukaryota;p__Ascomycota;c__Dothideomycetes;o__Pleosporales;f__Didymellaceae;g__Phoma;s__Phoma betae 9e-115 KC460811.1

OTU_1273 k__Eukaryota;p__Ascomycota;c__Saccharomycetes;o__Saccharomycetales;f__Phaffomycetaceae;g__Starmera;s__[Candida] stellimalicola 1e-169 FM199968.1

OTU_1274 k__Eukaryota;p__Unclassified;c__Unclassified;o__Unclassified;f__Unclassified;g__Unclassified;s__uncultured fungus 1e-132 GU083313.1

OTU_1275 k__Eukaryota;p__Basidiomycota;c__Tremellomycetes;o__Tremellales;f__Unclassified;g__Unclassified;s__uncultured Tremellales 0.0 KM067409.1

OTU_1276 k__Eukaryota;p__Ascomycota;c__Dothideomycetes;o__Pleosporales;f__Unclassified;g__Unclassified;s__uncultured Pleosporales 9e-152 GU909731.1

OTU_1277 k__Eukaryota;p__Unclassified;c__Unclassified;o__Unclassified;f__Unclassified;g__Unclassified;s__uncultured fungus 1e-101 FJ237229.1

OTU_1278 k__Eukaryota;p__Unclassified;c__Unclassified;o__Unclassified;f__Unclassified;g__Unclassified;s__uncultured fungus 1e-45 FJ362304.1

OTU_1279 k__Eukaryota;p__Ascomycota;c__Sordariomycetes;o__Hypocreales;f__Hypocreaceae;g__Trichoderma;s__Trichoderma sp. KBS0814F 1e-157 JQ437611.1

OTU_128 k__Eukaryota;p__Unclassified;c__Unclassified;o__Unclassified;f__Unclassified;g__Unclassified;s__uncultured fungus 0.0 KX195148.1

OTU_1280 k__Eukaryota;p__Ascomycota;c__Dothideomycetes;o__Dothideales;f__Saccotheciaceae;g__Aureobasidium;s__Aureobasidium namibiae 7e-128 KY472292.1

OTU_1281 k__Eukaryota;p__Ascomycota;c__Sordariomycetes;o__Xylariales;f__Xylariaceae;g__Podosordaria;s__Podosordaria tulasnei 1e-166 KT281902.1

OTU_1283 k__Eukaryota;p__Ascomycota;c__Dothideomycetes;o__Pleosporales;f__Pleosporaceae;g__Unclassified;s__uncultured Neocamarosporium 2e-94 KU245684.1

OTU_1284 k__Eukaryota;p__Ascomycota;c__Saccharomycetes;o__Saccharomycetales;f__Trichomonascaceae;g__Blastobotrys;s__Blastobotrys sp. AB-2017a 4e-89 MG018986.1

OTU_1285 k__Eukaryota;p__Unclassified;c__Unclassified;o__Unclassified;f__Unclassified;g__Unclassified;s__uncultured fungus 1e-163 KX195463.1

OTU_1286 k__Eukaryota;p__Unclassified;c__Unclassified;o__Unclassified;f__Unclassified;g__Unclassified;s__uncultured fungus 4e-151 HM136638.1

OTU_1287 k__Eukaryota;p__Ascomycota;c__Dothideomycetes;o__Botryosphaeriales;f__Botryosphaeriaceae;g__Lasiodiplodia;s__Lasiodiplodia theobromae 3e-170 MG576123.1

OTU_1288 k__Eukaryota;p__Ascomycota;c__Dothideomycetes;o__Pleosporales;f__Pleosporaceae;g__Unclassified;s__uncultured Neocamarosporium 2e-106 KU245684.1

OTU_1289 k__Eukaryota;p__Ascomycota;c__Dothideomycetes;o__Capnodiales;f__Cladosporiaceae;g__Cladosporium;s__Cladosporium cladosporioides 2e-146 MF173069.1

OTU_129 k__Eukaryota;p__Ascomycota;c__Unclassified;o__Unclassified;f__Unclassified;g__Unclassified;s__Ascomycota sp. H-8 5e-169 FJ375144.1

OTU_1290 k__Eukaryota;p__Ascomycota;c__Dothideomycetes;o__Pleosporales;f__Didymellaceae;g__Ascochyta;s__Ascochyta fabae 1e-95 FM180029.1

OTU_1291 k__Eukaryota;p__Unclassified;c__Unclassified;o__Unclassified;f__Unclassified;g__Unclassified;s__uncultured fungus 7e-14 MF570481.1

OTU_1292 k__Eukaryota;p__Ascomycota;c__Sordariomycetes;o__Hypocreales;f__Nectriaceae;g__Fusarium;s__Fusarium nematophilum 2e-177 MG543773.1

OTU_1293 k__Eukaryota;p__Unclassified;c__Unclassified;o__Unclassified;f__Unclassified;g__Unclassified;s__uncultured fungus 9e-146 KF800433.1

OTU_1294 k__Eukaryota;p__Ascomycota;c__Dothideomycetes;o__Botryosphaeriales;f__Aplosporellaceae;g__Aplosporella;s__Aplosporella sp. 2e-171 KX774161.1

OTU_1295 k__Eukaryota;p__Ascomycota;c__Leotiomycetes;o__Helotiales;f__Unclassified;g__Phialocephala;s__Phialocephala fortinii 4e-154 KY910215.1

OTU_1296 k__Eukaryota;p__Unclassified;c__Unclassified;o__Unclassified;f__Unclassified;g__Unclassified;s__uncultured fungus 1e-12 KP889943.1

OTU_1297 k__Eukaryota;p__Ascomycota;c__Sordariomycetes;o__Hypocreales;f__Clavicipitaceae;g__Metarhizium;s__Metarhizium anisopliae 1e-141 JN206646.1

OTU_1298 k__Eukaryota;p__Ascomycota;c__Eurotiomycetes;o__Eurotiales;f__Aspergillaceae;g__Penicillium;s__Penicillium oxalicum 3e-170 MG543697.1

OTU_1299 k__Eukaryota;p__Unclassified;c__Unclassified;o__Unclassified;f__Unclassified;g__Unclassified;s__uncultured fungus 1e-49 FJ362304.1

OTU_13 k__Eukaryota;p__Basidiomycota;c__Tremellomycetes;o__Tremellales;f__Rhynchogastremataceae;g__Papiliotrema;s__Papiliotrema terrestris 1e-178 KY495734.1

OTU_130 k__Eukaryota;p__Unclassified;c__Unclassified;o__Unclassified;f__Unclassified;g__Unclassified;s__uncultured fungus 2e-150 HQ389461.1

OTU_1300 k__Eukaryota;p__Unclassified;c__Unclassified;o__Unclassified;f__Unclassified;g__Unclassified;s__fungal endophyte sp. D3-4A3-1 2e-149 FJ025302.1

OTU_1301 k__Eukaryota;p__Ascomycota;c__Dothideomycetes;o__Pleosporales;f__Didymellaceae;g__Epicoccum;s__Epicoccum nigrum 2e-146 KY977575.1

OTU_1302 k__Eukaryota;p__Unclassified;c__Unclassified;o__Unclassified;f__Unclassified;g__Unclassified;s__uncultured fungus 1e-15 JQ313107.1

OTU_1303 k__Eukaryota;p__Unclassified;c__Unclassified;o__Unclassified;f__Unclassified;g__Unclassified;s__uncultured fungus 4e-157 MF569982.1

OTU_1304 k__Eukaryota;p__Ascomycota;c__Dothideomycetes;o__Pleosporales;f__Pleosporaceae;g__Alternaria;s__Alternaria alternata 8e-146 KR149267.1

OTU_1305 k__Eukaryota;p__Unclassified;c__Unclassified;o__Unclassified;f__Unclassified;g__Unclassified;s__uncultured fungus 2e-180 GQ999397.1

OTU_1306 k__Eukaryota;p__Ascomycota;c__Dothideomycetes;o__Capnodiales;f__Cladosporiaceae;g__Cladosporium;s__Cladosporium sp. 1 BRM-2015 2e-143 KP309884.1

OTU_1307 k__Eukaryota;p__Unclassified;c__Unclassified;o__Unclassified;f__Unclassified;g__Unclassified;s__uncultured fungus 1e-129 KC978070.1

OTU_1308 k__Eukaryota;p__Unclassified;c__Unclassified;o__Unclassified;f__Unclassified;g__Unclassified;s__fungal sp. 2e-134 MF347807.1

OTU_1309 k__Eukaryota;p__Unclassified;c__Unclassified;o__Unclassified;f__Unclassified;g__Unclassified;s__uncultured fungus 7e-128 HG328155.1

OTU_131 k__Eukaryota;p__Ascomycota;c__Saccharomycetes;o__Saccharomycetales;f__Saccharomycopsidaceae;g__Saccharomycopsis;s__Saccharomycopsis vini 9e-180 KY105290.1

OTU_1310 k__Eukaryota;p__Ascomycota;c__Dothideomycetes;o__Unclassified;f__Unclassified;g__Unclassified;s__Dothideomycetes sp. genotype 257 4e-154 KX908765.1

OTU_1311 k__Eukaryota;p__Ascomycota;c__Dothideomycetes;o__Pleosporales;f__Pleosporaceae;g__Alternaria;s__Alternaria tenuissima 1e-141 KF996845.1

OTU_1312 k__Eukaryota;p__Unclassified;c__Unclassified;o__Unclassified;f__Unclassified;g__Unclassified;s__uncultured fungus 3e-145 KT328643.1

OTU_1313 k__Eukaryota;p__Unclassified;c__Unclassified;o__Unclassified;f__Unclassified;g__Unclassified;s__uncultured fungus 4e-52 MF570757.1

OTU_1314 k__Eukaryota;p__Unclassified;c__Unclassified;o__Unclassified;f__Unclassified;g__Unclassified;s__uncultured fungus 3e-127 KC965887.1

OTU_1315 k__Eukaryota;p__Ascomycota;c__Dothideomycetes;o__Pleosporales;f__Didymellaceae;g__Phoma;s__Phoma betae 2e-119 KX239986.1

OTU_1316 k__Eukaryota;p__Unclassified;c__Unclassified;o__Unclassified;f__Unclassified;g__Unclassified;s__uncultured fungus 7e-14 JX043102.1

OTU_1317 k__Eukaryota;p__Unclassified;c__Unclassified;o__Unclassified;f__Unclassified;g__Unclassified;s__uncultured fungus 1e-76 KX193383.1

OTU_1318 k__Eukaryota;p__Ascomycota;c__Leotiomycetes;o__Helotiales;f__Sclerotiniaceae;g__Botrytis;s__Botrytis cinerea 1e-141 KY419551.1

OTU_1319 k__Eukaryota;p__Ascomycota;c__Orbiliomycetes;o__Orbiliales;f__Orbiliaceae;g__Arthrobotrys;s__Arthrobotrys oligospora 0.0 JQ360170.1

OTU_132 k__Eukaryota;p__Unclassified;c__Unclassified;o__Unclassified;f__Unclassified;g__Unclassified;s__uncultured fungus 2e-165 MF569395.1

OTU_1320 k__Eukaryota;p__Unclassified;c__Unclassified;o__Unclassified;f__Unclassified;g__Unclassified;s__uncultured fungus 5e-15 MF571135.1

OTU_1321 k__Eukaryota;p__Ascomycota;c__Dothideomycetes;o__Capnodiales;f__Cladosporiaceae;g__Cladosporium;s__Cladosporium perangustum 1e-141 KJ620970.1

OTU_1322 k__Eukaryota;p__Ascomycota;c__Unclassified;o__Unclassified;f__Unclassified;g__Unclassified;s__uncultured Tetracladium 4e-151 KT735277.1

OTU_1323 k__Eukaryota;p__Ascomycota;c__Dothideomycetes;o__Dothideales;f__Saccotheciaceae;g__Aureobasidium;s__aff. Aureobasidium sp. 3e-170 KT150691.1

OTU_1324 k__Eukaryota;p__Ascomycota;c__Dothideomycetes;o__Pleosporales;f__Pleosporaceae;g__Neocamarosporium;s__Neocamarosporium calvescens 1e-166 KY940773.1

OTU_1325 k__Eukaryota;p__Mucoromycota;c__Unclassified;o__Mucorales;f__Rhizopodaceae;g__Rhizopus;s__Rhizopus microsporus 4e-160 KY606252.1

OTU_1326 k__Eukaryota;p__Ascomycota;c__Dothideomycetes;o__Pleosporales;f__Didymellaceae;g__Didymella;s__Didymella americana 9e-149 KY099740.1

OTU_1327 k__Eukaryota;p__Unclassified;c__Unclassified;o__Unclassified;f__Unclassified;g__Unclassified;s__uncultured fungus 1e-06 MF571135.1

OTU_1328 k__Eukaryota;p__Ascomycota;c__Dothideomycetes;o__Pleosporales;f__Didymellaceae;g__Phoma;s__Phoma betae 1e-79 KC460811.1

OTU_1329 k__Eukaryota;p__Ascomycota;c__Dothideomycetes;o__Pleosporales;f__Pleosporaceae;g__Unclassified;s__uncultured Neocamarosporium 6e-85 KU245684.1

OTU_133 k__Eukaryota;p__Ascomycota;c__Leotiomycetes;o__Helotiales;f__Unclassified;g__Hyalodendriella;s__Hyalodendriella sp. FN-4-S1-1 2e-168 KJ463525.1

OTU_1330 k__Eukaryota;p__Unclassified;c__Unclassified;o__Unclassified;f__Unclassified;g__Unclassified;s__uncultured fungus 5e-73 FJ237229.1

OTU_1331 k__Eukaryota;p__Ascomycota;c__Dothideomycetes;o__Pleosporales;f__Unclassified;g__Unclassified;s__uncultured Pleosporales 2e-171 HQ389500.1

OTU_1332 k__Eukaryota;p__Basidiomycota;c__Agaricomycetes;o__Agaricales;f__Unclassified;g__Unclassified;s__uncultured Agaricales 6e-172 FJ554169.1

OTU_1333 k__Eukaryota;p__Unclassified;c__Unclassified;o__Unclassified;f__Unclassified;g__Unclassified;s__uncultured fungus 7e-63 FJ237229.1

OTU_1334 k__Eukaryota;p__Ascomycota;c__Eurotiomycetes;o__Eurotiales;f__Aspergillaceae;g__Penicillium;s__Penicillium adametzii 8e-171 KP050693.1

OTU_1335 k__Eukaryota;p__Ascomycota;c__Sordariomycetes;o__Xylariales;f__Amphisphaeriaceae;g__Seimatosporium;s__Seimatosporium lichenicola 2e-158 GU244511.1

OTU_1336 k__Eukaryota;p__Ascomycota;c__Dothideomycetes;o__Pleosporales;f__Pleosporaceae;g__Alternaria;s__Alternaria sp. YLN5 5e-144 KC139493.1

OTU_1337 k__Eukaryota;p__Mucoromycota;c__Unclassified;o__Mucorales;f__Mucoraceae;g__Mucor;s__Mucor racemosus 8e-171 KX060585.1

OTU_1338 k__Eukaryota;p__Unclassified;c__Unclassified;o__Unclassified;f__Unclassified;g__Unclassified;s__uncultured fungus 5e-144 GU174335.1

OTU_1339 k__Eukaryota;p__Ascomycota;c__Dothideomycetes;o__Dothideales;f__Saccotheciaceae;g__Aureobasidium;s__Aureobasidium pullulans 2e-119 KY294714.1

OTU_134 k__Eukaryota;p__Basidiomycota;c__Tremellomycetes;o__Tremellales;f__Cryptococcaceae;g__Kwoniella;s__Kwoniella dendrophila 3e-164 JQ993377.1

OTU_1340 k__Eukaryota;p__Unclassified;c__Unclassified;o__Unclassified;f__Unclassified;g__Unclassified;s__uncultured fungus 3e-93 MF569495.1

OTU_1341 k__Eukaryota;p__Unclassified;c__Unclassified;o__Unclassified;f__Unclassified;g__Unclassified;s__uncultured fungus 1e-160 KC884316.1

OTU_1342 k__Eukaryota;p__Unclassified;c__Unclassified;o__Unclassified;f__Unclassified;g__Unclassified;s__uncultured fungus 2e-122 MF569334.1

OTU_1343 k__Eukaryota;p__Ascomycota;c__Saccharomycetes;o__Saccharomycetales;f__Unclassified;g__Candida;s__Candida galis 6e-21 NR_151797.1

OTU_1344 k__Eukaryota;p__Unclassified;c__Unclassified;o__Unclassified;f__Unclassified;g__Unclassified;s__uncultured fungus 3e-90 KT758090.1

OTU_1345 k__Eukaryota;p__Ascomycota;c__Dothideomycetes;o__Pleosporales;f__Didymellaceae;g__Didymella;s__Didymella eucalyptica 9e-149 GU237846.1

OTU_1346 k__Eukaryota;p__Basidiomycota;c__Exobasidiomycetes;o__Microstromatales;f__Quambalariaceae;g__Quambalaria;s__Quambalaria cyanescens 4e-65 KY001602.1

OTU_1347 k__Eukaryota;p__Unclassified;c__Unclassified;o__Unclassified;f__Unclassified;g__Unclassified;s__uncultured fungus 5e-79 KT219279.1

OTU_1348 k__Eukaryota;p__Ascomycota;c__Sordariomycetes;o__Glomerellales;f__Glomerellaceae;g__Colletotrichum;s__Colletotrichum gloeosporioides 3e-68 KX906602.1

OTU_1349 k__Eukaryota;p__Ascomycota;c__Leotiomycetes;o__Helotiales;f__Sclerotiniaceae;g__Sclerotinia;s__Sclerotinia sclerotiorum 4e-126 JX839987.1

OTU_135 k__Eukaryota;p__Ascomycota;c__Dothideomycetes;o__Pleosporales;f__Lophiostomataceae;g__Sigarispora;s__Sigarispora caulium 3e-167 LC001724.1

OTU_1350 k__Eukaryota;p__Basidiomycota;c__Agaricomycetes;o__Polyporales;f__Polyporaceae;g__Trametes;s__Trametes hirsuta 0.0 KX611625.1

OTU_1351 k__Eukaryota;p__Ascomycota;c__Eurotiomycetes;o__Eurotiales;f__Aspergillaceae;g__Penicillium;s__Penicillium sp. QTYC19 2e-109 KM103307.1

OTU_1352 k__Eukaryota;p__Unclassified;c__Unclassified;o__Unclassified;f__Unclassified;g__Unclassified;s__fungal sp. TZ-2013 5e-169 KC131372.1

OTU_1353 k__Eukaryota;p__Ascomycota;c__Dothideomycetes;o__Pleosporales;f__Pleosporaceae;g__Alternaria;s__Alternaria rosae 2e-156 KY472289.1

OTU_1354 k__Eukaryota;p__Unclassified;c__Unclassified;o__Unclassified;f__Unclassified;g__Unclassified;s__uncultured fungus 6e-156 MF568995.1

OTU_1355 k__Eukaryota;p__Unclassified;c__Unclassified;o__Unclassified;f__Unclassified;g__Unclassified;s__uncultured fungus 2e-165 MF570603.1

OTU_1356 k__Eukaryota;p__Ascomycota;c__Unclassified;o__Unclassified;f__Unclassified;g__Unclassified;s__uncultured Leptodontidium 3e-161 KJ188596.1

OTU_1357 k__Eukaryota;p__Ascomycota;c__Dothideomycetes;o__Pleosporales;f__Didymellaceae;g__Phoma;s__Phoma betae 4e-86 KC460811.1

OTU_1358 k__Eukaryota;p__Unclassified;c__Unclassified;o__Unclassified;f__Unclassified;g__Unclassified;s__uncultured fungus 0.0 KX194778.1

OTU_1359 k__Eukaryota;p__Basidiomycota;c__Agaricomycetes;o__Agaricales;f__Psathyrellaceae;g__Unclassified;s__uncultured Psathyrellaceae 0.0 KX115709.1

OTU_136 k__Eukaryota;p__Unclassified;c__Unclassified;o__Unclassified;f__Unclassified;g__Unclassified;s__uncultured fungus 3e-133 MF569277.1

OTU_1360 k__Eukaryota;p__Unclassified;c__Unclassified;o__Unclassified;f__Unclassified;g__Unclassified;s__uncultured fungus 2e-140 MF570373.1

OTU_1361 k__Eukaryota;p__Basidiomycota;c__Agaricomycetes;o__Polyporales;f__Irpicaceae;g__Irpex;s__Irpex lacteus 0.0 KY824786.1

OTU_1362 k__Eukaryota;p__Ascomycota;c__Unclassified;o__Unclassified;f__Unclassified;g__Unclassified;s__mycorrhizal ascomycete of Rhododendron type 3 1e-147 AB089661.1

OTU_1363 k__Eukaryota;p__Unclassified;c__Unclassified;o__Unclassified;f__Unclassified;g__Unclassified;s__uncultured fungus 5e-147 KC765994.1

OTU_1364 k__Eukaryota;p__Unclassified;c__Unclassified;o__Unclassified;f__Unclassified;g__Unclassified;s__fungal sp. L10 5e-135 KF887091.1

OTU_1365 k__Eukaryota;p__Ascomycota;c__Dothideomycetes;o__Capnodiales;f__Unclassified;g__Unclassified;s__uncultured Rachicladosporium 4e-157 KT581654.1

OTU_1366 k__Eukaryota;p__Unclassified;c__Unclassified;o__Unclassified;f__Unclassified;g__Unclassified;s__uncultured fungus 1e-49 FJ362303.1

OTU_1367 k__Eukaryota;p__Ascomycota;c__Dothideomycetes;o__Dothideales;f__Saccotheciaceae;g__Aureobasidium;s__Aureobasidium namibiae 1e-132 KY472292.1

OTU_1368 k__Eukaryota;p__Ascomycota;c__Dothideomycetes;o__Pleosporales;f__Leptosphaeriaceae;g__Leptosphaeria;s__Leptosphaeria biglobosa 1e-49 FO905597.1

OTU_1369 k__Eukaryota;p__Ascomycota;c__Dothideomycetes;o__Pleosporales;f__Phaeosphaeriaceae;g__Chaetosphaeronema;s__Chaetosphaeronema achilleae 2e-100 KX765265.1

OTU_137 k__Eukaryota;p__Unclassified;c__Unclassified;o__Unclassified;f__Unclassified;g__Unclassified;s__fungal sp. 1e-120 KY776236.1

OTU_1370 k__Eukaryota;p__Ascomycota;c__Dothideomycetes;o__Pleosporales;f__Didymellaceae;g__Phoma;s__Phoma betae 2e-88 EU003450.1

OTU_1371 k__Eukaryota;p__Ascomycota;c__Dothideomycetes;o__Pleosporales;f__Pleosporaceae;g__Alternaria;s__Alternaria tenuissima 3e-142 KF996866.1

OTU_1372 k__Eukaryota;p__Ascomycota;c__Dothideomycetes;o__Pleosporales;f__Leptosphaeriaceae;g__Leptosphaeria;s__Leptosphaeria biglobosa 9e-07 FO906080.1

OTU_1373 k__Eukaryota;p__Unclassified;c__Unclassified;o__Unclassified;f__Unclassified;g__Unclassified;s__uncultured fungus 6e-178 KX193034.1

OTU_1374 k__Eukaryota;p__Unclassified;c__Unclassified;o__Unclassified;f__Unclassified;g__Unclassified;s__uncultured fungus 6e-159 KP889806.1

OTU_1375 k__Eukaryota;p__Unclassified;c__Unclassified;o__Unclassified;f__Unclassified;g__Unclassified;s__uncultured fungus 2e-134 AM260861.1

OTU_1376 k__Eukaryota;p__Unclassified;c__Unclassified;o__Unclassified;f__Unclassified;g__Unclassified;s__fungal sp. 8e-146 KX611076.1

OTU_1377 k__Eukaryota;p__Ascomycota;c__Saccharomycetes;o__Saccharomycetales;f__Saccharomycodaceae;g__Hanseniaspora;s__Hanseniaspora thailandica 2e-174 MG493194.1

OTU_1378 k__Eukaryota;p__Basidiomycota;c__Exobasidiomycetes;o__Golubeviales;f__Golubeviaceae;g__Golubevia;s__Golubevia pallescens 0.0 KX034342.1

OTU_1379 k__Eukaryota;p__Ascomycota;c__Sordariomycetes;o__Xylariales;f__Sporocadaceae;g__Pestalotiopsis;s__Pestalotiopsis microspora 8e-174 MF375901.1

OTU_138 k__Eukaryota;p__Ascomycota;c__Dothideomycetes;o__Pleosporales;f__Phaeosphaeriaceae;g__Neosetophoma;s__Neosetophoma italica 5e-135 KP711356.1

OTU_1380 k__Eukaryota;p__Unclassified;c__Unclassified;o__Unclassified;f__Unclassified;g__Unclassified;s__uncultured fungus 6e-08 EU917114.1

OTU_1381 k__Eukaryota;p__Ascomycota;c__Sordariomycetes;o__Unclassified;f__Unclassified;g__Unclassified;s__uncultured Myrmecridium 2e-161 HG936420.1

OTU_1382 k__Eukaryota;p__Ascomycota;c__Dothideomycetes;o__Pleosporales;f__Lentitheciaceae;g__Keissleriella;s__Keissleriella poagena 3e-164 KJ869112.1

OTU_1384 k__Eukaryota;p__Unclassified;c__Unclassified;o__Unclassified;f__Unclassified;g__Unclassified;s__uncultured fungus 7e-08 KX195280.1

OTU_1385 k__Eukaryota;p__Ascomycota;c__Saccharomycetes;o__Saccharomycetales;f__Saccharomycodaceae;g__Hanseniaspora;s__Hanseniaspora uvarum 1e-172 KY103569.1

OTU_1386 k__Eukaryota;p__Ascomycota;c__Dothideomycetes;o__Pleosporales;f__Phaeosphaeriaceae;g__Phaeosphaeria;s__Phaeosphaeria sp. 1e-169 KT269495.1

OTU_1387 k__Eukaryota;p__Ascomycota;c__Dothideomycetes;o__Capnodiales;f__Cladosporiaceae;g__Unclassified;s__uncultured Davidiella 2e-131 HG935294.1

OTU_1388 k__Eukaryota;p__Unclassified;c__Unclassified;o__Unclassified;f__Unclassified;g__Unclassified;s__uncultured fungus 3e-59 FJ362304.1

OTU_1389 k__Eukaryota;p__Ascomycota;c__Dothideomycetes;o__Dothideales;f__Saccotheciaceae;g__Aureobasidium;s__Aureobasidium namibiae 2e-143 KY472292.1

OTU_139 k__Eukaryota;p__Basidiomycota;c__Microbotryomycetes;o__Sporidiobolales;f__Sporidiobolaceae;g__Rhodosporidiobolus;s__Rhodosporidiobolus ruineniae 0.0 KX771223.1

OTU_1390 k__Eukaryota;p__Unclassified;c__Unclassified;o__Unclassified;f__Unclassified;g__Unclassified;s__uncultured fungus 6e-159 MF571283.1

OTU_1391 k__Eukaryota;p__Ascomycota;c__Dothideomycetes;o__Dothideales;f__Saccotheciaceae;g__Aureobasidium;s__Aureobasidium sp. AUMC 7757 1e-166 JQ425384.1

OTU_1392 k__Eukaryota;p__Ascomycota;c__Dothideomycetes;o__Pleosporales;f__Pleosporaceae;g__Neocamarosporium;s__Neocamarosporium goegapense 6e-162 MG065849.1

OTU_1393 k__Eukaryota;p__Ascomycota;c__Dothideomycetes;o__Unclassified;f__Unclassified;g__Unclassified;s__uncultured Dothideomycetes 1e-135 JX010730.1

OTU_1394 k__Eukaryota;p__Unclassified;c__Unclassified;o__Unclassified;f__Unclassified;g__Unclassified;s__uncultured fungus 4e-06 FJ362304.1

OTU_1395 k__Eukaryota;p__Basidiomycota;c__Exobasidiomycetes;o__Golubeviales;f__Golubeviaceae;g__Golubevia;s__Golubevia pallescens 0.0 KX034342.1

OTU_1396 k__Eukaryota;p__Unclassified;c__Unclassified;o__Unclassified;f__Unclassified;g__Unclassified;s__uncultured fungus 6e-98 MF570694.1

OTU_1397 k__Eukaryota;p__Ascomycota;c__Dothideomycetes;o__Pleosporales;f__Pleosporaceae;g__Alternaria;s__Alternaria helianthiinficiens 4e-157 JX101648.1

OTU_1398 k__Eukaryota;p__Ascomycota;c__Dothideomycetes;o__Pleosporales;f__Leptosphaeriaceae;g__Leptosphaeria;s__Leptosphaeria proteicola 8e-140 JQ044439.1

OTU_1399 k__Eukaryota;p__Ascomycota;c__Dothideomycetes;o__Capnodiales;f__Cladosporiaceae;g__Cladosporium;s__Cladosporium sp. 1 BRM-2015 1e-132 KP309884.1

OTU_14 k__Eukaryota;p__Ascomycota;c__Eurotiomycetes;o__Eurotiales;f__Aspergillaceae;g__Aspergillus;s__Aspergillus niger 5e-175 KY288052.1

OTU_140 k__Eukaryota;p__Unclassified;c__Unclassified;o__Unclassified;f__Unclassified;g__Unclassified;s__uncultured fungus 7e-168 JF300545.1

OTU_1400 k__Bacteria;p__Proteobacteria;c__Alphaproteobacteria;o__Rhodospirillales;f__Acetobacteraceae;g__Gluconobacter;s__Gluconobacter oxydans 3e-06 CP016328.1

OTU_1401 k__Eukaryota;p__Ascomycota;c__Dothideomycetes;o__Pleosporales;f__Leptosphaeriaceae;g__Leptosphaeria;s__Leptosphaeria sp. CSE211 2e-165 KT024988.1

OTU_1402 k__Eukaryota;p__Unclassified;c__Unclassified;o__Unclassified;f__Unclassified;g__Unclassified;s__uncultured fungus 7e-57 FJ362304.1

OTU_1403 k__Eukaryota;p__Ascomycota;c__Sordariomycetes;o__Coniochaetales;f__Coniochaetaceae;g__Coniochaeta;s__Lecythophora sp. 1e-157 KT264687.1

OTU_1404 k__Eukaryota;p__Unclassified;c__Unclassified;o__Unclassified;f__Unclassified;g__Unclassified;s__uncultured fungus 6e-85 KX194703.1

OTU_1405 k__Eukaryota;p__Basidiomycota;c__Agaricomycetes;o__Thelephorales;f__Thelephoraceae;g__Unclassified;s__uncultured Tomentella 1e-151 KT948425.1

OTU_1406 k__Eukaryota;p__Unclassified;c__Unclassified;o__Unclassified;f__Unclassified;g__Unclassified;s__fungal sp. CA151RZ 8e-66 KP403992.1

OTU_1407 k__Eukaryota;p__Basidiomycota;c__Tremellomycetes;o__Unclassified;f__Unclassified;g__Cryptococcus;s__Cryptococcus sp. 2 TMS-2011 0.0 HQ631032.1

OTU_1408 k__Eukaryota;p__Ascomycota;c__Leotiomycetes;o__Helotiales;f__Unclassified;g__Unclassified;s__uncultured Chalara 2e-155 HG936248.1

OTU_1409 k__Eukaryota;p__Ascomycota;c__Dothideomycetes;o__Capnodiales;f__Mycosphaerellaceae;g__Mycosphaerella;s__Mycosphaerella sp. NW-AN001 1e-150 JN809911.1

OTU_141 k__Eukaryota;p__Ascomycota;c__Saccharomycetes;o__Saccharomycetales;f__Pichiaceae;g__Pichia;s__Pichia sp. feni 108 5e-144 KP223719.1

OTU_1410 k__Eukaryota;p__Ascomycota;c__Dothideomycetes;o__Dothideales;f__Dothideaceae;g__Endoconidioma;s__Endoconidioma populi 2e-165 KT824245.1

OTU_1411 k__Eukaryota;p__Ascomycota;c__Dothideomycetes;o__Dothideales;f__Saccotheciaceae;g__Aureobasidium;s__Aureobasidium pullulans 1e-147 KY294714.1

OTU_1412 k__Eukaryota;p__Unclassified;c__Unclassified;o__Unclassified;f__Unclassified;g__Unclassified;s__uncultured fungus 6e-156 KX195121.1

OTU_1413 k__Eukaryota;p__Basidiomycota;c__Tremellomycetes;o__Filobasidiales;f__Piskurozymaceae;g__Solicoccozyma;s__Solicoccozyma phenolicus 0.0 KY037851.1

OTU_1414 k__Eukaryota;p__Basidiomycota;c__Agaricomycetes;o__Agaricales;f__Agaricaceae;g__Unclassified;s__uncultured Coprinus 0.0 JX135082.1

OTU_1415 k__Eukaryota;p__Ascomycota;c__Dothideomycetes;o__Pleosporales;f__Phaeosphaeriaceae;g__Nodulosphaeria;s__Nodulosphaeria multiseptata 5e-61 KY496748.1

OTU_1416 k__Eukaryota;p__Basidiomycota;c__Agaricomycetes;o__Agaricales;f__Clavariaceae;g__Clavaria;s__Clavaria falcata 9e-177 KC759447.1

OTU_1417 k__Eukaryota;p__Ascomycota;c__Dothideomycetes;o__Pleosporales;f__Massarinaceae;g__Massarina;s__Massarina sp. JP-2013 2e-10 JX981477.1

OTU_1418 k__Eukaryota;p__Unclassified;c__Unclassified;o__Unclassified;f__Unclassified;g__Unclassified;s__fungal sp. CA151RZ 6e-45 KP403992.1

OTU_1419 k__Eukaryota;p__Ascomycota;c__Dothideomycetes;o__Pleosporales;f__Didymosphaeriaceae;g__Paraphaeosphaeria;s__Paraphaeosphaeria michotii 1e-157 KY228674.1

OTU_142 k__Eukaryota;p__Unclassified;c__Unclassified;o__Unclassified;f__Unclassified;g__Unclassified;s__fungal sp. 4e-166 KY404958.1

OTU_1421 k__Eukaryota;p__Unclassified;c__Unclassified;o__Unclassified;f__Unclassified;g__Unclassified;s__uncultured fungus 0.0 FN397399.1

OTU_1423 k__Eukaryota;p__Unclassified;c__Unclassified;o__Unclassified;f__Unclassified;g__Unclassified;s__uncultured fungus 1e-120 KU061787.1

OTU_1424 k__Eukaryota;p__Unclassified;c__Unclassified;o__Unclassified;f__Unclassified;g__Unclassified;s__uncultured fungus 8e-11 MF570389.1

OTU_1425 k__Eukaryota;p__Ascomycota;c__Sordariomycetes;o__Hypocreales;f__Nectriaceae;g__Fusarium;s__Fusarium napiforme 6e-162 MG515227.1

OTU_1426 k__Eukaryota;p__Basidiomycota;c__Tremellomycetes;o__Trichosporonales;f__Trichosporonaceae;g__Cryptococcus;s__Cryptococcus sp. CMT8 5e-172 JQ754007.1

OTU_1427 k__Eukaryota;p__Unclassified;c__Unclassified;o__Unclassified;f__Unclassified;g__Unclassified;s__uncultured fungus 1e-135 MF569386.1

OTU_1428 k__Eukaryota;p__Basidiomycota;c__Exobasidiomycetes;o__Robbauerales;f__Robbaueraceae;g__Robbauera;s__Robbauera albescens 0.0 NR_138401.1

OTU_1429 k__Eukaryota;p__Ascomycota;c__Dothideomycetes;o__Pleosporales;f__Phaeosphaeriaceae;g__Chaetosphaeronema;s__Chaetosphaeronema sp. 2e-159 KX774114.1

OTU_143 k__Eukaryota;p__Basidiomycota;c__Tremellomycetes;o__Tremellales;f__Bulleribasidiaceae;g__Vishniacozyma;s__Vishniacozyma dimennae 2e-134 KM246197.1

OTU_1430 k__Eukaryota;p__Ascomycota;c__Dothideomycetes;o__Pleosporales;f__Didymellaceae;g__Phoma;s__Phoma betae 4e-117 KC460811.1

OTU_1431 k__Eukaryota;p__Unclassified;c__Unclassified;o__Unclassified;f__Unclassified;g__Unclassified;s__uncultured fungus 5e-172 MF569833.1

OTU_1432 k__Eukaryota;p__Basidiomycota;c__Agaricomycetes;o__Agaricales;f__Agaricaceae;g__Coprinus;s__Coprinus sterquilinus 0.0 AF345821.1

OTU_1434 k__Eukaryota;p__Unclassified;c__Unclassified;o__Unclassified;f__Unclassified;g__Unclassified;s__fungal endophyte 4e-83 KF673692.1

OTU_1435 k__Eukaryota;p__Ascomycota;c__Dothideomycetes;o__Pleosporales;f__Pleosporaceae;g__Unclassified;s__uncultured Neocamarosporium 1e-89 KU245684.1

OTU_1436 k__Eukaryota;p__Unclassified;c__Unclassified;o__Unclassified;f__Unclassified;g__Unclassified;s__uncultured fungus 2e-41 FJ362303.1

OTU_1437 k__Eukaryota;p__Unclassified;c__Unclassified;o__Unclassified;f__Unclassified;g__Unclassified;s__uncultured fungus 9e-155 KC966048.1

OTU_1438 k__Eukaryota;p__Unclassified;c__Unclassified;o__Unclassified;f__Unclassified;g__Unclassified;s__uncultured fungus 4e-37 MF570568.1

OTU_1439 k__Eukaryota;p__Ascomycota;c__Pezizomycetes;o__Pezizales;f__Pezizaceae;g__Unclassified;s__uncultured Pezizaceae 5e-49 KR819048.1

OTU_144 k__Eukaryota;p__Ascomycota;c__Sordariomycetes;o__Hypocreales;f__Nectriaceae;g__Fusarium;s__Fusarium lateritium 7e-171 MG572381.1

OTU_1440 k__Eukaryota;p__Unclassified;c__Unclassified;o__Unclassified;f__Unclassified;g__Unclassified;s__uncultured fungus 2e-103 EF505561.1

OTU_1441 k__Eukaryota;p__Basidiomycota;c__Agaricomycetes;o__Agaricales;f__Inocybaceae;g__Inocybe;s__Inocybe soluta 0.0 KX897420.1

OTU_1442 k__Eukaryota;p__Basidiomycota;c__Tremellomycetes;o__Filobasidiales;f__Filobasidiaceae;g__Naganishia;s__Naganishia liquefaciens 0.0 FJ515202.1

OTU_1443 k__Eukaryota;p__Ascomycota;c__Dothideomycetes;o__Pleosporales;f__Phaeosphaeriaceae;g__Phaeosphaeria;s__Phaeosphaeria podocarpi 1e-138 NR_137933.1

OTU_1444 k__Eukaryota;p__Ascomycota;c__Dothideomycetes;o__Capnodiales;f__Mycosphaerellaceae;g__Lecanosticta;s__Lecanosticta gloeospora 2e-07 KU948431.1

OTU_1445 k__Eukaryota;p__Unclassified;c__Unclassified;o__Unclassified;f__Unclassified;g__Unclassified;s__uncultured fungus 0.0 HQ021892.1

OTU_1446 k__Eukaryota;p__Basidiomycota;c__Cystobasidiomycetes;o__Cystobasidiales;f__Cystobasidiaceae;g__Cystobasidium;s__Cystobasidium sp. 0.0 MG250437.1

OTU_1447 k__Eukaryota;p__Unclassified;c__Unclassified;o__Unclassified;f__Unclassified;g__Unclassified;s__uncultured fungus 2e-119 KT195261.1

OTU_1448 k__Eukaryota;p__Ascomycota;c__Eurotiomycetes;o__Eurotiales;f__Aspergillaceae;g__Aspergillus;s__Aspergillus niger 1e-144 KY288052.1

OTU_1449 k__Eukaryota;p__Unclassified;c__Unclassified;o__Unclassified;f__Unclassified;g__Unclassified;s__uncultured fungus 0.0 KX194009.1

OTU_145 k__Eukaryota;p__Basidiomycota;c__Tremellomycetes;o__Tremellales;f__Bulleribasidiaceae;g__Vishniacozyma;s__Vishniacozyma carnescens 2e-134 MG250423.1

OTU_1450 k__Eukaryota;p__Unclassified;c__Unclassified;o__Unclassified;f__Unclassified;g__Unclassified;s__uncultured fungus 1e-157 KF800110.1

OTU_1451 k__Eukaryota;p__Ascomycota;c__Eurotiomycetes;o__Eurotiales;f__Aspergillaceae;g__Penicillium;s__Penicillium brevicompactum 1e-154 KY929273.1

OTU_1452 k__Eukaryota;p__Ascomycota;c__Eurotiomycetes;o__Eurotiales;f__Aspergillaceae;g__Aspergillus;s__Aspergillus welwitschiae 3e-136 MG576117.1

OTU_1453 k__Eukaryota;p__Ascomycota;c__Dothideomycetes;o__Pleosporales;f__Phaeosphaeriaceae;g__Phaeosphaeria;s__Phaeosphaeria sp. 2e-152 KT269529.1

OTU_1454 k__Eukaryota;p__Unclassified;c__Unclassified;o__Unclassified;f__Unclassified;g__Unclassified;s__uncultured fungus 6e-150 EF505161.1

OTU_1455 k__Eukaryota;p__Unclassified;c__Unclassified;o__Unclassified;f__Unclassified;g__Unclassified;s__uncultured fungus 9e-171 KP897858.1

OTU_1456 k__Eukaryota;p__Unclassified;c__Unclassified;o__Unclassified;f__Unclassified;g__Unclassified;s__uncultured fungus 5e-73 FJ237229.1

OTU_1457 k__Eukaryota;p__Ascomycota;c__Dothideomycetes;o__Pleosporales;f__Didymellaceae;g__Phoma;s__Phoma betae 4e-86 KC460811.1

OTU_1458 k__Eukaryota;p__Ascomycota;c__Dothideomycetes;o__Pleosporales;f__Leptosphaeriaceae;g__Leptosphaeria;s__Leptosphaeria biglobosa 9e-07 FO906089.1

OTU_1459 k__Eukaryota;p__Ascomycota;c__Dothideomycetes;o__Pleosporales;f__Leptosphaeriaceae;g__Leptosphaeria;s__Leptosphaeria biglobosa 9e-07 FO905597.1

OTU_1460 k__Eukaryota;p__Unclassified;c__Unclassified;o__Unclassified;f__Unclassified;g__Unclassified;s__uncultured fungus 3e-111 KP828191.1

OTU_1461 k__Eukaryota;p__Ascomycota;c__Dothideomycetes;o__Pleosporales;f__Leptosphaeriaceae;g__Leptosphaeria;s__Leptosphaeria biglobosa 9e-07 FO906089.1

OTU_1462 k__Eukaryota;p__Basidiomycota;c__Cystobasidiomycetes;o__Unclassified;f__Unclassified;g__Buckleyzyma;s__Buckleyzyma armeniaca 2e-178 KY101783.1

OTU_1463 k__Eukaryota;p__Unclassified;c__Unclassified;o__Unclassified;f__Unclassified;g__Unclassified;s__uncultured fungus 3e-145 KP897899.1

OTU_1464 k__Eukaryota;p__Ascomycota;c__Sordariomycetes;o__Hypocreales;f__Nectriaceae;g__Fusarium;s__Fusarium biseptatum 1e-172 MG543699.1

OTU_1465 k__Eukaryota;p__Unclassified;c__Unclassified;o__Unclassified;f__Unclassified;g__Unclassified;s__uncultured fungus 2e-134 KF823610.1

OTU_1466 k__Eukaryota;p__Unclassified;c__Unclassified;o__Unclassified;f__Unclassified;g__Unclassified;s__fungal sp. CCFEE 5323 7e-168 FJ392866.1

OTU_1467 k__Eukaryota;p__Unclassified;c__Unclassified;o__Unclassified;f__Unclassified;g__Unclassified;s__uncultured fungus 2e-54 KX193907.1

OTU_1468 k__Eukaryota;p__Ascomycota;c__Dothideomycetes;o__Pleosporales;f__Phaeosphaeriaceae;g__Paraphoma;s__Paraphoma chrysanthemicola 6e-156 KX823408.1

OTU_1469 k__Eukaryota;p__Ascomycota;c__Dothideomycetes;o__Pleosporales;f__Leptosphaeriaceae;g__Leptosphaeria;s__Leptosphaeria biglobosa 2e-07 FO906089.1

OTU_147 k__Eukaryota;p__Ascomycota;c__Sordariomycetes;o__Sordariales;f__Chaetomiaceae;g__Chaetomium;s__Chaetomium angulare 2e-168 KP336763.1

OTU_1470 k__Eukaryota;p__Unclassified;c__Unclassified;o__Unclassified;f__Unclassified;g__Unclassified;s__uncultured fungus 7e-122 KX194778.1

OTU_1471 k__Eukaryota;p__Ascomycota;c__Dothideomycetes;o__Pleosporales;f__Leptosphaeriaceae;g__Leptosphaeria;s__Leptosphaeria biglobosa 9e-07 FO906079.1

OTU_1472 k__Eukaryota;p__Basidiomycota;c__Agaricomycetes;o__Agaricales;f__Psathyrellaceae;g__Coprinopsis;s__Coprinopsis cinerea 0.0 MF919357.1

OTU_1473 k__Eukaryota;p__Unclassified;c__Unclassified;o__Unclassified;f__Unclassified;g__Unclassified;s__fungal sp. NIOCC Y9 7e-162 FJ357790.1

OTU_1474 k__Eukaryota;p__Ascomycota;c__Sordariomycetes;o__Sordariales;f__Chaetomiaceae;g__Chaetomium;s__Chaetomium sp. 5e-169 MG493192.1

OTU_1475 k__Eukaryota;p__Unclassified;c__Unclassified;o__Unclassified;f__Unclassified;g__Unclassified;s__uncultured fungus 9e-07 MF571277.1

OTU_1476 k__Eukaryota;p__Unclassified;c__Unclassified;o__Unclassified;f__Unclassified;g__Unclassified;s__uncultured fungus 5e-144 JX043099.1

OTU_1477 k__Eukaryota;p__Unclassified;c__Unclassified;o__Unclassified;f__Unclassified;g__Unclassified;s__uncultured fungus 1e-49 FJ362304.1

OTU_1478 k__Eukaryota;p__Ascomycota;c__Sordariomycetes;o__Glomerellales;f__Glomerellaceae;g__Colletotrichum;s__Colletotrichum gloeosporioides 2e-143 KX906602.1

OTU_1479 k__Eukaryota;p__Unclassified;c__Unclassified;o__Unclassified;f__Unclassified;g__Unclassified;s__uncultured fungus 0.0 JX990048.1

OTU_148 k__Eukaryota;p__Unclassified;c__Unclassified;o__Unclassified;f__Unclassified;g__Unclassified;s__uncultured fungus 5e-169 JX984777.1

OTU_1480 k__Eukaryota;p__Unclassified;c__Unclassified;o__Unclassified;f__Unclassified;g__Unclassified;s__uncultured fungus 5e-73 FJ237229.1

OTU_1481 k__Eukaryota;p__Basidiomycota;c__Agaricomycetes;o__Thelephorales;f__Thelephoraceae;g__Unclassified;s__uncultured Thelephoraceae 5e-154 KM403041.1

OTU_1482 k__Eukaryota;p__Ascomycota;c__Leotiomycetes;o__Helotiales;f__Sclerotiniaceae;g__Botrytis;s__Botrytis cinerea 5e-135 KY419551.1

OTU_1483 k__Eukaryota;p__Basidiomycota;c__Unclassified;o__Unclassified;f__Unclassified;g__Unclassified;s__uncultured Basidiomycota 0.0 KJ194443.1

OTU_1484 k__Eukaryota;p__Ascomycota;c__Dothideomycetes;o__Pleosporales;f__Didymellaceae;g__Phoma;s__Phoma betae 1e-126 KC460811.1

OTU_1485 k__Eukaryota;p__Unclassified;c__Unclassified;o__Unclassified;f__Unclassified;g__Unclassified;s__uncultured fungus 3e-40 FJ362304.1

OTU_1486 k__Eukaryota;p__Ascomycota;c__Leotiomycetes;o__Helotiales;f__Sclerotiniaceae;g__Monilinia;s__Monilinia seaverii 3e-142 KX982705.1

OTU_1487 k__Eukaryota;p__Ascomycota;c__Dothideomycetes;o__Pleosporales;f__Leptosphaeriaceae;g__Leptosphaeria;s__Leptosphaeria biglobosa 4e-09 FO905431.1

OTU_1488 k__Eukaryota;p__Ascomycota;c__Dothideomycetes;o__Capnodiales;f__Cladosporiaceae;g__Cladosporium;s__Cladosporium sp. SC7b 1e-138 KT948378.1

OTU_1489 k__Eukaryota;p__Ascomycota;c__Dothideomycetes;o__Dothideales;f__Dothioraceae;g__Unclassified;s__uncultured Aureobasidium 1e-141 JQ951580.1

OTU_149 k__Eukaryota;p__Ascomycota;c__Leotiomycetes;o__Helotiales;f__Unclassified;g__Unclassified;s__Helotiales sp. MUT 5177 4e-163 KT699138.1

OTU_1490 k__Eukaryota;p__Ascomycota;c__Dothideomycetes;o__Pleosporales;f__Pleosporaceae;g__Unclassified;s__uncultured Neocamarosporium 1e-82 KU245684.1

OTU_1491 k__Eukaryota;p__Unclassified;c__Unclassified;o__Unclassified;f__Unclassified;g__Unclassified;s__uncultured fungus 6e-79 KX193383.1

OTU_1492 k__Eukaryota;p__Ascomycota;c__Orbiliomycetes;o__Orbiliales;f__Orbiliaceae;g__Brachyphoris;s__Brachyphoris oviparasitica 0.0 JX403725.1

OTU_1493 k__Eukaryota;p__Ascomycota;c__Dothideomycetes;o__Capnodiales;f__Mycosphaerellaceae;g__Phaeophleospora;s__Phaeophleospora eugeniae 1e-79 KF901615.1

OTU_1494 k__Eukaryota;p__Ascomycota;c__Dothideomycetes;o__Pleosporales;f__Phaeosphaeriaceae;g__Unclassified;s__uncultured Phaeosphaeria 2e-165 HG936185.1

OTU_1495 k__Eukaryota;p__Ascomycota;c__Sordariomycetes;o__Hypocreales;f__Cordycipitaceae;g__Cordyceps;s__Cordyceps bifusispora 1e-169 AY245627.1

OTU_1496 k__Eukaryota;p__Unclassified;c__Unclassified;o__Unclassified;f__Unclassified;g__Unclassified;s__fungal sp. mh2223.6 3e-164 GQ996168.1

OTU_1497 k__Eukaryota;p__Ascomycota;c__Saccharomycetes;o__Saccharomycetales;f__Saccharomycodaceae;g__Hanseniaspora;s__Hanseniaspora uvarum 4e-179 MG020690.1

OTU_1498 k__Eukaryota;p__Ascomycota;c__Dothideomycetes;o__Capnodiales;f__Cladosporiaceae;g__Cladosporium;s__Cladosporium cladosporioides 2e-121 KY929278.1

OTU_1499 k__Eukaryota;p__Unclassified;c__Unclassified;o__Unclassified;f__Unclassified;g__Unclassified;s__uncultured fungus 3e-07 KX195773.1

OTU_15 k__Eukaryota;p__Ascomycota;c__Dothideomycetes;o__Pleosporales;f__Didymellaceae;g__Phoma;s__Phoma sp. 7e-168 MG385090.1

OTU_150 k__Eukaryota;p__Ascomycota;c__Leotiomycetes;o__Unclassified;f__Pseudeurotiaceae;g__Pseudogymnoascus;s__Pseudogymnoascus roseus 6e-150 JF320815.1

OTU_1500 k__Eukaryota;p__Ascomycota;c__Dothideomycetes;o__Pleosporales;f__Phaeosphaeriaceae;g__Setomelanomma;s__Setomelanomma sp. 4e-163 MF120201.1

OTU_1501 k__Eukaryota;p__Unclassified;c__Unclassified;o__Unclassified;f__Unclassified;g__Unclassified;s__uncultured fungus 2e-113 MF570922.1

OTU_1502 k__Eukaryota;p__Unclassified;c__Unclassified;o__Unclassified;f__Unclassified;g__Unclassified;s__uncultured fungus 1e-116 KX515855.1

OTU_1503 k__Eukaryota;p__Basidiomycota;c__Unclassified;o__Unclassified;f__Unclassified;g__Unclassified;s__uncultured Agaricomycotina 0.0 FJ554447.1

OTU_1504 k__Eukaryota;p__Ascomycota;c__Eurotiomycetes;o__Onygenales;f__Onygenaceae;g__Auxarthron;s__Auxarthron kuehnii 8e-137 HQ380777.1

OTU_1505 k__Eukaryota;p__Ascomycota;c__Dothideomycetes;o__Dothideales;f__Saccotheciaceae;g__Aureobasidium;s__Aureobasidium pullulans 2e-137 KY294714.1

OTU_1506 k__Eukaryota;p__Unclassified;c__Unclassified;o__Unclassified;f__Unclassified;g__Unclassified;s__uncultured fungus 2e-50 MF570389.1

OTU_1507 k__Eukaryota;p__Unclassified;c__Unclassified;o__Unclassified;f__Unclassified;g__Unclassified;s__uncultured fungus 3e-161 DQ421212.1

OTU_1508 k__Eukaryota;p__Unclassified;c__Unclassified;o__Unclassified;f__Unclassified;g__Unclassified;s__uncultured fungus 4e-108 MF570839.1

OTU_1509 k__Eukaryota;p__Ascomycota;c__Sordariomycetes;o__Hypocreales;f__Ophiocordycipitaceae;g__Ophiocordyceps;s__Ophiocordyceps heteropoda 7e-168 FJ765028.1

OTU_151 k__Eukaryota;p__Ascomycota;c__Saccharomycetes;o__Saccharomycetales;f__Debaryomycetaceae;g__Candida;s__Candida tropicalis 6e-159 KX664666.1

OTU_1510 k__Eukaryota;p__Unclassified;c__Unclassified;o__Unclassified;f__Unclassified;g__Unclassified;s__uncultured fungus 3e-112 KF800524.1

OTU_1511 k__Eukaryota;p__Basidiomycota;c__Agaricomycetes;o__Agaricales;f__Tricholomataceae;g__Clitocybe;s__Clitocybe vibecina 0.0 KM453733.1

OTU_1512 k__Eukaryota;p__Ascomycota;c__Leotiomycetes;o__Erysiphales;f__Erysiphaceae;g__Golovinomyces;s__Golovinomyces orontii 7e-171 KY319040.1

OTU_1513 k__Eukaryota;p__Unclassified;c__Unclassified;o__Unclassified;f__Unclassified;g__Unclassified;s__uncultured fungus 6e-128 KX193788.1

OTU_1514 k__Eukaryota;p__Basidiomycota;c__Tremellomycetes;o__Tremellales;f__Unclassified;g__Unclassified;s__Tremellales sp. LM47 3e-155 EF060429.1

OTU_1515 k__Eukaryota;p__Unclassified;c__Unclassified;o__Unclassified;f__Unclassified;g__Unclassified;s__fungal sp. AM2013 3e-161 KC506197.1

OTU_1516 k__Eukaryota;p__Ascomycota;c__Leotiomycetes;o__Helotiales;f__Hyaloscyphaceae;g__Cistella;s__Cistella albidolutea 1e-126 JN033429.1

OTU_1517 k__Eukaryota;p__Ascomycota;c__Sordariomycetes;o__Glomerellales;f__Glomerellaceae;g__Colletotrichum;s__Colletotrichum gloeosporioides 4e-126 KX906602.1

OTU_1518 k__Eukaryota;p__Unclassified;c__Unclassified;o__Unclassified;f__Unclassified;g__Unclassified;s__uncultured fungus 1e-160 KX192740.1

OTU_1519 k__Eukaryota;p__Ascomycota;c__Dothideomycetes;o__Pleosporales;f__Sporormiaceae;g__Preussia;s__Preussia australis 2e-174 MG025882.1

OTU_152 k__Eukaryota;p__Ascomycota;c__Dothideomycetes;o__Pleosporales;f__Phaeosphaeriaceae;g__Sclerostagonospora;s__Sclerostagonospora sp. 2e-168 KX774087.1

OTU_1520 k__Eukaryota;p__Ascomycota;c__Dothideomycetes;o__Dothideales;f__Saccotheciaceae;g__Aureobasidium;s__Aureobasidium namibiae 3e-127 KY472292.1

OTU_1521 k__Eukaryota;p__Unclassified;c__Unclassified;o__Unclassified;f__Unclassified;g__Unclassified;s__uncultured fungus 1e-46 MF569495.1

OTU_1522 k__Eukaryota;p__Ascomycota;c__Eurotiomycetes;o__Chaetothyriales;f__Chaetothyriaceae;g__Unclassified;s__uncultured Knufia 3e-173 KM062079.1

OTU_1523 k__Eukaryota;p__Unclassified;c__Unclassified;o__Unclassified;f__Unclassified;g__Unclassified;s__uncultured fungus 2e-155 KX195343.1

OTU_1524 k__Eukaryota;p__Unclassified;c__Unclassified;o__Unclassified;f__Unclassified;g__Unclassified;s__uncultured fungus 8e-177 KX193300.1

OTU_1525 k__Eukaryota;p__Mucoromycota;c__Glomeromycetes;o__Glomerales;f__Glomeraceae;g__Glomus;s__Glomus sp. 0518 4e-154 AY174699.1

OTU_1526 k__Eukaryota;p__Unclassified;c__Unclassified;o__Unclassified;f__Unclassified;g__Unclassified;s__uncultured fungus 3e-74 KX194094.1

OTU_1527 k__Eukaryota;p__Unclassified;c__Unclassified;o__Unclassified;f__Unclassified;g__Unclassified;s__uncultured fungus 6e-125 KP891596.1

OTU_1528 k__Eukaryota;p__Unclassified;c__Unclassified;o__Unclassified;f__Unclassified;g__Unclassified;s__uncultured fungus 7e-162 MF569824.1

OTU_1529 k__Eukaryota;p__Ascomycota;c__Dothideomycetes;o__Dothideales;f__Saccotheciaceae;g__Aureobasidium;s__Aureobasidium sp. JSKim-2015 1e-141 LC018816.1

OTU_153 k__Eukaryota;p__Ascomycota;c__Dothideomycetes;o__Pleosporales;f__Sporormiaceae;g__Preussia;s__Preussia sp. 4e-166 KX774110.1

OTU_1530 k__Eukaryota;p__Unclassified;c__Unclassified;o__Unclassified;f__Unclassified;g__Unclassified;s__uncultured fungus 3e-16 MF571123.1

OTU_1531 k__Eukaryota;p__Ascomycota;c__Dothideomycetes;o__Pleosporales;f__Phaeosphaeriaceae;g__Neosetophoma;s__Neosetophoma italica 2e-106 KP711356.1

OTU_1532 k__Eukaryota;p__Unclassified;c__Unclassified;o__Unclassified;f__Unclassified;g__Unclassified;s__uncultured fungus 2e-73 MF570613.1

OTU_1533 k__Eukaryota;p__Ascomycota;c__Dothideomycetes;o__Pleosporales;f__Leptosphaeriaceae;g__Leptosphaeria;s__Leptosphaeria biglobosa 9e-07 FO906079.1

OTU_1534 k__Eukaryota;p__Unclassified;c__Unclassified;o__Unclassified;f__Unclassified;g__Unclassified;s__uncultured fungus 2e-63 FJ237229.1

OTU_1535 k__Eukaryota;p__Ascomycota;c__Dothideomycetes;o__Dothideales;f__Saccotheciaceae;g__Aureobasidium;s__aff. Aureobasidium sp. 5e-129 KT150707.1

OTU_1536 k__Eukaryota;p__Basidiomycota;c__Microbotryomycetes;o__Sporidiobolales;f__Sporidiobolaceae;g__Rhodotorula;s__Rhodotorula glutinis 6e-144 KY611826.1

OTU_1537 k__Eukaryota;p__Basidiomycota;c__Microbotryomycetes;o__Sporidiobolales;f__Sporidiobolaceae;g__Rhodotorula;s__Rhodotorula glutinis 0.0 KY104784.1

OTU_1538 k__Eukaryota;p__Unclassified;c__Unclassified;o__Unclassified;f__Unclassified;g__Unclassified;s__uncultured fungus 6e-98 AY704739.1

OTU_1539 k__Eukaryota;p__Ascomycota;c__Leotiomycetes;o__Helotiales;f__Sclerotiniaceae;g__Botrytis;s__Botrytis cinerea 2e-128 KY419551.1

OTU_154 k__Eukaryota;p__Mucoromycota;c__Unclassified;o__Mucorales;f__Rhizopodaceae;g__Rhizopus;s__Rhizopus sp. 0.0 MG517549.1

OTU_1540 k__Eukaryota;p__Unclassified;c__Unclassified;o__Unclassified;f__Unclassified;g__Unclassified;s__uncultured fungus 1e-52 FJ362304.1

OTU_1541 k__Eukaryota;p__Unclassified;c__Unclassified;o__Unclassified;f__Unclassified;g__Unclassified;s__uncultured fungus 4e-06 FJ362304.1

OTU_1542 k__Eukaryota;p__Ascomycota;c__Dothideomycetes;o__Pleosporales;f__Didymellaceae;g__Phoma;s__Phoma betae 5e-141 KC460811.1

OTU_1543 k__Eukaryota;p__Ascomycota;c__Dothideomycetes;o__Pleosporales;f__Didymellaceae;g__Phoma;s__Phoma betae 4e-129 KC460811.1

OTU_1544 k__Eukaryota;p__Unclassified;c__Unclassified;o__Unclassified;f__Unclassified;g__Unclassified;s__uncultured fungus 1e-92 MF569420.1

OTU_1545 k__Eukaryota;p__Unclassified;c__Unclassified;o__Unclassified;f__Unclassified;g__Unclassified;s__uncultured fungus 6e-48 FJ362304.1

OTU_1546 k__Eukaryota;p__Ascomycota;c__Dothideomycetes;o__Dothideales;f__Saccotheciaceae;g__Aureobasidium;s__Aureobasidium pullulans 3e-127 KX249734.1

OTU_1547 k__Eukaryota;p__Ascomycota;c__Dothideomycetes;o__Pleosporales;f__Sporormiaceae;g__Sporormiella;s__Sporormiella septenaria 3e-167 MG250465.1

OTU_1548 k__Eukaryota;p__Ascomycota;c__Dothideomycetes;o__Pleosporales;f__Pleosporaceae;g__Unclassified;s__uncultured Neocamarosporium 1e-110 KU245684.1

OTU_1549 k__Eukaryota;p__Ascomycota;c__Dothideomycetes;o__Capnodiales;f__Cladosporiaceae;g__Cladosporium;s__Cladosporium sp. BF72 2e-128 AM901688.1

OTU_155 k__Eukaryota;p__Unclassified;c__Unclassified;o__Unclassified;f__Unclassified;g__Unclassified;s__uncultured fungus 0.0 KX514839.1

OTU_1550 k__Eukaryota;p__Ascomycota;c__Unclassified;o__Unclassified;f__Unclassified;g__Unclassified;s__uncultured Ascomycota 1e-166 KY430483.1

OTU_1551 k__Eukaryota;p__Ascomycota;c__Eurotiomycetes;o__Chaetothyriales;f__Herpotrichiellaceae;g__Unclassified;s__uncultured Herpotrichiellaceae 6e-150 KX116032.1

OTU_1552 k__Eukaryota;p__Ascomycota;c__Sordariomycetes;o__Hypocreales;f__Stachybotryaceae;g__Myrothecium;s__Myrothecium gramineum 3e-176 KJ780796.1

OTU_1553 k__Eukaryota;p__Unclassified;c__Unclassified;o__Unclassified;f__Unclassified;g__Unclassified;s__uncultured fungus 1e-150 AJ875373.1

OTU_1554 k__Eukaryota;p__Ascomycota;c__Dothideomycetes;o__Pleosporales;f__Pleosporaceae;g__Alternaria;s__Alternaria sp. 3e-133 KX110402.1

OTU_1555 k__Eukaryota;p__Unclassified;c__Unclassified;o__Unclassified;f__Unclassified;g__Unclassified;s__uncultured fungus 7e-165 KX193161.1

OTU_1556 k__Eukaryota;p__Ascomycota;c__Dothideomycetes;o__Pleosporales;f__Leptosphaeriaceae;g__Leptosphaeria;s__Leptosphaeria biglobosa 9e-07 FO906080.1

OTU_1557 k__Eukaryota;p__Unclassified;c__Unclassified;o__Unclassified;f__Unclassified;g__Unclassified;s__uncultured fungus 3e-164 JX984726.1

OTU_1558 k__Eukaryota;p__Ascomycota;c__Unclassified;o__Unclassified;f__Unclassified;g__Unclassified;s__uncultured Ascomycota 2e-134 AM901946.1

OTU_1559 k__Eukaryota;p__Ascomycota;c__Dothideomycetes;o__Pleosporales;f__Leptosphaeriaceae;g__Leptosphaeria;s__Leptosphaeria biglobosa 9e-07 FO906994.1

OTU_156 k__Eukaryota;p__Ascomycota;c__Unclassified;o__Unclassified;f__Unclassified;g__Unclassified;s__uncultured Ascomycota 2e-162 HQ433030.1

OTU_1560 k__Eukaryota;p__Unclassified;c__Unclassified;o__Unclassified;f__Unclassified;g__Unclassified;s__uncultured fungus 4e-129 MF570775.1

OTU_1561 k__Eukaryota;p__Ascomycota;c__Dothideomycetes;o__Pleosporales;f__Didymellaceae;g__Phoma;s__Phoma betae 2e-100 EU003450.1

OTU_1562 k__Eukaryota;p__Unclassified;c__Unclassified;o__Unclassified;f__Unclassified;g__Unclassified;s__uncultured fungus 3e-161 KX194299.1

OTU_1563 k__Eukaryota;p__Unclassified;c__Unclassified;o__Unclassified;f__Unclassified;g__Unclassified;s__uncultured fungus 4e-102 MF569495.1

OTU_1564 k__Eukaryota;p__Ascomycota;c__Eurotiomycetes;o__Eurotiales;f__Aspergillaceae;g__Aspergillus;s__Aspergillus aculeatinus 2e-146 MG543743.1

OTU_1565 k__Eukaryota;p__Ascomycota;c__Dothideomycetes;o__Dothideales;f__Unclassified;g__Hortaea;s__Hortaea werneckii 4e-166 KX427195.1

OTU_1566 k__Eukaryota;p__Basidiomycota;c__Microbotryomycetes;o__Sporidiobolales;f__Sporidiobolaceae;g__Rhodotorula;s__Rhodotorula glutinis 0.0 KY104784.1

OTU_1567 k__Eukaryota;p__Unclassified;c__Unclassified;o__Unclassified;f__Unclassified;g__Unclassified;s__uncultured fungus 8e-140 FN397333.1

OTU_1568 k__Eukaryota;p__Unclassified;c__Unclassified;o__Unclassified;f__Unclassified;g__Unclassified;s__fungal endophyte 8e-174 KP335567.1

OTU_157 k__Eukaryota;p__Basidiomycota;c__Agaricomycetes;o__Geastrales;f__Geastraceae;g__Geastrum;s__Geastrum papinuttii 8e-171 KP687515.1

OTU_1570 k__Eukaryota;p__Unclassified;c__Unclassified;o__Unclassified;f__Unclassified;g__Unclassified;s__uncultured fungus 3e-164 MF569274.1

OTU_1571 k__Eukaryota;p__Basidiomycota;c__Microbotryomycetes;o__Sporidiobolales;f__Sporidiobolaceae;g__Rhodotorula;s__Rhodotorula glutinis 6e-172 KY611826.1

OTU_1572 k__Eukaryota;p__Unclassified;c__Unclassified;o__Unclassified;f__Unclassified;g__Unclassified;s__uncultured fungus 6e-156 KT965072.1

OTU_1573 k__Eukaryota;p__Ascomycota;c__Unclassified;o__Unclassified;f__Unclassified;g__Amphobotrys;s__Amphobotrys ricini 5e-147 KR527112.1

OTU_1574 k__Eukaryota;p__Ascomycota;c__Saccharomycetes;o__Saccharomycetales;f__Unclassified;g__Candida;s__Candida quercitrusa 2e-162 MF574303.1

OTU_1575 k__Eukaryota;p__Mucoromycota;c__Unclassified;o__Mortierellales;f__Unclassified;g__Unclassified;s__uncultured Mortierellales 2e-94 MF482194.1

OTU_1576 k__Eukaryota;p__Ascomycota;c__Dothideomycetes;o__Pleosporales;f__Phaeosphaeriaceae;g__Parastagonospora;s__Parastagonospora nodorum 6e-14 CP022842.1

OTU_1577 k__Eukaryota;p__Unclassified;c__Unclassified;o__Unclassified;f__Unclassified;g__Unclassified;s__uncultured fungus 4e-160 KX192672.1

OTU_1578 k__Eukaryota;p__Ascomycota;c__Leotiomycetes;o__Erysiphales;f__Erysiphaceae;g__Unclassified;s__uncultured Blumeria 1e-163 HG937004.1

OTU_1579 k__Eukaryota;p__Basidiomycota;c__Tremellomycetes;o__Tremellales;f__Rhynchogastremataceae;g__Papiliotrema;s__Papiliotrema terrestris 3e-158 KY495734.1

OTU_158 k__Eukaryota;p__Ascomycota;c__Dothideomycetes;o__Pleosporales;f__Pleosporaceae;g__Neocamarosporium;s__Neocamarosporium sp. 2e-165 MG065823.1

OTU_1580 k__Eukaryota;p__Ascomycota;c__Sordariomycetes;o__Xylariales;f__Bartaliniaceae;g__Bartalinia;s__Bartalinia pondoensis 2e-143 KU556132.1

OTU_1581 k__Eukaryota;p__Basidiomycota;c__Tremellomycetes;o__Tremellales;f__Rhynchogastremataceae;g__Papiliotrema;s__Papiliotrema flavescens 1e-166 KY104461.1

OTU_1582 k__Eukaryota;p__Basidiomycota;c__Exobasidiomycetes;o__Exobasidiales;f__Exobasidiaceae;g__Exobasidium;s__Exobasidium rhododendri 2e-144 EU784219.1

OTU_1583 k__Eukaryota;p__Ascomycota;c__Dothideomycetes;o__Dothideales;f__Saccotheciaceae;g__Aureobasidium;s__Aureobasidium sp. JSKim-2015 6e-116 LC018816.1

OTU_1584 k__Eukaryota;p__Basidiomycota;c__Agaricomycetes;o__Polyporales;f__Polyporaceae;g__Coriolopsis;s__Coriolopsis sp. 0.0 KX099628.1

OTU_1585 k__Eukaryota;p__Ascomycota;c__Dothideomycetes;o__Pleosporales;f__Phaeosphaeriaceae;g__Ophiosphaerella;s__Ophiosphaerella agrostidis 2e-168 KP017823.1

OTU_1586 k__Eukaryota;p__Ascomycota;c__Sordariomycetes;o__Xylariales;f__Hypoxylaceae;g__Hypoxylon;s__Hypoxylon begae 2e-168 KT224877.1

OTU_1587 k__Eukaryota;p__Unclassified;c__Unclassified;o__Unclassified;f__Unclassified;g__Unclassified;s__uncultured fungus 0.0 EU516702.1

OTU_1588 k__Eukaryota;p__Basidiomycota;c__Unclassified;o__Unclassified;f__Unclassified;g__Unclassified;s__Basidiomycota sp. 0.0 MF788213.1

OTU_1589 k__Eukaryota;p__Unclassified;c__Unclassified;o__Unclassified;f__Unclassified;g__Unclassified;s__fungal sp. CA151RZ 4e-43 KP403992.1

OTU_159 k__Eukaryota;p__Ascomycota;c__Eurotiomycetes;o__Eurotiales;f__Aspergillaceae;g__Penicillium;s__Penicillium sp. QTYC19 2e-155 KM103307.1

OTU_1590 k__Eukaryota;p__Unclassified;c__Unclassified;o__Unclassified;f__Unclassified;g__Unclassified;s__uncultured fungus 2e-78 KX193962.1

OTU_1591 k__Eukaryota;p__Ascomycota;c__Dothideomycetes;o__Capnodiales;f__Neodevriesiaceae;g__Neodevriesia;s__Neodevriesia poagena 2e-165 KR611885.1

OTU_1592 k__Eukaryota;p__Ascomycota;c__Eurotiomycetes;o__Chaetothyriales;f__Trichomeriaceae;g__Knufia;s__Knufia aspidiotus 2e-177 JX843780.1

OTU_1593 k__Eukaryota;p__Ascomycota;c__Pezizomycetes;o__Pezizales;f__Unclassified;g__Unclassified;s__uncultured Pezizales 1e-142 MF487530.1

OTU_1594 k__Eukaryota;p__Unclassified;c__Unclassified;o__Unclassified;f__Unclassified;g__Unclassified;s__uncultured fungus 2e-159 KX193023.1

OTU_1595 k__Eukaryota;p__Ascomycota;c__Sordariomycetes;o__Glomerellales;f__Glomerellaceae;g__Colletotrichum;s__Colletotrichum gloeosporioides 2e-97 KX906602.1

OTU_1596 k__Eukaryota;p__Ascomycota;c__Dothideomycetes;o__Dothideales;f__Saccotheciaceae;g__Aureobasidium;s__Aureobasidium pullulans 8e-137 KY294714.1

OTU_1597 k__Eukaryota;p__Ascomycota;c__Sordariomycetes;o__Glomerellales;f__Glomerellaceae;g__Colletotrichum;s__Colletotrichum gloeosporioides 2e-125 KX906602.1

OTU_1598 k__Eukaryota;p__Ascomycota;c__Sordariomycetes;o__Sordariales;f__Chaetomiaceae;g__Collariella;s__Collariella virescens 3e-164 MG250411.1

OTU_1599 k__Eukaryota;p__Ascomycota;c__Eurotiomycetes;o__Onygenales;f__Onygenaceae;g__Auxarthron;s__Auxarthron sp. 2e-100 KU702351.1

OTU_16 k__Eukaryota;p__Ascomycota;c__Eurotiomycetes;o__Eurotiales;f__Aspergillaceae;g__Penicillium;s__Penicillium brevicompactum 2e-171 KY929273.1

OTU_160 k__Eukaryota;p__Unclassified;c__Unclassified;o__Unclassified;f__Unclassified;g__Unclassified;s__uncultured fungus 2e-152 MF570315.1

OTU_1600 k__Bacteria;p__Proteobacteria;c__Alphaproteobacteria;o__Rhodospirillales;f__Acetobacteraceae;g__Gluconobacter;s__Gluconobacter albidus 2e-146 CP014689.1

OTU_1601 k__Eukaryota;p__Ascomycota;c__Dothideomycetes;o__Pleosporales;f__Periconiaceae;g__Periconia;s__Periconia sp. 4LY-3 1e-166 KU375677.1

OTU_1602 k__Eukaryota;p__Ascomycota;c__Saccharomycetes;o__Saccharomycetales;f__Pichiaceae;g__Pichia;s__Pichia sp. TCJ3 2e-134 HM044858.1

OTU_1603 k__Eukaryota;p__Unclassified;c__Unclassified;o__Unclassified;f__Unclassified;g__Unclassified;s__uncultured fungus 3e-158 MF569449.1

OTU_1604 k__Eukaryota;p__Unclassified;c__Unclassified;o__Unclassified;f__Unclassified;g__Unclassified;s__uncultured fungus 3e-179 KX192881.1

OTU_1605 k__Eukaryota;p__Unclassified;c__Unclassified;o__Unclassified;f__Unclassified;g__Unclassified;s__uncultured fungus 2e-162 KX516515.1

OTU_1606 k__Eukaryota;p__Ascomycota;c__Leotiomycetes;o__Helotiales;f__Sclerotiniaceae;g__Botrytis;s__Botrytis cinerea 1e-132 KY419551.1

OTU_1607 k__Bacteria;p__Proteobacteria;c__Alphaproteobacteria;o__Rhodospirillales;f__Acetobacteraceae;g__Komagataeibacter;s__Komagataeibacter xylinus 5e-76 CP004360.1

OTU_1608 k__Eukaryota;p__Ascomycota;c__Dothideomycetes;o__Pleosporales;f__Leptosphaeriaceae;g__Leptosphaeria;s__Leptosphaeria biglobosa 4e-06 FO906069.1

OTU_1609 k__Eukaryota;p__Ascomycota;c__Eurotiomycetes;o__Onygenales;f__Unclassified;g__Chrysosporium;s__Chrysosporium merdarium 4e-163 KY320600.1

OTU_161 k__Eukaryota;p__Ascomycota;c__Sordariomycetes;o__Sordariales;f__Sordariaceae;g__Neurospora;s__Neurospora sp. 7e-165 KX987245.1

OTU_1610 k__Eukaryota;p__Ascomycota;c__Sordariomycetes;o__Hypocreales;f__Unclassified;g__Acremonium;s__Acremonium sclerotigenum 9e-140 KY929277.1

OTU_1611 k__Eukaryota;p__Ascomycota;c__Dothideomycetes;o__Pleosporales;f__Phaeosphaeriaceae;g__Muriphaeosphaeria;s__Muriphaeosphaeria ambrosiae 2e-165 KX765267.1

OTU_162 k__Eukaryota;p__Ascomycota;c__Dothideomycetes;o__Pleosporales;f__Massarinaceae;g__Stagonospora;s__Stagonospora sp. 2e-165 MF788208.1

OTU_163 k__Eukaryota;p__Ascomycota;c__Sordariomycetes;o__Xylariales;f__Sporocadaceae;g__Pestalotiopsis;s__Pestalotiopsis sp. UCD10Syrah 2e-171 HM849055.1

OTU_164 k__Eukaryota;p__Ascomycota;c__Saccharomycetes;o__Saccharomycetales;f__Saccharomycetaceae;g__Lachancea;s__Lachancea kluyveri 0.0 KY103979.1

OTU_165 k__Eukaryota;p__Ascomycota;c__Dothideomycetes;o__Capnodiales;f__Mycosphaerellaceae;g__Zasmidium;s__Zasmidium dalbergiae 4e-12 NR_137803.1

OTU_166 k__Eukaryota;p__Ascomycota;c__Dothideomycetes;o__Pleosporales;f__Didymosphaeriaceae;g__Dendrothyrium;s__Dendrothyrium sp. 7e-168 KX774142.1

OTU_167 k__Eukaryota;p__Ascomycota;c__Unclassified;o__Unclassified;f__Unclassified;g__Unclassified;s__uncultured Ascomycota 5e-141 HM239801.1

OTU_168 k__Eukaryota;p__Ascomycota;c__Eurotiomycetes;o__Eurotiales;f__Aspergillaceae;g__Penicillium;s__Penicillium guanacastense 7e-168 KU556125.1

OTU_169 k__Eukaryota;p__Ascomycota;c__Saccharomycetes;o__Saccharomycetales;f__Saccharomycodaceae;g__Saccharomycodes;s__Saccharomycodes ludwigii 0.0 KY105244.1

OTU_17 k__Eukaryota;p__Ascomycota;c__Dothideomycetes;o__Capnodiales;f__Mycosphaerellaceae;g__Paramycosphaerella;s__Paramycosphaerella marksii 4e-160 GQ852747.1

OTU_170 k__Eukaryota;p__Unclassified;c__Unclassified;o__Unclassified;f__Unclassified;g__Unclassified;s__uncultured fungus 5e-163 MF570611.1

OTU_171 k__Eukaryota;p__Ascomycota;c__Dothideomycetes;o__Capnodiales;f__Mycosphaerellaceae;g__Septoria;s__Septoria arundinacea 3e-167 GU361964.1

OTU_172 k__Eukaryota;p__Unclassified;c__Unclassified;o__Unclassified;f__Unclassified;g__Unclassified;s__uncultured fungus 6e-159 KC965786.1

OTU_173 k__Eukaryota;p__Unclassified;c__Unclassified;o__Unclassified;f__Unclassified;g__Unclassified;s__fungal endophyte 6e-162 KY038612.1

OTU_174 k__Eukaryota;p__Unclassified;c__Unclassified;o__Unclassified;f__Unclassified;g__Unclassified;s__fungal sp. L10 5e-135 KF887091.1

OTU_175 k__Eukaryota;p__Ascomycota;c__Sordariomycetes;o__Hypocreales;f__Cordycipitaceae;g__Isaria;s__Isaria farinosa 2e-171 MF950892.1

OTU_176 k__Eukaryota;p__Basidiomycota;c__Exobasidiomycetes;o__Microstromatales;f__Unclassified;g__Pseudomicrostroma;s__Pseudomicrostroma juglandis 0.0 KY367530.1

OTU_177 k__Eukaryota;p__Ascomycota;c__Eurotiomycetes;o__Eurotiales;f__Aspergillaceae;g__Aspergillus;s__Aspergillus welwitschiae 7e-162 MG576117.1

OTU_178 k__Eukaryota;p__Unclassified;c__Unclassified;o__Unclassified;f__Unclassified;g__Unclassified;s__uncultured fungus 1e-150 KX515855.1

OTU_179 k__Eukaryota;p__Unclassified;c__Unclassified;o__Unclassified;f__Unclassified;g__Unclassified;s__fungal endophyte 1e-141 KP335569.1

OTU_18 k__Eukaryota;p__Basidiomycota;c__Tremellomycetes;o__Filobasidiales;f__Filobasidiaceae;g__Filobasidium;s__Filobasidium magnum 0.0 KY558595.1

OTU_180 k__Eukaryota;p__Mucoromycota;c__Unclassified;o__Mucorales;f__Mucoraceae;g__Mucor;s__Mucor circinelloides 0.0 KC460850.1

OTU_181 k__Eukaryota;p__Unclassified;c__Unclassified;o__Unclassified;f__Unclassified;g__Unclassified;s__uncultured fungus 6e-150 GU174335.1

OTU_182 k__Eukaryota;p__Basidiomycota;c__Cystobasidiomycetes;o__Unclassified;f__Unclassified;g__Symmetrospora;s__Symmetrospora coprosmae 0.0 KX067833.1

OTU_183 k__Eukaryota;p__Ascomycota;c__Dothideomycetes;o__Pleosporales;f__Pleosporaceae;g__Comoclathris;s__Comoclathris sp. 1e-156 MG065813.1

OTU_184 k__Eukaryota;p__Ascomycota;c__Dothideomycetes;o__Pleosporales;f__Sporormiaceae;g__Unclassified;s__uncultured Preussia 4e-163 KR232517.1

OTU_185 k__Eukaryota;p__Ascomycota;c__Dothideomycetes;o__Capnodiales;f__Schizothyriaceae;g__Zygophiala;s__Zygophiala emperorae 4e-163 KF857348.1

OTU_186 k__Eukaryota;p__Basidiomycota;c__Exobasidiomycetes;o__Exobasidiales;f__Brachybasidiaceae;g__Meira;s__Meira sp. 17 YHW-2013 0.0 JX682694.1

OTU_187 k__Eukaryota;p__Unclassified;c__Unclassified;o__Unclassified;f__Unclassified;g__Unclassified;s__uncultured fungus 3e-173 MF570664.1

OTU_188 k__Eukaryota;p__Ascomycota;c__Dothideomycetes;o__Unclassified;f__Unclassified;g__Leptospora;s__Leptospora galii 2e-168 KX599547.1

OTU_189 k__Eukaryota;p__Basidiomycota;c__Agaricomycetes;o__Cantharellales;f__Unclassified;g__Unclassified;s__uncultured Minimedusa 0.0 HG936627.1

OTU_19 k__Eukaryota;p__Ascomycota;c__Sordariomycetes;o__Hypocreales;f__Nectriaceae;g__Fusarium;s__Gibberella sp. 2e-165 KY413688.1

OTU_190 k__Eukaryota;p__Unclassified;c__Unclassified;o__Unclassified;f__Unclassified;g__Unclassified;s__uncultured fungus 0.0 MF570325.1

OTU_191 k__Eukaryota;p__Ascomycota;c__Sordariomycetes;o__Diaporthales;f__Valsaceae;g__Valsa;s__Valsa mali 8e-174 MF442534.1

OTU_192 k__Eukaryota;p__Unclassified;c__Unclassified;o__Unclassified;f__Unclassified;g__Unclassified;s__uncultured fungus 2e-150 GU187883.1

OTU_194 k__Eukaryota;p__Ascomycota;c__Dothideomycetes;o__Capnodiales;f__Dissoconiaceae;g__Dissoconium;s__Dissoconium sp. N1.9B5 7e-162 KJ719561.1

OTU_195 k__Eukaryota;p__Unclassified;c__Unclassified;o__Unclassified;f__Unclassified;g__Unclassified;s__uncultured fungus 7e-171 JX489817.1

OTU_196 k__Eukaryota;p__Unclassified;c__Unclassified;o__Unclassified;f__Unclassified;g__Unclassified;s__uncultured fungus 2e-168 DQ421059.1

OTU_197 k__Eukaryota;p__Ascomycota;c__Saccharomycetes;o__Saccharomycetales;f__Saccharomycopsidaceae;g__Saccharomycopsis;s__Saccharomycopsis fibuligera 0.0 MG519729.1

OTU_198 k__Eukaryota;p__Ascomycota;c__Eurotiomycetes;o__Eurotiales;f__Aspergillaceae;g__Penicillium;s__Penicillium copticola 8e-177 MF326615.1

OTU_199 k__Eukaryota;p__Unclassified;c__Unclassified;o__Unclassified;f__Unclassified;g__Unclassified;s__uncultured fungus 8e-11 MF568708.1

OTU_2 k__Eukaryota;p__Ascomycota;c__Saccharomycetes;o__Saccharomycetales;f__Saccharomycodaceae;g__Hanseniaspora;s__Hanseniaspora uvarum 0.0 MG020690.1

OTU_20 k__Eukaryota;p__Basidiomycota;c__Agaricomycetes;o__Agaricales;f__Bolbitiaceae;g__Panaeolus;s__Panaeolus sp. 0.0 MF629829.1

OTU_200 k__Eukaryota;p__Ascomycota;c__Dothideomycetes;o__Pleosporales;f__Sporormiaceae;g__Preussia;s__Preussia funiculata 1e-132 KC427070.1

OTU_201 k__Eukaryota;p__Basidiomycota;c__Agaricomycetes;o__Agaricales;f__Psathyrellaceae;g__Unclassified;s__uncultured Psathyrellaceae 6e-178 KX115720.1

OTU_202 k__Eukaryota;p__Unclassified;c__Unclassified;o__Unclassified;f__Unclassified;g__Unclassified;s__uncultured fungus 7e-165 MF568872.1

OTU_203 k__Eukaryota;p__Ascomycota;c__Saccharomycetes;o__Saccharomycetales;f__Debaryomycetaceae;g__Candida;s__Candida albicans 2e-165 KY611820.1

OTU_204 k__Eukaryota;p__Basidiomycota;c__Cystobasidiomycetes;o__Erythrobasidiales;f__Erythrobasidiaceae;g__Erythrobasidium;s__Erythrobasidium hasegawianum 0.0 FJ515203.1

OTU_205 k__Eukaryota;p__Ascomycota;c__Dothideomycetes;o__Capnodiales;f__Mycosphaerellaceae;g__Virosphaerella;s__Virosphaerella pseudomarksii 2e-152 EU882113.1

OTU_206 k__Eukaryota;p__Ascomycota;c__Unclassified;o__Unclassified;f__Unclassified;g__Unclassified;s__uncultured Ascomycota 5e-135 MG207413.1

OTU_207 k__Eukaryota;p__Basidiomycota;c__Unclassified;o__Unclassified;f__Unclassified;g__Unclassified;s__uncultured Basidiomycota 4e-154 DQ672271.1

OTU_208 k__Eukaryota;p__Ascomycota;c__Pezizomycetes;o__Unclassified;f__Unclassified;g__Unclassified;s__Pezizomycetes sp. 9e-152 KP991751.1

OTU_209 k__Eukaryota;p__Unclassified;c__Unclassified;o__Unclassified;f__Unclassified;g__Unclassified;s__uncultured fungus 6e-156 MF569941.1

OTU_21 k__Eukaryota;p__Ascomycota;c__Saccharomycetes;o__Saccharomycetales;f__Phaffomycetaceae;g__Wickerhamomyces;s__Wickerhamomyces anomalus 0.0 MG519713.1

OTU_210 k__Eukaryota;p__Unclassified;c__Unclassified;o__Unclassified;f__Unclassified;g__Unclassified;s__uncultured fungus 2e-158 KC965721.1

OTU_211 k__Eukaryota;p__Ascomycota;c__Saccharomycetes;o__Saccharomycetales;f__Saccharomycetaceae;g__Nakaseomyces;s__[Candida] glabrata 0.0 KY963096.1

OTU_212 k__Eukaryota;p__Ascomycota;c__Sordariomycetes;o__Hypocreales;f__Nectriaceae;g__Fusarium;s__Fusarium sp. HS19 8e-174 KP091289.1

OTU_213 k__Eukaryota;p__Ascomycota;c__Saccharomycetes;o__Saccharomycetales;f__Saccharomycodaceae;g__Hanseniaspora;s__Hanseniaspora sp. 0.0 KY977693.1

OTU_214 k__Eukaryota;p__Unclassified;c__Unclassified;o__Unclassified;f__Unclassified;g__Unclassified;s__uncultured fungus 6e-122 MF569941.1

OTU_215 k__Eukaryota;p__Unclassified;c__Unclassified;o__Unclassified;f__Unclassified;g__Unclassified;s__uncultured fungus 1e-153 KP897774.1

OTU_216 k__Eukaryota;p__Unclassified;c__Unclassified;o__Unclassified;f__Unclassified;g__Unclassified;s__fungal sp. TZ-2015b 5e-141 KR698815.1

OTU_217 k__Eukaryota;p__Unclassified;c__Unclassified;o__Unclassified;f__Unclassified;g__Unclassified;s__fungal sp. 1e-172 MF347767.1

OTU_218 k__Eukaryota;p__Unclassified;c__Unclassified;o__Unclassified;f__Unclassified;g__Unclassified;s__fungal endophyte 4e-123 KP335348.1

OTU_219 k__Eukaryota;p__Ascomycota;c__Pezizomycetes;o__Pezizales;f__Ascodesmidaceae;g__Cephaliophora;s__Cephaliophora sp. 5e-169 KY814682.1

OTU_22 k__Eukaryota;p__Unclassified;c__Unclassified;o__Unclassified;f__Unclassified;g__Unclassified;s__uncultured fungus 6e-159 MF976091.1

OTU_220 k__Eukaryota;p__Ascomycota;c__Sordariomycetes;o__Diaporthales;f__Diaporthaceae;g__Diaporthe;s__Diaporthe phaseolorum 1e-166 KR708965.1

OTU_221 k__Eukaryota;p__Unclassified;c__Unclassified;o__Unclassified;f__Unclassified;g__Unclassified;s__uncultured fungus 6e-162 MF568944.1

OTU_222 k__Eukaryota;p__Basidiomycota;c__Agaricomycetes;o__Agaricales;f__Cortinariaceae;g__Gymnopilus;s__Gymnopilus igniculus 0.0 HG969656.1

OTU_223 k__Eukaryota;p__Ascomycota;c__Saccharomycetes;o__Saccharomycetales;f__Saccharomycodaceae;g__Hanseniaspora;s__Hanseniaspora uvarum 0.0 MG020690.1

OTU_224 k__Eukaryota;p__Basidiomycota;c__Tremellomycetes;o__Trichosporonales;f__Trichosporonaceae;g__Cryptococcus;s__Cryptococcus aff. amylolyticus AS 2.2398 0.0 EF363151.1

OTU_225 k__Eukaryota;p__Unclassified;c__Unclassified;o__Unclassified;f__Unclassified;g__Unclassified;s__uncultured fungus 3e-121 MF976066.1

OTU_226 k__Eukaryota;p__Unclassified;c__Unclassified;o__Unclassified;f__Unclassified;g__Unclassified;s__uncultured fungus 5e-144 KX515551.1

OTU_227 k__Eukaryota;p__Ascomycota;c__Dothideomycetes;o__Pleosporales;f__Pleosporaceae;g__Setosphaeria;s__Setosphaeria rostrata 0.0 MG573216.1

OTU_228 k__Eukaryota;p__Unclassified;c__Unclassified;o__Unclassified;f__Unclassified;g__Unclassified;s__uncultured fungus 1e-172 KX515960.1

OTU_229 k__Eukaryota;p__Ascomycota;c__Dothideomycetes;o__Dothideales;f__Saccotheciaceae;g__Aureobasidium;s__Aureobasidium pullulans 2e-159 MG333439.1

OTU_23 k__Eukaryota;p__Ascomycota;c__Sordariomycetes;o__Diaporthales;f__Schizoparmaceae;g__Coniella;s__Coniella sp. KTC-2017a 3e-173 KX890016.1

OTU_230 k__Eukaryota;p__Unclassified;c__Unclassified;o__Unclassified;f__Unclassified;g__Unclassified;s__uncultured fungus 5e-104 KP897406.1

OTU_231 k__Eukaryota;p__Basidiomycota;c__Tremellomycetes;o__Unclassified;f__Unclassified;g__Cryptococcus;s__Cryptococcus sp. 0.0 LC272866.1

OTU_232 k__Eukaryota;p__Ascomycota;c__Eurotiomycetes;o__Eurotiales;f__Aspergillaceae;g__Aspergillus;s__Aspergillus flavus 5e-175 MG576099.1

OTU_233 k__Eukaryota;p__Ascomycota;c__Sordariomycetes;o__Hypocreales;f__Unclassified;g__Unclassified;s__Hypocreales sp. B1a0194PD2CC591 5e-172 KP322782.1

OTU_234 k__Eukaryota;p__Basidiomycota;c__Agaricomycetes;o__Hymenochaetales;f__Unclassified;g__Rickenella;s__Rickenella fibula 0.0 JQ694100.1

OTU_235 k__Eukaryota;p__Unclassified;c__Unclassified;o__Unclassified;f__Unclassified;g__Unclassified;s__uncultured fungus 2e-168 DQ420807.1

OTU_236 k__Eukaryota;p__Unclassified;c__Unclassified;o__Unclassified;f__Unclassified;g__Unclassified;s__uncultured fungus 0.0 MF570611.1

OTU_237 k__Eukaryota;p__Ascomycota;c__Dothideomycetes;o__Pleosporales;f__Phaeosphaeriaceae;g__Phaeosphaeria;s__Phaeosphaeria sp. 7e-165 KT264716.1

OTU_238 k__Eukaryota;p__Ascomycota;c__Sordariomycetes;o__Diaporthales;f__Valsaceae;g__Phomopsis;s__Phomopsis sp. 2e-168 MF800893.1

OTU_239 k__Eukaryota;p__Ascomycota;c__Dothideomycetes;o__Capnodiales;f__Cladosporiaceae;g__Cladosporium;s__Cladosporium cladosporioides 1e-147 KY929278.1

OTU_24 k__Eukaryota;p__Unclassified;c__Unclassified;o__Unclassified;f__Unclassified;g__Unclassified;s__uncultured fungus 1e-113 FJ237229.1

OTU_240 k__Eukaryota;p__Ascomycota;c__Sordariomycetes;o__Hypocreales;f__Nectriaceae;g__Fusarium;s__Fusarium delphinoides 8e-174 KY776658.1

OTU_241 k__Eukaryota;p__Ascomycota;c__Saccharomycetes;o__Saccharomycetales;f__Saccharomycetaceae;g__Kazachstania;s__Kazachstania humilis 0.0 HG532084.1

OTU_242 k__Eukaryota;p__Ascomycota;c__Dothideomycetes;o__Pleosporales;f__Didymellaceae;g__Stagonosporopsis;s__Stagonosporopsis ligulicola 5e-169 KJ868167.1

OTU_243 k__Eukaryota;p__Ascomycota;c__Saccharomycetes;o__Saccharomycetales;f__Saccharomycodaceae;g__Hanseniaspora;s__Hanseniaspora uvarum 0.0 MG020690.1

OTU_244 k__Eukaryota;p__Ascomycota;c__Dothideomycetes;o__Pleosporales;f__Phaeosphaeriaceae;g__Wojnowiciella;s__Wojnowiciella cissampeli 4e-160 KX228272.1

OTU_245 k__Eukaryota;p__Unclassified;c__Unclassified;o__Unclassified;f__Unclassified;g__Unclassified;s__uncultured fungus 3e-161 KX515747.1

OTU_246 k__Eukaryota;p__Ascomycota;c__Saccharomycetes;o__Saccharomycetales;f__Saccharomycetaceae;g__Zygosaccharomyces;s__Zygosaccharomyces bisporus 3e-179 KY106029.1

OTU_247 k__Eukaryota;p__Unclassified;c__Unclassified;o__Unclassified;f__Unclassified;g__Unclassified;s__uncultured fungus 1e-172 MF570167.1

OTU_248 k__Eukaryota;p__Ascomycota;c__Dothideomycetes;o__Unclassified;f__Unclassified;g__Unclassified;s__Dothideomycetes sp. 1e-92 KP991455.1

OTU_249 k__Eukaryota;p__Unclassified;c__Unclassified;o__Unclassified;f__Unclassified;g__Unclassified;s__uncultured fungus 1e-163 MF568725.1

OTU_25 k__Eukaryota;p__Ascomycota;c__Eurotiomycetes;o__Eurotiales;f__Aspergillaceae;g__Penicillium;s__Penicillium sp. QTYC19 5e-169 KM103307.1

OTU_250 k__Eukaryota;p__Ascomycota;c__Leotiomycetes;o__Helotiales;f__Hyaloscyphaceae;g__Solenopezia;s__Solenopezia solenia 2e-87 U57991.1

OTU_251 k__Eukaryota;p__Basidiomycota;c__Tremellomycetes;o__Filobasidiales;f__Filobasidiaceae;g__Filobasidium;s__Filobasidium wieringae 0.0 KY037853.1

OTU_252 k__Eukaryota;p__Unclassified;c__Unclassified;o__Unclassified;f__Unclassified;g__Unclassified;s__fungal sp. 7e-168 KY945013.1

OTU_253 k__Eukaryota;p__Unclassified;c__Unclassified;o__Unclassified;f__Unclassified;g__Unclassified;s__uncultured fungus 2e-177 KF800191.1

OTU_254 k__Eukaryota;p__Unclassified;c__Unclassified;o__Unclassified;f__Unclassified;g__Unclassified;s__uncultured fungus 4e-108 MF570027.1

OTU_255 k__Eukaryota;p__Unclassified;c__Unclassified;o__Unclassified;f__Unclassified;g__Unclassified;s__uncultured fungus 0.0 MF570960.1

OTU_256 k__Eukaryota;p__Basidiomycota;c__Agaricomycetes;o__Cantharellales;f__Ceratobasidiaceae;g__Unclassified;s__uncultured Thanatephorus 0.0 AB712278.1

OTU_257 k__Eukaryota;p__Unclassified;c__Unclassified;o__Unclassified;f__Unclassified;g__Unclassified;s__uncultured fungus 1e-123 JX984796.1

OTU_258 k__Eukaryota;p__Ascomycota;c__Dothideomycetes;o__Capnodiales;f__Cladosporiaceae;g__Cladosporium;s__Cladosporium macrocarpum 3e-139 MG385087.1

OTU_259 k__Eukaryota;p__Unclassified;c__Unclassified;o__Unclassified;f__Unclassified;g__Unclassified;s__uncultured fungus 4e-157 KP889774.1

OTU_26 k__Eukaryota;p__Ascomycota;c__Saccharomycetes;o__Saccharomycetales;f__Debaryomycetaceae;g__Meyerozyma;s__Meyerozyma caribbica 0.0 KY363459.1

OTU_260 k__Eukaryota;p__Ascomycota;c__Sordariomycetes;o__Hypocreales;f__Nectriaceae;g__Fusarium;s__Fusarium polyphialidicum 4e-166 NR_144930.1

OTU_261 k__Eukaryota;p__Unclassified;c__Unclassified;o__Unclassified;f__Unclassified;g__Unclassified;s__fungal sp. 8e-177 KY775828.1

OTU_262 k__Eukaryota;p__Ascomycota;c__Dothideomycetes;o__Unclassified;f__Micropeltidaceae;g__Stomiopeltis;s__Stomiopeltis sp. T36A1b 3e-136 JQ358788.1

OTU_263 k__Eukaryota;p__Basidiomycota;c__Agaricostilbomycetes;o__Agaricostilbales;f__Kondoaceae;g__Kondoa;s__Kondoa sorbi 0.0 AY233343.1

OTU_264 k__Eukaryota;p__Ascomycota;c__Sordariomycetes;o__Xylariales;f__Amphisphaeriaceae;g__Unclassified;s__Amphisphaeriaceae sp. 4e-163 KX721644.1

OTU_265 k__Eukaryota;p__Unclassified;c__Unclassified;o__Unclassified;f__Unclassified;g__Unclassified;s__uncultured fungus 6e-159 JN889866.1

OTU_266 k__Eukaryota;p__Ascomycota;c__Sordariomycetes;o__Diaporthales;f__Diaporthaceae;g__Diaporthe;s__Diaporthe sp. 1e-169 KX953400.1

OTU_267 k__Eukaryota;p__Unclassified;c__Unclassified;o__Unclassified;f__Unclassified;g__Unclassified;s__uncultured fungus 3e-167 HG327913.1

OTU_268 k__Eukaryota;p__Unclassified;c__Unclassified;o__Unclassified;f__Unclassified;g__Unclassified;s__uncultured fungus 1e-150 KP897609.1

OTU_269 k__Eukaryota;p__Unclassified;c__Unclassified;o__Unclassified;f__Unclassified;g__Unclassified;s__uncultured fungus 2e-174 GQ851846.1

OTU_27 k__Eukaryota;p__Basidiomycota;c__Exobasidiomycetes;o__Exobasidiales;f__Cryptobasidiaceae;g__Acaromyces;s__Acaromyces ingoldii 4e-166 HM595575.1

OTU_270 k__Eukaryota;p__Unclassified;c__Unclassified;o__Unclassified;f__Unclassified;g__Unclassified;s__uncultured fungus 0.0 AB981988.1

OTU_271 k__Eukaryota;p__Ascomycota;c__Sordariomycetes;o__Sordariales;f__Sordariaceae;g__Unclassified;s__uncultured Sordaria 1e-160 HG936858.1

OTU_272 k__Eukaryota;p__Unclassified;c__Unclassified;o__Unclassified;f__Unclassified;g__Unclassified;s__uncultured fungus 2e-168 KX515758.1

OTU_273 k__Eukaryota;p__Basidiomycota;c__Exobasidiomycetes;o__Ceraceosorales;f__Unclassified;g__Unclassified;s__Ceraceosorales sp. SA252w 2e-113 KM591597.1

OTU_274 k__Eukaryota;p__Ascomycota;c__Dothideomycetes;o__Dothideales;f__Dothioraceae;g__Unclassified;s__uncultured Dothioraceae 1e-160 FJ553079.1

OTU_275 k__Eukaryota;p__Unclassified;c__Unclassified;o__Unclassified;f__Unclassified;g__Unclassified;s__uncultured fungus 2e-78 KT243423.1

OTU_276 k__Eukaryota;p__Unclassified;c__Unclassified;o__Unclassified;f__Unclassified;g__Unclassified;s__uncultured fungus 8e-140 KC884479.1

OTU_277 k__Eukaryota;p__Basidiomycota;c__Tremellomycetes;o__Tremellales;f__Sirobasidiaceae;g__Sirobasidium;s__Sirobasidium brefeldianum 6e-73 JN053472.1

OTU_278 k__Eukaryota;p__Ascomycota;c__Dothideomycetes;o__Pleosporales;f__Lentitheciaceae;g__Keissleriella;s__Keissleriella cirsii 7e-165 KY497783.1

OTU_279 k__Eukaryota;p__Ascomycota;c__Dothideomycetes;o__Pleosporales;f__Unclassified;g__Unclassified;s__Pleosporales sp. 1 TMS-2011 6e-162 HQ631002.1

OTU_28 k__Eukaryota;p__Unclassified;c__Unclassified;o__Unclassified;f__Unclassified;g__Unclassified;s__uncultured fungus 1e-172 KX192911.1

OTU_280 k__Eukaryota;p__Unclassified;c__Unclassified;o__Unclassified;f__Unclassified;g__Unclassified;s__uncultured fungus 9e-180 HG764536.1

OTU_281 k__Eukaryota;p__Ascomycota;c__Dothideomycetes;o__Pleosporales;f__Pleosporaceae;g__Bipolaris;s__Bipolaris stenospila 5e-172 MG551561.1

OTU_282 k__Eukaryota;p__Ascomycota;c__Unclassified;o__Unclassified;f__Unclassified;g__Unclassified;s__uncultured Ascomycota 1e-157 FR682366.1

OTU_283 k__Eukaryota;p__Basidiomycota;c__Microbotryomycetes;o__Sporidiobolales;f__Sporidiobolaceae;g__Rhodotorula;s__Rhodotorula mucilaginosa 0.0 MG270571.1

OTU_284 k__Eukaryota;p__Basidiomycota;c__Tremellomycetes;o__Unclassified;f__Unclassified;g__Cryptococcus;s__Cryptococcus sp. 0.0 KX620506.1

OTU_285 k__Eukaryota;p__Basidiomycota;c__Exobasidiomycetes;o__Ceraceosorales;f__Unclassified;g__Unclassified;s__Ceraceosorales sp. SA252w 1e-107 KM591597.1

OTU_286 k__Eukaryota;p__Ascomycota;c__Eurotiomycetes;o__Onygenales;f__Unclassified;g__Unclassified;s__uncultured Emmonsia 2e-177 JQ247333.1

OTU_287 k__Eukaryota;p__Unclassified;c__Unclassified;o__Unclassified;f__Unclassified;g__Unclassified;s__uncultured fungus 1e-160 KX194608.1

OTU_288 k__Eukaryota;p__Ascomycota;c__Dothideomycetes;o__Venturiales;f__Sympoventuriaceae;g__Scolecobasidium;s__Scolecobasidium terreum 6e-70 HQ667544.1

OTU_289 k__Eukaryota;p__Ascomycota;c__Sordariomycetes;o__Sordariales;f__Chaetomiaceae;g__Chaetomium;s__Chaetomium grande 1e-157 KU702687.1

OTU_29 k__Eukaryota;p__Basidiomycota;c__Microbotryomycetes;o__Sporidiobolales;f__Unclassified;g__Unclassified;s__uncultured Sporidiobolales 0.0 KP974240.1

OTU_290 k__Eukaryota;p__Unclassified;c__Unclassified;o__Unclassified;f__Unclassified;g__Unclassified;s__uncultured fungus 0.0 HQ446066.1

OTU_291 k__Eukaryota;p__Ascomycota;c__Dothideomycetes;o__Pleosporales;f__Pleosporaceae;g__Curvularia;s__Curvularia lunata 8e-174 MG576110.1

OTU_292 k__Eukaryota;p__Basidiomycota;c__Agaricomycetes;o__Thelephorales;f__Thelephoraceae;g__Unclassified;s__uncultured Tomentella 0.0 JX898972.1

OTU_293 k__Eukaryota;p__Ascomycota;c__Sordariomycetes;o__Sordariales;f__Chaetomiaceae;g__Subramaniula;s__Subramaniula thielavioides 7e-162 MG250407.1

OTU_294 k__Eukaryota;p__Ascomycota;c__Dothideomycetes;o__Pleosporales;f__Lentitheciaceae;g__Keissleriella;s__Keissleriella cirsii 1e-120 KY497783.1

OTU_295 k__Eukaryota;p__Ascomycota;c__Sordariomycetes;o__Hypocreales;f__Unclassified;g__Acremonium;s__Acremonium sp. 904C 3e-161 KF669512.1

OTU_296 k__Eukaryota;p__Ascomycota;c__Sordariomycetes;o__Hypocreales;f__Unclassified;g__Acremonium;s__Acremonium sp. KUC21262 5e-175 KT207780.1

OTU_297 k__Eukaryota;p__Ascomycota;c__Dothideomycetes;o__Pleosporales;f__Phaeosphaeriaceae;g__Wojnowiciella;s__Wojnowiciella leptocarpi 5e-135 KX306775.1

OTU_298 k__Eukaryota;p__Unclassified;c__Unclassified;o__Unclassified;f__Unclassified;g__Unclassified;s__uncultured fungus 1e-166 MF569559.1

OTU_299 k__Eukaryota;p__Ascomycota;c__Eurotiomycetes;o__Eurotiales;f__Aspergillaceae;g__Penicillium;s__Penicillium chrysogenum 1e-172 KU216743.1

OTU_3 k__Eukaryota;p__Ascomycota;c__Dothideomycetes;o__Pleosporales;f__Pleosporaceae;g__Alternaria;s__Alternaria alternata 3e-170 MG576108.1

OTU_30 k__Eukaryota;p__Unclassified;c__Unclassified;o__Unclassified;f__Unclassified;g__Unclassified;s__uncultured fungus 7e-168 MF569273.1

OTU_300 k__Eukaryota;p__Ascomycota;c__Saccharomycetes;o__Saccharomycetales;f__Trigonopsidaceae;g__Botryozyma;s__Botryozyma nematodophila 0.0 KY101765.1

OTU_301 k__Eukaryota;p__Basidiomycota;c__Tremellomycetes;o__Tremellales;f__Trimorphomycetaceae;g__Saitozyma;s__Saitozyma podzolica 2e-171 LC155937.1

OTU_302 k__Eukaryota;p__Unclassified;c__Unclassified;o__Unclassified;f__Unclassified;g__Unclassified;s__uncultured fungus 1e-153 MF569322.1

OTU_303 k__Eukaryota;p__Ascomycota;c__Unclassified;o__Unclassified;f__Unclassified;g__Unclassified;s__ascomycete sp. ALr-1 2e-81 EF210107.1

OTU_304 k__Eukaryota;p__Ascomycota;c__Dothideomycetes;o__Pleosporales;f__Didymellaceae;g__Allophoma;s__Allophoma zantedeschiae 7e-168 KX033403.1

OTU_305 k__Eukaryota;p__Ascomycota;c__Dothideomycetes;o__Dothideales;f__Dothioraceae;g__Sydowia;s__Sydowia polyspora 5e-172 KX099686.1

OTU_306 k__Eukaryota;p__Ascomycota;c__Sordariomycetes;o__Sordariales;f__Unclassified;g__Unclassified;s__uncultured Sordariales 2e-158 HQ389529.1

OTU_307 k__Eukaryota;p__Ascomycota;c__Sordariomycetes;o__Glomerellales;f__Glomerellaceae;g__Colletotrichum;s__Colletotrichum viniferum 4e-160 KX786436.1

OTU_308 k__Eukaryota;p__Mucoromycota;c__Unclassified;o__Mucorales;f__Mucoraceae;g__Mucor;s__Mucor fragilis 3e-176 MF800910.1

OTU_309 k__Eukaryota;p__Ascomycota;c__Leotiomycetes;o__Unclassified;f__Pseudeurotiaceae;g__Leuconeurospora;s__Leuconeurospora sp. T11Cd2 2e-158 JQ857041.1

OTU_31 k__Eukaryota;p__Basidiomycota;c__Microbotryomycetes;o__Sporidiobolales;f__Sporidiobolaceae;g__Sporidiobolus;s__Sporidiobolus pararoseus 0.0 KY226609.1

OTU_310 k__Eukaryota;p__Ascomycota;c__Leotiomycetes;o__Helotiales;f__Sclerotiniaceae;g__Botrytis;s__Botrytis cinerea 3e-145 KY419551.1

OTU_311 k__Eukaryota;p__Ascomycota;c__Eurotiomycetes;o__Onygenales;f__Onygenaceae;g__Auxarthron;s__Auxarthron sp. 1e-163 KT264293.1

OTU_312 k__Eukaryota;p__Unclassified;c__Unclassified;o__Unclassified;f__Unclassified;g__Unclassified;s__uncultured fungus 2e-162 FJ362343.1

OTU_313 k__Eukaryota;p__Unclassified;c__Unclassified;o__Unclassified;f__Unclassified;g__Unclassified;s__fungal sp. 0.0 MG189961.1

OTU_314 k__Eukaryota;p__Unclassified;c__Unclassified;o__Unclassified;f__Unclassified;g__Unclassified;s__uncultured fungus 3e-127 KC588639.1

OTU_315 k__Eukaryota;p__Mucoromycota;c__Unclassified;o__Mortierellales;f__Mortierellaceae;g__Mortierella;s__Mortierella elongata 0.0 MF671811.1

OTU_316 k__Eukaryota;p__Ascomycota;c__Dothideomycetes;o__Pleosporales;f__Sporormiaceae;g__Preussia;s__Preussia grandispora 6e-159 KX710255.1

OTU_317 k__Eukaryota;p__Ascomycota;c__Dothideomycetes;o__Dothideales;f__Saccotheciaceae;g__Aureobasidium;s__Aureobasidium sp. JSKim-2015 9e-146 LC018816.1

OTU_318 k__Eukaryota;p__Basidiomycota;c__Tremellomycetes;o__Tremellales;f__Cryptococcaceae;g__Kwoniella;s__Kwoniella shandongensis 0.0 JQ327851.1

OTU_319 k__Eukaryota;p__Ascomycota;c__Dothideomycetes;o__Unclassified;f__Unclassified;g__Unclassified;s__Dothideomycetes sp. Ponipodef 11 1e-169 HQ731651.1

OTU_32 k__Eukaryota;p__Ascomycota;c__Dothideomycetes;o__Capnodiales;f__Dissoconiaceae;g__Ramichloridium;s__Ramichloridium punctatum 2e-168 KU131679.1

OTU_320 k__Eukaryota;p__Unclassified;c__Unclassified;o__Unclassified;f__Unclassified;g__Unclassified;s__uncultured fungus 2e-162 HQ625442.1

OTU_321 k__Eukaryota;p__Unclassified;c__Unclassified;o__Unclassified;f__Unclassified;g__Unclassified;s__uncultured fungus 5e-150 KF617763.1

OTU_322 k__Eukaryota;p__Ascomycota;c__Sordariomycetes;o__Sordariales;f__Chaetomiaceae;g__Chaetomium;s__Chaetomium iranianum 2e-165 NR_145148.1

OTU_323 k__Eukaryota;p__Ascomycota;c__Dothideomycetes;o__Pleosporales;f__Leptosphaeriaceae;g__Ophiobolus;s__Ophiobolus disseminans 7e-168 KP117305.1

OTU_324 k__Eukaryota;p__Ascomycota;c__Sordariomycetes;o__Hypocreales;f__Cordycipitaceae;g__Beauveria;s__Beauveria bassiana 2e-171 MG012792.1

OTU_325 k__Eukaryota;p__Unclassified;c__Unclassified;o__Unclassified;f__Unclassified;g__Unclassified;s__uncultured fungus 2e-143 EF504322.1

OTU_326 k__Eukaryota;p__Ascomycota;c__Sordariomycetes;o__Hypocreales;f__Unclassified;g__Sarocladium;s__Sarocladium strictum 3e-179 KY465767.1

OTU_327 k__Eukaryota;p__Unclassified;c__Unclassified;o__Unclassified;f__Unclassified;g__Unclassified;s__uncultured fungus 2e-36 KP897400.1

OTU_328 k__Eukaryota;p__Ascomycota;c__Sordariomycetes;o__Hypocreales;f__Cordycipitaceae;g__Cordyceps;s__Cordyceps militaris 3e-161 CP023322.1

OTU_329 k__Eukaryota;p__Ascomycota;c__Dothideomycetes;o__Pleosporales;f__Pleosporaceae;g__Alternaria;s__Alternaria alternata 3e-161 MG576108.1

OTU_33 k__Eukaryota;p__Ascomycota;c__Sordariomycetes;o__Xylariales;f__Unclassified;g__Robillarda;s__Robillarda sessilis 3e-167 KU892285.1

OTU_330 k__Eukaryota;p__Ascomycota;c__Dothideomycetes;o__Pleosporales;f__Cucurbitariaceae;g__Pyrenochaeta;s__Pyrenochaeta cava 7e-168 LC163507.1

OTU_331 k__Eukaryota;p__Ascomycota;c__Dothideomycetes;o__Pleosporales;f__Pleosporaceae;g__Alternaria;s__Alternaria sp. 2e-162 MF326585.1

OTU_332 k__Eukaryota;p__Unclassified;c__Unclassified;o__Unclassified;f__Unclassified;g__Unclassified;s__uncultured fungus 6e-162 GQ892250.1

OTU_333 k__Eukaryota;p__Ascomycota;c__Dothideomycetes;o__Pleosporales;f__Pleosporaceae;g__Alternaria;s__Alternaria alternata 1e-160 KT192381.1

OTU_334 k__Eukaryota;p__Ascomycota;c__Geoglossomycetes;o__Geoglossales;f__Geoglossaceae;g__Trichoglossum;s__Trichoglossum durandii 2e-171 HQ222875.1

OTU_335 k__Eukaryota;p__Unclassified;c__Unclassified;o__Unclassified;f__Unclassified;g__Unclassified;s__uncultured fungus 2e-13 KX195274.1

OTU_336 k__Eukaryota;p__Ascomycota;c__Dothideomycetes;o__Dothideales;f__Saccotheciaceae;g__Kabatiella;s__Kabatiella sp. SQU-MA24 1e-150 KU945924.1

OTU_337 k__Eukaryota;p__Ascomycota;c__Dothideomycetes;o__Pleosporales;f__Sporormiaceae;g__Preussia;s__Preussia funiculata 7e-168 KP101185.1

OTU_338 k__Eukaryota;p__Unclassified;c__Unclassified;o__Unclassified;f__Unclassified;g__Unclassified;s__fungal sp. 4e-163 KU747774.1

OTU_339 k__Eukaryota;p__Basidiomycota;c__Tremellomycetes;o__Trichosporonales;f__Trichosporonaceae;g__Apiotrichum;s__Apiotrichum porosum 3e-173 KY929281.1

OTU_34 k__Eukaryota;p__Ascomycota;c__Dothideomycetes;o__Capnodiales;f__Mycosphaerellaceae;g__Cercospora;s__Cercospora citrullina 2e-161 KY824771.1

OTU_340 k__Eukaryota;p__Ascomycota;c__Unclassified;o__Unclassified;f__Unclassified;g__Unclassified;s__uncultured Coniosporium 3e-161 KF385284.1

OTU_341 k__Eukaryota;p__Unclassified;c__Unclassified;o__Unclassified;f__Unclassified;g__Unclassified;s__uncultured fungus 1e-172 KX195844.1

OTU_342 k__Eukaryota;p__Ascomycota;c__Sordariomycetes;o__Hypocreales;f__Unclassified;g__Acremonium;s__Acremonium sp. 8e-177 MG572351.1

OTU_343 k__Eukaryota;p__Ascomycota;c__Sordariomycetes;o__Glomerellales;f__Glomerellaceae;g__Colletotrichum;s__Colletotrichum graminicola 3e-170 KF278451.1

OTU_344 k__Eukaryota;p__Ascomycota;c__Leotiomycetes;o__Helotiales;f__Sclerotiniaceae;g__Botrytis;s__Botrytis cinerea 4e-151 KY419551.1

OTU_345 k__Eukaryota;p__Unclassified;c__Unclassified;o__Unclassified;f__Unclassified;g__Unclassified;s__uncultured fungus 1e-163 KX193979.1

OTU_346 k__Eukaryota;p__Unclassified;c__Unclassified;o__Unclassified;f__Unclassified;g__Unclassified;s__fungal endophyte 8e-146 KT203050.1

OTU_347 k__Eukaryota;p__Ascomycota;c__Dothideomycetes;o__Capnodiales;f__Dissoconiaceae;g__Ramichloridium;s__Ramichloridium eucleae 1e-141 KJ869155.1

OTU_348 k__Eukaryota;p__Ascomycota;c__Sordariomycetes;o__Glomerellales;f__Glomerellaceae;g__Colletotrichum;s__Colletotrichum capsici 2e-168 KY659060.1

OTU_349 k__Eukaryota;p__Basidiomycota;c__Tremellomycetes;o__Tremellales;f__Unclassified;g__Unclassified;s__uncultured Tremellales 2e-11 KF060236.1

OTU_35 k__Eukaryota;p__Basidiomycota;c__Microbotryomycetes;o__Sporidiobolales;f__Sporidiobolaceae;g__Rhodotorula;s__Rhodotorula sp. 0.0 KU350341.1

OTU_350 k__Eukaryota;p__Ascomycota;c__Eurotiomycetes;o__Eurotiales;f__Aspergillaceae;g__Penicillium;s__Penicillium griseofulvum 4e-163 AB369901.1

OTU_351 k__Eukaryota;p__Unclassified;c__Unclassified;o__Unclassified;f__Unclassified;g__Unclassified;s__uncultured fungus 5e-166 KX195268.1

OTU_352 k__Eukaryota;p__Chytridiomycota;c__Neocallimastigomycetes;o__Neocallimastigales;f__Unclassified;g__Unclassified;s__uncultured Neocallimastigales 0.0 GU909864.1

OTU_353 k__Eukaryota;p__Ascomycota;c__Eurotiomycetes;o__Eurotiales;f__Aspergillaceae;g__Penicillium;s__Penicillium sclerotiorum 3e-161 MF135518.1

OTU_354 k__Eukaryota;p__Ascomycota;c__Unclassified;o__Unclassified;f__Unclassified;g__Unclassified;s__uncultured Ascomycota 0.0 FR682361.1

OTU_355 k__Eukaryota;p__Unclassified;c__Unclassified;o__Unclassified;f__Unclassified;g__Unclassified;s__uncultured fungus 6e-156 MF569901.1

OTU_356 k__Eukaryota;p__Unclassified;c__Unclassified;o__Unclassified;f__Unclassified;g__Unclassified;s__uncultured fungus 3e-59 KU537036.1

OTU_357 k__Eukaryota;p__Basidiomycota;c__Tremellomycetes;o__Unclassified;f__Unclassified;g__Cryptococcus;s__Cryptococcus sp. 7e-162 AJ344111.1

OTU_358 k__Eukaryota;p__Unclassified;c__Unclassified;o__Unclassified;f__Unclassified;g__Unclassified;s__uncultured fungus 8e-11 MF571177.1

OTU_359 k__Eukaryota;p__Ascomycota;c__Dothideomycetes;o__Pleosporales;f__Phaeosphaeriaceae;g__Phaeosphaeria;s__Phaeosphaeria ammophilae 1e-135 KF766146.1

OTU_36 k__Eukaryota;p__Unclassified;c__Unclassified;o__Unclassified;f__Unclassified;g__Unclassified;s__fungal endophyte 2e-171 KY765183.1

OTU_360 k__Eukaryota;p__Ascomycota;c__Sordariomycetes;o__Xylariales;f__Sporocadaceae;g__Monochaetia;s__Monochaetia dimorphospora 7e-162 LC146750.1

OTU_361 k__Eukaryota;p__Ascomycota;c__Dothideomycetes;o__Pleosporales;f__Phaeosphaeriaceae;g__Dematiopleospora;s__Dematiopleospora cirsii 8e-140 KX274243.1

OTU_362 k__Eukaryota;p__Unclassified;c__Unclassified;o__Unclassified;f__Unclassified;g__Unclassified;s__fungal sp. 1e-141 KU728286.1

OTU_363 k__Eukaryota;p__Basidiomycota;c__Tremellomycetes;o__Tremellales;f__Rhynchogastremataceae;g__Papiliotrema;s__Papiliotrema cf. pseudoalba MG27 0.0 KM246223.1

OTU_364 k__Eukaryota;p__Unclassified;c__Unclassified;o__Unclassified;f__Unclassified;g__Unclassified;s__fungal endophyte 3e-173 KY022798.1

OTU_365 k__Eukaryota;p__Unclassified;c__Unclassified;o__Unclassified;f__Unclassified;g__Unclassified;s__uncultured fungus 1e-147 KX194439.1

OTU_366 k__Eukaryota;p__Ascomycota;c__Dothideomycetes;o__Pleosporales;f__Phaeosphaeriaceae;g__Nodulosphaeria;s__Nodulosphaeria multiseptata 2e-94 KY496748.1

OTU_367 k__Eukaryota;p__Ascomycota;c__Eurotiomycetes;o__Onygenales;f__Onygenaceae;g__Auxarthron;s__Auxarthron conjugatum 5e-135 MF996764.1

OTU_368 k__Eukaryota;p__Ascomycota;c__Dothideomycetes;o__Capnodiales;f__Dissoconiaceae;g__Dissoconium;s__Dissoconium sp. DA001 2e-168 JX415826.1

OTU_369 k__Eukaryota;p__Ascomycota;c__Leotiomycetes;o__Helotiales;f__Sclerotiniaceae;g__Botrytis;s__Botrytis cinerea 1e-144 KY419551.1

OTU_37 k__Eukaryota;p__Unclassified;c__Unclassified;o__Unclassified;f__Unclassified;g__Unclassified;s__fungal endophyte 2e-155 KT203193.1

OTU_370 k__Eukaryota;p__Ascomycota;c__Dothideomycetes;o__Pleosporales;f__Periconiaceae;g__Periconia;s__Periconia macrospinosa 8e-171 MG576113.1

OTU_371 k__Eukaryota;p__Unclassified;c__Unclassified;o__Unclassified;f__Unclassified;g__Unclassified;s__uncultured fungus 4e-163 MF569712.1

OTU_372 k__Eukaryota;p__Basidiomycota;c__Unclassified;o__Unclassified;f__Unclassified;g__Unclassified;s__basidiomycete sp. IasaF07 0.0 DQ117964.1

OTU_373 k__Eukaryota;p__Basidiomycota;c__Cystobasidiomycetes;o__Cystobasidiales;f__Cystobasidiaceae;g__Cystobasidium;s__Cystobasidium sp. 0.0 LC272874.1

OTU_374 k__Eukaryota;p__Ascomycota;c__Dothideomycetes;o__Capnodiales;f__Mycosphaerellaceae;g__Pseudocercospora;s__Pseudocercospora carbonacea 2e-84 KC677897.1

OTU_375 k__Eukaryota;p__Unclassified;c__Unclassified;o__Unclassified;f__Unclassified;g__Unclassified;s__uncultured fungus 7e-168 MF569101.1

OTU_376 k__Eukaryota;p__Ascomycota;c__Eurotiomycetes;o__Eurotiales;f__Aspergillaceae;g__Penicillium;s__Penicillium raistrickii 3e-173 KY558625.1

OTU_377 k__Eukaryota;p__Ascomycota;c__Leotiomycetes;o__Helotiales;f__Helotiaceae;g__Rhizoscyphus;s__Rhizoscyphus ericae aggregate 1e-160 LC131012.1

OTU_378 k__Eukaryota;p__Ascomycota;c__Sordariomycetes;o__Hypocreales;f__Unclassified;g__Fusariella;s__Fusariella sinensis 2e-162 KP269058.1

OTU_379 k__Eukaryota;p__Unclassified;c__Unclassified;o__Unclassified;f__Unclassified;g__Unclassified;s__uncultured fungus 3e-59 MF570987.1

OTU_38 k__Eukaryota;p__Ascomycota;c__Dothideomycetes;o__Pleosporales;f__Didymellaceae;g__Phoma;s__Phoma betae 7e-165 KC460811.1

OTU_380 k__Eukaryota;p__Ascomycota;c__Dothideomycetes;o__Pleosporales;f__Unclassified;g__Unclassified;s__uncultured Pleosporales 9e-152 GQ924022.1

OTU_381 k__Eukaryota;p__Mucoromycota;c__Unclassified;o__Mortierellales;f__Mortierellaceae;g__Mortierella;s__Mortierella sp. S-24 0.0 KJ735018.1

OTU_382 k__Eukaryota;p__Ascomycota;c__Dothideomycetes;o__Capnodiales;f__Cladosporiaceae;g__Cladosporium;s__Cladosporium cladosporioides 5e-135 KY929278.1

OTU_383 k__Eukaryota;p__Basidiomycota;c__Tremellomycetes;o__Tremellales;f__Rhynchogastremataceae;g__Papiliotrema;s__Papiliotrema terrestris 1e-154 KY495734.1

OTU_384 k__Eukaryota;p__Ascomycota;c__Dothideomycetes;o__Unclassified;f__Unclassified;g__Unclassified;s__Dothideomycetes sp. genotype 701 JMUR-2016 1e-169 KX908683.1

OTU_385 k__Eukaryota;p__Unclassified;c__Unclassified;o__Unclassified;f__Unclassified;g__Unclassified;s__uncultured fungus 6e-141 KP898052.1

OTU_386 k__Eukaryota;p__Ascomycota;c__Dothideomycetes;o__Pleosporales;f__Unclassified;g__Unclassified;s__uncultured Pleosporales 2e-165 GU910977.1

OTU_387 k__Bacteria;p__Proteobacteria;c__Gammaproteobacteria;o__Pseudomonadales;f__Pseudomonadaceae;g__Pseudomonas;s__Pseudomonas stutzeri 6e-169 EU617084.1

OTU_388 k__Eukaryota;p__Basidiomycota;c__Cystobasidiomycetes;o__Cystobasidiales;f__Cystobasidiaceae;g__Cystobasidium;s__Cystobasidium pallidum 0.0 KY103146.1

OTU_389 k__Eukaryota;p__Unclassified;c__Unclassified;o__Unclassified;f__Unclassified;g__Unclassified;s__uncultured fungus 1e-166 EU144632.1

OTU_39 k__Eukaryota;p__Basidiomycota;c__Microbotryomycetes;o__Sporidiobolales;f__Sporidiobolaceae;g__Rhodosporidiobolus;s__Rhodosporidiobolus odoratus 0.0 KU609532.1

OTU_390 k__Eukaryota;p__Ascomycota;c__Dothideomycetes;o__Pleosporales;f__Cucurbitariaceae;g__Pyrenochaeta;s__Pyrenochaeta sp. DTO 305-C7 1e-169 KX147607.1

OTU_391 k__Eukaryota;p__Ascomycota;c__Saccharomycetes;o__Saccharomycetales;f__Phaffomycetaceae;g__Starmera;s__[Candida] stellimalicola 4e-160 JQ927544.1

OTU_392 k__Eukaryota;p__Ascomycota;c__Unclassified;o__Unclassified;f__Unclassified;g__Unclassified;s__Ascomycota sp. AR-2010 4e-160 HQ607855.1

OTU_393 k__Eukaryota;p__Basidiomycota;c__Tremellomycetes;o__Trichosporonales;f__Trichosporonaceae;g__Trichosporon;s__Trichosporon coremiiforme 5e-175 MF370976.1

OTU_394 k__Eukaryota;p__Unclassified;c__Unclassified;o__Unclassified;f__Unclassified;g__Unclassified;s__uncultured fungus 6e-156 KP889602.1

OTU_395 k__Eukaryota;p__Ascomycota;c__Unclassified;o__Unclassified;f__Unclassified;g__Unclassified;s__Ascomycota sp. UNEX FECRGA 2012E547 4e-160 KP899402.1

OTU_396 k__Eukaryota;p__Unclassified;c__Unclassified;o__Unclassified;f__Unclassified;g__Unclassified;s__fungal sp. 8e-146 KX611076.1

OTU_397 k__Eukaryota;p__Basidiomycota;c__Cystobasidiomycetes;o__Cystobasidiales;f__Cystobasidiaceae;g__Cystobasidium;s__Cystobasidium minutum 0.0 KY495759.1

OTU_398 k__Eukaryota;p__Ascomycota;c__Sordariomycetes;o__Xylariales;f__Amphisphaeriaceae;g__Seimatosporium;s__Seimatosporium discosioides 6e-159 EF600970.1

OTU_399 k__Eukaryota;p__Basidiomycota;c__Agaricomycetes;o__Agaricales;f__Tricholomataceae;g__Laccaria;s__Laccaria lateritia 0.0 MF461608.1

OTU_4 k__Eukaryota;p__Ascomycota;c__Sordariomycetes;o__Glomerellales;f__Glomerellaceae;g__Colletotrichum;s__Colletotrichum gloeosporioides 5e-169 KX906602.1

OTU_40 k__Eukaryota;p__Basidiomycota;c__Tremellomycetes;o__Tremellales;f__Bulleribasidiaceae;g__Dioszegia;s__Dioszegia zsoltii 2e-159 JF817339.1

OTU_400 k__Eukaryota;p__Unclassified;c__Unclassified;o__Unclassified;f__Unclassified;g__Unclassified;s__fungal sp. 2e-146 KY522928.1

OTU_401 k__Eukaryota;p__Ascomycota;c__Unclassified;o__Unclassified;f__Unclassified;g__Unclassified;s__uncultured Ascomycota 4e-163 HG937025.1

OTU_402 k__Eukaryota;p__Unclassified;c__Unclassified;o__Unclassified;f__Unclassified;g__Unclassified;s__uncultured fungus 1e-15 MF569488.1

OTU_403 k__Eukaryota;p__Unclassified;c__Unclassified;o__Unclassified;f__Unclassified;g__Unclassified;s__uncultured fungus 1e-86 MF570555.1

OTU_404 k__Eukaryota;p__Unclassified;c__Unclassified;o__Unclassified;f__Unclassified;g__Unclassified;s__uncultured fungus 1e-163 KX515412.1

OTU_405 k__Bacteria;p__Proteobacteria;c__Gammaproteobacteria;o__Enterobacterales;f__Enterobacteriaceae;g__Escherichia;s__Escherichia coli 8e-152 CP023377.1

OTU_406 k__Eukaryota;p__Unclassified;c__Unclassified;o__Unclassified;f__Unclassified;g__Unclassified;s__uncultured fungus 6e-150 HQ446092.1

OTU_407 k__Eukaryota;p__Ascomycota;c__Dothideomycetes;o__Pleosporales;f__Didymellaceae;g__Phoma;s__Phoma sp. 2e-161 MF061785.1

OTU_408 k__Eukaryota;p__Ascomycota;c__Eurotiomycetes;o__Eurotiales;f__Aspergillaceae;g__Penicillium;s__Penicillium sumatrense 5e-172 KJ775693.1

OTU_409 k__Eukaryota;p__Basidiomycota;c__Agaricomycetes;o__Agaricales;f__Cortinariaceae;g__Unclassified;s__uncultured Cortinarius 0.0 HM146806.1

OTU_41 k__Eukaryota;p__Unclassified;c__Unclassified;o__Unclassified;f__Unclassified;g__Unclassified;s__fungal sp. 7e-168 KU728286.1

OTU_410 k__Eukaryota;p__Ascomycota;c__Sordariomycetes;o__Xylariales;f__Bartaliniaceae;g__Truncatella;s__Truncatella angustata 6e-153 KX099657.1

OTU_411 k__Eukaryota;p__Ascomycota;c__Eurotiomycetes;o__Eurotiales;f__Aspergillaceae;g__Penicillium;s__Penicillium phoeniceum 7e-168 KY929272.1

OTU_412 k__Eukaryota;p__Ascomycota;c__Dothideomycetes;o__Pleosporales;f__Pleosporaceae;g__Unclassified;s__uncultured Neocamarosporium 3e-130 KU245684.1

OTU_413 k__Eukaryota;p__Unclassified;c__Unclassified;o__Unclassified;f__Unclassified;g__Unclassified;s__fungal endophyte 7e-134 HQ871964.1

OTU_414 k__Eukaryota;p__Unclassified;c__Unclassified;o__Unclassified;f__Unclassified;g__Unclassified;s__uncultured fungus 1e-138 KX192620.1

OTU_415 k__Eukaryota;p__Unclassified;c__Unclassified;o__Unclassified;f__Unclassified;g__Unclassified;s__uncultured fungus 3e-124 KF297007.1

OTU_416 k__Eukaryota;p__Unclassified;c__Unclassified;o__Unclassified;f__Unclassified;g__Unclassified;s__uncultured fungus 8e-11 MF568914.1

OTU_417 k__Eukaryota;p__Ascomycota;c__Sordariomycetes;o__Hypocreales;f__Unclassified;g__Trichothecium;s__Trichothecium roseum 0.0 MF288732.1

OTU_418 k__Eukaryota;p__Basidiomycota;c__Agaricomycetes;o__Agaricales;f__Cortinariaceae;g__Cortinarius;s__Cortinarius ahsii 0.0 KX882649.1

OTU_419 k__Eukaryota;p__Unclassified;c__Unclassified;o__Unclassified;f__Unclassified;g__Unclassified;s__uncultured fungus 4e-108 KX194578.1

OTU_42 k__Eukaryota;p__Ascomycota;c__Dothideomycetes;o__Capnodiales;f__Cladosporiaceae;g__Cladosporium;s__Cladosporium sphaerospermum 7e-165 KY929282.1

OTU_420 k__Eukaryota;p__Unclassified;c__Unclassified;o__Unclassified;f__Unclassified;g__Unclassified;s__uncultured fungus 1e-37 MF571123.1

OTU_421 k__Eukaryota;p__Ascomycota;c__Saccharomycetes;o__Saccharomycetales;f__Trichomonascaceae;g__Blastobotrys;s__Blastobotrys sp. AB-2017a 4e-126 MG018986.1

OTU_422 k__Eukaryota;p__Unclassified;c__Unclassified;o__Unclassified;f__Unclassified;g__Unclassified;s__uncultured fungus 1e-138 KX515855.1

OTU_423 k__Eukaryota;p__Ascomycota;c__Dothideomycetes;o__Capnodiales;f__Mycosphaerellaceae;g__Mycosphaerella;s__Mycosphaerella rosigena 3e-161 GU214632.1

OTU_424 k__Eukaryota;p__Basidiomycota;c__Agaricomycetes;o__Agaricales;f__Pleurotaceae;g__Nematoctonus;s__Nematoctonus robustus 0.0 EF409759.1

OTU_425 k__Eukaryota;p__Unclassified;c__Unclassified;o__Unclassified;f__Unclassified;g__Unclassified;s__uncultured fungus 1e-117 JF697069.1

OTU_426 k__Eukaryota;p__Unclassified;c__Unclassified;o__Unclassified;f__Unclassified;g__Unclassified;s__uncultured fungus 8e-140 EF505546.1

OTU_427 k__Eukaryota;p__Basidiomycota;c__Exobasidiomycetes;o__Unclassified;f__Unclassified;g__Unclassified;s__Exobasidiomycetidae sp. IBL 03150 0.0 DQ682574.2

OTU_428 k__Eukaryota;p__Unclassified;c__Unclassified;o__Unclassified;f__Unclassified;g__Unclassified;s__uncultured fungus 4e-132 MF570550.1

OTU_429 k__Eukaryota;p__Unclassified;c__Unclassified;o__Unclassified;f__Unclassified;g__Unclassified;s__fungal endophyte 2e-143 KT203050.1

OTU_43 k__Eukaryota;p__Ascomycota;c__Saccharomycetes;o__Saccharomycetales;f__Trichomonascaceae;g__Zygoascus;s__Zygoascus meyerae 0.0 KY495726.1

OTU_430 k__Eukaryota;p__Ascomycota;c__Dothideomycetes;o__Unclassified;f__Unclassified;g__Unclassified;s__Dothideomycetes sp. 2e-140 KX611079.1

OTU_431 k__Eukaryota;p__Unclassified;c__Unclassified;o__Unclassified;f__Unclassified;g__Unclassified;s__uncultured fungus 4e-06 MF571161.1

OTU_432 k__Eukaryota;p__Basidiomycota;c__Agaricomycetes;o__Polyporales;f__Polyporaceae;g__Perenniporia;s__Perenniporia tenuis 1e-178 HQ848474.1

OTU_433 k__Eukaryota;p__Unclassified;c__Unclassified;o__Unclassified;f__Unclassified;g__Unclassified;s__fungal sp. 1e-110 KU728286.1

OTU_434 k__Eukaryota;p__Basidiomycota;c__Tremellomycetes;o__Unclassified;f__Unclassified;g__Unclassified;s__uncultured Cryptococcus 1e-147 JF831497.1

OTU_435 k__Eukaryota;p__Unclassified;c__Unclassified;o__Unclassified;f__Unclassified;g__Unclassified;s__uncultured fungus 2e-121 KP828191.1

OTU_436 k__Eukaryota;p__Unclassified;c__Unclassified;o__Unclassified;f__Unclassified;g__Unclassified;s__uncultured fungus 2e-165 MF569343.1

OTU_437 k__Eukaryota;p__Ascomycota;c__Unclassified;o__Unclassified;f__Unclassified;g__Phacidiella;s__Phacidiella eucalypti 1e-126 EF110620.1

OTU_438 k__Eukaryota;p__Ascomycota;c__Sordariomycetes;o__Trichosphaeriales;f__Trichosphaeriaceae;g__Nigrospora;s__Nigrospora oryzae 3e-167 GU073125.1

OTU_439 k__Eukaryota;p__Ascomycota;c__Saccharomycetes;o__Saccharomycetales;f__Trichomonascaceae;g__Blastobotrys;s__Blastobotrys malaysiensis 7e-159 KY101753.1

OTU_44 k__Eukaryota;p__Ascomycota;c__Leotiomycetes;o__Erysiphales;f__Erysiphaceae;g__Erysiphe;s__Erysiphe necator 0.0 LC228619.1

OTU_440 k__Eukaryota;p__Ascomycota;c__Leotiomycetes;o__Helotiales;f__Helotiaceae;g__Phaeohelotium;s__Phaeohelotium succineoguttulatum 2e-143 KC411989.1

OTU_441 k__Eukaryota;p__Unclassified;c__Unclassified;o__Unclassified;f__Unclassified;g__Unclassified;s__uncultured fungus 3e-96 FN397293.1

OTU_442 k__Eukaryota;p__Unclassified;c__Unclassified;o__Unclassified;f__Unclassified;g__Unclassified;s__uncultured fungus 2e-131 EU144632.1

OTU_443 k__Eukaryota;p__Ascomycota;c__Dothideomycetes;o__Pleosporales;f__Pleosporaceae;g__Curvularia;s__Curvularia inaequalis 1e-172 KX826472.1

OTU_444 k__Eukaryota;p__Unclassified;c__Unclassified;o__Unclassified;f__Unclassified;g__Unclassified;s__uncultured fungus 5e-09 KX195142.1

OTU_445 k__Eukaryota;p__Ascomycota;c__Dothideomycetes;o__Capnodiales;f__Mycosphaerellaceae;g__Ramularia;s__Ramularia interstitiales 1e-160 KX287458.1

OTU_446 k__Eukaryota;p__Basidiomycota;c__Agaricomycetes;o__Thelephorales;f__Thelephoraceae;g__Unclassified;s__uncultured Thelephoraceae 0.0 GQ240903.1

OTU_447 k__Eukaryota;p__Basidiomycota;c__Agaricomycetes;o__Agaricales;f__Cortinariaceae;g__Cortinarius;s__Cortinarius croceus 0.0 GQ159909.1

OTU_448 k__Eukaryota;p__Unclassified;c__Unclassified;o__Unclassified;f__Unclassified;g__Unclassified;s__uncultured fungus 2e-143 HQ022292.1

OTU_449 k__Eukaryota;p__Ascomycota;c__Leotiomycetes;o__Thelebolales;f__Unclassified;g__Unclassified;s__uncultured Thelebolales 1e-163 GU910336.1

OTU_45 k__Eukaryota;p__Unclassified;c__Unclassified;o__Unclassified;f__Unclassified;g__Unclassified;s__uncultured fungus 5e-178 MF569279.1

OTU_450 k__Eukaryota;p__Unclassified;c__Unclassified;o__Unclassified;f__Unclassified;g__Unclassified;s__fungal sp. 1e-163 KY522700.1

OTU_451 k__Eukaryota;p__Ascomycota;c__Dothideomycetes;o__Pleosporales;f__Corynesporascaceae;g__Corynespora;s__Corynespora cassiicola 1e-172 MG333433.1

OTU_452 k__Eukaryota;p__Unclassified;c__Unclassified;o__Unclassified;f__Unclassified;g__Unclassified;s__uncultured fungus 2e-165 JX984769.1

OTU_453 k__Eukaryota;p__Ascomycota;c__Dothideomycetes;o__Pleosporales;f__Periconiaceae;g__Periconia;s__Periconia sp. EPU33CB 5e-169 KP128003.1

OTU_454 k__Eukaryota;p__Unclassified;c__Unclassified;o__Unclassified;f__Unclassified;g__Unclassified;s__uncultured fungus 0.0 KF800681.1

OTU_455 k__Eukaryota;p__Ascomycota;c__Dothideomycetes;o__Botryosphaeriales;f__Botryosphaeriaceae;g__Dothiorella;s__Dothiorella viticola 7e-168 KY385663.1

OTU_457 k__Eukaryota;p__Unclassified;c__Unclassified;o__Unclassified;f__Unclassified;g__Unclassified;s__uncultured fungus 9e-155 JX043029.1

OTU_458 k__Eukaryota;p__Basidiomycota;c__Tremellomycetes;o__Tremellales;f__Rhynchogastremataceae;g__Papiliotrema;s__Papiliotrema flavescens 2e-168 LC191378.1

OTU_459 k__Eukaryota;p__Ascomycota;c__Dothideomycetes;o__Dothideales;f__Saccotheciaceae;g__Aureobasidium;s__Aureobasidium pullulans 2e-146 KY294714.1

OTU_46 k__Eukaryota;p__Ascomycota;c__Saccharomycetes;o__Saccharomycetales;f__Saccharomycetaceae;g__Kazachstania;s__Kazachstania hellenica 0.0 KY103644.1

OTU_460 k__Eukaryota;p__Unclassified;c__Unclassified;o__Unclassified;f__Unclassified;g__Unclassified;s__uncultured fungus 7e-171 KX515681.1

OTU_461 k__Eukaryota;p__Ascomycota;c__Leotiomycetes;o__Helotiales;f__Helotiaceae;g__Unclassified;s__uncultured Helotiaceae 2e-161 FJ554443.1

OTU_462 k__Eukaryota;p__Ascomycota;c__Dothideomycetes;o__Pleosporales;f__Unclassified;g__Unclassified;s__uncultured Pleosporales 3e-148 GU910879.1

OTU_463 k__Eukaryota;p__Unclassified;c__Unclassified;o__Unclassified;f__Unclassified;g__Unclassified;s__uncultured fungus 5e-147 MF569365.1

OTU_464 k__Eukaryota;p__Basidiomycota;c__Agaricomycetes;o__Agaricales;f__Hydnangiaceae;g__Hydnangium;s__Hydnangium carneum 0.0 HQ832445.1

OTU_465 k__Eukaryota;p__Ascomycota;c__Eurotiomycetes;o__Phaeomoniellales;f__Unclassified;g__Neophaeomoniella;s__Neophaeomoniella zymoides 3e-173 KR909194.1

OTU_466 k__Eukaryota;p__Ascomycota;c__Unclassified;o__Unclassified;f__Unclassified;g__Unclassified;s__ascomycete sp. IMGERS-Plot IIc 1e-126 DQ884463.1

OTU_467 k__Eukaryota;p__Basidiomycota;c__Agaricomycetes;o__Corticiales;f__Corticiaceae;g__Sistotrema;s__Sistotrema cf. brinkmannii MX373 0.0 KJ831971.1

OTU_468 k__Eukaryota;p__Ascomycota;c__Dothideomycetes;o__Pleosporales;f__Unclassified;g__Unclassified;s__Pleosporales sp. OUCMBI101133 2e-165 HQ914849.1

OTU_469 k__Eukaryota;p__Ascomycota;c__Dothideomycetes;o__Pleosporales;f__Didymellaceae;g__Unclassified;s__uncultured Didymella 1e-08 HG936940.1

OTU_47 k__Eukaryota;p__Unclassified;c__Unclassified;o__Unclassified;f__Unclassified;g__Unclassified;s__fungal sp. TZ-2015b 4e-163 KR698902.1

OTU_470 k__Eukaryota;p__Ascomycota;c__Dothideomycetes;o__Pleosporales;f__Pleosporaceae;g__Curvularia;s__Curvularia intermedia 0.0 KX611668.1

OTU_471 k__Eukaryota;p__Ascomycota;c__Dothideomycetes;o__Botryosphaeriales;f__Botryosphaeriaceae;g__Phaeobotryon;s__Phaeobotryon sp. JC-2017a 2e-168 MF193891.1

OTU_472 k__Eukaryota;p__Basidiomycota;c__Cystobasidiomycetes;o__Unclassified;f__Unclassified;g__Symmetrospora;s__Symmetrospora sp. Gr 0.0 FJ613120.1

OTU_473 k__Eukaryota;p__Unclassified;c__Unclassified;o__Unclassified;f__Unclassified;g__Unclassified;s__uncultured fungus 7e-162 KP890998.1

OTU_474 k__Eukaryota;p__Ascomycota;c__Saccharomycetes;o__Saccharomycetales;f__Saccharomycodaceae;g__Hanseniaspora;s__Hanseniaspora occidentalis 0.0 JQ425357.1

OTU_475 k__Eukaryota;p__Unclassified;c__Unclassified;o__Unclassified;f__Unclassified;g__Unclassified;s__uncultured fungus 7e-11 MF571521.1

OTU_476 k__Eukaryota;p__Unclassified;c__Unclassified;o__Unclassified;f__Unclassified;g__Unclassified;s__uncultured fungus 1e-111 AM260797.1

OTU_477 k__Eukaryota;p__Basidiomycota;c__Tremellomycetes;o__Trichosporonales;f__Trichosporonaceae;g__Cryptococcus;s__Cryptococcus sp. HLS105 3e-164 FJ770078.1

OTU_478 k__Eukaryota;p__Ascomycota;c__Eurotiomycetes;o__Eurotiales;f__Aspergillaceae;g__Aspergillus;s__Aspergillus alliaceus 8e-171 MF417468.1

OTU_479 k__Eukaryota;p__Ascomycota;c__Sordariomycetes;o__Xylariales;f__Apiosporaceae;g__Arthrinium;s__Arthrinium saccharicola 3e-167 KT207737.1

OTU_48 k__Eukaryota;p__Basidiomycota;c__Tremellomycetes;o__Tremellales;f__Bulleribasidiaceae;g__Vishniacozyma;s__Vishniacozyma taibaiensis 1e-144 NR_144810.1

OTU_480 k__Eukaryota;p__Ascomycota;c__Unclassified;o__Unclassified;f__Unclassified;g__Acrophialophora;s__Acrophialophora sp. 4e-160 KY379656.1

OTU_481 k__Eukaryota;p__Unclassified;c__Unclassified;o__Unclassified;f__Unclassified;g__Unclassified;s__uncultured fungus 1e-126 MF570979.1

OTU_482 k__Eukaryota;p__Unclassified;c__Unclassified;o__Unclassified;f__Unclassified;g__Unclassified;s__uncultured fungus 1e-138 KC884399.1

OTU_483 k__Eukaryota;p__Unclassified;c__Unclassified;o__Unclassified;f__Unclassified;g__Unclassified;s__uncultured fungus 8e-174 MF570093.1

OTU_484 k__Eukaryota;p__Unclassified;c__Unclassified;o__Unclassified;f__Unclassified;g__Unclassified;s__uncultured fungus 3e-10 KX515370.1

OTU_485 k__Eukaryota;p__Unclassified;c__Unclassified;o__Unclassified;f__Unclassified;g__Unclassified;s__uncultured fungus 3e-07 KX192613.1

OTU_486 k__Eukaryota;p__Ascomycota;c__Dothideomycetes;o__Pleosporales;f__Unclassified;g__Unclassified;s__uncultured Pleosporales 3e-102 KJ827565.1

OTU_487 k__Eukaryota;p__Unclassified;c__Unclassified;o__Unclassified;f__Unclassified;g__Unclassified;s__uncultured fungus 6e-98 AB507831.1

OTU_488 k__Eukaryota;p__Ascomycota;c__Dothideomycetes;o__Pleosporales;f__Didymosphaeriaceae;g__Paraphaeosphaeria;s__Paraphaeosphaeria sp. 4e-163 KX774101.1

OTU_489 k__Eukaryota;p__Ascomycota;c__Eurotiomycetes;o__Chaetothyriales;f__Herpotrichiellaceae;g__Unclassified;s__uncultured Herpotrichiellaceae 1e-132 KX116039.1

OTU_49 k__Eukaryota;p__Ascomycota;c__Dothideomycetes;o__Pleosporales;f__Leptosphaeriaceae;g__Leptosphaeria;s__Leptosphaeria sp. 2e-159 KX721540.1

OTU_490 k__Eukaryota;p__Basidiomycota;c__Agaricomycetes;o__Agaricales;f__Omphalotaceae;g__Lentinula;s__Lentinula edodes 0.0 KU870678.1

OTU_491 k__Eukaryota;p__Ascomycota;c__Eurotiomycetes;o__Eurotiales;f__Trichocomaceae;g__Talaromyces;s__Talaromyces purpureogenus 1e-169 KY230505.1

OTU_492 k__Eukaryota;p__Unclassified;c__Unclassified;o__Unclassified;f__Unclassified;g__Unclassified;s__uncultured fungus 8e-11 MF568914.1

OTU_493 k__Eukaryota;p__Ascomycota;c__Dothideomycetes;o__Capnodiales;f__Cladosporiaceae;g__Cladosporium;s__Cladosporium tenuissimum 1e-150 MG572368.1

OTU_494 k__Eukaryota;p__Ascomycota;c__Dothideomycetes;o__Botryosphaeriales;f__Botryosphaeriaceae;g__Botryosphaeria;s__Botryosphaeria fabicerciana 1e-150 KY655197.1

OTU_495 k__Eukaryota;p__Basidiomycota;c__Tremellomycetes;o__Trichosporonales;f__Trichosporonaceae;g__Cryptococcus;s__Cryptococcus sp. YM24366 0.0 KM107823.1

OTU_496 k__Eukaryota;p__Ascomycota;c__Sordariomycetes;o__Togniniales;f__Togniniaceae;g__Phaeoacremonium;s__Phaeoacremonium sp. 13E199 6e-153 LC163525.1

OTU_497 k__Eukaryota;p__Unclassified;c__Unclassified;o__Unclassified;f__Unclassified;g__Unclassified;s__uncultured fungus 7e-134 MF569333.1

OTU_498 k__Eukaryota;p__Basidiomycota;c__Agaricomycetes;o__Auriculariales;f__Auriculariaceae;g__Auricularia;s__Auricularia polytricha 0.0 KY293392.1

OTU_499 k__Eukaryota;p__Ascomycota;c__Unclassified;o__Unclassified;f__Unclassified;g__Unclassified;s__uncultured Ascomycota 4e-120 MG207405.1

OTU_5 k__Eukaryota;p__Ascomycota;c__Dothideomycetes;o__Capnodiales;f__Cladosporiaceae;g__Cladosporium;s__Cladosporium cladosporioides 3e-164 MF173069.1

OTU_50 k__Eukaryota;p__Unclassified;c__Unclassified;o__Unclassified;f__Unclassified;g__Unclassified;s__uncultured fungus 1e-169 JX984795.1

OTU_500 k__Eukaryota;p__Unclassified;c__Unclassified;o__Unclassified;f__Unclassified;g__Unclassified;s__uncultured fungus 6e-153 MF568857.1

OTU_501 k__Eukaryota;p__Ascomycota;c__Dothideomycetes;o__Pleosporales;f__Didymosphaeriaceae;g__Paraconiothyrium;s__Paraconiothyrium estuarinum 1e-138 KX611652.1

OTU_502 k__Eukaryota;p__Ascomycota;c__Leotiomycetes;o__Erysiphales;f__Erysiphaceae;g__Erysiphe;s__Erysiphe heraclei 0.0 KY073878.1

OTU_503 k__Eukaryota;p__Ascomycota;c__Saccharomycetes;o__Saccharomycetales;f__Saccharomycodaceae;g__Hanseniaspora;s__Hanseniaspora uvarum 0.0 MG020690.1

OTU_504 k__Eukaryota;p__Ascomycota;c__Unclassified;o__Unclassified;f__Unclassified;g__Phaeodactylium;s__Phaeodactylium stadleri 3e-136 HF678526.1

OTU_505 k__Eukaryota;p__Unclassified;c__Unclassified;o__Unclassified;f__Unclassified;g__Unclassified;s__fungal sp. NLEndoHerit_029_2008N4-30-2C 5e-169 JX978258.1

OTU_506 k__Eukaryota;p__Unclassified;c__Unclassified;o__Unclassified;f__Unclassified;g__Unclassified;s__uncultured fungus 0.0 KX194687.1

OTU_507 k__Eukaryota;p__Unclassified;c__Unclassified;o__Unclassified;f__Unclassified;g__Unclassified;s__uncultured fungus 3e-142 EU689236.1

OTU_508 k__Eukaryota;p__Unclassified;c__Unclassified;o__Unclassified;f__Unclassified;g__Unclassified;s__fungal endophyte 3e-179 KF673761.1

OTU_509 k__Eukaryota;p__Ascomycota;c__Sordariomycetes;o__Hypocreales;f__Unclassified;g__Emericellopsis;s__Emericellopsis pallida 5e-172 MG250441.1

OTU_51 k__Eukaryota;p__Basidiomycota;c__Tremellomycetes;o__Filobasidiales;f__Filobasidiaceae;g__Naganishia;s__Naganishia albida 0.0 KY558586.1

OTU_510 k__Eukaryota;p__Unclassified;c__Unclassified;o__Unclassified;f__Unclassified;g__Unclassified;s__uncultured fungus 4e-49 MF570389.1

OTU_511 k__Eukaryota;p__Ascomycota;c__Dothideomycetes;o__Dothideales;f__Saccotheciaceae;g__Aureobasidium;s__Aureobasidium pullulans 3e-133 KY294714.1

OTU_512 k__Eukaryota;p__Unclassified;c__Unclassified;o__Unclassified;f__Unclassified;g__Unclassified;s__uncultured fungus 2e-162 KX193660.1

OTU_513 k__Eukaryota;p__Unclassified;c__Unclassified;o__Unclassified;f__Unclassified;g__Unclassified;s__uncultured fungus 0.0 AB520305.1

OTU_514 k__Eukaryota;p__Ascomycota;c__Sordariomycetes;o__Glomerellales;f__Glomerellaceae;g__Colletotrichum;s__Colletotrichum gloeosporioides 4e-83 KX906602.1

OTU_515 k__Eukaryota;p__Ascomycota;c__Dothideomycetes;o__Pleosporales;f__Leptosphaeriaceae;g__Leptosphaeria;s__Leptosphaeria microscopica 1e-157 MF576268.1

OTU_516 k__Eukaryota;p__Unclassified;c__Unclassified;o__Unclassified;f__Unclassified;g__Unclassified;s__uncultured fungus 4e-102 MF569495.1

OTU_517 k__Eukaryota;p__Ascomycota;c__Sordariomycetes;o__Xylariales;f__Unclassified;g__Unclassified;s__Xylariales sp. E15 4e-151 KF887081.1

OTU_518 k__Eukaryota;p__Ascomycota;c__Dothideomycetes;o__Pleosporales;f__Pleosporaceae;g__Alternaria;s__Alternaria sp. 6e-162 KY228354.1

OTU_519 k__Eukaryota;p__Unclassified;c__Unclassified;o__Unclassified;f__Unclassified;g__Unclassified;s__uncultured fungus 2e-91 KX193691.1

OTU_52 k__Eukaryota;p__Ascomycota;c__Sordariomycetes;o__Xylariales;f__Bartaliniaceae;g__Bartalinia;s__Bartalinia pondoensis 1e-166 KU059948.1

OTU_520 k__Eukaryota;p__Ascomycota;c__Dothideomycetes;o__Dothideales;f__Saccotheciaceae;g__Aureobasidium;s__Aureobasidium pullulans 9e-152 KX249734.1

OTU_521 k__Eukaryota;p__Ascomycota;c__Unclassified;o__Unclassified;f__Unclassified;g__Bahusakala;s__Bahusakala australiensis 2e-155 GQ272637.1

OTU_522 k__Eukaryota;p__Unclassified;c__Unclassified;o__Unclassified;f__Unclassified;g__Unclassified;s__uncultured fungus 3e-136 AF461661.1

OTU_523 k__Eukaryota;p__Unclassified;c__Unclassified;o__Unclassified;f__Unclassified;g__Unclassified;s__uncultured fungus 1e-163 KX664713.1

OTU_524 k__Eukaryota;p__Unclassified;c__Unclassified;o__Unclassified;f__Unclassified;g__Unclassified;s__fungal sp. 3e-167 KT948030.1

OTU_525 k__Eukaryota;p__Ascomycota;c__Dothideomycetes;o__Pleosporales;f__Didymellaceae;g__Phoma;s__Phoma sp. KACC 42479 3e-167 EF600963.1

OTU_526 k__Eukaryota;p__Basidiomycota;c__Agaricomycetes;o__Agaricales;f__Clavariaceae;g__Clavaria;s__Clavaria rosea 2e-11 KP257133.1

OTU_527 k__Eukaryota;p__Ascomycota;c__Sordariomycetes;o__Hypocreales;f__Ophiocordycipitaceae;g__Hirsutella;s__Hirsutella sp. Hx1214M 3e-164 KP670436.1

OTU_528 k__Eukaryota;p__Ascomycota;c__Eurotiomycetes;o__Eurotiales;f__Aspergillaceae;g__Aspergillus;s__Aspergillus restrictus 2e-174 KY087767.1

OTU_529 k__Eukaryota;p__Ascomycota;c__Dothideomycetes;o__Unclassified;f__Unclassified;g__Unclassified;s__Dothideomycetes sp. genotype 826 JMUR-2016 5e-144 KX908527.1

OTU_53 k__Eukaryota;p__Unclassified;c__Unclassified;o__Unclassified;f__Unclassified;g__Unclassified;s__uncultured fungus 1e-153 EF505561.1

OTU_530 k__Eukaryota;p__Unclassified;c__Unclassified;o__Unclassified;f__Unclassified;g__Unclassified;s__uncultured fungus 0.0 KF800096.1

OTU_531 k__Eukaryota;p__Ascomycota;c__Unclassified;o__Unclassified;f__Unclassified;g__Phacidiella;s__Phacidiella sp. DTO 305-C5 4e-15 KX147603.1

OTU_532 k__Eukaryota;p__Ascomycota;c__Dothideomycetes;o__Pleosporales;f__Unclassified;g__Unclassified;s__uncultured Pleosporales 2e-158 GQ924063.1

OTU_533 k__Eukaryota;p__Ascomycota;c__Dothideomycetes;o__Pleosporales;f__Didymosphaeriaceae;g__Paraphaeosphaeria;s__Paraphaeosphaeria sporulosa 3e-167 KY977581.1

OTU_534 k__Eukaryota;p__Ascomycota;c__Unclassified;o__Unclassified;f__Unclassified;g__Unclassified;s__uncultured Tetracladium 3e-136 KT581829.1

OTU_535 k__Eukaryota;p__Ascomycota;c__Dothideomycetes;o__Pleosporales;f__Sporormiaceae;g__Preussia;s__Preussia funiculata 1e-153 KC427070.1

OTU_536 k__Eukaryota;p__Ascomycota;c__Dothideomycetes;o__Capnodiales;f__Cladosporiaceae;g__Cladosporium;s__Cladosporium tenuissimum 2e-155 MG572368.1

OTU_537 k__Eukaryota;p__Ascomycota;c__Eurotiomycetes;o__Onygenales;f__Gymnoascaceae;g__Neogymnomyces;s__Neogymnomyces sp. 14PA11 7e-11 JX270473.1

OTU_538 k__Eukaryota;p__Unclassified;c__Unclassified;o__Unclassified;f__Unclassified;g__Unclassified;s__uncultured fungus 3e-164 HG764547.1

OTU_539 k__Eukaryota;p__Ascomycota;c__Dothideomycetes;o__Dothideales;f__Saccotheciaceae;g__Aureobasidium;s__aff. Aureobasidium sp. 5e-144 KT150707.1

OTU_54 k__Eukaryota;p__Ascomycota;c__Sordariomycetes;o__Xylariales;f__Unclassified;g__Hansfordia;s__Hansfordia pulvinata 7e-168 KP772241.1

OTU_540 k__Eukaryota;p__Unclassified;c__Unclassified;o__Unclassified;f__Unclassified;g__Unclassified;s__uncultured fungus 1e-150 GU931726.1

OTU_541 k__Eukaryota;p__Basidiomycota;c__Ustilaginomycetes;o__Ustilaginales;f__Ustilaginaceae;g__Tranzscheliella;s__Tranzscheliella yupeitaniae 0.0 KX832824.1

OTU_542 k__Eukaryota;p__Unclassified;c__Unclassified;o__Unclassified;f__Unclassified;g__Unclassified;s__uncultured fungus 8e-11 MF568708.1

OTU_543 k__Eukaryota;p__Ascomycota;c__Sordariomycetes;o__Xylariales;f__Amphisphaeriaceae;g__Synnemapestaloides;s__Synnemapestaloides rhododendri 3e-148 LC228666.1

OTU_544 k__Eukaryota;p__Unclassified;c__Unclassified;o__Unclassified;f__Unclassified;g__Unclassified;s__uncultured fungus 9e-152 MF570222.1

OTU_545 k__Eukaryota;p__Unclassified;c__Unclassified;o__Unclassified;f__Unclassified;g__Unclassified;s__uncultured fungus 2e-152 KF800431.1

OTU_546 k__Eukaryota;p__Unclassified;c__Unclassified;o__Unclassified;f__Unclassified;g__Unclassified;s__fungal sp. 3e-139 KY522928.1

OTU_547 k__Eukaryota;p__Ascomycota;c__Sordariomycetes;o__Phyllachorales;f__Unclassified;g__Unclassified;s__uncultured Phyllachorales 2e-158 EU726301.1

OTU_548 k__Eukaryota;p__Unclassified;c__Unclassified;o__Unclassified;f__Unclassified;g__Unclassified;s__uncultured fungus 1e-175 MF568721.1

OTU_549 k__Eukaryota;p__Unclassified;c__Unclassified;o__Unclassified;f__Unclassified;g__Unclassified;s__uncultured fungus 0.0 KX194723.1

OTU_55 k__Eukaryota;p__Ascomycota;c__Sordariomycetes;o__Hypocreales;f__Nectriaceae;g__Fusarium;s__Fusarium acuminatum 2e-174 MG572461.1

OTU_550 k__Eukaryota;p__Unclassified;c__Unclassified;o__Unclassified;f__Unclassified;g__Unclassified;s__uncultured fungus 1e-169 KX195312.1

OTU_551 k__Eukaryota;p__Ascomycota;c__Unclassified;o__Unclassified;f__Unclassified;g__Unclassified;s__uncultured Ascomycota 6e-122 FJ552927.1

OTU_552 k__Eukaryota;p__Ascomycota;c__Dothideomycetes;o__Pleosporales;f__Phaeosphaeriaceae;g__Paraphoma;s__Paraphoma chrysanthemicola 3e-161 KP690076.1

OTU_553 k__Eukaryota;p__Ascomycota;c__Leotiomycetes;o__Unclassified;f__Unclassified;g__Unclassified;s__Leotiomycetes sp. genotype 672 JMUR-2016 1e-157 KX909226.1

OTU_554 k__Eukaryota;p__Unclassified;c__Unclassified;o__Unclassified;f__Unclassified;g__Unclassified;s__uncultured fungus 1e-154 EF505187.1

OTU_555 k__Eukaryota;p__Unclassified;c__Unclassified;o__Unclassified;f__Unclassified;g__Unclassified;s__fungal endophyte 3e-133 KF435160.1

OTU_556 k__Eukaryota;p__Basidiomycota;c__Tremellomycetes;o__Tremellales;f__Bulleribasidiaceae;g__Vishniacozyma;s__Vishniacozyma carnescens 2e-124 KX009439.1

OTU_557 k__Eukaryota;p__Basidiomycota;c__Agaricomycetes;o__Agaricales;f__Cortinariaceae;g__Unclassified;s__uncultured Cortinarius 0.0 KU727192.1

OTU_558 k__Eukaryota;p__Ascomycota;c__Saccharomycetes;o__Saccharomycetales;f__Saccharomycetaceae;g__Kazachstania;s__Kazachstania bulderi 0.0 JQ808009.1

OTU_559 k__Eukaryota;p__Mucoromycota;c__Glomeromycetes;o__Archaeosporales;f__Archaeosporaceae;g__Archaeospora;s__Archaeospora sp. 8e-168 KP756479.1

OTU_56 k__Eukaryota;p__Basidiomycota;c__Microbotryomycetes;o__Sporidiobolales;f__Sporidiobolaceae;g__Sporobolomyces;s__Sporobolomyces roseus 0.0 KY495743.1

OTU_560 k__Eukaryota;p__Unclassified;c__Unclassified;o__Unclassified;f__Unclassified;g__Unclassified;s__uncultured fungus 1e-169 FN397180.1

OTU_561 k__Eukaryota;p__Ascomycota;c__Eurotiomycetes;o__Eurotiales;f__Aspergillaceae;g__Aspergillus;s__Aspergillus sp. 8e-174 KY379630.1

OTU_562 k__Eukaryota;p__Unclassified;c__Unclassified;o__Unclassified;f__Unclassified;g__Unclassified;s__uncultured fungus 8e-165 MF569688.1

OTU_563 k__Eukaryota;p__Basidiomycota;c__Tremellomycetes;o__Cystofilobasidiales;f__Cystofilobasidiaceae;g__Cystofilobasidium;s__Cystofilobasidium infirmominiatum 0.0 KY103173.1

OTU_564 k__Eukaryota;p__Unclassified;c__Unclassified;o__Unclassified;f__Unclassified;g__Unclassified;s__uncultured fungus 3e-170 KC965680.1

OTU_565 k__Eukaryota;p__Unclassified;c__Unclassified;o__Unclassified;f__Unclassified;g__Unclassified;s__uncultured fungus 4e-68 KX195328.1

OTU_566 k__Eukaryota;p__Ascomycota;c__Sordariomycetes;o__Xylariales;f__Diatrypaceae;g__Diatrypella;s__Diatrypella sp. 3e-71 KY069207.1

OTU_567 k__Eukaryota;p__Unclassified;c__Unclassified;o__Unclassified;f__Unclassified;g__Unclassified;s__uncultured fungus 4e-157 MF976169.1

OTU_568 k__Eukaryota;p__Ascomycota;c__Dothideomycetes;o__Dothideales;f__Dothioraceae;g__Dothiora;s__Dothiora pyrenophora 1e-166 KY929146.1

OTU_569 k__Eukaryota;p__Ascomycota;c__Leotiomycetes;o__Helotiales;f__Sclerotiniaceae;g__Botrytis;s__Botrytis cinerea 1e-135 KY419551.1

OTU_57 k__Eukaryota;p__Unclassified;c__Unclassified;o__Unclassified;f__Unclassified;g__Unclassified;s__uncultured fungus 2e-106 LT604510.1

OTU_570 k__Eukaryota;p__Unclassified;c__Unclassified;o__Unclassified;f__Unclassified;g__Unclassified;s__uncultured fungus 2e-81 KU536774.1

OTU_571 k__Eukaryota;p__Basidiomycota;c__Agaricomycetes;o__Agaricales;f__Inocybaceae;g__Inocybe;s__Inocybe lanatodisca 3e-167 KX897449.1

OTU_572 k__Eukaryota;p__Ascomycota;c__Sordariomycetes;o__Hypocreales;f__Unclassified;g__Acremonium;s__Acremonium persicinum 9e-180 JX524288.1

OTU_573 k__Eukaryota;p__Unclassified;c__Unclassified;o__Unclassified;f__Unclassified;g__Unclassified;s__uncultured fungus 7e-128 MF569322.1

OTU_574 k__Eukaryota;p__Ascomycota;c__Unclassified;o__Unclassified;f__Unclassified;g__Unclassified;s__uncultured Ascomycota 4e-166 HM239792.1

OTU_575 k__Eukaryota;p__Unclassified;c__Unclassified;o__Unclassified;f__Unclassified;g__Unclassified;s__uncultured fungus 0.0 KX826865.1

OTU_576 k__Eukaryota;p__Unclassified;c__Unclassified;o__Unclassified;f__Unclassified;g__Unclassified;s__uncultured fungus 3e-142 KF800545.1

OTU_577 k__Eukaryota;p__Unclassified;c__Unclassified;o__Unclassified;f__Unclassified;g__Unclassified;s__uncultured fungus 1e-175 MF569912.1

OTU_578 k__Eukaryota;p__Unclassified;c__Unclassified;o__Unclassified;f__Unclassified;g__Unclassified;s__uncultured fungus 5e-135 JX043072.1

OTU_579 k__Bacteria;p__Proteobacteria;c__Alphaproteobacteria;o__Rhodospirillales;f__Acetobacteraceae;g__Gluconobacter;s__Gluconobacter oxydans 6e-91 LT900339.1

OTU_58 k__Eukaryota;p__Unclassified;c__Unclassified;o__Unclassified;f__Unclassified;g__Unclassified;s__uncultured fungus 7e-168 KX515792.1

OTU_580 k__Eukaryota;p__Ascomycota;c__Leotiomycetes;o__Helotiales;f__Hyaloscyphaceae;g__Arachnopeziza;s__Arachnopeziza aurata 3e-145 KY228700.1

OTU_581 k__Eukaryota;p__Ascomycota;c__Leotiomycetes;o__Helotiales;f__Unclassified;g__Unclassified;s__Helotiales sp. 4e-129 KY228523.1

OTU_582 k__Eukaryota;p__Basidiomycota;c__Exobasidiomycetes;o__Tilletiales;f__Tilletiaceae;g__Tilletia;s__Tilletia moliniae 0.0 EU659137.1

OTU_583 k__Eukaryota;p__Ascomycota;c__Unclassified;o__Unclassified;f__Unclassified;g__Unclassified;s__Ascomycota sp. 4e-92 KX611054.1

OTU_584 k__Eukaryota;p__Ascomycota;c__Sordariomycetes;o__Xylariales;f__Microdochiaceae;g__Microdochium;s__Microdochium nivale 8e-174 KJ780751.1

OTU_585 k__Eukaryota;p__Ascomycota;c__Leotiomycetes;o__Helotiales;f__Unclassified;g__Unclassified;s__uncultured Helotiales 1e-156 KT581774.1

OTU_586 k__Eukaryota;p__Ascomycota;c__Eurotiomycetes;o__Eurotiales;f__Aspergillaceae;g__Aspergillus;s__Aspergillus fumigatiaffinis 5e-172 KY042104.1

OTU_587 k__Eukaryota;p__Unclassified;c__Unclassified;o__Unclassified;f__Unclassified;g__Unclassified;s__uncultured fungus 9e-155 JQ666600.1

OTU_588 k__Eukaryota;p__Basidiomycota;c__Wallemiomycetes;o__Wallemiales;f__Unclassified;g__Unclassified;s__uncultured Wallemiales 5e-46 MF483887.1

OTU_589 k__Eukaryota;p__Ascomycota;c__Eurotiomycetes;o__Eurotiales;f__Aspergillaceae;g__Penicillium;s__Penicillium sp. 3e-170 KY092669.1

OTU_59 k__Eukaryota;p__Ascomycota;c__Saccharomycetes;o__Saccharomycetales;f__Saccharomycopsidaceae;g__Saccharomycopsis;s__Saccharomycopsis crataegensis 0.0 KY495772.1

OTU_590 k__Eukaryota;p__Unclassified;c__Unclassified;o__Unclassified;f__Unclassified;g__Unclassified;s__fungal sp. mh1129.6 1e-79 GQ996165.1

OTU_591 k__Eukaryota;p__Ascomycota;c__Sordariomycetes;o__Xylariales;f__Sporocadaceae;g__Pestalotiopsis;s__Pestalotiopsis sp. CJL-2014 2e-168 KJ542337.1

OTU_592 k__Eukaryota;p__Unclassified;c__Unclassified;o__Unclassified;f__Unclassified;g__Unclassified;s__uncultured fungus 1e-166 MF568926.1

OTU_593 k__Eukaryota;p__Unclassified;c__Unclassified;o__Unclassified;f__Unclassified;g__Unclassified;s__fungal sp. 1e-166 KY775916.1

OTU_594 k__Eukaryota;p__Ascomycota;c__Dothideomycetes;o__Pleosporales;f__Leptosphaeriaceae;g__Unclassified;s__uncultured Leptosphaeria 4e-95 HM136635.1

OTU_595 k__Eukaryota;p__Ascomycota;c__Sordariomycetes;o__Sordariales;f__Chaetomiaceae;g__Arcopilus;s__Arcopilus flavigenus 7e-171 KX976587.1

OTU_596 k__Eukaryota;p__Ascomycota;c__Sordariomycetes;o__Sordariales;f__Chaetomiaceae;g__Humicola;s__Humicola sp. CY186 2e-165 HQ608016.1

OTU_597 k__Eukaryota;p__Unclassified;c__Unclassified;o__Unclassified;f__Unclassified;g__Unclassified;s__uncultured fungus 2e-152 EU144795.1

OTU_598 k__Eukaryota;p__Unclassified;c__Unclassified;o__Unclassified;f__Unclassified;g__Unclassified;s__uncultured fungus 2e-162 KC966319.1

OTU_599 k__Eukaryota;p__Ascomycota;c__Saccharomycetes;o__Saccharomycetales;f__Saccharomycetaceae;g__Zygosaccharomyces;s__Zygosaccharomyces parabailii 0.0 CP019500.1

OTU_6 k__Eukaryota;p__Ascomycota;c__Leotiomycetes;o__Helotiales;f__Sclerotiniaceae;g__Botrytis;s__Botrytis cinerea 2e-162 KY419551.1

OTU_60 k__Eukaryota;p__Unclassified;c__Unclassified;o__Unclassified;f__Unclassified;g__Unclassified;s__fungal sp. 603 AI-2013 7e-168 KC662236.1

OTU_600 k__Eukaryota;p__Ascomycota;c__Dothideomycetes;o__Dothideales;f__Saccotheciaceae;g__Aureobasidium;s__Aureobasidium pullulans 8e-146 KF887089.1

OTU_601 k__Eukaryota;p__Ascomycota;c__Dothideomycetes;o__Capnodiales;f__Mycosphaerellaceae;g__Virosphaerella;s__Virosphaerella irregularis 4e-126 EU882110.1

OTU_602 k__Eukaryota;p__Ascomycota;c__Saccharomycetes;o__Saccharomycetales;f__Saccharomycetaceae;g__Eremothecium;s__Eremothecium coryli 0.0 KJ562859.1

OTU_603 k__Eukaryota;p__Basidiomycota;c__Agaricomycetes;o__Agaricales;f__Bolbitiaceae;g__Conocybe;s__Conocybe lobauensis 0.0 JX968176.1

OTU_604 k__Eukaryota;p__Unclassified;c__Unclassified;o__Unclassified;f__Unclassified;g__Unclassified;s__uncultured fungus 2e-143 MF570459.1

OTU_605 k__Eukaryota;p__Ascomycota;c__Dothideomycetes;o__Pleosporales;f__Unclassified;g__Unclassified;s__Pleosporales sp. 1e-157 KY228508.1

OTU_606 k__Eukaryota;p__Unclassified;c__Unclassified;o__Unclassified;f__Unclassified;g__Unclassified;s__uncultured fungus 7e-08 MF570568.1

OTU_607 k__Eukaryota;p__Unclassified;c__Unclassified;o__Unclassified;f__Unclassified;g__Unclassified;s__uncultured fungus 7e-125 KC965730.1

OTU_608 k__Eukaryota;p__Ascomycota;c__Dothideomycetes;o__Capnodiales;f__Mycosphaerellaceae;g__Mycocentrospora;s__Mycocentrospora acerina 2e-168 KU341523.1

OTU_609 k__Eukaryota;p__Unclassified;c__Unclassified;o__Unclassified;f__Unclassified;g__Unclassified;s__uncultured fungus 0.0 KX196098.1

OTU_61 k__Eukaryota;p__Ascomycota;c__Eurotiomycetes;o__Eurotiales;f__Aspergillaceae;g__Aspergillus;s__Aspergillus niger 5e-166 KX622774.1

OTU_610 k__Eukaryota;p__Ascomycota;c__Leotiomycetes;o__Helotiales;f__Unclassified;g__Unclassified;s__Helotiales sp. 1e-156 KY228672.1

OTU_611 k__Eukaryota;p__Basidiomycota;c__Agaricomycetes;o__Agaricales;f__Hygrophoraceae;g__Unclassified;s__uncultured Hygrocybe 0.0 KX115618.1

OTU_612 k__Eukaryota;p__Basidiomycota;c__Tremellomycetes;o__Tremellales;f__Bulleribasidiaceae;g__Dioszegia;s__Dioszegia takashimae 7e-100 DQ003332.1

OTU_613 k__Eukaryota;p__Ascomycota;c__Eurotiomycetes;o__Eurotiales;f__Aspergillaceae;g__Aspergillus;s__Aspergillus aculeatinus 8e-143 MG543743.1

OTU_614 k__Eukaryota;p__Basidiomycota;c__Tremellomycetes;o__Tremellales;f__Bulleribasidiaceae;g__Dioszegia;s__Dioszegia sp. YM24626 2e-161 JQ320369.1

OTU_615 k__Eukaryota;p__Basidiomycota;c__Agaricomycetes;o__Agaricales;f__Schizophyllaceae;g__Schizophyllum;s__Schizophyllum commune 0.0 KY082748.1

OTU_616 k__Eukaryota;p__Unclassified;c__Unclassified;o__Unclassified;f__Unclassified;g__Unclassified;s__uncultured fungus 7e-168 MF569370.1

OTU_617 k__Eukaryota;p__Basidiomycota;c__Agaricomycetes;o__Agaricales;f__Cortinariaceae;g__Cortinarius;s__Cortinarius sp. 0.0 MG552979.1

OTU_618 k__Eukaryota;p__Ascomycota;c__Saccharomycetes;o__Saccharomycetales;f__Debaryomycetaceae;g__Debaryomyces;s__Debaryomyces sp. NYNU 16766 0.0 KY213815.1

OTU_619 k__Eukaryota;p__Ascomycota;c__Sordariomycetes;o__Glomerellales;f__Glomerellaceae;g__Colletotrichum;s__Colletotrichum gloeosporioides 1e-92 KX906602.1

OTU_62 k__Eukaryota;p__Unclassified;c__Unclassified;o__Unclassified;f__Unclassified;g__Unclassified;s__uncultured fungus 1e-98 MF571140.1

OTU_620 k__Eukaryota;p__Ascomycota;c__Leotiomycetes;o__Helotiales;f__Unclassified;g__Unclassified;s__Helotiales sp. 3e-136 KY228788.1

OTU_621 k__Eukaryota;p__Basidiomycota;c__Microbotryomycetes;o__Sporidiobolales;f__Sporidiobolaceae;g__Rhodotorula;s__Rhodotorula graminis 2e-177 KY037856.1

OTU_622 k__Eukaryota;p__Ascomycota;c__Dothideomycetes;o__Pleosporales;f__Pleosporaceae;g__Alternaria;s__Alternaria sp. 2e-171 KX925567.1

OTU_623 k__Eukaryota;p__Unclassified;c__Unclassified;o__Unclassified;f__Unclassified;g__Unclassified;s__uncultured fungus 2e-137 KF617709.1

OTU_624 k__Eukaryota;p__Ascomycota;c__Sordariomycetes;o__Microascales;f__Microascaceae;g__Wardomyces;s__Wardomyces moseri 4e-114 LN850995.1

OTU_625 k__Eukaryota;p__Ascomycota;c__Sordariomycetes;o__Glomerellales;f__Glomerellaceae;g__Colletotrichum;s__Colletotrichum magnum 5e-169 MG542874.1

OTU_626 k__Bacteria;p__Proteobacteria;c__Alphaproteobacteria;o__Rhodospirillales;f__Acetobacteraceae;g__Gluconobacter;s__Gluconobacter oxydans 1e-135 CP004373.1

OTU_627 k__Eukaryota;p__Unclassified;c__Unclassified;o__Unclassified;f__Unclassified;g__Unclassified;s__uncultured fungus 1e-12 MF570267.1

OTU_628 k__Eukaryota;p__Unclassified;c__Unclassified;o__Unclassified;f__Unclassified;g__Unclassified;s__uncultured fungus 0.0 JX174765.1

OTU_629 k__Eukaryota;p__Basidiomycota;c__Agaricomycetes;o__Agaricales;f__Bolbitiaceae;g__Conocybe;s__Conocybe aff. ochrostriata NL-0830 3e-170 JX968236.1

OTU_63 k__Eukaryota;p__Basidiomycota;c__Tremellomycetes;o__Filobasidiales;f__Filobasidiaceae;g__Filobasidium;s__Filobasidium chernovii 0.0 FN400936.1

OTU_630 k__Eukaryota;p__Ascomycota;c__Unclassified;o__Unclassified;f__Unclassified;g__Unclassified;s__fungal endophyte MUT 2727 2e-155 AF373063.1

OTU_631 k__Eukaryota;p__Ascomycota;c__Sordariomycetes;o__Glomerellales;f__Glomerellaceae;g__Colletotrichum;s__Colletotrichum gloeosporioides 3e-145 KX906602.1

OTU_632 k__Eukaryota;p__Unclassified;c__Unclassified;o__Unclassified;f__Unclassified;g__Unclassified;s__fungal sp. M38 XS-2012 1e-160 JX298911.1

OTU_633 k__Eukaryota;p__Ascomycota;c__Sordariomycetes;o__Microascales;f__Halosphaeriaceae;g__Unclassified;s__uncultured Halosphaeriaceae 8e-103 DQ900988.1

OTU_634 k__Eukaryota;p__Unclassified;c__Unclassified;o__Unclassified;f__Unclassified;g__Unclassified;s__uncultured fungus 4e-151 KC965947.1

OTU_635 k__Eukaryota;p__Ascomycota;c__Dothideomycetes;o__Pleosporales;f__Phaeosphaeriaceae;g__Unclassified;s__Phaeosphaeriaceae sp. AK-2017b 1e-153 MF684869.1

OTU_636 k__Eukaryota;p__Unclassified;c__Unclassified;o__Unclassified;f__Unclassified;g__Unclassified;s__uncultured fungus 1e-172 KP897575.1

OTU_637 k__Eukaryota;p__Basidiomycota;c__Tremellomycetes;o__Tremellales;f__Cryptococcaceae;g__Kwoniella;s__Kwoniella mangrovensis 0.0 EF174042.1

OTU_638 k__Eukaryota;p__Unclassified;c__Unclassified;o__Unclassified;f__Unclassified;g__Unclassified;s__uncultured fungus 9e-81 KT758090.1

OTU_639 k__Eukaryota;p__Ascomycota;c__Sordariomycetes;o__Glomerellales;f__Glomerellaceae;g__Colletotrichum;s__Colletotrichum gloeosporioides 2e-128 KX906602.1

OTU_64 k__Eukaryota;p__Ascomycota;c__Sordariomycetes;o__Hypocreales;f__Unclassified;g__Trichothecium;s__Trichothecium roseum 0.0 MF288732.1

OTU_640 k__Eukaryota;p__Ascomycota;c__Dothideomycetes;o__Unclassified;f__Unclassified;g__Unclassified;s__Dothideomycetes sp. genotype 283 2e-156 JQ760107.1

OTU_641 k__Eukaryota;p__Basidiomycota;c__Agaricomycetes;o__Russulales;f__Hericiaceae;g__Hericium;s__Hericium erinaceus 0.0 KJ920141.1

OTU_642 k__Eukaryota;p__Ascomycota;c__Dothideomycetes;o__Dothideales;f__Saccotheciaceae;g__Aureobasidium;s__Aureobasidium pullulans 4e-114 KY294714.1

OTU_643 k__Eukaryota;p__Unclassified;c__Unclassified;o__Unclassified;f__Unclassified;g__Unclassified;s__uncultured fungus 1e-156 JQ312752.1

OTU_644 k__Eukaryota;p__Unclassified;c__Unclassified;o__Unclassified;f__Unclassified;g__Unclassified;s__fungal endophyte 4e-06 KF673692.1

OTU_645 k__Eukaryota;p__Ascomycota;c__Dothideomycetes;o__Venturiales;f__Sympoventuriaceae;g__Ochroconis;s__Ochroconis mirabilis 0.0 LM644520.1

OTU_646 k__Eukaryota;p__Unclassified;c__Unclassified;o__Unclassified;f__Unclassified;g__Unclassified;s__uncultured fungus 1e-95 KU537157.1

OTU_647 k__Eukaryota;p__Unclassified;c__Unclassified;o__Unclassified;f__Unclassified;g__Unclassified;s__uncultured fungus 0.0 KX192450.1

OTU_648 k__Eukaryota;p__Unclassified;c__Unclassified;o__Unclassified;f__Unclassified;g__Unclassified;s__uncultured fungus 1e-110 GU307142.1

OTU_649 k__Eukaryota;p__Ascomycota;c__Sordariomycetes;o__Glomerellales;f__Glomerellaceae;g__Colletotrichum;s__Colletotrichum siamense 1e-61 KY319114.1

OTU_65 k__Eukaryota;p__Unclassified;c__Unclassified;o__Unclassified;f__Unclassified;g__Unclassified;s__uncultured fungus 4e-163 KY823599.1

OTU_650 k__Eukaryota;p__Unclassified;c__Unclassified;o__Unclassified;f__Unclassified;g__Unclassified;s__uncultured fungus 4e-06 KX193383.1

OTU_651 k__Eukaryota;p__Ascomycota;c__Dothideomycetes;o__Pleosporales;f__Leptosphaeriaceae;g__Sphaerellopsis;s__Sphaerellopsis filum 6e-159 JF811430.1

OTU_652 k__Eukaryota;p__Unclassified;c__Unclassified;o__Unclassified;f__Unclassified;g__Unclassified;s__uncultured fungus 0.0 KX192443.1

OTU_653 k__Eukaryota;p__Basidiomycota;c__Agaricomycetes;o__Agaricales;f__Bolbitiaceae;g__Descolea;s__Descolea maculata 0.0 KY654754.1

OTU_654 k__Eukaryota;p__Ascomycota;c__Dothideomycetes;o__Unclassified;f__Micropeltidaceae;g__Stomiopeltis;s__Stomiopeltis betulae 8e-69 GU214701.1

OTU_655 k__Eukaryota;p__Ascomycota;c__Dothideomycetes;o__Dothideales;f__Saccotheciaceae;g__Aureobasidium;s__Aureobasidium pullulans 3e-142 KT898763.1

OTU_656 k__Eukaryota;p__Ascomycota;c__Dothideomycetes;o__Capnodiales;f__Mycosphaerellaceae;g__Septoria;s__cf. Septoria sp. CPC 19311 6e-51 KF251241.1

OTU_657 k__Eukaryota;p__Unclassified;c__Unclassified;o__Unclassified;f__Unclassified;g__Unclassified;s__uncultured fungus 6e-150 MF569682.1

OTU_658 k__Eukaryota;p__Unclassified;c__Unclassified;o__Unclassified;f__Unclassified;g__Unclassified;s__uncultured fungus 4e-154 KF800327.1

OTU_659 k__Eukaryota;p__Ascomycota;c__Unclassified;o__Unclassified;f__Unclassified;g__Unclassified;s__uncultured Ascomycota 4e-129 MG206616.1

OTU_66 k__Eukaryota;p__Unclassified;c__Unclassified;o__Unclassified;f__Unclassified;g__Unclassified;s__uncultured fungus 3e-161 MF570611.1

OTU_660 k__Eukaryota;p__Unclassified;c__Unclassified;o__Unclassified;f__Unclassified;g__Unclassified;s__uncultured fungus 2e-128 KP897289.1

OTU_661 k__Bacteria;p__Proteobacteria;c__Alphaproteobacteria;o__Rhodospirillales;f__Acetobacteraceae;g__Acetobacter;s__Acetobacter pasteurianus 7e-168 AP014881.1

OTU_662 k__Eukaryota;p__Ascomycota;c__Sordariomycetes;o__Xylariales;f__Apiosporaceae;g__Arthrinium;s__Arthrinium rasikravindrae 3e-167 KX034336.1

OTU_663 k__Eukaryota;p__Ascomycota;c__Eurotiomycetes;o__Eurotiales;f__Unclassified;g__Unclassified;s__uncultured Eurotiales 6e-156 HQ389458.1

OTU_664 k__Eukaryota;p__Unclassified;c__Unclassified;o__Unclassified;f__Unclassified;g__Unclassified;s__uncultured fungus 4e-129 GQ851635.1

OTU_665 k__Eukaryota;p__Ascomycota;c__Saccharomycetes;o__Saccharomycetales;f__Trichomonascaceae;g__Zygoascus;s__Zygoascus meyerae 0.0 KY495726.1

OTU_666 k__Eukaryota;p__Basidiomycota;c__Agaricomycetes;o__Polyporales;f__Polyporaceae;g__Ganoderma;s__Ganoderma sichuanense 0.0 KY364250.1

OTU_667 k__Eukaryota;p__Ascomycota;c__Saccharomycetes;o__Saccharomycetales;f__Pichiaceae;g__Pichia;s__Pichia sp. feni 106 7e-134 KP223717.1

OTU_668 k__Eukaryota;p__Ascomycota;c__Dothideomycetes;o__Dothideales;f__Saccotheciaceae;g__Aureobasidium;s__Aureobasidium pullulans 7e-131 KY294714.1

OTU_67 k__Eukaryota;p__Unclassified;c__Unclassified;o__Unclassified;f__Unclassified;g__Unclassified;s__fungal sp. 2e-168 KY776384.1

OTU_670 k__Eukaryota;p__Unclassified;c__Unclassified;o__Unclassified;f__Unclassified;g__Unclassified;s__uncultured fungus 2e-161 KX193543.1

OTU_671 k__Eukaryota;p__Ascomycota;c__Eurotiomycetes;o__Eurotiales;f__Aspergillaceae;g__Aspergillus;s__Aspergillus foetidus 2e-149 AJ280009.1

OTU_672 k__Eukaryota;p__Ascomycota;c__Dothideomycetes;o__Pleosporales;f__Pleosporaceae;g__Alternaria;s__Alternaria sp. C3_14 2e-174 KM246290.1

OTU_673 k__Eukaryota;p__Unclassified;c__Unclassified;o__Unclassified;f__Unclassified;g__Unclassified;s__uncultured fungus 8e-11 MF568708.1

OTU_674 k__Eukaryota;p__Ascomycota;c__Dothideomycetes;o__Dothideales;f__Saccotheciaceae;g__Aureobasidium;s__Aureobasidium pullulans 5e-141 HQ267773.1

OTU_675 k__Eukaryota;p__Ascomycota;c__Eurotiomycetes;o__Eurotiales;f__Aspergillaceae;g__Aspergillus;s__Aspergillus terreus 9e-180 MG250398.1

OTU_676 k__Eukaryota;p__Ascomycota;c__Eurotiomycetes;o__Eurotiales;f__Unclassified;g__Unclassified;s__uncultured Eurotiales 6e-113 HQ389458.1

OTU_677 k__Eukaryota;p__Basidiomycota;c__Agaricomycetes;o__Agaricales;f__Hygrophoraceae;g__Hygrocybe;s__Hygrocybe lepida 0.0 KF306333.1

OTU_678 k__Eukaryota;p__Ascomycota;c__Saccharomycetes;o__Saccharomycetales;f__Saccharomycetaceae;g__Lachancea;s__Lachancea thermotolerans 0.0 KY207383.1

OTU_679 k__Eukaryota;p__Ascomycota;c__Dothideomycetes;o__Pleosporales;f__Phaeosphaeriaceae;g__Parastagonospora;s__Parastagonospora nodorum 5e-33 CP022842.1

OTU_68 k__Eukaryota;p__Unclassified;c__Unclassified;o__Unclassified;f__Unclassified;g__Unclassified;s__fungal sp. 2e-165 MG515308.1

OTU_680 k__Eukaryota;p__Unclassified;c__Unclassified;o__Unclassified;f__Unclassified;g__Unclassified;s__uncultured fungus 1e-160 KX193196.1

OTU_681 k__Eukaryota;p__Ascomycota;c__Dothideomycetes;o__Dothideales;f__Saccotheciaceae;g__Aureobasidium;s__Aureobasidium pullulans 5e-144 KY294714.1

OTU_682 k__Bacteria;p__Proteobacteria;c__Alphaproteobacteria;o__Rhodospirillales;f__Acetobacteraceae;g__Gluconobacter;s__Gluconobacter oxydans 4e-154 CP004373.1

OTU_683 k__Eukaryota;p__Basidiomycota;c__Unclassified;o__Unclassified;f__Unclassified;g__Unclassified;s__uncultured Basidiomycota 0.0 DQ672324.1

OTU_684 k__Eukaryota;p__Ascomycota;c__Dothideomycetes;o__Pleosporales;f__Pleosporaceae;g__Alternaria;s__Alternaria chlamydosporigena 6e-159 KX343144.1

OTU_685 k__Eukaryota;p__Ascomycota;c__Dothideomycetes;o__Pleosporales;f__Didymellaceae;g__Peyronellaea;s__Peyronellaea sp. 1e-150 MG250460.1

OTU_686 k__Eukaryota;p__Ascomycota;c__Sordariomycetes;o__Glomerellales;f__Glomerellaceae;g__Colletotrichum;s__Colletotrichum sp. 2e-66 KX400812.1

OTU_687 k__Eukaryota;p__Ascomycota;c__Leotiomycetes;o__Helotiales;f__Sclerotiniaceae;g__Botrytis;s__Botrytis cinerea 3e-139 KY419551.1

OTU_688 k__Eukaryota;p__Basidiomycota;c__Agaricomycetes;o__Atheliales;f__Atheliaceae;g__Piloderma;s__Piloderma sphaerosporum 0.0 MF926559.1

OTU_689 k__Eukaryota;p__Unclassified;c__Unclassified;o__Unclassified;f__Unclassified;g__Unclassified;s__uncultured fungus 7e-165 KX195871.1

OTU_69 k__Eukaryota;p__Ascomycota;c__Sordariomycetes;o__Hypocreales;f__Nectriaceae;g__Fusarium;s__Fusarium sp. 7e-165 MG575045.1

OTU_690 k__Eukaryota;p__Ascomycota;c__Leotiomycetes;o__Unclassified;f__Unclassified;g__Hyphozyma;s__Hyphozyma sp. RF-2017a 4e-163 KY695000.1

OTU_691 k__Eukaryota;p__Unclassified;c__Unclassified;o__Unclassified;f__Unclassified;g__Unclassified;s__uncultured fungus 1e-157 KP889977.1

OTU_692 k__Eukaryota;p__Ascomycota;c__Sordariomycetes;o__Hypocreales;f__Unclassified;g__Emericellopsis;s__Emericellopsis sp. 3e-176 KX381180.1

OTU_693 k__Eukaryota;p__Ascomycota;c__Dothideomycetes;o__Capnodiales;f__Mycosphaerellaceae;g__Polythrincium;s__Polythrincium trifolii 0.0 KY554953.1

OTU_694 k__Eukaryota;p__Unclassified;c__Unclassified;o__Unclassified;f__Unclassified;g__Unclassified;s__uncultured fungus 9e-137 KF780606.1

OTU_695 k__Eukaryota;p__Ascomycota;c__Dothideomycetes;o__Pleosporales;f__Sporormiaceae;g__Sporormia;s__Sporormia sp. BAB-4552 4e-157 KT199721.1

OTU_696 k__Eukaryota;p__Ascomycota;c__Unclassified;o__Unclassified;f__Unclassified;g__Unclassified;s__Ascomycota sp. L310 3e-167 KU535795.1

OTU_697 k__Eukaryota;p__Unclassified;c__Unclassified;o__Unclassified;f__Unclassified;g__Unclassified;s__uncultured fungus 1e-135 KX223155.1

OTU_698 k__Eukaryota;p__Ascomycota;c__Dothideomycetes;o__Pleosporales;f__Unclassified;g__Unclassified;s__Pleosporales sp. AFCN990 6e-156 LC085192.1

OTU_699 k__Eukaryota;p__Mucoromycota;c__Unclassified;o__Mucorales;f__Mucoraceae;g__Mucor;s__Mucor hiemalis 0.0 MG385097.1

OTU_7 k__Eukaryota;p__Basidiomycota;c__Microbotryomycetes;o__Sporidiobolales;f__Sporidiobolaceae;g__Rhodotorula;s__Rhodotorula glutinis 0.0 KY611826.1

OTU_70 k__Eukaryota;p__Ascomycota;c__Dothideomycetes;o__Pleosporales;f__Pleosporaceae;g__Alternaria;s__Alternaria alternata 3e-170 MF141014.1

OTU_700 k__Eukaryota;p__Ascomycota;c__Saccharomycetes;o__Saccharomycetales;f__Pichiaceae;g__Pichia;s__Pichia manshurica 1e-147 FM199959.1

OTU_701 k__Eukaryota;p__Unclassified;c__Unclassified;o__Unclassified;f__Unclassified;g__Unclassified;s__fungal endophyte 2e-174 KY038594.1

OTU_702 k__Eukaryota;p__Unclassified;c__Unclassified;o__Unclassified;f__Unclassified;g__Unclassified;s__uncultured fungus 5e-15 KP889943.1

OTU_703 k__Eukaryota;p__Unclassified;c__Unclassified;o__Unclassified;f__Unclassified;g__Unclassified;s__uncultured fungus 6e-132 MF570145.1

OTU_704 k__Eukaryota;p__Ascomycota;c__Sordariomycetes;o__Hypocreales;f__Nectriaceae;g__Fusarium;s__Fusarium lateritium 3e-170 KT192395.1

OTU_705 k__Eukaryota;p__Ascomycota;c__Leotiomycetes;o__Helotiales;f__Leotiaceae;g__Pezoloma;s__Pezoloma ericae 6e-162 JQ711893.1

OTU_706 k__Eukaryota;p__Unclassified;c__Unclassified;o__Unclassified;f__Unclassified;g__Unclassified;s__uncultured fungus 0.0 LT608086.1

OTU_707 k__Eukaryota;p__Unclassified;c__Unclassified;o__Unclassified;f__Unclassified;g__Unclassified;s__uncultured fungus 1e-153 KC588585.1

OTU_708 k__Eukaryota;p__Ascomycota;c__Eurotiomycetes;o__Eurotiales;f__Aspergillaceae;g__Aspergillus;s__Aspergillus niger 1e-160 KF305745.1

OTU_709 k__Eukaryota;p__Ascomycota;c__Eurotiomycetes;o__Eurotiales;f__Aspergillaceae;g__Aspergillus;s__Aspergillus penicillioides 0.0 KY087763.1

OTU_71 k__Eukaryota;p__Ascomycota;c__Dothideomycetes;o__Pleosporales;f__Pleosporaceae;g__Stemphylium;s__Stemphylium vesicarium 3e-170 MG065799.1

OTU_710 k__Eukaryota;p__Basidiomycota;c__Tremellomycetes;o__Tremellales;f__Rhynchogastremataceae;g__Papiliotrema;s__Papiliotrema taeanensis 0.0 KY104475.1

OTU_711 k__Eukaryota;p__Ascomycota;c__Dothideomycetes;o__Patellariales;f__Patellariaceae;g__Banhegyia;s__Banhegyia cf. setispora G.M. 2015-04-29.1 2e-88 KY654708.1

OTU_712 k__Eukaryota;p__Unclassified;c__Unclassified;o__Unclassified;f__Unclassified;g__Unclassified;s__uncultured fungus 1e-166 GU053799.1

OTU_713 k__Eukaryota;p__Ascomycota;c__Dothideomycetes;o__Pleosporales;f__Didymellaceae;g__Didymella;s__Didymella macrostoma 4e-151 DQ474094.1

OTU_714 k__Eukaryota;p__Basidiomycota;c__Tremellomycetes;o__Trichosporonales;f__Trichosporonaceae;g__Cryptococcus;s__Cryptococcus sp. SJ10L02 2e-158 FJ153172.1

OTU_715 k__Eukaryota;p__Ascomycota;c__Sordariomycetes;o__Hypocreales;f__Ophiocordycipitaceae;g__Hirsutella;s__Hirsutella sp. 5e-178 MG561950.1

OTU_716 k__Eukaryota;p__Ascomycota;c__Saccharomycetes;o__Saccharomycetales;f__Saccharomycodaceae;g__Hanseniaspora;s__Hanseniaspora sp. 0.0 KY977693.1

OTU_717 k__Eukaryota;p__Unclassified;c__Unclassified;o__Unclassified;f__Unclassified;g__Unclassified;s__uncultured fungus 1e-148 KX195142.1

OTU_718 k__Eukaryota;p__Unclassified;c__Unclassified;o__Unclassified;f__Unclassified;g__Unclassified;s__uncultured fungus 5e-150 MF570715.1

OTU_719 k__Eukaryota;p__Basidiomycota;c__Exobasidiomycetes;o__Microstromatales;f__Unclassified;g__Sympodiomycopsis;s__Sympodiomycopsis yantaiensis 0.0 KF246552.1

OTU_72 k__Eukaryota;p__Ascomycota;c__Dothideomycetes;o__Pleosporales;f__Pleosporaceae;g__Pithomyces;s__Pithomyces chartarum 3e-167 MG572383.1

OTU_720 k__Eukaryota;p__Ascomycota;c__Dothideomycetes;o__Pleosporales;f__Leptosphaeriaceae;g__Leptosphaeria;s__Leptosphaeria sclerotioides 3e-170 MF326616.1

OTU_721 k__Bacteria;p__Proteobacteria;c__Alphaproteobacteria;o__Rhodospirillales;f__Acetobacteraceae;g__Gluconobacter;s__Gluconobacter oxydans 4e-148 CP016328.1

OTU_722 k__Eukaryota;p__Unclassified;c__Unclassified;o__Unclassified;f__Unclassified;g__Unclassified;s__uncultured fungus 2e-168 FJ820830.1

OTU_723 k__Eukaryota;p__Ascomycota;c__Saccharomycetes;o__Saccharomycetales;f__Unclassified;g__Candida;s__Candida sp. G1 3e-173 JN636809.1

OTU_724 k__Eukaryota;p__Ascomycota;c__Sordariomycetes;o__Sordariales;f__Lasiosphaeriaceae;g__Lasiosphaeria;s__Lasiosphaeria lanuginosa 5e-144 AY587920.1

OTU_725 k__Eukaryota;p__Unclassified;c__Unclassified;o__Unclassified;f__Unclassified;g__Unclassified;s__uncultured fungus 0.0 JX984707.1

OTU_726 k__Eukaryota;p__Unclassified;c__Unclassified;o__Unclassified;f__Unclassified;g__Unclassified;s__fungal endophyte 8e-174 KY038592.1

OTU_727 k__Eukaryota;p__Unclassified;c__Unclassified;o__Unclassified;f__Unclassified;g__Unclassified;s__uncultured fungus 1e-138 KX193981.1

OTU_728 k__Eukaryota;p__Ascomycota;c__Dothideomycetes;o__Capnodiales;f__Cladosporiaceae;g__Cladosporium;s__Cladosporium sp. Tn-11 1e-135 LT604478.1

OTU_729 k__Eukaryota;p__Unclassified;c__Unclassified;o__Unclassified;f__Unclassified;g__Unclassified;s__uncultured fungus 0.0 MF571135.1

OTU_73 k__Eukaryota;p__Ascomycota;c__Sordariomycetes;o__Diaporthales;f__Valsaceae;g__Cytospora;s__Cytospora sp. 2e-174 KX774150.1

OTU_730 k__Eukaryota;p__Ascomycota;c__Sordariomycetes;o__Hypocreales;f__Nectriaceae;g__Fusarium;s__Fusarium oxysporum 7e-168 KY283781.1

OTU_731 k__Eukaryota;p__Unclassified;c__Unclassified;o__Unclassified;f__Unclassified;g__Unclassified;s__uncultured fungus 5e-178 KU164260.1

OTU_732 k__Eukaryota;p__Ascomycota;c__Leotiomycetes;o__Helotiales;f__Hyaloscyphaceae;g__Hyaloscypha;s__Hyaloscypha sp. A SS-2011 2e-159 JN943607.1

OTU_733 k__Eukaryota;p__Ascomycota;c__Leotiomycetes;o__Unclassified;f__Myxotrichaceae;g__Oidiodendron;s__Oidiodendron truncatum 9e-155 KC009296.1

OTU_734 k__Eukaryota;p__Unclassified;c__Unclassified;o__Unclassified;f__Unclassified;g__Unclassified;s__uncultured fungus 1e-06 MF571177.1

OTU_735 k__Eukaryota;p__Basidiomycota;c__Agaricomycetes;o__Agaricales;f__Tricholomataceae;g__Laccaria;s__Laccaria sp. GL-2017 0.0 LT716039.1

OTU_736 k__Eukaryota;p__Unclassified;c__Unclassified;o__Unclassified;f__Unclassified;g__Unclassified;s__uncultured Dikarya 4e-160 JQ247362.1

OTU_737 k__Eukaryota;p__Unclassified;c__Unclassified;o__Unclassified;f__Unclassified;g__Unclassified;s__fungal sp. 6e-79 KT923210.1

OTU_738 k__Eukaryota;p__Ascomycota;c__Dothideomycetes;o__Pleosporales;f__Phaeosphaeriaceae;g__Phaeosphaeria;s__Phaeosphaeria sp. 4e-157 MF120205.1

OTU_739 k__Eukaryota;p__Unclassified;c__Unclassified;o__Unclassified;f__Unclassified;g__Unclassified;s__uncultured fungus 2e-165 MF570460.1

OTU_74 k__Eukaryota;p__Basidiomycota;c__Tremellomycetes;o__Unclassified;f__Unclassified;g__Cryptococcus;s__Cryptococcus sp. SY2S01 6e-153 FJ153165.1

OTU_740 k__Eukaryota;p__Unclassified;c__Unclassified;o__Unclassified;f__Unclassified;g__Unclassified;s__fungal endophyte 4e-179 KF436234.1

OTU_741 k__Eukaryota;p__Unclassified;c__Unclassified;o__Unclassified;f__Unclassified;g__Unclassified;s__uncultured fungus 6e-162 MF568799.1

OTU_742 k__Eukaryota;p__Basidiomycota;c__Microbotryomycetes;o__Sporidiobolales;f__Sporidiobolaceae;g__Rhodotorula;s__Rhodotorula sp. CBS 10104 0.0 AJ876487.1

OTU_743 k__Eukaryota;p__Ascomycota;c__Dothideomycetes;o__Pleosporales;f__Unclassified;g__Unclassified;s__Pleosporales sp. 7 PV-2016 2e-131 KU933735.1

OTU_744 k__Eukaryota;p__Ascomycota;c__Dothideomycetes;o__Capnodiales;f__Mycosphaerellaceae;g__Pseudocercospora;s__Pseudocercospora carbonacea 4e-86 KC677897.1

OTU_745 k__Eukaryota;p__Unclassified;c__Unclassified;o__Unclassified;f__Unclassified;g__Unclassified;s__uncultured fungus 1e-12 MF569014.1

OTU_746 k__Eukaryota;p__Unclassified;c__Unclassified;o__Unclassified;f__Unclassified;g__Unclassified;s__uncultured fungus 6e-178 MF569442.1

OTU_747 k__Eukaryota;p__Unclassified;c__Unclassified;o__Unclassified;f__Unclassified;g__Unclassified;s__uncultured fungus 5e-12 KP889943.1

OTU_748 k__Eukaryota;p__Ascomycota;c__Dothideomycetes;o__Capnodiales;f__Cladosporiaceae;g__Cladosporium;s__Cladosporium tenuissimum 5e-147 MG572368.1

OTU_749 k__Eukaryota;p__Ascomycota;c__Dothideomycetes;o__Pleosporales;f__Leptosphaeriaceae;g__Leptosphaeria;s__Leptosphaeria biglobosa 2e-11 FO905684.1

OTU_75 k__Eukaryota;p__Ascomycota;c__Unclassified;o__Unclassified;f__Unclassified;g__Unclassified;s__uncultured Ascomycota 2e-155 FR682366.1

OTU_750 k__Eukaryota;p__Unclassified;c__Unclassified;o__Unclassified;f__Unclassified;g__Unclassified;s__uncultured fungus 4e-132 KX193248.1

OTU_751 k__Eukaryota;p__Basidiomycota;c__Tremellomycetes;o__Filobasidiales;f__Piskurozymaceae;g__Piskurozyma;s__Piskurozyma capsuligena 0.0 KY104661.1

OTU_752 k__Eukaryota;p__Ascomycota;c__Pezizomycetes;o__Unclassified;f__Unclassified;g__Unclassified;s__Pezizomycetes sp. 1e-160 KY977450.1

OTU_753 k__Eukaryota;p__Unclassified;c__Unclassified;o__Unclassified;f__Unclassified;g__Unclassified;s__uncultured fungus 0.0 KF617992.1

OTU_754 k__Eukaryota;p__Unclassified;c__Unclassified;o__Unclassified;f__Unclassified;g__Unclassified;s__uncultured fungus 9e-44 FJ362304.1

OTU_755 k__Eukaryota;p__Ascomycota;c__Sordariomycetes;o__Diaporthales;f__Diaporthaceae;g__Diaporthe;s__Diaporthe sp. 3e-164 KY011896.1

OTU_756 k__Eukaryota;p__Unclassified;c__Unclassified;o__Unclassified;f__Unclassified;g__Unclassified;s__uncultured fungus 0.0 KX193638.1

OTU_757 k__Eukaryota;p__Unclassified;c__Unclassified;o__Unclassified;f__Unclassified;g__Unclassified;s__uncultured fungus 5e-09 MF569057.1

OTU_758 k__Eukaryota;p__Ascomycota;c__Dothideomycetes;o__Pleosporales;f__Cucurbitariaceae;g__Pyrenochaeta;s__Pyrenochaeta sp. 4e-166 MF687678.1

OTU_759 k__Eukaryota;p__Ascomycota;c__Dothideomycetes;o__Pleosporales;f__Pleosporaceae;g__Alternaria;s__Alternaria alternata 1e-119 KY228355.1

OTU_76 k__Eukaryota;p__Unclassified;c__Unclassified;o__Unclassified;f__Unclassified;g__Unclassified;s__uncultured fungus 4e-160 KX514820.1

OTU_760 k__Eukaryota;p__Mucoromycota;c__Unclassified;o__Mucorales;f__Choanephoraceae;g__Choanephora;s__Choanephora cucurbitarum 5e-178 KY080447.1

OTU_761 k__Eukaryota;p__Unclassified;c__Unclassified;o__Unclassified;f__Unclassified;g__Unclassified;s__uncultured fungus 0.0 MF571090.1

OTU_762 k__Eukaryota;p__Unclassified;c__Unclassified;o__Unclassified;f__Unclassified;g__Unclassified;s__uncultured fungus 5e-144 KT328643.1

OTU_763 k__Eukaryota;p__Unclassified;c__Unclassified;o__Unclassified;f__Unclassified;g__Unclassified;s__uncultured fungus 1e-82 FJ237229.1

OTU_764 k__Eukaryota;p__Unclassified;c__Unclassified;o__Unclassified;f__Unclassified;g__Unclassified;s__uncultured fungus 3e-142 KX193279.1

OTU_765 k__Eukaryota;p__Unclassified;c__Unclassified;o__Unclassified;f__Unclassified;g__Unclassified;s__fungal sp. HS492 LX-2016 1e-163 KY496814.1

OTU_766 k__Eukaryota;p__Ascomycota;c__Sordariomycetes;o__Xylariales;f__Diatrypaceae;g__Diatrype;s__Diatrype stigma 1e-160 KX828152.1

OTU_767 k__Eukaryota;p__Basidiomycota;c__Tremellomycetes;o__Tremellales;f__Bulleribasidiaceae;g__Vishniacozyma;s__Vishniacozyma heimaeyensis 2e-152 KX096666.1

OTU_768 k__Eukaryota;p__Basidiomycota;c__Tremellomycetes;o__Tremellales;f__Bulleribasidiaceae;g__Vishniacozyma;s__Vishniacozyma carnescens 9e-121 MG250423.1

OTU_769 k__Eukaryota;p__Ascomycota;c__Unclassified;o__Unclassified;f__Unclassified;g__Unclassified;s__Ascomycota sp. OY30807 4e-163 FJ571431.1

OTU_77 k__Eukaryota;p__Unclassified;c__Unclassified;o__Unclassified;f__Unclassified;g__Unclassified;s__uncultured fungus 2e-140 KC766054.1

OTU_770 k__Eukaryota;p__Basidiomycota;c__Agaricomycetes;o__Agaricales;f__Inocybaceae;g__Inocybe;s__Inocybe alloumbrina 1e-151 KJ778850.1

OTU_771 k__Eukaryota;p__Mucoromycota;c__Unclassified;o__Mortierellales;f__Mortierellaceae;g__Mortierella;s__Mortierella globalpina 0.0 AB476420.1

OTU_772 k__Eukaryota;p__Ascomycota;c__Sordariomycetes;o__Unclassified;f__Unclassified;g__Unclassified;s__Sordariomycetes sp. 2e-162 KY413704.1

OTU_773 k__Eukaryota;p__Ascomycota;c__Dothideomycetes;o__Pleosporales;f__Pleosporaceae;g__Alternaria;s__Alternaria alternata 1e-147 KY014448.1

OTU_774 k__Eukaryota;p__Basidiomycota;c__Tremellomycetes;o__Trichosporonales;f__Trichosporonaceae;g__Cryptococcus;s__Cryptococcus sp. SJ8L06 1e-178 FJ153168.1

OTU_775 k__Eukaryota;p__Ascomycota;c__Dothideomycetes;o__Capnodiales;f__Cladosporiaceae;g__Cladosporium;s__Cladosporium tenuissimum 2e-155 MG572368.1

OTU_776 k__Eukaryota;p__Unclassified;c__Unclassified;o__Unclassified;f__Unclassified;g__Unclassified;s__uncultured fungus 7e-88 KX195086.1

OTU_777 k__Eukaryota;p__Ascomycota;c__Unclassified;o__Unclassified;f__Unclassified;g__Aporospora;s__Aporospora terricola 1e-163 KC460893.1

OTU_778 k__Eukaryota;p__Basidiomycota;c__Tremellomycetes;o__Tremellales;f__Naemateliaceae;g__Naematelia;s__Naematelia encephala 2e-168 KY037858.1

OTU_779 k__Eukaryota;p__Unclassified;c__Unclassified;o__Unclassified;f__Unclassified;g__Unclassified;s__fungal sp. CA151RZ 1e-49 KP403992.1

OTU_78 k__Eukaryota;p__Ascomycota;c__Dothideomycetes;o__Pleosporales;f__Didymellaceae;g__Phoma;s__Phoma sp. 4e-160 KY463477.1

OTU_780 k__Eukaryota;p__Ascomycota;c__Sordariomycetes;o__Hypocreales;f__Cordycipitaceae;g__Simplicillium;s__Simplicillium lanosoniveum 1e-178 KT878334.1

OTU_781 k__Eukaryota;p__Unclassified;c__Unclassified;o__Unclassified;f__Unclassified;g__Unclassified;s__uncultured fungus 4e-83 KT244456.1

OTU_782 k__Eukaryota;p__Ascomycota;c__Saccharomycetes;o__Saccharomycetales;f__Phaffomycetaceae;g__Cyberlindnera;s__Cyberlindnera fabianii 0.0 KY657572.1

OTU_783 k__Eukaryota;p__Ascomycota;c__Dothideomycetes;o__Pleosporales;f__Leptosphaeriaceae;g__Leptosphaeria;s__Leptosphaeria sp. PHY-30 7e-165 JX401955.1

OTU_784 k__Eukaryota;p__Ascomycota;c__Dothideomycetes;o__Pleosporales;f__Massarinaceae;g__Stagonospora;s__Stagonospora foliicola 8e-109 KF251256.1

OTU_785 k__Eukaryota;p__Unclassified;c__Unclassified;o__Unclassified;f__Unclassified;g__Unclassified;s__uncultured fungus 8e-103 MF569941.1

OTU_786 k__Eukaryota;p__Basidiomycota;c__Exobasidiomycetes;o__Microstromatales;f__Unclassified;g__Sympodiomycopsis;s__Sympodiomycopsis sp. S6A 0.0 AM931015.1

OTU_787 k__Eukaryota;p__Unclassified;c__Unclassified;o__Unclassified;f__Unclassified;g__Unclassified;s__uncultured fungus 7e-165 MF569271.1

OTU_788 k__Eukaryota;p__Ascomycota;c__Dothideomycetes;o__Pleosporales;f__Unclassified;g__Unclassified;s__Pleosporales sp. CSV4 2e-165 KU571702.1

OTU_789 k__Eukaryota;p__Ascomycota;c__Dothideomycetes;o__Pleosporales;f__Phaeosphaeriaceae;g__Neosetophoma;s__Neosetophoma italica 3e-111 KP711356.1

OTU_79 k__Eukaryota;p__Unclassified;c__Unclassified;o__Unclassified;f__Unclassified;g__Unclassified;s__uncultured fungus 4e-77 KX194578.1

OTU_790 k__Eukaryota;p__Unclassified;c__Unclassified;o__Unclassified;f__Unclassified;g__Unclassified;s__uncultured fungus 2e-39 MF570568.1

OTU_791 k__Eukaryota;p__Chytridiomycota;c__Neocallimastigomycetes;o__Neocallimastigales;f__Unclassified;g__Unclassified;s__uncultured Neocallimastigales 0.0 KC431236.1

OTU_792 k__Eukaryota;p__Unclassified;c__Unclassified;o__Unclassified;f__Unclassified;g__Unclassified;s__uncultured fungus 2e-165 KF800187.1

OTU_793 k__Eukaryota;p__Unclassified;c__Unclassified;o__Unclassified;f__Unclassified;g__Unclassified;s__fungal sp. ARIZ AZ0955 9e-97 HM123626.1

OTU_794 k__Eukaryota;p__Unclassified;c__Unclassified;o__Unclassified;f__Unclassified;g__Unclassified;s__uncultured fungus 1e-153 MF569509.1

OTU_796 k__Eukaryota;p__Basidiomycota;c__Agaricomycetes;o__Agaricales;f__Tricholomataceae;g__Unclassified;s__uncultured Mycena 0.0 JF519610.1

OTU_797 k__Eukaryota;p__Ascomycota;c__Unclassified;o__Unclassified;f__Unclassified;g__Unclassified;s__Ascomycota sp. AR-2010 4e-148 HQ607855.1

OTU_798 k__Eukaryota;p__Unclassified;c__Unclassified;o__Unclassified;f__Unclassified;g__Unclassified;s__uncultured fungus 1e-169 KX193649.1

OTU_799 k__Eukaryota;p__Unclassified;c__Unclassified;o__Unclassified;f__Unclassified;g__Unclassified;s__uncultured fungus 5e-178 KX194917.1

OTU_8 k__Eukaryota;p__Ascomycota;c__Sordariomycetes;o__Hypocreales;f__Unclassified;g__Acremonium;s__Acremonium sclerotigenum 2e-177 KY929277.1

OTU_80 k__Eukaryota;p__Ascomycota;c__Dothideomycetes;o__Pleosporales;f__Didymosphaeriaceae;g__Paraconiothyrium;s__Paraconiothyrium brasiliense 1e-166 MG572394.1

OTU_800 k__Eukaryota;p__Basidiomycota;c__Agaricomycetes;o__Agaricales;f__Bolbitiaceae;g__Descomyces;s__Descomyces sp. JAC9526 0.0 KP191851.1

OTU_801 k__Eukaryota;p__Ascomycota;c__Sordariomycetes;o__Hypocreales;f__Unclassified;g__Acremonium;s__Acremonium sclerotigenum 1e-163 KY929277.1

OTU_802 k__Eukaryota;p__Ascomycota;c__Sordariomycetes;o__Hypocreales;f__Ophiocordycipitaceae;g__Ophiocordyceps;s__Ophiocordyceps sinensis 7e-168 MG457822.1

OTU_803 k__Eukaryota;p__Unclassified;c__Unclassified;o__Unclassified;f__Unclassified;g__Unclassified;s__uncultured fungus 6e-178 KP897575.1

OTU_804 k__Eukaryota;p__Ascomycota;c__Sordariomycetes;o__Xylariales;f__Hypoxylaceae;g__Hypoxylon;s__Hypoxylon sp. HJ 5e-169 EU744544.1

OTU_805 k__Eukaryota;p__Ascomycota;c__Eurotiomycetes;o__Eurotiales;f__Aspergillaceae;g__Penicillium;s__Penicillium citrinum 4e-157 MF135514.1

OTU_806 k__Eukaryota;p__Unclassified;c__Unclassified;o__Unclassified;f__Unclassified;g__Unclassified;s__uncultured fungus 2e-08 MF571135.1

OTU_807 k__Eukaryota;p__Ascomycota;c__Saccharomycetes;o__Saccharomycetales;f__Pichiaceae;g__Pichia;s__Pichia sp. TCJ3 3e-130 HM044858.1

OTU_808 k__Eukaryota;p__Unclassified;c__Unclassified;o__Unclassified;f__Unclassified;g__Unclassified;s__uncultured fungus 1e-46 FJ362303.1

OTU_809 k__Eukaryota;p__Basidiomycota;c__Tremellomycetes;o__Trichosporonales;f__Trichosporonaceae;g__Cutaneotrichosporon;s__Cutaneotrichosporon jirovecii 8e-174 KY441474.1

OTU_81 k__Eukaryota;p__Ascomycota;c__Dothideomycetes;o__Pleosporales;f__Unclassified;g__Unclassified;s__Pleosporales sp. 2e-168 KY379800.1

OTU_810 k__Eukaryota;p__Ascomycota;c__Sordariomycetes;o__Chaetosphaeriales;f__Chaetosphaeriaceae;g__Pseudolachnea;s__Pseudolachnea fraxini 4e-120 AB934069.1

OTU_811 k__Eukaryota;p__Unclassified;c__Unclassified;o__Unclassified;f__Unclassified;g__Unclassified;s__uncultured fungus 4e-166 KX192573.1

OTU_812 k__Eukaryota;p__Unclassified;c__Unclassified;o__Unclassified;f__Unclassified;g__Unclassified;s__uncultured fungus 2e-128 MF568863.1

OTU_813 k__Eukaryota;p__Ascomycota;c__Saccharomycetes;o__Saccharomycetales;f__Debaryomycetaceae;g__Scheffersomyces;s__Scheffersomyces amazonensis 5e-98 NR_138231.1

OTU_814 k__Eukaryota;p__Ascomycota;c__Saccharomycetes;o__Saccharomycetales;f__Saccharomycodaceae;g__Hanseniaspora;s__Hanseniaspora uvarum 3e-180 MG020690.1

OTU_815 k__Eukaryota;p__Ascomycota;c__Sordariomycetes;o__Hypocreales;f__Nectriaceae;g__Neonectria;s__Neonectria candida 5e-172 MG000969.1

OTU_816 k__Eukaryota;p__Basidiomycota;c__Tremellomycetes;o__Unclassified;f__Unclassified;g__Unclassified;s__uncultured Cryptococcus 3e-164 KY430495.1

OTU_817 k__Eukaryota;p__Ascomycota;c__Dothideomycetes;o__Pleosporales;f__Pleosporaceae;g__Comoclathris;s__Comoclathris sp. 9e-152 MG065811.1

OTU_818 k__Eukaryota;p__Unclassified;c__Unclassified;o__Unclassified;f__Unclassified;g__Unclassified;s__uncultured fungus 2e-11 MF570089.1

OTU_819 k__Eukaryota;p__Basidiomycota;c__Exobasidiomycetes;o__Ceraceosorales;f__Unclassified;g__Unclassified;s__Ceraceosorales sp. SA252w 4e-111 KM591597.1

OTU_82 k__Eukaryota;p__Ascomycota;c__Dothideomycetes;o__Unclassified;f__Unclassified;g__Unclassified;s__Dothideomycetes sp. genotype csw019 4e-160 KM519308.1

OTU_820 k__Eukaryota;p__Ascomycota;c__Dothideomycetes;o__Pleosporales;f__Leptosphaeriaceae;g__Leptosphaeria;s__Leptosphaeria biglobosa 9e-07 FO906079.1

OTU_821 k__Eukaryota;p__Ascomycota;c__Dothideomycetes;o__Pleosporales;f__Phaeosphaeriaceae;g__Chaetosphaeronema;s__Chaetosphaeronema sp. 2e-103 KX611055.1

OTU_822 k__Eukaryota;p__Basidiomycota;c__Tremellomycetes;o__Tremellales;f__Cryptococcaceae;g__Kwoniella;s__Kwoniella mangrovensis 2e-153 KY103935.1

OTU_823 k__Eukaryota;p__Ascomycota;c__Dothideomycetes;o__Pleosporales;f__Didymellaceae;g__Phoma;s__Phoma sp. YIMPH30013 6e-162 KP230814.1

OTU_824 k__Eukaryota;p__Unclassified;c__Unclassified;o__Unclassified;f__Unclassified;g__Unclassified;s__uncultured fungus 6e-11 MF976148.1

OTU_825 k__Eukaryota;p__Unclassified;c__Unclassified;o__Unclassified;f__Unclassified;g__Unclassified;s__uncultured fungus 4e-83 MF569470.1

OTU_826 k__Eukaryota;p__Ascomycota;c__Unclassified;o__Unclassified;f__Unclassified;g__Unclassified;s__uncultured Ascomycota 3e-145 HM162212.1

OTU_827 k__Eukaryota;p__Unclassified;c__Unclassified;o__Unclassified;f__Unclassified;g__Unclassified;s__uncultured fungus 0.0 KF617566.1

OTU_828 k__Eukaryota;p__Ascomycota;c__Unclassified;o__Unclassified;f__Unclassified;g__Dichotomophthora;s__Dichotomophthora sp. 9e-149 MF196163.1

OTU_829 k__Eukaryota;p__Unclassified;c__Unclassified;o__Unclassified;f__Unclassified;g__Unclassified;s__uncultured fungus 0.0 MF570305.1

OTU_83 k__Eukaryota;p__Basidiomycota;c__Agaricomycetes;o__Agaricales;f__Agaricaceae;g__Agaricus;s__Agaricus pseudopratensis 0.0 DQ182526.1

OTU_830 k__Eukaryota;p__Ascomycota;c__Saccharomycetes;o__Saccharomycetales;f__Saccharomycetaceae;g__Saccharomyces;s__Saccharomyces cerevisiae 0.0 KY596697.1

OTU_831 k__Eukaryota;p__Unclassified;c__Unclassified;o__Unclassified;f__Unclassified;g__Unclassified;s__uncultured fungus 1e-153 JQ312925.1

OTU_832 k__Eukaryota;p__Unclassified;c__Unclassified;o__Unclassified;f__Unclassified;g__Unclassified;s__uncultured fungus 2e-159 KU931415.1

OTU_833 k__Eukaryota;p__Unclassified;c__Unclassified;o__Unclassified;f__Unclassified;g__Unclassified;s__uncultured fungus 7e-165 MF570917.1

OTU_834 k__Eukaryota;p__Ascomycota;c__Dothideomycetes;o__Capnodiales;f__Teratosphaeriaceae;g__Catenulostroma;s__Catenulostroma protearum 2e-156 GU214629.1

OTU_835 k__Eukaryota;p__Unclassified;c__Unclassified;o__Unclassified;f__Unclassified;g__Unclassified;s__fungal sp. L10 3e-133 KF887091.1

OTU_836 k__Eukaryota;p__Unclassified;c__Unclassified;o__Unclassified;f__Unclassified;g__Unclassified;s__uncultured fungus 2e-47 FJ362303.1

OTU_837 k__Eukaryota;p__Unclassified;c__Unclassified;o__Unclassified;f__Unclassified;g__Unclassified;s__uncultured fungus 1e-172 KX664712.1

OTU_838 k__Eukaryota;p__Basidiomycota;c__Agaricomycetes;o__Agaricales;f__Cortinariaceae;g__Unclassified;s__uncultured Cortinarius 0.0 EF619680.1

OTU_839 k__Eukaryota;p__Ascomycota;c__Dothideomycetes;o__Pleosporales;f__Didymellaceae;g__Phoma;s__Phoma betae 5e-110 KC460811.1

OTU_84 k__Eukaryota;p__Ascomycota;c__Sordariomycetes;o__Hypocreales;f__Nectriaceae;g__Fusarium;s__Fusarium brachygibbosum 7e-171 MG250444.1

OTU_840 k__Eukaryota;p__Ascomycota;c__Unclassified;o__Unclassified;f__Unclassified;g__Unclassified;s__fungal endophyte MS5 IS33 2e-152 AF413044.1

OTU_841 k__Eukaryota;p__Unclassified;c__Unclassified;o__Unclassified;f__Unclassified;g__Unclassified;s__uncultured fungus 2e-162 MF570710.1

OTU_842 k__Eukaryota;p__Unclassified;c__Unclassified;o__Unclassified;f__Unclassified;g__Unclassified;s__uncultured fungus 2e-131 GQ851635.1

OTU_843 k__Eukaryota;p__Ascomycota;c__Dothideomycetes;o__Capnodiales;f__Mycosphaerellaceae;g__Pseudocercospora;s__Pseudocercospora nephrolepidicola 2e-155 KP050647.1

OTU_844 k__Eukaryota;p__Unclassified;c__Unclassified;o__Unclassified;f__Unclassified;g__Unclassified;s__uncultured fungus 3e-167 EU690464.1

OTU_845 k__Eukaryota;p__Ascomycota;c__Dothideomycetes;o__Pleosporales;f__Didymellaceae;g__Phoma;s__Phoma omnivirens 2e-165 KP686177.1

OTU_846 k__Bacteria;p__Proteobacteria;c__Gammaproteobacteria;o__Pseudomonadales;f__Pseudomonadaceae;g__Pseudomonas;s__Pseudomonas oryzae 7e-66 LT629751.1

OTU_847 k__Eukaryota;p__Basidiomycota;c__Agaricomycetes;o__Agaricales;f__Psathyrellaceae;g__Unclassified;s__uncultured Psathyrellaceae 5e-163 KX115711.1

OTU_848 k__Eukaryota;p__Ascomycota;c__Dothideomycetes;o__Pleosporales;f__Leptosphaeriaceae;g__Leptosphaeria;s__Leptosphaeria biglobosa 7e-57 FO906144.1

OTU_849 k__Eukaryota;p__Unclassified;c__Unclassified;o__Unclassified;f__Unclassified;g__Unclassified;s__uncultured fungus 2e-143 GU174335.1

OTU_85 k__Eukaryota;p__Ascomycota;c__Dothideomycetes;o__Pleosporales;f__Pleosporaceae;g__Curvularia;s__Curvularia sp. 3e-170 MG250434.1

OTU_850 k__Eukaryota;p__Ascomycota;c__Leotiomycetes;o__Helotiales;f__Unclassified;g__Unclassified;s__uncultured Helotiales 2e-109 KP963663.1

OTU_851 k__Eukaryota;p__Basidiomycota;c__Unclassified;o__Unclassified;f__Unclassified;g__Unclassified;s__uncultured Sporobolomyces 5e-144 KC922117.1

OTU_852 k__Eukaryota;p__Ascomycota;c__Saccharomycetes;o__Saccharomycetales;f__Saccharomycodaceae;g__Hanseniaspora;s__Hanseniaspora uvarum 1e-179 MG020690.1

OTU_853 k__Eukaryota;p__Ascomycota;c__Leotiomycetes;o__Unclassified;f__Pseudeurotiaceae;g__Geomyces;s__Geomyces sp. CZ-2011d 2e-137 JN104554.1

OTU_854 k__Eukaryota;p__Ascomycota;c__Eurotiomycetes;o__Eurotiales;f__Aspergillaceae;g__Penicillium;s__Penicillium brevicompactum 3e-167 DQ249211.1

OTU_855 k__Eukaryota;p__Unclassified;c__Unclassified;o__Unclassified;f__Unclassified;g__Unclassified;s__uncultured fungus 0.0 MF568812.1

OTU_856 k__Eukaryota;p__Ascomycota;c__Unclassified;o__Unclassified;f__Unclassified;g__Unclassified;s__uncultured Ascomycota 1e-157 DQ182422.1

OTU_857 k__Eukaryota;p__Ascomycota;c__Dothideomycetes;o__Pleosporales;f__Phaeosphaeriaceae;g__Unclassified;s__uncultured Ophiosphaerella 2e-159 HG936717.1

OTU_858 k__Eukaryota;p__Unclassified;c__Unclassified;o__Unclassified;f__Unclassified;g__Unclassified;s__uncultured fungus 5e-144 KX515738.1

OTU_859 k__Eukaryota;p__Ascomycota;c__Dothideomycetes;o__Pleosporales;f__Pleosporaceae;g__Alternaria;s__Alternaria sp. Cf1 2e-155 KF564051.1

OTU_86 k__Eukaryota;p__Ascomycota;c__Sordariomycetes;o__Hypocreales;f__Nectriaceae;g__Fusarium;s__Fusarium proliferatum 3e-173 MG562501.1

OTU_860 k__Eukaryota;p__Ascomycota;c__Sordariomycetes;o__Glomerellales;f__Glomerellaceae;g__Colletotrichum;s__Colletotrichum gloeosporioides 1e-89 KX906602.1

OTU_861 k__Eukaryota;p__Unclassified;c__Unclassified;o__Unclassified;f__Unclassified;g__Unclassified;s__uncultured fungus 2e-177 MF568841.1

OTU_862 k__Eukaryota;p__Ascomycota;c__Dothideomycetes;o__Pleosporales;f__Leptosphaeriaceae;g__Alternariaster;s__Alternariaster helianthi 6e-162 JX893018.1

OTU_863 k__Eukaryota;p__Ascomycota;c__Leotiomycetes;o__Helotiales;f__Sclerotiniaceae;g__Botrytis;s__Botrytis cinerea 2e-137 KY419551.1

OTU_864 k__Eukaryota;p__Ascomycota;c__Eurotiomycetes;o__Eurotiales;f__Unclassified;g__Unclassified;s__uncultured Eurotiales 1e-120 HQ389458.1

OTU_865 k__Eukaryota;p__Ascomycota;c__Dothideomycetes;o__Capnodiales;f__Mycosphaerellaceae;g__Mycosphaerella;s__Mycosphaerella polygoni-cuspidati 3e-142 LC146384.1

OTU_866 k__Eukaryota;p__Unclassified;c__Unclassified;o__Unclassified;f__Unclassified;g__Unclassified;s__uncultured fungus 8e-106 KC884338.1

OTU_867 k__Eukaryota;p__Unclassified;c__Unclassified;o__Unclassified;f__Unclassified;g__Unclassified;s__uncultured fungus 9e-174 AY704751.1

OTU_868 k__Eukaryota;p__Unclassified;c__Unclassified;o__Unclassified;f__Unclassified;g__Unclassified;s__uncultured fungus 7e-165 KX192960.1

OTU_869 k__Eukaryota;p__Unclassified;c__Unclassified;o__Unclassified;f__Unclassified;g__Unclassified;s__uncultured fungus 4e-71 JX319026.1

OTU_87 k__Eukaryota;p__Unclassified;c__Unclassified;o__Unclassified;f__Unclassified;g__Unclassified;s__uncultured fungus 7e-168 MF568856.1

OTU_870 k__Eukaryota;p__Ascomycota;c__Sordariomycetes;o__Microascales;f__Microascaceae;g__Petriella;s__Petriella sp. 1e-172 KU702714.1

OTU_871 k__Eukaryota;p__Ascomycota;c__Dothideomycetes;o__Pleosporales;f__Phaeosphaeriaceae;g__Parastagonospora;s__Parastagonospora avenae 1e-160 KY090646.1

OTU_872 k__Eukaryota;p__Ascomycota;c__Eurotiomycetes;o__Chaetothyriales;f__Herpotrichiellaceae;g__Exophiala;s__Exophiala sp. DTO 305-D1 7e-08 KX147577.1

OTU_873 k__Eukaryota;p__Basidiomycota;c__Tremellomycetes;o__Trichosporonales;f__Trichosporonaceae;g__Cryptococcus;s__Cryptococcus sp. CBS 8355 4e-157 AF444385.1

OTU_874 k__Eukaryota;p__Ascomycota;c__Dothideomycetes;o__Capnodiales;f__Cladosporiaceae;g__Cladosporium;s__Cladosporium tenuissimum 7e-125 MG572368.1

OTU_875 k__Eukaryota;p__Ascomycota;c__Dothideomycetes;o__Pleosporales;f__Leptosphaeriaceae;g__Leptosphaeria;s__Leptosphaeria biglobosa 9e-07 FO906089.1

OTU_876 k__Eukaryota;p__Ascomycota;c__Dothideomycetes;o__Pleosporales;f__Leptosphaeriaceae;g__Leptosphaeria;s__Leptosphaeria biglobosa 3e-25 FO906071.1

OTU_877 k__Eukaryota;p__Unclassified;c__Unclassified;o__Unclassified;f__Unclassified;g__Unclassified;s__uncultured fungus 3e-115 KX194904.1

OTU_878 k__Eukaryota;p__Ascomycota;c__Dothideomycetes;o__Dothideales;f__Saccotheciaceae;g__Aureobasidium;s__Aureobasidium pullulans 3e-142 KY294714.1

OTU_879 k__Eukaryota;p__Ascomycota;c__Sordariomycetes;o__Unclassified;f__Unclassified;g__Unclassified;s__uncultured Phaeoacremonium 5e-70 HQ022067.1

OTU_88 k__Eukaryota;p__Unclassified;c__Unclassified;o__Unclassified;f__Unclassified;g__Unclassified;s__uncultured fungus 6e-51 MF570389.1

OTU_880 k__Eukaryota;p__Ascomycota;c__Dothideomycetes;o__Pleosporales;f__Leptosphaeriaceae;g__Leptosphaeria;s__Leptosphaeria biglobosa 9e-07 FO906080.1

OTU_881 k__Eukaryota;p__Ascomycota;c__Saccharomycetes;o__Saccharomycetales;f__Pichiaceae;g__Pichia;s__Pichia manshurica 3e-139 KP250848.1

OTU_882 k__Eukaryota;p__Ascomycota;c__Unclassified;o__Unclassified;f__Unclassified;g__Unclassified;s__uncultured Pezizomycotina 3e-161 FJ554411.1

OTU_883 k__Eukaryota;p__Basidiomycota;c__Agaricomycetes;o__Agaricales;f__Amanitaceae;g__Amanita;s__Amanita umbrinella 1e-126 AY194981.1

OTU_884 k__Eukaryota;p__Unclassified;c__Unclassified;o__Unclassified;f__Unclassified;g__Unclassified;s__uncultured fungus 0.0 HE979326.1

OTU_885 k__Eukaryota;p__Basidiomycota;c__Agaricomycetes;o__Agaricales;f__Agaricaceae;g__Unclassified;s__uncultured Agaricaceae 0.0 KM247484.1

OTU_886 k__Eukaryota;p__Ascomycota;c__Dothideomycetes;o__Pleosporales;f__Lentitheciaceae;g__Keissleriella;s__Keissleriella cirsii 2e-125 KY497783.1

OTU_887 k__Eukaryota;p__Unclassified;c__Unclassified;o__Unclassified;f__Unclassified;g__Unclassified;s__uncultured fungus 0.0 KX195355.1

OTU_888 k__Eukaryota;p__Ascomycota;c__Sordariomycetes;o__Glomerellales;f__Glomerellaceae;g__Colletotrichum;s__Colletotrichum fructicola 3e-102 KJ131635.1

OTU_889 k__Eukaryota;p__Basidiomycota;c__Agaricomycetes;o__Agaricales;f__Bolbitiaceae;g__Descolea;s__Descolea alba 0.0 DQ328209.1

OTU_89 k__Eukaryota;p__Basidiomycota;c__Tremellomycetes;o__Unclassified;f__Unclassified;g__Cryptococcus;s__Cryptococcus sp. 0.0 KY887683.1

OTU_890 k__Eukaryota;p__Unclassified;c__Unclassified;o__Unclassified;f__Unclassified;g__Unclassified;s__uncultured fungus 4e-117 EU144632.1

OTU_891 k__Eukaryota;p__Ascomycota;c__Saccharomycetes;o__Saccharomycetales;f__Phaffomycetaceae;g__Wickerhamomyces;s__Wickerhamomyces anomalus 8e-174 MG519713.1

OTU_892 k__Eukaryota;p__Ascomycota;c__Eurotiomycetes;o__Eurotiales;f__Unclassified;g__Unclassified;s__Eurotiales sp. KO-groupB 2014 1e-163 AB986438.1

OTU_893 k__Eukaryota;p__Ascomycota;c__Dothideomycetes;o__Pleosporales;f__Didymosphaeriaceae;g__Unclassified;s__Montagnulaceae sp. A1S3-D34 1e-147 KJ780753.1

OTU_894 k__Eukaryota;p__Ascomycota;c__Eurotiomycetes;o__Eurotiales;f__Aspergillaceae;g__Aspergillus;s__Aspergillus versicolor 2e-174 MG551559.1

OTU_895 k__Eukaryota;p__Ascomycota;c__Dothideomycetes;o__Capnodiales;f__Mycosphaerellaceae;g__Lecanosticta;s__Lecanosticta gloeospora 4e-12 KU948431.1

OTU_896 k__Eukaryota;p__Unclassified;c__Unclassified;o__Unclassified;f__Unclassified;g__Unclassified;s__uncultured fungus 0.0 MF569437.1

OTU_897 k__Eukaryota;p__Ascomycota;c__Sordariomycetes;o__Hypocreales;f__Clavicipitaceae;g__Metarhizium;s__Metarhizium anisopliae 2e-180 MF678583.1

OTU_898 k__Eukaryota;p__Unclassified;c__Unclassified;o__Unclassified;f__Unclassified;g__Unclassified;s__uncultured fungus 1e-70 MF569797.1

OTU_9 k__Eukaryota;p__Ascomycota;c__Saccharomycetes;o__Saccharomycetales;f__Saccharomycetaceae;g__Saccharomyces;s__Saccharomyces paradoxus 0.0 KY495744.1

OTU_90 k__Eukaryota;p__Ascomycota;c__Sordariomycetes;o__Trichosphaeriales;f__Trichosphaeriaceae;g__Nigrospora;s__Nigrospora oryzae 3e-167 MF580967.1

OTU_900 k__Eukaryota;p__Unclassified;c__Unclassified;o__Unclassified;f__Unclassified;g__Unclassified;s__uncultured fungus 6e-156 HG328049.1

OTU_901 k__Eukaryota;p__Unclassified;c__Unclassified;o__Unclassified;f__Unclassified;g__Unclassified;s__uncultured fungus 5e-61 FJ237229.1

OTU_902 k__Eukaryota;p__Ascomycota;c__Sordariomycetes;o__Togniniales;f__Togniniaceae;g__Phaeoacremonium;s__Phaeoacremonium sp. CBS 122685 2e-08 EU552161.1

OTU_903 k__Eukaryota;p__Unclassified;c__Unclassified;o__Unclassified;f__Unclassified;g__Unclassified;s__uncultured fungus 7e-165 KF800124.1

OTU_904 k__Eukaryota;p__Ascomycota;c__Sordariomycetes;o__Hypocreales;f__Unclassified;g__Acremonium;s__Acremonium sclerotigenum 3e-158 KY929277.1

OTU_905 k__Eukaryota;p__Ascomycota;c__Sordariomycetes;o__Sordariales;f__Lasiosphaeriaceae;g__Podospora;s__Podospora bicolor 2e-168 KP117268.1

OTU_906 k__Eukaryota;p__Basidiomycota;c__Agaricomycetes;o__Agaricales;f__Bolbitiaceae;g__Agrocybe;s__Agrocybe subpediades 0.0 KP826790.1

OTU_907 k__Eukaryota;p__Ascomycota;c__Eurotiomycetes;o__Eurotiales;f__Aspergillaceae;g__Aspergillus;s__Aspergillus inflatus 1e-163 AJ608959.1

OTU_908 k__Eukaryota;p__Unclassified;c__Unclassified;o__Unclassified;f__Unclassified;g__Unclassified;s__fungal sp. TZ-2015b 3e-139 KR698833.1

OTU_909 k__Eukaryota;p__Unclassified;c__Unclassified;o__Unclassified;f__Unclassified;g__Unclassified;s__uncultured fungus 2e-168 FN397299.1

OTU_91 k__Eukaryota;p__Ascomycota;c__Dothideomycetes;o__Dothideales;f__Dothideaceae;g__Dothidea;s__Dothidea sp. 2e-171 KX774104.1

OTU_910 k__Eukaryota;p__Ascomycota;c__Dothideomycetes;o__Pleosporales;f__Sporormiaceae;g__Preussia;s__Preussia sp. 1 JAS-2013 2e-159 KC427071.1

OTU_911 k__Eukaryota;p__Ascomycota;c__Sordariomycetes;o__Togniniales;f__Togniniaceae;g__Phaeoacremonium;s__Phaeoacremonium sp. 2e-180 MF136545.1

OTU_912 k__Eukaryota;p__Unclassified;c__Unclassified;o__Unclassified;f__Unclassified;g__Unclassified;s__uncultured fungus 2e-165 HG764521.1

OTU_913 k__Eukaryota;p__Unclassified;c__Unclassified;o__Unclassified;f__Unclassified;g__Unclassified;s__uncultured fungus 9e-118 KF800339.1

OTU_914 k__Eukaryota;p__Unclassified;c__Unclassified;o__Unclassified;f__Unclassified;g__Unclassified;s__uncultured fungus 9e-155 MF569682.1

OTU_915 k__Eukaryota;p__Basidiomycota;c__Microbotryomycetes;o__Sporidiobolales;f__Sporidiobolaceae;g__Rhodosporidiobolus;s__Rhodosporidiobolus odoratus 0.0 KY495748.1

OTU_916 k__Eukaryota;p__Ascomycota;c__Leotiomycetes;o__Helotiales;f__Unclassified;g__Cadophora;s__Cadophora sp. 34f 1e-120 JX243972.1

OTU_917 k__Eukaryota;p__Ascomycota;c__Sordariomycetes;o__Glomerellales;f__Glomerellaceae;g__Colletotrichum;s__Colletotrichum gloeosporioides 2e-84 KX906602.1

OTU_918 k__Eukaryota;p__Ascomycota;c__Dothideomycetes;o__Pleosporales;f__Leptosphaeriaceae;g__Ampelomyces;s__Ampelomyces quisqualis 2e-165 JX681067.1

OTU_919 k__Eukaryota;p__Unclassified;c__Unclassified;o__Unclassified;f__Unclassified;g__Unclassified;s__uncultured fungus 7e-165 MF568728.1

OTU_92 k__Eukaryota;p__Ascomycota;c__Dothideomycetes;o__Pleosporales;f__Lentitheciaceae;g__Keissleriella;s__Keissleriella cirsii 1e-132 KY497783.1

OTU_920 k__Eukaryota;p__Ascomycota;c__Sordariomycetes;o__Xylariales;f__Xylariaceae;g__Unclassified;s__uncultured cf. Daldinia 4e-151 HM998754.1

OTU_921 k__Eukaryota;p__Basidiomycota;c__Agaricomycetes;o__Corticiales;f__Vuilleminiaceae;g__Vuilleminia;s__Vuilleminia erastii 1e-163 NR_119979.1

OTU_922 k__Eukaryota;p__Ascomycota;c__Sordariomycetes;o__Hypocreales;f__Nectriaceae;g__Fusarium;s__Fusarium lateritium 7e-171 MF521451.1

OTU_923 k__Eukaryota;p__Unclassified;c__Unclassified;o__Unclassified;f__Unclassified;g__Unclassified;s__uncultured fungus 2e-137 JX136499.1

OTU_924 k__Eukaryota;p__Unclassified;c__Unclassified;o__Unclassified;f__Unclassified;g__Unclassified;s__fungal endophyte 1e-141 KT203190.1

OTU_925 k__Eukaryota;p__Ascomycota;c__Leotiomycetes;o__Helotiales;f__Unclassified;g__Hyalodendriella;s__Hyalodendriella betulae 7e-162 FJ755263.1

OTU_926 k__Eukaryota;p__Basidiomycota;c__Tremellomycetes;o__Tremellales;f__Cryptococcaceae;g__Kwoniella;s__Kwoniella heveanensis 9e-180 KX385850.1

OTU_927 k__Eukaryota;p__Unclassified;c__Unclassified;o__Unclassified;f__Unclassified;g__Unclassified;s__uncultured fungus 2e-159 FJ820763.1

OTU_928 k__Eukaryota;p__Unclassified;c__Unclassified;o__Unclassified;f__Unclassified;g__Unclassified;s__uncultured fungus 2e-168 KX192566.1

OTU_929 k__Eukaryota;p__Unclassified;c__Unclassified;o__Unclassified;f__Unclassified;g__Unclassified;s__uncultured fungus 1e-160 KX196111.1

OTU_93 k__Eukaryota;p__Ascomycota;c__Eurotiomycetes;o__Eurotiales;f__Aspergillaceae;g__Aspergillus;s__Aspergillus aculeatinus 3e-161 MG543743.1

OTU_930 k__Eukaryota;p__Ascomycota;c__Sordariomycetes;o__Coronophorales;f__Scortechiniaceae;g__Pseudocatenomycopsis;s__Pseudocatenomycopsis rothmanniae 2e-66 KF777185.1

OTU_931 k__Eukaryota;p__Unclassified;c__Unclassified;o__Unclassified;f__Unclassified;g__Unclassified;s__uncultured fungus 4e-157 MF571051.1

OTU_932 k__Eukaryota;p__Unclassified;c__Unclassified;o__Unclassified;f__Unclassified;g__Unclassified;s__fungal sp. 5e-172 MF347918.1

OTU_933 k__Eukaryota;p__Unclassified;c__Unclassified;o__Unclassified;f__Unclassified;g__Unclassified;s__fungal sp. 6e-159 KY776388.1

OTU_934 k__Eukaryota;p__Basidiomycota;c__Unclassified;o__Unclassified;f__Unclassified;g__Unclassified;s__uncultured Basidiomycota 6e-150 AM901961.1

OTU_935 k__Eukaryota;p__Ascomycota;c__Dothideomycetes;o__Pleosporales;f__Leptosphaeriaceae;g__Leptosphaeria;s__Leptosphaeria biglobosa 5e-33 FO906089.1

OTU_936 k__Eukaryota;p__Unclassified;c__Unclassified;o__Unclassified;f__Unclassified;g__Unclassified;s__fungal sp. 2e-155 KY379666.1

OTU_937 k__Eukaryota;p__Ascomycota;c__Dothideomycetes;o__Capnodiales;f__Mycosphaerellaceae;g__Septoria;s__cf. Septoria sp. CPC 19311 1e-52 KF251241.1

OTU_938 k__Eukaryota;p__Unclassified;c__Unclassified;o__Unclassified;f__Unclassified;g__Unclassified;s__uncultured fungus 0.0 KF800506.1

OTU_939 k__Eukaryota;p__Unclassified;c__Unclassified;o__Unclassified;f__Unclassified;g__Unclassified;s__uncultured fungus 6e-162 MF976185.1

OTU_94 k__Eukaryota;p__Ascomycota;c__Sordariomycetes;o__Hypocreales;f__Stachybotryaceae;g__Myrothecium;s__Myrothecium sp. 8e-177 MG562611.1

OTU_940 k__Eukaryota;p__Unclassified;c__Unclassified;o__Unclassified;f__Unclassified;g__Unclassified;s__uncultured fungus 3e-69 KX195664.1

OTU_941 k__Eukaryota;p__Ascomycota;c__Dothideomycetes;o__Capnodiales;f__Cladosporiaceae;g__Cladosporium;s__Cladosporium sp. BF72 8e-140 AM901688.1

OTU_942 k__Eukaryota;p__Ascomycota;c__Sordariomycetes;o__Glomerellales;f__Glomerellaceae;g__Colletotrichum;s__Colletotrichum gloeosporioides 2e-155 KX906602.1

OTU_943 k__Eukaryota;p__Ascomycota;c__Dothideomycetes;o__Pleosporales;f__Lentitheciaceae;g__Keissleriella;s__Keissleriella cirsii 3e-96 KY497783.1

OTU_944 k__Eukaryota;p__Ascomycota;c__Dothideomycetes;o__Dothideales;f__Saccotheciaceae;g__Aureobasidium;s__Aureobasidium sp. KUC1427 4e-151 KY294712.1

OTU_945 k__Eukaryota;p__Ascomycota;c__Dothideomycetes;o__Pleosporales;f__Pleosporaceae;g__Bipolaris;s__Bipolaris variabilis 3e-161 NR_151855.1

OTU_946 k__Eukaryota;p__Basidiomycota;c__Agaricomycetes;o__Atheliales;f__Atheliaceae;g__Athelia;s__Athelia bombacina 0.0 DQ449026.1

OTU_947 k__Eukaryota;p__Basidiomycota;c__Agaricomycetes;o__Agaricales;f__Psathyrellaceae;g__Coprinellus;s__Coprinellus sp. 0.0 MF972506.1

OTU_948 k__Eukaryota;p__Unclassified;c__Unclassified;o__Unclassified;f__Unclassified;g__Unclassified;s__uncultured fungus 1e-08 FJ362304.1

OTU_949 k__Eukaryota;p__Ascomycota;c__Dothideomycetes;o__Dothideales;f__Saccotheciaceae;g__Aureobasidium;s__Aureobasidium pullulans 1e-113 KY294714.1

OTU_95 k__Eukaryota;p__Ascomycota;c__Sordariomycetes;o__Xylariales;f__Unclassified;g__Zygosporium;s__Zygosporium gibbum 1e-154 KY853482.1

OTU_950 k__Eukaryota;p__Ascomycota;c__Saccharomycetes;o__Saccharomycetales;f__Unclassified;g__Candida;s__Candida sp. NY7122 7e-162 AB663086.1

OTU_951 k__Eukaryota;p__Ascomycota;c__Sordariomycetes;o__Microascales;f__Ceratocystidaceae;g__Ceratocystis;s__Ceratocystis fimbriata 2e-168 KY580898.1

OTU_952 k__Eukaryota;p__Unclassified;c__Unclassified;o__Unclassified;f__Unclassified;g__Unclassified;s__uncultured fungus 2e-149 MF569396.1

OTU_953 k__Eukaryota;p__Ascomycota;c__Leotiomycetes;o__Erysiphales;f__Erysiphaceae;g__Golovinomyces;s__Golovinomyces artemisiae 1e-166 AB769430.1

OTU_954 k__Eukaryota;p__Unclassified;c__Unclassified;o__Unclassified;f__Unclassified;g__Unclassified;s__uncultured fungus 3e-176 MF568823.1

OTU_955 k__Eukaryota;p__Ascomycota;c__Leotiomycetes;o__Helotiales;f__Unclassified;g__Unclassified;s__Helotiales sp. 6-NN-2017 2e-162 LC218320.1

OTU_956 k__Eukaryota;p__Unclassified;c__Unclassified;o__Unclassified;f__Unclassified;g__Unclassified;s__uncultured fungus 2e-23 MF570140.1

OTU_957 k__Eukaryota;p__Ascomycota;c__Sordariomycetes;o__Sordariales;f__Cephalothecaceae;g__Phialemonium;s__Phialemonium dimorphosporum 5e-169 KU751865.1

OTU_958 k__Eukaryota;p__Unclassified;c__Unclassified;o__Unclassified;f__Unclassified;g__Unclassified;s__uncultured fungus 4e-160 KF617727.1

OTU_959 k__Eukaryota;p__Ascomycota;c__Leotiomycetes;o__Helotiales;f__Hyaloscyphaceae;g__Cistella;s__Cistella sp. 8e-149 KT269719.1

OTU_96 k__Eukaryota;p__Basidiomycota;c__Tremellomycetes;o__Tremellales;f__Bulleribasidiaceae;g__Vishniacozyma;s__Vishniacozyma dimennae 9e-155 NR_144808.1

OTU_960 k__Eukaryota;p__Ascomycota;c__Dothideomycetes;o__Pleosporales;f__Pleosporaceae;g__Curvularia;s__Curvularia lunata 2e-174 KY100122.1

OTU_961 k__Eukaryota;p__Ascomycota;c__Sordariomycetes;o__Glomerellales;f__Glomerellaceae;g__Colletotrichum;s__Colletotrichum gloeosporioides 8e-140 KX906602.1

OTU_962 k__Eukaryota;p__Ascomycota;c__Dothideomycetes;o__Pleosporales;f__Pleosporaceae;g__Curvularia;s__Curvularia heteropogonis 1e-154 NR_137071.1

OTU_963 k__Eukaryota;p__Ascomycota;c__Leotiomycetes;o__Helotiales;f__Sclerotiniaceae;g__Botrytis;s__Botrytis cinerea 7e-137 KX463512.1

OTU_964 k__Eukaryota;p__Unclassified;c__Unclassified;o__Unclassified;f__Unclassified;g__Unclassified;s__uncultured fungus 2e-140 EF504322.1

OTU_965 k__Eukaryota;p__Ascomycota;c__Dothideomycetes;o__Pleosporales;f__Unclassified;g__Unclassified;s__Pleosporales sp. 3e-102 KY228578.1

OTU_966 k__Eukaryota;p__Ascomycota;c__Leotiomycetes;o__Erysiphales;f__Erysiphaceae;g__Podosphaera;s__Podosphaera xanthii 2e-159 KY388505.1

OTU_967 k__Eukaryota;p__Basidiomycota;c__Agaricomycetes;o__Unclassified;f__Unclassified;g__Unclassified;s__uncultured Agaricomycetes 0.0 FJ554417.1

OTU_968 k__Eukaryota;p__Unclassified;c__Unclassified;o__Unclassified;f__Unclassified;g__Unclassified;s__uncultured fungus 6e-153 KC222698.1

OTU_969 k__Eukaryota;p__Unclassified;c__Unclassified;o__Unclassified;f__Unclassified;g__Unclassified;s__uncultured fungus 0.0 EU218878.1

OTU_97 k__Eukaryota;p__Ascomycota;c__Unclassified;o__Unclassified;f__Unclassified;g__Unclassified;s__Ascomycota sp. UNEX FECRGA 2012E207 5e-169 KP899393.1

OTU_970 k__Eukaryota;p__Ascomycota;c__Sordariomycetes;o__Hypocreales;f__Stachybotryaceae;g__Paramyrothecium;s__Paramyrothecium sp. 0.0 KY126419.1

OTU_971 k__Eukaryota;p__Unclassified;c__Unclassified;o__Unclassified;f__Unclassified;g__Unclassified;s__uncultured fungus 8e-11 MF568708.1

OTU_972 k__Eukaryota;p__Ascomycota;c__Saccharomycetes;o__Saccharomycetales;f__Saccharomycopsidaceae;g__Unclassified;s__uncultured Saccharomycopsis 1e-175 JX159585.1

OTU_973 k__Eukaryota;p__Unclassified;c__Unclassified;o__Unclassified;f__Unclassified;g__Unclassified;s__uncultured fungus 1e-132 MG206934.1

OTU_974 k__Eukaryota;p__Ascomycota;c__Dothideomycetes;o__Dothideales;f__Saccotheciaceae;g__Aureobasidium;s__Aureobasidium pullulans 5e-144 KY294714.1

OTU_975 k__Eukaryota;p__Basidiomycota;c__Exobasidiomycetes;o__Tilletiales;f__Tilletiaceae;g__Tilletia;s__Tilletia horrida 0.0 DQ827699.1

OTU_976 k__Eukaryota;p__Unclassified;c__Unclassified;o__Unclassified;f__Unclassified;g__Unclassified;s__uncultured fungus 1e-166 KF800384.1

OTU_977 k__Eukaryota;p__Unclassified;c__Unclassified;o__Unclassified;f__Unclassified;g__Unclassified;s__uncultured fungus 6e-45 FJ362304.1

OTU_978 k__Eukaryota;p__Unclassified;c__Unclassified;o__Unclassified;f__Unclassified;g__Unclassified;s__fungal endophyte 2e-158 KF673747.1

OTU_979 k__Eukaryota;p__Ascomycota;c__Saccharomycetes;o__Saccharomycetales;f__Saccharomycodaceae;g__Hanseniaspora;s__Hanseniaspora uvarum 4e-170 MG020690.1

OTU_98 k__Eukaryota;p__Basidiomycota;c__Cystobasidiomycetes;o__Unclassified;f__Unclassified;g__Symmetrospora;s__Symmetrospora symmetrica 0.0 KJ701207.1

OTU_980 k__Bacteria;p__Proteobacteria;c__Alphaproteobacteria;o__Rhodospirillales;f__Acetobacteraceae;g__Acetobacter;s__Acetobacter persici 3e-161 CP014687.1

OTU_981 k__Eukaryota;p__Ascomycota;c__Dothideomycetes;o__Dothideales;f__Saccotheciaceae;g__Aureobasidium;s__Aureobasidium sp. JSKim-2015 5e-147 LC018758.1

OTU_982 k__Eukaryota;p__Unclassified;c__Unclassified;o__Unclassified;f__Unclassified;g__Unclassified;s__uncultured fungus 5e-15 KP889943.1

OTU_983 k__Eukaryota;p__Ascomycota;c__Sordariomycetes;o__Diaporthales;f__Diaporthaceae;g__Diaporthe;s__Diaporthe oncostoma 3e-164 KT459422.1

OTU_984 k__Eukaryota;p__Ascomycota;c__Dothideomycetes;o__Dothideales;f__Saccotheciaceae;g__Aureobasidium;s__Aureobasidium pullulans 1e-141 KY294714.1

OTU_985 k__Eukaryota;p__Ascomycota;c__Leotiomycetes;o__Helotiales;f__Helotiaceae;g__Unclassified;s__uncultured Articulospora 1e-156 KU176320.1

OTU_986 k__Eukaryota;p__Ascomycota;c__Eurotiomycetes;o__Chaetothyriales;f__Unclassified;g__Unclassified;s__uncultured Sarcinomyces 2e-171 KP843480.1

OTU_987 k__Eukaryota;p__Unclassified;c__Unclassified;o__Unclassified;f__Unclassified;g__Unclassified;s__fungal endophyte 3e-139 KT203156.1

OTU_988 k__Eukaryota;p__Unclassified;c__Unclassified;o__Unclassified;f__Unclassified;g__Unclassified;s__fungal sp. R3 1e-135 AY699666.1

OTU_989 k__Eukaryota;p__Ascomycota;c__Saccharomycetes;o__Saccharomycetales;f__Debaryomycetaceae;g__Kurtzmaniella;s__[Candida] oleophila 0.0 KY076621.1

OTU_99 k__Eukaryota;p__Ascomycota;c__Dothideomycetes;o__Pleosporales;f__Leptosphaeriaceae;g__Leptosphaeria;s__Leptosphaeria sp. MPSC 36 1e-153 AF439468.1

OTU_990 k__Eukaryota;p__Ascomycota;c__Dothideomycetes;o__Dothideales;f__Saccotheciaceae;g__Aureobasidium;s__aff. Aureobasidium sp. 9e-146 KT150707.1

OTU_991 k__Eukaryota;p__Ascomycota;c__Unclassified;o__Unclassified;f__Unclassified;g__Acremonium;s__Acremonium citrinum 3e-164 HF680236.1

OTU_992 k__Eukaryota;p__Unclassified;c__Unclassified;o__Unclassified;f__Unclassified;g__Unclassified;s__uncultured fungus 3e-148 KX194484.1

OTU_993 k__Eukaryota;p__Unclassified;c__Unclassified;o__Unclassified;f__Unclassified;g__Unclassified;s__uncultured fungus 1e-160 MF569463.1

OTU_994 k__Eukaryota;p__Ascomycota;c__Saccharomycetes;o__Saccharomycetales;f__Saccharomycodaceae;g__Hanseniaspora;s__Hanseniaspora sp. 5e-163 KY977693.1

OTU_995 k__Eukaryota;p__Ascomycota;c__Leotiomycetes;o__Helotiales;f__Unclassified;g__Cadophora;s__Cadophora orchidicola 3e-161 KY271872.1

OTU_996 k__Eukaryota;p__Unclassified;c__Unclassified;o__Unclassified;f__Unclassified;g__Unclassified;s__fungal sp. SL-57 7e-168 KC460851.1

OTU_997 k__Bacteria;p__Firmicutes;c__Clostridia;o__Clostridiales;f__Lachnospiraceae;g__Roseburia;s__Roseburia hominis 3e-127 CP003040.1

OTU_998 k__Eukaryota;p__Basidiomycota;c__Tremellomycetes;o__Tremellales;f__Bulleraceae;g__Fonsecazyma;s__Fonsecazyma mujuensis 5e-95 NR_137814.1

OTU_999 k__Eukaryota;p__Unclassified;c__Unclassified;o__Unclassified;f__Unclassified;g__Unclassified;s__fungal sp. SNB-LD7.2 1e-144 KF164418.1
