# Supplementary material for: The Biogeography of Fungal Communities Across Different Chinese Wine-Producing Regions Associated With Environmental Factors and Spontaneous Fermentation Performance
Source: Front Microbiol. 2022 Feb 25;12:636639. doi: 10.3389/fmicb.2021.636639 (PMC8914289; doi:10.3389/fmicb.2021.636639)
Supplement: Supplementary file 3 [file Presentation_1.pdf]

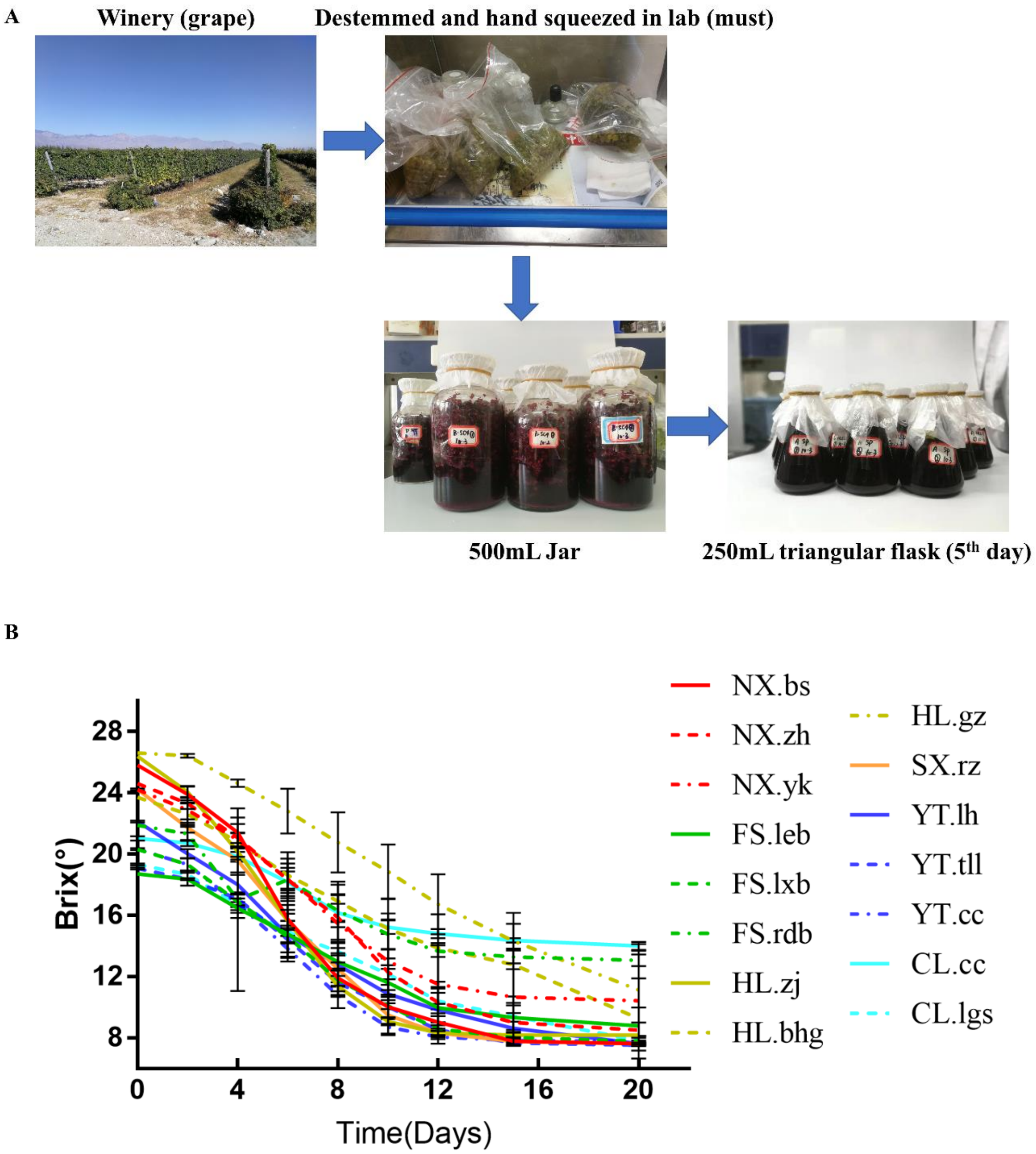

Figure S1 Spontaneous fermentation process of Marselan grape. (**A**) Experiment process; (**B**) The variation of °Brix.

A

B

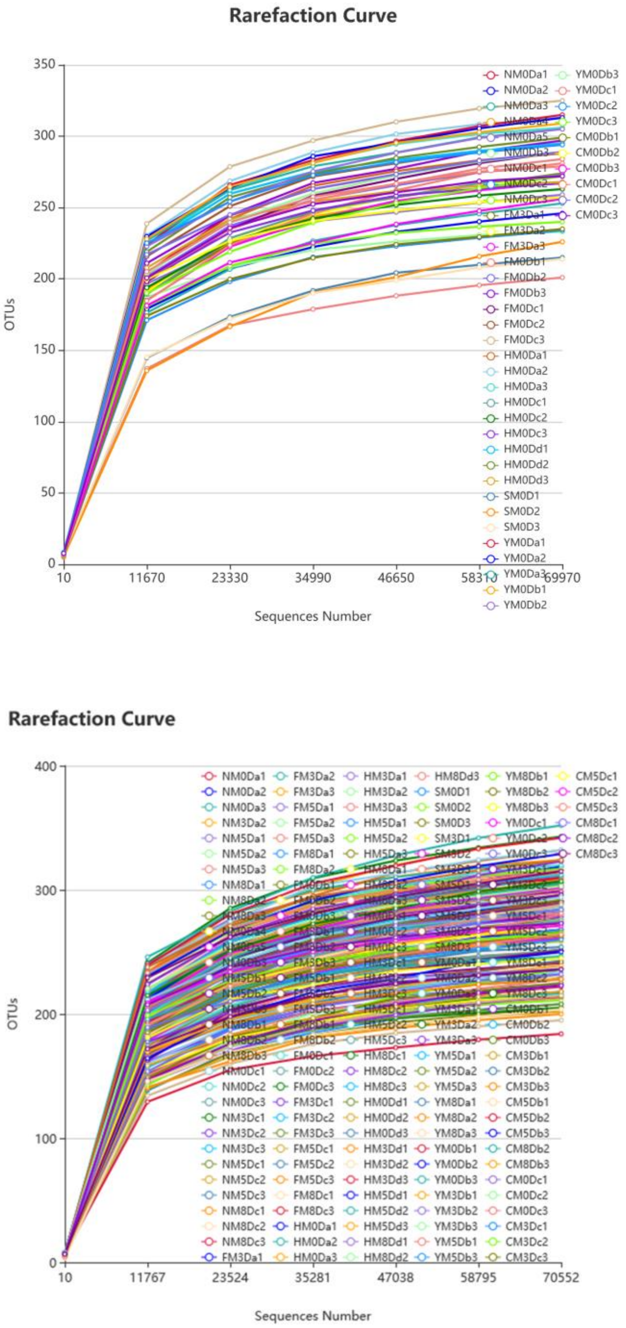

Figure S2 Rarefaction curves of Marselan must **(A)** and fermented **(B)** samples.

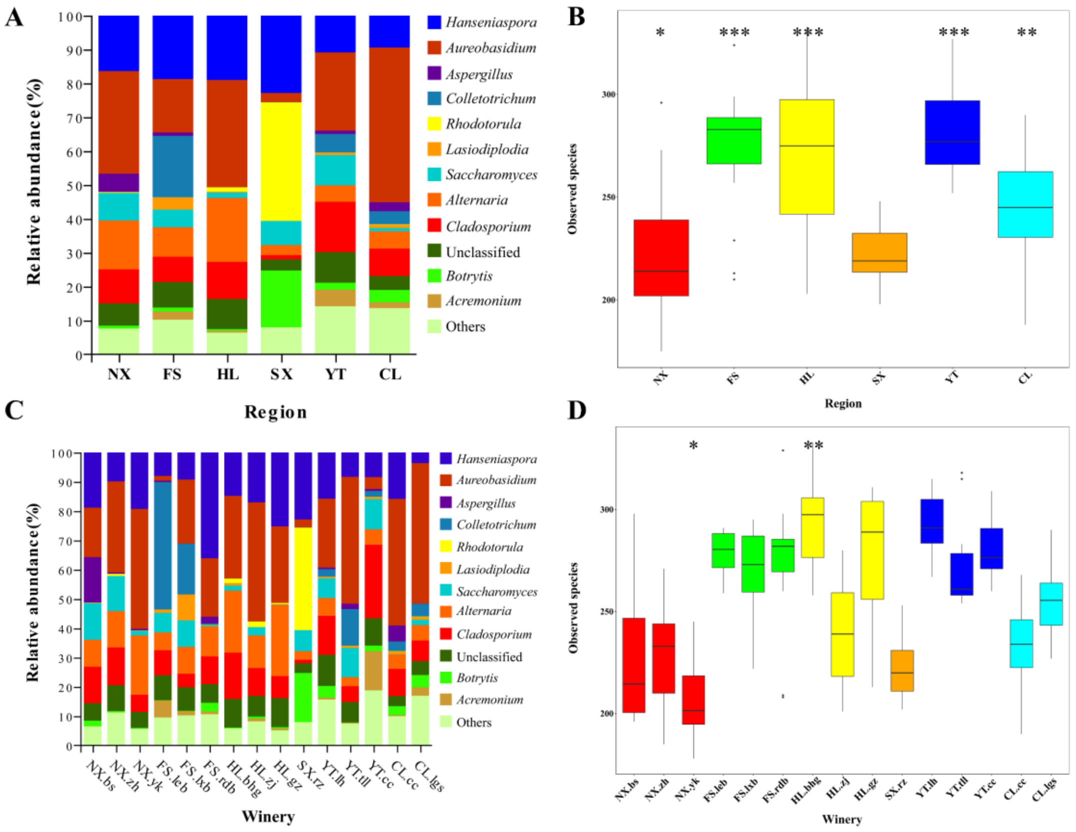

Figure S3 Marselan fermented sample fungal communities. (**A**) Relative abundance percentages (%) of the Marselan fermented sample fungal genera from different regions; (**B**) Wilcox test involving the Marselan fermented sample fungal species numbers of different regions; (**C**) Relative abundance percentages (%) of the Marselan fermented sample fungal genera of different wineries; (**D**) Wilcox test involving the Marselan fermented sample fungal species numbers of different wineries.

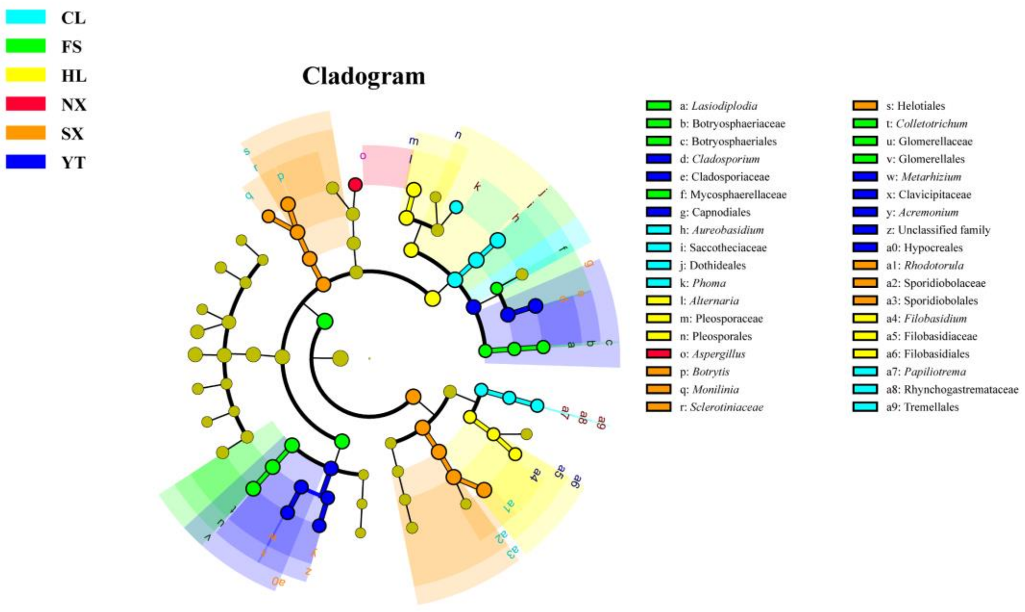

Figure S4 LEfSe results reporting the biomarkers of different regions in the Marselan fermented sample fungal communities.
