# Supplementary material for: The Biogeography of Fungal Communities Across Different Chinese Wine-Producing Regions Associated With Environmental Factors and Spontaneous Fermentation Performance
Source: Front Microbiol. 2022 Feb 25;12:636639. doi: 10.3389/fmicb.2021.636639 (PMC8914289; doi:10.3389/fmicb.2021.636639)
Supplement: Supplementary file 4 [file Table_1.docx]

**Table S1** Sampling regions and wineries of Marselan grape.

| **Region name (abbreviation)** | **Winery** | **Winery name abbreviation (number)** | **Coordinate position** | **Climatic characteristics** |
| --- | --- | --- | --- | --- |
| Ningxia's Helan  Mountain's  Eastern Region  (**NX**) | Baoshi | NX.bs (A) | N38°43′42.06″,  E106°05′41.38″ | temperate climate,  arid zone |
|  | Zhihuiyuanshi | NX.zh (B) | N38°34′55.90″,  E106°02′22.17″ |  |
|  | Yunkou | NX.yk (C) | N38°37′39.54″,  E106°01′18.92″ |  |
| Fangshan District, Beijing  (**FS**) | Chateau Lion | FS.leb (A) | N39°47′17.40″,  E116°12′37.71″ | warm climate,  semi-humid zones |
|  | Chateau Longxi | FS.lxb (B) | N39°35′25.35″,  E115°44′15.75″ |  |
|  | Chateau Ryden | FS.rdb (C) | N39°49′58.86″,  E116°03′47.06″ |  |
| Huailai County, Hebei Province (**HL**) | Amethyard | HL.zj (A) | N40°18′50.07″,  E115°49′21.28″ |  |
|  | Baihuagu | HL.bhg (B) | N40°19′58.68″,  E115°49′34.93″ |  |
|  | Guizu | HL.gz (C) | N40°22′23.61″,  E115°35′37.42″ |  |
| Changli County, Hebei Province  (**CL**) | Great Wall (CL) | CL.cc (A) | N39°44′19.19″,  E119°11′45.86″ |  |
|  | Langgesi | CL.lgs (B) | N39°46′6.63″,  E119°15′10.76″ |  |
| Xiangning County, Shanxi Province  (**SX**) | Chateau Rongzi | SX.rz (A) | N35°59′50.56″,  E110°45′57.54″. |  |
| Penglai City, Shandong Province  (**YT**) | Longhu | YT.lh (A) | N37°45′12.83″,  E120°44′32.71″ | hot climate,  semi-humid zone |
|  | Tonglili | YT.tll (B) | N37°46′47.58″,  E120°55′2.29″ |  |
|  | Great Wall (Penglai) | YT.cc (C) | N37°46′57.52″,  E120°53′57.87″ |  |

**Table S2** Physical and chemical indexes of Marselan must in different wineries.

| **Winery** | **Glucose (g/L)** | **Fructose (g/L)** | **pH** | **°Brix** | **Total acid (g/L)** | **Polyphenol (mg/L)** | **Anthocyanin**  **(mg/L)** |
| --- | --- | --- | --- | --- | --- | --- | --- |
| NX.bs | 148.53±0.16 | 129.53±0.43 | 3.35±0.01 | 25.8 | 5.16±0.05 | 174.00±2.64 | 3.66±0.57 |
| NX.zh | 152.37±0.35 | 120.51±0.22 | 3.27±0.01 | 24.6 | 5.9±0.2 | 156.33±1.15 | 4.66±0.57 |
| NX.yk | 147.55±0.38 | 131.37±0.38 | 3.78±0.01 | 24.2 | 4.8±0.17 | 265.33±1.15 | 7.66±0.57 |
| FS.leb | 99.64±0.24 | 99.98±0.37 | 3.49 | 18.7 | 8.41±6.68 | 137.77±60.31 | 7.66±0.57 |
| FS.lxb | 110.74±0.12 | 95.63±0.17 | 2.94 | 20.3 | 4.6±0.1 | 156.66±2.30 | 8.00±6.92 |
| FS.rdb | 116.88±0.1 | 99.58±0.31 | 3.07±0.01 | 21.9 | 4.36±0.15 | 161.33±2.08 | 5.66±4.61 |
| HL.zj | 127.01±0.27 | 111.73±0.28 | 3.40 | 26.4 | 4.10 | 167.33±2.08 | 3.66±0.57 |
| HL.bhg | 136.75±0.27 | 126.61±0.09 | 3.09±0.01 | 23.7 | 5.53±0.49 | 156.00±3.60 | 4.66±0.57 |
| HL.gz | 124.92±0.7 | 115.14±0.44 | 3.19±0.02 | 26.6 | 4.96±0.05 | 162.33±2.08 | 7.66±0.57 |
| SX.rz | 116.93±0.06 | 100.66±0.19 | 3.05±0.01 | 24.2±0.05 | 5.40±0.3 | 118.00±3.00 | 5.00±1.73 |
| YT.lh | 114.55±0.05 | 92.43±0.06 | 3.12±0.01 | 22.1±0.05 | 6.30±0.26 | 91.66±2.08 | 6.33±0.57 |
| YT.tll | 102.65±0.13 | 97.8±0.05 | 3.35 | 19.0±0.05 | 4.10±0.17 | 127.33±0.57 | 6.33±1.52 |
| YT.cc | 107.57±0.07 | 94.03±0.12 | 3.10 | 20.3±0.05 | 4.26±0.11 | 115.33±4.04 | 4.00±1.00 |
| CL.cc | 100.14±0.16 | 92.49±0.1 | 3.44±0.02 | 25.8 | 4.60±0.1 | 156.66±2.30 | 4.66±0.57 |
| CL.lgs | 126.33±0.39 | 117.28±0.36 | 3.56±0.01 | 24.6 | 4.36±0.15 | 161.33±2.08 | 6.33±4.04 |

**Table S3** Removed samples for fungal community analysis

| **Sample name** | **Region** | **Winery** | **Sampling time** | **Reason** |
| --- | --- | --- | --- | --- |
| FM0Da1 | FS | FS.leb (A) | must | unqualified for sequencing |
| FM0Da2 | FS | FS.leb (A) | must | unqualified for sequencing |
| FM0Da3 | FS | FS.leb (A) | must | unqualified for sequencing |
| NM3Db1 | NX | NX.zh (B) | 3 d | unqualified for sequencing |
| NM3Db2 | NX | NX.zh (B) | 3 d | unqualified for sequencing |
| NM3Db3 | NX | NX.zh (B) | 3 d | unqualified for sequencing |
| NM3Da1 | NX | NX.bs (A) | 3 d | abnormal data |
| NM3Da3 | NX | NX.bs (A) | 3 d | abnormal data |
| YM5Db2 | YT | YT.tll (B) | 5 d | abnormal data |
| FM8Da3 | FS | FS.leb (A) | 8 d | abnormal data |
| FM8Db3 | FS | FS.lxb (B) | 8 d | abnormal data |
| FM8Dc2 | FS | FS.rdb (C) | 8 d | abnormal data |
| SM8D1 | SX | SX.rz (A) | 8 d | abnormal data |
| CM8Db1 | CL | CL.lgs (B) | 8 d | abnormal data |

**Table S4** Relative proportion of Marselan fungal must and fermented sample consortium.

| **Genus Name** | **Percentage (%)** | |
| --- | --- | --- |
|  | **must** | **fermented samples** |
| *Aureobasidium* | 24.4 | 24.88 |
| *Alternaria* | 17.29 | 9.16 |
| *Hanseniaspora* | 2.34 | 16.21 |
| *Cladosporium* | 14.82 | 8.78 |
| *Rhodotorula* | 3.17 | 6.14 |
| *Colletotrichum* | 5.84 | 4.59 |
| *Saccharomyces* | 0.01 | 5.32 |
| *Botrytis* | 2.92 | 4.16 |
| *Metarhizium* | 3.12 | 1.98 |
| *Papiliotrema* | 2.49 | 1.16 |
| *Acremonium* | 2.26 | 1.59 |
| *Lasiodiplodia* | 2.21 | 0.93 |
| *Filobasidium* | 1.92 | 0.97 |
| *Aspergillus* | 1.57 | 1.66 |
| *Phoma* | 1.68 | 0.98 |
| Unclassified | 7.97 | 6.57 |
| Others | 5.99 | 4.92 |

**Table S5** Fungal α-diversity of Marselan must **(A)** and fermented samples **(B)** from different regions based on internal transcribed spacer II (ITS2) of rDNA analysis.

**A**

| **Winery** | **Observed species** | **Shannon** | **Simpson** | **chao1** | **ACE** | **Good’s coverage** |
| --- | --- | --- | --- | --- | --- | --- |
| SX | 208.41±1.52 | 3.76±0.22 | 0.84±0.03 | 206.86±2.36 | 210.32±4.16 | 0.99 |
| NX | 237.77±26.33 | 4.24±0.22 | 0.87±0.02 | 239.74±27.94 | 242.06±28.54 | 0.99 |
| CL | 254.42±21.34 | 4.42±0.38 | 0.86±0.04 | 256.49±21.45 | 259.62±22.1 | 0.99 |
| FS | 276.44±16.74 | 4.11±0.47 | 0.83±0.06 | 278.89±16.97 | 282.35±17.24 | 0.99 |
| HL | 276.44±19.26 | 4.4±0.15 | 0.89±0.01 | 277.55±19.31 | 279.74±19.62 | 0.99 |
| YT | 283.44±13.13 | 4.49±0.39 | 0.87±0.05 | 285.59±12.48 | 288.63±12.26 | 0.99 |

**B**

| **Winery** | **Observed species** | **Shannon** | **Simpson** | **chao1** | **ACE** | **Good’s coverage** |
| --- | --- | --- | --- | --- | --- | --- |
| NX | 217.41±28.18 | 3.93±0.34 | 0.84±0.04 | 229.95±30.38 | 227.94±29.61 | 0.9997 |
| SX | 220.54±12.27 | 3.83±0.26 | 0.81±0.02 | 207.18±16.19 | 203.85±16.23 | 0.9996 |
| CL | 243.78±22.6 | 3.98±0.64 | 0.79±0.1 | 257.86±23.5 | 253.13±23.78 | 0.9997 |
| HL | 273.4±25.92 | 4.15±0.46 | 0.85±0.05 | 288.39±27.84 | 285.35±26.24 | 0.9996 |
| FS | 280.16±36.36 | 4.12±0.45 | 0.85±0.05 | 284.1±36.44 | 279.24±36.74 | 0.9997 |
| YT | 281.57±20.73 | 4.39±0.46 | 0.86±0.06 | 299.29±23.75 | 294.56±21.8 | 0.9996 |

**Table S6** Fungal α-diversity of Marselan must **(A)** and fermented samples **(B)** from different wineries based on internal transcribed spacer II (ITS2) of rDNA analysis.

**A**

| **Winery** | **Observed species** | **Shannon** | **Simpson** | **Chao1** | **ACE** | **Good’s coverage** |
| --- | --- | --- | --- | --- | --- | --- |
| SX.rz | 208.33±1.52 | 3.76±0.22 | 0.84±0.03 | 208.78±4.61 | 212.05±5.33 | 0.9997±0.0001 |
| NX.yk | 225.00±22.71 | 4.05±0.26 | 0.86±0.02 | 216.12±21.88 | 218.26±21.39 | 0.9998 |
| NX.bs | 235.66±20.23 | 4.36±0.21 | 0.88±0.03 | 252.92±18.12 | 257.03±17.4 | 0.9997 |
| CL.cc | 243.33±12.89 | 4.43±0.52 | 0.86±0.06 | 242.11±13.03 | 245.73±13.21 | 0.9998 |
| NX.zh | 246.00±16.37 | 4.31±0.11 | 0.88 | 245.8±16.82 | 247.53±17.84 | 0.9998 |
| HL.zj | 256.66±3.21 | 4.23±0.06 | 0.88±0.01 | 258.24±3.44 | 260.31±4.02 | 0.9998 |
| FS.leb | 262.66±2.51 | 4.07±0.32 | 0.82±0.03 | 258.51±2.7 | 260.93±3.2 | 0.9998 |
| CL.lgs | 272.00±5.56 | 4.47±0.32 | 0.85±0.03 | 273.36±6.37 | 276.77±6.97 | 0.9998 |
| FS.lxb | 274.33±7.5 | 3.78±0.59 | 0.79±0.09 | 276.3±8.01 | 280.24±8.47 | 0.9998 |
| YT.cc | 275.33±15.5 | 4.5±0.38 | 0.89±0.03 | 274.01±15.02 | 278.08±14.11 | 0.9997 |
| HL.gz | 282.66±15.37 | 4.5±0.06 | 0.89±0 | 282.08±16.39 | 283.79±17.11 | 0.9998 |
| FS.rdb | 286.33±10.78 | 4.24±0.49 | 0.82±0.07 | 290.94±9.74 | 294.33±8.82 | 0.9997 |
| YT.tll | 291.00±18.35 | 4.48±0.3 | 0.88±0.03 | 292.51±18.3 | 295.2±18.54 | 0.9998 |
| YT.lh | 293.33±12.66 | 4.47±0.14 | 0.89±0.01 | 294.01±12.49 | 296.2±12.09 | 0.9998 |
| HL.bhg | 299.00±4.35 | 4.74±0.17 | 0.9±0.01 | 301.37±5.05 | 304.4±4.67 | 0.9998 |

**B**

| **Winery** | **Observed species** | **Shannon** | **Simpson** | **chao1** | **ACE** | **Good’s coverage** |
| --- | --- | --- | --- | --- | --- | --- |
| NX.yk | 208.33±1.52 | 3.76±0.22 | 0.84±0.03 | 208.78±4.61 | 212.05±5.33 | 0.9997±0.0001 |
| SX.rz | 225.00±22.71 | 4.05±0.26 | 0.86±0.02 | 216.12±21.88 | 218.26±21.39 | 0.9998 |
| NX.bs | 218.66±20.23 | 4.36±0.21 | 0.88±0.03 | 252.92±18.12 | 257.03±17.4 | 0.9997 |
| CL.cc | 243.33±12.89 | 4.43±0.52 | 0.86±0.06 | 242.11±13.03 | 245.73±13.21 | 0.9998 |
| NX.zh | 246.00±16.37 | 4.31±0.11 | 0.88 | 245.8±16.82 | 247.53±17.84 | 0.9998 |
| HL.zj | 248.66±3.21 | 4.23±0.06 | 0.88±0.01 | 258.24±3.44 | 260.31±4.02 | 0.9998 |
| CL.lgs | 252.66±2.51 | 4.07±0.32 | 0.82±0.03 | 258.51±2.7 | 260.93±3.2 | 0.9998 |
| YT.tll | 265.00±5.56 | 4.47±0.32 | 0.85±0.03 | 273.36±6.37 | 276.77±6.97 | 0.9998 |
| FS.lxb | 272.33±7.5 | 3.78±0.59 | 0.79±0.09 | 276.3±8.01 | 280.24±8.47 | 0.9998 |
| YT.cc | 275.33±15.5 | 4.5±0.38 | 0.89±0.03 | 274.01±15.02 | 278.08±14.11 | 0.9997 |
| FS.leb | 282.66±15.37 | 4.5±0.06 | 0.89±0 | 282.08±16.39 | 283.79±17.11 | 0.9998 |
| FS.rdb | 286.33±10.78 | 4.24±0.49 | 0.82±0.07 | 290.94±9.74 | 294.33±8.82 | 0.9997 |
| HL.gz | 291.00±18.35 | 4.48±0.3 | 0.88±0.03 | 292.51±18.3 | 295.2±18.54 | 0.9998 |
| YT.lh | 293.33±12.66 | 4.47±0.14 | 0.89±0.01 | 294.01±12.49 | 296.2±12.09 | 0.9998 |
| HL.bhg | 299.00±4.35 | 4.74±0.17 | 0.9±0.01 | 301.37±5.05 | 304.4±4.67 | 0.9998 |

**Table S7** Adonis and MRPP tests of the Marselan must and fermented sample fungal communities in different wineries of different regions based on weighted UniFrac distance. Significant (*P* <0.05); extremely significant (*P* <0.01).

|  | **Region** | **Adonis** | | **MRPP** | | |  |
| --- | --- | --- | --- | --- | --- | --- | --- |
|  |  | R^2^ | *P* | | R | *P* | |
| **Must** | NX | 0.474 | 0.02 | | 0.253 | 0.012 | |
|  | FS | 0.797 | 0.001 | | 0.466 | 0.007 | |
|  | HL | 0.655 | 0.008 | | 0.383 | 0.007 | |
|  | YT | 0.677 | 0.004 | | 0.372 | 0.003 | |
|  | CL | 0.169 | 0.4 | | -0.003 | 0.4 | |
| **Fermented samples** | NX | 0.139 | 0.036 | | 0.062 | 0.009 | |
|  | FS | 0.356 | 0.001 | | 0.212 | 0.001 | |
|  | HL | 0.134 | 0.053 | | 0.016 | 0.068 | |
|  | YT | 0.384 | 0.001 | | 0.218 | 0.001 | |
|  | CL | 0.072 | 0.163 | | 0.027 | 0.086 | |
